# An assessment of marine, estuarine, and riverine habitat vulnerability to climate change in the Northeast U.S.

**DOI:** 10.1371/journal.pone.0260654

**Published:** 2021-12-09

**Authors:** Emily R. Farr, Michael R. Johnson, Mark W. Nelson, Jonathan A. Hare, Wendy E. Morrison, Matthew D. Lettrich, Bruce Vogt, Christopher Meaney, Ursula A. Howson, Peter J. Auster, Frank A. Borsuk, Damian C. Brady, Matthew J. Cashman, Phil Colarusso, Jonathan H. Grabowski, James P. Hawkes, Renee Mercaldo-Allen, David B. Packer, David K. Stevenson

**Affiliations:** 1 Office of Habitat Conservation, National Marine Fisheries Service, National Oceanic and Atmospheric Administration, Silver Spring, Maryland, United States of America; 2 Habitat and Ecosystem Services Division, Greater Atlantic Regional Fisheries Office, National Marine Fisheries Service, National Oceanic and Atmospheric Administration, Gloucester, Massachusetts, United States of America; 3 ECS, Under contract to the Office of Science and Technology, National Marine Fisheries Service, National Oceanic and Atmospheric Administration, Silver Spring, Maryland, United States of America; 4 Northeast Fisheries Science Center, National Marine Fisheries Service, National Oceanic and Atmospheric Administration, Woods Hole, Massachusetts, United States of America; 5 Office of Sustainable Fisheries, National Marine Fisheries Service, National Oceanic and Atmospheric Administration, Silver Spring, Maryland, United States of America; 6 NOAA Chesapeake Bay Office, National Marine Fisheries Service, National Oceanic and Atmospheric Administration, Annapolis, Maryland, United States of America; 7 Gulf of Maine Coastal Program, U.S. Fish and Wildlife Service, Falmouth, Maine, United States of America; 8 Office of Renewable Energy Programs, Bureau of Ocean Energy Management, Sterling, Virginia, United States of America; 9 Mystic Aquarium & University of Connecticut, Groton, Connecticut, United States of America; 10 Region 3, U.S. Environmental Protection Agency, Wheeling, West Virginia, United States of America; 11 Darling Marine Center, University of Maine, Walpole, Maine, United States of America; 12 Maryland-Delaware-DC Water Science Center, U.S. Geological Survey, Baltimore, Maryland, United States of America; 13 Region 1, U.S. Environmental Protection Agency, Boston, Massachusetts, United States of America; 14 Marine Science Center, Northeastern University, Nahant, Massachusetts, United States of America; 15 Northeast Fisheries Science Center, National Marine Fisheries Service, National Oceanic and Atmospheric Administration, Orono, Maine, United States of America; 16 Milford Laboratory, Northeast Fisheries Science Center, National Marine Fisheries Service, National Oceanic and Atmospheric Administration, Milford, Connecticut, United States of America; 17 James J. Howard Marine Sciences Laboratory, Northeast Fisheries Science Center, National Marine Fisheries Service, National Oceanic and Atmospheric Administration, Highlands, New Jersey, United States of America; Maurice Lamontagne Institute, CANADA

## Abstract

Climate change is impacting the function and distribution of habitats used by marine, coastal, and diadromous species. These impacts often exacerbate the anthropogenic stressors that habitats face, particularly in the coastal environment. We conducted a climate vulnerability assessment of 52 marine, estuarine, and riverine habitats in the Northeast U.S. to develop an ecosystem-scale understanding of the impact of climate change on these habitats. The trait-based assessment considers the overall vulnerability of a habitat to climate change to be a function of two main components, sensitivity and exposure, and relies on a process of expert elicitation. The climate vulnerability ranks ranged from low to very high, with living habitats identified as the most vulnerable. Over half of the habitats examined in this study are expected to be impacted negatively by climate change, while four habitats are expected to have positive effects. Coastal habitats were also identified as highly vulnerable, in part due to the influence of non-climate anthropogenic stressors. The results of this assessment provide regional managers and scientists with a tool to inform habitat conservation, restoration, and research priorities, fisheries and protected species management, and coastal and ocean planning.

## Introduction

Climate change is impacting all aspects of marine ecosystems [1–4]. There has been substantial work to understand the effect of climate change on marine species and communities [5–9]. In comparison, the effect of climate change on marine, estuarine, and riverine habitats is not as well understood. In some cases, significant research has advanced our understanding of the effects of climate change on individual habitats, or a single component of habitat. For example, many studies have investigated shifting thermal habitat and its effects on species distributions [10–15]. Other studies have explored the impacts of projected changes in the climate on living habitats such as corals [16, 17], mollusks [18–21], seagrasses [22–24], and coastal wetlands [25–28]. However, these studies typically focus on a limited number of climate drivers (e.g., temperature, pH), and do not provide a comprehensive assessment of how climate change may affect habitats that support marine, coastal, and diadromous species (i.e., fish, invertebrates, and protected species). Warming waters in rivers, estuaries, and the ocean, in concert with ocean acidification, water column stratification, deoxygenation, and sea level rise (SLR) can interact with one another and with other stressors to cause complex and often unanticipated synergistic climate effects to species and habitats [4, 29–33].

Understanding how climate change will impact habitats across an ecosystem is necessary to inform decisions about habitat conservation, fisheries management, and coastal and offshore planning. Habitats have long been impacted by human activities such as land-use and land-cover change, point and non-point source pollution, dredging and filling, fishing, sand and gravel mining, oil and gas exploration, damming, and shoreline hardening [34–39]. There is a growing understanding that climate change also has affected, and will increasingly affect, riverine, estuarine, and marine habitats. The effects of climate change will exacerbate the vulnerability of species, habitats, and ecosystems that are already under stress from natural and anthropogenic stressors [4, 40–42].

Habitat requirements differ by species, and as climate change affects habitats, the indirect effects on species will be multifaceted [32, 41]. One of the most straightforward examples is the change in species distribution as a result of warming waters [43–45]. The water column is habitat for aquatic species, and most, if not all, have an optimal temperature range [29, 46, 47]; as water temperature increases, the optimal temperature for marine species would generally shift poleward and species distributions would follow.

There are numerous other effects of climate on habitat and thus numerous other indirect effects of climate on species that use those habitats, especially those (e.g., coastal wetlands, shellfish habitats, kelp forests, and corals) which provide important ecosystem services and functions for key life stages of marine and coastal organisms [1, 4, 48]. Over the past century, coastal wetlands have been affected by SLR, contributing to a cumulative loss of habitat [49–54]. Coastal wetland habitat supports juvenile growth and survival for many marine fish species [55] and their prey [56], and the climate-driven impairment of habitat may result in decreased population productivity for sensitive species. Marsh loss also threatens the populations of many species of birds that depend on coastal wetland habitat for breeding, nesting, and wintering [57]. Increasing temperatures may shift the geographic range of kelp beds, with implications for the myriad fish, birds, invertebrates, and marine mammals that they support [58, 59]. Calcifying marine organisms, including mollusks, echinoderms, and corals, are particularly sensitive to changes in pH, carbonate ion concentration, and the saturation state of calcium carbonate minerals–collectively known as ocean acidification [20, 60–63]. Rising temperatures and ocean acidification are negatively impacting shallow and deep sea corals [16, 17, 64]. Loss of live coral cover is related to decreases in abundance of a number of fish species; the magnitude of decline has been associated with the dependence on live coral [64–67].

Although the effects of climate change on living habitats may be more pronounced, the effects on habitats with primarily non-living characteristics (e.g., sand, mud, rock, water column) cannot be overlooked. These habitats serve an important role in the reproduction of several groups of protected species and in the foraging of other species. For example, beaches that pinnipeds use for pupping and sea turtles use for nesting [68] may be affected by increased erosion and inundation from SLR, and the frontal features that aggregate prey for fish, seabirds, and marine mammals in the open ocean may shift in location and strength [69, 70]. SLR is also impacting the intertidal foraging [71] and coastal nesting [72] habitat used by shorebirds and seabirds.

To develop an ecosystem-scale understanding of the effect of climate change on habitats, we conducted a trait-based climate vulnerability assessment for marine, estuarine, and coastal riverine habitats in the Northeast U.S. Shelf Ecosystem, from Cape Hatteras through the Gulf of Maine. The coastal northeastern U.S. and the adjacent continental shelf are warming at a particularly rapid rate [73–75] that is expected to increase [76]. Observed rates of sea level rise in the Northeast have also been higher than the global mean, and are projected to increase [77]. This underscores the importance of understanding how these changes will impact the region’s habitats. A trait-based climate vulnerability assessment is one method used to evaluate the potential risks of climate change to species or ecosystems [78–81]. An expert elicitation process was used to estimate climate sensitivities and exposure, which can provide broad, transparent, and relatively rapid evaluation of the vulnerability of multiple species [80, 82–84], habitats [85, 86], or ecosystems [87]. This approach facilitates assessment of a large geographic area with variability in the availability of data across habitats and space (e.g., habitat range and condition, physical and chemical thresholds for habitat impacts, and limitations in downscaled climate projections for nearshore areas). The main purpose of this assessment is to provide information for scientists and natural resource managers to identify research priorities and improve management decisions for these particular habitats and the species that rely on them. Further, we seek to begin to elucidate the many indirect effects of climate change on species through direct effects on habitats.

## Methods

### Method development

At a NOAA Fisheries workshop in July 2018, habitat specialists and managers reviewed literature of various existing climate vulnerability assessment (CVA) methodologies and decided to base this habitat CVA on a hybrid of the framework developed for NOAA’s Fish Stock Climate Vulnerability Assessment (FSCVA) [81, 88], and a habitat vulnerability model developed for the Northeastern Association of Fish and Wildlife Agencies (NEAFWA) [85]. This hybrid approach adapted elements from each framework to design a methodology that could be applied to the full suite of marine, estuarine, and riverine habitats in the Northeast U.S. Using the overall structure of the NOAA’s FSCVA allowed the results of this assessment to be easily synthesized with the vulnerability of specific species. The NEAFWA methodology provided attributes indicative of the response of terrestrial and non-tidal wetland habitats to climate change, which were adapted to marine, estuarine, and riverine habitats.

This assessment used a trait-based framework that incorporated two components: sensitivity and exposure. The general premise was that the overall climate vulnerability of a habitat is a function of the interplay between the sensitivity and the potential exposure to future change. Many vulnerability assessments also include an adaptive capacity component. However, adaptive capacity and sensitivity in biological systems are confounded; the same trait that infers high sensitivity may be viewed as low adaptive capacity, and vice versa [89]. Therefore, we incorporated adaptive capacity into the sensitivity component.

### Habitat selection

In this assessment, habitat is defined as the coastal rivers, estuaries, and marine waters, from the bottom through the water column including the physical, geological, chemical, and biological components of an ecosystem that a species depends on to complete its life cycle–reproduction, development, growth, and survival [90, 91]. We included 52 habitats based on their importance to NOAA’s trust resources (https://www.fisheries.noaa.gov/region/new-england-mid-atlantic#species) in the Northeast U.S.: 23 marine habitats, 19 estuarine habitats, and 10 riverine habitats (Table 1). Habitats that were present in multiple systems (e.g., submerged aquatic vegetation is present in all three systems) were assessed separately to capture differences in the climate and non-climate stressors on that habitat. Selected habitats were arranged in a hierarchical classification system based on the Federal Geographic Data Committee update [92] to the Cowardin Classification of Wetlands and Deepwater Habitats of the United States [93], with some modifications. For example, categories were included for water column habitats in the riverine, estuarine, and marine systems, which are not present in the Cowardin classification system. The resulting classification system allows for a comparison of the climate vulnerability of habitats across systems, sub-systems, classes, sub-classes, and geographic areas, which can reveal patterns and key drivers of vulnerability.

**Table 1:** Classification of habitats included in the assessment.

System	Sub-system	Class	Sub-class	Common Name	Category	Geographic Area
Marine	Intertidal	Reef	Mollusk	Marine Intertidal Shellfish Reef	Living	Entire Area
Marine	Intertidal	Rocky Bottom	Bedrock, Rubble, Cobble, Gravel	Marine Intertidal Rocky Bottom	Bottom Substrate	Entire Area
Marine	Intertidal	Unconsolidated Bottom	Mud	Marine Intertidal Mud Bottom	Bottom Substrate	Entire Area
Marine	Intertidal	Unconsolidated Bottom	Sand	Marine Intertidal Sand Bottom	Bottom Substrate	Entire Area
Marine	Subtidal	Aquatic Bed	Kelp	Marine Kelp	Living	Entire Area
Marine	Subtidal & Intertidal	Aquatic Bed	Red, Green, Small-brown Algae	Marine Red, Green, Small-brown Algae	Living	Entire Area
Marine	Subtidal & Intertidal	Aquatic Bed	Rooted Vascular	Marine Submerged Aquatic Vegetation	Living	Entire Area
Marine	Subtidal	Reef	Mollusk	Marine Subtidal Shellfish Reef	Living	Entire Area
Marine	Subtidal <200 m	Rocky Bottom	Bedrock, Rubble, Cobble, Gravel	Marine Rocky Bottom <200 m	Bottom Substrate	Entire Area
Marine	Subtidal <200 m	Unconsolidated Bottom	Mud	Marine Mud Bottom <200 m	Bottom Substrate	Entire Area
Marine	Subtidal <200 m	Unconsolidated Bottom	Sand	Marine Sand Bottom <200 m	Bottom Substrate	Entire Area
Marine	Subtidal >200 m	Reef	Deep Sea Coral & Sponge	Deep Sea Coral & Sponge: Gulf of Maine	Living	Gulf of Maine
Marine	Subtidal >200 m	Reef	Deep Sea Coral & Sponge	Deep Sea Coral & Sponge: Seamounts and Canyons	Living	Seamounts & Canyons
Marine	Subtidal >200 m	Rocky Bottom	Bedrock, Rubble, Cobble, Gravel	Marine Rocky Bottom >200 m	Bottom Substrate	Entire Area
Marine	Subtidal >200 m	Unconsolidated Bottom	Mud	Marine Mud Bottom >200 m	Bottom Substrate	Entire Area
Marine	Subtidal >200 m	Unconsolidated Bottom	Sand	Marine Sand Bottom >200 m	Bottom Substrate	Entire Area
Marine	Subtidal & Intertidal	Reef	Mollusk Aquaculture	Marine Shellfish Aquaculture	Artificial	Entire Area
Marine	Subtidal & Intertidal	Rocky Bottom	Artificial Structures	Marine Artificial Structures	Artificial	Entire Area
Marine	Subtidal <20 m	Water Column	Shallow Inner Shelf	Marine Shallow Inner Shelf Water Column	Water Column	Entire Area
Marine	Subtidal <200 m	Water Column	Shelf Surface	Marine Shelf Surface Water Column	Water Column	Entire Area
Marine	Subtidal <200 m	Water Column	Shelf Bottom	Marine Shelf Bottom Water Column	Water Column	Entire Area
Marine	Subtidal >200 m	Water Column	Slope Surface	Marine Slope Surface Water Column	Water Column	Entire Area
Marine	Subtidal >200 m	Water Column	Slope Bottom	Marine Slope Bottom Water Column	Water Column	Entire Area
Estuarine	Intertidal	Emergent Wetland	Invasive Wetland	Estuarine Invasive Wetland: Mid-Atlantic	Invasive	Mid-Atlantic
Estuarine	Intertidal	Emergent Wetland	Invasive Wetland	Estuarine Invasive Wetland: New England	Invasive	New England
Estuarine	Intertidal	Emergent Wetland	Native Wetland	Estuarine Native Wetland: Mid-Atlantic	Living	Mid-Atlantic
Estuarine	Intertidal	Emergent Wetland	Native Wetland	Estuarine Native Wetland: New England	Living	New England
Estuarine	Intertidal	Reef	Mollusk	Estuarine Intertidal Shellfish Reef	Living	Entire Area
Estuarine	Intertidal	Rocky Bottom	Bedrock, Rubble, Cobble, Gravel	Estuarine Intertidal Rocky Bottom	Bottom Substrate	Entire Area
Estuarine	Intertidal	Rocky Bottom	Artificial Structures	Estuarine Intertidal Artificial Structures	Artificial	Entire Area
Estuarine	Intertidal	Unconsolidated Bottom	Mud	Estuarine Intertidal Mud Bottom	Bottom Substrate	Entire Area
Estuarine	Intertidal	Unconsolidated Bottom	Sand	Estuarine Intertidal Sand Bottom	Bottom Substrate	Entire Area
Estuarine	Subtidal	Aquatic Bed	Kelp	Estuarine Kelp	Living	Entire Area
Estuarine	Subtidal & Intertidal	Aquatic Bed	Red, Green, Small-brown Algae	Estuarine Red, Green, Small-brown Algae	Living	Entire Area
Estuarine	Subtidal & Intertidal	Aquatic Bed	Rooted Vascular	Estuarine Submerged Aquatic Vegetation	Living	Entire Area
Estuarine	Subtidal	Reef	Mollusk	Estuarine Subtidal Shellfish Reef	Living	Entire Area
Estuarine	Subtidal	Rocky Bottom	Bedrock, Rubble, Cobble, Gravel	Estuarine Subtidal Rocky Bottom	Bottom Substrate	Entire Area
Estuarine	Subtidal & Intertidal	Reef	Mollusk Aquaculture	Estuarine Shellfish Aquaculture	Artificial	Entire Area
Estuarine	Subtidal	Rocky Bottom	Artificial Structures	Estuarine Subtidal Artificial Structures	Artificial	Entire Area
Estuarine	Subtidal	Unconsolidated Bottom	Mud	Estuarine Subtidal Mud Bottom	Bottom Substrate	Entire Area
Estuarine	Subtidal	Unconsolidated Bottom	Sand	Estuarine Subtidal Sand Bottom	Bottom Substrate	Entire Area
Estuarine	Subtidal	Water Column	Well-mixed	Estuarine Water Column	Water Column	Entire Area
Riverine	Non-tidal	Emergent Wetland	Invasive Wetland	Riverine Non-tidal Invasive Wetland	Invasive	Entire Area
Riverine	Non-tidal	Emergent Wetland	Native Wetland	Riverine Non-tidal Native Wetland	Living	Entire Area
Riverine	Tidal	Emergent Wetland	Invasive Wetland	Riverine Tidal Invasive Wetland	Invasive	Entire Area
Riverine	Tidal	Emergent Wetland	Native Wetland	Riverine Tidal Native Wetland	Living	Entire Area
Riverine	Tidal & Non-Tidal	Aquatic Bed	Algae	Riverine Algae	Living	Entire Area
Riverine	Tidal & Non-Tidal	Aquatic Bed	Rooted Vascular	Riverine Submerged Aquatic Vegetation	Living	Entire Area
Riverine	Tidal & Non-Tidal	Rocky Bottom	Bedrock, Rubble, Cobble, Gravel	Riverine Rocky Bottom	Bottom Substrate	Entire Area
Riverine	Tidal & Non-Tidal	Unconsolidated Bottom	Mud	Riverine Mud Bottom	Bottom Substrate	Entire Area
Riverine	Tidal & Non-Tidal	Unconsolidated Bottom	Sand	Riverine Sand Bottom	Bottom Substrate	Entire Area
Riverine	Tidal & Non-Tidal	Water Column	Well-mixed	Riverine Water Column	Water Column	Entire Area

See S1 File for a more detailed description of each habitat.

Definitions were developed for features and living and non-living characteristics of each habitat (S1 File). In order to explore patterns in the results, we also categorized the habitats into bottom substrate, living, water column, artificial structures, and invasive species. While each habitat was assigned to a single category based on its defining characteristics, some habitats could fit in multiple categories. For example, sand and mud bottom substrates also include living components (e.g., bivalve and gastropod infauna and epifauna communities), and invasive species (e.g., *Phragmites australis*) are also living habitats.

For most habitats, we assessed climate vulnerability across the full geographic range of the study area, with three exceptions: estuarine emergent native wetland, estuarine emergent invasive wetland, and deep sea coral & sponge. Estuarine emergent wetlands (both native and invasive sub-classes) were assessed separately in the Mid-Atlantic and New England because biogeographic differences in coastal wetlands and/or the rate of SLR could result in differential climate vulnerabilities for this habitat. Deep sea coral and sponge habitats were split between the Gulf of Maine and the areas farther offshore on the Northeast U.S. Continental Shelf, slope, submarine canyons, and seamounts to assess potential differences in climate vulnerability associated with coral and sponge habitat density, depth, biodiversity, population genetics, and anthropogenic drivers such as impacts from fishing gear [65, 94–98].

In addition, sub-systems, classes, and sub-classes were differentiated to capture expected differences in sensitivity or exposure to climate change within the region. For example, emergent wetlands in tidal and non-tidal portions of the riverine system were assessed separately to evaluate the potential effects of changes in salinity and/or SLR on these two habitat types. Similarly, emergent wetland habitats for both the estuarine and riverine systems were further divided into native (e.g., *Spartina* spp.) and invasive species (e.g., non-native genotype of *Phragmites australis*). Although other prominent invasive plant species occur in the Northeast U.S. region, their populations are not large enough to form a distinct habitat type like the dense and pervasive stands of *Phragmites australis* present in estuaries and tidal and non-tidal portions of rivers and streams in the region. In the marine system, artificial reefs and intertidal and subtidal artificial hard bottoms (e.g., riprap for shoreline protection) were assessed under a single artificial category. However, because of the prevalence of hardened shoreline structures in the estuarine system, intertidal and subtidal artificial structures were assessed separately. All river and stream habitats used by diadromous species were included in the assessment, and were not differentiated by stream order.

### Sensitivity

The sensitivity of habitats to climate change was evaluated using nine sensitivity attributes (Table 2 and S2 File) covering a diverse range of traits indicative of how a habitat will respond to future changes in climate. For example, Habitat Condition and Habitat Fragmentation both reflect the ability of a habitat to support a natural and fully-functioning ecological community of organisms, while Mobility/Ability to Spread or Disperse, Resilience, and Sensitivity to Changes in Abiotic Factors are attributes that measure how well a habitat may respond to changes in climate. Sensitivity and Intensity of Non-climate Stressors was included to assess the effects of a suite of anthropogenic stressors on a habitat, because such stressors have the potential to reduce the ecological function and the ability of habitats to withstand climate-related stressors [4, 40–42]. This attribute includes non-climate stressors that may affect habitats in riverine (e.g., dams, water diversions), estuarine (e.g., navigational dredging, eutrophication, shoreline hardening), and marine (e.g., bottom-tending fishing gear, ocean energy development) systems. To ensure consistency and repeatability in the application of these sensitivity attributes, a written definition and scoring bins were developed for each attribute. The scoring bins characterize Low, Moderate, High, and Very High scores for each sensitivity attribute. The assessment used a five-tally scoring method, as described in the FSCVA framework, where each scorer could distribute their five tallies between any of the scoring bins to best describe the uncertainty and/or the geographic variability within the study area. Whereas it is possible that some of these sensitivity attributes may have a stronger effect on vulnerability than others, this relationship is unknown and may differ between habitats; therefore, the sensitivity attributes were all given equal weightings.

**Table 2:** Sensitivity attributes.

Sensitivity Attribute	Assessment Definition
Habitat Condition	The ability of the habitat to support a natural, fully-functional ecological community of organisms and the associated/expected ecosystem services.
Habitat Fragmentation	The extent to which a previously contiguous habitat is subdivided into isolated patches or fragments due to anthropogenic causes.
Distribution and Range	The historic geographic extent of a habitat, including the leading (i.e., the expanding or colonizing) edge and trailing (i.e., contracting or declining) edge, if applicable, and the water depths for which the habitat naturally occurs.
Mobility/Ability to Spread or Disperse	The ability or capability of a habitat to disperse, move, or spread to areas beyond its existing location.
Resistance	The ability of a habitat to tolerate a stressor and persist while retaining its functionality when subjected to a disturbance.
Resilience	The ability of, and the time period for, a habitat to recover from a disturbance.
Sensitivity to Changes in Abiotic Factors	A measure of a habitat’s ability to tolerate changes in chemical and physical characteristics of the environment (temperature, salinity, dissolved oxygen, carbonate chemistry, and synergistic effects).
Sensitivity and Intensity of Non-Climate Stressors	A measure of a habitat’s response to existing non-climate stressors, as well as the intensity of those stressors (dredging/filling, pollution/eutrophication, invasive species, harmful algal blooms, shoreline hardening, and synergistic effects).
Dependency on Critical Ecological Linkages	The extent to which a habitat depends upon associated species to maintain its health or function as a habitat.

See S2 File for more detailed descriptions of the sensitivity attributes.

### Climate exposure

We used eleven equally weighted exposure factors to indicate the magnitude and overlap of projected changes in climate with the distribution of habitats (Table 3 and S3 File). This methodology uses a wide range of exposure factors, any of which could increase the vulnerability of a habitat, instead of focusing on just a single variable (e.g., temperature). We used this approach because individual habitats are likely to respond differently to the various changes that are anticipated with climate change. This multiple stressor approach also allowed us to identify potential cumulative or compounding impacts of multiple exposure factors. It is important to note that not all exposure factors directly impact each of these habitats. Only the factors that were applicable to each habitat were scored. For example, exposure to SLR was scored for coastal, shallow-water habitats (e.g., emergent wetlands, intertidal mud and sand), but not offshore habitats (e.g., deep sea corals and sponges). Multiple temperature (surface, bottom, air, and river) and salinity (surface and bottom) factors were included to account for variation in conditions by depth and location. To avoid double counting within each habitat, only the most appropriate temperature or salinity exposure factor was scored.

**Table 3:** Exposure factors.

Exposure Factor	Projection / Source
Sea Surface Temperature	Northwest Atlantic Regional Ocean Modeling System
Bottom Temperature	Northwest Atlantic Regional Ocean Modeling System
Sea Surface Salinity	Northwest Atlantic Regional Ocean Modeling System
Bottom Salinity	Northwest Atlantic Regional Ocean Modeling System
pH	Coupled Model Intercomparison Project Phase 5
Precipitation	Coupled Model Intercomparison Project Phase 5
Air Temperature	Coupled Model Intercomparison Project Phase 5
Streamflow (Floods and Droughts)	Demaria et al. (2016)
River Temperature	Letcher et al. (2016)
Sea Level Rise	Sweet et al. (2017)

See S3 File for more detailed descriptions of the Streamflow, River Temperature, and Sea Level Rise exposure factors.

The selected exposure factors represent the main climate-driven impacts to the function and viability of habitats in the Northeast U.S. For most of the exposure factors, estimates of projected change were taken from NOAA’s Ocean Climate Change Web Portal (https://psl.noaa.gov/ipcc/). Projections were based on the Intergovernmental Panel on Climate Change, Representative Concentration Pathway 8.5 (RCP 8.5), which represents a scenario that assumes little to no stabilization of greenhouse gas emissions over the time horizon for the assessment [99, 100]. We used the results from the downscaled Northwest Atlantic Regional Ocean Modeling System (ROMS-NWA) for ocean temperature and salinity exposure factor projections. Precipitation, air temperature (which was used as a proxy for water temperature in intertidal habitats), and pH were taken from the Coupled Model Intercomparison Project Phase 5 (CMIP5) global model results. For precipitation, experts considered the projected change in the annual mean from CMIP5 as well as information on the projected change in the frequency and intensity of extreme precipitation events (S3 File). Reproducing the method used in NOAA’s previous CVAs [81–83, 101], scoring of these model-derived exposure factors was based on a comparison of the future modeled mean to the historic variability as a change in standard deviations. However, unlike these previous CVAs, which were based on mid-century projections, the end-of-century time frame was applied in this study because the structures in many large-scale projects that may impact fish habitats (e.g., bridges and roads, hydropower dams, and major coastal, offshore, and urban development) can be in place over a 50 to 75-year time horizon [38]. In addition, for temporal scales less than 50 years, natural variability contributes substantial uncertainty in climate projections over local and regional spatial scales [102, 103]. For example, the change in global mean sea surface temperature for the first half of the 21^st^ century is similar under most emission scenarios and increasingly diverges later in the century [104]. The end-of-century time frame for ROMS-NWA is 2070–2099, and for CMIP is 2050–2099. The exposure scoring bins from the FSCVA were scaled to appropriately capture the greater changes expected by the end of the century, and to ensure comparability with the species vulnerability assessments.

Projections for SLR were based on the 1.0 m (Intermediate) mean global SLR scenario for 2100 [77], which resulted in a range of SLR between 1.2 and 1.4 m for the study area. Because other projections (ROMS, CMIP) present mean change over a broader period of time (i.e., the latter half of the 21st century), we chose a SLR projection matching the midpoint of the broader projections. The exposure score for SLR was based on both the magnitude of change in sea level and spatial overlap of the relevant habitats with projected change in sea level (S3 File). For example, intertidal habitats were assumed to have greater exposure to SLR than shallow water subtidal habitats.

Because the climate drivers in the riverine system are very different from those in the marine and estuarine systems, we developed a suite of additional exposure factors that are unique to riverine habitats. For example, river flow rates are driven by a complex array of factors (e.g. precipitation, evapotranspiration, groundwater contributions, land use, and anthropogenic water management) [105], such that precipitation alone is not an adequate proxy to capture the impact of climate change on stream and river flow. Therefore, we developed a scoring rubric for flooding and droughts based on a suite of regional streamflow projections [106] (S3 File).

Similarly, air temperature is not a linear predictor of river temperature, which is also influenced by landscape and environmental drivers such as riparian cover, snow melt, and groundwater [107]. River temperature projections were based on data retrieved from the U.S. Geological Survey’s (USGS) Spatial Hydro-Ecological Decision System (SHEDS) Stream Temperature Model [107]. Using the +4°C air temperature scenario (an approximation of the end-of-century projections in the Northeast U.S.), we used the projected mean summer stream temperature, historic mean summer stream temperature, and the variability of the historic mean stream temperature to develop scoring bins similar to our modeled climate projections for other exposure factors. These data were aggregated by the USGS Watershed Boundary Dataset 6-digit hydrological unit code (HUC6) basins and displayed on a map color coded with the appropriate scoring bins (S3 File). The SHEDS model includes temperature data for first, second, and third order streams only, largely due to greater potential influence of non-climate anthropogenic activities on temperatures in larger rivers. For the purposes of this assessment, we used the aggregated HUC6 stream temperature data for all riverine habitats.

### Scoring protocol

The scoring for this assessment was based on the protocol described in the FSCVA framework [88]. Fifteen experts participated in the sensitivity scoring, with five experts scoring each of the three systems (marine, estuarine, and riverine). Experts from academia, state, and federal institutions were selected based on recommendations from regional NOAA Fisheries and fishery management council staff to cover a range of geographic and habitat expertise. We conducted a pilot scoring with a subset of scorers and habitats to gather feedback on the process, resulting in improvements in the guidance materials and sensitivity attribute definitions. Scoring was a two-step process similar to the Delphi approach [108, 109]. Experts were first given the opportunity to independently score each sensitivity attribute for each habitat based on the Sensitivity Attribute Definitions (S2 File), a synthesis of information and literature for each habitat provided to the experts in “habitat profiles”, and their own knowledge of the habitat. In addition, the experts assigned a data quality score for the sensitivity attributes for each habitat to reflect the availability of information and uncertainty (Table 4). At the end of this initial round, the scores from each expert were compiled and analyzed for discussion. A second round of scoring was completed at a facilitated workshop in February 2020, where the fifteen experts discussed their individual scores and were given the opportunity to adjust their scores based on group discussions. Many of the habitats were closely related between systems (e.g., intertidal rocky bottom in the marine and estuarine systems), so experts from other systems were encouraged to participate in the discussions for similar habitats. This two-step scoring process necessitated each expert to develop their own scores based on the materials provided and their expert opinion, and then leverage the knowledge and expertise of the other experts shared at the facilitated workshop to inform their final scores. This process alleviates geographic bias or differing interpretations of the materials used during scoring. However, experts were not compelled to change their scores, as consensus was not the aim.

**Table 4:** Data quality matrix.

Data Quality Score	Description
3	Adequate Data. The score is based on data which have been observed, modeled or empirically measured for the habitat in question and come from a reputable source.
2	Limited Data. The score is based on data which has a higher degree of uncertainty. The data used to score the attribute may be based on related or similar habitat, come from outside the study area, or the reliability of the source may be limited.
1	Expert Judgment. The attribute score reflects the expert judgment of the reviewer and is based on their general knowledge of this attribute for the habitat or a related habitat.
0	No Data. No information to base an attribute score on. Very little is known about this attribute for this habitat, and there is no basis for forming an expert opinion (to be used judiciously).

Criteria used in sensitivity and exposure scoring to identify quality and availability of data.

The exposure factors for all habitats were scored by five experts with experience using climate projections in vulnerability assessments. Maps depicting the distribution of each habitat were generated primarily from the Northeast Data Portal (https://www.northeastoceandata.org/) and the Marine Cadastre (https://marinecadastre.gov/). For habitats whose distribution was not well mapped in the study area (e.g., red, green, and small brown algae), or occurred at too fine a scale for spatial comparison with climate projections (e.g., riverine bottom substrate), experts relied on a textual description of the habitat’s distribution. Independently, each climate exposure expert visually integrated the habitat distribution maps with the climate projection maps and provided a score for each exposure factor based on the overlap between the habitat distribution and the magnitude of the change in exposure. If an exposure factor was not directly applicable to a habitat (e.g., river temperature is not directly relevant to offshore habitats), the factor was not scored. Each exposure factor was also given a data quality score by each expert to reflect any perceived uncertainties based on the availability and resolution of habitat mapping and the resolution of the modeled climate exposure projections. The resulting exposure scores were compiled and analyzed for discussion with the other scorers. The second round of exposure scoring was completed over a series of webinars where differences in scoring and nuances in the projections and habitat maps were discussed. Experts were then given an opportunity to adjust their scores based on these discussions.

### Calculating vulnerability ranks

Overall vulnerability ranks for each habitat were calculated in a three-step process developed in the FSCVA framework. The scoring bins for the sensitivity attributes and exposure factors were assigned a numerical value (Low = 1, Moderate = 2, High = 3, Very High = 4). A weighted mean for all the tallies across all expert’s scores was calculated for each attribute and factor. These attribute and factor means were rolled up into a component score by applying the logic model described in Table 5. The purpose of this logic model is to identify the attributes of highest importance for a habitat. Averages were not used to calculate component scores because the low scores tend to devalue important drivers of climate vulnerability. Note that the criteria for the Very High component score is a difficult threshold to achieve and was designed to indicate that the habitat has multiple very large climate vulnerability drivers. Finally, the component scores were multiplied to provide a categorical vulnerability rank classified as: 1–3 low, 4–6 moderate, 8–9 high, and 12–16 very high.

**Table 5:** Logic rule for calculating climate exposure and sensitivity for each habitat type.

Logic Rule	Component Score	Habitat Sensitivity and Exposure Categorical Rank
3 or more attribute or factor means ≥ 3.5	4	Very High
2 or more attribute or factor means ≥ 3.0	3	High
2 or more attribute or factor means ≥ 2.5	2	Moderate
All other scores	1	Low

The scoring rubric is based on a logic model where a certain number of individual scores above a certain threshold are used to determine the overall climate exposure and sensitivity (Hare et al. 2016).

This methodology utilizes discrete scoring thresholds at several stages while calculating overall vulnerability rank. To detect borderline cases, where the change in just a few tallies in one or more attributes could change the overall vulnerability rank, we conducted a bootstrap analysis for each habitat. The tallies for every attribute and factor were resampled, with replacement, which were then used to recalculate the attribute means, component scores, and vulnerability ranks. Each outcome was recorded and the process repeated 5,000 times. The proportion of the outcomes in each bootstrap vulnerability rank was then compared to the categorical vulnerability rank. This analysis helped identify borderline cases where there was a significant likelihood the habitat could have a higher or lower vulnerability rank. In addition, a leave-one-out sensitivity analysis was performed by leaving out the score for each sensitivity attribute or exposure factor to identify its influence on the overall vulnerability rank across habitats. The same analysis was performed to identify the influence of each scorer on the vulnerability ranks for the system they scored.

An additional analysis was performed on the riverine habitats based on feedback that the classification system may have obscured key interdependencies between riverine habitats. As part of this additional analysis, the individual tallies of the exposure and sensitivity from the riverine water column were combined with the tallies for riverine aquatic bed (algae and submerged aquatic bed), and streambed and bank rocky, sand, and mud bottom habitats. New sensitivity and exposure component scores were calculated based on these combined bed and water column tallies, then the logic model was applied to develop combined vulnerability ranks.

### Direction of climate effect

After completing the final round of scoring in the February 2020 workshop, the habitat experts were queried on what the overall effect of climate change would be for each habitat (i.e., positive, neutral, or negative). As an example, positive climate effects could include expansion of range, improved condition of the habitat, or reduced habitat fragmentation. Negative climate impacts could include a contraction of range, reduced condition of the habitat, increased fragmentation, or a loss of critical ecological linkages. Scoring was completed similarly to the sensitivity attribute scoring ‒ each expert was given four tallies to spread amongst three scoring bins. The tallies for each habitat were summed across all experts’ scores, and summary statistics were calculated. Positive tallies received a value of 1, neutral received 0, and negative received -1. If the weighted average of the tallies was greater than 0.5, the habitat was classified as likely to be positively affected by climate change; conversely, if the value was less than -0.5, the habitat was classified as likely to be negatively impacted by climate change.

### Habitat narratives

Summaries of the assessment results and information used to score each habitat’s climate vulnerability were compiled into habitat narratives (S4 File). These narratives may be informative to end users, as they identify the details of the important drivers of climate sensitivity, exposure, and vulnerability ranks, the key data gaps, relevant non-climate stressors, and overall climate vulnerability of each habitat.

## Results

### Overall habitat climate vulnerability

**Fig 1:**
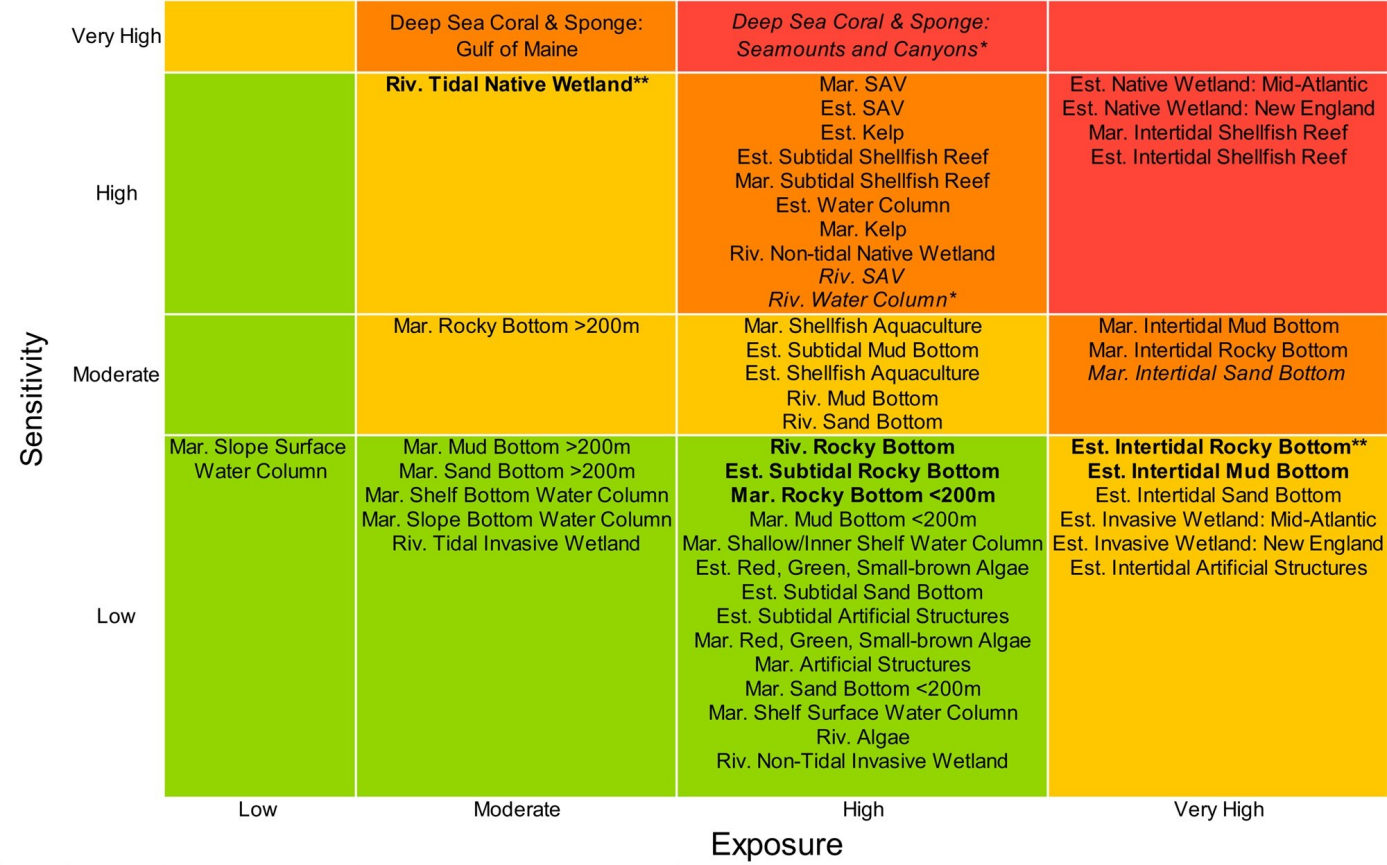
Overall climate vulnerability matrix. Overall climate vulnerability rank is denoted by color: low (green), moderate (yellow), high (orange), and very high (red). Mar = Marine; Est = Estuarine; Riv = Riverine. Categorical ranks used for overall habitat vulnerability in the matrix, and order within each vulnerability cell based on bootstrap rank. Borderline cases from bootstrap results indicated with bold and italics: **bold** ≥0.25 probability that the habitat’s vulnerability rank is one rank higher than the categorical rank; *italics* ≥0.25 probability that the habitat’s vulnerability rank is one rank lower than the categorical rank; *bootstrap analysis found the greatest probability that the habitat’s vulnerability rank is one rank lower than the categorical rank; **bootstrap analysis found the greatest probability that the habitat’s vulnerability rank is one rank higher than the categorical rank.

The climate vulnerability ranks for the 52 habitats spanned from Low to Very High (Fig 1 and S5 File). The Low vulnerability rank contained the largest number of habitats (20 habitats; 38%), followed by High (14 habitats; 27%), Moderate (14 habitats; 27%), and Very High (five habitats; 10%).

**Fig 2:**
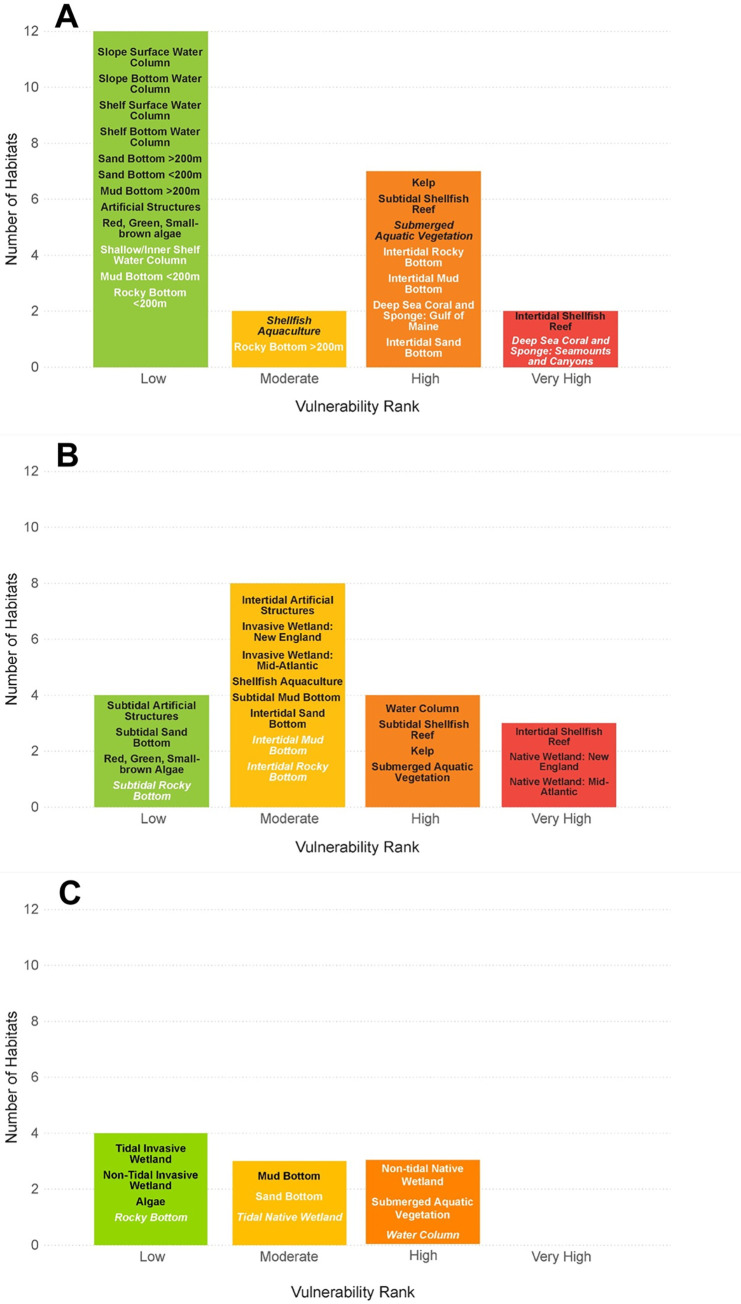
Climate vulnerability in the marine, estuarine, and riverine systems. Number of habitats in the marine (A), estuarine (B), and riverine (C) systems with low, moderate, high, and very high vulnerability ranks. Certainty in rank is denoted by text font and text color: very high certainty (>95%, black font), high certainty (90–95%, black, italic font), moderate certainty (66–90%, white font), low certainty (<66%, white, italic font).

**Fig 3:**
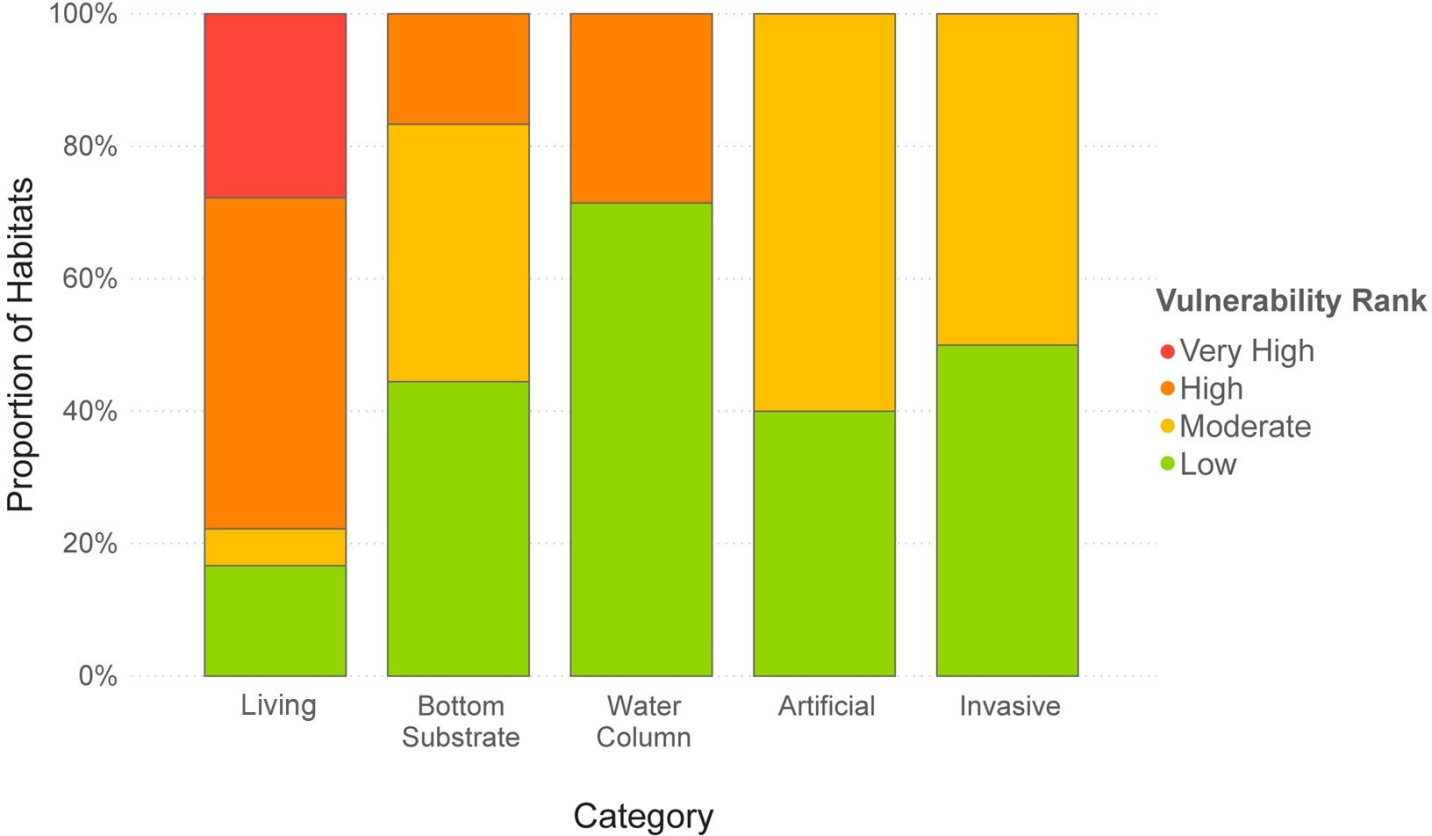
Relative climate vulnerability by category. Proportion of habitats in each category with low, moderate, high, and very high vulnerability ranks.

The marine system had the highest proportion of habitats with High or Very High vulnerability ranks (nine habitats, 39%), followed by seven habitats (37%) in the estuarine system, and three habitats (30%) in the riverine system (Fig 2). The five habitats receiving Very High vulnerability ranks were all living habitats in the marine and estuarine systems. Fifteen of the top twenty most vulnerable habitats were in the living category according to the categorical ranking (Fig 3). Habitats in the artificial structures and invasive categories generally had the lowest vulnerability ranks, none of which ranked greater than Moderate.

The supplemental riverine analysis that combined riverine water column with other riverine habitats yielded two differences from the original results (Table 6). First, when combined with water column scores, riverine rocky bottom changed from Low to Moderate sensitivity and vulnerability. Second, riverine submerged aquatic vegetation changed from High to Moderate sensitivity and vulnerability. The change for submerged aquatic vegetation is counterintuitive given that both riverine water column and submerged aquatic vegetation had High vulnerability ranks in the original analysis, but can be explained by the way the logic model is used to calculate vulnerability. Specifically, different attributes drove the High sensitivity ranks for the two individual habitats such that when combined, the component scores no longer met the threshold for a High sensitivity rank.

**Table 6:** Supplemental riverine analysis.

Riverine Habitat Combinations	Rank		
	Sensitivity	Exposure	Vulnerability
Algae + water column	Low	High	Low
Submerged aquatic vegetation + water column	Moderate	High	Moderate
Rocky bottom + water column	Moderate	High	Moderate
Mud + water column	Moderate	High	Moderate
Sand + water column	Moderate	High	Moderate

### Sensitivity

**Fig 4:**
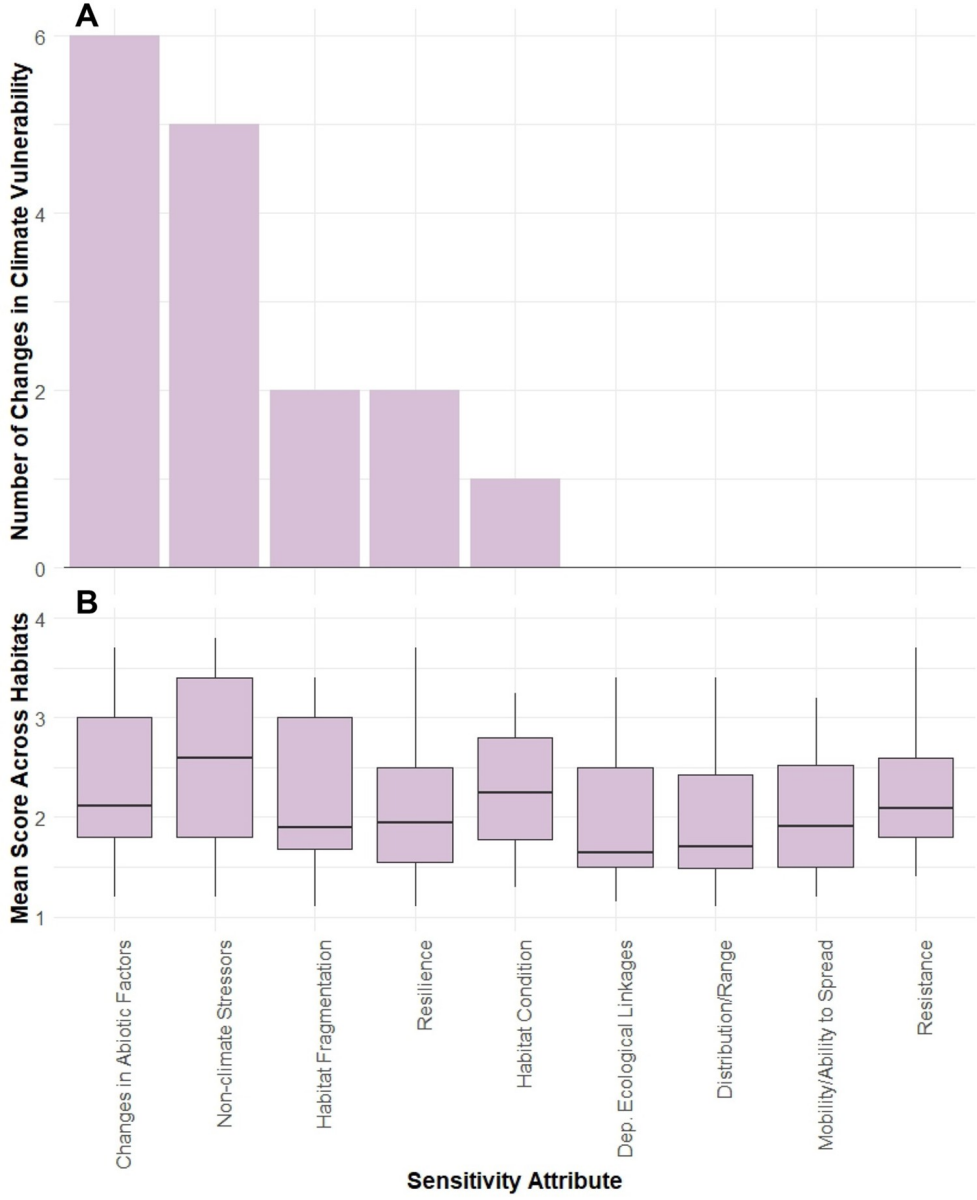
Sensitivity attributes. Results of sensitivity analysis for the influence of individual sensitivity attributes on overall vulnerability rank (A) and mean sensitivity scores across all habitats [boxes represent median and interquartile range (IQR), whiskers are values within 1.5*IQR, and points are potential outliers] (B).

Sensitivity ranks for habitats ranged from Low to Very High. One half (26) of the habitats were ranked as Low, 15 were High (29%), nine were Moderate (17%), and only two were Very High (4%). The two habitats with Very High sensitivity ranks (deep sea coral and sponge habitats for both Gulf of Maine and the seamounts and canyons) had multiple High to Very High mean sensitivity attribute scores. The sensitivity attributes that had the strongest influence on climate vulnerability were Sensitivity to Changes in Abiotic Factors and Sensitivity and Intensity of Non-Climate Stressors (Fig 4A). Removal of the Sensitivity to Changes in Abiotic Factors attribute in the leave-one-out sensitivity analysis changed the vulnerability ranks of six habitats, and removal of the Sensitivity and Intensity of Non-Climate Stressors attribute changed the vulnerability ranks of five habitats. Removal of Resilience and Habitat Fragmentation each changed the vulnerability ranks of two habitats. Due to the way the logic model calculates categorical ranks, in every case where the leave-one-out sensitivity analysis changed the vulnerability of a habitat, the vulnerability went down by one rank. Habitat Condition scored High for many of the marine and estuarine nearshore and riverine living habitats (Fig 4B). Dependency on Critical Ecological Linkages generally had the lowest scores of all the sensitivity attributes. Scorers debated the importance and meaning of this attribute, which may have contributed to its low scores. A sensitivity analysis to identify the influence of individual scorers found that no scorers had outsized influence on the vulnerability ranks. Two scorers had slightly greater influence than others, and removal of those two scorers only changed the vulnerability ranks of three habitats each. Overall, the leave-one-out analysis for scorers increased the vulnerability in 13 habitats and decreased the vulnerability in seven (each by one rank).

### Climate exposure

**Fig 5:**
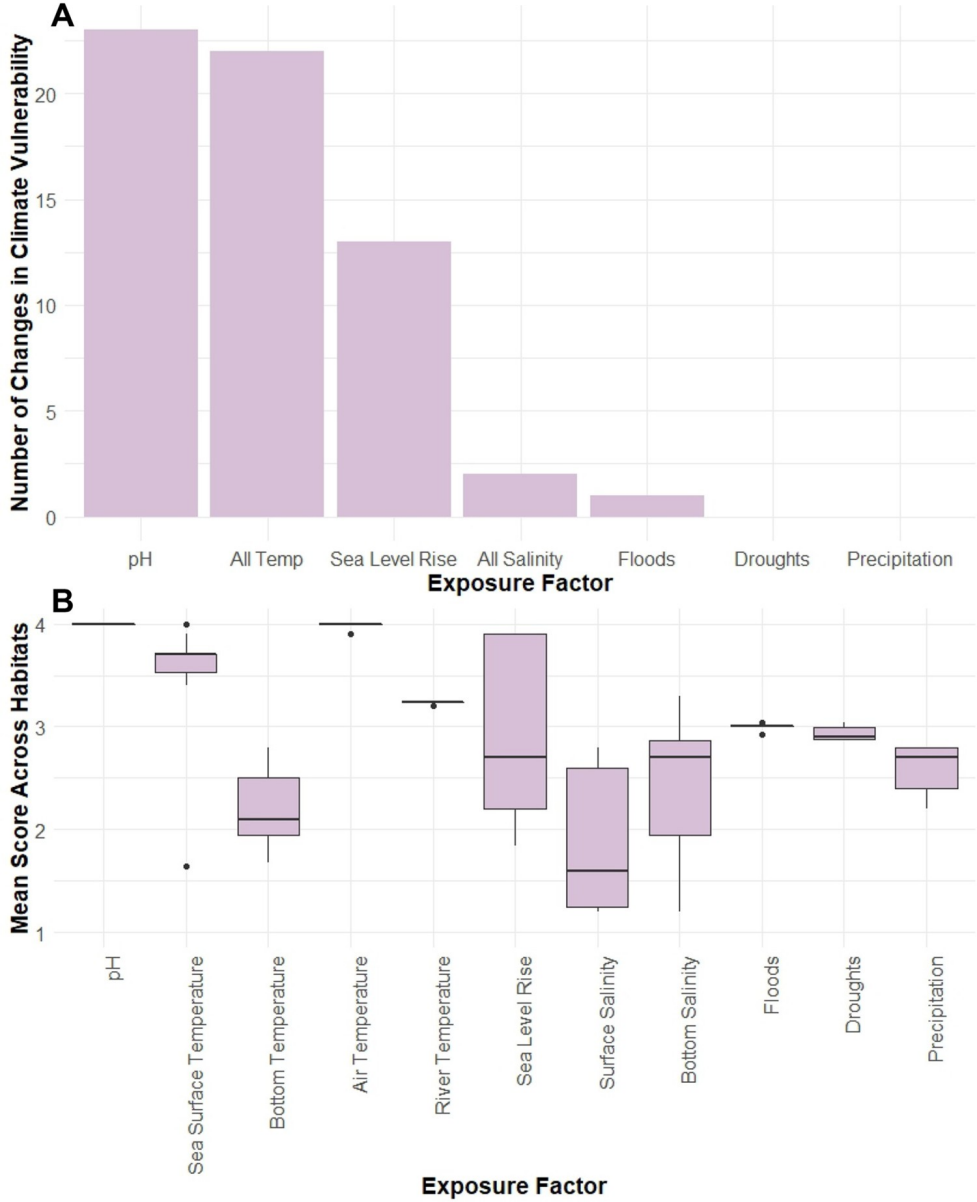
Exposure factors. Results of sensitivity analysis for the influence of individual exposure factors on overall vulnerability rank (A) and mean exposure scores across all habitats (boxes represent median and interquartile range (IQR), whiskers are values within 1.5*IQR, and points are potential outliers) (B). All four Temperature exposure factors (Air, Surface, Bottom, River) were analyzed together in the sensitivity analysis, since only one temperature exposure factor was scored for each habitat. The same was done for the two Salinity exposure factors (Surface, Bottom).

Overall, the climate exposure ranks for habitats tended to be higher than their sensitivity ranks; 37 of the 52 habitats had higher exposure ranks, whereas only three had higher sensitivity ranks (12 had the same sensitivity and exposure ranks). Although exposure ranks ranged from Low to Very High, most of the habitats received a High rank (30; 58%), followed by Very High (13; 25%), Moderate (8; 15%), and Low (1; 2%). The exposure factors that had the strongest influence on climate vulnerability were pH, Temperature (Air, Surface, Bottom, and River), and SLR (Fig 5A). Removal of pH in the leave-one-out sensitivity analysis changed the vulnerability ranks of 72% of the habitats for which that exposure factor was scored (23 of 32 habitats); removal of Temperature exposure factors changed the vulnerability ranks of 42% of habitats for which those exposure factors were scored (22 of 52 habitats); and removal of SLR changed the vulnerability ranks of 65% of the habitats for which that exposure factor was scored (13 of 20 habitats). In the riverine system, removal of Floods changed the vulnerability rank of one habitat, and removal of Floods and Droughts together changed the vulnerability rank of five habitats (50%). Precipitation was not highly influential for any of the exposure ranks of all three systems. Bottom temperature had the lowest overall scores across all of the temperature exposure factors (Fig 5B). The sea level rise and two salinity exposure factors had a large spread, reflecting spatial variability in the study area.

### Bootstrap analysis

For the majority of habitats, the bootstrap outcomes matched the categorical vulnerability ranks calculated from the experts’ tallies. However, the bootstrap analysis identified ten habitats that had more than 25% of the outcomes in a higher or lower vulnerability rank, and all but two were influenced primarily by the exposure scores. This finding indicated that the climate vulnerability of these habitats are on the borderline between vulnerability ranks. Four of these habitats had greater than 50% of the bootstrap outcomes in a different rank, indicating an extreme case of borderline categorical rank (i.e., deep sea coral and sponge: seamounts and canyons, riverine water column, estuarine intertidal rocky bottom, and riverine tidal native wetland). The habitat narratives discuss the specific details for each of these cases (S4 File).

### Direction of climate effect

**Fig 6:**
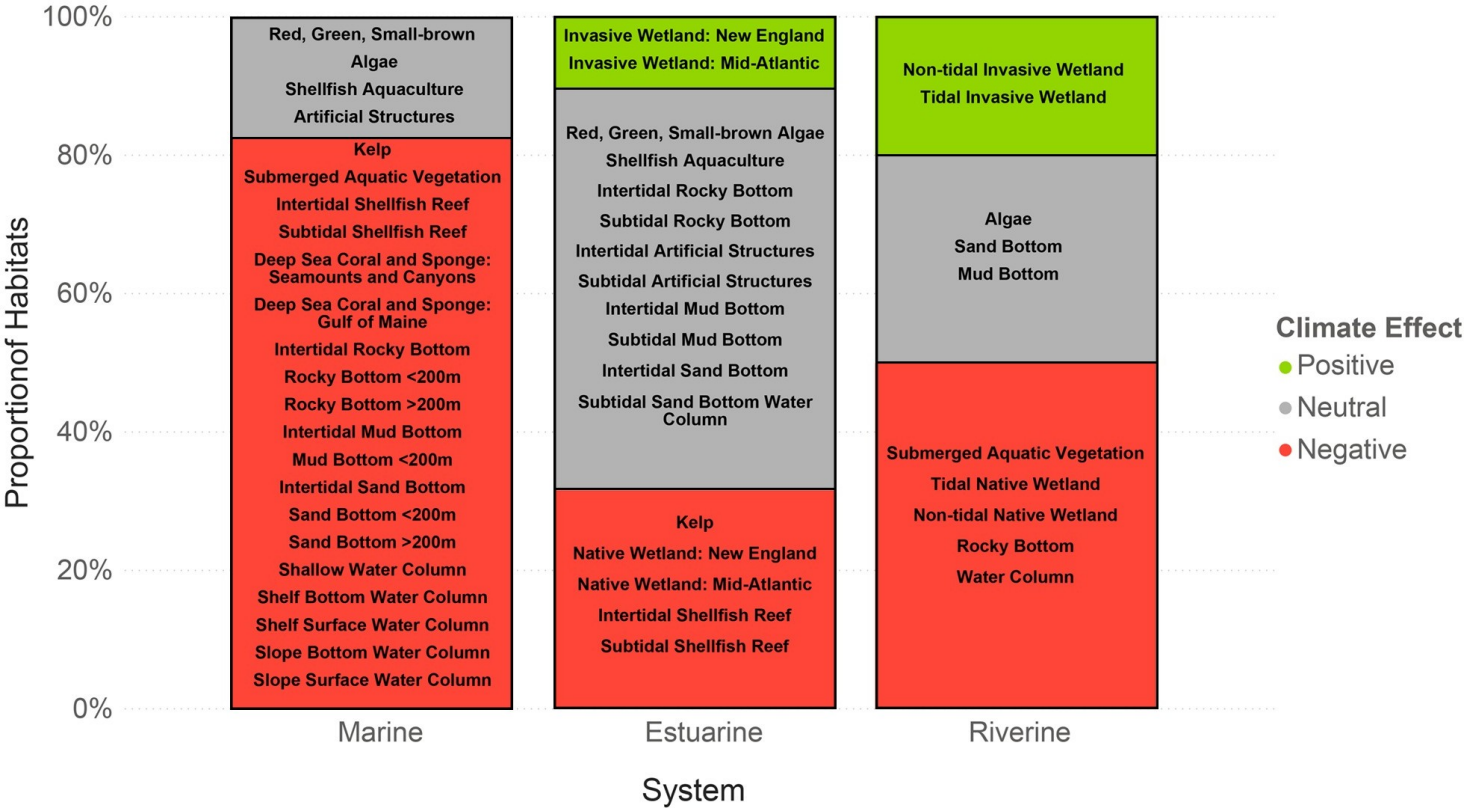
Direction of climate effect by system. Proportion of habitats in each system expected to be positively, neutrally, and negatively affected by climate change.

**Fig 7:**
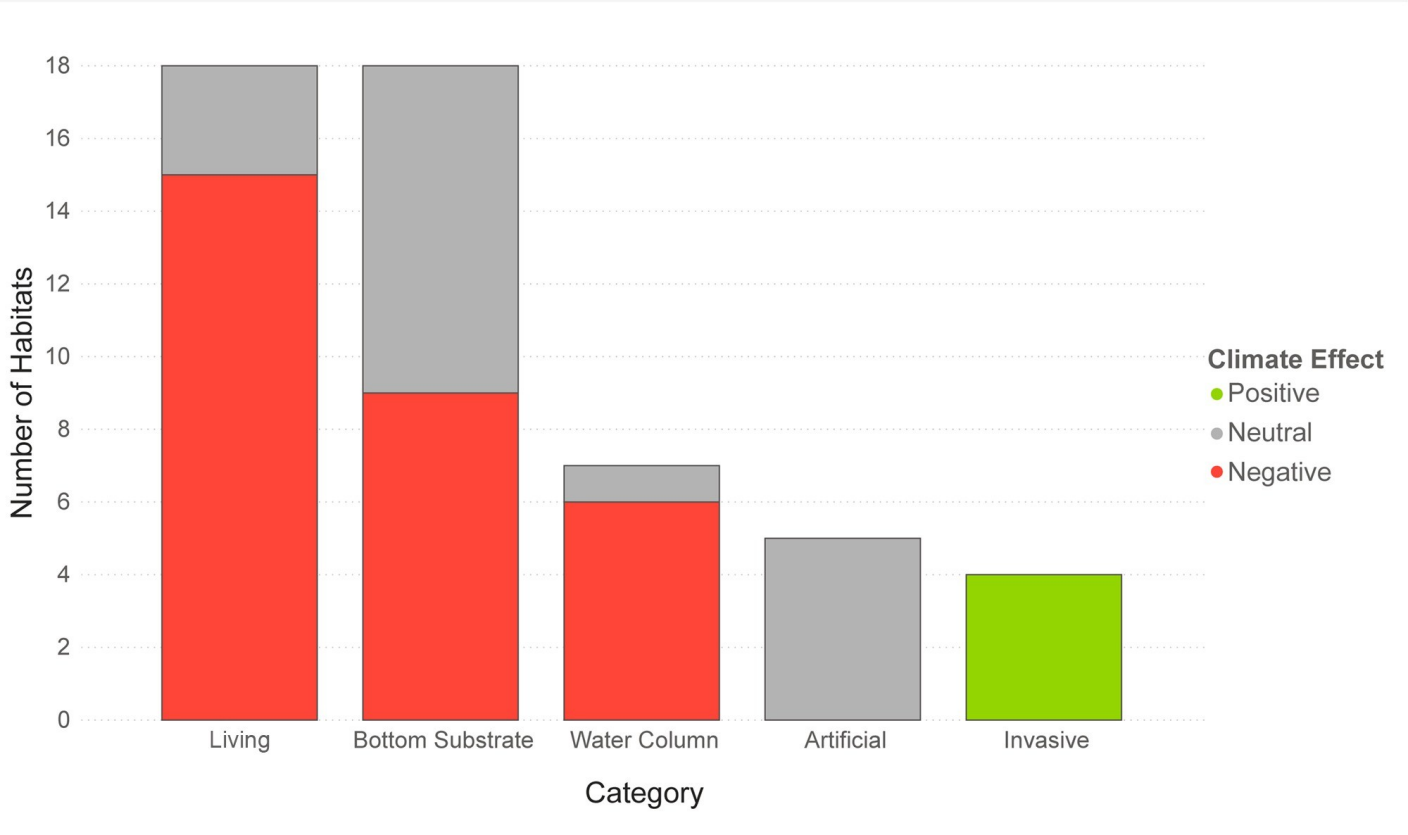
Direction of climate effect by category. Number of habitats in each category expected to be positively, neutrally, and negatively affected by climate change.

Based on the experts’ qualitative assessment of climate vulnerability, 54% (28) of the habitats are expected to be negatively impacted by climate change, 38% (20) are expected to be neutrally impacted by climate change, and only 8% (4) of the habitats are expected to be positively affected by climate change (S6 File). Most of the marine system habitats were classified as being negatively impacted by climate change, whereas potential impacts to estuarine and riverine system habitats were more mixed (Fig 6). When split by category, the majority of living and water column habitats were expected to be negatively impacted by climate change (Fig 7). The four invasive emergent wetland habitats were the only habitats that were expected to be positively affected by climate change.

## Discussion

The results of this habitat climate vulnerability assessment revealed a wide range of climate vulnerabilities for marine, estuarine, and riverine habitats in the Northeast U.S. This assessment found that climate exposure had a greater influence than sensitivity on the overall climate vulnerability for most habitats. The climate models used in our assessment projected that a number of climate factors will deviate substantially from the historical variability and range. This is consistent with other climate studies for the Northeast U.S., including projected changes in sea surface temperature [76, 110, 111], sea levels [77], and ocean pH and aragonite saturation state [112, 113], and as reported in other climate vulnerability assessments for this region [81, 114]. Many of the habitats with high sensitivity to physical and chemical changes associated with climate change, including increasing temperature, ocean acidification, and SLR, ranked the highest in overall climate vulnerability. However, even habitats with moderate sensitivity will be vulnerable to climate change given the magnitude of the projected changes.

Projected change in temperature (surface, bottom, air, and riverine) was an important climate driver for nearly all of the habitats evaluated. While the CMIP5 and ROMS-NWA projections used in this assessment generally show more uniform warming across the U.S. Northeast, regional ocean observations, in the surface and bottom, indicate that the Gulf of Maine and Georges Bank have warmed faster than the Mid-Atlantic Bight [115]. This enhanced warming in New England waters is associated with a northern shift of the Gulf Stream and a weakening of the Labrador Current around the tail end of the Grand Banks [116–118], which is resolved in the high-resolution global climate model CM2.6 [76]. Therefore, marine habitats in portions of the Gulf of Maine, where Gulf Stream associated slope water enters at a greater proportion, and along the shelf break and flanks of Georges Bank may be more vulnerable to climate change than other areas. The CM2.6 model resolves this regional circulation change [76] and is consistent with recent observations indicating a faster and less uniform warming than the ROMS-NWA model, which may have caused the temperature scores for offshore habitats to be biased low in our assessment.

Ocean acidification was also a major factor in the climate exposure for marine and estuarine habitats based on the large projected decrease in pH (20–60 standard deviations from the historical mean). However, the impacts of higher concentrations of CO_2_ and acidification for some habitats are generally not well known, and could potentially be advantageous for habitats such as fleshy alga, submerged aquatic vegetation, and emergent wetlands [62, 113, 119–121]. SLR was also an important driver of vulnerability for all intertidal habitats in the marine and estuarine systems. The exposure scores for Droughts and Floods were less important for riverine habitats than temperature overall, which may be a result of the variable patterns of projected change in high and low flow events over the study area [106], whereas the trend for temperature is unidirectional.

### Patterns in climate vulnerability across habitats

The majority of the habitats that were identified as highly or very highly vulnerable to climate change are living and occur in the estuarine and marine coastal environment, including emergent wetlands, shellfish reefs, subtidal kelp, and submerged aquatic vegetation, and several intertidal habitats in both the estuarine and marine systems. At least 50% of all commercially valuable fish and shellfish in the U.S. depend upon estuaries and nearby coastal waters during one or more life history stages [122], although other reports estimate the dependency as high as 85% [123]. In particular, estuaries provide critical nursery and settlement habitat for many species [122, 124, 125]. Destruction or even a reduction in the condition of important estuarine and coastal habitats can ripple through the food web and lead to decreased abundance of commercially important fish and shellfish species [126–130].

Coastal wetlands, submerged aquatic vegetation, and shellfish reefs minimize coastal flooding, erosion, and runoff, and provide protection to the coastal environment from storm surge and higher sea levels [131, 132]. In addition, because carbon sequestered in the soils of coastal wetlands can be stored for centuries to thousands of years, the loss of coastal wetlands will have significant implications for mitigating greenhouse gas emissions [133, 134]. Stored carbon often is released to the atmosphere when wetlands are destroyed or converted to a different habitat type [135–137], or through increased decomposition due to higher temperature [27, 30]. Although living habitats may have some capacity to adapt to climate change through long-term genetic change, or through short-term acclimatization and phenotypic plasticity, the rate of climate change could exceed the adaptive capacity of many aquatic habitats [32, 138, 139].

Climate change impacts coastal habitats in a multitude of ways. Estuarine and shallow coastal marine water temperatures are influenced by atmospheric warming and solar radiation to a greater degree than oceanic waters. Habitats in the intertidal and shallow subtidal zone are also vulnerable to increased inundation by higher sea levels, erosion, and more frequent and intense coastal storms, leading to physical disruption and conversion of the habitat to a different type (e.g., vegetated to unvegetated, intertidal to subtidal) [25, 52, 140, 141]. In addition, because coastal waters are subject to more sources of low-salinity, acidic waters from rivers and streams, and generally are less buffered than oceanic waters, they are potentially more susceptible to acidification than oceanic waters [31, 113]. More frequent and intense precipitation events can also decrease the salinity and dissolved oxygen conditions in estuarine waters for extended periods of time, such as occurred in the Chesapeake Bay in 2018 and 2019 [142], with implications for fish and shellfish.

Many coastal emergent wetlands in the Northeast U.S. have failed to keep pace with SLR over the past few decades [54, 143–146], leading to erosion and submergence of marsh platforms, and loss of coastal wetland habitat [52, 147]. The mean rate of SLR in the Northeast U.S. over the 20th and 21st centuries has been approximately 2–6 mm per year [148], and could increase to approximately 12–14 mm per year by the end of the century under a 1.0 m global SLR scenario [77]. Some studies in the Northeast U.S. have reported maximum vertical accretion rates for coastal emergent wetlands of around 5–7 mm per year [49, 145, 149, 150], suggesting that many coastal emergent wetlands may become inundated by rising sea levels in the second half of the 21st century. Furthermore, coastal areas hardened by shoreline structures will restrict the capacity of coastal wetlands to migrate inland with increasing SLR [25, 50, 151].

The coastal habitats that were identified as having the highest climate vulnerability are also those most often at risk from degradation due to coastal development. Coastal habitats are threatened by stormwater pollution, eutrophication and general water quality degradation, navigational dredging, shoreline hardening, dredging and filling for coastal development, and the spread of invasive species [37, 38, 42, 152, 153]. Climate-related impacts are likely to exacerbate historic and ongoing degradation of habitats that are already in poor condition from non-climate, anthropogenic impacts [4, 40, 41].

Deep sea corals and sponges found on both seamounts and canyons in the Mid-Atlantic and the Gulf of Maine were the fourth and sixth most vulnerable habitats for all systems, respectively. While deep sea corals and sponges are believed to have lower exposure to many non-climate, anthropogenic effects due to their water depths and distance from shore, these habitats are generally expected to be very sensitive to changes in climate [17, 154, 155], and to anthropogenic activities such as bottom-tending fishing gear. Current observations and historic records suggest that coral habitats were once more extensive in the Gulf of Maine and that current habitat represents refuges that have persisted in the face of intensive bottom fishing, while much of the habitat that was lost has not recovered [66, 96, 156].

Riverine habitats that support diadromous species are experiencing significant climate impacts, including changing hydrology and increasing water temperature. In the past century, stream discharges for rivers with near-natural streamflow in New England and the Mid-Atlantic have generally increased, as have the magnitude and frequency of floods [157–161]. Climate studies that incorporate hydrological models have projected increased variability in streamflow, with greater frequencies of both high-flow and low-flow events predicted for much of the Northeast region [106, 162]. Changes in streamflow magnitude, frequency, and timing can impact riverine habitats and the aquatic species that rely on them [163, 164]. Increases in high flow events can cause stream channel erosion and increased sediment, nutrient, and microbial pathogen delivery to streams, while droughts and decreases in low flow volume can expose aquatic organisms to high temperatures and low dissolved oxygen [105]. Stream temperatures are projected to increase significantly by the end of the century, with the largest increases in the southern Mid-Atlantic and northern New England [107]. These changes make cold- and cool-water rocky-bottom river systems in the Northeast U.S., and the species they support, particularly vulnerable, with implications to food web structure and energy flow in riverine communities [165–169]. In addition, riverine habitats have been historically altered by a host of non-climate perturbations, including damming, water diversion, mineral mining, and storm water pollution [170–173], which can exacerbate climate-related changes in temperature and streamflow.

We considered the vulnerability of several artificial and invasive habitats due to their prevalence in the region and the role they play in providing habitat for some species. The vulnerability ranks for all of these habitats were Moderate and Low. Artificial structures constructed in the subtidal zone include many different materials and purposes, from shoreline protection (e.g., groins, jetties), wrecks, and artificial fishing reefs. Although the materials used in artificial reefs and wrecks are often non-natural (e.g., concrete, steel), they also support biotic communities that can provide ecosystem benefits in some cases. Many of the organisms associated with these structures, particularly mollusks and other shell-forming organisms, are sensitive to changes in temperature and pH. Conversely, artificial structures such as riprap revetments and seawalls constructed for shoreline protection generally support less diverse communities and provide fewer ecological benefits compared to natural shoreline habitats [174–177], and can contain higher occurrences of marine exotic/invasive species compared to native material [178–180].

Four habitats were assessed under the invasive category: two estuarine (New England and Mid-Atlantic) and two riverine (tidal and non-tidal waters) emergent wetland habitat types. These habitats were the only ones in the assessment expected to be positively affected by climate change. The results here are consistent with other studies which suggest invasive species (e.g., *Phragmites)* may be better adapted to anthropogenic stressors and the effects of climate change, and can out-compete native plant community habitats [42, 181, 182].

### Management applications and future research

The climate vulnerability of habitats has important implications for the management and protection of fisheries and protected species. Loss, change, or degradation of habitats will impact the species that depend on them. For example, even when a physical habitat may appear to be unchanged, increasing water temperature can impact the water column surrounding it, which in turn can affect the distribution and abundance of associated species, with potential ecosystem-wide effects [37, 42, 183, 184].

Understanding habitat vulnerability can provide a more complete picture of the vulnerability of species. For example, the FSCVA [81] ranked winter flounder as very highly vulnerable to climate change due to low stock status in the southern part of its range and declining population productivity associated with increased nearshore temperature that has been linked to poor stock recruitment. Habitats important to winter flounder—including submerged aquatic vegetation, kelp, intertidal sand and mud, and tidal wetlands [124, 185–188]—are vulnerable to higher air and water temperature, SLR, and habitat fragmentation. The high climate vulnerability of these habitats, and high dependency of winter flounder on these habitats, suggests a potential critical nexus of climate vulnerability for this species. More broadly, the results from this assessment may help fisheries managers better understand ecosystem drivers of species vulnerability, particularly in cases where fish populations, at least in part, are not meeting fishery objectives due to factors other than fishing mortality [7, 39, 41, 42, 189–191].

The results of this assessment can be used by managers in several additional ways, including updates to designations for Essential Fish Habitat (EFH) and Habitat Areas of Particular Concern under the Magnuson-Stevens Fishery Conservation and Management Act, especially as species distributions shift into new areas. They can also be used to support EFH consultations, Endangered Species Act consultations and critical habitat designations, environmental assessments and environmental impact statements prepared under the National Environmental Policy Act, and updates to Fishery Management Plans. Information about climate vulnerability can also be used to prioritize investments in habitat conservation and restoration. Those involved in developing or implementing state wildlife action plans or place-based management plans like the National Estuary Program’s Comprehensive Conservation Management Plans may find this assessment useful as it will help identify vulnerable habitats and assist in prioritizing efforts to mobilize conservation action and collaboration. Similarly, these results can inform coastal and ocean planning to minimize impacts on highly vulnerable habitats from nearshore and offshore development, and build coastal resilience. As an example, living shorelines that incorporate vegetation alone or in combination with hardened shoreline structures can serve as an alternative approach to traditional “gray” coastal infrastructure for risk reduction, and may provide additional social and ecological benefits [176, 192–194]. Protecting and conserving riverine, estuarine, and marine habitats not only provides productive, functioning habitat for fish, invertebrate, and protected species populations in the short-term, but also increases the climate resiliency of habitats in the long-term.

One of the goals of this assessment was to develop a framework for habitat climate vulnerability that can be applied to other regions. After applying this framework for habitats in the Northeast U.S. region, several elements may be improved upon in future assessments. Classifying the habitats into meaningful categories required balancing the need to keep the assessment to a manageable number of habitats, but avoiding generalized habitat types that would miss important nuances. For example, the marine artificial structures category included several types of structures (e.g., artificial reefs, wrecks, jetties, riprap, and living shorelines), making it difficult to provide a single sensitivity score. We also chose to score bottom substrate, living, and water column habitats separately, rather than assessing “ecological niches” made up of multiple habitat types (e.g., intertidal rocky bottom with attached kelp). In the riverine system, we did not separate habitats into different thermal regimes or sizes. These decisions may have had implications for the vulnerability ranks of some habitats. For instance, it is documented in the literature that cold water, rocky bottom stream habitats are highly vulnerable to climate change [149, 150, 153], so the low vulnerability rank for rocky bottom habitat may have missed important relationships between water column and substrate, and the different ecological roles of cold- and warm-water riverine habitats. Although we conducted supplemental analyses to better understand the vulnerability of riverine “ecological niche” habitats after the scoring process was complete, future assessments may consider defining riverine habitats differently.

Future assessments may consider additional exposure factors, and synergies between them. This climate vulnerability framework could benefit from a greater consideration of exposure to extreme weather events. While extreme precipitation events were considered, this assessment did not account for exposure to marine heatwaves or other events anticipated to occur as a result of climate change. Further, because climate projections were not readily available for water column stratification [110, 195] and hypoxia [196], we did not evaluate these climate exposure factors, but their impacts on habitat quality may warrant inclusion in future habitat CVAs. Lastly, a number of climate exposure factors, such as warming waters, ocean acidification, and deoxygenation, can interact with one another and with other stressors to cause complex and often unanticipated synergistic climate impacts on habitats in the Northeast U.S. [4, 30, 31, 33]. Assessing potential synergistic and additive climate impacts may be useful for future CVAs.

This assessment applied a broad approach to examining the vulnerability of marine, estuarine, and riverine habitats across the Northeast U.S. to climate change. Yet, climate change often impacts habitats at much smaller scales, and exposure and sensitivity may also vary between watersheds, estuaries, or basins. Thus, the climate vulnerability ranking of some habitats may have been higher or lower had we conducted this assessment on a smaller geographic scale. One of the challenges in a smaller-scale analysis is the resolution of climate models, underscoring the importance of downscaled climate models with better resolution in the nearshore and coastal environments. Finally, this assessment complements prior work to understand the vulnerability of fish and invertebrate species [81] and fishing communities in the Northeast U.S. [114] because climate vulnerabilities of these components of the system are closely linked. For instance, the vulnerability of a habitat influences the vulnerability of species, which in turn influences the vulnerability of communities that rely on those habitats and species. Future assessments should therefore examine the vulnerability of each of these components as a connected social-ecological system rather than as individual, independent parts.

## Supporting information

S1 File Habitat classification and definitions. Descriptions and definitions of each habitat in the assessment.
(PDF)

S2 File Sensitivity attribute definitions. Background, definitions, and scoring bins for each sensitivity attribute.
(PDF)

S3 File Exposure factor descriptions. Descriptions and scoring bins for the precipitation, sea level rise, streamflow (droughts and floods), and stream temperature exposure factors.
(PDF)

S4 File Habitat narratives. Detailed scoring results for and descriptions of the climate impacts on each habitat including the key drivers of vulnerability, data quality and gaps, and background information on the habitat.
(PDF)

S5 File Bootstrap and categorical vulnerability natrix. Table showing the bootstrap and categorical vulnerability ranks of each habitat.
(XLSX)

S6 File Direction of climate effect. Table showing the direction of climate effect scores for each habitat.
(XLSX)
