# Supplementary material for: An assessment of marine, estuarine, and riverine habitat vulnerability to climate change in the Northeast U.S
Source: PLoS One. 2021 Dec 9;16(12):e0260654. doi: 10.1371/journal.pone.0260654 (PMC8659346; doi:10.1371/journal.pone.0260654)
Supplement: S1 File — Descriptions and definitions of each habitat in the assessment. (PDF) [file pone.0260654.s001.pdf]

S1. Habitat Classification and Definitions

Habitat Class	Habitat Sub-Classes Included in Class	Definition
**Marine System: Open ocean overlying continental shelf and slope, and nearshore areas with salinities > 30 ppt. The nearshore marine subtidal subsystem includes areas from the shoreline to locations where the depth reaches 200 meters, while the offshore marine subtidal system includes locations where the water is deeper than 200 meters. Intertidal sub-classes encompass the mean high to mean low water lines, and include both the benthic habitat and the water from diurnal tidal inundation.**		
Marine Rocky Bottom	● Marine subtidal rocky bottom bedrock, rubble, cobble/gravel (offshore; >200m)<br>● Marine subtidal rocky bottom bedrock, rubble, cobble/gravel (nearshore; <200m)<br>● Marine intertidal rocky bottom bedrock, rubble, cobble/gravel<br>● Artificial structures including artificial fishing reefs and wrecks, riprap, living shorelines, and groins/jetties	Rocky bottom habitat established on surfaces and crevices of relatively immobile rocky surfaces, including loose rocks of various sizes (rubble, cobble/gravel) and exposed bedrock. In addition, this habitat class includes the epibenthic flora and fauna associated with hard bottoms, including calcareous algae (but not non-calcareous algae included in the red, green, and small brown algae sub-class). Includes shallow corals growing on rocky bottom in <150m water depths. Artificial sub-class includes artificial fishing reefs and wrecks, riprap, living shorelines, and groins/jetties.
Marine Unconsolidated Sand Bottom	● Marine subtidal unconsolidated sand bottom (offshore; >200m)<br>● Marine subtidal unconsolidated sand bottom (nearshore; <200m)<br>● Marine intertidal unconsolidated sand bottom	Subtidal offshore, inshore, and intertidal zone sand habitats. The nearshore marine subtidal sub-class includes areas from the mean low water to locations where the depth reaches 200 meters, while the offshore marine subtidal sub-class includes locations where the water is deeper than 200 meters. Intertidal sub-subclass includes the mean high to mean low water lines. This habitat class includes the epifauna and infauna associated with unconsolidated sand bottom, such as non-reef-forming mollusks (e.g., soft-shell clams, hard clams, sea scallops, surf clams, ocean quahogs), marine worms, small crustaceans, gastropods, and polychaetes. This class excludes specific habitats identified elsewhere (i.e., non-calcareous algal bed, rooted vascular beds, and reef-forming mollusks, i.e., blue mussels, eastern oysters).
Marine Unconsolidated Mud Bottom	● Marine subtidal unconsolidated mud bottom (offshore; >200m)<br>● Marine subtidal unconsolidated mud bottom (nearshore; <200m)<br>● Marine intertidal unconsolidated mud bottom	Subtidal offshore, nearshore, and intertidal zone mud habitats. The nearshore marine subtidal sub-class includes areas from the mean low water to locations where the depth reaches 200 meters, while the offshore marine subtidal sub-class includes locations where the water is deeper than 200 meters. Intertidal sub-class includes the mean high to mean low water lines. This habitat class includes the epifauna and infauna associated with unconsolidated mud bottom, such as non-reef-forming mollusks (e.g., soft-shell clams, hard clams, sea scallops, surf

		clams, ocean quahogs), marine worms, small crustaceans, gastropods, and polychaetes. This class excludes specific habitats identified elsewhere (i.e., non-calcareous algal bed, rooted vascular beds, and reef-forming mollusks, i.e., blue mussels, eastern oysters).
Marine Reef (Offshore)	● Marine subtidal reef, coral-dominated hardbottom, Gulf of Maine (offshore)<br>● Marine subtidal reef, coral-dominated hardbottom, canyons and seamounts (offshore)	Hard-bottom coral and sponge habitats in offshore areas >150 m, including coral gardens, sponge gardens, coral thickets, etc. dominated by hard corals, soft corals, black corals, glass sponges, and demosponges. Shallow water corals (<150 m) are included in the marine nearshore rocky bottom sub-class.
Marine Reef (Mollusk)	● Marine subtidal reef, mollusk (oyster/mussel) (nearshore; <200m)<br>● Marine intertidal reef, mollusk (oyster/mussel)<br>● Cultured mollusks (aquaculture) in subtidal and intertidal zone	Bivalve reefs in the subtidal and intertidal zones in the marine system. Habitat may be on both hard and soft substrates. Specifically focused on reef-building shellfish (e.g. mussels, oyster) that create a biotic hard substrate on the bottom. Note: natural forming, non-reef-building shellfish (e.g., scallop, soft-shell clam, surf clam) are included in unconsolidated sand and mud bottom sub-classes (except for some cultured shellfish, such as hard clam and scallop aquaculture). The intertidal sub-class includes both the reef and the water from diurnal tidal inundation.
Marine Aquatic Bed	● Marine nearshore subtidal and intertidal kelp algal habitats<br>● Marine nearshore subtidal and intertidal red, green, and small brown algal habitats<br>● Marine nearshore subtidal and intertidal rooted vascular bed habitats	Algal and rooted vascular (seagrass) species occurring throughout the study area. Both groups photosynthesize, so are generally limited to the photo zone of the water column. This class also includes aquaculture for macroalgae (e.g., kelp farms in New England). Seagrasses in the marine system of the study area include species occurring only in full salinity waters (>30 ppt). Algal species include, non-rooted, benthic macrophytes separated by kelp species and non-kelp (i.e., red, green, and small brown) algal species occurring in the marine system. Both groups generally occur in both the subtidal and intertidal zones, although are mostly limited to the lower and middle elevations of the intertidal zone due to sensitivity from desiccation.
Marine Water Column	● Marine subtidal water column, shallow inner shelf (well-mixed)<br>● Marine subtidal water column, shelf surface (stratified)<br>● Marine subtidal water column, shelf bottom (stratified)<br>● Marine subtidal water column, slope surface<br>● Marine subtidal water column, slope bottom	The water column is a concept used in oceanography to describe the physical (e.g., temperature, salinity, light penetration) and chemical (e.g., pH, dissolved oxygen, nutrients, salts) characteristics of seawater at different depths. Water column habitats create the foundation for marine food webs, home to primary producers such as phytoplankton and microbes. These habitats are highly dynamic and exhibit swift responses to environmental variables. The marine water column encompasses the open ocean overlying continental shelf and slope, and the associated high-energy nearshore areas with salinities >30 ppt. The shallow inner shelf waters (<20 m water depth) are vertically mixed year round. The shelf

		surface waters (<200 m in depth) are stratified, salinities >30 ppt and <34 ppt., and above the seasonal thermocline, while the shelf bottom waters (<200m in depth) are stratified, salinities >30 ppt and <34 ppt., and below the seasonal thermocline. The slope surface waters (0 to 200 m) with salinities >34 ppt. are on the outer continental shelf and slope (>200 m in depth), while the slope bottom waters (200-1000 m in depth) with salinities >34 ppt., and are the intermediate and bottom layers of those slope waters.
**Estuarine System: Semi-enclosed bodies with salinities ≤30.0 to >0.5 ppt, brackish water. Includes subtidal and intertidal zones, where the intertidal sub-classes include both the benthic habitat and the water from diurnal tidal inundation.**		
Estuarine Rocky Bottom	● Estuarine subtidal natural rocky bottom bedrock, rubble, cobble/gravel<br>● Estuarine intertidal natural rocky bottom bedrock, rubble, cobble/gravel<br>● Estuarine subtidal artificial structures, including artificial reefs and wrecks, riprap, groins, and breakwaters<br>● Estuarine intertidal artificial structures, including artificial reefs and wrecks, riprap, groins, and breakwaters	Includes estuarine bedrock, rubble, cobble/gravel habitats. This class also includes artificial reefs and wrecks, riprap, groins, and breakwaters in the subtidal, estuarine zone. Includes separate sub-classes for natural and artificial structures for both subtidal and intertidal zones in the estuarine system. This habitat class includes the epibenthic flora and fauna associated with these hard bottoms, but exclude the specific habitats identified elsewhere (i.e., non-calcareous algal and rooted vascular beds, coral-dominated hard bottom, mollusk reef). Calcareous algae is included in this class. The artificial subclass includes riprap, artificial reefs and wrecks, and groin/jetties in the subtidal and intertidal, estuarine zones.
Estuarine Unconsolidated Bottom	● Estuarine subtidal unconsolidated sand bottom<br>● Estuarine intertidal unconsolidated sand bottom/shore<br>● Estuarine subtidal unconsolidated mud bottom<br>● Estuarine intertidal unconsolidated mud bottom/shore	Includes intertidal and subtidal sub-classes for both mud and sand habitats, as well as the overtopping water column for intertidal sub-classes. This habitat class includes the epifauna and infauna associated with unconsolidated bottoms, such as non-reef-forming mollusks (e.g., soft-shell clams, hard clams, sea scallops, surf clams, ocean quahogs), marine worms, small crustaceans, gastropods, and polychaetes. This subclass excludes specific habitats identified elsewhere (i.e., non-calcareous algal bed, rooted vascular beds, and reef-forming mollusks such as blue mussels, eastern oysters).
Estuarine Aquatic Bed	● Estuarine subtidal and intertidal kelp algae<br>● Estuarine subtidal and intertidal red, green, and small brown algae<br>● Estuarine subtidal and intertidal rooted vascular beds	Algal and rooted vascular (seagrass) species occurring throughout the study area. Both groups photosynthesize, so are generally limited to the photo zone of the water column. This class also includes aquaculture for macroalgae (e.g., kelp farms in New England). Rooted vascular plants (seagrasses) occurring in the estuarine system of the study area include species occurring in brackish waters (≤30 ppt to >0.5 ppt). Algal species include non-rooted, benthic macrophytes separated into kelp and non-kelp (i.e., red, green, and small brown algal species) sub-classes occurring in the salinity range of the estuarine system. Both groups

		generally occur in both the subtidal and intertidal zones, although are mostly limited to the lower and middle elevations of the intertidal zone due to sensitivity from desiccation.
Estuarine Reef	• Estuarine subtidal mollusk reef habitats (oyster/mussel)<br>• Estuarine intertidal mollusk reef habitats (oyster/mussel)<br>• Estuarine mollusk aquaculture in subtidal and intertidal zone	Includes mollusk reefs in the subtidal and intertidal zones in the estuarine system. Reefs may form on hard or soft substrates. These sub-classes focus on reef-building shellfish (e.g. mussels, oyster) that create a biotic hard substrate at the sediments. This class also includes mollusk aquaculture, which may include both reef and non-reef forming species. Note: natural (non-aquaculture) non-reef-building shellfish (e.g., scallop, soft-shell clam, surf clam) are included in unconsolidated sand and mud bottom subclasses. The intertidal subclass includes both the reef and the water from diurnal tidal inundation.
Estuarine Emergent Wetland	• Estuarine Mid-Atlantic native intertidal, emergent wetlands, native persistent & non-persistent<br>• Estuarine Mid-Atlantic invasive, intertidal emergent wetlands<br>• Estuarine New England native, intertidal emergent wetlands, native persistent & non-persistent<br>• Estuarine New England invasive intertidal emergent wetlands	Wetlands dominated by intertidal perennial plants (characterized by erect, rooted, herbaceous hydrophytes), in a estuarine system where salinity is ≤30 ppt to >0.5 ppt. Includes brackish to full salinity emergent wetlands, persistent and non-persistent. Includes separate sub-classes for native and invasive species for both Mid-Atlantic and New England sub-regions.
Estuarine Water Column	• Estuarine subtidal water column (well-mixed)	The estuarine water column encompasses the stratum from the surface (mean low water) to a maximum depth of 200 m (although few if any estuaries approach this depth). All estuaries in the study area are considered well-mixed.
**Riverine System: Terminates at the downstream end where the concentration of ocean-derived salts in the water ≥0.5 ppt. during the period of annual average low flow, or where the channel enters a lake.**		
Riverine Rocky Streambed and Bank	• Riverine rocky streambed bedrock, rubble, cobble/gravel, tidal and non-tidal	Bedrock, rubble, cobble/gravel streambed and banks for tidal and non-tidal areas of rivers. This includes the epibenthic flora and fauna associated with hard bottoms, but excludes specific habitats (e.g., algal beds, rooted vascular beds, emergent wetlands) that are included in other classes. Riverine rocky shores support sparse plant and animal communities, including lichens and blue-green algae. Also includes large woody debris, boulders, tree roots, and other structural elements that characterize rocky streambed/bank.
Riverine Unconsolidated Streambed and Bank	• Riverine sand streambed and bank, tidal and non-tidal<br>• Riverine mud streambed and bank, tidal and non-tidal	Sand and mud streambeds and banks of tidal and non-tidal rivers, including large woody debris, tree roots, and other structural elements that occur in unconsolidated streambed/bank. Characterized by substrates lacking vegetation except for pioneering plants during brief favorable periods. This includes the

		epifauna/infauna and epiflora associated with these hard bottoms (e.g., freshwater mussels) but exclude specific habitats (e.g., algal beds, rooted vascular beds, emergent wetlands) that are included in other classes.
Riverine Aquatic Bed	• Riverine algal bed, tidal and non-tidal<br>• Riverine rooted vascular bed, tidal and non-tidal	Riverine aquatic beds where the salinity is <0.5 ppt. during the period of annual average low flow. Terminates where the river or stream channel enters a lake. Algal beds occur in both tidal and non-tidal portions of a river. Algal bed species include filamentous green algae occurring in tidal portions of rivers (e.g., *Spirogyra* sp. and *Cladophora* sp.). Non-tidal, freshwater green algae species include muskgrass (*Chara* sp.) and brittle grass (*Nitella* sp.). Rooted vascular beds occur in the lower river within the influence of tidal action and include widgeon grass (*Ruppia maritima*)- a freshwater plant that is tolerant of both fresh and saltwater and wild celery (*Vallisneria americana*). In addition, the pondweed community, including sago pondweed (*Stuckenia pectinata*) and redhead grass (*Potamogeton perfoliatus*) are freshwater submerged plants that have some tolerance to salinities up to about 10 ppt. Hydrilla (*Hydrilla verticillata*) is an invasive freshwater plant that tolerates some salinity (up to 7 ppt). In freshwater, non-tidal portions of rivers, rooted vascular beds in the study area include water stargrass (*Heteranthera dubia*), widgeon grass, wild celery, Eurasian watermilfoil (*Myriophyllum spicatum*), and hydrilla.
Riverine Emergent Wetland	• Riverine tidal native emergent wetlands, persistent and non-persistent<br>• Riverine non-tidal native emergent wetlands, persistent and non-persistent<br>• Riverine tidal invasive emergent wetlands<br>• Riverine non-tidal invasive emergent wetlands	Wetlands dominated by perennial plants (characterized by erect, rooted, herbaceous hydrophytes) in a riverine system where salinity is less than or equal to 0.5 ppt. Includes both tidal and non-tidal wetlands, and both native (persistent and non-persistent) and invasive species. Native tidal species include *Spartina* spp., and native non-tidal species include *Typha* spp. Invasive tidal species include common reed (*Phragmites australis*), and invasive non-tidal species include common reed and purple loosestrife (*Lythrum salicaria*).
Riverine Water Column	• Riverine water column, tidal and non-tidal	The 3-dimensional space of water for both tidal and non-tidal zones in the river. The class includes the physical, chemical, and biological components of the water, but not the river bottom/banks, algal beds, rooted vascular beds, or emergent and riparian vegetation. Terminates at the downstream end where the concentration of ocean-derived salts in the water ≥0.5 ppt. during the period of annual average low flow, and where the channel enters a lake.
