# Supplementary material for: An assessment of marine, estuarine, and riverine habitat vulnerability to climate change in the Northeast U.S
Source: PLoS One. 2021 Dec 9;16(12):e0260654. doi: 10.1371/journal.pone.0260654 (PMC8659346; doi:10.1371/journal.pone.0260654)
Supplement: S2 File — Background, definitions, and scoring bins for each sensitivity attribute. (PDF) [file pone.0260654.s002.pdf]

S2. Sensitivity Attribute Definitions

Background

The goal of this project is to provide regional fisheries, habitat, and protected species managers and scientists with a practical tool to efficiently assess the vulnerability of habitats to climate change. Vulnerability is defined here as the extent to which a habitat could be impacted by climate change. The potential for a change in distribution and positive or negative effects of a changing climate are also assessed. This project considers the overall vulnerability of habitat to climate change to be a function of two main components: exposure and sensitivity/adaptive capacity.

Exposure is a measure of the predicted environmental change that a habitat may experience within the study area. It is the overlap between the current distribution of habitat and the magnitude and spatial distribution of the expected environmental change. The factors accounted for in exposure may include increases in temperature, changes to freshwater input, rise in sea level, ocean acidification and changes to ocean circulation. The exposure factors may vary from one assessment to another to capture the relevant environmental factors specific to the specific study area (e.g., sea ice coverage in the Arctic, coral bleaching in the tropics).

The sensitivity/adaptive capacity component is composed of habitat attributes that are believed to be indicative of the response of a habitat to potential changes in climate. Here attributes that describe the sensitivity and adaptive capacity of a habitat are combined, because of the difficulty in clearly separating these two components of vulnerability. This document provides definitions, justifications, and relationships with climate change, as well as scoring bins for each of the sensitivity/adaptive capacity attributes.

This vulnerability assessment can be completed at a variety of levels of detail with regards to habitats. The hierarchical Cowardin habitat scheme (Cowardin et al. 1979) is used as a structure and specific users can assess habitats of interest at different levels of specificity. The Cowardin scheme covers freshwater, estuarine, and marine habitats which matches well with the habitats used by managed fish and shellfish species and protected species.

This is the first version of this methodology. It is viewed that this assessment would be repeated at some frequency (5-10 years) and new information would be incorporated in each iteration.

This methodology leans heavily on expert opinions. Experts should use their knowledge to interpret these attributes and attribute bins. For example, experts may encounter a situation where the scoring bins suggest a specific attribute score, but their expert knowledge of the habitat leads them to think the score should be higher, lower, or more uncertain. We are counting on the experts to make these decisions.

Definition of Habitat

Habitat is defined as coastal rivers and watersheds, estuaries, and marine waters, from the bottom through the water column. This definition includes an area’s physical, geological, chemical, and biological components that support the survival, growth, and reproduction of plants and animals (NAO 216-17: NOAA National Habitat Policy).

HCVA Sensitivity/Adaptive Capacity Attributes

Habitat Condition

Goal: To determine if a habitat's current status or condition is limiting the ability of that habitat to respond to climate induced changes.

Definition: The ability of the habitat to support a natural, fully-functional ecological community of organisms and the associated/expected ecosystem services.

Background and relationship to climate change: Healthy, intact habitats are expected to be less vulnerable to climate change than degraded habitats. Habitats that have been impacted by either natural or anthropogenic stressors and are in poor condition and have impaired functions, are generally not able to support productive and resilient organisms and communities. Habitat condition and quality can be reduced by a variety of anthropogenic factors, including sedimentation, nutrients, toxic chemicals, physical disturbance, and colonization by exotic and invasive species of plants and animals. In addition, climate change can affect the condition of habitats through various means including warming water, ocean acidification, and sea level rise. The degradation of habitat condition and quality affects a range of ecological processes, including primary and secondary production, trophic dynamics, succession, and species diversity (Deegan and Buchsbaum 2005; Robinson and Pederson 2005). Habitats in poor condition are vulnerable to novel or increased existing natural and anthropogenic stressors, resulting in synergistic and cumulative effects that reduces a habitat's resiliency and adaptation to climate change (Brander 2008; Jackson 2010; Staudt et al. 2012; Staudt et al. 2013; Crozier et al. 2019).

There are several large-scale habitat condition summaries that have been produced. These may be used to develop habitat profiles, along with more regionally specific studies into habitat condition.
EPA Coastal Condition Report
NMFS-FWS Status and Trends
Habitat Condition Assessments for Watershed Health

How to use expert opinion: Based on the background material provided with the assessment and the expert's knowledge, a determination needs to be made as to the condition of the habitat within the study area. It is likely that there is variability in habitat condition across most study areas and these variability should be considered by experts in their scoring.

Bins:

- Low Sensitivity: High quality habitat (near pristine over much of the region), and stable to positive trends in habitat condition across most of the range.
- Moderate Sensitivity: Moderate quality (generally intact but somewhat degraded), or stable trends in habitat condition over much of the range.
- High Sensitivity: Moderate quality over much of the range or poor quality over some of the range (generally intact but somewhat degraded), and decreasing trends in habitat condition over much of the range.
- Very High Sensitivity: Poor condition of habitat across much of the range (significantly degraded), and decreasing trends in habitat condition over the range.

Habitat Fragmentation

Goal: To determine the change in fragmentation of the habitat within the study area. Habitats can be fragmented by natural causes as well as by a variety of human activities. For our purposes, we are primarily interested in anthropogenic changes that generally occur over shorter time frames compared to natural fragmentation, and cause the habitat to reside outside its expected range of natural variability.

Definition: "A landscape-level process in which a specific habitat is progressively sub-divided into smaller, geometrically altered, and more isolated fragments as a result of both natural and human activities, and this process involves changes in landscape composition, structure, and function at many scales and occurs on a backdrop of a natural patch mosaic created by changing landforms and natural disturbances" (McGarigal and McComb 1999) (see http://www.umass.edu/landeco/pubs/mcgarigal.mccomb.1995.pdf and http://www.umass.edu/landeco/teaching/landscape_ecology/labs/fragprotocol.pdf). An alternative definition of fragmentation is "a process whereby a contiguous patch of habitat is transformed into a number of smaller, convoluted and/or disjunct patches, isolated from each other by a matrix of habitat unlike the original" (Wang et al. 2014).

Background and relationship to climate change: Continuous, well connected habitats are expected to be less vulnerable to climate change than fragmented, poorly connected habitats. Fragmentation of habitats can lead to loss of biodiversity and limit the ecological services that the habitat provides. Fragmented habitats also have increased edge effects, which can result in loss of smaller habitat patches through disturbance and change.

Background:
Wang et al. (2014): https://besjournals.onlinelibrary.wiley.com/doi/abs/10.1111/2041-210X.12198
McGarigal et al. (2005): http://www.umass.edu/landeco/teaching/landscape_ecology/labs/fragprotocol.pdf
Crozier et al. (2019):
https://www.researchgate.net/publication/334657545_Climate_vulnerability_assessment_for_Pacific_salmon_and_steelhead_in_the_California_Current_Large_Marine_Ecosystem

How to use expert opinion: Experts are asked to estimate the degree of anthropogenic fragmentation within the natural/historic ecological niche/range of the habitat (e.g., occurrence w/i MLW - 30 feet as the niche for eelgrass beds). Experts should consider whether fragmentation is detrimental to the habitat's function. If this information is not available at the necessary scale, experts are asked to provide an opinion based on their experience and the background material provided.

Bins:

- Low Sensitivity: Habitat is not fragmented and is mostly in its natural, expected state, continuous with large to moderately-sized patches. Habitat function and/or connectivity is not currently limited, and habitat fragmentation is stable to decreasing.
- Moderate Sensitivity: Habitat is mostly continuous with limited fragmentation beyond its natural, expected state. Habitat function and/or connectivity is partially limited, and habitat fragmentation is generally stable.

High Sensitivity: Habitat is fragmented beyond its natural, expected state. Habitat is partially continuous with numerous small to moderately-sized patches. Habitat function and/or connectivity is currently moderately limited, and habitat fragmentation is increasing.

Very High Sensitivity: Habitat is highly fragmented beyond its natural, expected state. Habitat is limited to numerous small patches. Habitat function and/or connectivity is severely limited, and habitat fragmentation is increasing.

Distribution/Range

Goal: To determine the extent of the geographic range of a habitat within the assessment area.

Definition: The historic geographic extent of a habitat, including the leading (i.e., the expanding or colonizing) edge and trailing (i.e., contracting or declining) edge, if applicable, and the water depths for which the habitat naturally occurs.

Background and relationship to climate change: Habitats that occur over a wide latitudinal and depth range within the assessment area are thought to be less vulnerable to climate change. Widely distributed habitats are more likely to be able to persist through a localized destructive event. On the other hand, habitats which exist only on small scales have an increased likelihood of being impacted by a single localized destructive event (i.e., storm, major pollution event, scouring). Habitats which can occur across a wide range of depths are also thought to have reduced sensitivity to change because certain depths may offer refugia to mitigate some changes. Finally, as habitats shift poleward with increasing water temperatures, it is important to keep in mind which habitats are expanding into the study area and which habitats are being pushed out of our study area.

How to use expert opinion: Large-scale maps of habitat distributions are generally available and experts should consider the range of the habitat within the assessment area. When accounting for range across the study area, experts should consider only the area where the habitat could naturally occur. For instance, SAV would only be expected to be at certain depths within the photic zone. Experts should also consider if the study area includes the trailing edge of the habitat distribution.

Bins:

Low Sensitivity: Habitat naturally is found across the latitudinal and depth range of the study area; habitat trailing edge is not found within the study area; or the leading edge of the habitat is expanding into the study area.

Moderate Sensitivity: Habitat is found across the latitudinal range of the study area where it occurs naturally but has a limited depth distribution; habitat trailing edge is not found within the study area.

High Sensitivity: Habitat is somewhat limited in latitude and depth, where it is expected to occur naturally, but the habitat trailing edge is not found within the study area.

Very High Sensitivity: Habitat has limited latitude and depth range within the study area and the trailing edge of the species' distribution is found within the study area.

Mobility/Ability to spread or disperse

Goal: To estimate the ability or capability of the habitat to spread to new areas if their current area becomes less suitable.

Definition: The ability or capability of a habitat to disperse, move, or spread to areas beyond its existing location. Biotic (plant) habitats may disperse vegetatively or reproductively (e.g., seeds, propagules). Some habitats may have the intrinsic capacity to disperse (e.g., salt marsh), but their capability may be limited if extrinsic barriers exist (e.g., seawalls).

Background and relationship to climate change: The mobility of a habitat is a function of (1) the availability of suitable areas to inhabit (e.g., corridors for marsh migration) and (2) intrinsic capacity to disperse (e.g., seeds, propagules, sponge or bivalve larvae). The ability or capability to move, spread, or disperse decreases the vulnerability of a habitat to climate change. As conditions change, if a habitat can expand into new suitable areas the habitat will be more likely to persist over the long term. However, natural or man made barriers can impact a habitat's ability to move or spread into otherwise suitable areas. This attribute is primarily focused on biotic habitats, or the biotic component of abiotic habitats (e.g., epifauna or epiflora). The inability of some abiotic habitats such as large boulders to disperse does not necessarily make those habitats more sensitive to climate change, while the high mobility of others (e.g., marine water column) does not make them less sensitive. For biotic habitats, mobility influences the habitat's ability to adapt to gradual change (e.g., to fill a shifting thermal niche). This attribute is related to, but distinct from, habitat fragmentation, as habitats with low mobility may also be more susceptible to fragmentation.

How to use expert opinion: Scorers should consider both the ability of a habitat to move or spread, as well as natural or anthropogenic barriers to that expansion. When scoring an abiotic habitat, consider only the biotic component of that habitat. If there is no biotic component, give it a low sensitivity score. In cases where scorers judge that the abiotic habitat itself is sensitive due to its lack of mobility, scorers should use their expert opinion to score accordingly. For example, hard bottom reef habitat generally includes epifauna and epiflora communities. While the abiotic (rock) component of the habitat may have low mobility, the associated epifauna and epiflora may have a high ability/capability to spread or move. In this example, we may consider this habitat to have a low sensitivity for this attribute.

Bins:

- Low Sensitivity: High ability / capability to spread or move (e.g., water column habitats)
- Moderate Sensitivity: Habitats which, given time, can move or spread into new areas as they become available, assuming the new areas are not blocked by some sort of barrier.
- High Sensitivity: Habitats in which the ability to spread is limited by the availability of suitable areas with proper conditions, or because of the presence of barriers.
- Very High Sensitivity: Habitats with little or no ability to disperse or move; or habitats in which their ability to spread is blocked by anthropogenic barriers.

Resistance

Goal: To determine, on a relative scale, how durable a habitat (including its function and ecosystem services) is to changing conditions or exposure to disturbance.

Definition: The ability of a habitat to tolerate a stressor and persist while retaining its functionality when subjected to a disturbance.

Background and relationship to climate change: In the literature, resilience and resistance are related concepts. Here we define resistance as the magnitude of disturbance that can be withstood without changing the functional processes and

services normally provided by that habitat. Certain marine habitats are inherently more durable than others. Habitats that are solid and durable will be less likely to be impacted by increased physical disturbances that may arise with projected changes in climate (e.g., increased wave energy, storm frequency or intensity).
References:
https://academic.oup.com/bioscience/article/67/3/208/2900174

How to use expert opinion: There is no one measure of resistance and thus experts must use their opinion as guided by the scoring bins. Looking at past disturbances, how durable is the habitat and is the function of the habitat maintained when disturbances occur? Consider disturbances such as hydrodynamic energy (e.g., wave, current, eddy), sea level rise, and coastal storms. Do not consider other abiotic disturbance factors (e.g., temperature, pH, salinity) as those will be scored under "sensitivity to changes in abiotic factors."

Bins:
- Low Sensitivity: Very resistant to natural disturbance. Functionality of habitat retained even in high disturbance; epiflora and epifauna also adapted to high disturbance.
- Moderate Sensitivity: Moderately resistant to natural disturbance. Functionality and services of habitat can be compromised by disturbance; epiflora, epifauna and infauna somewhat adapted to disturbance.
- High Sensitivity: Generally not adapted for resistance to significant natural disturbance. Functionality of habitats impacted by disturbance; epiflora, epifauna and infauna not well adapted to disturbance.
- Very High Sensitivity: Minimally resistant to natural disturbance. Habitat function easily disturbed in a way that directly impacts services to related epiflora, epifauna and infauna.

Resilience

Goal: To determine, on a relative scale, the rate of recovery for a habitat to likely climate-related disturbances.

Definition: Resilience measures the ability of, and the time period for, a habitat to recover from a disturbance. Recover in this sense means the return to approximately the functional equivalency prior to the disturbance (i.e., the time frame to return to the previous state after a disturbance).

Background and relationship to climate change: In the literature, resilience and resistance are related concepts. Resilience measures the ability of a habitat to recover from a disturbance. Recover in this sense means return close to functional equivalency. In some cases, these disruptive events can increase adaptation capacity by the proliferation of remaining resistant individuals. A habitat that has a high rate of recovery after a disturbance is thought to be better able to adapt to changes in climate. This can be accomplished by the habitat bouncing back, or re-establishing itself, after a disruptive event.
References:
http://www.pnas.org/content/114/31/8301
https://www.nefsc.noaa.gov/publications/tm/tm181/

How to use expert opinion: Looking at past disturbances, how long has it taken for the habitat to return to original structure and function. Past disturbances can include extreme natural events or seasonal changes in conditions, including hydrodynamic energy (e.g., wave, current, eddy), sea level rise, and coastal storms. If a habitat is re-established in a new

location, consider whether the change in location alters its function within the geographic context of the study area. Scorers should consider both the magnitude of the possible disturbances and the rate of recovery of the habitat to those various disturbances. Refer to the following conceptual diagram as guidance:

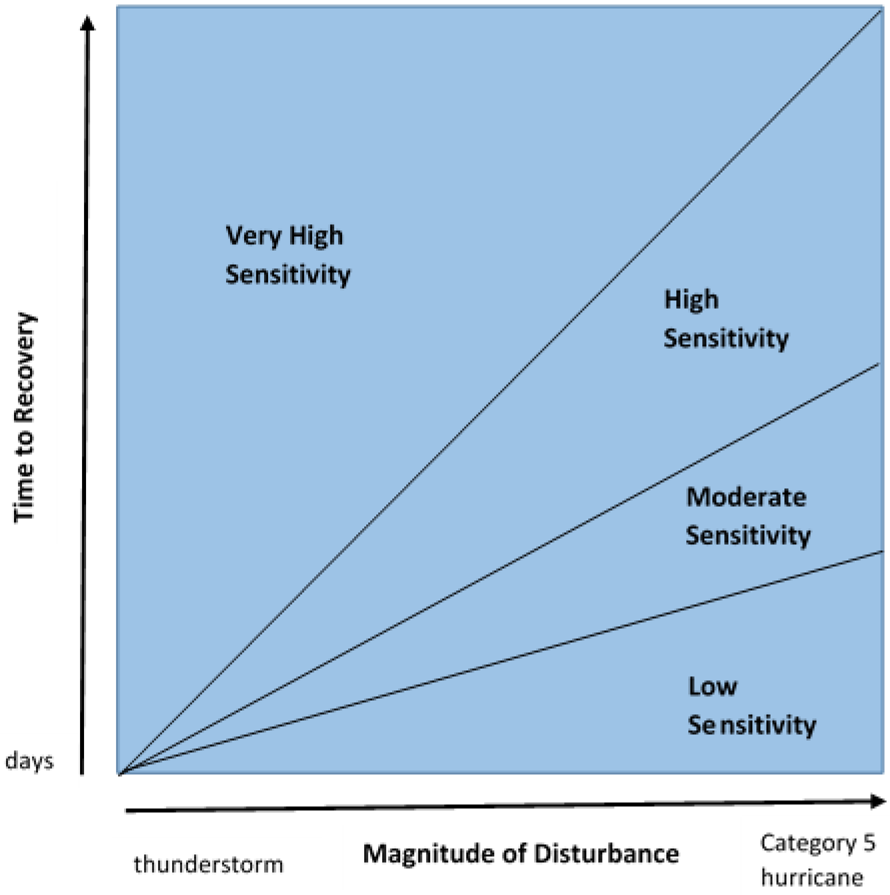

Bins:

Low Sensitivity: Very resilient to high natural disturbance. Habitat function, structure, and services of the habitat is able to return to the previous state in a relatively short period even after high disturbance; epiflora, epifauna, and infauna also able to recover in a short period after high disturbance.

Moderate Sensitivity: Moderately resilient to moderate natural disturbance. Habitat function, structure, and services of the habitat is able to return to the previous state in a relatively short period after moderate disturbance; epiflora, epifauna, and infauna somewhat able to recover in a short period after moderate disturbance.

High Sensitivity: Generally not resilient to moderate to high natural disturbance. Habitat function, structure, and services of the habitat is unable to return to the previous state in a relatively short period after moderate disturbance; epiflora, epifauna, and infauna also unable to recover in a short period after moderate disturbance.

Very High Sensitivity: Minimally resilient to natural disturbance. Habitat function, structure, and services unable to return to the previous state even after minimal disturbance; epiflora, epifauna and infauna also unable to recover after minimal disturbance.

Sensitivity to changes in abiotic factors

Goal: To determine, on a relative scale, how susceptible a habitat is to an acute or persistent change in physical and chemical conditions which are anticipated under climate change (e.g., temperature, pH, salinity).

Definition: Sensitivity to changes in abiotic factors is a measure of a habitat's ability to tolerate changes in chemical and physical characteristics of the environment. For this study, abiotic factors include temperature, salinity, dissolved oxygen, and carbonate chemistry/CO2 concentration, but may include other factors (e.g., nitrogen) and synergistic effects.

Background and relationship to climate change: This attribute seeks to evaluate how sensitive a habitat is to changes in multiple abiotic factors. Slightly sensitive represents a chronic impact with effects being demonstrated on the scale of 1 year or more. Moderately sensitive represents a chronic impact with effects being demonstrated on the scale of less than a year. Highly sensitive represents an acute impact. Habitats that are sensitive to changes in abiotic factors will be more vulnerable to climate change than habitats that are not sensitive to changes in abiotic factors because of the compounding effects of multiple stressors.

How to use expert opinion: Experts should take into account both the number of abiotic factors as well as magnitude (see Table below). Abiotic factors stressors include: temperature, salinity, dissolved oxygen, sea level rise, and carbonate chemistry/CO2 concentration, but may include other factors (e.g., nitrogen) and synergistic effects. Take synergistic impacts into account and treat the synergistic effect as another factor. The expert should then sum across columns to estimate the sensitivity. Avoid double counting the same factors between this attribute and sensitivity and intensity of non-climate stressors.

**Sensitivity**	**Temperature**	**Dissolved O_2**	**Carbonate Chemistry/ CO_2 concentration**	**Salinity**	**Other Abiotic Factors**	**Synergistic Factors**
Not sensitive	0	0	0	0	0	0
Slightly sensitive	1	1	1	1	1	1
Moderately sensitive	2	2	2	2	2	2
Highly sensitive	3	3	3	3	3	3

Bins:

Low Sensitivity: Score < 5
Moderate Sensitivity: Score 5-7
High Sensitivity: Score 8-10
Very High Sensitivity: Score > 10

Sensitivity and intensity of non-climate stressors

Goal: To determine the relative impact of non-climate impacts currently affecting the habitats.

Definition: The sensitivity of a habitat to existing non-climate stressors, as well as a measure of the intensity of each non-climate stressor, can increase its vulnerability to climate change. For this study, non-climate stressors may include dredging/filling, pollution/eutrophication, invasive species, harmful algal blooms, and shoreline hardening, but can also include other stressors (e.g., fishing gear) and synergistic effects.

Background and relationship to climate change: Habitats that are already stressed by human influences are likely to be more vulnerable to change. Impacted habitats are less able to resist climate change impacts and are less resilient to disturbance. Habitats that are sensitive to non-climate stressors may be vulnerable to novel or increased existing natural and anthropogenic stressors, resulting in synergistic and cumulative effects that reduces a habitat's resiliency and adaptation to climate change (Brander 2008; Jackson 2010; Staudt et al. 2012; Staudt et al. 2013). Consider only negative impacts to non-climate stressors when scoring the sensitivity (e.g., if a habitat will be positively impacted by eutrophication, it should not be scored highly sensitive).

This report contains detailed information on a wide-range of anthropogenic impacts to habitats in the Northeast Region: NOAA Tech Memo on Non-fishing habitat impact report

How to use expert opinion: Estimate the sensitivity and intensity of the habitat for all applicable non-climate stressors, from highly sensitive (3), moderately sensitive (2), slightly sensitive (1), or not sensitive (0). The sum of sensitivity scores determines the sensitivity of the habitat. Additional and relevant non-climate stressors not listed in the table should be described and scored in "Other/Synergistic Effects". Experts should take into account both the number of non-climate stressors as well as intensity (adjust score higher for stressors with high intensity, but do not adjust score lower for stressors with low intensity). For example, submerged aquatic vegetation (SAV) is very sensitive to mechanical impacts from dredging, but if the probability or intensity of dredging in a geographic area is low the habitat is not vulnerable to the stressor in that location. However, the score should not be lowered for SAV in that location because the intensity is low. On the other hand, SAV may not be sensitive to harmful algal blooms (HAB) and generally able to tolerate those conditions, but if the intensity of HAB is very high and the blooms substantially impair light transmittance over weeks or months it may increase the vulnerability of SAV in an estuary. In this case, the sensitivity score should be adjusted higher.

Scorers should also consider projected future changes in the magnitude of each stressor in addition to its current magnitude. An example may be that an increasing trend in shoreline hardening may warrant higher scores if a habitat type may be sensitive to wide-spread shoreline hardening. Take synergistic impacts into account and treat the synergistic effect as another stressor. Avoid double counting the same factors between this attribute and sensitivity to changes in abiotic factors.

	Dredging/ filling	Pollution/ Eutrophication	Harmful Algal Bloom	Invasive Species	Shoreline Hardening/ Built Environment	Other / Synergistic Effect
Not sensitive	0	0	0	0	0	0
Slightly sensitive	1	1	1	1	1	1
Moderately sensitive	2	2	2	2	2	2
Highly sensitive	3	3	3	3	3	3

Bins:
- Low Sensitivity: Score < 5
- Moderate Sensitivity: Score 5-7
- High Sensitivity: Score 8-10
- Very High Sensitivity: Score > 10

Dependency on critical ecological linkages

Goal: To determine the relative importance of other species in maintaining the ecological function and health of a habitat. This trait is included because some habitats are not only vulnerable to direct impacts of climate change but also to the climate impacts on key species that are associated with the habitats.

Definition: Some habitats may depend upon associated species to maintain the health or function of a habitat. In some extreme cases, certain habitats cannot exist without the active maintenance or engineering of associated species. Examples of high-dependency habitats include coral reef symbionts (i.e., zooxanthellae), grazers on eelgrass, riparian vegetation or beavers in riverine habitats, urchin grazers on macroalgae, rocky intertidal invertebrates and vegetated communities.

Background and relationship to climate change: Habitats requiring the active participation or presence of associated species to maintain the health or function of a habitat. The more dependencies on ecological linkages, the higher likelihood that one or more of these linkages will be affected by climate change.

How to use expert opinion: Ecosystems, of which habitats are part of, are inherently complicated. All habitats depend on associated species to some extent. Experts should use their judgment to determine the relative important linkages between keystone species and the likelihood that these linkages could be disrupted by changes in climate.

Bins:
- Low Sensitivity: Habitats which can persist without intervention from ecological engineers or keystone species.
- Moderate Sensitivity: Habitats which have limited dependence on outside species in order to maintain their function.
- High Sensitivity: Habitats which depend on key linkages with other species to maintain their condition or function.

Very High Sensitivity: Habitats which are actively maintained by a key species, or suite of species, which may be vulnerable to changes in climate conditions. Without intervention from these species, the habitats will become degraded.
