# Supplementary material for: An assessment of marine, estuarine, and riverine habitat vulnerability to climate change in the Northeast U.S
Source: PLoS One. 2021 Dec 9;16(12):e0260654. doi: 10.1371/journal.pone.0260654 (PMC8659346; doi:10.1371/journal.pone.0260654)
Supplement: S3 File — Descriptions and scoring bins for the precipitation, sea level rise, streamflow (droughts and floods), and stream temperature exposure factors. (PDF) [file pone.0260654.s003.pdf]

S3. Exposure Factor Descriptions

Exposure Factor: Precipitation

Projected Change in Annual Mean Precipitation

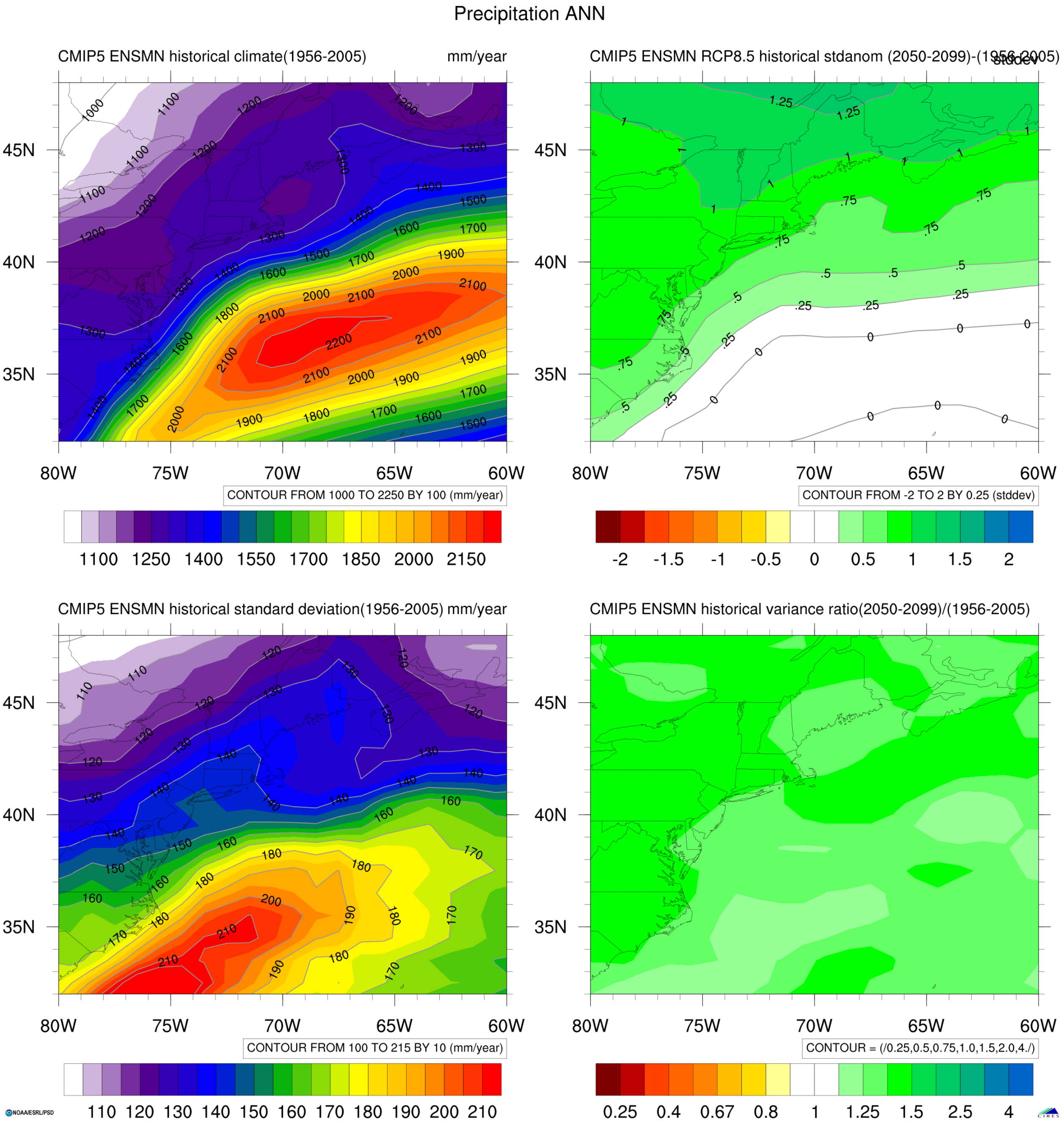

Figure 1. End-of-century (2050-2099) projected precipitation (in millimeters per year) from the Coupled Model Intercomparison Project 5 (CMIP5). Scoring bins are based on the top right panel, which depicts the

standardized historic anomaly (z-score, difference between the projected end-of-century mean for each exposure factor and the variability of the historic mean). Source: ESRL Climate Portal.

Extreme Events

Increased extreme precipitation events can negatively impact habitats equally or greater than changes to long-term mean precipitation. Heavy precipitation events can cause compounding or synergistic effects, such as increased sediment and stormwater runoff, overloading of municipal or agricultural waste systems, and large freshwater input from rivers and streams. These events may also cause physical impacts to habitats, such as scouring and erosion of unconsolidated sediments and epiflora.

Observed:

Annual precipitation has shown a shift towards greater variability and higher totals across the entire United States since 1970, although extreme precipitation events have been higher on the east coast than on the west coast and midwest regions (Karl and Knight 1998; Sun and Lall 2015; Walsh et al. 2014). Between 1958 and 2016, the northeast region saw a 55 percent increase in the amount of precipitation falling as very heavy events (defined as the total precipitation falling in the top 1 percent of all days with precipitation) (Easterling et al. 2017). Furthermore, the number of 2-day events with a precipitation total exceeding the largest 2-day amount that is expected to occur, on average, only once every 5 years increased by 92 percent in the northeast region (Easterling et al. 2017). Douglas and Fairbank (2011) reported strong increases in the magnitude of extreme precipitation events between 1970 and 2008 in New England, and especially in eastern Massachusetts and southern New Hampshire. They also found increases in the frequency of extreme rainfall events in New England, which was highest during August through November, but the increased frequency was not as great as the increase in the magnitude of extreme events.

Projected:

The intensity and frequency of heavy precipitation events are projected to increase (USGCRP 2017). The Fourth National Climate Assessment (NCA 4) found that heavy precipitation events above the 99th percentile of daily values have increased by 38 percent when measured from 1901, and by 55 percent from 1958 when measured with a more robust set of observations in the Northeast U.S. Under the RCP8.5 scenario in the end-of-century time period (2070-2099), the change in total annual precipitation falling in these 99th percentile heavy rainfall events is expected to exceed 40% in the Northeast U.S. (Figure 2). This is linked to observed and projected increases in the frequency of thunderstorms and the associated amount of precipitation (USGCRP 2017). Future droughts in most regions will likely be more intense and last longer, though these trends are expected to be less pronounced in the Northeast compared with other regions (USGCRP 2017).

The NCA 4 reported projected increases in extreme precipitation for the northeast region, indicating the 20–year return period amount for daily precipitation (a daily precipitation quantity that has only a 5 percent chance of being exceeded in any year) will increase by 14 percent and 22 percent under the RCP4.5 and RCP8.5 scenarios, respectively (Easterling et al. 2017). For the mid-Atlantic region, most IPCC AR4 models have been in agreement that precipitation intensity is projected to increase. For example, under the high-emissions

scenario (A1B), precipitation intensity is projected to increase by one standard deviation by 2100 (Meehl et al. 2007).

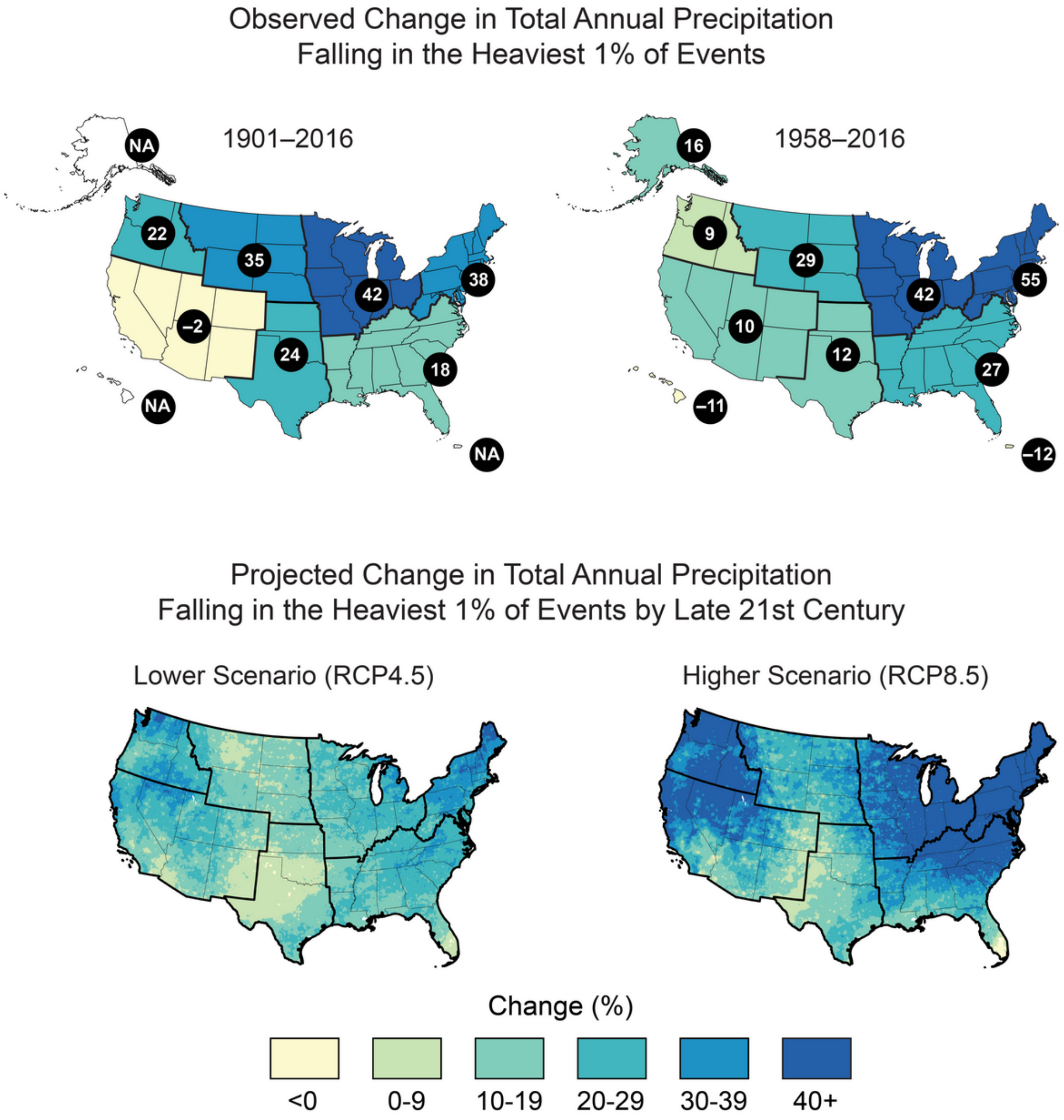

Figure 2: Heavy precipitation is becoming more intense and more frequent across most of the United States, particularly in the Northeast and Midwest, and these trends are expected to continue in the future. This map shows the observed (top; numbers in black circles give the percentage change) and projected (bottom) change in the amount of precipitation falling in the heaviest 1% of events (99th percentile of the distribution). Observed historical trends are quantified in two ways. The observed trend for 1901-2016 (top left) is calculated as the difference between 1901-1960 and 1986-2016. The values for 1958-2016 (top right), a period with a denser station network, are linear trend changes over the period. The trends are averaged over each National Climate Assessment region. Projected future trends are for a lower (RCP4.5, left) and higher

(RCP8.5, right) scenario for the period 2070-2099 relative to 1986-2015. Adapted from Easterling et al. 2017. (Source: USGCRP 2017)

Observed and Projected Tropical and Extratropical Storms:
The IPCC AR5 reported no significant observed trends in frequency of global tropical or extratropical cyclones over the past century (Hartmann et al. 2013). However, historical data indicate that between the 1950s and 2010s, mid-latitude synoptic storm systems (i.e., extratropical cyclones) which include blizzards and nor'easters in the northeast region, increased in intensity and their tracks shifted northward (Vose et al. 2014; Wang et al. 2012). Lehmann et al. (2014) examined the results of CMIP5 RCP8.5 and concluded that up to the year 2100 the northeast region would likely experience an increase in the frequency and intensity of winter extratropical cyclone events, but a slight decrease in summer events. Colle et al. (2013) projected extratropical cyclones may become more intense (10-40 percent) along the northeast coast, especially during the mid-twenty-first century as a result of an increase in latent heat release due to a moister atmosphere.

The northeast region is also affected by tropical cyclone systems that originate in the Atlantic and Caribbean basins. Various researchers have hypothesized that an increase in SST will increase the maximum potential intensity of tropical cyclones and should be reflected by an increase in the frequency of the strongest hurricanes (Knutson et al. 2010; Kossin et al. 2007; Trenberth 2005). In fact, over the satellite era the intensity of the strongest hurricanes in the Atlantic increased, although there is little evidence for this trend in other regions and the globe (Hartmann et al. 2013; Kossin et al. 2007). The intensification of Atlantic tropical cyclones is borne out in the historical record. Between the 1970s and early 2000s, the number of major hurricanes (Category 4 and 5) in an average year approximately doubled (Emanuel 2005; Webster et al. 2005).

How to use expert opinion:
The impacts of changes in precipitation can manifest in two different ways, long term changes to the mean annual precipitation and the change in the timing/variability/intensity in precipitation events. We ask the scorer to incorporate both factors into their precipitation scores. Start with the standard scoring bins below based on the changes in the mean annual precipitation and adjust based on the information above about changes in extreme rainfall events. Scorers should adjust their scores higher if the evidence above shows that high-intensity precipitation events are expected to increase in frequency or magnitude.

Scoring Bins:
Baseline scoring bins based on change in annual mean; adjusted for extreme precipitation events.

- Low: 0-1.5
- Moderate: 1.5-4
- High: 4-5.5
- Very High: 5.5+

References

Colle BA, Zhang Z, Lombardo KA, Chang E, Liu P, Zhang M. 2013. Historical evaluation and future prediction of eastern North American and western Atlantic extratropical cyclones in the CMIP5 models during the cool season. Journal of Climate 26(18):6882-903.

Douglas EM, Fairbank CA. 2011. Is precipitation in northern New England becoming more extreme? Statistical analysis of extreme rainfall in Massachusetts, New Hampshire, and Maine and updated estimates of the 100-year storm. Journal of Hydrologic Engineering 16(3):203-17.

Easterling DR, Kunkel KE, Arnold JR, Knutson T, LeGrande AN, Leung LR, Vose RS, Waliser DE, F. WM. 2017. Precipitation change in the United States. Climate Science Special Report: Fourth National Climate Assessment. [Wuebbles DJ, Fahey DW, Hibbard KA, Dokken DJ, Stewart BC, Maycock TK (eds)]. U.S. Global Change Research Program. p. 207-30.

Emanuel K. 2005. Increasing destructiveness of tropical cyclones over the past 30 years. Nature 436(7051):686-8.

Hartmann DL, Klein Tank AMG, Rusticucci M, Alexander LV, Brönnimann S, Charabi YA-R, Dentener FJ, Dlugokencky EJ, Easterling DR, Kaplan A, Soden BJ, Thorne PW, Wild M, Zhai P. 2013. Observations: atmosphere and surface. Climate change 2013. The physical science basis. Contribution of Working Group I to the Fifth Assessment Report of the Intergovernmental Panel on Climate Change. [Stocker T, Qin D, Plattner G-K, Tignor M, Allen S, Boschung J, Nauels A, Xia Y, Bex V, Midgley P (eds)]. Intergovernmental Panel on Climate Change. p. 159-254.

Karl TR, Knight RW. 1998. Secular Trends of Precipitation Amount, Frequency, and Intensity in the United States. Bulletin of the American Meteorological Society 79(2):231-42.

Knutson TR, McBride JL, Chan J, Emanuel K, Holland G, Landsea C, Held I, Kossin JP, Srivastava AK, Sugi M. 2010. Tropical cyclones and climate change. Nature Geoscience 3(3):157-63.

Kossin JP, Knapp KR, Vimont DJ, Murnane RJ, Harper BA. 2007. A globally consistent reanalysis of hurricane variability and trends. Geophysical Research Letters 34(4):1-6.

Lehmann J, Coumou D, Frieler K, Eliseev AV, Levermann A. 2014. Future changes in extratropical storm tracks and baroclinicity under climate change. Environmental Research Letters 9:8.

Meehl GA, Stocker TF, Collins WD, Friedlingstein P, Gaye AT, Gregory JM, Kitoh A, Knutti R, Murphy JM, Noda A, Raper SCB, Watterson IG, Weaver AJ, Zhao Z-C. 2007. Global climate projections. Climate

change 2007. The physical science basis. Contribution of Working Group I to the Fourth Assessment Report of the Intergovernmental Panel on Climate Change. [Solomon S, Qin D, Manning M, Chen Z, Marquis M, Averyt KB, Tignor M, Miller HL (eds)]. Intergovernmental Panel on Climate Change. p. 748-845.

Sun X, Lall U. 2015. Spatially coherent trends of annual maximum daily precipitation in the United States. Geophysical Research Letters 42(22):9781-9.

Trenberth K. 2005. Uncertainty in hurricanes and global warming. Science 308(5729):1753-4.

USGCRP. 2017: *Climate Science Special Report: Fourth National Climate Assessment, Volume I* [Wuebbles, D.J., D.W. Fahey, K.A. Hibbard, D.J. Dokken, B.C. Stewart, and T.K. Maycock (eds.)]. U.S. Global Change Research Program, Washington, DC, USA, 470 pp, doi: 10.7930/J0J964J6.

Vose RS, Applequist S, Bourassa MA, Pryor SC, Barthelmie RJ, Blanton B, Bromirski PD, Brooks HE, DeGaetano AT, Dole RM, Easterling DR, Jensen RE, Karl TR, Katz RW, Klink K, Kruk MC, Kunkel KE, MacCracken MC, Peterson TC, Shein K, Thomas BR, Walsh JE, Wang XL, Wehner MF, Wuebbles DJ, Young RS. 2014. Monitoring and understanding changes in extremes: extratropical storms, winds, and waves. Bulletin of the American Meteorological Society 95(3):377-86.

Walsh J, Wuebbles D, Hayhoe K, Kossin J, Kunkel K, Stephens G, Thorne P, Vose R, Wehner M, Willis J. 2014. Chapter 2: our changing climate. Climate change impacts in the United States. The Third National Climate Assessment. [Melillo JM, Richmond TC, Yohe GW (eds)]. U.S. Global Change Research Program. p. 19-67.

Wang XL, Feng Y, Compo GP, Swail VR, Zwiers FW, Allan RJ, Sardeshmukh PD. 2012. Trends and low frequency variability of extra-tropical cyclone activity in the ensemble of twentieth century reanalysis. Climate Dynamics 40(11-12):2775-800.

Webster PJ, Holland GJ, Curry JA, Chang H-R. 2005. Changes in tropical cyclone number, duration, and intensity in a warming environment. Science 309(5742):1844-6.

Exposure Factor: Sea Level Rise

Historic and Current Sea Level Rise

Key Points:

- Sea level rise (SLR) is caused by a combination of changes in the density of seawater (warming and freshening), melting of glaciers, ice caps, and ice sheets, and vertical land movements such as subsidence and sedimentation.
- The annual mean rate of SLR for nearly all locations on the northeast coast is higher than the global average and, in general, the rate for the Mid-Atlantic is higher than the New England region.

Background and Global Effects

Two major processes are responsible for altering the volume of water in the global oceans and raising global sea levels. Steric forces involve changes in the density of seawater, and include thermosteric, or thermal expansion, which is the result of increased warming of the oceans, and halosteric, or the freshening of ocean waters. Eustatic forces are contributions from the melting of glaciers, ice caps, ice sheets, and other land water reservoirs, including changes in land hydrology and the atmosphere. In addition, regional sea level can be affected by dynamic changes associated with changing ocean currents and the redistribution of mass in the ocean. A third process, isostatic forces or vertical land movements resulting from postglacial rebound, plate tectonics, subsidence, and sedimentation, can influence the relative sea level of coastal landmasses (Church et al. 2008; Church et al. 2013). Since the early 1970s, the combined glacier mass loss and ocean thermal expansion from warming are attributed to about 75% of the observed global mean SLR (IPCC 2014).

Since the mid-19th century, the rate of global mean SLR has been larger than the mean rate during the previous 2,000 years (IPCC 2014; Kemp et al. 2011). Using statistical meta-analysis of proxy relative sea-level reconstructions and tide-gauge data, Kopp et al. (2016) reported a significant acceleration of global SLR beginning in the 19th century and yielded a 20th century rise that is extremely likely to be greater than during any of the previous 2,800 years. Global mean sea level has increased by about 21 cm to 24 cm since 1880, with about 8 cm occurring since 1993 (Church and White 2011; Hay et al., 2015). The change in global mean sea level from 1993-2018, estimated from satellite radar altimeters and monitored against a network of tide gauges, is 3.1 ± 0.4 mm/year (Figure 1).

Figure 1. Change in mean global sea level (in millimeters) from 1993-2018 (Univ. of Colorado Sea Level Research Group; http://sealevel.colorado.edu/).

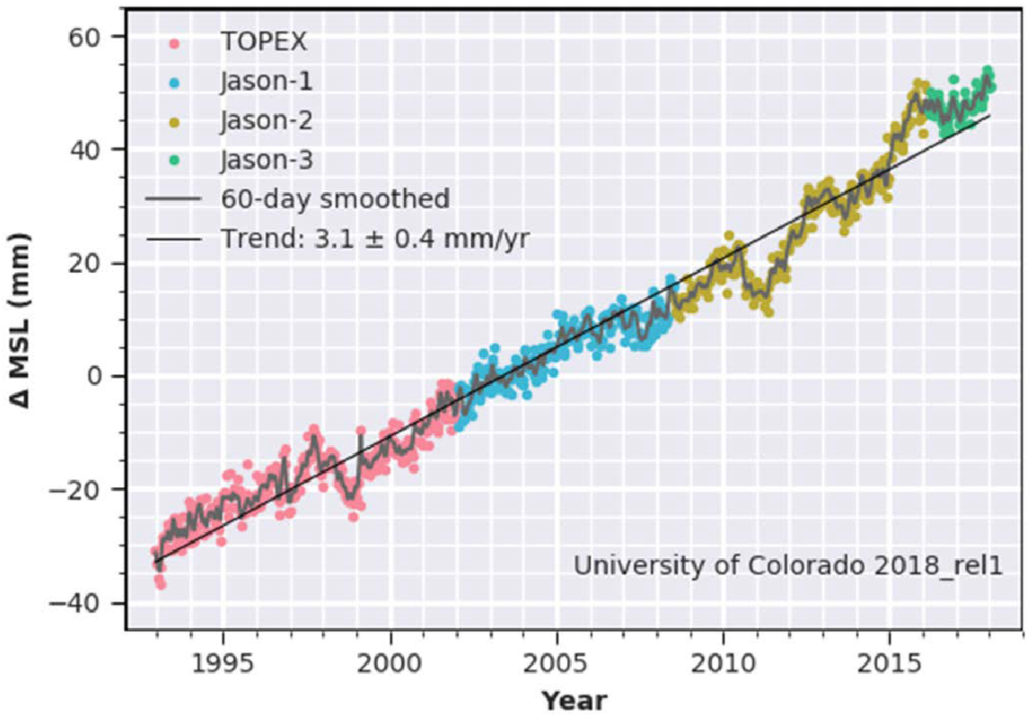

Northeast Sea Level Rise

The rate of SLR is not uniform spatially, and in some regions the rates can be several times higher than the global mean, while in other regions sea level is falling (Bindoff et al. 2007). Yin et al. (2009) found that changes in ocean circulation in the North Atlantic – specifically, a weakening of the Gulf Stream – is playing a role in increasing SLR on the U.S. east coast. Sallenger et al. (2012) reported a recent acceleration in SLR on 1,000 km of the east coast north of Cape Hatteras, which may be attributed to high-latitude, North Atlantic warming and rising surface water buoyancy. These dynamic sea level changes associated with changing ocean currents and the Atlantic Meridional Overturning Circulation are attributed to an accelerating rate in the Mid-Atlantic region of SLR 3–4 times higher than the global average (Levermann et al. 2005; Sallenger et al. 2012; Yin et al. 2009).

The annual mean rate of SLR for nearly all locations on the northeast coast is higher than the global average and, in general, the rate for the Mid-Atlantic is higher than the New England region (Figure 2). The relative SLR in the Chesapeake Bay region between 1927 and 2006 was 3.5–4.4 mm per year, faster than any other location on the Atlantic coast and twice the global average eustatic rate (DeJong et al. 2015; Eggleston and Pope 2013). Post-glacial adjustment has been reported to be the primary cause of the deviation from global SLR trends in the Chesapeake Bay region (DeJong et al. 2015), although compaction due to extensive groundwater pumping has been attributed to more than half of the observed land subsidence in this region (Eggleston and Pope 2013).

Figure 2. Relative sea level trends for 26 northeast U.S. cities over the 20th century to 2017. The red line depicts the global mean rate of SLR from tide gauge data for the 20th century and 21st century; the blue line depicts the global mean rate of SLR from satellite altimetry data from 1993-2018. The beginning of record for tide gauge data varies by station, from 1900 and 1970 (NOAA Tides and Currents; https://tidesandcurrents.noaa.gov/sltrends/sltrends.html).

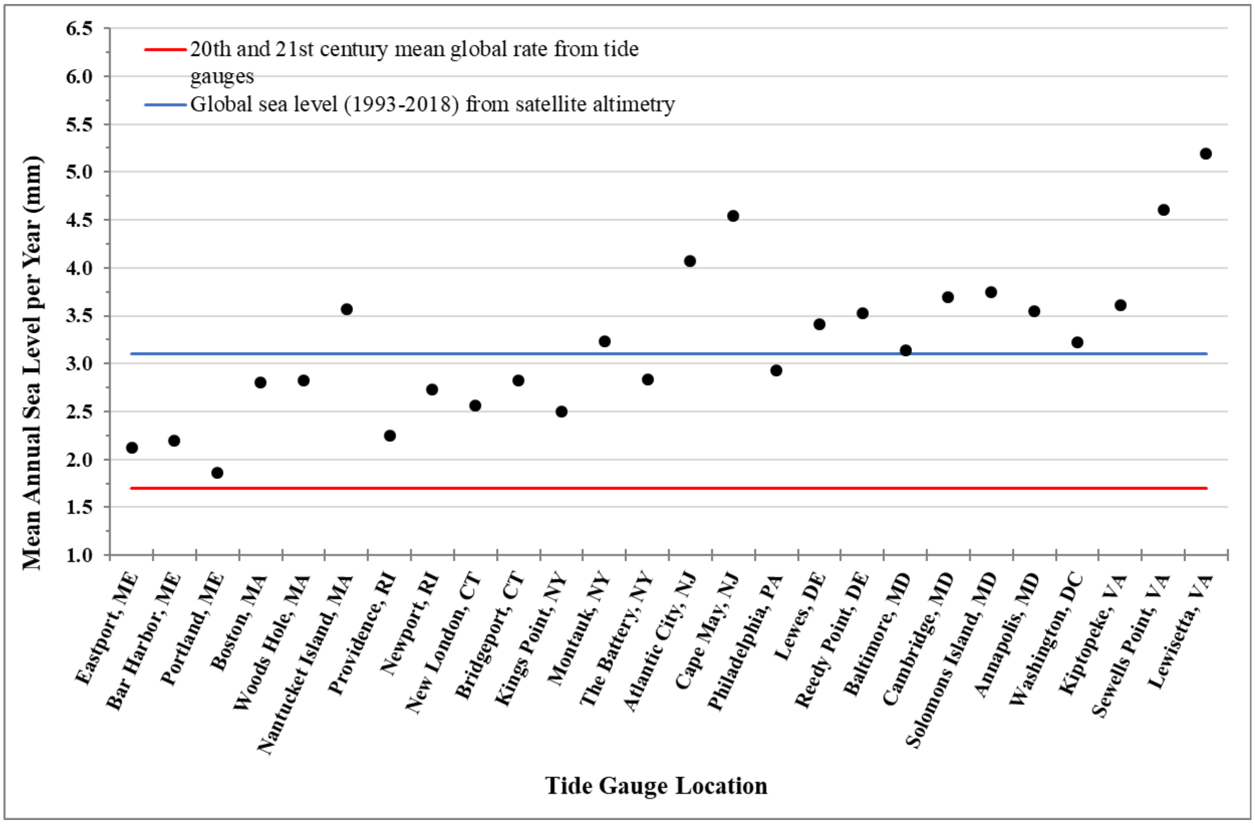

Projected Sea Level Rise

Key Points:

- IPCC (2013) projected that the global mean SLR for the period 2081–2100 (relative to 1986–2005) for the Intergovernmental Panel on Climate Change (IPCC) emissions pathway RCP8.5 will likely be in the range of 0.45–0.82 m. More recent studies have proposed higher levels of global SLR.
- This Northeast habitat climate vulnerability assessment is relying on a 1.0 m global (Intermediate) SLR scenario, which is projected to result in a 0.9 to 1.1 m increase in relative SLR, or a rate of 11-13 mm per year, for the study area in 2085.

Although significant uncertainties remain, including SLR related to the magnitude and rate of the ice-sheet contribution and the regional distribution of mass in the oceans, global mean sea level will continue to rise during the 21st century under all RCP scenarios. Furthermore, future SLR will very likely exceed the rate observed between 1971 and 2010 due to increased ocean warming and increased loss of mass from glaciers and ice sheets (IPCC 2013).

The global mean SLR for the period 2081–2100 (relative to 1986–2005) for IPCC emissions pathway RCP8.5 will likely be in the range of 0.45–0.82 m (IPCC 2013). There is little difference in global SLR projections between other emissions pathways during the first half of this century. However, ice sheets may be melting at a

rate faster than previously reported by IPCC (Bamber et al. 2009; Vaughan 2008; Velicogna 2009). Although complete loss of the ice sheets in the 21st century is unlikely, even a partial loss of large ice masses would have a significant effect on global sea levels. Using a coupled ice-sheet and climate dynamics model, DeConto and Pollard (2016) concluded Antarctica alone has the potential to contribute >1 m of global mean SLR by 2100 under the RCP8.5 emissions scenario.

Based upon the observational data and modeling related to rapid ice melt and ice sheet instability in Greenland and Antarctica available at the time, Sweet et al. (2017) presented six projections for global SLR for 2100: Low (0.3 m), Intermediate-Low (0.5 m), Intermediate (1.0 m), Intermediate-High (1.5 m), High (2.0 m), and Extreme (2.5 m). Based upon analyses conducted by Kopp et al. (2014), the probabilities of exceeding the Low and Intermediate-Low scenarios under RCP8.5 emissions scenario are 100% and 96%, respectively. The probabilities of exceeding the Intermediate, Intermediate-High, High, and Extreme SLR scenarios are 17%, 1.3%, 0.3%, and 0.1%, respectively.

For the Northeast Habitat Climate Vulnerability Assessment, a 1.0 m global (Intermediate) SLR scenario has been chosen. Although higher global SLR scenarios are feasible, especially as new research advances our understanding of ice-sheet dynamics, the 1.0 m SLR scenario is the most likely over the 21st century (W. Sweet, personal communication). Sweet et al. (2017) developed regional SLR projections for the U.S. coastline, including a number of locations on the east coast. Figure 3 depicts projected relative SLR for 18 east coast locations for 2085 using a 1.0 m (intermediate) global projection. Note that the 2085 projections shown in the figure are the mean of 2080 and 2090 relative SLR projections from Sweet et al. (2017).

Figure 3. Relative sea level rise projections (in meters) for 2085 on the U.S. east coast using the global intermediate scenario of 1.0 m (Sweet et al. 2017).

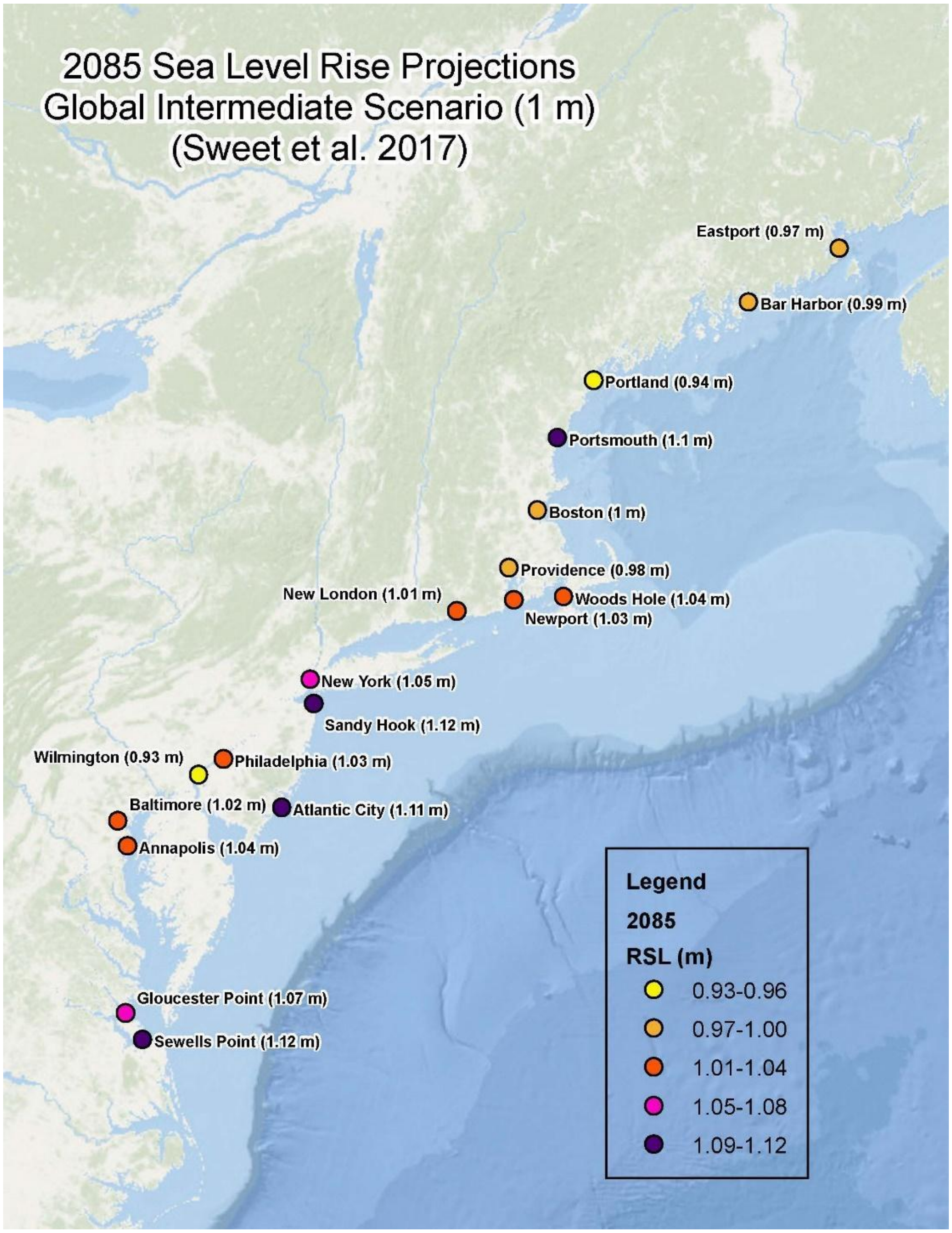

Using the projected relative SLR for locations on the east coast developed by Sweet et al. (2017), the rate of SLR for these locations were calculated for 2085 (Figure 4).

Figure 4. Rate of relative sea level rise (in millimeters per year) for 2085 in U.S. east coast cities using the global intermediate scenario of 1.0 m (Sweet et al. 2017).

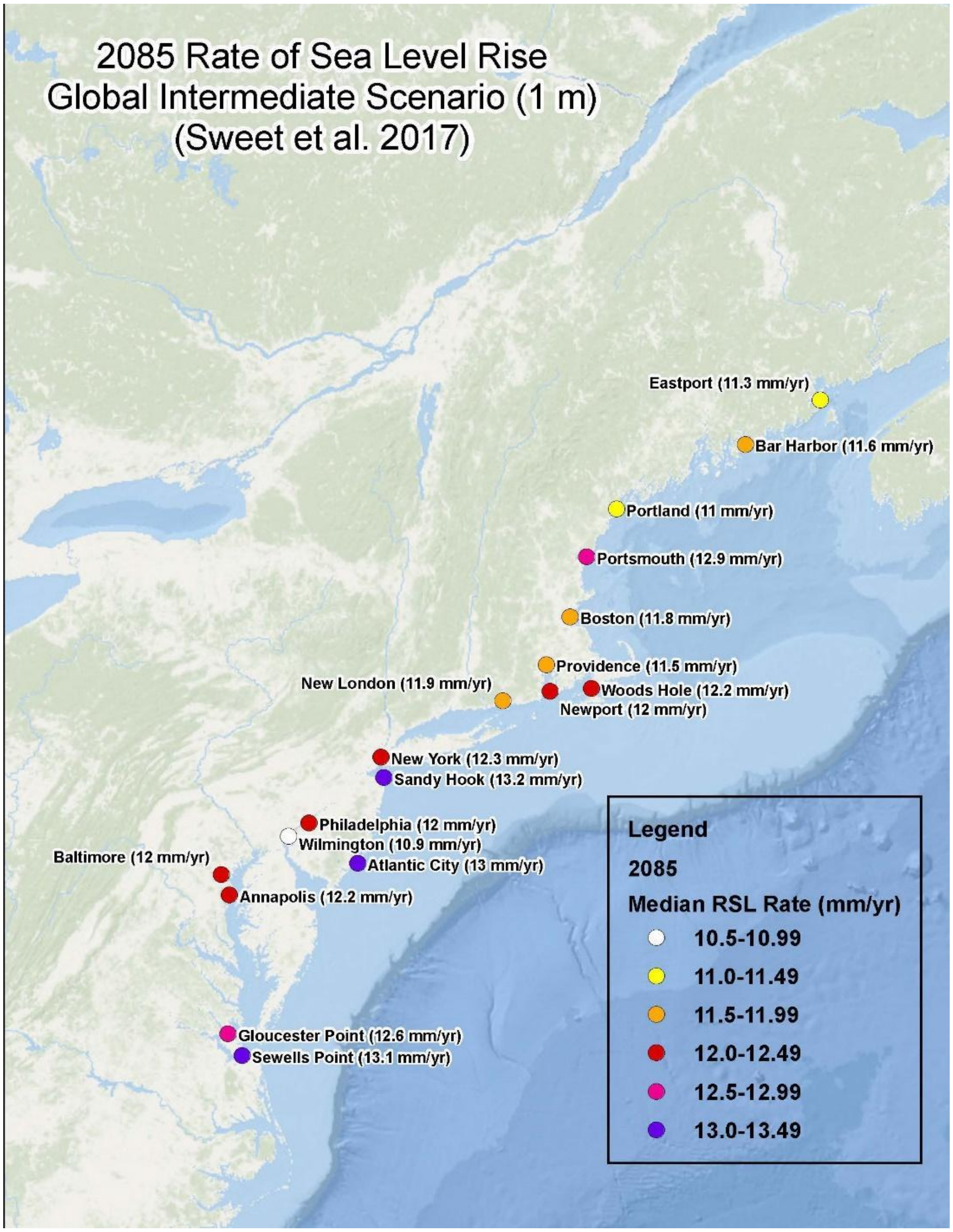

How to use Expert Opinion:

There is a continuum of spatial overlap for projected SLR and habitats, ranging from high or very high in the intertidal zone to effectively none in deep, offshore waters. Higher sea levels will have the greatest effects on intertidal habitats because they are within the vertical spatial range under a projected 0.9 to 1.1 m increase in relative SLR, or a rate of 11-13 mm per year scenario. Therefore, intertidal habitats should receive the highest exposure scores. Some spatial overlap exists in shallow, subtidal habitats with higher sea levels because these habitats will be converted to deeper-water habitats, and experience changing characteristics such as irradiance and exposure to surface water dissolved oxygen, temperature, and salinity. Therefore, shallow, subtidal habitats

should be scored, accordingly. Riverine habitats also experience some spatial overlap with SLR. In particular, the upper tidal reaches of rivers will have greater tidal influence and become more saline. The non-tidal riverine habitats will also experience some exposure to SLR as the salt wedge (e.g., the head of tide) moves further inland, increasing saltwater intrusion. Scores should be distributed to reflect this spatial overlap in both tidal and non-tidal riverine habitats. Deeper water benthic habitats share little to no vertical overlap with changes to sea levels, and should therefore receive a low score.

Using your expert knowledge of habitats, and the spatial distribution of the habitat subclasses under a projected 0.9 to 1.1 m increase in relative SLR, or a rate of 11-13 mm per year scenario for 2085, distribute the five tallies across the four bins (Low, Moderate, High, Very High) according to the following rubric:

1. Low: Habitat subclass is in deeper water or upper reach of rivers with little to no vertical overlap with rising sea levels and therefore, no exposure to projected SLR.
2. Moderate: Habitat subclass is somewhat within or immediately adjacent to the vertical spatial range and exposure of projected SLR.
3. High: Habitat subclass is partially within the vertical spatial range and exposure of projected SLR.
4. Very High: Habitat subclass is fully within the vertical spatial range and exposure of projected SLR.

References

Bamber JL, Riva REM, Vermeersen BLA, LeBrocq AM. 2009. Reassessment of the potential sea-level rise from a collapse of the West Antarctic Ice Sheet. Science 324(5929):901-3.

Bindoff N, Willebrand J, Artale V, Cazenave A, M. Gregory J, Gulev S, Hanawa K, Le Quéré C, Levitus S, Nojiri Y, Shum CK, Talley L, Alakkat U. 2007. Observations: oceanic climate change and sea level. Climate change 2007. The physical science basis. Contribution of Working Group I to the Fourth Assessment Report of the Intergovernmental Panel on Climate Change. [Solomon S, Qin D, Manning M, Chen Z, Marquis M, Averyt KB, Tignor M, Miller HL (eds)]. Intergovernmental Panel on Climate Change. p. 386-432.

Church JA, Clark PU, Cazenave A, Gregory JM, Jevrejeva S, Levermann A, Merrifield MA, Milne GA, Nerem RS, Nunn PD, Payne AJ, Pfeffer WT, Stammer D, Unnikrishnan AS. 2013. Sea level change. Climate change 2013. The physical science basis. Working Group I Contribution to the Fifth Assessment Report of the Intergovernmental Panel on Climate Change. [Stocker TF, Qin D, Plattner G-K, Tignor M, Allen SK, Boschung J, Nauels A, Xia Y, Bex V, Midgle PM (eds)]. Intergovernmental Panel on Climate Change. p. 1137-216.

Church JA, White NJ. 2011. Sea-level rise from the late 19th to the early 21st century. Surveys in Geophysics 32(4-5):585-602.

Church JA, White NJ, Aarup T, Wilson WS, Woodworth PL, Domingues CM, Hunter JR, Lambeck K. 2008. Understanding global sea levels: past, present and future. Sustainability Science 3(1):9-22.

DeConto RM, Pollard D. 2016. Contribution of Antarctica to past and future sea-level rise. Nature 531(7596):591-7.

DeJong BD, Bierman PR, Newell WL, Rittenour TM, Mahan SA, Balco G, Rood DH. 2015. Pleistocene relative sea levels in the Chesapeake Bay region and their implications for the next century. GSA Today:4-10.

Eggleston J, Pope J. 2013. Land subsidence and relative sea-level rise in the southern Chesapeake Bay region. Reston, Virginia: U.S. Department of the Interior, U.S. Geological Survey. p. 1-30.

Hay CC, Morrow E, Kopp RE, Mitrovica JX. 2015. Probabilistic reanalysis of twentieth-century sea-level rise. Nature 517(7535):481-4.

IPCC, Intergovernmental Panel on Climate Change. 2013. Summary for policymakers. Climate change 2013. The physical science basis. Contribution of Working Group I to the Fifth Assessment Report of the Intergovernmental Panel on Climate Change. [Stocker T, Qin D, Plattner G-K, Tignor M, Allen S, Boschung J, Nauels A, Xia Y, Bex V, Midgley P (eds)]. Intergovernmental Panel on Climate Change. p. 1-29.

IPCC, Intergovernmental Panel on Climate Change. 2014. Climate change 2014. Synthesis report. Contribution of Working Groups I, II and III to the Fifth Assessment Report of the Intergovernmental Panel on Climate Change. [Core WT, Pachauri R, Meyer L (eds)]. Intergovernmental Panel on Climate Change. p. 1-151.

Kemp AC, Horton BP, Donnelly JP, Mann ME, Vermeere M, Rahmstorff S. 2011. Climate related sea-level variations over the past two millennia. Proceedings of the National Academy of Sciences of the United States of America 108(27):11017-22.

Kopp RE, Horton RM, Little CM, Mitrovica JX, Oppenheimer M, Rasmussen DJ, Strauss BH, Tebaldi C. 2014. Probabilistic 21st and 22nd century sea-level projections at a global network of tide-gauge sites. Earth's Future 2(8):383-406.

Kopp RE, Kemp AC, Bittermann K, Horton BP, Donnelly JP, Gehrels WR, Hay CC, Mitrovicak JX, Morrowa ED, Rahmstorf S. 2016. Temperature-driven global sea-level variability in the Common Era. Proceedings of the National Academy of Sciences of the United States of America 113(11):E1434-41.

Levermann A, Griesel A, Hofmann M, Montoya M, Rahmstorf S. 2005. Dynamic sea level changes following changes in the thermohaline circulation. Climate Dynamics 24(4):347-54.

NOAA Tides and Currents. 2020. Website accessed 2020 Mar. 18. https://tidesandcurrents.noaa.gov/sltrends/sltrends.html.

Sallenger AH, Doran KS, Howd PA. 2012. Hotspot of accelerated sea-level rise on the Atlantic coast of North America. Nature Climate Change 2(12):884-8.

Sweet WV, Kopp RE, Weaver CP, Obeysekera J, Horton RM, Thieler ER, Zervas C. 2017. Global and regional sea level rise scenarios for the United States. National Oceanic and Atmospheric Administration, National Ocean Service. NOAA Technical Report NOS CO-OPS 083. p. 1-56.

Univ. of Colorado Sea Level Research Group. 2020. Website accessed 2020 Mar. 18. http://sealevel.colorado.edu/.

Vaughan DG. 2008. West Antarctic Ice Sheet collapse – the fall and rise of a paradigm. Climatic Change 91(1-2):65-79.

Velicogna I. 2009. Increasing rates of ice mass loss from the Greenland and Antarctic ice sheets revealed by GRACE. Geophysical Research Letters 36(19).

Yin J, Schlesinger ME, Stouffer RJ. 2009. Model projections of rapid sea-level rise on the northeast coast of the United States. Nature Geoscience 2(4):262-6.

Exposure Factor: Streamflow

Changes in streamflow volume and timing are driven by a complex array of factors. They are in large part driven by changes in precipitation, including extreme events, as well as by evapotranspiration, groundwater contributions, and in some areas snow/ice melt. Local land use, land-cover changes, and water management also play a role in streamflow (USGCRP 2017). Changes in streamflow magnitude, frequency, and timing can impact riverine habitats and the aquatic species that rely on them throughout their life cycle (Demaria et al. 2016; Blum et al. 2018). Increases in high flow events can cause streambed erosion and increased sediment, nutrient, and microbial pathogen delivery to streams, while droughts and decreases in low flow volume can expose aquatic life to high temperatures and low dissolved oxygen (USGCRP 2017).

Historic changes in streamflow

In the past century, stream discharges for rivers with near-natural streamflow in New England and the Mid-Atlantic have generally increased (Hodgkins and Dudley 2005; Lins and Slack 2005). There has also been a historical increase in the magnitude and frequency of floods in Northeast U.S. rivers, with a step increase around 1970 (Collins 2009; Armstrong et al. 2012, 2014). While catastrophic floods (i.e., occurring less frequently than once every 10 years) can cause significant erosion, frequent, low-magnitude floods are responsible for most of the sediment flux in a watershed and channel formation of streams (Armstrong et al. 2012). Floods in the Northeast U.S. can occur any time of year, but they most commonly occur in the late winter-early spring and some parts of the region have a secondary flood season in the fall and/or winter. Despite the warm season typically being a flood-poor time of year in the Northeast, recent trends in flood frequency have been driven by increases in warm season (Jun-Oct) flood counts (Frei et al. 2015; Collins 2018).

The timing of snowmelt-related streamflow has changed in New England. Dudley et al. (2017) found that the winter-spring center volume date (reflecting the timing of spring snowmelt) has shifted earlier (average of roughly eight days).

Projected changes in streamflow

The intensity and frequency of heavy precipitation events are projected to increase (USGCRP 2017). Monthly precipitation will also change, but the trends in monthly precipitation are not spatially or seasonally uniform (Johnson et al. 2019). Precipitation is projected to increase in the winter and spring, with minimal change in the summer and fall, and larger overall changes in New England (USGCRP 2017). By the end of the century (2070-2100) under the RCP 8.5 scenario, monthly precipitation is projected to be approximately 1 inch greater for December through April in the Northeast (USGRCP 2017). (See supplemental figures 1 and 2 below for seasonal projections, and the CMIP5 precipitation file for annual projection).

Studies where hydrology models were driven by climate models indicate increased variability in streamflow, with more high-flow events and more low-flow events (Hayhoe et al. 2007). Several studies showed a projected shift toward higher winter flows and lower spring flows (Campbell et al. 2011; Hayhoe et al. 2007). The timing of streamflow is also expected to continue to change. As northeast winters warm, precipitation is expected to fall as rain rather than snow, and snowmelt-related runoff will occur earlier, leading to earlier peak streamflows (USGCRP 2017). Spring peak flows are projected to become earlier by 10-15 days at the end of the century (Hayhoe et al. 2007).

Demaria et al. (2016) published the only region-wide study evaluating changes in streamflow based on climate projections. They evaluated changes to three streamflow characteristics in the Northeast based on mid-century climate projections: 3-day peak flows, 7-day low flows, and mean base flows. The 3-day peak flows are the maximum annual streamflows over a three-day period (indicative of floods) (Das et al. 2011). The 7-day low flows are the average flow measured during the 7 consecutive days of lowest flow during any given year, and are an indicator of low flow conditions during drought (USGS 2008). Droughts can be damaging to riverine habitats and the aquatic species that rely on them. Mean base flows are representative of the groundwater contribution to streamflow throughout the year. Under RCP 8.5 (2028-2082) in the Northeast, Demaria et al. (2016) found the following (Note that all of these findings are based on mid-century projections, while the Northeast HCVA is assessing climate exposure under end-of-century projections. However, they are likely representative of general trends expected to continue into the end of the century):

Base flow:

- Mix of positive and negative trends (statistically insignificant) in annual mean base flow (Figure 2).

Peak flow (indicative of floods):

- Positive trends in 3-day peak flows due to more intense precipitation, except for northern New England, where peak flows show a downward trend (Figure 2).
- Up to 40% increase in the estimated magnitude of 3-day peak flows with a 100-year return period by mid-century in the Mid-Atlantic (indicating higher high flows), but decreases in parts of New England and New York—especially northern and interior sections (Figure 3). This metric represents the highest of the highs.
- Increase in the frequency of daily flows exceeding the 90th percentile of all daily flows (approximately 20% increase throughout the Northeast), indicating higher frequency of flooding.

Low flow (indicative of droughts):

- Widespread negative trends in 7-day low flows (Figure 2). Negative trends refer to a decreasing trend in the amount of water, in other words a lower low flow. Hayhoe et al. (2007) predicted a similar change, with more extensive low-flow periods during summer months with lower flows (less water).
- Up to 90% decrease in the estimated magnitude of 7-day low flows with a 100-year return period by mid-century throughout the region (indicating lower low flows) (Figure 3). This metric represents the lowest of the lows.
- Variability in frequency of daily flows below the 10th percentile of all daily flows across the range, with an 8.4% decrease in frequency in New England due to increased precipitation, and 13.6% increase in frequency in the Mid-Atlantic. The length of the low flow season is also projected to increase by an average of 5±3 days (18%) across the region.

In summary, Demaria found: (a) Mid-Atlantic: increasing magnitude and frequency of floods; increasing magnitude and frequency of low flow events, and (b) for New England: decreasing magnitude of peak flows, but increasing frequency; increasing magnitude of low flows, but decreasing frequency.

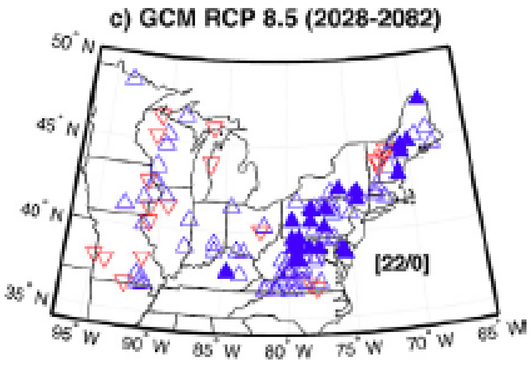

Fig.1 (left). Linear trends in annual maximum cumulative 5-day precipitation for the GMC ensemble-mean: RCP 8.5 future period (2028–2082). Statistically significant trends are shown with filled triangles (0.05). Upward pointing triangles indicated positive trends and downward pointing ones denote negative trends. (Source: Demaria et al. 2016).

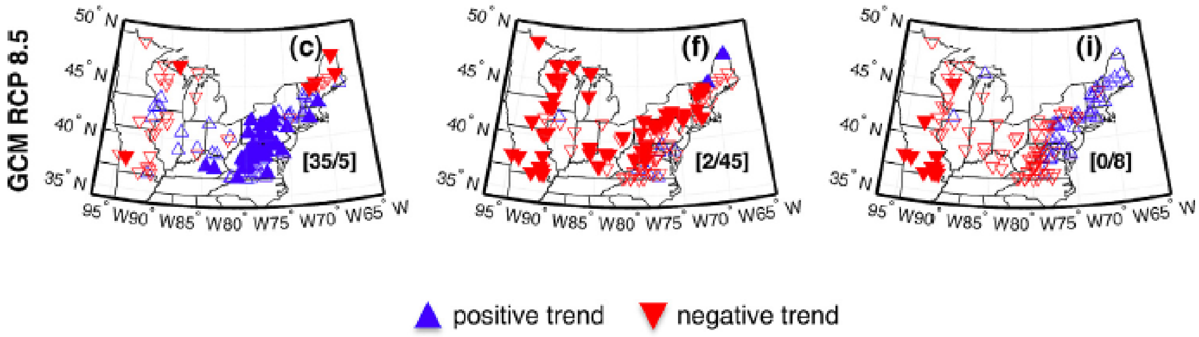

Fig 2 (above). Trends in GCM-driven simulations of 3-day peak flows (left column, c), 7-day low flows (middle column, f), and annual pre-whitened mean base flows (right column, i), during the RCP 8.5 future (2028–2082) periods. The numbers in the lower right corner indicate the percentage of basins with statistically significant trends. (Source: Demaria et al. 2016).

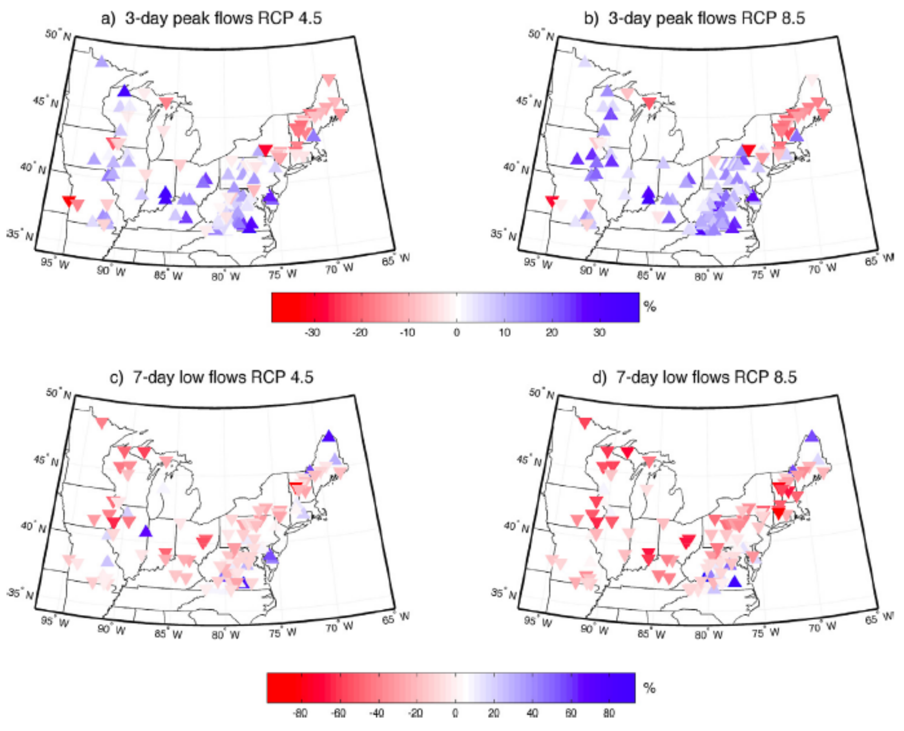

Fig. 3 Percentage changes in the magnitude of the 100-year return period 3-day peak flow (top panels) and 7-day low flows (bottom panels). Changes are expressed as percentage change from the historical period (1951–2005). (Source: Demaria et al. 2016).

Scoring Exposure to Changes in Stream Flow

Using your knowledge of the distribution of the riverine habitat subclasses, and the projected changes in streamflow, distribute the five tallies across the four bins (Low, Moderate, High, Very High) according to the following rubric. When considering whether changes will be "moderate" or "significant", consider impacts on the function of the habitat:

Floods:

1. Low: Peak flows (floods) are not projected to change in the region.
2. Moderate: Peak flows (floods) are projected to increase in magnitude or frequency, but not both.
3. High: Peak flows are projected to increase in magnitude and frequency to a moderate degree.
4. Very High: Peak flows are projected to increase in magnitude and frequency to a significant degree.

Droughts:

1. Low: Low flows (droughts) are not projected to change in the region.
2. Moderate: Low flows (droughts) expected to increase in magnitude or frequency, but not both.
3. High: Low flows are projected to increase in magnitude (become more extreme lows) and frequency to a moderate degree.
4. Very High: Low flows are projected to increase in magnitude (become more extreme lows) and frequency to a significant degree.

References

Armstrong, W.H., Collins, M.J., and Snyder N.P. 2012. Increased Frequency of Low‐Magnitude Floods in New England. Journal of the American Water Resources Association (JAWRA) 48(2): 306‐320. DOI: 10.1111/j.1752‐1688.2011.00613.x

Armstrong, W.H., Collins, M.J., and Snyder N.P. 2014. Hydroclimatic flood trends in the northeastern United States and linkages with large-scale atmospheric circulation patterns, Hydrological Sciences Journal, 59(9): 1636-1655. DOI: 10.1080/02626667.2013.862339

Blum, A.G., Kanno, Y.,, and Letcher, B.H. 2018. Seasonal streamflow extremes are key drivers of Brook Trout young-of-the-year abundance. Ecosphere 9(8).

Campbell, J. L., Driscoll, C.T., Pourmokhtarian, A., and Hayhoe, K. 2011. Streamflow responses to past and projected future changes in climate at the Hubbard Brook Experimental Forest, New Hampshire, United States. Water Resources Research 47(2). DOI: 10.1029/2010WR009438.

Collins, M.J. 2009. Evidence for Changing Flood Risk in New England Since the Late 20th Century. Journal of the American Water Resources Association 45: 279‐290. DOI: 10.1111/j.1752‐1688.2008.00277.x

Collins, M.J. 2018. River flood seasonality in the Northeast United States: Characterization and trends. Hydrological Processes 33(5): 687-698. DOI: 10.1002/hyp.13355

Das, T., Dettinger, M.D., Cayan, D.R., Hidalgo, H.G. 2011. Potential increase in floods in California's Sierra Nevada under future climate projections. Clim.Change 109: 71–94. DOI: 10.1016/j.jhydrol.2017.01.051

Demaria, E.M.C., Palmer, R.N., and Round, J.K. 2016. Regional climate change projections of streamflow characteristics in the Northeast and Midwest U.S. Journal of Hydrology: Regional Studies 5: 309-323.

Dudley, R.W., Hodgkins, G.A., McHale, M.R., Kolian, M.J., and Renard, B. 2017. Trends in snowmelt-related streamflow timing in the conterminous United States. Journal of Hydrology 547: 208-221.

Frei, A., Kunkel, K. E., and Matonse, A. 2015. The seasonal nature of extreme hydrological events in the Northeastern United States. American Meteorological Society 16: 2065-2085. DOI: 10.1175/JHM-D-14-0237.1

Hayhoe, K., Wake, C.P., Huntington, T.G., Luo, L., Schwartz, M.D., Sheffield, J., Wood, E., Anderson, B., Bradbury, J., DeGaetano, A., Troy, T.J., and Wolfe, D. 2007. Past and future changes in climate and hydrological indicators in the US Northeast. Climate Dynamics 28: 381–407. DOI: 10.1007/s00382-006-0187-8

Hodgkins, G.A. and Dudley, R.W., 2005. Changes in the magnitude of annual and monthly streamflows in New England, 1902–2002. U.S. Geological Survey Scientific Investigations Report 2005–5135, 44 pp.

Johnson, M.R, Boelke, C, Chiarella, L.A, and Greene, K. 2019. Guidance for Integrating Climate

Change Information in Greater Atlantic Region Habitat Conservation Division Consultation Processes. Greater Atlantic Region Policy Series 19-01. NOAA Fisheries Greater Atlantic Regional Fisheries Office - www.greateratlantic.fisheries.noaa.gov/policyseries/. 235p.

Lins, Harry F. and James R. Slack. 2005. Seasonal and Regional Characteristics of U.S. Streamflow Trends in the United States from 1940 to 1999, Physical Geography, 26:6, 489-501, DOI: 10.2747/0272-3646.26.6.489

USGCRP. 2017: *Climate Science Special Report: Fourth National Climate Assessment, Volume I* [Wuebbles, D.J., D.W. Fahey, K.A. Hibbard, D.J. Dokken, B.C. Stewart, and T.K. Maycock (eds.)]. U.S. Global Change Research Program, Washington, DC, USA, 470 pp, doi: 10.7930/J0J964J6.

USGS. 2008. Calculating flow-duration and low-flow frequency statistics at streamflow-gaging stations. U.S. Geological Survey Scientific Investigations Report 2008-5126. https://pubs.usgs.gov/sir/2008/5126/section3.html

Supplementary Figures

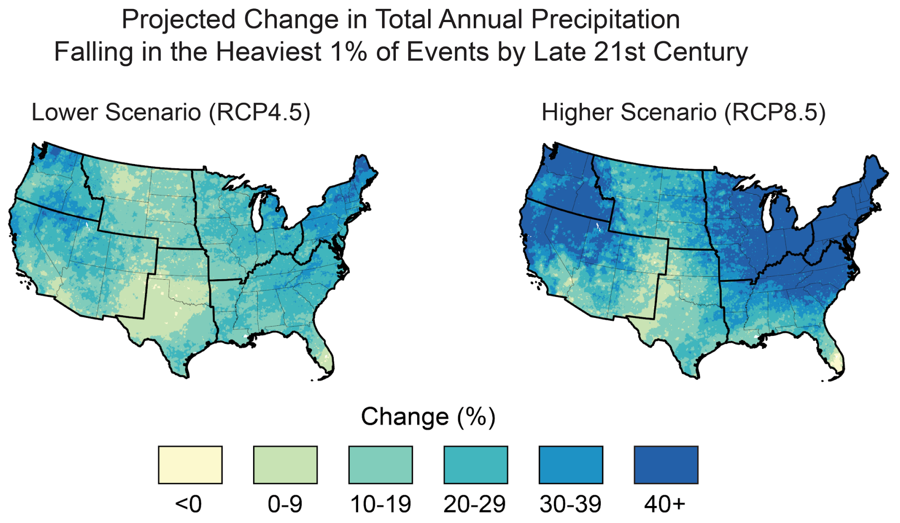

Supplementary Figure 1: (Source: USGCRP 2017)

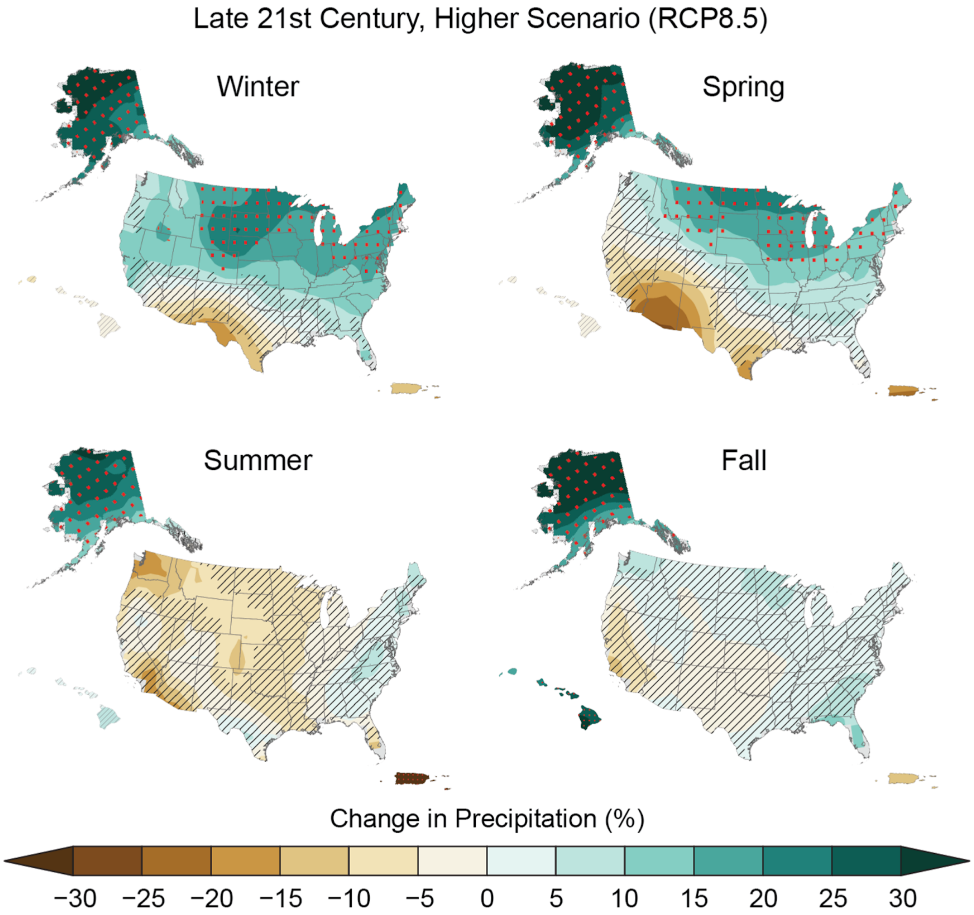

Supplementary Figure 2: Observed and projected precipitation changes vary by season and region. Areas with red dots show where projected changes are large compared to natural variations; areas that are hatched show where changes are small and relatively insignificant. (Source: USGCRP 2017)

Stream Temperature Exposure Factor

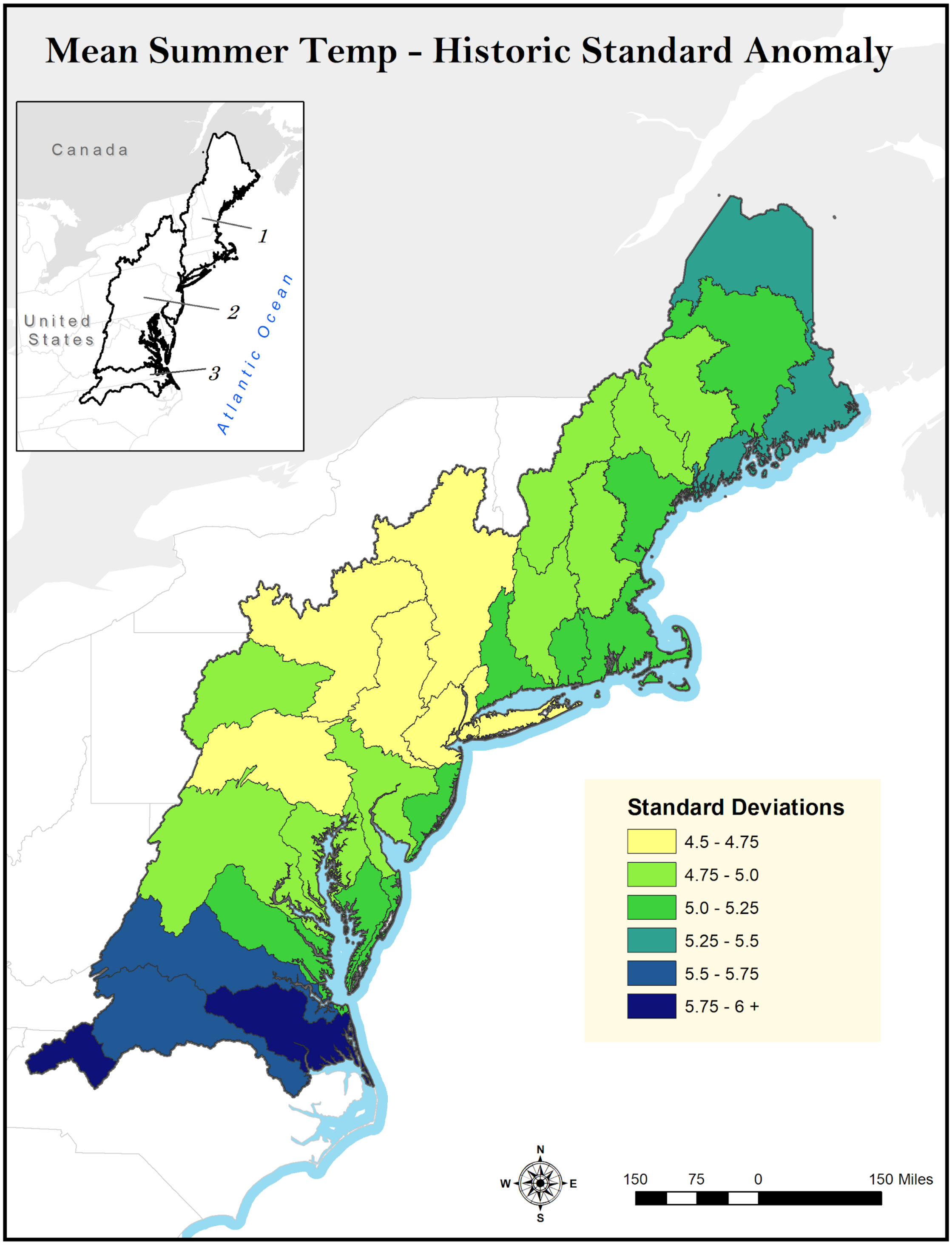

Figure 1: Mean summer temperature standardized historic anomaly values for HUC6 hydrologic units in the Northeast U.S. under +4C mean summer air temperature scenario (a proxy for RCP 8.5 end of century scenario).

This map of stream temperature projections was created using the USGS SHEDS Stream Temperature Model (Letcher et al. 2016). A detailed description of the model can be found here. It is important to note that the model does not include temperatures for major rivers in the analysis (anything beyond third order streams), largely due to the significant influence of human activities on temperatures in larger rivers. The map depicts a single value (standardized historic anomaly) across each HUC6 hydrologic unit. This value represents the weighted spatial mean of all catchments within the HUC6, excluding rivers larger than third order streams.

The scoring bins represent the standardized historic anomaly comparing mean summer stream temperatures under a future scenario based on a mean summer air temperature anomaly of +4C with the historic stream temperature from 1980-2018. A mean summer air temperature anomaly of +4C is a proxy to represent regional end-of-century projections for air temperature based on the Intergovernmental Panel on Climate Change RCP 8.5 emissions scenario (Figure 2).

Scoring bins:

- Low: <1.5 standard deviations
- Moderate: 1.5-4.0 standard deviations
- High: 4.0-5.5 standard deviations
- Very High: >5.5 standard deviations

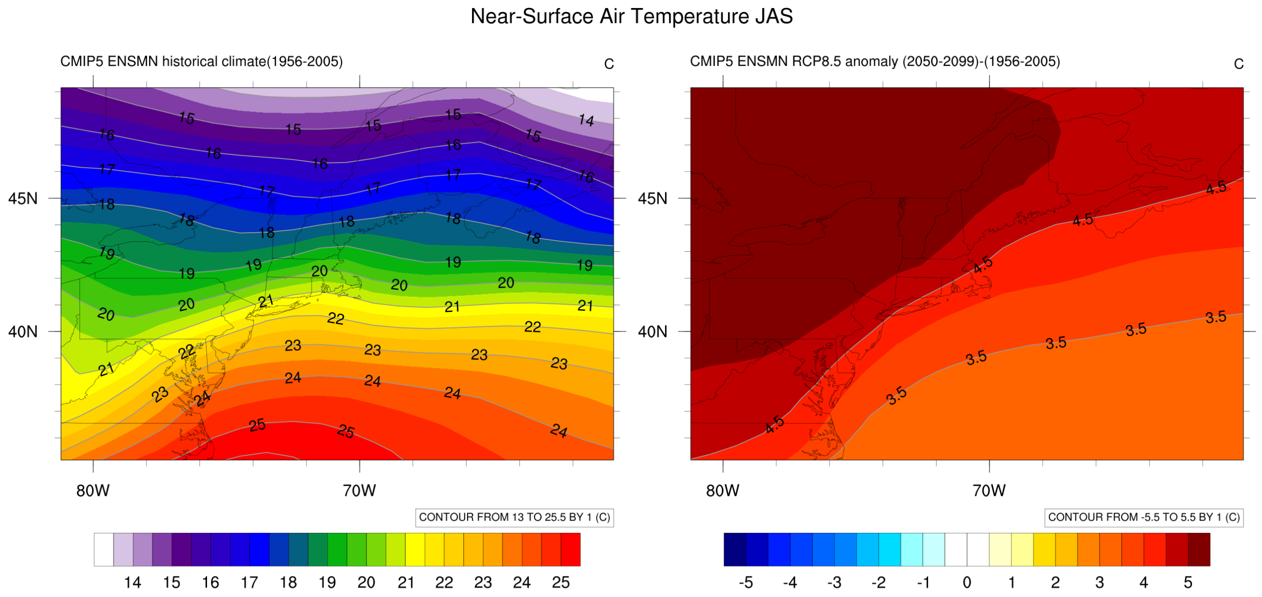

Figure 2: CMIP5 end-of-century air temperature projection under IPCC RCP 8.5 emissions scenario. The left panel is the historic temperature, and the right panel is the end-of-century anomaly.

References

Letcher, Benjamin H., Daniel J. Hocking, Kyle O'Neil, Andrew R. Whiteley, Keith H. Nislow, and Matthew J. O'Donnell. 2016. "A Hierarchical Model of Daily Stream Temperature Using Air-Water Temperature Synchronization, Autocorrelation, and Time Lags." *PeerJ* 4: e1727. doi:10.7717/peerj.1727.
