# Supplementary material for: An assessment of marine, estuarine, and riverine habitat vulnerability to climate change in the Northeast U.S
Source: PLoS One. 2021 Dec 9;16(12):e0260654. doi: 10.1371/journal.pone.0260654 (PMC8659346; doi:10.1371/journal.pone.0260654)
Supplement: S4 File — Detailed scoring results for and descriptions of the climate impacts on each habitat including the key drivers of vulnerability, data quality and gaps, and background information on the habitat. (PDF) [file pone.0260654.s004.pdf]

Table of Contents

Marine Habitats

Marine Intertidal Shellfish Reef.......... 3
Marine Intertidal Rocky Bottom.......... 10
Marine Intertidal Mud Bottom.......... 15
Marine Intertidal Sand Bottom.......... 21
Marine Kelp.......... 28
Marine Red, Green, Small-brown Algae.......... 34
Marine Submerged Aquatic Vegetation.......... 41
Marine Subtidal Shellfish Reef.......... 47
Marine Rocky Bottom <200 m.......... 54
Marine Mud Bottom <200 m.......... 60
Marine Sand Bottom <200 m.......... 67
Deep Sea Coral & Sponge: Gulf of Maine.......... 72
Deep Sea Coral & Sponge: Seamounts and Canyons Marine.......... 81
Rocky Bottom >200 m.......... 90
Marine Mud Bottom >200 m.......... 96
Marine Sand Bottom >200 m.......... 102
Marine Shellfish Aquaculture.......... 108
Marine Artificial Structures.......... 113
Marine Shallow Inner Shelf Water Column.......... 118
Marine Shelf Surface Water Column.......... 123
Marine Shelf Bottom Water Column.......... 128
Marine Slope Surface Water Column.......... 133
Marine Slope Bottom Water Column.......... 139

Estuarine Habitats

Estuarine Invasive Wetland: Mid-Atlantic.......... 143
Estuarine Invasive Wetland: New England.......... 149
Estuarine Native Wetland: Mid-Atlantic.......... 155
Estuarine Native Wetland: New England.......... 161
Estuarine Intertidal Shellfish Reef.......... 168
Estuarine Intertidal Rocky Bottom.......... 176
Estuarine Intertidal Artificial Structures.......... 183
Estuarine Intertidal Mud Bottom.......... 187
Estuarine Intertidal Sand Bottom.......... 192
Estuarine Kelp.......... 199
Estuarine Red, Green, Small-brown Algae.......... 205
Estuarine Submerged Aquatic Vegetation.......... 212
Estuarine Subtidal Shellfish Reef.......... 219

Estuarine Subtidal Rocky Bottom.. 227
Estuarine Shellfish Aquaculture.. 233
Estuarine Subtidal Artificial Structures.. 238
Estuarine Subtidal Mud Bottom.. 242
Estuarine Subtidal Sand Bottom... 248
Estuarine Water Column... 255

Riverine Habitats

Riverine Non-tidal Invasive Wetland.. 260
Riverine Non-tidal Native Wetland... 264
Riverine Tidal Invasive Wetland... 269
Riverine Tidal Native Wetland.. 273
Riverine Algae.. 278
Riverine Submerged Aquatic Vegetation.. 283
Riverine Rocky Bottom... 288
Riverine Mud Bottom.. 295
Riverine Sand Bottom... 301
Riverine Water Column.. 308

Marine Intertidal Shellfish Reef

System: Marine

Subsystem: Intertidal

Class: Reef

Sub-class: Mollusk

Geographic Area: Entire Area

Overall Vulnerability Rank = Very High

Habitat Sensitivity = High

Climate Exposure = Very High

	Marine Intertidal Shellfish Reef	Attribute Mean	Data Quality	Distribution of Expert Scores
Sensitivity Attributes	Habitat condition	2.8	2	
	Habitat fragmentation	2.8	1.8	
	Distribution/Range	2.5	2.2	
	Mobility/Ability to spread or disperse	2.1	1.9	
	Resistance	2.2	1.9	
	Resilience	2.8	2.2	
	Sensitivity to changes in abiotic factors	3.2	2.3	
	Sensitivity and intensity of non−climate stressors	3.7	2.4	
	Dependency on critical ecological linkages	2.7	1.6	
	Sensitivity Component Score	High		
Exposure Factors	Sea surface temp	n/a	n/a	
	Bottom temp	n/a	n/a	
	Air temp	4	3	
	River temp	n/a	n/a	
	Surface salinity	1.3	2.5	
	Bottom salinity	n/a	n/a	
	pH	4	2	
	Sea level rise	3.9	2.2	
	Precipitation	2.5	2.1	
	Floods	n/a	n/a	
	Droughts	n/a	n/a	
	Exposure Component Score	Very High		
**Overall Vulnerability Rank**		**Very High**		

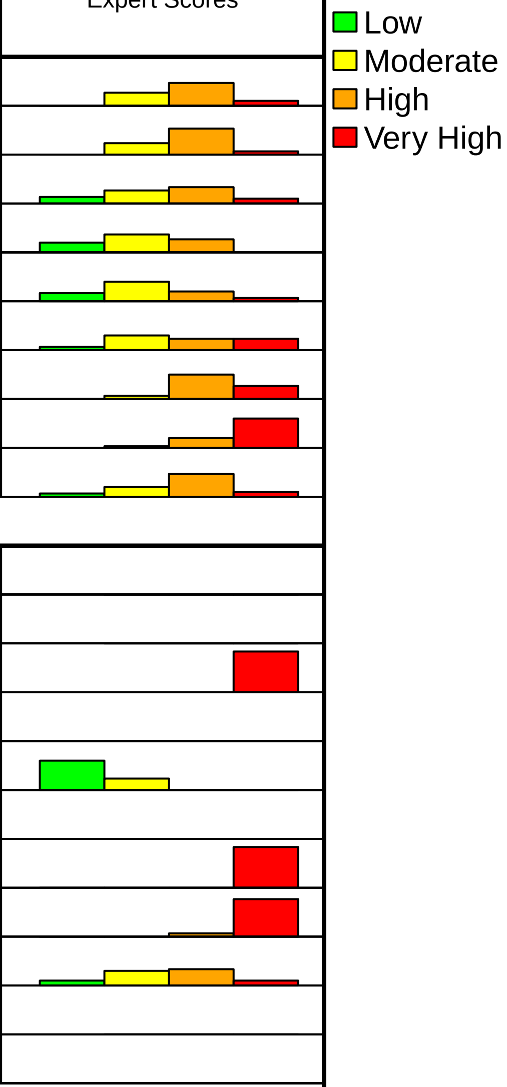

Habitat Name: Marine Intertidal Shellfish Reef

System**:** Marine

Subsystem: Intertidal

Class: Reef

Sub-class: Mollusk

Geographic Area: Entire Area

<u>Habitat Description</u>: This sub-class includes intertidal, reef-building shellfish that create a biotic hard substrate on benthic substrates in marine waters >30 ppt. The most common species in the study area are blue mussels (*Mytilus edulis*) and eastern oyster (*Crassostrea virginica*). Bivalves like oysters and mussels grow in dense aggregations forming reefs that extend off the seafloor. Oysters range from the Gulf of St. Lawrence, Canada to the Yucatan, West Indies and Brazil (Gunter 1951). Suitable substrates consist of sand, firm mud, or clay, whereas shifting sand and extremely soft mud substrates are generally the only unsuitable substrates for oyster reef habitats (Galstoff 1964; Bahr and Lanier 1981). The blue mussel is a cosmopolitan species common to temperate and polar waters in the northern Atlantic Ocean from the southern Canadian Maritime provinces to North Carolina. Shellfish reefs are common in the intertidal and subtidal down to 10m.

Shellfish reefs serve as habitat for diverse assemblages of polychaetes, crustaceans, and other resident invertebrate and fish species (Wells 1961; Bahr and Lanier 1981; Rothschild et al. 1994; Coen et al. 1999; Peterson et al. 2003; zu Ermgassen et al. 2016). They are filter feeders, thereby promoting greater water clarity and benthic productivity (Dame et a. 1984; Newell 1988; Ulanowicz and Tuttle 1992; Paerl et al. 1998). Shellfish reefs also remove excess nitrogen from coastal estuaries by promoting bacterially mediated denitrification as a consequence of concentrating bottom deposits of feces and pseudofeces (Newell et al. 2002; Piehler and Smyth 2011).

<u>Overall Climate Vulnerability Rank</u>: Very High (99% certainty from bootstrap analysis).

<u>Climate Exposure</u>: Very High. The overall very high exposure score was influenced by three Very High attribute means: Air Temperature (4.0), pH (4.0), and Sea Level Rise (SLR) (3.9). The exposure attribute score for Precipitation (2.5) was Moderate to High, whereas Surface Salinity (1.3) was relatively Low. SLR is expected to impact the entire study area but the greatest relative rise is projected in the Mid-Atlantic. The intertidal nature of this habitat places it at the nexus of significant atmospheric and oceanic change.

<u>Habitat Sensitivity</u>: High. Six of the nine sensitivity attribute means were ≥2.7: Habitat Condition (2.8), Habitat Fragmentation (2.8), Resilience (2.8), Sensitivity to Changes in Abiotic Factors (3.2), Sensitivity and Intensity of Non-Climate Stressors (3.7), and Dependency on Critical Ecological Linkages (2.7). The attribute means for Mobility/Ability to Spread or Disperse (2.1), Resistance (2.2) and Distribution/Range (2.5) were Moderate.

<u>Data Quality & Gaps</u>: Data quality for the four climate exposure factors were predominantly Moderate: Surface Salinity (2.5), SLR (2.2), pH (2) and Precipitation (2.1), while the Air Temperature was the highest (3.0). The relatively low score for Precipitation is due to uncertainty in the specific spatial nature of projected changes in extreme events. In addition, data quality is lower for nearshore coastal habitats due to the low resolution of CMIP5 and ROMS-NWA projections for climate exposure in nearshore, shallow coastal areas. Another uncertainty factor is the relative disparate spatial data for mollusk reefs in some portions of the study area.

Data quality scores for all of the nine habitat sensitivity attributes were scored between 1.6 and 2.4, with Habitat Fragmentation (1.8), Mobility/Ability to Spread or Disperse (1.9), Resistance (1.9) and Dependency on Critical Ecological Linkages (1.6) under 2.0. The two highest data quality scores were Sensitivity and Intensity of Non−Climate Stressors (2.4) and Sensitivity to Changes in Abiotic Factors (2.3). Although the climate sensitivity for marine subtidal mollusk reefs are expected to be high, there remains some uncertainty in species' capacity to adapt and respond to climate change (e.g., synergistic effects of climate and non-climate stressors).

Relatively moderate to low data quality scores could reflect the fact that few comprehensive assessments for shellfish habitat exist in the study area as much of it was lost centuries ago (Kirby 2004; Rothschild et al. 1994; zu Ermgassen et al. 2012).

Positive or Negative Climate Effect for the Northeast U.S.: The effect of climate change on marineintertidal shellfish habitats in the Northeast U.S. is expected to be negative (90% of the experts' scores were negative, and 10% were neutral).

Climate Effects on Habitat Condition and Distribution: Several studies have examined the effects of climate and non-climate impacts on shellfish reefs. While overharvesting has historically been attributed to oyster reef habitat loss in the northeast and elsewhere, hypoxia, disease, predators, competition, and sedimentation have impeded recent restoration efforts (Rothschild et al. 1994; Kirby 2004; Beck et al. 2011; zu Ermgassen et al. 2012). In addition, there is growing evidence that suggests potential climate effects on shellfish reefs may also be a factor.

Increasing seawater temperatures may affect the condition and distribution of intertidal shellfish in nearshore marine waters. For example, southern populations of blue mussels appear to be shifting northward in response to warming temperatures (Jones et al. 2010). Thermal stress, in conjunction with ocean acidification conditions, was found to cause metabolic depression in blue mussels from the Gulf of Maine (Lesser 2016), suggesting that elevated temperatures may result in suboptimal conditions for this cold-adapted species. Oyster growth and reproductive rates peak in waters ranging in temperature from 20-30°C and can live in water temperatures of 0-36°C (Shumway 1996; Lenihan 1999). In comparison, mussels are common in colder, more temperate to polar waters, thriving in 5-20°C, with an upper thermal tolerance limit of 29°C (Animal Diversity Web 2020).

Although the ROMS-NWA projections limit the ability to make precise climate predictions on impacts to marine intertidal shellfish reefs, water and air temperatures are projected to increase compared to historic means. The ROMS-NWA projections for RCP8.5 indicate the standardized anomaly for sea surface temperature will increase by at least 4 standard deviation from the historic means, and higher in the Gulf of Maine. In addition, CMIP5 projections indicate standardized anomaly for air temperature will increase by 6 standard deviations from the historic means. Therefore, there is a possibility the maximum temperature thresholds for one or more life stages for shellfish may be exceeded by the end of the century, especially during episodic heat waves (Jones et al. 2009; Jones et al. 2010; Zippay and Helmuth 2012; Speights et al. 2017). Warming air and seawater can also increase the susceptibility of shellfish to disease, parasites and predation by local and invasive species (Smolowitz 2013; Burge et al. 2014).

Oyster performance peaks in salinities from 15 to 30 practical salinity units (psu), and they can withstand salinities of 0-40 psu. However, oysters tend to grow faster and be in better condition with less variation in salinity (Galtsoff 1964; Shumway 1996). Blue mussel is a euryhaline species, and can survive periodic fluctuations below 15 psu, although they do not thrive in low salinity conditions (Animal Diversity Web 2020). The ROMS-NWA climate projections for RCP8.5 indicate a decline in surface salinity for the U.S. continental shelf and the Gulf of Maine. Although the ROMS-NWA projections indicate salinities for estuarine waters in Long Island Sound, Delaware Bay, and Chesapeake Bay are projected to increase by at least 2 standard deviations from the historic means, it is unclear if shallow, nearshore marine waters may also increase in salinity.

Nearshore marine waters are generally more susceptible to acidification than oceanic waters because theyare subject to more acid sources, and are generally less buffered than oceanic waters (i.e., differences in the amount of dissolved inorganic carbon, dissolved and particulate organic carbon, total alkalinity and nutrients from riverine and estuarine sources) (Waldbusser et al. 2011; Ekstrom et al. 2015; Gledhill et al. 2015). Ocean acidification will likely most directly negatively impact shellfish reefs, with larger negative effects on survival for larvae than adults (Kroeker et al. 2013). Ocean acidification is expected to reduce the shell size, thickness, calcification rates and growth, and survival of embryo and larval bivalves (Gazeau et al. 2013). Ries et al. (2009) exposed both mussels and oysters to low pH levels, and found that acidification negatively impacts oyster calcification rates whereas there was no relationship between pH and the calcification rate of

blue mussels. Yet, increased water and air temperature in the northeastern U.S. is likely resulting in reduced physiological performance of mussels (Zippay and Helmuth 2012), potentially explaining why mussel beds are becoming less common in southern New England the western portions of the Gulf of Maine when temperatures have exceeded the thermal maxima for mussels. Dodd etal. (2015) exposed oysters and their predators to increased acidification and found that acidification negatively affected oyster growth, but also reduced crab consumption of oysters. The synergistic effects of temperature, salinity, and pH on metabolism in marine mollusks may be greater than reduced pH alone. Some studies have shown additive and synergistic negative effects on bivalves and gastropods from ocean acidification and low dissolved oxygen (Kroeker et al. 2013; Gobler et al. 2014; Clark and Gobler 2016; Gobler and Bauman 2016; Griffith and Gobler 2017).

Intertidal marine shellfish habitats may experience disturbance from storms, which are expected to increase in frequency and intensity in the Northeast region (USGCRP 2017). Coastal storms may increase physical/mechanical disturbance and stress to shellfish reef structure and associated organisms, and repeated disturbance may prohibit formation of robust three-dimensional complex structures.

Sea-level rise poses both direct and indirect threats to intertidal habitats. Oyster reefs generally have the capacity to grow vertically and landward to keep up with sea level rise. However, because they exist across a wide geographical and environmental range and diverse landscape settings, they are influenced by different types and magnitudes of stress which influence growth rates. Baillie and Grabowski (2018) reported lower recruitment and higher mortality of oysters at higher elevations in the intertidal zone, likely the result of desiccation and food limitation. Existing intertidal oyster reefs that have reached their growth ceiling could respond to sea level rise and the associated reduction in aerial exposure time by demonstrating enhanced vertical accretion. Enhanced accretion rates of intertidal oyster reefs have the potential to surpass all other coastal ecosystem engineers, including saltmarsh and seagrass (Rodriguez et al. 2014). Therefore, intertidal high-salinity areas may be hotspots for oyster-reef productivity (Rodriguezet al. 2014). However, many developed intertidal areas may not provide the physical space for oyster reefs to expand landward if these shorelines have been hardened (Rodriguez et al. 2014; Ridge et al. 2015).

<u>Habitat Summary</u>: Shellfish habitats have been highly altered, with estimates in the U.S. suggesting that 68% of historic oyster reef extent and over 80% of the productivity of these habitats have been lost primarily due to overharvesting and destructive harvesting practices, but also as a consequence of dredge and fill activities, disease, sedimentation, predators, and competition (zu Ermgassen et al. 2012).

Historically, overharvesting has been the largest threat to shellfish reefs, with 85% of oyster reefs lost worldwide (Beck et al. 2011), and similar impacts having occurred in the U.S. (zu Ermgassen et al. 2012). Kirby (2004) suggested that harvesting of oysters peaked and then the fisheries collapsed in the northeastern U.S. in coastal Massachusetts and Southern New England in the early 1800s. Efforts to conserve and rebuild shellfish reefs have been challenged by anthropogenic disturbances such as bottom water hypoxia, dredge and fill activity, shoreline hardening, diseases such as Dermo and MSX, sedimentation, predation, and competition (Rothschild et al. 1994; Kirby 2004; Beck et al. 2011; zu Ermgassen et al. 2012). In coastal New England, many shellfish reefs suffer from poor recruitment, motivating restoration practitioners to seed reefs with juvenile oysters set on dead oyster shells at oyster hatcheries and then transplanted to shallow reefs (Grabowski, personal observation). Unfortunately, many of the restored oyster reefs in coastal Rhode Island’s salt ponds have failed to increase natural recruitment (Grabowski and Hughes, unpublished data). Anecdotally, many of the intertidal mussel beds that were common in northeastern Massachusetts are no longer present (Grabowski, personal observation), possibly a consequence of local warming of sea and air temperatures (Zippay and Helmuth 2012).

Climate change will exacerbate other anthropogenic effects that have negatively impacted shellfish reefs. Oysters are thought to be among the most vulnerable of species to ocean acidification given its impacts on shell calcification rates and the protection that the shell provides from predators (Ries et al 2009). Although oyster reefs can grow relatively quickly and keep pace with sea-level rise, shoreline development in many regions may have removed available space for reefs to migrate landward. While mussels may be less vulnerable to acidification compared to oysters (Ries et al. 2009), they are being crowded out of coastal waters in New England likely as a consequence of air and sea water warming (Zippay and Helmuth 2012).

Warming coupled with eutrophication common in many coastal waters will likely amplify the conditions that result in bottom water hypoxia, further contributing to intertidal shellfish reef habitat loss. Further investigation of how warming and acidification are impacting the early life history of shellfish could help elucidate why recruitment failure is common in some areas of the northeastern U.S.

Literature Cited

Animal Diversity Web. 2020. [Internet]. *Mytilus edulis*. (Accessed 31 August 2020). https://animaldiversity.org/accounts/Mytilus_edulis/.

Bahr LM, Lanier WP. 1981. The ecology of intertidal oyster reefs of the South Atlantic Coast: a community profile. U.S. Fish and Wildlife Service. FWS/OBS-81/15.

Baillie CB, Grabowski JH. 2018. Factors affecting recruitment, growth and survival of the eastern oyster *Crassostrea virginica* across an intertidal elevation gradient in southern New England. Marine Ecology Progress Series 609:119–32. https://doi.org/10.3354/meps12830

Beck MW, Brumbaugh RD, Airoldi L, Carranza A, Coen LD, Crawford C, Defeo O, Edgar G, HancockB, Kay M, Lenihan H, Luckenbach M, Toropova C, Zhang G. 2011. Oyster reefs at risk and recommendations for conservation, restoration and management. Bioscience 61:107-16.

Burge CA, Eakin CM, Friedman CS, Froelich B, Hershberger PK, Hofmann EE, Petes LE, Prager KC,Weil E, Willis BL, Ford SE, Harvell CD. 2014. Climate change influences on marine infectious diseases: implications for management and society. Annual Review of Marine Science 6:249-77.

Clark H, Gobler CJ. 2016. Diurnal fluctuations in CO_2 and dissolved oxygen concentrations do not provide a refuge from hypoxia and acidification for early-life-stage bivalves. Marine Ecology Progress Series 558:43114. https://doi.org/10.3354/meps11852.

Coen LD, Luckenbach MW, Breitburg DL. 1999. The role of oyster reefs as essential fish habitat: a review of current knowledge and some new perspectives. American Fisheries Society Symposium22:438-54.

Dame RF, Zingmark RG, Haskins E. 1984. Oyster reefs as processors of estuarine materials. Journal of Experimental Marine Biology and Ecology 83:239-47.

Dodd LF, Grabowski JH, Piehler MF, Westfield I, Ries JB. 2015. Ocean acidification impairs crab foraging behaviour. Proceedings of the Royal Society B: Biological Sciences 282.

Ekstrom JA, Suatoni L, Cooley SR, Pendleton LH, Waldbusser GG, Cinner JE, Ritter J, Langdon C, van Hooidonk R, Gledhill D, Wellman K, Beck MW, Brander LM, Rittschof D, Doherty C, Edwards PET, Portela R. 2015. Vulnerability and adaptation of US shellfisheries to ocean acidification. Nature Climate Change 5(3):207-14.

Galtsoff PS. 1964. The American oyster *Crassostrea virginica* (Gmelin). U.S. Fish Wildlife Service Fisheries Bulletin 64:1-480.

Gazeau F, Parker LM, Comeau S, Gattuso J-P, O'Connor WA, Martin S, Pörtner H-O, Ross PM. 2013. Impacts of ocean acidification on marine shelled molluscs. Marine Biology 160(8):2207-45.

Gledhill D, White M, Salisbury J, Thomas H, Misna I, Liebman M, Mook B, Grear J, Candelmo A, Chambers RC, Gobler C, Hunt C, King A, Price N, Signorini S, Stancioff E, Stymiest C, Wahle R, Waller J, Rebuck N, Wang Z, Capson T, Morrison JR, Cooley S, Doney S. 2015. Ocean and coastal acidification off New England and Nova Scotia. Oceanography 25(2):182-97.

Gobler CJ, Baumann H. 2016. Hypoxia and acidification in ocean ecosystems: coupled dynamics and effects

on marine life. Biology Letters 12(5):20150976.

Gobler CJ, DePasquale EL, Griffith AW, Baumann H. 2014. Hypoxia and acidification have additive and synergistic negative effects on the growth, survival, and metamorphosis of early life stage bivalves. PLoS One 9(1):e83648.

Griffith AW, Gobler CJ. 2017. Transgenerational exposure of North Atlantic bivalves to ocean acidification renders offspring more vulnerable to low pH and additional stressors. Scientific Reports7:11394.

Gunter G. 1951. The West Indian tree on the Louisiana coast, and notes on growth of the Gulf Coast oysters. Science 113:16-517.

Jones SJ, Lima FP, Wethey DS. 2010. Rising environmental temperatures and biogeography: poleward range contraction of the blue mussel, *Mytilus edulis* L., in the western Atlantic. Journal of Biogeography 37(12):2243-59.

Jones SJ, Mieszkowska N, Wethey DS. 2009. Linking thermal tolerances and biogeography: *Mytilus edulis* (L.) at its southern limit on the east coast of the United States. Biological Bulletin 217(1):73-85.

Kirby MX. 2004. Fishing down the coast: Historical expansion and collapse of oyster fisheries along continental margins. Proceedings of the National Academy of Science of the United States of America 101:13096-99.

Kroeker KJ, Kordas RL, Crim R, Hendriks IE, Ramajo L, Singh GS, Duarte CM, Gattuso J-P. 2013. Impacts of ocean acidification on marine organisms: quantifying sensitivities and interaction with warming. Global Change Biology 19(6):1884-96.

Lenihan HS. 1999. Physical-biological coupling on oyster reefs: how habitat structure influences individual performance. Ecological Monographs 69:251-75.

Lesser MP. 2016. Climate change stressors cause metabolic depressions in the blue mussel, *Mytilus edulis*, from the Gulf of Maine. Limnology and Oceanography 61:1705-17.

Newell RIE. 1988. Ecological changes in Chesapeake Bay: are they the result of overharvesting the American oyster, *Crassostrea virginica*? In: Lynch MP, Krome EC (editors). Understanding the estuary: advances in Chesapeake Bay research. Chesapeake Research Consortium, Baltimore, Maryland, USA.

Newell RIE, Cornwell JC, Owens MS. 2002. Influence of simulated bivalve biodeposition and microphyrobenthos on sediment nitrogen dynamics: a laboratory study. Limnology and Oceanography 47:1367-79.

Paerl HW, Pinckney JL, Fear JM, Peierls BL. 1998. Ecosystem responses to internal and watershed organic matter loading: consequences for hypoxia in the eutrophying Neuse river estuary, North Carolina, USA. Marine Ecology Progress Series 166:17-25.

Peterson CH, Grabowski JH, Powers SP. 2003. Estimated enhancement of fish production resulting from restoring oyster reef habitat: quantitative valuation. Marine Ecology Progress Series 264:249-64.

Piehler MF, Smyth AR. 2011. Impacts of ecosystem engineers on estuarine nitrogen cycling. Ecosphere 2:art12.

Ridge JT, Rodriguez AB, Fodrie FJ, Lindquist NL, Brodeur MC, Coleman SE, Grabowski JH, Theuerkauf EJ. 2015. Maximizing oyster-reef growth supports green infrastructure with accelerating sea-level rise. Scientific Reports 5: 14785.

Ries JB, Cohen AL, McCorkle DC. 2009. Marine calcifiers exhibit mixed responses to CO_2-induced ocean acidification. Geology 37:1131-4.

Rodriguez AB, Fodrie FJ, Ridge JT, Lindquist NL, Theuerkauf EJ, Coleman SE, Grabowski JH, Brodeur MC, Gittman RK, Keller DA, Kenworthy MD. 2014. Oyster reefs can outpace sea-level rise. Nature Climate Change 4:493-7.

Rothschild BJ, Ault JS, Goulletquer P, Heral M. 1994. Decline of the Chesapeake Bay oyster population: a century of habitat destruction and overfishing. Marine Ecology Progress Series 111:29-39.

Shumway CA. 1999. A neglected science: applying behavior to aquatic conservation. Environmental Biology of Fishes 55:183-201.

Smolowitz R. 2013. A review of current state of knowledge concerning *Perkinsus marinus* effects on *Crassostrea virginica* (Gmelin) (the Eastern oyster). Veterinary Pathology 50(3):404-11.

Speights C J, Silliman BR, McCoy MW. 2017. The effects of elevated temperature and dissolved ρCO_2 on a marine foundation species. Ecology and evolution 7(11):3808–14. https://doi.org/10.1002/ece3.2969

Ulanowicz RE, Tuttle JH. 1992. The trophic consequences of oyster stock rehabilitation in Chesapeake Bay. Estuaries 15:298-306.

U.S. Global Change Research Program (USGCRP). 2017. Climate Science Special Report: Fourth National Climate Assessment. Volume I. [Wuebbles, D.J., D.W. Fahey, K.A. Hibbard, D.J. Dokken, B.C. Stewart, and T.K. Maycock (eds.)]. U.S. Global Change Research Program. Washington, DC. USA. 470 pp. doi: 10.7930/J0J964J6.

Waldbusser GG, Voigt EP, Bergschneider H, Green MA, Newell RIE. 2011. Biocalcification in the eastern oyster (Crassostrea virginica) in relation to long-term trends in Chesapeake Bay pH. Estuaries and Coasts 34(2):221-31.

Wells HW. 1961. The fauna of oyster beds, with special reference to the salinity factor. Ecological Monographs 31:239-66.

Zippay ML, Helmuth B. 2012. Effects of temperature change on mussel, Mytilus. Integrative Zoology 7:312-27.

zu Ermgassen PSE, Grabowski JH, Gair JR, Powers SP. 2016. Quantifying fish and mobile invertebrate production from a threatened nursery habitat. Journal of Applied Ecology 53:596-606.

zu Ermgassen PSE, Spalding MD, Blake B, Coen LD, Dumbauld B, Geiger S, Grabowski JH, Grizzle R, Luckenbach M, McGraw K, Rodney W, Ruesink JL, Powers SP, Brumbaugh RD. 2012. Historical ecology with real numbers: past and present extent and biomass of an imperilled estuarine habitat. Proceedings of the Royal Society B: Biological Sciences 279:3393-400.

Marine Intertidal Rocky Bottom

System: Marine

Subsystem: Intertidal

Class: Rocky Bottom

Sub-class: Bedrock, Rubble, Cobble, Gravel

Geographic Area: Entire Area

Overall Vulnerability Rank = High

Habitat Sensitivity = Moderate

Climate Exposure = Very High

	Marine Intertidal Rocky Bottom	Attribute Mean	Data Quality	Distribution of Expert Scores
Sensitivity Attributes	Habitat condition	2.5	2.4	
	Habitat fragmentation	2.2	2.2	
	Distribution/Range	3.2	2.4	
	Mobility/Ability to spread or disperse	2.2	2.2	
	Resistance	2	2.4	
	Resilience	2	2.2	
	Sensitivity to changes in abiotic factors	1.8	1.8	
	Sensitivity and intensity of non−climate stressors	2.8	2.2	
	Dependency on critical ecological linkages	1.9	1.6	
	Sensitivity Component Score	Moderate		
Exposure Factors	Sea surface temp	n/a	n/a	
	Bottom temp	n/a	n/a	
	Air temp	4	3	
	River temp	n/a	n/a	
	Surface salinity	1.3	2.7	
	Bottom salinity	n/a	n/a	
	pH	4	2	
	Sea level rise	3.9	2.2	
	Precipitation	2.4	2.1	
	Floods	n/a	n/a	
	Droughts	n/a	n/a	
	Exposure Component Score	Very High		
**Overall Vulnerability Rank**		**High**		

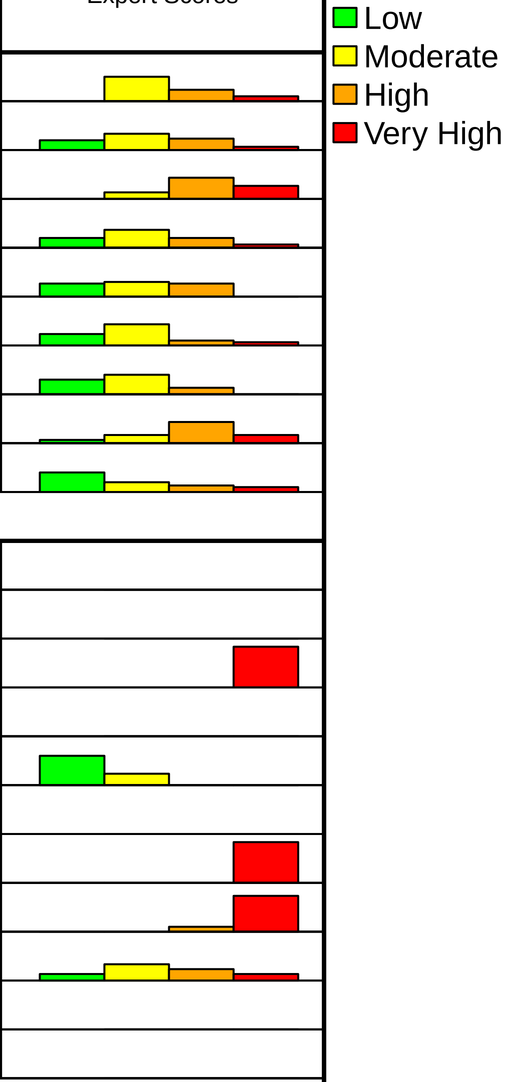

Habitat Name: Marine Intertidal Rocky Bottom

System**:** Marine

Subsystem: Intertidal

Class: Rocky Bottom

Sub-class: Bedrock, Rubble, Cobble, Gravel

Geographic Area: Entire Area

Habitat Description: This sub-class includes marine intertidal rocky bottom bedrock, rubble, cobble/gravel. Rocky bottom habitat established on surfaces and crevices of relatively immobile rocky surfaces, including loose rocks of various sizes (rubble, cobble/gravel) and exposed bedrock. In addition, this habitat subclass includes the epibenthic flora and fauna associated with hard bottoms, including calcareous algae. This subclass does not include non-calcareous algae, which are included in the marine aquatic bed habitat narrative.

Natural rocky habitats occur across a wide latitudinal range in New England, but are much less common in the Mid-Atlantic. The extent of intertidal rocky habitats in the Gulf of Maine is limited by the tidal range and by shoreline gradient. New England, particularly the Gulf of Maine, has a larger tidal range and steeper shorelines than the Mid-Atlantic. Natural rocky habitats range from granule/pebble (or gravel) to cobbles, boulders, and ledge/bedrock. Biota associated with all these habitat types also varies. Natural rocky habitats in New England are partially continuous with multiple, moderately-sized patches. Rocky shorelines and bottom habitats are usually in close proximity to each other.

Overall Climate Vulnerability Rank: High (81% certainty from bootstrap analysis). Bootstrap analysis found a 20% probability that the overall vulnerability rank is Very High.

Climate Exposure: Very High. The overall Very High component score was influenced by three Very High climate exposure means: Air Temperature (4.0), Sea Level Rise (SLR) (3.9), pH (4.0), and to a lesser degree Precipitation (2.4). The exposure attribute mean for Surface Salinity was 1.3. pH is projected to drastically decrease from historic levels for the entire study area by the end-of-century (increasing ocean acidification), and projected changes in air temperature and SLR are also expected to be very high throughout the range. The intertidal nature of this habitat places it at the nexus of significant atmospheric and oceanic change.

Habitat Sensitivity: Moderate. Three of the nine sensitivity attribute means were ≥2.5, with Distribution/Range being the highest (3.2). The remaining six were between 1.8 and 2.2. The higher sensitivity for Distribution/Range likely reflects the skewed distribution of rocky bottom habitats in the Gulf of Maine and southern New England compared to the Mid-Atlantic. The Sensitivity and Intensity of Non−Climate Stressors and Habitat Condition attribute means were 2.5 and 2.8. Most of the sensitivity attributes were spread across all of the scoring bins, which likely reflects the variability of sensitivity in the abiotic (rocky bottom) and biotic (flora and fauna) habitat components associated with this subclass.

Data Quality & Gaps: The data quality scores for Air Temperature and Surface Salinity were high (3.0 and 2.7, respectively), while the others were relatively low for pH, SLR, and Precipitation (≤2.2). Data quality is lower in general for intertidal habitats due to the low resolution of CMIP5 and ROMS-NWA projections for climate exposure in nearshore, shallow coastal areas.

For habitat sensitivity, data quality for seven of the nine attributes were deemed moderate (≥2.2), and none were >2.4. The two attributes with the lowest data quality scores were Dependency on Critical Ecological Linkages and Sensitivity to Changes in Abiotic Factors (1.6 and 1.8, respectively).

Positive or Negative Climate Effect for the Northeast U.S.: The effect of climate change on marine intertidal rocky bottom in the Northeast U.S. is expected to be mostly negative (95% of the experts' scores were negative and 5% neutral).

Climate Effects on Habitat Condition and Distribution: In general, intertidal rocky substrates are not themselves sensitive to climate change, but associated organisms are, and responses vary between species. However, intertidal organisms are generally more adapted to extreme conditions (e.g., temperature, salinity) than those in the subtidal zone. Mollusk species associated with intertidal rocky habitats are sensitive to low pH, with larger negative effects on survival for larvae than adults (Kroeker et al. 2013).

Ocean acidification is expected to reduce the shell size, thickness, calcification rates and growth, and survival of embryo and larval bivalves and gastropods (Gazeau et al. 2013). Kroeker et al. (2013) reported echinoderms will experience negative effects on their growth and survival, with the largest effects on growth for larval stages.

Sea level rise will submerge intertidal rocky bottom habitats, and may lead to coastal erosion. The rate of erosion is influenced by the rate of SLR, but is also affected by the intensity and frequency of, and exposure to, storms, tectonic events, and other factors (Nicholls et al. 2007). Cliffed coasts are also vulnerable to coastal erosion and SLR, although hard rock cliffs are more resistant to erosion than softer lithologies (Nicholls et al. 2007; Ashton et al. 2011).

Intertidal rocky bottom substrates generally have limited mobility, and natural sediment transport may be further limited by hardened shoreline structures. However, organisms associated with rocky habitats have a high capability to spread (e.g., larval dispersal) and establish, as long as hard substrate is present. There is an abundance of rocky substrate in New England. In the Mid-Atlantic, where natural rocky substrates are less common, organisms that attach to hard substrates are more restricted to artificial hard substrates. However, there is some evidence that rocky habitat epifauna do not settle, grow, and survive assuccessfully on all types of artificial substrates (e.g., seawalls, pilings) (Gittman et al. 2016). Intertidal rocky habitats (and their associated flora and fauna) may also experience some disturbance from storms, which are expected to increase in frequency and intensity in the Northeast region (USGCRP 2017).

Organisms associated with marine intertidal rocky habitats are adapted to highly variable conditions, such as extreme temperatures (and desiccation), changing salinity, and high wave energy. However, the fauna may experience impacts as temperatures become more extreme and exceed tolerance thresholds. Intertidal biota are also sensitive physical removal, damage, and predation.

Habitat Summary: Intertidal rocky bottom habitat ranges from pristine to moderately degraded (EPA 2012), and is more exposed to coastal development, freshwater runoff and other pollution, and natural disturbances like extreme weather than subtidal habitats. Natural rocky habitats in the intertidal zone are under threat from growing population density and coastal development, which are expected to cause increased habitat loss in the future (Sorte et al. 2017). Shoreline hardening with non-native, engineered stone can replace native, natural rocky bottom shoreline and result in the mortality of flora and fauna.

Although biodiversity and abundance on engineered riprap and breakwaters are not vastly different from natural shorelines, the effects are highly heterogeneous across habitat type and species and the results are subject to some uncertainty (Gittman et al. 2016). Increased shoreline hardening in heavily populated and industrialized areas is expected as climate change effects become more severe and widespread. Titus et al. (2009) estimated that almost 60% of land within 1 m above the high tide line on the U.S. Atlantic coast (Florida to Massachusetts) is developed or expected to be developed.

Like most marine species, intertidal flora and fauna are sensitive to pollution (e.g., oil spills, heavy metals), and exposure to intertidal habitat may be more common than subtidal habitats due to the proximity to impervious surfaces and development. Southern New England and Mid-Atlantic coastal areas have high contaminant loads due to legacy pollution from industrialization, and continued urbanization (EPA 2012). Rocky habitats and coarse-grained sediments such as sand generally contain less total organic carbon levels,

and hence lower concentrations of contaminants compared to soft-grained sediments (ICES 1992; Pearce 1994). However, benthic infauna can be sensitive to contaminants and have toxic effects to many compounds. Fauna and flora can also be highly sensitive to eutrophication from increased nutrient runoff.

Bedrock, boulders, and large cobble are highly resistant to disturbance, to a greater degree than smaller substrates like gravel. When physically disturbed (e.g., ice scour, storms), the function of these habitats is not impaired, though they may be temporarily buried by mobile sand. Some attached species recover quickly, while others may take years (Bertness et al. 2002). In the presence of both native (e.g., littorinid snails) and invasive (e.g., green crabs) predators, recovery of fauna and flora in the intertidal zone can take years (Bertness et al. 2002), and is dependent on species-specific methods of reproduction, patch size, and season of disturbance (Kim & DeWreede 1996). Recovery of longer-lived species (e.g., sponges, anemones, tunicates, molluscs), although mobile species generally re-occupy rocky habitats relatively quickly after disturbance (Johnson et al. 2008).

Intertidal rocky infauna and epifauna are sensitive to invasive species, including the green crab (*Carcinus maenas*) and the colonial ascidian, *Botrylloides violaceus*. Green crabs have established populations in New England and northern Mid-Atlantic in both subtidal and intertidal zones. It is a predator of many forms of marine life, including worms and mollusks (GISD 2020). In some areas (particularly New England), the crab's voracious appetite has affected the commercial shellfish industry (Webber 2013; Beal 2014).

Species that graze on epiflora and epifauna (e.g., sea urchins) control the abundances of species associated with rocky bottom habitats. For example, in some areas of the Gulf of Maine, urchin populations have exploded and decimated kelp beds (i.e., "urchin barrens"), which has been attributed to overfishing of the top predators (Steneck et al. 2002). Likewise, the collapse in the populations of sea urchins can cause proliferation of macroalgae species that reduces species diversity of rocky habitats, and can interfere with settlement of benthic and demersal larval life stages.

Literature Cited

Ashton AD, Walkden MJA, Dickson ME. 2011. Equilibrium responses of cliffed coasts to changes in the rate of sea level rise. Marine Geology 284(1):217-29.

Beal BF. 2014. Final report: Green crab, *Carcinus maenas*, trapping studies in the Harraseeket River and manipulative field trials to determine effects of green crabs on the fate and growth of wild and cultured individuals of soft-shell clams, *Mya arenaria* (May to November 2013). Downeast Institutefor Applied Marine Research and Education, Beals, ME. 76 pp. (Accessed 30 Oct. 2020). https://downeastinstitute.org/wp-content/uploads/2018/08/1_24-final-report-freeport-shellfish-restoration-project-b.-beal.pdf.

Bertness MD, G.C. Trussell GC, Ewanchuk PJ, Silliman B.R. 2002. Do alternate stable community states exist in the Gulf of Maine rocky intertidal zone? Ecology 83(12):3434-48.

Gazeau F, Parker LM, Comeau S, Gattuso J-P, O'Connor WA, Martin S, Pörtner H-O, Ross PM. 2013. Impacts of ocean acidification on marine shelled molluscs. Marine Biology 160(8):2207-45.

Gittman RK, Scyphers SB, Smith CS, Neylan IP, Grabowski JH. 2016. Ecological consequences of shoreline hardening: a meta-analysis. Bioscience 66(9):763-73.

Global Invasive Species Database (GISD). 2020. [Internet]. Species profile: *Carcinus maenas*. (Accessed 3 Aug. 2020). http://www.iucngisd.org/gisd/species.php?sc=114

International Council for the Exploration of the Sea (ICES). 1992. Report of the ICES working group on the

effects of extraction of marine sediments on fisheries. Copenhagen (Denmark): ICES Cooperative Research Report # 182.

Johnson MR, Boelke C, Chiarella LA, Colosi PD, Greene K, Lellis-Dibble K, Ludemann H, Ludwig M, McDermott S, Ortiz J, Rusanowsky D, Scott M, Smith J. 2008. Impacts to marine fisheries habitat from nonfishing activities in the northeastern United States. NOAA Technical Memorandum. National Oceanic and Atmospheric Administration, National Marine Fisheries Service.NMFS-NE-2O9. 1-328.

Kim JH, DeWreede R.E. 1996. Effects of size and season of disturbance on algal patch recovery in arocky intertidal community. Marine Ecology Progress Series 133:217-28.

Kroeker KJ, Kordas RL, Crim R, Hendriks IE, Ramajo L, Singh GS, Duarte CM, Gattuso J-P. 2013. Impacts of ocean acidification on marine organisms: quantifying sensitivities and interaction with warming. Global Change Biology 19(6):1884-96.

Nicholls RJ, Wong PP, Burkett V, Codignotto J, Hay J, McLean R, Ragoonaden S, Woodroffe CD. 2007. Coastal systems and low-lying areas. Climate change 2007: impacts, adaptation and vulnerability. Contribution of Working Group II to the Fourth Assessment Report of the Intergovernmental Panel on Climate Change. [Parry ML, Canziani OF, Palutikof JP, van der Linden PJ, Hanson CE (eds)]. Intergovernmental Panel on Climate Change. 315-56.

Pearce JB. 1994. Mining of seabed aggregates. *In*: Langton RW, Pearce JB, Gibson JA, editors. Selected living resources, habitat conditions, and human perturbations of the Gulf of Maine: environmental and ecological considerations for fishery management. Woods Hole, MA. NOAA Technical Memorandum. NMFS-NE-106. 48-50.

Sorte CJB, Davidson VE, Franklin MC, Benes KM, Doellman MM, Etters RJ, Hannigan RE, Lubchenco J, Menge BA. 2017. Long-term declines in an intertidal foundation species parallel shifts in community composition. Global Change Biology 23(1):341-52.

Steneck R, Graham MH, Bourque BJ, Corbett D, Erlandson JM, Estes JA, Tegner MJ. 2002. Kelp forest ecosystems: biodiversity, stability, resilience and future. Marine Sciences Faculty Scholarship. 65. https://digitalcommons.library.umaine.edu/sms_facpub/65

Titus JG, Hudgens DE, Trescott DL, Craghan M, Nuckols WH, Hershner CH, Kassakian JM, Linn CJ, Merritt PG, McCue TM, O'Connell JF, Tanski J, Wang J. 2009. State and local governments plan for development of most land vulnerable to rising sea level along the US Atlantic coast. Environmental Research Letters 4(4):1-7.

U.S. Environmental Protection Agency (EPA). 2012. National Coastal Condition Report IV.EPA-R-842-10-003. US Environmental Protection Agency, Washington D.C. https://www.epa.gov/sites/production/files/2014-10/documents/0_nccr_4_report_508_bookmarks.pdf.

U.S. Global Change Research Program (USGCRP). 2017. Climate Science Special Report: Fourth National Climate Assessment. Volume I [Wuebbles, D.J., D.W. Fahey, K.A. Hibbard, D.J. Dokken, B.C. Stewart, and T.K. Maycock (eds.)]. U.S. Global Change Research Program. Washington, DC.USA. 470 pp. doi: 10.7930/J0J964J6.

Webber MM. 2013. Results of the one-day green crab trapping survey conducted along the Maine coast from August 27 to 28, 2013. Maine Department of Marine Resources, Augusta, ME. 6 pp. (Accessed 30 October 2020). https://www.maine.gov/dmr/science-research/species/invasives/greencrabs/documents/trapsurveyrpt2 013.pdf.

Marine Intertidal Mud Bottom

System: Marine

Subsystem: Intertidal

Class: Unconsolidated Bottom

Sub-class: Mud

Geographic Area: Entire Area

Overall Vulnerability Rank = High

Habitat Sensitivity = Moderate

Climate Exposure = Very High

	Marine intertidal mud	Attribute Mean	Data Quality	Distribution of Expert Scores
Sensitivity Attributes	Habitat condition	2.7	2.2	
	Habitat fragmentation	2.4	2.4	
	Distribution/Range	2.2	2.4	
	Mobility/Ability to spread or disperse	1.9	2.2	
	Resistance	2.8	2.2	
	Resilience	2	2.2	
	Sensitivity to changes in abiotic factors	1.8	2	
	Sensitivity and intensity of non−climate stressors	3.2	2.2	
	Dependency on critical ecological linkages	1.6	1.4	
	Sensitivity Component Score	Moderate		
Exposure Factors	Sea surface temp	n/a	n/a	
	Bottom temp	n/a	n/a	
	Air temp	4	3	
	River temp	n/a	n/a	
	Surface salinity	1.2	2.7	
	Bottom salinity	n/a	n/a	
	pH	4	2	
	Sea level rise	3.9	2.2	
	Precipitation	2.4	2.1	
	Floods	n/a	n/a	
	Droughts	n/a	n/a	
	Exposure Component Score	Very High		
**Overall Vulnerability Rank**		**High**		

Low
Moderate
High
Very High

Habitat Name: Marine Intertidal Mud Bottom

System**:** Marine

Subsystem: Intertidal

Class: Unconsolidated Bottom

Sub-class: Mud

Geographic Area: Entire Area

Habitat Description: This habitat sub-class includes marine intertidal mud from mean high to the mean low water lines, where salinity is >30 ppt. This habitat also include the epifauna and infauna associated with unconsolidated mud bottom, including non-reef-forming mollusks (e.g., soft-shell clams, hard clams, sea scallops, surf clams, ocean quahogs), marine worms, small crustaceans, gastropods, and polychaetes. This subclass excludes specific habitats identified elsewhere (i.e., non-calcareous algal bed, rooted vascular beds, and reef-forming mollusks, such as blue mussels, eastern oysters).

Overall Climate Vulnerability Rank: High (80% certainty from bootstrap analysis). Although themajority of the bootstrap results match the results of the categorical vulnerability rank, 20% of the bootstrap results were in the Very High vulnerability rank.

Climate Exposure: Very High. The overall very high exposure score was influenced by three Very High attribute means: Air Temperature (4), pH (4), and Sea Level Rise (SLR) (3.9). Marine intertidal mud scored Moderate for Precipitation (2.4). The intertidal nature of this habitat places it at the nexus of significant atmospheric change in the form of increases in extreme precipitation events and air temperature, and oceanic change such as decreasing pH and rising sea level.

Habitat Sensitivity: Moderate. Only one of the nine sensitivity attributes means were >3.0 (Sensitivity and intensity of non-climate stressors, 3.2). The next highest scores were Resistance (2.8) and Habitat Condition (2.7). The mean scores are generally indicative of the resilient nature of mud habitats and an acknowledgement that the threat to intertidal habitats may be more related to development and proximity to human activity than climate change.

Data Quality & Gaps: The data quality scores for three of the five climate exposure factors (pH, SLR and Precipitation) were scored relatively low (<2.2). The other exposure factors, Surface Salinity and Air Temperature, were scored 2.7 and 3.0, respectively. Data quality is lower for intertidal habitats due to the low resolution of CMIP5 and ROMS-NWA projections for climate exposure in nearshore, shallow coastal areas.

For habitat sensitivity, two of the nine attributes had data quality scores of 2.4 (Habitat Fragmentation and Distribution/Range). The remaining seven attributes had relatively low (<2.2) data quality scores. These data quality scores likely reflect the difficulty in resolving dynamics and sensitivity to change in benthic habitats at the nexus of oceanic and terrestrial systems.

Positive or Negative Climate Effect in the Northeast U.S. Shelf: The effect of climate change on marine intertidal mud habitats on the Northeast U.S. Shelf is expected to be overwhelmingly negative (95% of the experts' scores were negative and 5% were neutral). The climate directionality scores were somewhat higher than the overall climate vulnerability rank. However, it should be noted that the higherclimate directionality effect for marine intertidal mud habitats compared to estuarine intertidal mud habitats, and the relatively high degree of uncertainty in climate sensitivity attributes, may stem from the higher dynamic environmental conditions in estuarine intertidal mud habitats which significantly increases the difficulty in assessing impact and parsing drivers of change.

Climate Effects on Habitat Condition and Distribution: Although the climate exposure of intertidal mud habitats is expected to be very high, the sensitivity of these habitats to climate change is less well understood. The Moderate sensitivity score for marine intertidal mud habitats likely reflects the general resilient nature of

mud substrates and associated infauna and epifauna. However, sea level rise is known to create a cascade of habitat changes, including potential conversion of intertidal mud to subtidal mud habitat. Predicting the ultimate result of this change on the amount, range and distribution, and connectivity of intertidal habitats will depend on the rate and magnitude of sea level rise and these habitat conversions. Additionally, while subtidal mud habitats may expand as sea level submerges intertidal mud habitats (Vos and van Kesteren 2000), the nature of where intertidal habitats expand may be dependent onthe rate of coastal development which is not anticipated to decline in the future. For example, shoreline development and hardening with riprap and seawalls are pervasive in most urban coastal areas (Johnson et al. 2008). One of the biggest threats to intertidal mud habitats is coastal squeeze, where beaches are trapped between rising sea levels and erosion, and human development on the other. Titus et al. (2009) estimated that almost 60% of land within 1 m above the high tide line on the U.S. Atlantic coast (Florida to Massachusetts) is developed or expected to be developed.

It is well known that acidification can impact bivalve larval development and growth by multiple modes of action (Gazeau et al. 2013; Waldbusser et al. 2015). In general, the larval life stage is more sensitive than juveniles and adults to ocean acidification (Kroeker et al. 2013). Ocean acidification is expected to reduce the shell size, thickness, calcification rates and growth, and survival of embryo and larval bivalves and gastropods (Gazeau et al. 2013). Some studies have shown additive and synergistic negative effects from ocean acidification and low dissolved oxygen on the growth, survival, and metamorphosis of larval bay scallop and hard clams (Kroeker et al. 2013; Griffith and Gobler 2017). Nearshore coastal waters are generally more susceptible to acidification than oceanic waters because they are subject to more acid sources and are generally less buffered than oceanic waters (i.e., differences in the amount of dissolved inorganic carbon, dissolved and particulate organic carbon, total alkalinity and nutrients from riverine and estuarine sources) (Ekstrom et al. 2015; Gledhill et al. 2015).

However, the impact of acidification in the benthos is much more difficult to detect *in situ*. For example, climate induced expansion of green crabs (*Carcinus maenas*) into more mudflats in the Northeast U.S. has significantly increased the predation pressure on settling soft shell clams (McClenachan et al. 2015), making the detection of acidification effects on soft shell clams difficult. Generally, ecological dependencies mediated by climate (e.g., invasive species and climate-migrations) will make direct correlations between biogeochemical dynamics, such as hypoxia and acidification, and important fisheries and protected species difficult to examine. Finally, in addition to climate-linked changes in species assemblages on these habitats, some climate related changes in biogeochemistry are also linked. For example, increased frequency of extreme precipitation events will change surface salinity in downstream ecosystems but will also decrease pH (Salisbury et al. 2008). However, due to the importance of shell-forming organisms in these areas (e.g., soft shell clams), a better understanding of pH dynamics maybe crucial for understanding the impact of climate on these habitats. The impacts of coastal acidification on these habitats will require significantly more research.

Infauna associated with intertidal mud habitats may be less sensitive than subtidal species to changes in air and water temperature because they are adapted to variability and extremes. Some mollusks appear to be affected by warming waters, and to a lesser degree water quality. Surf clam stocks in the Mid-Atlantic region have declined dramatically over the last 10 years, especially in New Jersey waters, and ocean quahog landings have shifted further north along the Atlantic coastline with substantial landings from southern New England waters (Lewis et al. 2001; MAFMC 2019). Increasing water temperatures, high nutrient levels, and algal blooms can trigger reductions in dissolved oxygen in the nearshore and intertidal water column and in sediments, which can have detrimental effects to infaunal and epifaunal species associated with mud habitats (Sharp et al. 1982; Brownlee et al. 2005).

Habitat Summary: Marine intertidal mud habitats are exposed to natural stressors including coastal storms, sediment transport, and tides, as well as a variety of anthropogenic impacts from recreation, marine transportation, development/hardening, dredging/filling, beach nourishment, stormwater discharge, and pollution (Johnson et al. 2008). Coastal population density and agriculture are associated with higher

eutrophication and contamination, which can disturb benthic habitat quality. According to the 2012 EPA Coastal Condition Report, sediment quality varies throughout the region with the poorest sediment quality in urbanized areas (EPA 2012). Low sediment quality ratings were primarily driven by sediment contamination, which are mostly due to elevated levels of metals, polychlorinated biphenyls, and pesticides. Fine-grained mud substrates generally contain higher total organic carbon (TOC) levels compared to coarse-grained, sandy sediments. Because contaminants such as polyaromatic hydrocarbons (PAH), pesticides, and polychlorinated biphenyl are sequestered in the TOC fraction of sediments, mud sediments tend to be more impacted by contaminants than sand substrates (ICES 1992; Pearce 1994). Benthic infauna are sensitive to contaminants and have toxic effects to many, including PAH compounds. PAH can persist in sediments for decades after the initial contamination, causing disruption of physiological and metabolic processes of benthic organisms (Vandermeulen and Mossman 1996).

Dredging, shoreline stabilization (e.g., riprap revetment, bulkheads, jetties, groins), and beach nourishment can alter the depth and sediment characteristics, with subsequent changes in infauna and epifauna/epiflora. All hardened shorelines have the potential to erode shallow water subtidal mud bottom. Hardened shorelines have been shown to have lower abundance, biomass, and diversity of benthic prey and predators (Seitz et al. 2006; Morley et al. 2012) and can have higher incidence of marine exotic/invasive species compared to native material (Tyrrell and Byers 2007).

Intertidal infauna and epifauna are sensitive to invasive species, including the green crab, which has established populations in New England and northern Mid-Atlantic in both subtidal and intertidal zones. It is a predator of many forms of marine life, including worms and mollusks (GISD 2020). In some areas (particularly New England), the crab's voracious appetite has affected the commercial shellfish industry (Webber 2013; Beal 2014).

Finally, in contrast with subtidal mud, intertidal mud habitats may be more susceptible to habitat fragmentation and changes in its range and distribution. In some locations, sea level rise may create new intertidal mud habitats while losing them in other locations. In highly fragmented intertidal habitats, recruitment of infauna can be compromised and significant reproductive effort can be lost into subtidal habitats that settling larvae are not adapted to thrive in. Future research in this area will benefit from detailed topography of intertidal and marsh areas that account for source and sink habitats under a range of sea level rise scenarios.

Literature Cited

Beal BF. 2014. [Internet]. Final report: Green crab, *Carcinus maenas*, trapping studies in the Harraseeket River andmanipulative field trials to determine effects of green crabs on the fate and growth of wild and cultured individuals of soft-shell clams, *Mya arenaria* (May to November 2013). Downeast Institute for Applied Marine Research and Education, Beals, ME. 76 pp. Accessed 30 Oct. 2020. https://downeastinstitute.org/wp-content/uploads/2018/08/1_24-final-report-freeport-shellfish-restoration-project-b.-beal.pdf.

Brownlee EF, Sellner SG, Sellner K.G. 2005. *Prorocentrum minimum* blooms: potential impacts on dissolved oxygen and Chesapeake Bay oyster settlement and growth. Harmful Algae 4 (3):593-602. https://doi.org/10.1016/j.hal.2004.08.009

Ekstrom JA, Suatoni L, Cooley SR, Pendleton LH, Waldbusser GG, Cinner JE, Ritter J, Langdon C, van Hooidonk R, Gledhill D, Wellman K, Beck MW, Brander LM, Rittschof D, Doherty C, Edwards PET, Portela R. 2015. Vulnerability and adaptation of US shellfisheries to ocean acidification. Nature Climate Change 5(3):207-14.

Gazeau F, Parker LM, Comeau S, Gattuso J-P, O'Connor WA, Martin S, Pörtner H-O, Ross PM. 2013.

Impacts of ocean acidification on marine shelled molluscs. Marine Biology 160(8):2207-45.

Gledhill D, White M, Salisbury J, Thomas H, Misna I, Liebman M, Mook B, Grear J, Candelmo A, Chambers RC, Gobler C, Hunt C, King A, Price N, Signorini S, Stancioff E, Stymiest C, Wahle R, Waller J, Rebuck N, Wang Z, Capson T, Morrison JR, Cooley S, Doney S. 2015. Ocean and coastalacidification off New England and Nova Scotia. Oceanography 25(2):182-97.

Global Invasive Species Database (GISD). 2020. [Internet]. Species profile: *Carcinus maenas*. (Accessed 3 Aug. 2020). http://www.iucngisd.org/gisd/species.php?sc=114.

Griffith AW, Gobler CJ. 2017. Transgenerational exposure of North Atlantic bivalves to ocean acidification renders offspring more vulnerable to low pH and additional stressors. Scientific Reports7:11394.

Hosack GR, Dumbauld BR, Ruesink JL, Armstrong DA. 2006. Habitat associations of estuarine species: comparisons of intertidal mudflat, seagrass (*Zostera marina*), and oyster (*Crassostrea gigas*) habitats. Estuaries and Coasts 29(6):1150-60.

International Council for the Exploration of the Sea (ICES). 1992. Report of the ICES working group on the effects of extraction of marine sediments on fisheries. Copenhagen (Denmark): ICES Cooperative Research Report # 182.

Johnson MR, Boelke C, Chiarella LA, Colosi PD, Greene K, Lellis-Dibble K, Ludemann H, Ludwig M, McDermott S, Ortiz J, Rusanowsky D, Scott M, Smith J. 2008. Impacts to marine fisheries habitat from nonfishing activities in the northeastern United States. NOAA Technical Memorandum. National Oceanic and Atmospheric Administration, National Marine Fisheries Service.NMFS-NE-2O9. 1-328.

Kroeker KJ, Kordas RL, Crim R, Hendriks IE, Ramajo L, Singh GS, Duarte CM, Gattuso J-P. 2013. Impacts of ocean acidification on marine organisms: quantifying sensitivities and interaction withwarming. Global Change Biology 19(6):1884-96.

Lewis CVW, Weinberg JR, Davis CS. 2001. Population structure and recruitment of the bivalve *Arctica islandica* (Linnaeus, 1767) on Georges Bank from 1980-1999. Journal of Shellfish Research 20: 1135-44.

McClenachan L, O'Connor G, Reynolds T. 2015. Adaptive capacity of co-management systems in the face of environmental change: the soft-shell clam fishery and invasive green crabs in Maine. Marine Policy 52:26-32.

Mid-Atlantic Fishery Management Council (MAFMC). 2019. [Internet]. Ocean quahog fishery information document. April 1, 2019. (Accessed 23 November 2020). http://www.mafmc.org/surfclams-quahogs.

Morley SA, Toft JD, Hanson KM. 2012. Ecological effects of shoreline armoring on intertidal habitats of a Puget Sound urban estuary. Estuaries and Coasts 35(3):774-84.

Pearce JB. 1994. Mining of seabed aggregates. *In*: Langton RW, Pearce JB, Gibson JA, editors. Selected living resources, habitat conditions, and human perturbations of the Gulf of Maine: environmental and ecological considerations for fishery management. Woods Hole, MA. NOAA Technical Memorandum. NMFS-NE-106. 48-50.

Salisbury J, Green M, Hunt C, Campbell J. 2008. Coastal acidification by rivers: a threat to shellfish? Eos, Transactions American Geophysical Union 89(50):513-28.

Seitz RD, Lipcius RN, Olmstead NH, Seebo MS, Lambert DM. 2006. Influence of shallow-water habitats and

shoreline development on abundance, biomass, and diversity of benthic prey and predators in Chesapeake Bay. Marine Ecology Progress Series 326:11-27.

Sharp JH, Culberson CH, Church TM. 1982. The chemistry of the Delaware estuary. General considerations. Limnology and Oceanography 27. doi:10.4319/lo.1982.27.6.1015.

Titus JG, Hudgens DE, Trescott DL, Craghan M, Nuckols WH, Hershner CH, Kassakian JM, Linn CJ, Merritt PG, McCue TM, O'Connell JF, Tanski J, Wang J. 2009. State and local governments plan for development of most land vulnerable to rising sea level along the US Atlantic coast. Environmental Research Letters 4(4):1-7.

Tyrrell MC, Byers JE. 2007. Do artificial substrates favor nonindigenous fouling species over native species? Journal of Experimental Marine Biology and Ecology 342(1):54-60.

U.S. Environmental Protection Agency (EPA). 2012. National Coastal Condition Report IV.EPA-R-842-10-003. US Environmental Protection Agency, Washington D.C. https://www.epa.gov/sites/production/files/2014-10/documents/0_nccr_4_report_508_bookmarks.pdf

Vandermeulen JH, Mossman D. 1996. Sources of variability in seasonal hepatic microsomal oxygenase activity in winter flounder (*Pleuronectes americanus*) from a coal tar contaminated estuary. Canadian Journal of Fisheries and Aquatic Sciences 53:1741-53.

Vos PC, van Kesteren WP. 2000. The long-term evolution of intertidal mudflats in the northern Netherlands during the Holocene; natural and anthropogenic processes. Continental Shelf Research20(12):1687-710.

Waldbusser GG, Hales B, Langdon CJ, Haley BA, Schrader P, Brunner EL, Gray MW, Miller CA,Gimenez I, Hutchinsonet G. 2015. Ocean acidification has multiple modes of action on bivalvelarvae. PLoS ONE 10(6):e0128376. https://doi.org/10.1371/journal.pone.0128376

Webber MM. 2013. [Internet]. Results of the one-day green crab trapping survey conducted along the Maine coast from August 27 to 28, 2013. Maine Department of Marine Resources, Augusta, ME. 6 pp. (Accessed 30 Oct. 2020). https://www.maine.gov/dmr/science-research/species/invasives/greencrabs/documents/trapsurveyrpt2013.pdf.

Marine Intertidal Sand Bottom

System: Marine

Subsystem: Intertidal

Class: Unconsolidated Bottom

Sub-class: Sand

Geographic Area: Entire Area

Overall Vulnerability Rank = High

Habitat Sensitivity = Moderate

Climate Exposure = Very High

	**Marine Intertidal Sand Bottom**	Attribute Mean	Data Quality	Distribution of Expert Scores
Sensitivity Attributes	Habitat condition	2.6	2.6	
	Habitat fragmentation	2.2	2.6	
	Distribution/Range	2.1	2.6	
	Mobility/Ability to spread or disperse	2	2.6	
	Resistance	2.2	2.2	
	Resilience	1.7	2.4	
	Sensitivity to changes in abiotic factors	1.6	2.4	
	Sensitivity and intensity of non−climate stressors	2.6	2.4	
	Dependency on critical ecological linkages	1.5	1.8	
	Sensitivity Component Score	Moderate		
Exposure Factors	Sea surface temp	n/a	n/a	
	Bottom temp	n/a	n/a	
	Air temp	4	3	
	River temp	n/a	n/a	
	Surface salinity	1.2	2.7	
	Bottom salinity	n/a	n/a	
	pH	4	2	
	Sea level rise	3.9	2.2	
	Precipitation	2.3	2.1	
	Floods	n/a	n/a	
	Droughts	n/a	n/a	
	Exposure Component Score	Very High		
**Overall Vulnerability Rank**		**High**		

Low
Moderate
High
Very High

Habitat Name: Marine Intertidal Sand Bottom

System**:** Marine

Subsystem: Intertidal

Class: Unconsolidated Bottom

Sub-class: Sand

Geographic Area: Entire Area

Habitat Description: This sub-class includes marine intertidal sand from mean high to the mean low water lines, where salinity is >30 ppt. This habitat also includes the epifauna and infauna associated with unconsolidated sand bottom, such as non-reef-forming mollusks (e.g., soft-shell clams, hard clams, sea scallops, surf clams, ocean quahogs), marine worms, small crustaceans, gastropods, and polychaetes. This subclass excludes specific habitats identified elsewhere (i.e., non-calcareous algal bed, rooted vascular beds, and reef-forming mollusks such as blue mussels and eastern oysters).

Overall Climate Vulnerability Rank: High (72% certainty from bootstrap analysis). The majority of the bootstrap results match the results of the categorical vulnerability rank, but 28% of the bootstrap results were in the Moderate vulnerability rank. This indicates that this habitat is in the low range of the High vulnerability rank.

Climate Exposure: Very High. Three exposure factors contributed to the High exposure score: Air Temperature (4.0), pH (4.0), and Sea Level Rise (SLR) (3.9). Air temperature and SLR are projected to increase, and pH is projected to decline, significantly throughout the region. Precipitation mean score was Moderate (2.3), primarily due to projected increases in the frequency and intensity of extreme rain events.The intertidal nature of this habitat places it at the nexus of significant atmospheric and oceanic change.

Habitat Sensitivity: Moderate. Only two sensitivity attribute mean scores were >2.2: Sensitivity and Intensity of Non-Climate Stressors and Habitat Condition (both 2.6). Resistance and Habitat Fragmentation both scored 2.2, and Distribution/Range was 2.1. The remainder of sensitivity attributes were ≤2.0. The low and moderate sensitivity scores likely reflect the general understanding that sand substrates are highly resistant and resilient to changes, and the infaunal and epifaunal organisms are relatively adapted to variable conditions.

Data Quality & Gaps: Two of the exposure factors had relatively high data quality: Surface Salinity (2.7) and Air Temperature (3.0), while the other three were relatively low (2.0-2.2). Data quality for climate exposure is lower for intertidal habitats due to the low resolution of CMIP5 and ROMS-NWA projections for climate exposure in nearshore, shallow coastal areas.

Only one of the nine sensitivity attribute data quality scores was ≤2 (Dependency on Critical Ecological Linkages, 1.8), while seven of the scores were either 2.4 or 2.6. This suggests that there was moderate confidence in understanding climate sensitivity of marine intertidal sand habitats.

Positive or Negative Climate Effect in the Northeast U.S.: The effect of climate change on marine intertidal sand in the Northeast U.S. is expected to be negative (95% of the experts' scores were negative, 5% were neutral). Experts' scores for marine intertidal sand were predominantly negative, likely reflecting experts' views of a substantial impact of erosion from sea level rise and storms, and rising temperatures and ocean acidification on this habitat.

Climate Effects on Habitat Condition and Distribution: Coast storms and sea level rise is projected to result in a loss of sand in the marine intertidal zone. Intertidal sand habitats are exposed to high wave energy and stormwater runoff during heavy rains, which increases erosion and habitat loss (Defeo et al. 2008). The frequency and intensity of heavy rain events are projected to increase in the Northeast region (USGCRP 2017). However, sandy beaches and their associated infauna and epifauna are generally well adapted to high

disturbance regimes, including changes in salinity, although recovery may take years depending on the magnitude of the storm (Lindholm et al. 2004).

Although the causes of sand shoreline erosion are complex and not all are related to climate change, the acceleration in sea level rise is expected to exacerbate beach erosion around the globe (Brown and McLachlan 2002; Nicholls et al. 2007; Chust et al. 2010;). Many sandy beaches will not recover from sea level rise, which are limited in their ability to migrate inland by coastal development and other anthropogenic barriers (Gutierrez et al. 2007). Sandy beaches are also impacted by seawalls and other forms of shoreline armoring, which exacerbate the effects of rising sea levels and further accelerate beach erosion (Defeo et al. 2008; O'Donnell 2017).

Infauna associated with intertidal sand habitats may be less sensitive than subtidal species to changes in air and water temperature because they are adapted to variability and extremes. However, some species appear to be affected by warming waters, and to a lesser degree water quality. Surf clam stocks in the Mid-Atlantic region have declined dramatically over the last 10 years, especially in New Jersey waters,and ocean quahog landings have shifted further north along the Atlantic coastline with substantial landings from southern New England waters (Lewis et al. 2001; MAFMC 2019). Increasing water temperatures, high nutrient levels, and algal blooms can trigger reductions in dissolved oxygen in the nearshore water column and in sediments, which can have detrimental effects to infaunal and epifaunal species associated with sand habitats (Sharp et al. 1982; Brownlee et al. 2005).

Mollusks are also sensitive to ocean acidification (Gazeau et al. 2013). In general, the larval life stage is more sensitive than juveniles and adults to ocean acidification (Kroeker et al. 2013). Ocean acidification is expected to reduce the shell size, thickness, calcification rates and growth, and survival of embryo and larval bivalves and gastropods (Gazeau et al. 2013). Some studies have shown additive and synergistic negative effects from ocean acidification and low dissolved oxygen on the growth, survival, and metamorphosis of larval bay scallop and hard clams (Griffith and Gobler 2017; Kroeker et al. 2013).

Nearshore coastal waters are generally more susceptible to acidification than oceanic waters because they are subject to more acid sources and are generally less buffered than oceanic waters (i.e., differences in the amount of dissolved inorganic carbon, dissolved and particulate organic carbon, total alkalinity and nutrients from riverine and estuarine sources) (Ekstrom et al. 2015; Gledhill et al. 2015).

Habitat Summary: Sandy intertidal habitats are relatively continuous throughout the Mid-Atlantic, but are more fragmented in the northern parts of New England (NOAA 2020). Sandy beaches vary in condition throughout the Northeast, with some undeveloped and undisturbed and others heavily degraded(Rice 2017). Sandy beach habitats are affected by natural perturbations such as storms, sediment transport, and tides, as well as a variety of human uses such as recreation, transport, development/hardening, dredging/filling, nourishment, and pollution (Johnson et al. 2008). One of the biggest threats facing sandy intertidal habitats is coastal squeeze, where beaches are trapped between rising sea levels and erosion, and human development on the other (Defeo et al. 2008). Titus et al. (2009)estimated that almost 60% of land within 1 m above the high tide line on the U.S. Atlantic coast (Florida to Massachusetts) is developed or expected to be developed.

According to the 2012 EPA Coastal Condition Report, sediment quality in the Northeast Coast region (Chesapeake Bay and north) is rated fair, with 12% of the coastal area in poor condition and 11% in fair condition, largely driven by sediment contamination from heavy metals, polychlorinated biphenyls, and pesticies, with poorer quality sediments near urban areas (EPA 2012). Coarse-grained sediments such as sand generally contain less total organic carbon (TOC) levels compared to soft-grained sediments. Because contaminants such aspolyaromatic hydrocarbons (PAH), pesticides, and polychlorinated biphenyl are sequestered in the TOC fraction of sediments, sand sediments tend to be less impacted by contaminants than mud substrates (ICES 1992; Pearce 1994). PAH can persist in sediments for decades after the initial contamination, causing disruption of physiological and metabolic processes of benthic organisms

(Vandermeulen and Mossman 1996). Southern New England and Mid-Atlantic coastal areas have high contaminant loads dueto legacy pollution from industrialization, and continued urbanization (EPA 2012). Benthic infauna and epifauna are sensitive to contaminants, which can have toxic effects on most species. Overall, further degradation of water and sediment quality is expected as urbanization and development in the coastal zone continues with increasing population growth.

Shoreline hardening can prevent inland migration of sandy beaches. Riprap revetment can convert sandy bottom to large diameter, engineered stone in the intertidal zone. All hardened shorelines have the potential to erode intertidal sand bottom. Hardened shorelines have been shown to have lower abundance, biomass, and diversity of benthic prey and predators (Kraus and McDougal 1996). Other effects of engineered-shore structures include loss of sediment and reductions in beach volume and dimension, loss of intertidal habitat and habitat fragmentation (NRC 2007; Bulleri and Chapman 2010), and can have higher incidence of marine exotic/invasive species compared to native material (Geraldi et al. 2013).

Beach renourishment, widely used to combat shoreline erosion, can also damage sandy beach habitats through burial and changes in beach morphology (Defeo et al. 2008). Sand sediments and associated infauna are sensitive to dredging impacts, though most species recover relatively rapidly (Wilber et al.2005; Rice 2017).

Intertidal infauna and epifauna are sensitive to invasive species, including green crab (*Carcinus maenas*), which has established populations in New England and northern Mid-Atlantic in both subtidal and intertidal zones. It is a predator of many forms of marine life, including worms and mollusks (GISD 2020). In some areas (particularly New England), the crab's voracious appetite has affected the commercial shellfish industry (Webber 2013; Beal 2014).

Literature Cited

Beal BF. 2014. [Internet]. Final report: Green crab, *Carcinus maenas*, trapping studies in the Harraseeket River and manipulative field trials to determine effects of green crabs on the fate and growth of wild and cultured individuals of soft-shell clams, *Mya arenaria* (May to November 2013). Downeast Institute for Applied Marine Research and Education, Beals, ME. 76 pp. (Accessed 30 Oct. 2020). https://downeastinstitute.org/wp-content/uploads/2018/08/1_24-final-report-freeport-shellfish-restoration-project-b.-beal.pdf.

Brown AC, McLachlan A. 2002. Sandy shore ecosystems and the threats facing them: some predictionsfor the year 2025. Environmental Conservation 29(1):62-77.

Brownlee EF, Sellner SG, Sellner K.G. 2005. *Prorocentrum minimum* blooms: potential impacts on dissolved oxygen and Chesapeake Bay oyster settlement and growth. Harmful Algae 4 (3):593-602. https://doi.org/10.1016/j.hal.2004.08.009

Bulleri F, Chapman MG. 2010. The introduction of coastal infrastructure as a driver of change in marine environments. Journal of Applied Ecology 47(1):26-35.

Chust G, Caballero A, Marcos M, Liria P, Hernández C, Borja Á. 2010. Regional scenarios of sea level rise and impacts on Basque (Bay of Biscay) coastal habitats, throughout the 21st century. Estuarine,Coastal and Shelf Science 87(1):113-24.

Defeo O, McLachlan A, Schoeman DS, Schlacher TA, Dugan J, Jones A, Lastra M, Scapini F. 2008.Threats to sandy beach ecosystems: a review. Estuarine, Coastal and Shelf Science 81(1):1-12.

Ekstrom JA, Suatoni L, Cooley SR, Pendleton LH, Waldbusser GG, Cinner JE, Ritter J, Langdon C, van

Hooidonk R, Gledhill D, Wellman K, Beck MW, Brander LM, Rittschof D, Doherty C, Edwards PET, Portela R. 2015. Vulnerability and adaptation of US shellfisheries to ocean acidification. Nature Climate Change 5(3):207-14.

Gazeau F, Parker LM, Comeau S, Gattuso J-P, O'Connor WA, Martin S, Pörtner H-O, Ross PM. 2013. Impacts of ocean acidification on marine shelled molluscs. Marine Biology 160(8):2207-45.

Gledhill D, White M, Salisbury J, Thomas H, Misna I, Liebman M, Mook B, Grear J, Candelmo A, Chambers RC, Gobler C, Hunt C, King A, Price N, Signorini S, Stancioff E, Stymiest C, Wahle R, Waller J, Rebuck N, Wang Z, Capson T, Morrison JR, Cooley S, Doney S. 2015. Ocean and coastalacidification off New England and Nova Scotia. Oceanography 25(2):182-97.

Global Invasive Species Database (GISD). 2020. [Internet]. Species profile: *Carcinus maenas*. (Accessed on 03 Aug. 2020). http://www.iucngisd.org/gisd/species.php?sc=114.

Geraldi NR, Smyth AR, Piehler MF, Peterson CH. 2013. Artificial substrates enhance non-nativemacroalga and N2 production. Biological Invasions 16(9):1819-31.

Griffith AW, Gobler CJ. 2017. Transgenerational exposure of North Atlantic bivalves to ocean acidification renders offspring more vulnerable to low pH and additional stressors. Scientific Reports7:11394.

Gutierrez BT, Williams SJ, Thieler ER. 2007. Potential for shoreline changes due to sea-level rise along the U.S. Mid-Atlantic region. U.S. Geological Survey. Open-File Report 2007-1278. 1-25.

International Council for the Exploration of the Sea (ICES). 1992. Report of the ICES working group on the effects of extraction of marine sediments on fisheries. Copenhagen (Denmark): ICES Cooperative Research Report # 182.

Johnson MR, Boelke C, Chiarella LA, Colosi PD, Greene K, Lellis-Dibble K, Ludemann H, Ludwig M, McDermott S, Ortiz J, Rusanowsky D, Scott M, Smith J. 2008. Impacts to marine fisheries habitat from nonfishing activities in the northeastern United States. NOAA Technical Memorandum. National Oceanic and Atmospheric Administration, National Marine Fisheries Service.NMFS-NE-2O9. 1-328.

Kraus NC, McDougal WG. 1996. The effects of seawalls on the beach: Part 1, an updated literature review. Journal of Coastal Research 12(3):691-701.

Kroeker KJ, Kordas RL, Crim R, Hendriks IE, Ramajo L, Singh GS, Duarte CM, Gattuso J-P. 2013. Impacts of ocean acidification on marine organisms: quantifying sensitivities and interaction withwarming. Global Change Biology 19(6):1884-96.

Lewis CVW, Weinberg JR, Davis CS. 2001. Population structure and recruitment of the bivalve *Arctica islandica* (Linnaeus, 1767) on Georges Bank from 1980-1999. Journal of Shellfish Research 20: 1135-44.

Lindholm J, Auster P, Valentine P. 2004. Role of a large marine protected area for conserving landscape attributes of sand habitats on Georges Bank (NW Atlantic). Marine Ecology Progress Series 269:61-8.

Mid-Atlantic Fishery Management Council (MAFMC). 2019. [Internet]. Ocean quahog fishery information document. April 1, 2019. (Accessed 23 November 2020). http://www.mafmc.org/surfclams-quahogs.

National Oceanic and Atmospheric Administration (NOAA). 2020. [Internet]. Environmental SensitivityIndex Maps and GIS Data. 2019. [Internet]. NOAA Office of Response and Restoration. (Accessed 8Dec. 2020). https://response.restoration.noaa.gov/maps-and-spatial-data/download-esi-maps-and-gis-data.html

National Research Council (NRC). 2007. Mitigating eroding sheltered shorelines: a trade-off in ecosystem services. Mitigating shore erosion along sheltered coasts. p. 78-97. Washington, DC:National Research Council.

Nicholls RJ, Wong PP, Burkett V, Codignotto J, Hay J, McLean R, Ragoonaden S, Woodroffe CD. 2007. Coastal systems and low-lying areas. Climate change 2007: impacts, adaptation and vulnerability. Contribution of Working Group II to the Fourth Assessment Report of the Intergovernmental Panel on Climate Change. [Parry ML, Canziani OF, Palutikof JP, van der Linden PJ, Hanson CE (eds)]. Intergovernmental Panel on Climate Change. 315-56.

O'Donnell JE. 2017. Living shorelines: a review of literature relevant to New England coasts. Journal of Coastal Research 33(2):435-51.

Pearce JB. 1994. Mining of seabed aggregates. *In*: Langton RW, Pearce JB, Gibson JA, editors. Selected living resources, habitat conditions, and human perturbations of the Gulf of Maine: environmental and ecological considerations for fishery management. Woods Hole, MA. NOAA Technical Memorandum. NMFS-NE-106. 48-50.

Rice TM. 2017. Inventory of habitat modifications to sandy ocean front beaches in the U.S. Atlantic coast breeding range of the piping plover (*Charadrius melodus*) as of 2015: Maine to North Carolina. Report submitted to the U.S. Fish and Wildlife Service, Hadley, Massachusetts. 295 p. https://northatlanticlcc.org/teams/coastal-resiliency/projects/beach-and-tidal-inlet-habitat-inventories/inventory-of-habitat-modifications-to-sandy-oceanfront-beaches-in-2015/index_html.

Sharp JH, Culberson CH, Church TM. 1982. The chemistry of the Delaware estuary. General considerations. Limnology and Oceanography 27. doi:10.4319/lo.1982.27.6.1015.

Titus JG, Hudgens DE, Trescott DL, Craghan M, Nuckols WH, Hershner CH, Kassakian JM, Linn CJ, Merritt PG, McCue TM, O'Connell JF, Tanski J, Wang J. 2009. State and local governments plan for development of most land vulnerable to rising sea level along the US Atlantic coast. Environmental Research Letters 4(4):1-7.

U.S. Environmental Protection Agency (EPA). 2012. National Coastal Condition Report IV.EPA-R-842-10-003. US Environmental Protection Agency, Washington D.C. https://www.epa.gov/sites/production/files/2014-10/documents/0_nccr_4_report_508_bookmarks.pdf.

U.S. Global Change Research Program (USGCRP). 2017. Climate Science Special Report: Fourth National Climate Assessment. Volume I [Wuebbles, D.J., D.W. Fahey, K.A. Hibbard, D.J. Dokken, B.C. Stewart, and T.K. Maycock (eds.)]. U.S. Global Change Research Program. Washington, DC.USA. 470 pp. doi: 10.7930/J0J964J6.

Vandermeulen JH, Mossman D. 1996. Sources of variability in seasonal hepatic microsomal oxygenase activity in winter flounder (*Pleuronectes americanus*) from a coal tar contaminated estuary. Canadian Journal of Fisheries and Aquatic Sciences 53:1741-53.

Webber MM. 2013. [Internet]. Results of the one-day green crab trapping survey conducted along the Maine coast from August 27 to 28, 2013. Maine Department of Marine Resources, Augusta, ME. 6 pp. (Accessed 30 Oct. 2020). https://www.maine.gov/dmr/science-research/species/invasives/greencrabs/documents/trapsurveyrpt2 013.pdf.

Wilber DH, Brostoff W, Clarke DG, Ray GL. 2005. Sedimentation: Potential biological effects from dredging

operations in estuarine and marine environments. DOER Technical Notes Collection (ERDCTN-DOER-E20). U.S. Army Engineer Research and Development Center, Vicksburg, MS.

Marine Kelp

System: Marine

Subsystem: Subtidal

Class: Aquatic Bed

Sub-class: Kelp

Geographic Area: Entire Area

Overall Vulnerability Rank = High

Habitat Sensitivity = High

Climate Exposure = High

	Marine Kelp	Attribute Mean	Data Quality	Distribution of Expert Scores
Sensitivity Attributes	Habitat condition	3.2	2.6	
	Habitat fragmentation	3	2.6	
	Distribution/Range	3.4	2.6	
	Mobility/Ability to spread or disperse	2.5	2.2	
	Resistance	2.6	2.4	
	Resilience	2.4	2.4	
	Sensitivity to changes in abiotic factors	2.5	2.4	
	Sensitivity and intensity of non−climate stressors	3.2	2.4	
	Dependency on critical ecological linkages	3	2	
	Sensitivity Component Score	High		
Exposure Factors	Sea surface temp	3.9	3	
	Bottom temp	n/a	n/a	
	Air temp	n/a	n/a	
	River temp	n/a	n/a	
	Surface salinity	1.3	2.8	
	Bottom salinity	n/a	n/a	
	pH	4	2.2	
	Sea level rise	2	2.2	
	Precipitation	n/a	n/a	
	Floods	n/a	n/a	
	Droughts	n/a	n/a	
	Exposure Component Score	High		
**Overall Vulnerability Rank**		**High**		

Low
Moderate
High
Very High

Habitat Name: Marine Kelp

System**:** Marine

Subsystem: Subtidal

Class: Aquatic Bed

Sub-class: Kelp

Geographic Area: Entire Area

Habitat Description: This sub-class includes marine subtidal kelp, which are non-rooted, brown algae of the order Laminariales, and are important components of nearshore, subtidal benthic communities. Kelp can form dense beds on rocky bottom habitat in the photic zone to depths of 10–25 m (Merzouk and Johnson 2011). Lopez et al. (2014) reported few kelp occur deeper than 5 m in Long Island Sound.

Suitable habitat for kelp is associated with areas rocky bottom and well mixed waters. The two largest brown algae species in the U.S. are sugar kelp (*Saccharina latissima*) and horsetail kelp (*Laminaria digitata*), although shotgun kelp (*Agarum clathratum*) and winged kelp (*Alaria esculenta*) are also prevalent in the study area. The historic U.S. range is Gulf of Maine to Long Island Sound, although the densities of both species in Long Island Sound are substantially reduced (Van Patton and Yarish 2009; Merzouk and Johnson 2011; Lopez et al. 2014; Wilson et al. 2019; Auster P, pers. comm. 2020). This habitat subclass also includes aquaculture (i.e., kelp farming). Commercial aquaculture of macroalgae ispredominantly for rockweed (*Ascophyllum nodosum*) (included in the non-kelp macroalgae habitat narrative), although *S. latissima* and *L. digitata* are also cultured.

Overall Climate Vulnerability Rank: High (100% certainty from bootstrap analysis).

Climate Exposure: High. The overall High exposure score was influenced by two Very High attribute means: Sea Surface Temperature (3.9) and pH (4.0). The exposure means for Surface Salinity and Sea Level Rise (SLR) were 1.3 and 2.0, respectively. The current geographic range of kelp in the study area includes Long Island Sound and the Gulf of Maine, where sea surface temperature is projected to have the greatest change in the study area. Although the projected change in pH for the Gulf of Maine is less than southern New England and the Mid-Atlantic, it is still expected to drastically decrease from historic levels.

Habitat Sensitivity: High. Five of the nine sensitivity attributes means were ≥ 3.0, while the other attributes had scores between 2.4 and 2.6. The highest sensitivity attribute means were for Distribution/Range (3.4), Habitat Condition, and Sensitivity and Intensity of Non−Climate Stressors (both3.2). The means for two of the attributes (Mobility/Ability to Spread or Disperse and Sensitivity to Changes in Abiotic Factors) were more moderate than the others.

Data Quality & Gaps: The data quality scores for two of the four climate exposure factors (pH and SLR) were scored relatively Low (2.2). For pH, this is likely attributed to the low resolution of ROMS-NWA projections for nearshore, shallow areas. In addition, because comprehensive mapping andbaseline data for kelp in the study area is lacking, text descriptions for spatial distribution of kelp were used for the climate exposure scoring.

For habitat sensitivity, only two of the nine attributes had relatively Low (2.2 and 2.0) data quality scores (Mobility/Ability to Spread or Disperse and Dependency on Critical Ecological Linkages). The remaining attributes were 2.4 or 2.6.

Positive or Negative Climate Effect for the Northeast U.S.: The effect of climate change on marine kelp in the Northeast U.S. is expected to be negative (100% of the experts' scores were negative).

Climate Effects on Habitat Condition and Distribution: Higher mean temperatures and heat waves have been attributed to reductions in abundance and range of kelp in the Gulf of Maine (Krumhansl et al.2016;

Witman and Lamb 2018) and southern New England (Lopez et al. 2014; Feehan et al. 2019), and climate projections indicate these trends will continue. The projected northward shift of the trailing edge of kelp species in the Northwestern Atlantic is attributed to reductions in growth and complete mortality, leading to a 36% reduction of *L. digitata* and 21% of *S. latissima* habitats for the end-of-century time frame under the RCP8.5 scenario (Wilson et al. 2019). Feehan et al. (2019) documented the decline of *S. latissima* and its replacement by turf-forming macroalgae in Narragansett Bay, Rhode Island, between 1980 and 2018, which they attributed to increasing water temperature. Turf algae generally refersto low-lying (<10 cm tall) species with densely packed fronds, lax and filamentous branches (e.g., *Cladophora, Ulva, and Polysiphonia*) (Connell et al. 2014).

Feehan et al. (2019) reported kelp attached to turf algae required significantly less force to detach from the substrate, and a pattern of lower survival following major storm events compared to rock-attached kelp. Witman and Lamb (2018) also reported higher kelp mortality with more storm disturbance, and suggested increasing wave disturbance from climate change, as well as warmer temperatures, may also contribute to the future loss of kelp foundation species. Storms are expected to increase in frequency and intensity in the Northeast region (USGCRP 2017), and increases in wave energy exposure could have deleterious effects on shallow-water habitats. Based on the results of CMIP5 RCP8.5, Lehmann et al. (2014) concluded that the northeast region would likely experience an increase in the frequency and intensity of winter extratropical cyclone events, but a slight decrease in summer events, by 2100. Colle et al. (2013) projected extratropical cyclones may become more intense (10-40%) along the northeast coast, especially during the mid-21st century as a result of an increase in latent heat release due to a moister atmosphere. The northeast region is also affected by tropical cyclone systems that originate in the Atlantic and Caribbean basins. Increases in sea surface temperatures may increase the maximum potential intensity of tropical cyclones and should be reflected by an increase in the frequency of the strongest hurricanes (Trenberth 2005; Kossin et al. 2007; Knutson et al. 2010). Between the 1970s and early 2000s,the number of major hurricanes (Category 4 and 5) in an average year approximately doubled (Emanuel 2005; Webster et al. 2005).

The projected change in pH for marine macroalgae species in the study area is very high. However, kelp are fleshy, non-calcifying algae, and are believed to have low sensitivities to pH and carbonate chemistry (Koch et al. 2013; Kroeker et al. 2013). Some marine macroalgae have shown higher growth rates under experimental elevated pCO_2 conditions through higher photosynthetic and growth rates (Gledhill et al. 2015; Young and Gobler 2016), which could mitigate some climate-related impacts associated withwarming waters.

<u>Habitat Summary</u>: Kelp are increasingly threatened by a variety of non-climate local stressors including overfishing, pollution, disease, herbivory, storms, and warming waters, and regional variation of these drivers may affect kelp populations (Steneck et al. 2002). Sea urchins are a natural predator of large brown algal species, including kelp, and in some areas urchins have decimated kelp beds (i.e., "urchin barrens"). There is evidence that overfishing of top predators may contribute to explosions of urchin populations (Steneck et al. 2002).

Wilson et al. (2019) suggested potential climate-induced shifts in dominance from native kelp species to invasive species, such as green algae (*Codium fragile*), and a loss of kelp may facilitate the transition to a turf algae dominated ecosystem. Ecosystem changes observed throughout the study area (e.g., warming waters, increasing intensity, frequency, and duration of coastal storms, increasing prevalence of invasive species and herbivory, and exposure to stormwater pollution) have been attributed to patterns of long-term shifts from kelp- to turf-dominated habitats in New England and the Gulf of Maine (Steneck et al. 2002; Dijkstra et al. 2017a; Dijkstra et al. 2017b; Filbee-Dexter and Wernberg 2018; Dijkstra et al. 2019; Feehan et al. 2019).

Warming ocean waters have been shown to eliminate thermal barriers that historically limit reproductive success of marine invasive species (e.g., the tunicate *Botrylloides violaceus*), which may impact kelp abundance and distribution (Dijkstra et al. 2017a). Increased reproduction of marine invasive species, combined with limited biological resistance in regions with cooler water temperatures, may lead to a

community state change. Marine invasive species, likely introduced via maritime transport vectors, are known to compete for space and foul benthic substrates (Pappal 2010) and may replace kelp species in portions of the study area (Scheibling and Gagnon 2006; Trott and Entreline 2019). *S. latissima* underwent a significant 36.2% decrease in abundance between 1987 and 2015 on Cashes Ledge, concurrent with a rapid warming of the GOM and invasion by the kelp-encrusting bryozoan *Membranipora membranacea* (Scheibling and Gagnon 2006; Witman and Lamb 2018).

Kelp requires moderate nutrient levels and well-mixed waters for optimal growth, as well as exposed rocky bottom (Steneck et al. 2002). Kelp abundance decreases in very wave sheltered areas (Bekkby et al.2019), and offshore densities of *S. latissima* were over 150 times greater than at coastal sites with similar but lower magnitude trends for congeneric *S. digitata* (Witman and Lamb 2018). This suggests kelp in marine waters may be more resilient to change compared to kelp in estuarine waters. In addition, kelp in marine waters are exposed to fewer anthropogenic impacts associated with nearshore coastal waters. The shallow kelp forest communities in some areas of the Gulf of Maine (e.g., Cashes Ledge) represent an oasis of unusually high kelp and fish abundance in the region, and as such, comprise a persistent abundance hotspot that is functionally significant for sustained biological productivity of offshore regionsin the study area (Witman and Lamb 2018).

Kelp requires rock bottom habitat to attach to the substrate, so placement of revetments, jetties, groins, and other structures in the marine environment (e.g., scour protection on offshore wind farms) may increase habitat availability if other habitat requirements are met. Although commercial and recreational harvest and culturing of kelp and rockweed occurs in Maine, there is little information available suggesting widespread impacts associated with controlled harvesting. However, at least one study (Wilsonet al. 2019) projects the geographic range of kelp will shift northward and contract in the study area (i.e., 36% reduction of *L. digitata* and 21% of *S. latissima* by the end of the century under the RCP8.5 scenario). Future harvests of these species may be problematic as abundance and distribution declines.

Literature Cited

Bekkby T, Smit C, Gundersen H, Rinde E, Steen H, Tveiten L, Gitmark JK, Fredriksen S, Albretsen J and Christie H. 2019. The abundance of kelp is modified by the combined impact of depth, waves and currents. Frontiers of Marine Science 6:475. doi:10.3389/fmars.2019.00475.

Colle BA, Zhang Z, Lombardo KA, Chang E, Liu P, Zhang M. 2013. Historical evaluation and future prediction of eastern North American and western Atlantic extratropical cyclones in the CMIP5 models during the cool season. Journal of Climate 26(18):6882-903.

Connell SD, Foster MS, Airoldi L. 2014. What are algal turfs? Towards a better description of turfs. Marine Ecology Progress Series 495:299–307.

Dijkstra JA, Westerman EL, Harris LG. 2017a. Elevated seasonal temperatures eliminate thermal barriers of reproduction of a dominant invasive species: A community state change for northern communities? Diversity and Distributions 23:1182–92.

Dijkstra JA, Harris LG, Mello K, Litterer A, Wells C, Ware C. 2017b. Invasive seaweeds transform habitat structure and increase biodiversity of associated species. Journal of Ecology 105: 1668-78. https://doi.org/10.1111/1365-2745.12775.

Dijkstra JA, Litterer A, Mello K, O'Brien BS, Rzhanov Y. 2019. Temperature, phenology, and turf macroalgae drive seascape change: Connections to mid-trophic level species. Ecosphere 10(11):e02923. https://doi.org/10.1002/ecs2.2923.

Emanuel K. 2005. Increasing destructiveness of tropical cyclones over the past 30 years. Nature 436(7051):686-8.

Feehan CJ, Grace SP, Narvaez CA. 2019. Ecological feedbacks stabilize a turf-dominated ecosystem at the southern extent of kelp forests in the Northwest Atlantic. Scientific Reports 9:7078. https://doi.org/10.1038/s41598-019-43536-5.

Filbee-Dexter K, and Wernberg T. 2018. Rise of turfs: A new battlefront for globally declining kelp forests. Bio-Science 68:64–76. https://doi.org/10.1093/biosci/bix147.

Gledhill DK, White MM, Salisbury J, Thomas H, Misna I, Liebman M, Mook B, Grear J et al. 2015. Ocean and coastal acidification off New England and Nova Scotia. Oceanography 28(2):182–97. http://dx.doi.org/10.5670/oceanog.2015.41.

Knutson TR, McBride JL, Chan J, Emanuel K, Holland G, Landsea C, Held I, Kossin JP, Srivastava AK, Sugi M. 2010. Tropical cyclones and climate change. Nature Geoscience 3(3):157-63.

Koch M, Bowes G, Ross C, Zhang X-H. 2013. Climate change and ocean acidification effects on seagrasses and marine macroalgae. Global Change Biology 19(1):103-32.

Kossin JP, Knapp KR, Vimont DJ, Murnane RJ, Harper BA. 2007. A globally consistent reanalysis of hurricane variability and trends. Geophysical Research Letters 34(4):1-6.

Kroeker KJ, Kordas RL, Crim R, Hendriks IE, Ramajo L, Singh GS, Duarte CM, Gattuso J-P. 2013. Impacts of ocean acidification on marine organisms: quantifying sensitivities and interaction with warming. Global Change Biology 19(6):1884-96.

Krumhansl KA, Okamoto DK, Rassweiler A, Novak M, Bolton JJ, Cavanaugh KC, Connell SD, Johnson CR et al. 2016. Global patterns of kelp forest change over the past half-century. Proceedings of the National Academy of Sciences 113 (48):13785-90. https://doi.org/10.1073/pnas.1606102113.

Lehmann J, Coumou D, Frieler K, Eliseev AV, Levermann A. 2014. Future changes in extratropical storm tracks and baroclinicity under climate change. Environmental Research Letters 9:8.

Lopez G, Carey D, Carlton J, Cerrato R, Dam H, DiGiovanni R, Elphick C, Frisk M, Gobler C, Hice L, Howell P, Jordaan A, Lin S, Liu S, et al. 2014. Biology and Ecology of Long Island Sound. *In*: Latimer J, Tedesco M, Swanson R, Yarish C, Stacey P, Garza C. (eds), Long Island Sound. SpringerSeries on Environmental Management. Springer, New York, NY. https://doi.org/10.1007/978-1-4614-6126-5_610.1007/978-1-4614-6126-5_6

Merzouk A, Johnson LE. 2011. Kelp distribution in the northwest Atlantic Ocean under a changing climate. Journal of Experimental Marine Biology and Ecology 400(1–2):90-8. https://doi.org/10.1016/j.jembe.2011.02.020.

Pappal A. 2010. Marine invasive species. State of the Gulf of Maine Report. [Currier P, Gould D, Hertz L, Huston J, Tremblay ML (eds)]. The Gulf of Maine Council on the Marine Environment. 1-21.

Scheibling RE, Gagnon P. 2006. Competitive interactions between the invasive green alga *Codium fragile*s ssp. *tomentosoides* and native canopy-forming seaweeds in Nova Scotia (Canada). Marine Ecology Progress Series 325:1–14. DOI:10.3354/meps325001.

Steneck R, Graham MH, Bourque BJ, Corbett D, Erlandson JM, Estes JA, Tegner MJ. 2002. Kelp forest

ecosystems: biodiversity, stability, resilience and future. Marine Sciences Faculty Scholarship. 65. https://digitalcommons.library.umaine.edu/sms_facpub/65.

Trenberth K. 2005. Uncertainty in hurricanes and global warming. Science 308(5729):1753-4.

Trott TJ, Enterline C. 2019. First record of the encrusting bryozoan *Cribrilina (Juxtacribrilina) mutabilis* (Ito, Onishi and Dick, 2015) in the Northwest Atlantic Ocean. BioInvasions Records 8.

USGCRP. 2017. Climate Science Special Report: Fourth National Climate Assessment, Volume I [Wuebbles, D.J., D.W. Fahey, K.A. Hibbard, D.J. Dokken, B.C. Stewart, and T.K. Maycock (eds.)]. U.S. Global Change Research Program. Washington, DC, USA. 470 pp. doi: 10.7930/J0J964J6.

Van Patten MS, Yarish C. 2009. Seaweeds of Long Island Sound. Bulletin 39. Connecticut College Arboretum.

Webster PJ, Holland GJ, Curry JA, Chang H-R. 2005. Changes in tropical cyclone number, duration, and intensity in a warming environment. Science 309(5742):1844-6.

Wilson KL, Skinner MA, Lotze HK. 2019. Projected 21st-century distribution of canopy-forming seaweeds in the Northwest Atlantic with climate change. Diversity and Distributions 25: 582–602. https://doi.org/10.1111/ddi.12897.

Witman JD, Lamb RW. 2018. Persistent differences between coastal and offshore kelp forest communities in a warming Gulf of Maine. PLoS ONE 13(1): e0189388. https://doi.org/.10.1371/journal.pone.0189388.

Young CS, Gobler CJ. 2016. Ocean acidification accelerates the growth of two bloom-forming macroalgae. PLoS ONE 11(5):e0155152. doi:10.1371/journal.pone.0155152.

Marine Red, Green, Small-brown Algae

System: Marine

Subsystem: Subtidal & Intertidal

Class: Aquatic Bed

Sub-class: Red, Green, Small-brown Algae

Geographic Area: Entire Area

Overall Vulnerability Rank = Low

Habitat Sensitivity = Low

Climate Exposure = High

Marine Red, Green, and Small-brown Algae		Attribute Mean	Data Quality	Distribution of Expert Scores
Sensitivity Attributes	Habitat condition	1.9	2.2	
	Habitat fragmentation	1.7	2.2	
	Distribution/Range	1.5	2.2	
	Mobility/Ability to spread or disperse	1.5	2.2	
	Resistance	1.5	2.2	
	Resilience	1.4	2.2	
	Sensitivity to changes in abiotic factors	1.5	2.2	
	Sensitivity and intensity of non−climate stressors	1.8	2.2	
	Dependency on critical ecological linkages	1.5	2	
	Sensitivity Component Score	Low		
Exposure Factors	Sea surface temp	3.6	3	
	Bottom temp	n/a	n/a	
	Air temp	n/a	n/a	
	River temp	n/a	n/a	
	Surface salinity	1.3	2.8	
	Bottom salinity	n/a	n/a	
	pH	4	2	
	Sea level rise	2.7	2.2	
	Precipitation	2.4	2.1	
	Floods	n/a	n/a	
	Droughts	n/a	n/a	
	Exposure Component Score	High		
**Overall Vulnerability Rank**		**Low**		

Low
Moderate
High
Very High

Habitat Name: Marine Red, Green, and Small-brown Algae
System**:** Marine
Subsystem: Subtidal and Intertidal
Class: Aquatic Bed
Sub-class: Red, Green, and Small-brown Algae
Geographic Area: Entire Area

Habitat Description: This sub-class includes marine red, green, and small brown (non-kelp) algal species that are non-rooted, benthic macrophytes occurring in both subtidal and intertidal zones. Red, green, and small brown algae photosynthesize, so are limited to the photic zone. Red, green, and brown algae are distributed across the entire study area and the sub-class contains a number of species, although there is considerable geographic variability among the species related to temperature tolerances and ecological requirements. A total of 316 macroalgae taxa have been identified from four Gulf of Maine embayments, including 81 green, 111 brown, and 124 red algae (Mathieson et al. 2010). Van Patton and Yarrish (2009) estimated 250 species of macroalgae in the Long Island Sound, and Orris (1980) listed 62 species from Maryland waters of the Chesapeake Bay (note the Gulf of Maine and Long Island Sound assessments include species of kelp).

Most species of non-kelp macroalgae that occur in the intertidal zone are restricted to lower and middle elevations due to their sensitivities to dessication, although green algae can occur from the uppermost partof the intertidal zone to the mid-intertidal zone (Van Patton and Yarish 2009). Red algae can absorb green and blue light, thus are capable of growing in greater depths of the subtidal zone. The red algae group contains many species and its morphologies can take the form of crusts, filamentous and branching, blades and sheets. There are also species of calcifying coralline algae, which are evaluated in the narrativefor marine rocky bottom habitat. Commercial aquaculture of non-kelp macroalgae is predominantly for rockweed, *Ascophyllum nodosum*.

Examples of some of the species for this sub-class include:
Small brown (non-kelp) algae: rockweeds *Ascophyllum nodosum* and *Fucus* spp., Sargasso weed *Sargassum filipendula*;
Red algae: Irish moss *Chondrus crispus, Gracilaria* spp., dulse *Palmaria palmata*, nori *Porphyra* spp., *Phyllophora pseudoceranoides,* Polly *Polysiphonia* spp.;
Green algae: sea lettuce *Ulva lactuca*, *Cladophora* spp, dead man's fingers *Codium fragile*.

Twenty taxa of non-indigenous algal species are known from the Northwest Atlantic, including two subspecies of the green alga *Codium fragile*, four brown algae, and fourteen red algae (Mathieson et al. 2008). Several species of introduced macroalgae found in the study area are also considered invasive and nuisance species, such as *Dasysiphonia japonica*, *C. fragile*, *Polysiphonia harveyi, Grateloupia turuturu, Gracilaria vermiculophylla,* and several species of *Porphyra* (Mathieson et al. 2008; Witman and Lamb 2018). These species are often more tolerant of anthropogenic impacts, and in particular warming waters,and in some cases compete with indigenous algal and other benthic species for space and resources (Scheibling and Gagnon 2006; Trott and Entreline 2019).

Overall Climate Vulnerability Rank: Low (100% certainty from bootstrap analysis).

Climate Exposure: High. The overall high exposure score was influenced by Sea Surface Temperature (3.6), pH (4.0), Sea Level Rise (SLR) (2.7), and Precipitation (2.4). The exposure mean for Surface Salinity was 1.3. Changes to Sea Surface Temperature, pH, and SLR in the nearshore photic zone where algal species occur is projected to be high or very high throughout the study area.

Habitat Sensitivity: Low. All nine of the sensitivity attributes means were ≤1.9, and six were ≤1.5, indicating that most small brown, red, and green algal populations in the marine system are in reasonably good condition

and have low sensitivity to climate and non-climate perturbations. The tolerance of the many species of non-indigenous macroalgae species to warming waters and anthropogenic effects likely contributes to the low sensitivity ranking for this subclass.

Although the overall climate sensitivity was considered low for non-kelp macroalgae in both estuarine and marine systems, it should be noted that the distribution of scores were distinct between the two systems. There were more scores across all of the attributes in the High bin for the estuarine system than the marine system (50 vs 16), and there were more scores in the Low bin for the marine system compared to the estuarine system (112 vs 65). Although some of these differences could be attributed to having different scorers in the two systems, the differences may reflect an understanding that estuarine system macroalgae are exposed to higher intensity and frequency of anthropogenic stressors than macroalgae in the marine system. This could result in marine macroalgae being less sensitive to climate change than estuarine macroalgae.

Data Quality & Gaps: The data quality scores for two of the five climate exposure factors (Sea Surface Temperature and Surface Salinity) were scored relatively High (3.0 and 2.8, respectively). The low data quality score for pH (2.0) was likely attributed to the low resolution of CMIP5 projections for nearshore, shallow areas. SLR and Precipitation also scored Low (2.2 and 2.4, respectively), but this may be attributed to the uncertainty of sea level rise exposure and the influence of precipitation over the wide vertical distribution of macroalgae.

For habitat sensitivity, all of the attributes were scored relatively Low (2.2 or 2.0). Much of the Low data quality scoring for sensitivity may be attributed to the abundant and diverse taxa and ecology of this sub-class. Algal species are found throughout the intertidal zone to the subtidal photic zone limits across the entire study area. In addition, a number of species are non-indigenous and appear to be tolerant of warmer water, and may flourish with projected future climate conditions.

Positive or Negative Climate Effect for the Northeast U.S.: The expected effect of climate change on marine red, green, and small brown algae in the Northeast U.S. is evenly split between negative (40%) and neutral (45%), with 15% experts assigning a positive score.

Climate Effects on Habitat Condition and Distribution: Using a species distribution model, Wilson et al. (2019) projected climate-induced shifts in dominance from native canopy-forming macroalgae to turf-algae by the end of the century. Turf algae generally refers to low-lying (<10 cm tall) species with densely packed fronds, lax and filamentous branches (e.g., *Cladophora, Ulva, and Polysiphonia*) (Connell et al. 2014). Primarily driven by increased sea surface temperature, the model projected northern range shifts for rockweed *A. nodosum*, the fucoid algae *Fucus vesiculosus,* Irish moss *C. crispus*, and the invasive green algae *Codium fragile*. The current southern range extent for *A. nodosum* and *C. crispus* is Long Island Sound, while *F. vesiculosus* and *C. fragile* occur throughout the entire study area. Wilson et al. (2019) projected the trailing edge of *F. vesiculosus, A. nodosum,* and *C. crispus* will shift northward under RCP8.5. The model projection for *A. nodosum* effectively results in the species to be extirpated from the entire study area, and the trailing edge for *C. crispus* and *F. vesiculosus* would no longer occur in waters south of the Gulf of Maine and Long Island, respectively. However, the overall habitat of all three species would expand into waters north of the Gulf of Maine. Interestingly, the species distribution model projected no shift in the trailing edge of the invasive *C. fragile*, and minimal northward range expansion, and an overall habitat contraction of 2% (Wilson et al. 2019).

Macroalgal species that occur in the intertidal zone are also sensitive to air temperature and desiccation. Although changes in air temperature were not evaluated in the climate exposure scoring for this sub-class, the projected change compared to historic periods under RCP8.5 was very high. Increasing air temperature may result in vertical distribution shifts in intertidal macroalgae to minimize thermal or desiccation stress during low tide (Harley et al. 2012).

Sea level rise exposure for this subclass is Moderate to High, although the effect of rising sea levels for macroalgal species is dependent upon their vertical distribution in the water column. In this regard, species in the intertidal and the shallowest subtidal zones will be most affected by changes in sea level. Because macroalgae require some minimal threshold of light for photosynthesis, they are expected to shift distribution landward to maintain exposure to sunlight (Steneck et al. 2002). However, the availability of suitable substrate, and both natural and artificial barriers may restrict landward migration of macroalgae.

Storms are expected to increase in frequency and intensity in the Northeast region (USGCRP 2017), and increases in wave energy exposure could have deleterious effects on intertidal and shallow-water habitats. Based on the results of CMIP5 RCP8.5, Lehmann et al. (2014) concluded that the northeast region would likely experience an increase in the frequency and intensity of winter extratropical cyclone events, but a slight decrease in summer events, by 2100. Colle et al. (2013) projected extratropical cyclones may become more intense (10-40%) along the northeast coast, especially during the mid-21st century as a result of an increase in latent heat release due to a moister atmosphere. The northeast region is also affected by tropical cyclone systems that originate in the Atlantic and Caribbean basins. Increases in sea surface temperatures may increase the maximum potential intensity of tropical cyclones and should be reflected by an increase in the frequency of the strongest hurricanes (Trenberth 2005; Kossin et al. 2007; Knutson et al. 2010). Between the 1970s and early 2000s, the number of major hurricanes (Category 4 and 5) in an average year approximately doubled (Emanuel 2005; Webster et al. 2005).

The projected change in pH for marine macroalgae species in the study area is very high. The majority of species in the study area are fleshy, non-calcifying algae, and are believed to have low sensitivities to pH and carbonate chemistry (Koch et al. 2013; Kroeker et al. 2013). However, coralline red algae and calcifying green algae occur in the study area, and some species have shown reduced calcification rates inhigh-CO_2 mesocosm experiments (Gao et al. 1993; Kroeker et al. 2013). This group of algal species are discussed under the marine and estuarine rocky bottom habitat sub-classes. Some marine macroalgae have shown higher growth rates under experimental elevated pCO_2 conditions through higher photosynthetic and growth rates (Gledhill et al. 2015; Young and Gobler 2016), which could mitigate some climate-related impacts associated with warming waters.

Habitat Summary: The effects of warming ocean waters and other environmental changes will likely result in “winners and losers” for this habitat subclass. Many species of macroalgae can tolerate temperature gradients of several degrees and display ecotypes that are capable of growing and reproducing over a wide range of physical and chemical conditions. Some of these species are characterized as turf algae, including *Cladophora, Ulva, and Polysiphonia* (Connell et al. 2014).

Ecosystem changes observed throughout the study area (e.g., warming waters, increasing intensity, frequency, and duration of coastal storms, increasing prevalence of invasive species and herbivory, and exposure to stormwater pollution) have been attributed to patterns of long-term shifts from kelp- to turf-dominated habitats in New England and the Gulf of Maine (Steneck et al. 2002; Dijkstra et al. 2017a; Dijkstra et al. 2017b; ; Filbee-Dexter & Wernberg 2018; Dijkstra et al. 2019; Feehan et al. 2019).

Increased reproduction of marine invasive species, combined with limited biological resistance in regions with cooler water temperatures, may lead to community state changes in shallow habitats in the Gulf of Maine (Dijkstra et al. 2017a). Warming waters have been shown to eliminate thermal barriers that historically limit reproductive success of marine invasive species (e.g., the tunicate *Botrylloides violaceus*), which may impact native macroalgal abundance and distribution (Dijkstra et al. 2017a).

Marine invasive species, likely introduced via maritime transport vectors, are known to compete for space and foul benthic substrates (Pappal 2010) and may replace native macroalgae species in portions of the study area (Scheibling and Gagnon 2006; Trott and Entreline 2019).

Although commercial and recreational harvest and culturing of rockweed occurs in Maine, there is little information available suggesting widespread impacts associated with controlled harvesting. However, at least one study (Wilson et al. 2019) projects the rockweed *A. nodosum* will be extirpated from U.S. waters by 2100 under the RCP8.5 scenario, which suggests future harvests of this species may be problematic.

Literature Cited

Colle BA, Zhang Z, Lombardo KA, Chang E, Liu P, Zhang M. 2013. Historical evaluation and future prediction of eastern North American and western Atlantic extratropical cyclones in the CMIP5 models during the cool season. Journal of Climate 26(18):6882-903.

Connell SD, Foster MS, Airoldi L. 2014. What are algal turfs? Towards a better description of turfs. Marine Ecology Progress Series 495:299–307.

Dijkstra JA, Westerman EL, Harris LG. 2017a. Elevated seasonal temperatures eliminate thermal barriers of reproduction of a dominant invasive species: A community state change for northern communities? Diversity and Distributions 23:1182–92.

Dijkstra JA, Harris LG, Mello K, Litterer A, Wells C, Ware C. 2017b. Invasive seaweeds transform habitat structure and increase biodiversity of associated species. Journal of Ecology 105: 1668-78. https://doi.org/10.1111/1365-2745.12775.

Dijkstra JA, Litterer A, Mello K, O'Brien BS, Rzhanov Y. 2019. Temperature, phenology, and turf macroalgae drive seascape change: Connections to mid-trophic level species. Ecosphere 10(11):e02923. https://doi.org/10.1002/ecs2.2923.

Emanuel K. 2005. Increasing destructiveness of tropical cyclones over the past 30 years. Nature 436(7051):686-8.

Feehan CJ, Grace SP, Narvaez CA. 2019. Ecological feedbacks stabilize a turf-dominated ecosystem at the southern extent of kelp forests in the Northwest Atlantic. Scientific Reports 9:7078. https://doi.org/10.1038/s41598-019-43536-5.

Filbee-Dexter K, and Wernberg T. 2018. Rise of turfs: a new battlefront for globally declining kelp forests. Bio-Science 68:64–76. https://doi.org/10.1093/biosci/bix147.

Gao K, Aruga Y, Asada K, Ishihara T, Akano T, Kiyohara M. 1993. Calcification in the articulated coralline alga *Corallina pilulifera*, with special reference to the effect of elevated CO_2 concentration. Marine Biology 117(1):129-32.

Gledhill DK, White MM, Salisbury J, Thomas H, Misna I, Liebman M, Mook B, Grear J et al. 2015. Ocean and coastal acidification off New England and Nova Scotia. Oceanography 28(2):182–97. http://dx.doi.org/10.5670/oceanog.2015.41.

Harley CDG, Anderson KM, Demes KW, Jorve JP, Kordas RL, Coyle TA, Graham MH. 2012. Effects of climate change on global seaweed communities. Journal of Phycology 48:1064–78. https://doi.org/10.1111/j.1529-8817.2012.01224.x.

Knutson TR, McBride JL, Chan J, Emanuel K, Holland G, Landsea C, Held I, Kossin JP, Srivastava AK, Sugi M. 2010. Tropical cyclones and climate change. Nature Geoscience 3(3):157-63.

Koch M, Bowes G, Ross C, Zhang X-H. 2013. Climate change and ocean acidification effects on seagrasses and marine macroalgae. Global Change Biology 19(1):103-32.

Kossin JP, Knapp KR, Vimont DJ, Murnane RJ, Harper BA. 2007. A globally consistent reanalysis of hurricane variability and trends. Geophysical Research Letters 34(4):1-6.

Kroeker KJ, Kordas RL, Crim R, Hendriks IE, Ramajo L, Singh GS, Duarte CM, Gattuso J-P. 2013. Impacts of ocean acidification on marine organisms: quantifying sensitivities and interaction with warming. Global Change Biology 19(6):1884-96.

Lehmann J, Coumou D, Frieler K, Eliseev AV, Levermann A. 2014. Future changes in extratropical storm tracks and baroclinicity under climate change. Environmental Research Letters 9:8.

Mathieson AC, Pederson JR, Neefus CD, Dawes CJ, Bray TL. 2008. Multiple assessments of introduced seaweeds in the Northwest Atlantic. ICES Journal of Marine Science 65: 730–41.

Mathieson AC, Dawes CJ, Hehre EJ, Harris LG. 2010. Floristic studies of seaweeds from Cobscook Bay, Maine. Northeastern Naturalist 16 (Monograph 5):1–48.

Orris PK. 1980. Revised species list and commentary on the macroalgae of the Chesapeake Bay in Maryland. Estuaries 3(3):200-6.

Pappal A. 2010. Marine invasive species. State of the Gulf of Maine Report. [Currier P, Gould D, Hertz L, Huston J, Tremblay ML (eds)]. The Gulf of Maine Council on the Marine Environment. 1-21.

Scheibling RE, Gagnon P. 2006. Competitive interactions between the invasive green alga *Codium fragile* ssp. *tomentosoides* and native canopy-forming seaweeds in Nova Scotia (Canada). Marine Ecology Progress Series 325:1–14. doi:10.3354/meps325001.

Steneck R, Graham MH, Bourque BJ, Corbett D, Erlandson JM, Estes JA, Tegner MJ. 2002. Kelp forest ecosystems: biodiversity, stability, resilience and future. Marine Sciences Faculty Scholarship. 65. https://digitalcommons.library.umaine.edu/sms_facpub/65.

Trenberth K. 2005. Uncertainty in hurricanes and global warming. Science 308(5729):1753-4.

Trott TJ, Enterline C. 2019. First record of the encrusting bryozoan *Cribrilina* (*Juxtacribrilina*) *mutabilis* (Ito, Onishi and Dick, 2015) in the Northwest Atlantic Ocean. BioInvasions Records 8.

USGCRP. 2017. Climate Science Special Report: Fourth National Climate Assessment, Volume I [Wuebbles, D.J., D.W. Fahey, K.A. Hibbard, D.J. Dokken, B.C. Stewart, and T.K. Maycock (eds.)]. U.S. Global Change Research Program. Washington, DC, USA. 470 pp. doi: 10.7930/J0J964J6.

Van Patten MS, Yarish C. 2009. Seaweeds of Long Island Sound. Bulletin 39. Connecticut College Arboretum.

Webster PJ, Holland GJ, Curry JA, Chang H-R. 2005. Changes in tropical cyclone number, duration, and intensity in a warming environment. Science 309(5742):1844-6.

Wilson KL, Skinner MA, Lotze HK. 2019. Projected 21st-century distribution of canopy-forming seaweeds in the Northwest Atlantic with climate change. Diversity and Distributions 25:582–602. https://doi.org/10.1111/ddi.12897.

Witman JD, Lamb RW. 2018. Persistent differences between coastal and offshore kelp forest communities in a warming Gulf of Maine. PLoS ONE 13(1):e0189388. https://doi.org/.10.1371/journal.pone.0189388.

Young CS, Gobler CJ. 2016. Ocean acidification accelerates the growth of two bloom-forming macroalgae. PLoS ONE 11(5):e0155152. doi:10.1371/journal.pone.0155152

Marine Submerged Aquatic Vegetation

System: Marine

Subsystem: Subtidal & Intertidal

Class: Aquatic Bed

Sub-class: Rooted Vascular

Geographic Area: Entire Area

Overall Vulnerability Rank = High

Habitat Sensitivity = High

Climate Exposure = High

	**Marine Submerged Aquatic Vegetation**	Attribute Mean	Data Quality	Distribution of Expert Scores
Sensitivity Attributes	Habitat condition	3.2	2.6	
	Habitat fragmentation	3.4	2	
	Distribution/Range	3.4	2.4	
	Mobility/Ability to spread or disperse	2.7	2.4	
	Resistance	3.1	2.4	
	Resilience	2.9	2.4	
	Sensitivity to changes in abiotic factors	2.8	2.4	
	Sensitivity and intensity of non−climate stressors	3.8	2.6	
	Dependency on critical ecological linkages	2.6	2	
	Sensitivity Component Score	High		
Exposure Factors	Sea surface temp	3.7	3	
	Bottom temp	n/a	n/a	
	Air temp	n/a	n/a	
	River temp	n/a	n/a	
	Surface salinity	1.2	2.8	
	Bottom salinity	n/a	n/a	
	pH	4	2	
	Sea level rise	2.8	2.2	
	Precipitation	2.5	2.1	
	Floods	n/a	n/a	
	Droughts	n/a	n/a	
	Exposure Component Score	High		
**Overall Vulnerability Rank**		**High**		

Low
Moderate
High
Very High

Habitat Name: Marine Submerged Aquatic Vegetation

System: Marine

Sub-System: Subtidal and Intertidal

Class: Aquatic Bed

Sub-class: Rooted Vascular

Geographic Area: Entire Area

Habitat Description: This sub-class includes rooted vascular beds occurring in the marine system of the study area in the subtidal and intertidal zone and full salinity waters (>30 ppt). Eelgrass (*Zostera marina*)is the dominant rooted vascular plant found in the marine environment over its western Atlantic range from North Carolina to Canada (Thayer et al. 1984). Widgeon grass (*Ruppia maritima*) can be found in marine waters in discrete meadows or intermixed with eelgrass (Kantrud 1991). *Ruppia* has a broad geographic range spanning Florida to Canada (Kantrud 1991). Both species require sediments that allow for root penetration, thus sand and silt are the most common. However, eelgrass can colonize areas of gravel with underlying sand and in one instance was observed growing on a section of the seafloor that was Boston blue clay (Colarusso, pers obs). Due to the high light requirements for both species, they are generally restricted to shallow coastal waters (Thayer et al. 1984; Kantrud 1991). Eelgrass has been observed rooted at 44 feet mean low water offshore of Rhode Island (Short, pers. comm.), but 25 feet mean low water is generally a maximum depth for New England (Colarusso, pers. obs.). Widgeon grass grows in shallow water with maximum depth limits of less than 10 feet mean low water (Kantrud 1991). Both species can persist in the intertidal, but only at higher latitudes (generally Maine/NH border northward) due to their sensitivity to desiccation (Thayer et al. 1984; Kantrud 1991).

Overall Climate Vulnerability Rank: High (94% certainty from the bootstrap analysis). Although the majority of the bootstrap results match the results of the categorical vulnerability rank, 6% of the bootstrap results were in the Very High vulnerability rank.

Climate Exposure: High. The overall high exposure score was influenced by two Very High attribute means: Sea Surface Temperature (3.7) and pH (4.0). Sea Level Rise (SLR) and Precipitation also received High scores (2.8, 2.5 respectively).

Habitat Sensitivity: High. All nine sensitivity attributes received High and Very High individual scores. Five of the nine sensitivity attributes means were >3.0, while the other attributes had scores between 2.6 and 2.9. The highest sensitivity attribute mean was for Sensitivity and Intensity of Non-Climate Stressors (3.8). Habitat Condition (3.2), Habitat Fragmentation (3.4), Distribution/Range (3.4) and Resistance (3.1) all scored above 3.0.

Data Quality & Gaps: The data quality scores for three of the five climate exposure factors (pH, Precipitation and SLR) were scored relatively Low ($\leq$2.2). Data quality is likely lower for marinerooted vascular beds due to the low resolution of CMIP5 and ROMS-NWA projections for climate exposure in estuaries and nearshore, shallow coastal areas.

For habitat sensitivity, only two of the nine attributes had relatively Low (2.0) data quality scores (Habitat Fragmentation and Dependency on Critical Ecological Linkages). The rest were 2.4 and 2.6, which likely reflects the literature and the moderate understanding of subtidal, rooted vascular plant responses to non-climate and climate stressors.

Positive or Negative Climate Effect in the Northeast U.S. Shelf: The effect of climate change on marine rooted vascular beds in the Northeast U.S. Shelf is expected to be negative (90% of the experts' scores were negative, 10% were neutral).

Climate Effects on Habitat Condition and Distribution: Eelgrass, the dominant rooted vascular bed species, is considered a "cold" water plant because when light is not a limiting factor it does best

metabolically at 5ºC (Marsh et al. 1986). In the absence of light limitation, it shows positive metabolic growth up to 25ºC, at which point carbon loss due to respiration equals carbon gain from photosynthesis (Marsh et al. 1986). At 25ºC, eelgrass growth is reduced (Kaldy 2014; Thom et al. 2014) and at temperatures above 23ºC, primary production is reduced (Moore et al. 1996). Plants can persist above 25ºC for a while persisting on carbon reserves, but extended time above this temperature risks depletingimportant reserves needed to overwinter.

Increases in water temperature may impact the normal timing of flowering and seed production in both eelgrass and widgeon grass (Short and Neckles 1999). Increases in water temperature as small as 1ºC has been shown to advance flower formation in eelgrass by 12 days and seedling maturation by 10.8 days (Blok et al. 2018). It is unclear what changes in the timing of the normal reproductive cycle may mean for the long-term survival of individual meadows.

Increased water temperatures have the potential to reduce the existing distribution and productivity of eelgrass over its existing range (Moore et al. 1996; Short and Neckles 1999). Widgeon grass is unlikely to be negatively affected by increasing water temperature along the Atlantic coast, due to its higher temperature tolerance (Kantrud 1991). As water temperatures increase, it is likely that widgeon grass distribution will actually increase, by replacing eelgrass meadows in the southern portion of eelgrass' current distribution (Moore et al. 2014). For most of its range, eelgrass actively grows from spring through fall. At the southern edge of its range, eelgrass grows from fall through spring, disappearing in the summer (Thayer et al. 1984; Short and Neckles 1999). As sea surface temperature increases, it is likely this adaptation in the growing season will move northward (Short and Neckles 1999).

Increased water temperature may also lead to greater survival and distribution of invasive species that have been shown to have negative impacts to eelgrass (Neckles 2015; Carman et al. 2019; Young and Elliot 2020). Warmer winter temperatures have led to greater green crab overwinter survival (Young and Elliott 2020) and green crabs have been shown to cause the decline of hundreds of acres of eelgrass in Maine and Canada (Neckles 2015). Invasive tunicates also have the potential to lead to eelgrass shoot mortality (Wong and Vercaemer 2012). Latitudinal changes in invasive tunicates distribution on eelgrass have been documented and changes in water temperature are likely a contributing factor (Carman et al. 2016; Carman et al. 2019).

Eelgrass has relatively high light requirements for its survival and it has been shown that small changes in water clarity can result in dramatic reductions in eelgrass production and survival (Short and Wyllie-Echeverria 1996; Bertelli and Unsworth 2018). Increased frequency and volume of rainfall due to climate change will result in diminished water clarity in many near shore environments. This will be particularly acute in urban areas and at the mouths of freshwater inputs. Eelgrass meadows that experience light limitation will exhibit this in two ways: the deep edge of the meadow will contract into shallower water, thus reducing the acreage of the habitat, and the main body of the meadow will also thin out, as reductions in shoot density reduces self-shading (Ralph et al. 2007). Reducing shoot density of a meadow also reduces the meadow's ecological functions. Sparser meadows are less valuable fish habitat, sequester less carbon, and are not as prolific primary producers (Novak et al. 2020). Widgeon grass tends to grow in shallower water than eelgrass, so light limitation is not as critical for this species. It is unlikelythat changes in water clarity will significantly impact widgeon grass.

Increases in the frequency and magnitude of large-scale storm events can have significant impacts to both of these species. In May, 2006, the "Mother's Day" storm delivered over 20 inches of rainfall to southern New Hampshire and the Great Bay watershed. This large infusion of freshwater resulted in salinities dropping in the bay to almost 0 ppt. for several weeks. Mapping of eelgrass in 2006 showed a greater than 1,000 acre reduction compared to levels in 2005 and a decline in widgeon grass as well (Short 2008; PREP 2018). Both eelgrass and widgeon grass are nearshore shallow water plants that are susceptible to impacts from storm generated waves. Significant sections of meadows of both of these species can be uprooted particularly if coastal storm generated waves coincide with a negative low tide. Estuarine subtidal vascular bed habitats may

experience greater disturbance from storms due to climate change, which are expected to increase in frequency and intensity in the Northeast region (USGCRP 2017).

None of the potential impacts of climate change occur in isolation. Reduced light penetration, due to enhanced storm activity and sea level rise, will have significant negative impacts to both eelgrass and widgeon grass distribution and productivity (Kantrud 1991; Short and Neckles 1999). For eelgrass in particular, small changes in light, co-occurring with elevated water temperatures can trigger mortality (Moore et al. 2012). Large scale die-offs of eelgrass have been observed at the southern end of its range in response to seemingly small temperature increases combined with small light reductions. Meadows that perished experienced water temperatures 1°C warmer and light reduced by 30% compared to meadows that survived (Moore et al 2012).

Eelgrass resilience in the face of climate change can be thought of in multiple ways. Meadows with higher genetic diversity have proven more resilient to extended heat waves (Dubois et al. 2019). Eelgrass meadows have some ability to recover from anthropogenic stress, provided that water clarity and sediment conditions remain conducive to eelgrass growth (Neckles et al. 2005).

Habitat Summary: This habitat exists in shallow water in close proximity to many anthropogenic stressors. Given the projected changes in climate and increasing anthropogenic activity in the near shore coastal zone, the abundance and condition of this habitat is expected to decline. Areas of greatest decline are in close proximity to large population centers and waters from Cape Cod south. Some meadows periodically become carbon limited, reducing their growth and production. Elevated CO_2 concentrations could reduce these periods of carbon limitation and enhance seagrass growth and production (Alexandre et al. 2012).

Marine rooted vascular plants are experiencing a global decline. It has been estimated that 110 km^2/yr of seagrass is being lost and 29% of the seagrass initially recorded in the late 1800s is now gone (Waycott et al. 2009). Since 1990 the rate of the global losses is accelerating and is estimated to be occurring at about 7% per year (Waycott et al. 2009).

Marine rooted vascular plants are at high risk for anthropogenic impacts due to their preference for shallow coastal waters and their limited resistance to many stressors. Relatively small declines in water quality, usually due to nutrient over-enrichment, can lead to relatively large reductions in plant resilience,productivity and abundance (Short and Wyllie-Echeverria 1996). Physical stressors, such as dredging, filling, scouring from moorings and cutting from propellers can impact rooted vascular plant distribution (Short and Wyllie-Echeverria 1996). Some fragmentation occurs naturally within seagrass meadows, but anthropogenic activities, especially the physical stressors can create holes and patchiness in meadows. The creation of holes and patchiness in meadows increases the amount of edge habitat, which increases the risk of erosion and reduces its value as a fish habitat.

Climate change exacerbates the levels of anthropogenic stress rooted vascular plants are already experiencing. Their preferred habitat of shallow coastal waters makes them particularly susceptible to the effects of climate change. For example, shallow waters are more susceptible to temperature changes compared to deeper ocean waters. Proximity to the shoreline puts these habitats at greater risk of experiencing increased levels of turbidity from larger and more frequent precipitation events.

Additionally, sea level rise will contribute to reduced quantities of light reaching these meadows, forcing a landward migration into shallower water where that may be possible. Shoreline armoring can impede landward movement of seagrass beds as sea levels rise (Short and Neckles 1999; Orth et al. 2017). Shoreline structures can deflect wave energy and cause increased turbulence and scouring of sediment and vegetation along their waterward edge, and increase suspended sediments and turbidity (Williams and Thom 2001).

Literature Cited

Alexandre A, Silva J, Buapet, P, Bjork M, Santos R. 2012. Effects of CO_2 enrichment on photosynthesis, growth and nitrogen metabolism of the seagrass *Zostera noltii*. Ecology and Evolution 2(10):2625-35.

Bertelli CM, Unsworth RKF. 2018. Light stress responses by the eelgrass, *Zostera marina* (L.). Frontiers of Environmental Science 6:39 https://doi.org/10.3389/fenvs.2018.00039.

Blok SE, Olesen B, Krause-Jensen D. 2018. Life history events of eelgrass *Zostera marina* L. populations across gradients of latitude and temperature. Marine Ecology Progress Series 590:70-93. https://doi.org/10.3354/meps12479.

Carman MR, Colarusso PD, Nelson EP, Grunden DW, Wong MC, McKenzie C, Matheson K, Davidson J, Fox S, Neckles HA, Bayley H, Schott S, Dijkstra JA, Stewart-Clark S. 2016. Distribution and diversity of tunicates utilizing eelgrass as substrate in the western North Atlantic between 39º and 47º north latitude (New Jersey to Newfoundland). Management of Biological Invasions 7(1):51-7.

Carman MR, Colarusso PD, Neckles HA, Bologna P, Caines S, Davidson JDP, Evans, NT, Fox SE, Grunden DW, Hoffman S, Ma KCK, Matheson K, McKenzie CH, Nelson EP, Plaisted H, Reddington E, Schott S, Wong MC. 2019. Biogeographical patterns of tunicates utilizing eelgrass as a substrate in the western North Atlantic between 39º and 47º north latitude (New Jersey to Newfoundland). Management of Biological Invasions 10(4):602-16.

Dubois K, Abbott JM, Williams SL, Stachowicz JJ. 2019. Relative performance of eelgrass genotypes shifts during an extreme warming event: disentangling the roles of multiple traits. Marine Ecology Progress Series 615:67-77. https://doi.ord/10.3354/meps12914

Kaldy JE. 2014. Effect of temperature and nutrient manipulations on eelgrass *Zostera marina* L. from the Pacific Northwest, USA. Journal of Experimental Marine Biology and Ecology 453:108-15. https://doi.org/10.1016/j.jembe.2013.12.0202

Kantrud HA. 1991. Widgeongrass (*Ruppia maritima* L.): a literature review. U.S. Fish and Wildlife Service. Fish and Wildlife Research 10. 58 pp.

Marsh JA, Dennison WC, Alberte RS. 1986. Effects of temperature on photosynthesis and respiration in eelgrass (*Zostera marina* L.). Journal of Experimental Marine Biology and Ecology 101:257-67.

Moore KA, Neckles HA, Orth RJ. 1996. *Zostera marina* (eelgrass) growth and survival along a gradient of nutrients and turbidity in the lower Chesapeake Bay. Marine Ecology Progress Series 142(1-3):247-59.

Moore KA, Shields EC, Parrish DB, Orth RJ. 2012. Eelgrass survival in two contrasting systems: role of turbidity and summer water temperatures. Marine Ecology Progress Series 448:247-58. https://doi:103354/meps09578.

Moore KA, Shields EC, Parrish DB. 2014. Impacts of varying estuarine temperature and light conditions on *Zostera marina* (eelgrass) and its interactions with *Ruppia maritima* (widgeongrass). Estuaries and Coasts 37(1):20-30.

Neckles HA. 2015. Loss of eelgrass in Casco Bay, Maine, linked to green crab disturbance. Northeastern Naturalist 22(3):478-500.

Neckles HA, Short FT, Barker S, Kopp BS. 2005. Disturbance of eelgrass *Zostera marina* by commercial

mussel *Mytilus edulis* harvesting in Maine: dragging impacts and habitat recovery. Marine Ecology Progress Series 285:57-73.

Novak AB, Pelletier MC, Colarusso P, Simpson J, Gutierrez MN, Arias-Ortiz A, Charpentier M, Masque P, Vella P. 2020. Factors influencing carbon stocks and accumulation rates in eelgrass meadows across New England, USA. Estuaries and Coasts 43:2076–91. https://doi.org/10.1007/s12237-020-00754-9.

Orth RJ, Dennison WC, Lefcheck JS, Gurbisz C, Hannam M, Keisman J, Landry JB, Moore KA, Murphy RR, Patrick CJ, Testa J, Weller DE, Wilcox DJ. 2017. Submersed aquatic vegetation in Chesapeake Bay: sentinel species in a changing world. BioScience 67(8):698-712.

Piscataqua Region Estuaries Partnership (PREP). 2018. [Internet]. State of Our Estuaries Report. 52 pp. (Accessed 8 Dec. 2020). https://prepestuaries.org/resources/2018-sooe-report/

Ralph P, Durako MJ, Enriquez S, Collier CJ, Doblin M. 2007. Impact of light limitation on seagrasses. Journal of Experimental Marine Biology and Ecology 350(1-2):176-93.

Short FT. 2008. [Internet]. Eelgrass distribution in the Great Bay estuary. PREP reports and publications. 86. (Accessed 8 Dec. 2020). https://scholars.unh.edu/prep/86.

Short FT, Neckles HA. 1999. The effects of global climate change on seagrasses. Aquatic Botany 63:169-96.

Short FT, Wyllie-Echeverria S. 1996. Natural and human-induced disturbance of seagrasses. Environmental Conservation 23(1):17-27.

Thayer GW, Kenworthy WJ, Fonseca MS. 1984. The ecology of eelgrass meadows of the Atlantic coast: a community profile. U.S. Fish Wildlife Service FWS/OBS-84/02. 147 pp.

Thom R, Southard S, Borde A. 2014. Climate-linked mechanisms driving spatial and temporal variation in eelgrass (*Zostera marina* L.) growth and assemblage structure in Pacific Northwest estuaries, U.S.A. Journal of Coastal Research Special Issue (68):1-11.

U.S. Global Change Research Program (USGCRP). 2017. Climate Science Special Report: Fourth National Climate Assessment, Volume I [Wuebbles, D.J., D.W. Fahey, K.A. Hibbard, D.J. Dokken, B.C. Stewart, and T.K. Maycock (eds.)]. U.S. Global Change Research Program, Washington, DC, USA. 470 pp. doi:10.7930/J0J964J6.

Waycott M, Duarte CM, Carruthers TBJ, Orth RJ, Dennison WC, Olyarnik S, Calladine A, Fourqurean JW, Heck jr KL, Hughes AR, Kendrick GA, Kenworthy WJ, Short FT, Williams SL. 2009. Accelerating loss of seagrass across the globe threatens coastal ecosystems. Proceedings of the National Academy of Sciences of the United States of America 106(30):12377-81. https://doi.org/10.1073/pnas.0905620106.

Wong MC, Vercaemer, B. 2012. Effects of invasive tunicates and a native sponge on the growth, survival, and light attenuation of eelgrass (*Zostera marina*). Aquatic invasions 7(3):315-26.

Young AM, Elliott JA. 2020. Life history and population dynamics of green crabs (*Carcinus maenas*). Fishes 5(1):4. https://doi10:3390/fishes5010004.

Marine Subtidal Shellfish Reef

System: Marine

Subsystem: Subtidal

Class: Reef

Sub-class: Mollusk

Geographic Area: Entire Area

Overall Vulnerability Rank = High

Habitat Sensitivity = High

Climate Exposure = High

	Marine Subtidal Shellfish Reef	Attribute Mean	Data Quality	Distribution of Expert Scores
Sensitivity Attributes	Habitat condition	2.8	1.8	
	Habitat fragmentation	3	1.8	
	Distribution/Range	2.3	2	
	Mobility/Ability to spread or disperse	2.2	1.9	
	Resistance	2.4	1.9	
	Resilience	2.8	2.2	
	Sensitivity to changes in abiotic factors	3.7	2.3	
	Sensitivity and intensity of non−climate stressors	3.8	2.4	
	Dependency on critical ecological linkages	2.7	1.6	
	Sensitivity Component Score	High		
Exposure Factors	Sea surface temp	3.8	2.8	
	Bottom temp	n/a	n/a	
	Air temp	n/a	n/a	
	River temp	n/a	n/a	
	Surface salinity	1.3	2.6	
	Bottom salinity	n/a	n/a	
	pH	4	2.2	
	Sea level rise	2.4	2.2	
	Precipitation	2.4	2.1	
	Floods	n/a	n/a	
	Droughts	n/a	n/a	
	Exposure Component Score	High		
**Overall Vulnerability Rank**		**High**		

Habitat Name: Marine Subtidal Shellfish Reef

System**:** Marine

Subsystem: Subtidal

Class: Reef

Sub-class: Mollusk

Geographic Area: Entire Area

Habitat Description: This sub-class includes subtidal, reef-building shellfish that create a biotic hard substrate on benthic substrates in marine waters >30 ppt. The most common species in the study area are blue mussels (*Mytilus edulis*) and eastern oyster (*Crassostrea virginica*). Bivalves like oysters and mussels grow in dense aggregations forming reefs that extend off the seafloor. Oysters range from the Gulf of St. Lawrence, Canada to the Yucatan, West Indies and Brazil (Gunter 1951). Suitable substrates consist of sand, firm mud, or clay, whereas shifting sand and extremely soft mud are thought to be the only unsuitable substrates for oyster reef habitats (Galstoff 1964; Bahr and Lanier 1981). The blue musselis a cosmopolitan species common to temperate and polar waters in the northern Atlantic Ocean from the southern Canadian Maritime provinces to North Carolina. Shellfish reefs are common in the intertidal andsubtidal down to 10m.

Shellfish reefs serve as habitat for diverse assemblages of polychaetes, crustaceans, and other resident invertebrate and fish species (Wells 1961; Bahr and Lanier 1981; Rothschild et al 1994; Coen et al. 1999; Peterson et al. 2003; zu Ermgassen et al. 2016). They are filter feeders, thereby promoting greater water clarity and benthic productivity (Dame et a. 1984; Newell 1988; Ulanowicz and Tuttle 1992; Paerl et al. 1998). Shellfish also remove excess nitrogen from coastal estuaries by promoting bacterially mediated denitrification as a consequence of concentrating bottom deposits of feces and pseudofeces (Newell et al. 2002; Piehler and Smyth 2011).

Overall Climate Vulnerability Rank: High (99.9% certainty from bootstrap analysis).

Climate Exposure: High. The overall high exposure score was influenced by the following Very High attribute means: pH (4.0) and Sea Surface Temperature (3.8). The exposure attribute score for Precipitation (2.4), Sea Level Rise (SLR) (2.4), and Surface Salinity (1.3) were Low to Moderate. SLR is expected to impact the entire study area but the greatest relative rise is projected in the Mid-Atlantic.

Habitat Sensitivity: High. Six of the nine sensitivity attribute means were ≥2.7: Habitat Condition (2.8), Habitat Fragmentation (3.0), Resilience (2.8), Sensitivity to Changes in Abiotic Factors (3.7), Sensitivity and Intensity of Non-Climate Stressors (3.8), and Dependency on Critical Ecological Linkages (2.7). The attribute means for Mobility/Ability to Spread or Disperse (2.2), Resistance (2.4) and Distribution range (2.3) were Moderate.

Data Quality & Gaps: Data quality for two of the climate exposure factors were relatively Moderate to High: Sea Surface Temperature (2.8) and Surface Salinity (2.6). Data quality for SLR (2.2), pH (2.2) and Precipitation (2.1) were lower. The relatively Low score for Precipitation is due to uncertainty inthe specific spatial nature of projected changes in extreme events. In addition, data quality is lower for nearshore coastal habitats due to the low resolution of CMIP5 and ROMS-NWA projections for climate exposure in estuaries and nearshore, shallow coastal areas. Another uncertainty factor is the relative disparate spatial data for mollusk reefs in some portions of the study area.

For habitat sensitivity attributes, data quality for all of the nine attributes were scored between 1.6 and 2.4, with Habitat Condition (1.8), Habitat Fragmentation (1.8), Mobility/Ability to Spread or Disperse (1.9), Resistance (1.9) and Dependency on Critical Ecological Linkages (1.6) under 2.0. Although the climate sensitivity for estuarine subtidal mollusk reefs are believed to be high, there remains some uncertainty in species' capacity to adapt and respond to climate change (e.g., synergistic effects of climate and non-climate stressors).

Relatively moderate to low data quality scores could reflect the fact that few comprehensive assessments for shellfish habitat exist in the study area as much of it was lost centuries ago (Kirby 2004; Rothschild et al.

1994; zu Ermgassen et al. 2012).

Positive or Negative Climate Effect for the Northeast U.S.: The effect of climate change on marine subtidal shellfish habitats in the Northeast U.S. is expected to be negative (95% of the experts' scores were negative, and 5% were neutral).

Climate Effects on Habitat Condition and Distribution: Several studies have examined the effects of climate and non-climate impacts on shellfish reefs. While overharvesting has historically been attributed to oyster reef habitat loss in the northeast and elsewhere, hypoxia, disease, predators, competition, and sedimentation have impeded recent restoration efforts (Rothschild et al. 1994; Kirby 2004; Beck et al. 2011; zu Ermgassen et al. 2012). In addition, there is growing evidence that suggests potential climate effects on shellfish reefs may also be a factor.

Increasing seawater temperatures may affect the condition and distribution of subtidal shellfish in nearshore marine waters. For example, southern populations of blue mussels appear to be shifting northward in response to warming temperatures (Jones et al. 2010). Thermal stress, in conjunction with ocean acidification conditions, was found to cause metabolic depression in blue mussels from the Gulf of Maine (Lesser 2016), suggesting that elevated temperatures may result in suboptimal conditions for this cold-adapted species. Oyster growth and reproductive rates peak in waters ranging in temperature from 20-30°C and can live in water temperatures of 0-36°C (Shumway 1996; Lenihan 1999). In comparison, mussels are common in colder, more temperate to polar waters, thriving in 5-20°C, with an upper thermaltolerance limit of 29°C (Animal Diversity Web 2020).

Although the ROMS-NWA projections limit the ability to make precise climate predictions on impacts to marine shellfish reefs, water temperatures are projected to increase compared to historic means. The ROMS-NWA projections for RCP8.5 indicate the standardized anomaly for sea surface temperature will increase by at least 4 standard deviations from the historic means, and higher in the Gulf of Maine. Therefore, there is a possibility the maximum temperature thresholds for one or more life stages of shellfish may be exceeded by the end of the century, especially during episodic heat waves (Jones et al.2009; Jones et al. 2010; Zippay and Helmuth 2012; Speights et al. 2017). Warming seawater can also increase the susceptibility of shellfish to disease, parasites and predation by local and invasive species (Smolowitz 2013; Burge et al. 2014).

Oyster performance peaks in salinities from 15 to 30 practical salinity units (psu), and they can withstand salinities of 0-40 psu. However, oysters tend to grow faster and be in better condition with less variation in salinity (Galtsoff 1964; Shumway 1996). Blue mussel is a euryhaline species, and can survive periodic fluctuations below 15 psu, although they do not thrive in low salinity conditions (Animal Diversity Web 2020). The ROMS-NWA climate projections for RCP8.5 indicate a decline in surface salinity for the U.S. continental shelf and the Gulf of Maine. Although the ROMS-NWA projections indicate salinities for estuarine waters in Long Island Sound, Delaware Bay, and Chesapeake Bay are projected to increase by at least 2 standard deviations from the historic means, it is unclear if shallow, nearshore marine waters may also increase in salinity.

Nearshore marine waters are generally more susceptible to acidification than oceanic waters because they are subject to more acid sources and are generally less buffered than oceanic waters (i.e., differences in the amount of dissolved inorganic carbon, dissolved and particulate organic carbon, total alkalinity and nutrients from riverine and estuarine sources) (Waldbusser et al. 2011; Ekstrom et al. 2015; Gledhill et al.2015). Ocean acidification will likely most directly negatively impact shellfish reefs, with larger negative effects on survival for larvae than adults (Kroeker et al. 2013). Ocean acidification is expected to reduce the shell size, thickness, calcification rates and growth, and survival of embryo and larval bivalves (Gazeau et al. 2013). Ries et al. (2009) exposed both mussels and oysters to low pH levels, and found that acidification negatively impacts oyster calcification rates whereas there was no relationship between pH and the calcification rate of blue mussels. Yet, increased water and air temperature in the northeastern U.S. is likely resulting in reduced physiological performance of mussels (Zippay and Helmuth 2012), potentially explaining why mussel beds are becoming less common in southern New England the western portions of the Gulf of Maine when temperatures have exceeded the thermal maxima for mussels. Dodd etal. (2015) exposed oysters and their

predators to increased acidification and found that acidification negatively affected oyster growth, but also reduced crab consumption of oysters. The synergistic effects of temperature, salinity, and pH on metabolism in marine mollusks may be greater than reduced pH alone. Some studies have shown additive and synergistic negative effects on bivalves and gastropods from oceanacidification and low dissolved oxygen (Kroeker et al. 2013; Gobler et al. 2014; Clark and Gobler 2016; Gobler and Bauman 2016; Griffith and Gobler 2017).

Shallow subtidal marine shellfish habitats may experience disturbance from storms, which are expected to increase in frequency and intensity in the Northeast region (USGCRP 2017). Coastal storms may increase physical/mechanical disturbance and stress to shellfish reef structure and associated organisms, and repeated disturbance may prohibit formation of robust three-dimensional complex structures.

Sea-level rise poses both direct and indirect threats to shallow subtidal habitats. Shellfish reefs generally have the capacity to grow vertically and landward to keep up with sea level rise (Rodriguez et al. 2014). However, because they exist across a wide geographical and environmental range and diverse landscape settings, they are influenced by different types and magnitudes of stress, which influence growth rates. Oysters and blue mussels are restricted to shallow waters, in part as a function of water flow that influences food availability and by the movement of drifting larval and juvenile stages (Lenihan 1999), soincreased rates of sea level rise could exceed the ability of subtidal shellfish reefs to maintain optimal water depths.

Habitat Summary: Shellfish reefs have been highly altered, with estimates in the U.S. suggesting that 68% of historic oyster reef extent and over 80% of the productivity of these habitats have been lost primarily due to overharvesting and destructive harvesting practices, but also as a consequence of dredge and fill activities disease, sedimentation, predators, and competition (zu Ermgassen et al. 2012).

Historically, overharvesting has been the largest threat to shellfish habitats, with 85% of oyster reefs lost worldwide (Beck et al. 2011), and similar impacts having occurred in the U.S. (zu Ermgassen et al. 2012). Kirby (2004) suggested that harvesting of oysters peaked and then the fisheries collapsed in the northeastern U.S. in coastal Massachusetts and Southern New England in the early 1800s. Efforts to conserve and rebuild shellfish reefs have been challenged by anthropogenic disturbances such as bottom water hypoxia, dredge and fill activity, shoreline hardening, diseases such as Dermo and MSX, sedimentation, predation, and competition (Rothschild et al. 1994; Kirby 2004; Beck et al. 2011; zu Ermgassen et al. 2012). High temperature and low salinity are known drivers for both MSX and Dermo (Burreson et al. 2000; Ford and Smolowitz 2007; Burge et al. 2014). Between 1990 and 1992, a dramatic range extension of Dermo disease was reported over a 500 km area in the northeastern United States fromDelaware Bay, New Jersey to Cape Cod Bay, Massachusetts (Ford and Smolowitz 2007). By 1995, Dermo was reported as far north as Maine (Burge et al. 2014).

In coastal New England, many shellfish reefs suffer from poor recruitment, motivating restoration practitioners to seed reefs with juvenile oysters set on dead oyster shells at oyster hatcheries and then transplanted on shallow reefs (personal observation). Unfortunately, many of the restored oyster reefs in coastal Rhode Island’s salt ponds have failed to increase natural recruitment (Grabowski and Hughes, unpublished data). Anecdotally, many of the mussel beds that were common historically in northeastern Massachusetts are no longer present (Grabowski, personal observation), possibly a consequence of local warming of sea and air temperatures (Zippay and Helmuth 2012).

Climate change will exacerbate other anthropogenic effects that have negatively impacted shellfish reefs. Oysters are thought to be among the most vulnerable of species to ocean acidification given its impacts on shell calcification rates and the protection that the shell provides from predators (Ries et al 2009). Although oyster reefs can grow relatively quickly and keep pace with sea-level rise, shoreline development in many regions may have removed available space for reefs to migrate landward (Ridge et al. 2015). While mussels may be less vulnerable to acidification compared to oysters (Ries et al. 2009), they are being crowded out of coastal waters in New England likely as a consequence of air and sea water warming (Zippay and Helmuth 2012). Warming coupled with eutrophication common in many nearshore coastal areas will likely amplify the conditions that result in bottom water hypoxia, further contributing to subtidal shellfish reef habitat loss. Further investigation of how warming and acidification are impacting the early life history of shellfish could

help elucidate why recruitment failure is common in some areas ofthe northeastern U.S.

Literature Cited

Animal Diversity Web. 2020. [Internet]. *Mytilus edulis*. (accessed 31 August 2020). https://animaldiversity.org/accounts/Mytilus_edulis/.

Bahr LM, Lanier WP. 1981. The ecology of intertidal oyster reefs of the South Atlantic Coast: a community profile. U.S. Fish and Wildlife Service. FWS/OBS-81/15.

Beck MW, Brumbaugh RD, Airoldi L, Carranza A, Coen LD, Crawford C, Defeo O, Edgar G, Hancock B, Kay M, Lenihan H, Luckenbach M, Toropova C, Zhang G. 2011. Oyster reefs at risk and recommendations for conservation, restoration and management. Bioscience 61:107-16.

Burge CA, Eakin CM, Friedman CS, Froelich B, Hershberger PK, Hofmann EE, Petes LE, Prager KC,Weil E, Willis BL, Ford SE, Harvell CD. 2014. Climate change influences on marine infectious diseases: implications for management and society. Annual Review of Marine Science 6:249-77.

Burreson EM, Stokes NA, Friedman CS. 2000. Increased virulence in an introduced pathogen: *Haplosporidium nelsoni* (MSX) in the eastern oyster *Crassostrea virginica*. Journal of Aquatic Animal Health 12(1):1-8.

Clark H, Gobler CJ. 2016. Diurnal fluctuations in CO_2 and dissolved oxygen concentrations do not provide a refuge from hypoxia and acidification for early-life-stage bivalves. Marine Ecology Progress Series 558:43114. https://doi.org/10.3354/meps11852

Coen LD, Luckenbach MW, Breitburg DL. 1999. The role of oyster reefs as essential fish habitat: a review of current knowledge and some new perspectives. American Fisheries Society Symposium 22:438-54.

Dame RF, Zingmark RG, Haskins E. 1984. Oyster reefs as processors of estuarine materials. Journal of Experimental Marine Biology and Ecology 83:239-47.

Dodd LF, Grabowski JH, Piehler MF, Westfield I, Ries JB. 2015. Ocean acidification impairs crab foraging behaviour. Proceedings of the Royal Society B: Biological Sciences 282.

Ekstrom JA, Suatoni L, Cooley SR, Pendleton LH, Waldbusser GG, Cinner JE, Ritter J, Langdon C, van Hooidonk R, Gledhill D, Wellman K, Beck MW, Brander LM, Rittschof D, Doherty C, Edwards PET, Portela R. 2015. Vulnerability and adaptation of US shellfisheries to ocean acidification. Nature Climate Change 5(3):207-14.

Ford SE, Smolowitz R. 2007. Infection dynamics of an oyster parasite in its newly expanded range. Marine Biology 151(1):119-33.

Galtsoff PS. 1964. The American oyster *Crassostrea virginica* (Gmelin). U.S. Fish Wildlife Service Fisheries Bulletin 64:1-480.

Gazeau F, Parker LM, Comeau S, Gattuso J-P, O'Connor WA, Martin S, Pörtner H-O, Ross PM. 2013. Impacts of ocean acidification on marine shelled molluscs. Marine Biology 160(8):2207-45.

Gledhill D, White M, Salisbury J, Thomas H, Misna I, Liebman M, Mook B, Grear J, Candelmo A, Chambers RC, Gobler C, Hunt C, King A, Price N, Signorini S, Stancioff E, Stymiest C, Wahle R, Waller J, Rebuck N, Wang Z, Capson T, Morrison JR, Cooley S, Doney S. 2015. Ocean and coastal acidification off New England and Nova Scotia. Oceanography 25(2):182-97.

Gobler CJ, Baumann H. 2016. Hypoxia and acidification in ocean ecosystems: coupled dynamics and effects on marine life. Biology Letters 12(5):20150976.

Gobler CJ, DePasquale EL, Griffith AW, Baumann H. 2014. Hypoxia and acidification have additive and synergistic negative effects on the growth, survival, and metamorphosis of early life stage bivalves. PLoS One 9(1):e83648.

Griffith AW, Gobler CJ. 2017. Transgenerational exposure of North Atlantic bivalves to ocean acidification renders offspring more vulnerable to low pH and additional stressors. Scientific Reports 7:11394.

Gunter G. 1951. The West Indian tree on the Louisiana coast, and notes on growth of the gulf coast oysters. Science 113:16-517.

Jones SJ, Lima FP, Wethey DS. 2010. Rising environmental temperatures and biogeography: poleward range contraction of the blue mussel, *Mytilus edulis* L., in the western Atlantic. Journal of Biogeography 37(12):2243-59.

Jones SJ, Mieszkowska N, Wethey DS. 2009. Linking thermal tolerances and biogeography: *Mytilus edulis* (L.) at its southern limit on the east coast of the United States. Biological Bulletin 217(1):73-85.

Kirby MX. 2004. Fishing down the coast: Historical expansion and collapse of oyster fisheries along continental margins. Proceedings of the National Academy of Science of the United States of America 101:13096-99.

Kroeker KJ, Kordas RL, Crim R, Hendriks IE, Ramajo L, Singh GS, Duarte CM, Gattuso J-P. 2013. Impacts of ocean acidification on marine organisms: quantifying sensitivities and interaction with warming. Global Change Biology 19(6):1884-96.

Lenihan HS. 1999. Physical-biological coupling on oyster reefs: how habitat structure influencesindividual performance. Ecological Monographs 69:251-75.

Lesser MP. 2016. Climate change stressors cause metabolic depressions in the blue mussel, *Mytilus edulis*, from the Gulf of Maine. Limnology and Oceanography 61:1705-17.

Newell RIE. 1988. Ecological changes in Chesapeake Bay: are they the result of overharvesting the American oyster, *Crassostrea virginica*? *In:* Lynch MP, Krome EC (editors). Understanding the estuary: advances in Chesapeake Bay research. Chesapeake Research Consortium, Baltimore, Maryland, USA.

Newell RIE, Cornwell JC, Owens MS. 2002. Influence of simulated bivalve biodeposition and microphyrobenthos on sediment nitrogen dynamics: a laboratory study. Limnology and Oceanography 47:1367-79.

Paerl HW, Pinckney JL, Fear JM, Peierls BL. 1998. Ecosystem responses to internal and watershed organic matter loading: consequences for hypoxia in the eutrophying Neuse river estuary, NorthCarolina, USA. Marine Ecology Progress Series 166:17-25.

Peterson CH, Grabowski JH, Powers SP. 2003. Estimated enhancement of fish production resulting from restoring oyster reef habitat: quantitative valuation. Marine Ecology Progress Series 264:249-64.

Piehler MF, Smyth AR. 2011. Impacts of ecosystem engineers on estuarine nitrogen cycling. Ecosphere 2:art12.

Ridge JT, Rodriguez AB, Fodrie FJ, Lindquist NL, Brodeur MC, Coleman SE, Grabowski JH, Theuerkauf EJ. 2015. Maximizing oyster-reef growth supports green infrastructure with accelerating sea-level rise. Scientific Reports 5:14785.

Ries JB, Cohen AL, McCorkle DC. 2009. Marine calcifiers exhibit mixed responses to CO_2-induced ocean acidification. Geology 37:1131-4.

Rodriguez AB, Fodrie FJ, Ridge JT, Lindquist NL, Theuerkauf EJ, Coleman SE, Grabowski JH, Brodeur MC, Gittman RK, Keller DA, Kenworthy MD. 2014. Oyster reefs can outpace sea-level rise. Nature Climate Change 4:493-7.

Rothschild BJ, Ault JS, Goulletquer P, Heral M. 1994. Decline of the Chesapeake Bay oyster population: a century of habitat destruction and overfishing. Marine Ecology Progress Series 111:29-39.

Shumway S. 1996. Natural environmental factors. *In:* Kennedy VS, Newell RIE, Ebele AF (eds). The eastern oyster, *Crassostrea virginica*. Maryland Sea Grant, College Park, Maryland, USA. pp. 467–513.

Smolowitz R. 2013. A review of current state of knowledge concerning *Perkinsus marinus* effects on *Crassostrea virginica* (Gmelin) (the Eastern oyster). Veterinary Pathology 50(3):404-11.

Speights C J, Silliman BR, McCoy MW. 2017. The effects of elevated temperature and dissolved ρCO_2 on a marine foundation species. Ecology and evolution 7(11):3808–14. https://doi.org/10.1002/ece3.2969

Ulanowicz RE, Tuttle JH. 1992. The trophic consequences of oyster stock rehabilitation in Chesapeake Bay. Estuaries 15:298-306.

U.S. Global Change Research Program (USGCRP). 2017. Climate Science Special Report: Fourth National Climate Assessment. Volume I. [Wuebbles, D.J., D.W. Fahey, K.A. Hibbard, D.J. Dokken, B.C. Stewart, and T.K. Maycock (eds.)]. U.S. Global Change Research Program. Washington, DC.USA. 470 pp. doi:10.7930/J0J964J6.

Waldbusser GG, Voigt EP, Bergschneider H, Green MA, Newell RIE. 2011. Biocalcification in the eastern oyster (*Crassostrea virginica*) in relation to long-term trends in Chesapeake Bay pH. Estuaries and Coasts 34:221-31.

Wells HW. 1961. The fauna of oyster beds, with special reference to the salinity factor. Ecological Monographs 31:239-66.

Zippay ML, Helmuth B. 2012. Effects of temperature change on mussel, *Mytilus*. Integrative Zoology 7:312-27.

zu Ermgassen PSE, Grabowski JH, Gair JR, Powers SP. 2016. Quantifying fish and mobile invertebrate production from a threatened nursery habitat. Journal of Applied Ecology 53:596-606.

zu Ermgassen PSE, Spalding MD, Blake B, Coen LD, Dumbauld B, Geiger S, Grabowski JH, Grizzle R, Luckenbach M, McGraw K, Rodney W, Ruesink JL, Powers SP, Brumbaugh RD. 2012. Historical ecology with real numbers: past and present extent and biomass of an imperilled estuarine habitat. Proceedings of the Royal Society B: Biological Sciences 279:3393-400.

Marine Rocky Bottom <200m

System: Marine

Subsystem: Subtidal <200m

Class: Rocky Bottom

Sub-class: Bedrock, Rubble, Cobble, Gravel

Geographic Area: Entire Area

Overall Vulnerability Rank = Low

Habitat Sensitivity = Low

Climate Exposure = High

	**Marine Rocky Bottom <200m**	Attribute Mean	Data Quality	Distribution of Expert Scores
Sensitivity Attributes	Habitat condition	2.1	2.2	
	Habitat fragmentation	2.2	2.2	
	Distribution/Range	3.1	2.4	
	Mobility/Ability to spread or disperse	2.2	2.4	
	Resistance	1.8	2.4	
	Resilience	2.3	2.4	
	Sensitivity to changes in abiotic factors	2.1	1.8	
	Sensitivity and intensity of non−climate stressors	2.3	2	
	Dependency on critical ecological linkages	1.6	1.2	
	Sensitivity Component Score	Low		
Exposure Factors	Sea surface temp	3.5	3	
	Bottom temp	n/a	n/a	
	Air temp	n/a	n/a	
	River temp	n/a	n/a	
	Surface salinity	1.2	3	
	Bottom salinity	n/a	n/a	
	pH	4	2.4	
	Sea level rise	n/a	n/a	
	Precipitation	n/a	n/a	
	Floods	n/a	n/a	
	Droughts	n/a	n/a	
	Exposure Component Score	High		
**Overall Vulnerability Rank**		**Low**		

Habitat Name: Marine Rocky Bottom <200 m

System: Marine

Subsystem: Subtidal <200 m

Class: Rocky Bottom

Sub-class: Bedrock, Rubble, Cobble, Gravel

Geographic Area: Entire Area

Habitat Description: This sub-class includes marine nearshore subtidal rocky bottom bedrock, rubble, cobble/gravel (<200 m). Rocky bottom habitat established on surfaces and crevices of relatively immobile rocky surfaces, including loose rocks of various sizes (rubble, cobble/gravel) and exposed bedrock. In addition, this habitat includes the epibenthic flora and fauna associated with hard bottoms, including calcareous algae. This subclass does not include non-calcareous algae, which are included in marine aquatic bed habitat narrative. Includes shallow water corals growing on rocky bottom in <150 m water depths.

Natural rocky habitats range from granule/pebble (or gravel) to cobbles, boulders, and ledge/bedrock. Glacial deposits of boulders and cobble occur on the nearshore New England shelf and on Georges Bank (Kostylev et al. 2005), and bedrock outcrops occur in shallow water in the Gulf of Maine and on Georges Bank (Stevenson et al. 2004; Quattrini et al. 2015). Gravel is commonly found adjacent to bedrock outcrops and glacial moraines at shallow depths (20-40m) and some deeper waters (100m) in eastern Maine (Stevenson et al. 2004). Biota associated with all these habitat types also varies. Soft corals (alcyonarians) can be found attached to rocky habitats, and provide additional structured habitat, though they primarily occur in slope environments, with limited numbers between 50 and 150 m (Stevenson et al.2004).

Overall Climate Vulnerability Rank: Low (67% certainty from bootstrap analysis). The majority of the bootstrap results match the results of the categorical vulnerability rank, but 33% of the bootstrap results were in the Moderate rank. This indicates that this habitat is in the high range of the Low vulnerability rank.

Climate Exposure: High. The overall High component score was influenced by two Very High climate exposure means: Sea Surface Temperature and pH (3.5 and 4.0, respectively). The exposure attribute mean for Surface Salinity was 1.2. pH is projected to drastically decrease from historic levels for the entire study area by the end-of-century (increasing ocean acidification). Projected changes in Sea Surface Temperature are high to very high throughout the range, but slightly greater in the Gulf of Maine, where rocky bottom is more common than in the Mid-Atlantic.

Habitat Sensitivity: Low. Only one of the nine sensitivity attribute means was >2.3 (Distribution/Range at 3.1). The other attribute means were between 1.6 and 2.3. The high attribute mean for Distribution/Range likely reflects the skewed distribution of rocky bottom habitats in the Gulf of Maine and southern New England compared to the Mid-Atlantic. Attributes with more moderate means (2.2 or2.3) were also spread across all of the scoring bins (e.g., Resilience, Sensitivity and Intensity of Non−Climate Stressors, and Mobility/Ability to Spread or Disperse). This likely reflects the variability in the sensitivity of the abiotic (rocky bottom) component compared to the biotic (flora and fauna) component of this habitat subclass. Rocky bottom habitats are unable to move or spread substantial distances, although the flora and fauna associated with them are generally capable of moving through reproductive dispersal or migration.

Data Quality & Gaps: For the exposure factors, the data quality scores for Surface Salinity and Sea Surface Temperature were high (3.0 for both), and were moderate for pH (2.4). The lower score for pH likely reflects the low resolution of CMIP5 projections for climate exposure in nearshore, shallow coastal areas.

For habitat sensitivity, data quality for six of the nine attributes were scored as Moderate (2.2 and 2.4), while the other three attributes were between 1.2 and 2.0. The low to moderate data quality scores may reflect uncertainty in responses to climate change from the biota associated with nearshore rocky bottom habitats.

Positive or Negative Climate Effect for the Northeast U.S.: The effect of climate change on marine nearshore rocky bottom in the Northeast U.S. is expected to be mostly negative (95% of the experts' scores were negative and 5% neutral).

Climate Effects on Habitat Condition and Distribution: In general, natural rocky substrates are not themselves sensitive to climate change, but associated organisms are, and responses vary between species. Subtidal organisms are less adapted to extreme conditions (e.g., temperature, salinity) than those in the intertidal zone. As ocean acidification increases, echinoderms associated with rocky bottom habitats will experience negative effects on their growth and survival. Kroeker et al. (2013) reported the largest effects on growth for larval echinoderms. For some species, the function of high complexity rocky habitat may decrease as waters warm due to spatial mismatch between these habitats and the thermal niche of species. For example, Hare et al. (2012) found that suitable rocky habitat for cusk in the Gulf of Maine, Georges Bank, and the Scotian Shelf will shrink and fragment as temperatures increase.

This habitat sub-class also includes shallow corals associated with rocky habitats in the Northeast U.S. Morato et al. (2020) modeled projected shifts in the distribution of cold-water corals by the end of the century under the RCP8.5 emissions scenario. While these corals primarily occur in waters deeper than 150 meters, most can live at depths as shallow as 20 to 100 meters. They projected an overall loss of suitable habitat and a shift in the depth distribution of several scleractinian corals (*L. pertusa* and *M. oculata*) to deeper waters, as climate change makes shallower depths unsuitable habitat. While they found an even more significant reduction of suitable habitat for octocorals, they projected a shift toward shallower depths for *A. arbuscula* and *A. armata* in the northernmost North Atlantic (northward of our study area). These projected losses in suitable habitat were due largely to ocean acidification, a decrease in food availability, and warming of deeper waters (Morato et al. 2020). Temperature increases of 3°C are projected for the upper ocean (0-300 m) in the Northwest Atlantic with a doubling of CO_2 by 2100 (Saba et al. 2016).

In addition, because oxygen is less soluble in warmer water, warming ocean waters can reduce the supply of dissolved oxygen available to benthic animals (Levin and Le Bris 2020). As surface waters warm, the water column becomes more stratified which reduces nutrient input from below the photic zone, primary production decreases and less particulate organic matter is delivered to the benthic animals that rely on it for food (Sweetman et al. 2017; Levin and Le Bris 2020). The combined effects of increased vertical stratification and reduced oxygen solubility in warmer water is expected to lead to widespread deoxygenation of oceanic waters, although this concern is mostly in areas >200 m (Levin and Le Bris 2020).

Rocky bottom substrates generally have limited mobility, and natural sediment transport may be further limited by hardened shoreline structures. However, organisms associated with rocky habitats have a high capability to spread (e.g., larval dispersal) and establish, as long as hard substrate is present. There is an abundance of rocky substrate in New England. In the Mid-Atlantic, where natural rocky substrates are less common, organisms that attach to hard substrates are more restricted to artificial hard substrates. However, there is some evidence that rocky habitat epifauna do not settle, grow, and survive as successfully on all types of artificial substrates (e.g., seawalls, pilings) (Gittman et al. 2016). Subtidal rocky habitats (and their associated flora and fauna) may also experience some disturbance from storms,which are expected to increase in frequency and intensity in the Northeast region (USGCRP 2017).

Habitat Summary: Nearshore subtidal rocky bottom habitat ranges from pristine to moderately degraded (EPA 2012), with greater impacts from anthropogenic activities closer to shore. Coastal development and shoreline hardening affect benthic and fish communities through impacts on water quality, reduction in shallow water habitat, changes in hydrology, and increases in nutrient inputs (Bilkovic and Roggero 2008; Kornis et al. 2017). Increased shoreline hardening in heavily populated and industrialized areas is expected as climate change effects become more severe and widespread. Fauna and flora can be highly sensitive to eutrophication from increased nutrient runoff. Dredging in shallow subtidal areas (Johnson et al. 2008) and bottom fishing in can also damage natural rocky bottom habitats (Stevenson et al. 2004), though several areas of nearshore rocky bottom are protected from mobile bottom gear. Marine construction in subtidal areas (e.g., marine terminals, wind farms) can convert rocky bottom habitat to non-native habitats, and shade epiflora growing on rocky bottoms (Johnson et al. 2008).

Bedrock, boulders, and large cobble are highly resistant to physical disturbance to a greater degree than smaller substrates like gravel. When physically disturbed (e.g., ice scour, storms), the function of these habitats is not impaired, though they may be temporarily buried by mobile sand. The time for species to recover from disturbance varies depending on the life history characteristics and the type and intensity of disturbance (Bertness et al. 2002). Recovery of longer-lived species (e.g., sponges, anemones, tunicates, molluscs) in deeper water can take years, although mobile species generally re-occupy rocky habitats relatively quickly after disturbance (Johnson et al. 2008).

Many introduced species have become established in rocky subtidal habitats in the Northeast, leading to changes in ecological processes and dynamics of fish and other mobile species (Dijkstra et al. 2019). In particular, warming temperatures and increasing frequency, intensity, and variability of storms favor the growth and persistence of turf macroalgae, which alter rocky bottom habitats, increase patchiness, and cause changes up the food web (Djikstra et al. 2017). Subtidal rocky bottom infauna and epifauna have been affected by the green crab (*Carcinus maenas*), an invasive species which is believed to have been carried by ships in ballast water and sold as fish bait in much of the world. It now has established populations in New England and northern Mid-Atlantic in both subtidal and intertidal zones. It is a predator of many forms of marine life, including worms and mollusks (GISD 2020). In some areas (particularly New England), the crab's voracious appetite has affected the commercial shellfish industry (Webber 2013; Beal 2014).

Species that graze on epiflora and epifauna (e.g., sea urchins) control the abundances of species associated with rocky bottom habitats. Likewise, top predators (e.g., cod) can control the populations of grazers. For example, in some areas of the Gulf of Maine, urchin populations have exploded and decimated kelp beds (i.e., "urchin barrens"), which has been attributed to overfishing of the top predators (Steneck et al. 2002). The collapse in the populations of sea urchins can cause proliferation of macroalgae species that reduces species diversity of rocky habitats, and can interfere with settlement of benthic and demersal larval life stages.

Literature Cited

Beal BF. 2014. Final report: Green crab, *Carcinus maenas*, trapping studies in the Harraseeket River and manipulative field trials to determine effects of green crabs on the fate and growth of wild and cultured individuals of soft-shell clams, *Mya arenaria* (May to November 2013). Downeast Institute for Applied Marine Research and Education, Beals, ME. 76 pp. (Accessed 30 Oct. 2020). https://downeastinstitute.org/wp-content/uploads/2018/08/1_24-final-report-freeport-shellfish-restoration-project-b.-beal.pdf.

Bilkovic DM, Roggero MM. 2008. Effects of coastal development on nearshore estuarine nekton communities. Marine Ecology Progress Series 358:27-39.

Bertness MD, G.C. Trussell GC, Ewanchuk PJ, Silliman B.R. 2002. Do alternate stable community states exist in the Gulf of Maine rocky intertidal zone? Ecology 83(12):3434-48.

Dijkstra JA, Litterer A, Mello K, O'Brien BS, Rzhanov Y. 2019. Temperature, phenology, and turf macroalgae drive seascape change: Connections to mid-trophic level species. Ecosphere 10(11):e02923. https://doi.org/10.1002/ecs2.2923.

Gittman RK, Scyphers SB, Smith CS, Neylan IP, Grabowski JH. 2016. Ecological consequences of shoreline hardening: a meta-analysis. Bioscience 66(9):763-73.

Global Invasive Species Database (GISD). 2020. [Internet]. Species profile: *Carcinus maenas*. (Accessed 3 September 2020). http://www.iucngisd.org/gisd/species.php?sc=114.

Hare JA, Manderson JP, Nye JA, Alexander MA, Auster PJ, Borggaard DL, Capotondi AM, Damon-Randall KB, Heupel E, Mateo I, O'Brien L, Richardson DE, Stock CA, Biegel ST. 2012.

Cusk (*Brosme brosme*) and climate change: assessing the threat to a candidate marine fish species under the US Endangered Species Act. ICES Journal of Marine Science 69(10):1753-68.

Johnson MR, Boelke C, Chiarella LA, Colosi PD, Greene K, Lellis-Dibble K, Ludemann H, Ludwig M, McDermott S, Ortiz J, Rusanowsky D, Scott M, Smith J. 2008. Impacts to marine fisheries habitat from nonfishing activities in the northeastern United States. NOAA Technical Memorandum. National Oceanic and Atmospheric Administration, National Marine Fisheries Service.NMFS-NE-2O9. 1-328.

Kornis MS, Breitburg D, Balouskus R, Bilkovic DM, Davias LA, Giordano S, Heggie K, Hines AH, Jacobs JM, Jordan TE, King Ryan S, Patrick CJ, Seitz RD, Soulen H, Targett TE, Weller DE, Whigham Dennis, Uphoff J. 2017. Linking the abundance of estuarine fish and crustaceans in nearshore waters to shoreline hardening and land cover. Estuaries and Coasts 40:1464–86. doi:10.1007/s12237-017-0213-6.

Kostylev V, Todd B, Longva O, Valentine P. 2005. Characterization of benthic habitat on Northeastern Georges Bank, Canada. American Fisheries Society Symposium 41:141-52.

Kroeker KJ, Kordas RL, Crim R, Hendriks IE, Ramajo L, Singh GS, Duarte CM, Gattuso J-P. 2013. Impacts of ocean acidification on marine organisms: quantifying sensitivities and interaction with warming. Global Change Biology 19(6):1884-96.

Levin LA, Le Bris N. 2020. The deep ocean under climate change. Science 350(6262):766-8.

Morato T, González-Irusta JM, Dominguez-Carrió C, Wei C-L, Davies A, Sweetman AK, Taranto GH, Beazley L et al. 2020. Climate-induced changes in the suitable habitat of cold-water corals and commercially important deep-sea fishes in the North Atlantic. Global Change Biology 26:2181-202. https://doi.org/10.1111/gcb.14996.

Quattrini AM, Nizinski MS, Chaytor JD, Demopoulos AWJ, Roark EB, France SC, Moore JA, Heyl T, Auster PJ, Kinlan B, Ruppel C, Elliott KP, Kennedy BRC, Lobecker E, Skarke A, Shank TM. 2015. Exploration

of the canyon-incised continental margin of the northeastern United States reveals dynamic habitats and diverse communities. PLoS ONE 10(10):e0139904. https://doi.org/10.1371/journal.pone.0139904.

Saba VS, Griffies SM, Anderson WG, Winton M, Alexander MA, Delworth TL, Hare JA, Harrison MJ, Rosati A, Vecchi GA, Zhang R. 2016. Enhanced warming of the Northwest Atlantic Ocean under climate change. Journal of Geophysical Research: Oceans 121(1):118-32.

Steneck R, Graham MH, Bourque BJ, Corbett D, Erlandson JM, Estes JA, Tegner MJ. 2002. Kelp forest ecosystems: biodiversity, stability, resilience and future. Marine Sciences Faculty Scholarship. 65. https://digitalcommons.library.umaine.edu/sms_facpub/65.

Stevenson D, Chiarella L, Stephan D, Reid R, Wilhelm K, McCarthy J, Pentony M. 2004. Characterization of the fishing practices and marine benthic ecosystems of the Northeast U.S. Shelf, and an evaluation of the potential effects of fishing on Essential Fish Habitat. NOAA Technical Memorandum NMFS-NE-181.179 p. https://www.midatlanticocean.org/roa-midatlanticocean-org/wp-content/uploads/2016/01/stevenson-et-al-2004.pdf.

Sweetman AK, Thurber AR, Smith CR, Levin LA, Mora C, Wei C-L, Gooday AJ, Jones DOB, Rex M, Yasuhara M, Ingels J, Ruhl HA, Frieder CA, Danovaro R, Würzberg L, Baco A, Grupe BM, Pasulka A, Meyer KS, Dunlop KM, Henry L-A, Roberts JM. 2017. Major impacts of climate change on deep-sea benthic ecosystems. Elementa Science of the Anthropocene 5:4. http://doi.org/10.1525/elementa.203.

U.S. Environmental Protection Agency (EPA). 2012. National Coastal Condition Report IV. EPA-R-842-10-003. US Environmental Protection Agency, Washington D.C. https://www.epa.gov/sites/production/files/2014-10/documents/0_nccr_4_report_508_bookmarks.pdf.

U.S. Global Change Research Program (USGCRP). 2017. Climate Science Special Report: Fourth National Climate Assessment. Volume I [Wuebbles, D.J., D.W. Fahey, K.A. Hibbard, D.J. Dokken, B.C. Stewart, and T.K. Maycock (eds.)]. U.S. Global Change Research Program. Washington, DC. USA. 470 pp. doi: 10.7930/J0J964J6.

Webber MM. 2013. Results of the one-day green crab trapping survey conducted along the Maine coast from August 27 to 28, 2013. Maine Department of Marine Resources, Augusta, ME. 6 pp. (Accessed 30 Oct. 2020). https://www.maine.gov/dmr/science-research/species/invasives/greencrabs/documents/trapsurveyrpt2013.pdf.

Marine Mud Bottom <200m

System: Marine

Subsystem: Subtidal <200m

Class: Unconsolidated Bottom

Sub-class: Mud

Geographic Area: Entire Area

Overall Vulnerability Rank = Low

Habitat Sensitivity = Low

Climate Exposure = High

	**Marine Mud Bottom <200m**	Attribute Mean	Data Quality	Distribution of Expert Scores
Sensitivity Attributes	Habitat condition	2.4	2.4	
	Habitat fragmentation	1.8	2.6	
	Distribution/Range	1.6	2.4	
	Mobility/Ability to spread or disperse	1.5	2.2	
	Resistance	2.3	2.4	
	Resilience	1.5	2.4	
	Sensitivity to changes in abiotic factors	1.9	2.4	
	Sensitivity and intensity of non−climate stressors	2.7	2.4	
	Dependency on critical ecological linkages	1.6	1.6	
	Sensitivity Component Score	Low		
Exposure Factors	Sea surface temp	3.4	3	
	Bottom temp	n/a	n/a	
	Air temp	n/a	n/a	
	River temp	n/a	n/a	
	Surface salinity	1.3	3	
	Bottom salinity	n/a	n/a	
	pH	4	2.4	
	Sea level rise	n/a	n/a	
	Precipitation	n/a	n/a	
	Floods	n/a	n/a	
	Droughts	n/a	n/a	
	Exposure Component Score	High		
**Overall Vulnerability Rank**		**Low**		

- Low
- Moderate
- High
- Very High

Habitat Name: Marine Mud Bottom <200 m

System**:** Marine

Subsystem: Subtidal <200 m

Class: Unconsolidated Bottom

Sub-class: Mud

Geographic Area: Entire Area

<u>Habitat Description</u>: This sub-class includes subtidal nearshore mud habitats from mean low water to locations where the depth reaches 200 meters. This habitat subclass includes the epifauna and infauna associated with unconsolidated mud bottom, such as non-reef-forming mollusks (e.g., soft-shell clams, hard clams, sea scallops, surf clams, ocean quahogs), marine worms, small crustaceans, gastropods, and polychaetes. This subclass excludes specific habitats identified elsewhere (i.e., non-calcareous algal bed, rooted vascular beds, and reef-forming mollusks such as blue mussels and eastern oysters).

<u>Overall Climate Vulnerability Rank</u>: Low (81% certainty from bootstrap analysis). Although the majority of the bootstrap results match the results of the categorical vulnerability rank, 19% of the bootstrap results were in the Moderate vulnerability rank.

<u>Climate Exposure</u>: High. The overall high exposure score was influenced by two High attribute means: Sea Surface Temperature (3.4) and pH (4.0). No other sensitivity attribute means were above 2.0. The high climate exposure score reflects the high degree of certainty that sea surface temperature will increase and pH will decrease throughout the region to a significant degree compared with historic levels.

<u>Habitat Sensitivity</u>: Low. All nine of the sensitivity attribute means were ≤2.7. Sensitivity and Intensity of Non−Climate Stressors had the highest mean score (2.7), and Habitat Condition the next highest (2.4). The scores are generally indicative of the resilient and spatially extensive (low fragmentation) nature of mud habitats.

<u>Data Quality & Gaps</u>: The data quality scores for two of the three climate exposure factors (Sea Surface Temperature and Surface Salinity) were scored High (3.0). pH was scored relatively Low (2.2), which is likely attributed to the low resolution of CMIP5 projections for nearshore, shallow areas.

For habitat sensitivity, generally the data quality scores were 2.4 ± 0.2 except for Dependency on Critical Ecological Linkages (1.6). This likely reflects a moderate degree of confidence in the responses of mud substrate and associated organisms to climate change. However, more research is needed to determine whether the habitat condition of subtidal marine mud habitats relies on critical ecological linkages.

<u>Positive or Negative Climate Effect in the Northeast U.S. Shelf</u>: The effect of climate change on marine subtidal mud (nearshore <200m) in the Northeast U.S. Shelf is expected to be largely negative (70% of the experts' scores were negative and 30% were neutral).

<u>Climate Effects on Habitat Condition and Distribution</u>: The distribution of nearshore marine subtidal mud habitat is unlikely to change significantly due to climate change, except for a potentially small expansion into the intertidal zone due to sea level rise. However, the condition of marine subtidal mud habitats may be significantly altered by a combination of physical changes in vertical stratification of thewater column, and temperature and biogeochemical changes related to coastal acidification.

Mollusks associated with subtidal mud habitats appear to be affected by warming waters, and to a lesser degree water quality. For example, ocean quahog landings have shifted further north along the Atlantic coastline with substantial landings from southern New England waters (MAFMC 2019). Increasing water temperatures, high nutrient levels, and algal blooms can trigger reductions in dissolved oxygen in the nearshore water column and in sediments, which can have detrimental effects to infaunal and epifaunal

species associated with mud habitats (Sharp et al. 1982; Brownlee et al. 2005).

The drivers of ocean acidification in nearshore coastal habitats in the Northeast US are varied but include local effects of adjacent rivers and internal dynamics of production and respiration, as well as remote drivers such as the mixing of highly saturated (aragonite) Gulf Stream water with relatively low saturatedwaters from the Gulf of St. Lawrence and Labrador Sea (Wanninkhof et al. 2015). Although marine waters, in general, may be less susceptible to acidification than estuarine water because they are more influenced by oceanic waters, nearshore marine waters can be subject to more acid sources and are generally less buffered than oceanic waters (i.e., differences in the amount of dissolved inorganic carbon,dissolved and particulate organic carbon, total alkalinity and nutrients from riverine and estuarine sources) (Ekstrom et al. 2015; Gledhill et al. 2015). Predicting the magnitude of acidification and impact on biological systems will depend on the source of acidification. For example, acidification in subtidal marine mud habitats may be due to high river flows (i.e., freshwater tends to have low pH and low total alkalinity; Salisbury et al. 2008), low pH ocean waters, or acidification caused by bottom water net respiration of sinking organic matter. Adding to this complication is the phenomenon of time of emergence or the time when a signal emerges from the noise of natural variability (Turk et al. 2019). That is, rapid warming will ameliorate changes in coastal acidification until the influx of inorganic carbon dueto climate change overwhelms the increase in pH caused by the warming (Salisbury and Jonsson 2018).

Mollusks are sensitive to ocean acidification (Gazeau et al. 2013; Waldbusser and Salisbury 2014), and in general the larval life stage is more sensitive than juveniles and adults (Kroeker et al. 2013). Ocean acidification is expected to reduce the shell size, thickness, calcification rates and growth, and survival of embryo and larval bivalves and gastropods (Gazeau et al. 2013). Some studies have shown additive and synergistic negative effects from ocean acidification and low dissolved oxygen on the growth, survival, and metamorphosis of larval bay scallop and hard clams (Kroeker et al. 2013; Griffith and Gobler 2017). However, most ocean observing programs characterize bottom water parameters using sensors that are actually located a meter or more off the bottom to reduce the possibility of damaging the sensors if they make contact with the bottom or to avoid measuring resuspension of sediment. Water quality dynamics in sediments are usually measured using specialized equipment for targeted studies (e.g., Onken et al. 2010), and are therefore, less consistently measured or available to the modeling community.

Habitat Summary: Anthropogenic impacts in nearshore mud habitats include mineral mining, navigational dredging, and bottom-tending fishing gear (Stevenson et al. 2004; Johnson et al. 2008). Marine subtidal mud habitats support important biological communities of infaunal organisms, sponges, polychaetes, amphipods, mysids, and other organisms that can take advantage of organic matter deposition in these relatively quiescent habitats. Although mud sediments and associated infauna are sensitive to dredging impacts, most species recover relatively rapidly. The rates of recovery of fine-grained mud and clay deposits depend upon the frequency of natural and anthropogenic disturbances and may be less than one year where a frequent disturbance regime is common (Newell et al. 1998).

Dredging can result in a 30-70% decrease in the benthic species diversity and 40-95% reduction in number of individuals and biomass (Wilber et al. 2005). Because this type of habitat is so extensive in the Northeast US, there is generally a high degree of connectivity. That is, if there is a physical disturbance in any one location, there is often a strong source population in the surrounding areas that can recruit to the site of the perturbation when recovered. Rijnsdorp et al. (2018) also documented that the impact of physical disturbances (such as bottom trawling) increased in habitats with many long-lived species and the mud habitats tend to contain mostly short-lived species. For these reasons, many habitat impact models developed by expert panels tend to characterize these habitats as having relatively low susceptibility and fast recovery times (Grabowski et al. 2014; Smeltz et al. 2019). However, Stevenson etal. (2004) reported bottom-tending mobile fishing gear tend to have somewhat higher impacts to physical and biological features of marine mud bottom compared to sand habitats.

The most pervasive climate-related threat to these habitats are likely to be the indirect effects of warming on stratification and respiration. By cutting off the bottom waters of marine subtidal areas from relatively oxygenated and low acidity surface waters, so called ‘dead zones’ of relatively high respiration, low dissolved oxygen, and high acidity may expand without significant offsetting of land derived nutrients that fuel eutrophication (Breitburg et al. 2018). Water quality management may become even more important for the protection of these habitats as climate change makes nutrients more impactful to ecosystems. Fortunately, with comprehensive habitat protection, these habitats have shown a tendency to recover relatively quickly even after extensive hypoxia has been ameliorated.

Marine subtidal mud habitats are also exposed to coastal development and other anthropogenic stressors. Coastal population density and agriculture are associated with higher eutrophication and contamination, which can disturb benthic habitat quality. According to the 2012 EPA Coastal Condition Report, sediment quality varies throughout the region, with the poorest sediment quality in proximity to urbanized areas. Low sediment quality ratings were primarily driven by sediment contamination, which are mostly due to elevated levels of metals, polychlorinated biphenyls, and pesticides (EPA 2012). Fine-grained mud substrates generally contain higher total organic carbon (TOC) levels compared to coarse-grained, sandy sediments. Because contaminants such as polyaromatic hydrocarbons (PAH), pesticides, and polychlorinated biphenyl are sequestered in the TOC fraction of sediments, mud sediments tend to be more impacted by contaminants than sand substrates (ICES 1992; Pearce 1994). Benthic infauna are sensitive to contaminants and have toxic effects to many, including PAH compounds. PAH can persist in sediments for decades after the initial contamination, causing disruption of physiological and metabolic processes of benthic organisms (Vandermeulen and Mossman 1996).

Dredging, shoreline stabilization (e.g., riprap revetment, bulkheads, jetties, groins), and beach nourishment can alter the depth and sediment characteristics, with subsequent changes in infauna and epifauna/epiflora. Riprap revetment can convert mud bottoms to large diameter, engineered stone in the upper subtidal zone. All hardened shorelines have the potential to erode shallow water subtidal mud bottom. Hardened shorelines have been shown to have lower abundance, biomass, and diversity of benthic prey and predators (Seitz et al. 2006; Morley et al. 2012) and can have higher incidence of marineexotic/invasive species compared to native material (Tyrrell and Byers 2007).

Subtidal infauna and epifauna are sensitive to invasive species, including green crab (*Carcinus maenas*) which is believed to have been carried by ships in ballast water and sold as fish bait in much of the world.It now has established populations in New England and northern Mid-Atlantic in both subtidal and intertidal zones. It is a predator of many forms of marine life, including worms and mollusks (GISD 2020). In some areas (particularly New England), the crab’s voracious appetite has affected the commercial shellfish industry (Webber 2013; Beal 2014).

Literature Cited

Beal BF. 2014. Final report: Green crab, *Carcinus maenas*, trapping studies in the Harraseeket River and manipulative field trials to determine effects of green crabs on the fate and growth of wild and cultured individuals of soft-shell clams, *Mya arenaria* (May to November 2013). Downeast Institute for Applied Marine Research and Education, Beals, ME. 76 pp. (Accessed 30 Oct. 2020). https://downeastinstitute.org/wp-content/uploads/2018/08/1_24-final-report-freeport-shellfish-restoration-project-b.-beal.pdf.

Breitburg D,Levin LA, Oschlies A, Grégoire M, Chavez FP, Conley DJ, Garçon V, Gilbert D, Gutiérrez D, Isensee K, Jacinto GS, Limburg KE, Montes I, Naqvi SWA, Pitcher GC, Rabalais NN, Roman MR, Rose KA, Seibel BA, Telszewski M, Yasuhara M, Zhang J. 2018. Declining oxygen in the globalocean and coastal waters. Science 359(6371):eaam7240.

Brownlee EF, Sellner SG, Sellner K.G. 2005. *Prorocentrum minimum* blooms: potential impacts on dissolved oxygen and Chesapeake Bay oyster settlement and growth. Harmful Algae 4(3):593-602. https://doi.org/10.1016/j.hal.2004.08.009

Ekstrom JA, Suatoni L, Cooley SR, Pendleton LH, Waldbusser GG, Cinner JE, Ritter J, Langdon C, van Hooidonk R, Gledhill D, Wellman K, Beck MW, Brander LM, Rittschof D, Doherty C, Edwards PET, Portela R. 2015. Vulnerability and adaptation of US shellfisheries to ocean acidification. Nature Climate Change 5(3):207-14.

Gazeau F, Parker LM, Comeau S, Gattuso J-P, O'Connor WA, Martin S, Pörtner H-O, Ross PM. 2013. Impacts of ocean acidification on marine shelled molluscs. Marine Biology 160(8):2207-45.

Gledhill D, White M, Salisbury J, Thomas H, Misna I, Liebman M, Mook B, Grear J, Candelmo A, Chambers RC, Gobler C, Hunt C, King A, Price N, Signorini S, Stancioff E, Stymiest C, Wahle R, Waller J, Rebuck N, Wang Z, Capson T, Morrison JR, Cooley S, Doney S. 2015. Ocean and coastal acidification off New England and Nova Scotia. Oceanography 25(2):182-97.

Global Invasive Species Database (GISD). 2020. [Internet]. Species profile: *Carcinus maenas*. (Accessed 03 Aug. 2020). http://www.iucngisd.org/gisd/species.php?sc=114.

Grabowski JH, Bachman M, Demarest C, Eayrs S, Harris BP, Malkoski V, Packer D, Stevenson D. 2014. Assessing the vulnerability of marine benthos to fishing gear impacts. Reviews in Fisheries Science & Aquaculture 22(2):142-55.

Griffith AW, Gobler CJ. 2017. Transgenerational exposure of North Atlantic bivalves to ocean acidification renders offspring more vulnerable to low pH and additional stressors. Scientific Reports7:11394.

International Council for the Exploration of the Sea (ICES). 1992. Report of the ICES working group on the effects of extraction of marine sediments on fisheries. Copenhagen (Denmark): ICES Cooperative Research Report #182.

Johnson MR, Boelke C, Chiarella LA, Colosi PD, Greene K, Lellis-Dibble K, Ludemann H, Ludwig M, McDermott S, Ortiz J, Rusanowsky D, Scott M, Smith J. 2008. Impacts to marine fisheries habitat from nonfishing activities in the northeastern United States. NOAA Technical Memorandum. National Oceanic and Atmospheric Administration, National Marine Fisheries Service.NMFS-NE-2O9. 1-328.

Kroeker KJ, Kordas RL, Crim R, Hendriks IE, Ramajo L, Singh GS, Duarte CM, Gattuso J-P. 2013. Impacts of ocean acidification on marine organisms: quantifying sensitivities and interaction withwarming. Global Change Biology 19(6):1884-96.

Mid-Atlantic Fishery Management Council (MAFMC). 2019. [Internet]. Ocean quahog fishery information document. April 1, 2019. (Accessed 23 November 2020). http://www.mafmc.org/surfclams-quahogs.

Morley SA, Toft JD, Hanson KM. 2012. Ecological effects of shoreline armoring on intertidal habitats of a Puget Sound urban estuary. Estuaries and Coasts 35(3):774-84.

Newell RC, Seiderer LJ, Hitchcock DR. 1998. The impact of dredging works in coastal waters: a review of the sensitivity to disturbance and subsequent recovery of biological resources on the sea bed. *In*: Ansell AD, Gibson RN, Barnes M, editors. Oceanography and marine biology: an annual review. Vol. 36. Oban, Argyll (Scotland):UCL Press. 127-78.

Onken R, Garbe H, Schröder S, Janik M. 2010. A new instrument for sediment temperature measurements.

Journal of Marine Science and Technology 15(4):427-33.

Pearce JB. 1994. Mining of seabed aggregates. *In*: Langton RW, Pearce JB, Gibson JA, editors. Selected living resources, habitat conditions, and human perturbations of the Gulf of Maine: environmental and ecological considerations for fishery management. Woods Hole, MA. NOAA Technical Memorandum NMFS-NE-106. 48-50.

Rijnsdorp AD, Bolam S. G, Garcia C, Hiddink JG, Hintzen NT, van Denderen PD, van Kooten T. 2018. Estimating sensitivity of seabed habitats to disturbance by bottom trawling based on the longevity of benthic fauna. Ecological Applications 28(5):1302-12.

Salisbury J, Green M, Hunt C, Campbell J. 2008. Coastal acidification by rivers: a threat to shellfish? Eos, Transactions American Geophysical Union 89(50):513-28.

Salisbury JE, Jönsson BF. 2018. Rapid warming and salinity changes in the Gulf of Maine alter surface ocean carbonate parameters and hide ocean acidification. Biogeochemistry 141(3):401-18.

Seitz RD, Lipcius RN, Olmstead NH, Seebo MS, Lambert DM. 2006. Influence of shallow-water habitats and shoreline development on abundance, biomass, and diversity of benthic prey and predators in Chesapeake Bay. Marine Ecology Progress Series 326:11-27.

Sharp JH, Culberson CH, Church TM. 1982. The chemistry of the Delaware estuary. General considerations. Limnology and Oceanography 27. doi: 10.4319/lo.1982.27.6.1015.

Smeltz TS, Harris BP, Olson JV, Sethi SA. 2019. A seascape-scale habitat model to support management of fishing impacts on benthic ecosystems. Canadian Journal of Fisheries and Aquatic Sciences 76(10):1836-44.

Stevenson D, Chiarella L, Stephan D, Reid R, Wilhelm K, McCarthy J, Pentony M. 2004. Characterization of the fishing practices and marine benthic ecosystems of the northeast US shelf, and an evaluation of the potential effects of fishing on essential habitat. NOAA Technical Memorandum NMFS NE 181. 179 pp.

Turk D, Wang H, Hu X, Gledhill DK, Wang ZA, Jiang L, Cai W-J. 2019. Time of emergence of surface ocean carbon dioxide trends in the North American coastal margins in support of ocean acidification observing system design. Frontiers in Marine Science 6:91.

Tyrrell MC, Byers JE. 2007. Do artificial substrates favor nonindigenous fouling species over native species? Journal of Experimental Marine Biology and Ecology 342(1):54-60.

U.S. Environmental Protection Agency (EPA). 2012. National Coastal Condition Report IV. EPA-R-842-10-003. US Environmental Protection Agency, Washington D.C. https://www.epa.gov/sites/production/files/2014-10/documents/0_nccr_4_report_508_bookmarks.pdf

Vandermeulen JH, Mossman D. 1996. Sources of variability in seasonal hepatic microsomal oxygenase activity in winter flounder (*Pleuronectes americanus*) from a coal tar contaminated estuary. Canadian Journal of Fisheries and Aquatic Sciences 53:1741-53.

Waldbusser GG, Salisbury JE. 2014. Ocean acidification in the coastal zone from an organism's perspective: multiple system parameters, frequency domains, and habitats. Annual Review of Marine Science 6(1):221-47. https://doi.org/10.1146/annurev-marine-121211-172238

Wanninkhof R, Barbero L, Byrne R, Cai W-J, Huang W-J, Zhang J-Z, Baringer M, Langdon C. 2015. Ocean acidification along the Gulf coast and the East coast of the USA. Continental Shelf Research

98:54-71.

Webber MM. 2013. Results of the one-day green crab trapping survey conducted along the Maine coast from August 27 to 28, 2013. Maine Department of Marine Resources, Augusta, ME. 6 pp. (Accessed 30 Oct. 2020). https://www.maine.gov/dmr/science-research/species/invasives/greencrabs/documents/trapsurveyrpt2 013.pdf.

Wilber DH, Brostoff W, Clarke DG, Ray GL. 2005. Sedimentation: Potential biological effects from dredging operations in estuarine and marine environments. DOER Technical Notes Collection (ERDCTN-DOER-E20). U.S. Army Engineer Research and Development Center, Vicksburg, MS.

Marine Sand Bottom <200m

System: Marine

Subsystem: Subtidal <200m

Class: Unconsolidated Bottom

Sub-class: Sand

Geographic Area: Entire Area

Overall Vulnerability Rank = Low

Habitat Sensitivity = Low

Climate Exposure = High

	**Marine Sand Bottom <200m**	Attribute Mean	Data Quality	Distribution of Expert Scores
Sensitivity Attributes	Habitat condition	2.2	2.6	
	Habitat fragmentation	1.8	2.6	
	Distribution/Range	1.5	2.6	
	Mobility/Ability to spread or disperse	1.6	2.6	
	Resistance	2.1	2.2	
	Resilience	1.7	2.4	
	Sensitivity to changes in abiotic factors	1.8	2.2	
	Sensitivity and intensity of non−climate stressors	2.2	2.4	
	Dependency on critical ecological linkages	1.5	1.6	
	Sensitivity Component Score	Low		
Exposure Factors	Sea surface temp	3.4	3	
	Bottom temp	n/a	n/a	
	Air temp	n/a	n/a	
	River temp	n/a	n/a	
	Surface salinity	1.2	3	
	Bottom salinity	n/a	n/a	
	pH	4	2.4	
	Sea level rise	n/a	n/a	
	Precipitation	n/a	n/a	
	Floods	n/a	n/a	
	Droughts	n/a	n/a	
	Exposure Component Score	High		
**Overall Vulnerability Rank**		**Low**		

Low
Moderate
High
Very High

Habitat Name: Marine Sand Bottom <200 m

System**:** Marine

Subsystem: Subtidal <200 m

Class: Unconsolidated Bottom

Sub-class: Sand

Geographic Area: Entire Area

Habitat Description: This sub-class includes marine subtidal sand from mean low water to locations where the depth reaches 200 m. This habitat includes the epifauna and infauna associated with unconsolidated sand bottom, such as non-reef-forming mollusks (e.g., soft-shell clams, hard clams, sea scallops, surf clams, ocean quahogs), marine worms, small crustaceans, gastropods, and polychaetes. Thissubclass excludes specific habitats identified elsewhere (i.e., non-calcareous algal bed, rooted vascular beds, and reef-forming mollusks such as blue mussels and eastern oysters).

Most of the northeast shelf benthic habitat is dominated by sandy habitats, with some geographic and depth variability. Sandy substrate is nearly continuous in the areas shallower than 200 meters south of Cape Cod and on Georges Bank. Areas of sandy substrate exist in small patches in the Gulf of Maine andthe inshore waters of New Hampshire, often offshore of sandy beaches (Stevenson et al. 2004).

Overall Climate Vulnerability Rank: Low (100% certainty from bootstrap analysis).

Climate Exposure: High. Two exposure factors contributed to the High exposure score: Sea Surface Temperature (3.4) and pH (4.0). Sea surface temperature and ocean acidification (decreasing pH) are projected to increase significantly throughout the region.

Habitat Sensitivity: Low. Most sensitivity attributes were centered around the Low and Moderate scoring bins. The highest sensitivity scores were for Sensitivity and Intensity of Non-Climate Stressors and Habitat Condition (both 2.2), followed by Resistance (2.1), and the remaining were ≤1.8. The Low and Moderate sensitivity scores likely reflect the general understanding that sand substrates are highly resistant and resilient to changes, and the infaunal and epifaunal organisms are relatively adapted to variable conditions compared to organisms associated with deep-water habitats.

Data Quality & Gaps: Two of the exposure data quality scores, Sea Surface Temperature and Surface Salinity were High (3.0), while pH was Moderate (2.4).

One of the nine sensitivity attribute data quality scores were Low: Dependency on Critical Ecological Linkages (1.6), and the others were between 2.2 and 2.6. This likely reflects a moderate degree of confidence in the responses of sand substrate and associated organisms to climate change.

Positive or Negative Climate Effect in the Northeast U.S.: The effect of climate change on marine sand <200 m in the Northeast U.S. is expected to be negative (80% of the experts' scores were negative, 20% were neutral). The scorers' overall climate directionality for this sub-class deviated somewhat with the overall sensitivity attribute score, which was relatively low. However, it is important to note that the climate directionality scores do not include an intensity value.

Climate Effects on Habitat Condition and Distribution: One direct climate impact on subtidal sand habitats shallower than 200 m is disturbance from storms, which are expected to increase in frequency and intensity in the Northeast region (USGCRP 2017). Storm energy and current speed and direction transport sediments (Knebel 1981). Bottom currents are more complex north and east of Cape Cod, with some sandy areas consistently in motion and others areas that are relatively stable and moved only by storms (Stevenson et al. 2004). On Georges Bank, for example, sand sediments are continuously redistributed by storms, and tidal and other currents. Associated epifauna and infauna have capacity to move or disperse into new

locations containing sand and mud habitats, especially the planktonic egg andlarval stages.

Although sand substrates can be redistributed through physical forces from storms, climate change is predominantly expected to affect the biota associated with sand bottom habitats. Mollusk species associated with sand infauna/epifauna are sensitive to low pH, with larger negative effects on survival for larvae than adults (Kroeker et al. 2013). Ocean acidification is expected to reduce the shell size, thickness,calcification rates and growth, and survival of embryo and larval bivalves and gastropods (Gazeau et al. 2013). However, marine waters may be less susceptible to ocean acidification and changes in carbonate chemistry than estuarine waters, because they are subject to less acid sources and are generally more buffered than estuarine waters (i.e., differences in the amount of dissolved inorganic carbon, dissolved and particulate organic carbon, total alkalinity and nutrients from riverine and estuarine sources) (Ekstromet al. 2015; Gledhill et al. 2015).

Mollusks are also affected by increasing water temperatures, which are affecting their range (E. Powell and R. Mann, South Carolina Department of Natural Resources, personal communication). Surf clam stocks in the Mid-Atlantic region have declined dramatically over the last 10 years, especially in New Jersey waters, and ocean quahog landings have shifted further north along the Atlantic coastline with substantial landings from southern New England waters (Lewis et al. 2001; MAFMC 2019). Increasing water temperatures, high nutrient levels, and algal blooms can trigger reductions in dissolved oxygen inthe nearshore water column and in sediments, which can have detrimental effects to infaunal and epifaunal species associated with sand habitats (Sharp et al. 1982; Brownlee et al. 2005).

Habitat Summary: Nearshore subtidal sand habitats are impacted by mining and beach nourishment, fishing gear, development, transport, and pollution (Stevenson et al. 2004; Johnson et al. 2008). Sand sediments and associated infauna are sensitive to dredging impacts, although most associated species recovery relatively rapidly (Wilber et al. 2005). Dredging can result in a 30-70% decrease in the benthic species diversity and 40-95% reduction in the number of individuals and biomass. The rates of recovery depend upon the frequency of natural and anthropogenic disturbances, and benthic infauna in sand substrates may take many years to recover (Newell et al. 1998). Nearshore sand deposits are often sourcesfor material in beach nourishment activities (Johnson et al. 2008). Sand habitats tend to be minimally impacted by bottom-tending mobile fishing gear compared with mud or rocky habitats (Stevenson et al. 2004). Wind farms are generally constructed in sandy habitats, but are not believed to substantially alter the range or extent of the habitat (Guida et al. 2017). Shoreline hardening can reduce the supply of sediments to nearshore sandy bottoms, or convert sand bottom to engineered stone in the upper subtidal zone. Hardened shorelines have the potential to erode shallow water subtidal sand bottom (Johnson et al.2008).

According to the 2012 EPA Coastal Condition Report, sediment quality in the Northeast Coast region (Chesapeake Bay and north) is rated fair, with 12% of the coastal area in poor condition and 11% in fair condition, largely driven by sediment contamination from heavy metals, polychlorinated biphenyls, and pesticides, with poorer quality sediments near urban areas. Generally, contaminants are more prevalent in nearshore than offshore habitats, and sand is less impacted by contaminants than mud. Southern New England and Mid-Atlantic coastal areas have high contaminant loads due to legacy pollution from industrialization, and continued urbanization (EPA 2012). Coarse-grained sediments such as sand generally contain less total organic carbon (TOC) levels compared to soft-grained sediments. Because contaminants such as polyaromatic hydrocarbons (PAH), pesticides, and polychlorinated biphenyl are sequestered in the TOC fraction of sediments, sand sediments tend to be less impacted by contaminants than mud substrates (ICES 1992; Pearce 1994). Benthic infauna are sensitive to contaminants and have toxic effects to many, including PAH compounds. PAH can persist in sediments for decades after the initial contamination, causing disruption of physiological and metabolic processes of benthic organisms (Vandermeulen and Mossman1996).

Subtidal infauna and epifauna are sensitive to invasive species, including green crab (*Carcinus maenas*) which is believed to have been carried by ships in ballast water and sold as fish bait in much of the world.It

now has established populations in New England and northern Mid-Atlantic in both subtidal and intertidal zones. It is a predator of many forms of marine life, including worms and mollusks (GISD 2020). In some areas (particularly New England), the crab's voracious appetite has affected the commercial shellfish industry (Webber 2013; Beal 2014).

Literature Cited

Beal BF. 2014. Final report: Green crab, *Carcinus maenas*, trapping studies in the Harraseeket River and manipulative field trials to determine effects of green crabs on the fate and growth of wild and cultured individuals of soft-shell clams, *Mya arenaria* (May to November 2013). Downeast Institute for Applied Marine Research and Education, Beals, ME. 76 pp. (Accessed 30 Oct. 2020). https://downeastinstitute.org/wp-content/uploads/2018/08/1_24-final-report-freeport-shellfish-restoration-project-b.-beal.pdf.

Brownlee EF, Sellner SG, Sellner K.G. 2005. *Prorocentrum minimum* blooms: potential impacts on dissolved oxygen and Chesapeake Bay oyster settlement and growth. Harmful Algae 4 (3):593-602. https://doi.org/10.1016/j.hal.2004.08.009

Ekstrom JA, Suatoni L, Cooley SR, Pendleton LH, Waldbusser GG, Cinner JE, Ritter J, Langdon C, van Hooidonk R, Gledhill D, Wellman K, Beck MW, Brander LM, Rittschof D, Doherty C, Edwards PET, Portela R. 2015. Vulnerability and adaptation of US shellfisheries to ocean acidification. Nature Climate Change 5(3):207-14.

Gazeau F, Parker LM, Comeau S, Gattuso J-P, O'Connor WA, Martin S, Pörtner H-O, Ross PM. 2013. Impacts of ocean acidification on marine shelled molluscs. Marine Biology 160(8):2207-45.

Gledhill D, White M, Salisbury J, Thomas H, Misna I, Liebman M, Mook B, Grear J, Candelmo A, Chambers RC, Gobler C, Hunt C, King A, Price N, Signorini S, Stancioff E, Stymiest C, Wahle R, Waller J, Rebuck N, Wang Z, Capson T, Morrison JR, Cooley S, Doney S. 2015. Ocean and coastal acidification off New England and Nova Scotia. Oceanography 25(2):182-97.

Global Invasive Species Database (GISD). 2020. [Internet]. Species profile: *Carcinus maenas*. (Accessed 3 Aug. 2020). http://www.iucngisd.org/gisd/species.php?sc=114.

Guida V, Drohan A, Welch H, McHenry J, Johnson D, Kentner V, Brink J, Timmons D, Estela-Gomez E. 2017. Habitat mapping and assessment of Northeast Wind Energy Areas. Sterling, VA US Departmentof Interior Bureau of Ocean Energy Management. 312 pp.

International Council for the Exploration of the Sea (ICES). 1992. Report of the ICES working group on the effects of extraction of marine sediments on fisheries. Copenhagen (Denmark): ICES Cooperative Research Report # 182.

Johnson MR, Boelke C, Chiarella LA, Colosi PD, Greene K, Lellis-Dibble K, Ludemann H, Ludwig M, McDermott S, Ortiz J, Rusanowsky D, Scott M, Smith J. 2008. Impacts to marine fisheries habitat from nonfishing activities in the northeastern United States. NOAA Technical Memorandum. National Oceanic and Atmospheric Administration, National Marine Fisheries Service.NMFS-NE-2O9. 1-328.

Knebel HJ. 1981. Processes controlling the characteristics of the surficial sand sheet, US Atlantic outer continental shelf. Marine Geology 42(1-4):349-68.

Kroeker KJ, Kordas RL, Crim R, Hendriks IE, Ramajo L, Singh GS, Duarte CM, Gattuso J-P. 2013. Impacts of ocean acidification on marine organisms: quantifying sensitivities and interaction with warming.

Global Change Biology 19(6):1884-96.

Lewis CVW, Weinberg JR, Davis CS. 2001. Population structure and recruitment of the bivalve *Arctica islandica* (Linnaeus, 1767) on Georges Bank from 1980-1999. Journal of Shellfish Research 20:1135-44.

Mid-Atlantic Fishery Management Council (MAFMC). 2019. [Internet]. Ocean quahog fishery information document. April 1, 2019. (Accessed 23 November 2020). http://www.mafmc.org/surfclams-quahogs.

Newell RC, Seiderer LJ, Hitchcock DR. 1998. The impact of dredging works in coastal waters: a review of the sensitivity to disturbance and subsequent recovery of biological resources on the sea bed. *In*: Ansell AD, Gibson RN, Barnes M, editors. Oceanography and marine biology: an annual review. Vol. 36. Oban, Argyll (Scotland): UCL Press. 127-78.

Pearce JB. 1994. Mining of seabed aggregates. *In*: Langton RW, Pearce JB, Gibson JA, editors. Selected living resources, habitat conditions, and human perturbations of the Gulf of Maine: environmental and ecological considerations for fishery management. Woods Hole, MA. NOAA Technical Memorandum. NMFS-NE-106. 48-50.

Sharp JH, Culberson CH, Church TM. 1982. The chemistry of the Delaware estuary. General considerations. Limnology and Oceanography 27. doi:10.4319/lo.1982.27.6.1015.

Stevenson D, Chiarella L, Stephan D, Reid R, Wilhelm K, McCarthy J, Pentony M. 2004. Characterization of the fishing practices and marine benthic ecosystems of the northeast US shelf, andan evaluation of the potential effects of fishing on essential habitat. NOAA Technical Memorandum NMFS NE 181; 179 pp.

U.S. Environmental Protection Agency (EPA). 2012. National Coastal Condition Report IV. EPA-R-842-10-003. US Environmental Protection Agency, Washington D.C. https://www.epa.gov/sites/production/files/2014-10/documents/0_nccr_4_report_508_bookmarks.pdf

U.S. Global Change Research Program (USGCRP). 2017. Climate Science Special Report: Fourth National Climate Assessment. Volume I [Wuebbles, D.J., D.W. Fahey, K.A. Hibbard, D.J. Dokken, B.C. Stewart, and T.K. Maycock (eds.)]. U.S. Global Change Research Program. Washington, DC.USA. 470 pp. doi: 10.7930/J0J964J6.

Vandermeulen JH, Mossman D. 1996. Sources of variability in seasonal hepatic microsomal oxygenase activity in winter flounder (*Pleuronectes americanus*) from a coal tar contaminated estuary. Canadian Journal of Fisheries and Aquatic Sciences 53:1741-53.

Webber MM. 2013. Results of the one-day green crab trapping survey conducted along the Maine coast from August 27 to 28, 2013. Maine Department of Marine Resources, Augusta, ME. 6 pp. (Accessed 30 Oct. 2020). https://www.maine.gov/dmr/science-research/species/invasives/greencrabs/documents/trapsurveyrpt2 013.pdf.

Wilber DH, Brostoff W, Clarke DG, Ray GL. 2005. Sedimentation: Potential biological effects from dredging operations in estuarine and marine environments. DOER Technical Notes Collection (ERDCTN-DOER-E20). U.S. Army Engineer Research and Development Center, Vicksburg, MS.

Deep Sea Coral and Sponge: Gulf of Maine

System: Marine

Subsystem: Subtidal >150m

Class: Reef

Sub-class: Deep Sea Coral and Sponge

Geographic Area: Gulf of Maine

Overall Vulnerability Rank = High

Habitat Sensitivity = Very High

Climate Exposure = Moderate

	Deep Sea Coral and Sponge: Gulf of Maine	Attribute Mean	Data Quality	Distribution of Expert Scores
Sensitivity Attributes	Habitat condition	3.2	2.2	
	Habitat fragmentation	3.1	2.2	
	Distribution/Range	2.9	2.2	
	Mobility/Ability to spread or disperse	3.1	2	
	Resistance	3.7	2.2	
	Resilience	3.7	2.2	
	Sensitivity to changes in abiotic factors	3.7	2	
	Sensitivity and intensity of non−climate stressors	3.4	1.8	
	Dependency on critical ecological linkages	3.2	1.8	
	Sensitivity Component Score	Very High		
Exposure Factors	Sea surface temp	n/a	n/a	
	Bottom temp	2.8	3	
	Air temp	n/a	n/a	
	River temp	n/a	n/a	
	Surface salinity	n/a	n/a	
	Bottom salinity	1.3	3	
	pH	4	2.4	
	Sea level rise	n/a	n/a	
	Precipitation	n/a	n/a	
	Floods	n/a	n/a	
	Droughts	n/a	n/a	
	Exposure Component Score	Moderate		
**Overall Vulnerability Rank**		**High**		

Low
Moderate
High
Very High

Habitat Name: Deep-sea Coral and Sponge: Gulf of Maine

System: Marine

Subsystem: Subtidal >150 m

Class: Reef

Sub-class: Deep Sea Coral and Sponge

Geographic Area: Gulf of Maine

Habitat Description: For the purposes of this study, the geographic areas for this habitat sub-class include Gulf of Maine deep-sea coral and sponge areas greater than 150 m. This sub-class is distinct from the more offshore coral/sponge areas on the rest of the Northeast continental shelf, slope, in the submarine canyons, and on the seamounts in that 1) they are dense and extensive, albeit spatially rare; 2) they are found relatively nearshore at only about 170-250 m depth, especially in steeper rocky areas; 3) they have low coral biodiversity, dominated by the shallower water structure-forming gorgonians (sea fans or alcyonaceans) *Paramuricea placomus* and *Primnoa resedaeformis*, plus an unknown number of *Acanthogorgia armata* and rarer occurrences of *Paragorgia arborea* and *Anthothela grandiflora*; 4) the populations of *Primnoa* are genetically distinct from those found in the offshore canyons; and, 5) compared to the deepwater slope, canyons, and seamounts, many more of our commercially important fish and shellfish species occur here and thus, there is more evidence of fishing gear and other anthropogenic impacts (Packer et al. 2007; Auster et al. 2013a; Auster et al. 2013b; Auster et al. 2014; Packer et al. 2017b). Structure-forming sponge species found in these hard-bottom areas include *Polymastia, Isodictya,* and *Phakellia/Axinella* spp. (vase/basket demosponges).

Deep-sea corals in the Gulf of Maine have been reported since the 19th century, both as fisheries bycatch and from naturalist surveys (Watling and Auster 2005; Packer et al. 2007; Packer et al. 2017a; Packer et al. 2017b). While at one time they may have been considered common on hard bottoms in the region (Wigley 1968), after a century of intensive fishing pressure using mobile bottom gear such as trawls and dredges as well as fixed gear such as lobster traps, the more dense populations of deep-sea corals and coral habitats are now confined to small areas where the rough bottom topography (e.g., boulders, walls, ridges, escarpments) makes them mostly inaccessible to these fisheries (Auster 2005; Packer et al. 2007; Auster et al. 2013a; Auster et al. 2013b; Watling and Packer et al. 2014; Packer et al. 2017a; Packer et al.2017b).

Overall Climate Vulnerability Rank: High (79% certainty from bootstrap analysis). Although the majority of the bootstrap results match the results of the categorical vulnerability rank, 17% of the bootstrap results were in the Very High vulnerability rank.

Climate Exposure: Moderate. The highest exposure scores were for pH (4.0) and Bottom Temperature (2.8), with a low score for Bottom Salinity (1.3). Although the projected change in pH for the Gulf of Maine is less than in southern New England and the Mid-Atlantic, it is a significant decrease (>5.5 standard deviation) in pH from historic levels.

Habitat Sensitivity: Very High. Eight of the nine sensitivity attribute means were ≥3.1. Distribution/Range scored 2.9. Resistance, Resilience, and Sensitivity to Changes in Abiotic Factors scored highest at 3.7. Deep-sea corals and coral/sponge habitats are fragile communities that take a long time to recover from impacts and disturbance. As a deep-sea habitat, they are very sensitive to changes in abiotic factors such as temperature and carbonate chemistry.

Data Quality & Gaps: For the climate exposure factors, pH scored relatively low (2.4); and Bottom Temperature and Bottom Salinity scored high (3.0). The relative low data quality score for pH is likely reflecting some uncertainty in how the CMIP5 projected changes in surface pH will affect the pH and calcium carbonate ($CaCO_3$) saturation levels in deeper water.

Most of the data quality scores for sensitivity attributes were relatively low, 2.0 and 2.2. Two of the attributes, Sensitivity and Intensity of Non-Climate Stressors and Dependency on Critical Ecological Linkages, scored 1.8.

In spite of the recent spate of deep-sea coral and sponge surveys and fieldwork both on and off the Northeast shelf, our knowledge of the temporal and spatial distribution and abundance of these organisms and habitats, as well as many aspects of their basic biology and habitat requirements, is still limited. In many cases, our knowledge is confined to simple presence and absence data.

Positive or Negative Climate Effect in the Northeast U.S. Shelf: The effects of climate change on deep-sea corals and sponges in the Gulf of Maine is expected to be negative (90% of the experts' scores were negative, 10% were neutral).

Climate Effects on Habitat Condition and Distribution: Ocean acidification is a major threat to deep-sea corals worldwide, with a potential significant loss of deep-sea coral habitat and the ecosystem services they provide (Orr et al. 2005; Guinotte et al. 2006; Tittensor et al. 2010; Thresher et al. 2011; Jackson et al. 2014; Zheng and Cao 2014; Roberts et al. 2016; Morato et al. 2020). Some of the major groups of corals that have been shown to be susceptible to the effects of ocean acidification and which also occur off the Northeast shelf include the colonial stony corals (e.g., *Lophelia pertusa*) (Lunden et al. 2014; Hennige et al. 2015; Büscher et al. 2019; Gomez et al. 2019; Morato et al. 2020) and alcyonaceans (Cerrano et al. 2013; Morato et al. 2020). However, based on recent surveys, stony corals have rarely been found in the Gulf of Maine, and when they are found, are in the form of tiny solitary cup corals (NOAA Northeast Fisheries Science Center and University of Connecticut 2015).

Recently, Morato et al. (2020) utilized environmental niche modelling, species occurrence data, and environmental parameters to model habitat suitability for three stony coral and three alcyonacean species that occur in the North Atlantic. One of the three alcyonaceans, *Paragorgia arborea*, is also found in the Gulf of Maine coral areas (Packer et al. 2019), albeit rarely now compared to the past (Wigley 1968), most likely because like other larger deep-sea corals, it has been "fished out" (Packer et al. 2007; Packeret al. 2017a). Morato et al. (2020) modeled under present-day environmental conditions (years 1951-2000) and a projected severe, high emissions future (years 2081-2100; RCP8.5 or "business-as-usual" scenario). The authors concluded that three predictors acting in concert: ocean warming (bottom water temperature), acidification (aragonite or calcite saturation), and decreasing food availability (as POC flux to the seafloor) will change the availability of suitable habitat for deep-sea corals. Specifically, off the Northeast shelf, predicted losses of alcyonacean habitat was linked to acidification (i.e., shoaling of the calcite saturation horizon), and *P. arborea* is not expected to survive the projected scenario, having at leasta 99% reduction of suitable North Atlantic habitat and almost no refugia by 2100. It is unclear whether this scenario also applies to the relatively-shallower water Gulf of Maine populations, as they probably were not included in their presence-only database used in the model.

Habitat Summary: In the Gulf of Maine and Georges Bank, current observations and historic records suggest that coral habitats were once more extensive and that current habitat represents refuges that have persisted in the face of intensive bottom fishing (Auster et al. 2014; Packer et al. 2017a; Packer pers. comm.), implying that some suitable habitats have been destroyed without recovery to date.

Depending on the study, there are any number of habitat parameters either alone or in combination that have been found to be important predictors of habitat suitability for, or may determine the distribution of,deep-sea corals. As one example, Quattrini et al. (2015) surveyed 11 Northeast submarine canyons, as well as intercanyon and slope sites, plus the Mytilus Seamount, and found that depth, habitat, salinity, and dissolved oxygen together explained 71% of the total variation in deep-sea coral assemblage structure, and the type of broad-scale habitat feature and high habitat heterogeneity in this region was an important factor that influenced the diversity of coral assemblages.

Examining some of the individual parameters across several studies, aragonite and calcite saturation rates,as discussed above (Morato et al. 2020) are an important factor in determining habitat suitability for alcyonaceans (Yesson et al. 2012; Morato et al. 2020). Another key variable for alcyonaceans across a variety of studies is temperature (Yesson et al. 2012; Guinotte and Davies 2014; Morato et al. 2020), with several studies focusing on the same alcyonacean species that also occur in the U.S. side of the Gulf of Maine (e.g., Bryan and Metaxas 2006; Bryan and Metaxas 2007), including using alcyonacean occurrence data from near

the U.S. Gulf of Maine (Mortensen and Buhl-Mortensen 2004; Mortensen et al. 2006; Buhl-Mortensen et al. 2015).

Depth is clearly a factor that significantly influences occurrences and distributions of alcyonaceans (Bakeret al. 2012; Guinotte and Davies 2014; Kinlan et al. 2020). For example, in the Northeast, most alcyonacean species occur >500 m, while at least three of the species that occur in the Gulf of Maine (*Primnoa*, *Paramuricea*, *Paragorgia*) also occur on other parts of the shelf and upper continental slope at <500 m (Watling and Auster 2005; Packer et al. 2007; Packer et al. 2017a). Quattrini et al. (2015) also found that although species richness did not change significantly with depth over the range of their Northeast surveys (494-3271 m), species composition did change at ~1600-1700 m. Species compositionin the canyons and other areas with hard substrates were significantly dissimilar across this depth boundary.

As mentioned previously, in the Gulf of Maine, seafloor topography and the presence of hard substrate have considerable influence on the occurrences and distribution of the gorgonians; this is also true of gorgonians elsewhere (Packer et al. 2007; Edinger et al. 2011; Baker et al. 2012; Auster et al. 2013a; Auster et al. 2013b; Auster et al. 2014; Packer et al. 2017a). For example, the Northeast region habitat suitability model of Kinlan et al. (2020) showed that rugosity (at the 1500 m scale) was an important environmental predictor for alcyonaceans, while in the Northeast Channel off Georges Bank, Canada, abundances of *Primnoa* and *Paragorgia* were positively correlated with cobble substrate (Mortensen andBuhl-Mortensen 2004).

Another topographic feature that can affect the occurrence and distribution of deep-sea corals is slope (e.g., Yesson et al. 2012; Rengstorf et al. 2013; Guinotte and Davies 2014; Morato et al. 2020 [specifically for the solitary stony cup coral *Desmophyllum* and gorgonian alcyonacean *Acanthogorgia*]) often at broader scales (Jones et al. 2009; Kinlan et al. 2020). For example, Kinlan et al. (2020) found thatin the Northeast region broad-scale slope and slope of slope (5 and 1.5 km scales, respectively) were identified as important environmental predictor variables for alcyonaceans, and large structure-forming taxa have been successfully predicted to occur mainly in canyon environments, particularly in areas of steep (>30°) slope. Furthermore, these areas almost always contain hard-bottom habitat (MAFMC and NMFS 2016). Slope appears to be important for alcyonacean distribution in the Gulf of Maine as well (Auster et al. 2014; Packer et al. 2017a) and has been shown to be one of the predictors of the overall distribution of the alcyonaceans *Paragorgia* and *Primnoa* (Bryan and Metaxas 2006; Bryan and Metaxas 2007). For example, on the Scotian Shelf, *Paragorgia* is found on steeper slopes than *Primnoa* (Bryan and Metaxas 2006). In the U.S. Gulf of Maine, where *Paragorgia* is scarce, *Primnoa* is found on steeper slopes and walls than the more common *Paramuricea* (Auster et al. 2014; Packer et al. 2017a). However, in general slope may be considered as a proxy for the distribution of hard substrates (Bryan and Metaxas 2007), and also for areas that accelerate and direct tidal currents which then transport food or nutrients to deep-sea corals as well affecting larval supply (e.g., the colonial stony coral *Lophelia*: Genin et al. 1986; Frederiksen et al. 1992; Mortensen et al. 2001; Thiem et al. 2006). Of course, the relationships between topography, currents, and the availability of food and larvae have been recognized as important factors in determining deep-sea coral distributions and suitable deep-sea coral habitat (Genin et al. 1986; Mortensen et al. 2001). Topography as well as location also has an effect on deep-sea coral reproductive output (for the latter there is geographic variability in reproductive output of *Paramuricea* and *Primnoa* in the Gulfof Maine: Fountain et al. 2019), hydrodynamic connectivity of deep-sea coral habitats (Corsair Canyon off Georges Bank: Metaxas et al. 2019) and genetic connectivity (Northwest Atlantic seamounts: Thoma et al. 2009).

Current strength is one factor that controls the distributions and abundances of *Paragorgia* and *Primnoa* in Atlantic Canada (Mortensen and Buhl-Mortensen 2004; Mortensen and Buhl-Mortensen 2005; Bryan and Metaxas 2006; Bryan and Metaxas 2007). In addition, food availability can be measured by either POC flux to the bottom or chlorophyll-*a* concentration (as a proxy for surface primary productivity), and several studies show its importance for both alcyonaceans and scleractinians (POC: White et al. 2005; Davies and Guinotte 2011; Yesson et al. 2012; Morato et al. 2020; chlorophyll-*a*: Huff et al. 2013; Knudby et al. 2013). In their modeling studies, Bryan and Metaxas (2006) and Bryan and Metaxas (2007)found that *Primnoa* and *Paragorgia* tend to occur in areas of low chlorophyll-*a* concentrations (< 3.0 mg m^{-3}) because deep-sea coral habitats tend to be found under oligotrophic areas. However, deep-sea corals may not need to be situated under high productivity areas in order to obtain sufficient nutrition, so long as there are adequate levels of

laterally advected POC into their habitat (Smith and Kaufmann 1999). Obviously, lack of adequate nutrition negatively affects coral physiology and condition (*Desmophyllum*: Naumann et al. 2011; *Lophelia*: Larsson et al. 2013; Büscher et al. 2017).

Bottom salinity is another factor that may determine alcyonacean habitat suitability (Mortensen et al. 2006; Yesson et al. 2012; Guinotte and Davies 2014; Quattrini et al. 2015; Kinlan et al. 2020), especially given the relatively consistency of the Gulf of Maine bottom water salinity (~33-34 ppt.) (Hopkins and Garfield 1979), and thus the narrow salinity range in which deep-sea corals would occur (Kinlan et al. 2020).

Finally, a few studies have shown dissolved oxygen to be another predictor variable for alcyonacean and scleractinian habitat suitability (scleractinians: Tittensor et al. 2010; alcyonaceans: Yesson et al. 2012; Quattrini et al. 2015). Climate change is expected to produce ocean deoxygenation which will become a major stressor for deep-sea corals (Sweetman et al. 2017). However, even though exposure to lowered concentrations of dissolved oxygen may prove detrimental or fatal to *Lophelia*, for example (Lunden et al. 2014), nevertheless there are some areas where *Lophelia* is currently thriving under both hypoxic conditions and warmer temperatures (e.g., Southeast Atlantic), thus denoting a tolerance for and the ability to adapt to extreme conditions, which may be facilitated by high surface ocean productivity (Hebbeln et al. 2020). These perhaps population-specific adaptations should be taken into consideration when trying to predict its future distributions (Hebbeln et al. 2020).

Literature Cited

Auster PJ. 2005. Are deep-water corals important habitats for fishes? *In*: Freiwald A, Roberts A (eds). Cold-water corals and ecosystems. Berlin, Heidelberg: Springer-Verlag. 643-56.

Auster PJ, Kilgour M, Packer D, Waller R, Auscavitch S, Watling L. 2013a. Octocoral gardens in the Gulf of Maine (NW Atlantic). Biodiversity 14:193.

Auster PJ, Packer D, Kilgour M, Watling L. 2013b. Supplementary comment: conservation of deep-sea corals off the northeast United States. Biodiversity 14(4):195. DOI:10.1080/14888386.2013.850885

Auster PJ, Packer D, Waller R, Auscavitch S, Kilgour MJ, Watling L, Nizinski MS, Babb I, Johnson D, Pessutti J, Drohan A, Kinlan B. 2014. Imaging surveys of select areas in the northern Gulf of Maine for deep-sea corals and sponges during 2013-2014. Report to the New England Fishery Management Council - 1 December 2014. DOI: 10.13140/RG.2.1.4760.0163

Baker KD, Wareham VE, Snelgrove PVR, Haedrich RL, Fifield DA, Edinger EN, Gilkinson KD. 2012. Distributional patterns of deep-sea coral assemblages in three submarine canyons off Newfoundland, Canada. Marine Ecology Progress Series 445:235-49. https://doi.org/10.3354/meps09448

Bryan TL, Metaxas A. 2006. Distribution of deep-water corals along the North American continental margins: relationships with environmental factors. Deep-Sea Research I53:1865–79.

Bryan TL, Metaxas A. 2007. Predicting suitable habitat for deep-water gorgonian corals on the Atlantic and Pacific Continental Margins of North America. Marine Ecology Progress Series 330:113–26.

Buhl-Mortensen L, Olafsdottir SH, Buhl-Mortensen P, Burgos JM, Ragnarsson SA. 2015. Distribution of nine cold-water coral species (Scleractinia and Gorgonacea) in the cold temperate North Atlantic: Effects of bathymetry and hydrography. Hydrobiologia 759(1):39–61. https://doi.org/10.1007/s10750-014-2116-x

Büscher JV, Form AU, Riebesell U. 2017. Interactive effects of ocean acidification and warming on growth, fitness and survival of the cold-water coral *Lophelia pertusa* under different food availabilities. Frontiers in Marine Science 4:101. https://doi.org/10.3389/fmars.2017.00101

Büscher JV, Wisshak M, Form AU, Titschack J, Nachtigall K, Riebesell U. 2019. In situ growth and

bioerosion rates of *Lophelia pertusa* in a Norwegian fjord and open shelf cold-water coral habitat. PeerJ 7:e7586. https://doi.org/10.7717/peerj.7586

Cerrano C, Cardini U, Bianchelli S, Corinaldesi C, Pusceddu A, Danovaro R. 2013. Red coral extinction risk enhanced by ocean acidification. Scientific Reports 3:1457. https://doi.org/10.1038/srep01457

Davies A J, Guinotte JM. 2011. Global habitat suitability for framework-forming cold-water corals. PLoS ONE 6(4):e18483. https://doi.org/10.1371/journ al.pone.0018483

Edinger EN, Sherwood OA, Piper DJW, Wareham VE, Baker KD, Gilkinson KD, Scott DB. 2011. Geologic features supporting deep-sea coral habitat in Atlantic Canada. Continental Shelf Research 31:S69-S84. 10.1016/j.csr.2010.07.004

Fountain CT, Waller RG, Auster PJ. 2019. Individual and population level variation in the reproductive potential of deep-sea corals from different regions within the Gulf of Maine. Frontiers in Marine Science 6:172. doi:10.3389/fmars.2019.00172

Frederiksen R, Jensen A, Westerberg H. 1992. The distribution of the scleractinian coral *Lophelia pertusa* around the Faroe Islands and the relation to internal tidal mixing. Sarsia 77:157–71.

Genin A, Dayton PK, Lonsdale PF, Spiess FN. 1986. Corals on seamount peaks provide evidence of current acceleration over deep-sea topography. Nature 322(6074):59–61. https://doi.org/10.1038/322059a0

Gomez CE, Wickes L, Deegan D, Etnoyer PJ, Cordes EE. 2019. Growth and feeding of deep-sea coral *Lophelia pertusa* from the California margin under simulated ocean acidification conditions. PeerJ 6:e5671. https://doi.org/10.7717/peerj.5671

Guinotte JM, Davies AJ. 2014. Predicted deep-sea coral habitat suitability for the US West Coast. PLoS ONE 9(4):e93918. https://doi.org/10.1371/journal.pone.0093918

Guinotte, JM, Orr J, Cairns S, Freiwald A, Morgan L, and George R. 2006. Will human-induced changes in seawater chemistry alter the distribution of deep-sea scleractinian corals? Frontiers in Ecology and the Environment, 4(3):141–6. https://doi.org/10.1890/1540-9295

Hebbeln D, Wienberg C, Dullo W-C, Freiwald A, Mienis F, Orejas C, Titschack J. 2020. Cold-water coral reefs thriving under hypoxia. Coral Reefs 39:853–9. https://doi.org/10.1007/s00338-020-01934-6

Hennige SJ, Wicks LC, Kamenos NA, Perna G, Findlay HS, Roberts JM. 2015. Hidden impacts of ocean acidification to live and dead coral framework. Proceedings of the Royal Society B: Biological Sciences 282(1813):20150990. https://doi.org/10.1098/rspb.2015.0990

Hopkins TS, Garfield III N. 1979. Gulf of Maine Intermediate Water. Journal of Marine Research 37(1):103-39.

Huff DD, Yoklavich MM, Love MS, Watters DW, Chai F, Lindley ST. 2013. Environmental factors that influence the distribution, size, and biotic relationships of the Christmas tree coral *Antipathes dendrochristos* in the Southern California Bight. Marine Ecology Progress Series 494:159–77. https://doi.org/10.3354/meps10591

Jackson EL, Davies AJ, Howell KL, Kershaw PJ, Hall-Spencer JM. 2014. Future-proofing marine protected area networks for cold water coral reefs. ICES Journal of Marine Science 71(9):2621–29. https://doi.org/10.1093/icesj ms/fsu099

Jones K, Devillers R, Edinger E. 2009. Relationships between cold-water corals off Newfoundland and Labrador and their environment. In: Sieber RE (ed.). Proceedings of the 2009 Spatial Knowledge and

Information Canada Conference, February 19-22. Fernie BC, Canada. Vol. 1:15-20.

Kinlan BP, Poti M, Drohan AF, Packer DB, Dorfman DS, Nizinski MS. 2020. Predictive modeling of suitable habitat for deep-sea corals offshore the Northeast United States. Deep Sea Research Part I: Oceanographic Research Papers 158:103229. https://doi.org/10.1016/j.dsr.2020.103229.

Knudby A, Lirette C, Kenchington E, Murillo FJ. 2013. Species distribution models of black corals, large gorgonian corals and sea pens in the NAFO regulatory area. NAFO Scientific Council Research Document 13/078, Serial Number N6276.

Larsson AI, Lundälv T, van Oevelen D. 2013. Skeletal growth, respiration rate and fatty acid compositionin the cold-water coral *Lophelia pertusa* under varying food conditions. Marine Ecology Progress Series 483:169–84. https://doi.org/10.3354/meps1 0284

Lunden JJ, McNicholl CG, Sears CR, Morrison CL, Cordes EE. 2014. Acute survivorship of the deep-sea coral *Lophelia pertusa* from the Gulf of Mexico under acidification, warming, and deoxygenation. Frontiers in Marine Science 1(78):1-12. https://doi.org/10.3389/fmars.2014.00078

Metaxas A, Lacharité M, Natasha De Mendonca S. 2019. Hydrodynamic connectivity of habitats of deep-water corals in Corsair Canyon, Northwest Atlantic: a case for cross-boundary conservation. Frontiers in Marine Science 6:159. doi: 10.3389/fmars.2019.00159

Mid-Atlantic Fisheries Management Council and National Marine Fisheries Service (MAFMC and NMFS). 2016. Amendment 16 to the Atlantic mackerel, squid, and butterfish Fishery Management Plan. Measures to protect deep sea corals from impacts of fishing gear. Environmental assessment,regulatory impact review, and initial regulatory flexibility analysis. https://www.mafmc.org/s/DeepSea-Corals-EA_Signed-FONSI.pdf

Morato T, González-Irusta JM, Dominguez-Carrió C, Wei C-L, Davies A, Sweetman AK, Taranto GH, Beazley L et al. 2020. Climate-induced changes in the suitable habitat of cold-water corals and commercially important deep-sea fishes in the North Atlantic. Global Change Biology 26:2181-202. https://doi.org/10.1111/gcb.14996

Mortensen PB, Buhl-Mortensen L. 2004. Distribution of deep-water gorgonian corals in relation to benthic habitat features in the Northeast Channel (Atlantic Canada). Marine Biology 144:1223–38.

Mortensen PB, Buhl-Mortensen L. 2005. Morphology and growth of the deep-water gorgonians *Primnoa resedaeformis* and *Paragorgia arborea*. Marine Biology 147:775–88.

Mortensen PB, Buhl-Mortensen L, Gordon DC. 2006. Distribution of deep-water corals in Atlantic Canada. *In*: Proceedings of the 10th International Coral Reef Symposium:1832–48.

Mortensen PB, Hovland T, Fosså JH, Furevik DM. 2001. Distribution, abundance and size of *Lophelia pertusa* coral reefs in mid-Norway in relation to seabed characteristics. Journal of the Marine Biological Association UK 81:581–97.

Naumann MS, Orejas C, Wild C, Ferrier-Pagès C. 2011. First evidence for zooplankton feeding sustaining key physiological processes in a scleractinian cold-water coral. Journal of Experimental Biology 214(21):3570–76. https://doi.org/10.1242/jeb.061390

National Oceanic and Atmospheric Administration (NOAA) Northeast Fisheries Science Center and University of Connecticut. 2015. [Internet]. Coral and sponge occurrence observations submitted to the NOAA National Database for Deep Sea Corals. (Accessed 8 Dec. 2020). https://deepseacoraldata.noaa.gov/.

Orr JC, Fabry VJ, Aumont O, Bopp L, Doney SC, Feely RA, Gnanadesikan A, Gruber N, Ishida A, Joos F, Key RM, Lindsay K, Maier-Reimer E, Matear R, Monfray P, Mouchet A, Najjar RG, Plattner G-K, Rodgers KB, Sabine CL, Sarmiento JL, Schlitzer R, Slater RD, Totterdell IJ, Weirig M-F, Yamanaka Y, Yool A. 2005. Anthropogenic ocean acidification over the twenty-first century and its impact on calcifying organisms. Nature 437(7059):681-6. https://www.nature.com/articles/nature04095

Packer DB, Boelke D, Guida V, McGee L-A. 2007. State of deep coral ecosystems in the northeastern US region: Maine to Cape Hatteras. In: Lumsden SE, Hourigan TF, Bruckner AW, Dorr G (eds). The state of deep coral ecosystems of the United States. NOAA Technical Memorandum CRCP-3. 195-232.

Packer DB, Nizinski MS, Bachman MS, Drohan AF, Poti M, Kinlan BP. 2017a. State of the deep-sea coral and sponge ecosystems off the Northeast United States. In: Hourigan T, Etnoyer PJ, Cairns SD (eds). The state of deep-sea coral and sponge ecosystems of the United States. NOAA Technical Memorandum NMFS-OHC-4. Silver Spring, MD. 237-97. https://deepseacoraldata.noaa.gov/library/resolveuid/95df948cc33444b7bea34ae78db8b600

Packer D, Nizinski MS, Cairns SD, Hourigan TF. 2017b. [Internet]. Deep-sea coral taxa in the U.S. Northeast Region: depth and geographical distribution. (Accessed 8 Dec. 2020). https://deepseacoraldata.noaa.gov/library/2017-state-of-deep-sea-corals-report

Packer D, Nizinski M, Metaxas A, Kentner T. 2019. Northern Neighbors: Exploring deepwater communities in the transboundary U.S. Gulf of Maine, 2019. Canadian ROV *ROPOS*/NOAA Ship *Henry B Bigelow*. Talk presented to the NOAA Deep-sea Coral Research and Technology Program,November 21, 2019.

Quattrini AM, Nizinski MS, Chaytor JD, Demopoulos AWJ, Roark EB, France SC, Moore, JA, Heyl T, Auster PJ, Kinlan B, Ruppel C, Elliott KP, Kennedy BRC, Lobecker E, Skarke A, Shank TM. 2015. Exploration of the canyon-incised continental margin of the Northeastern United States reveals dynamic habitats and diverse communities. PLoS ONE 10(10):e0139904. DOI:10.1371/journal.pone.0139904

Rengstorf AM, Yesson C, Brown C, Grehan AJ. 2013. High resolution habitat suitability modelling can improve conservation of vulnerable marine ecosystems in the deep sea. Journal of Biogeography 40(9):1702–14. https://doi.org/10.1111/jbi.12123

Roberts JM, Murray F, Anagnostou E, Hennige S, Gori A, Henry LA, Fox A, Kamenos N, Foster GL. 2016. Cold-water corals in an era of rapid global change: Are these the deep ocean's most vulnerable ecosystems? In: Goffredo S, Dubinsky Z (eds) The Cnidaria, past, present and future (pp. 593–606). Springer: Cham. 593-606. https://doi.org/10.1007/978-3-319-31305-4_36

Smith Jr. KL, Kaufmann RS. 1999. Long-term discrepancy between food supply and demand in the deep eastern North Pacific. Science 284:1174–7.

Sweetman AK, Thurber AR, Smith CR, Levin LA, Mora C, Wei C-L, Gooday AJ, Jones DOB, Rex M, Yasuhara M, Ingels J, Ruhl HA, Frieder CA, Danovaro R, Würzburg L, Baco A, Grupe BM, Pasulka A, Meyer KS, Dunlop KM, Henry L-A, Roberts JM. 2017. Major impacts of climate change on deep-sea benthic ecosystems. Elementa-Science of the Anthropocene 5:1–23. http://doi.org/10.1525/elementa.203

Thiem Ø, Ravagnan E, Fossa JH, Berntsen J. 2006. Food supply mechanisms for cold-water corals along a continental shelf edge. Journal of Marine Systems 60:207–19. https://doi.org/10.1016/j.jmarsys.2005.12.004.

Thoma JN, Pante E, Brugler MR, France SC. 2009. Deep-sea octocorals and antipatharians show no evidence of seamount-scale endemism in the NW Atlantic. Marine Ecology Progress Series 397:25-35.

Thresher RE, Tilbrook B, Fallon S, Wilson NC, Adkins J. 2011. Effects of chronic low carbonate saturation

levels on the distribution, growth and skeletal chemistry of deep-sea corals and other seamount megabenthos. Marine Ecology Progress Series 442:87–99. https://doi.org/10.3354/meps09400

Tittensor DP, Baco AR, Hall-Spencer JM, Orr, JC, Rogers, AD. 2010. Seamounts as refugia from ocean acidification for cold-water stony corals. Marine Ecology 31:212–25.

Watling L, Auster P. 2005. Distribution of deep-water Alcyonacea off the northeast coast of the United States. In: Freiwald A, Roberts A (eds). Cold-water corals and ecosystems. Berlin, Heidelberg: Springer-Verlag. 259-64.

White M, Mohn C, de Stigter H, Mottram G. 2005. Deep-water coral development as a function of hydrodynamics and surface productivity around the submarine banks of the Rockall Trough, NE Atlantic. *In*: Freiwald A, Roberts A (eds). Cold-water corals and ecosystems. Berlin, Heidelberg:Springer-Verlag. 503–14.

Wigley RL. 1968. Benthic invertebrates of the New England fishing banks. Underwater Naturalist 5:8-13.

Yesson C, Taylor ML, Tittensor DP, Davies AJ, Guinotte J, Baco A, Black J, Hall-Spencer JM, Rogers AD. 2012. Global habitat suitability of cold-water octocorals. Journal of Biogeography 39:1278–92. https://doi.org/10.1111/j.1365-2699.2011.02681.x

Zheng MD, Cao L. 2014. Simulation of global ocean acidification and chemical habitats of shallow- and cold-water coral reefs. Advances in Climate Change Research 5(4):189–96. https://doi.org/10.1016/j.accre.2015.05.002.

Deep Sea Coral and Sponge: Seamounts and Canyons

System: Marine

Subsystem: Subtidal >150m

Class: Reef

Sub-class: Deep Sea Coral and Sponge

Geographic Area: Seamounts and Canyons

Overall Vulnerability Rank = Very High

Habitat Sensitivity = Very High

Climate Exposure = High

	Deep sea coral and sponge: seamounts and canyons	Attribute Mean	Data Quality	Distribution of Expert Scores
Sensitivity Attributes	Habitat condition	2.4	1.8	
	Habitat fragmentation	2	2.2	
	Distribution/Range	1.9	2.2	
	Mobility/Ability to spread or disperse	2.9	1.8	
	Resistance	3.6	2.2	
	Resilience	3.6	2.2	
	Sensitivity to changes in abiotic factors	3.6	2.1	
	Sensitivity and intensity of non−climate stressors	3.2	1.9	
	Dependency on critical ecological linkages	3.2	1.8	
	Sensitivity Component Score	Very High		
Exposure Factors	Sea surface temp	n/a	n/a	
	Bottom temp	2.2	3	
	Air temp	n/a	n/a	
	River temp	n/a	n/a	
	Surface salinity	n/a	n/a	
	Bottom salinity	3.3	2.6	
	pH	4	2.4	
	Sea level rise	n/a	n/a	
	Precipitation	n/a	n/a	
	Floods	n/a	n/a	
	Droughts	n/a	n/a	
	Exposure Component Score	High		
**Overall Vulnerability Rank**		**Very High**		

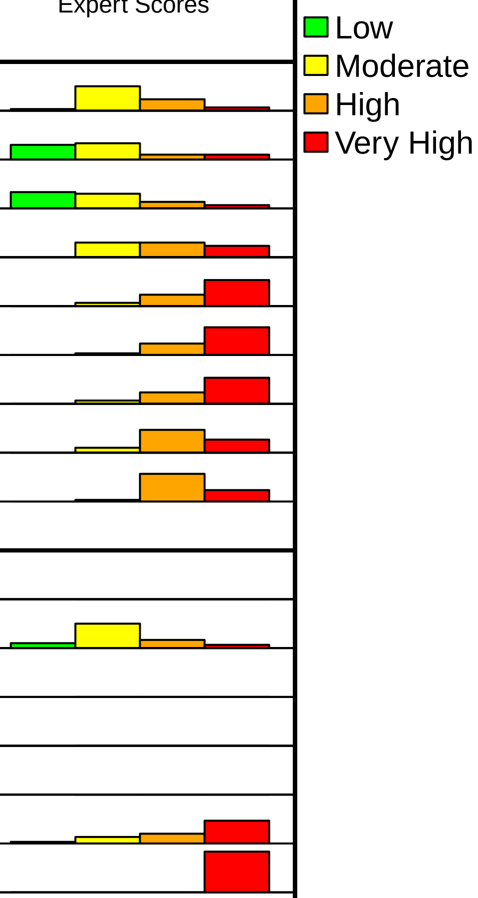

Habitat Name: Deep-sea Coral and Sponge: Seamounts and Canyons

System**:** Marine

Subsystem: Subtidal >150 m

Class: Reef

Sub-class: Deep Sea Coral and Sponge

Geographic Area: Seamounts and Canyons

Habitat Description: For the purposes of this study, the geographic areas for this habitat sub-class include the continental shelf outside of the Gulf of Maine, slope, submarine canyons, and the four seamounts (within the EEZ) from Georges Bank to Cape Hatteras containing corals/sponges or coral/sponge habitats at depths >150 m. The types of corals and sponges that occur here include stony corals (scleractinians, both solitary and colonial), true soft corals and gorgonians (alcyonaceans), sea pens(also alcyonaceans, but occur only on soft sediments[1]), black corals, glass sponges, and demosponges (Packer et al. 2007; Packer et al. 2017a; Packer et al. 2017b).

These habitats and communities are often hotspots of biological diversity and provide essential habitat for several commercially important species. Because deep-sea corals and sponges are slow-growing, long lived (often for hundreds of years) (Lazier et al. 1999; Andrews et al. 2002; Risk et al. 2002), and for corals especially, often have complex, branching forms of growth that makes them very fragile, they are sensitive to disturbance and highly vulnerable to human impacts (such as from fishing gear). Recovery potential of deep-sea coral habitats is extremely low over time periods of years to decades (NOAA 2010).

Overall Climate Vulnerability Rank: Very High (41% certainty from bootstrap analysis). The majority (58%) of the bootstrap results were in the High scoring bin, which differs from the Very High categorical vulnerability rank. This indicates that the vulnerability rank is on the borderline between High and Very High. This result is due to three sensitivity attributes (i.e., Resistance, Resilience, and Sensitivity to Changes in Abiotic Factors) scoring just above the Very High threshold. The bootstrap results therefore indicate that a small change in the distribution of the expert opinion tallies could have led to a High categorical rank instead of a Very High.

Climate Exposure: Moderate. The highest exposure scores were for pH (4.0), Bottom Salinity (3.3) and Bottom Temperature (2.2). The projected change in pH for southern New England and the Mid-Atlantic is a significant decrease (>5.5 standard deviation) from historic levels.

Habitat Sensitivity: Very High. Five of the nine sensitivity attributes scores were ≥3.0, with Resistance, Resilience, and Sensitivity to Changes in Abiotic Factors scoring the highest at 3.6. Corals and coral/sponge habitats are fragile communities that take a long time to recover from impacts and disturbance. As a deep-sea habitat, they are very sensitive to changes in abiotic factors such as temperature and carbonate chemistry. For the lower scoring attributes: Mobility/Ability to Spread or Disperse scored 2.9, Habitat Condition scored 2.4, Habitat Fragmentation scored 2.0, and Distribution/Range scored 1.9.

Data Quality & Gaps: For the data quality scores for climate exposure factors, pH and Bottom Salinity scored Moderate (2.4 and 2.6, respectively), and Bottom Temperature scored High (3.0). The relative Low data quality score for pH is likely reflecting some uncertainty in how the CMIP5 projected changes in surface pH will affect the pH and calcium carbonate ($CaCO_3$) saturation levels in deeper water. The data quality scores for sensitivity were all relatively Low: 1.8 –2.2. Habitat Condition, Mobility/Ability to Spread or Disperse, Sensitivity and Intensity of Non-Climate Stressors, and Dependency on Critical Ecological Linkages all scored <2.0.

[1] When alcyonaceans are being discussed in this document, it is generally referring to only soft corals and gorgonians.

In spite of the recent spate of deep-sea coral and sponge surveys and fieldwork both on and off the Northeast shelf, our knowledge of the temporal and spatial distribution and abundance of these organisms and habitats, as well as many aspects of their basic biology and habitat requirements, is still severely limited. In many cases, our knowledge is confined to simple presence or absence data.

Positive or Negative Climate Effect in the Northeast U.S. Shelf: The effects of climate change on deep-sea corals and sponges on and off the Northeast shelf is expected to be negative (85% of the experts' scores were negative, 15% were neutral).

Climate Effects on Habitat Condition and Distribution: Ocean acidification is a major threat to deep-sea corals worldwide, with a potential significant loss of deep-sea coral habitat and the ecosystem services they provide (Orr et al. 2005; Guinotte et al. 2006; Tittensor et al. 2010; Thresher et al. 2011; Jackson et al. 2014; Zheng and Cao 2014; Roberts et al. 2016; Morato et al. 2020;). Some of the major groups of corals that have been shown to be susceptible to the effects of ocean acidification and which also occur off the Northeast shelf include the colonial stony corals (e.g., *Lophelia pertusa*) (Lunden et al. 2014; Hennige et al. 2015; Büscher et al. 2019; Gomez et al. 2019; Morato et al. 2020) and alcyonaceans (Cerrano et al. 2013; Morato et al. 2020). However, it should also be noted that *Lophelia* and some other colonial structure-forming scleractinians as well as the solitary stony coral *Desmophyllum dianthus* have been shown to be fairly resistant to acidification (Form and Riebesell 2012; Maier et al. 2012; Maier et al.2013; Hennige et al. 2014; Movilla et al. 2014; Hennige et al. 2015; Büscher et al. 2017), except perhaps when subject to multiple stressors (e.g., *Desmophyllum*: Carreiro-Silva et al. 2014; Gori et al. 2016; *Lophelia*: Hennige et al. 2014; *Madrepora*: Maier et al. 2016).

Recently, Morato et al. (2020) utilized environmental niche modelling, species occurrence data, and environmental parameters to model habitat suitability for two stony coral species (the colonial *Lophelia pertusa* and the solitary *Desmophyllum dianthus*) and three alcyonaceans (*Acanthogorgia armata*, *Acanella arbuscula*, and *Paragorgia arborea*) that occur in the North Atlantic, including off the Northeast shelf. They modeled under present-day environmental conditions (years 1951-2000) and a projected severe, high emissions future (years 2081-2100; RCP8.5 or "business-as-usual" scenario). The authors concluded that three predictors acting in concert: ocean warming (bottom water temperature), acidification (aragonite or calcite saturation), and decreasing food availability (as POC flux to the seafloor) will change the availability of suitable habitat for the aforementioned deep-sea corals.

Specifically, off the Northeast shelf, predicted losses of scleractinian habitat was linked to warming of deeper waters, while projected losses of alcyonacean habitat was linked to acidification (i.e., shoaling of the calcite saturation horizon). Their modeling results suggest that overall, suitable habitat for *L. pertusa* may be reduced in the North Atlantic by over 79%, with a projected shift towards deeper waters resulting from loss of habitat. *D. dianthus* may lose approximately 30-45% of its habitat. But the results also predicted limited climate refugia locations for scleractinian by 2100 of between 30-42% of present-day habitat. The three alcyonacean species are not expected to survive the projected scenario, with an overall reduction of suitable North Atlantic habitat of >80%, and *P. arborea* having at least a 99% reduction of suitable habitat. The refugia locations projected for *A. arbuscula* and *A. armata* are only 6-14% of present-day habitat, with *P. arborea* projected to have almost no refugia. *A. arbuscula* and *A. armata* are also projected to shift toward shallower depths.

Bottom salinity is another factor that may determine scleractinian and alcyonacean habitat suitability (Mortensen et al. 2006; Davies and Guinotte 2011; Yesson et al. 2012; Guinotte and Davies 2014; Quattrini et al. 2015; Kinlan et al. 2020) especially given the relative consistency of deep ocean bottom salinity (~34-35 ppt.) and thus the narrow salinity range in which deep-sea corals would occur (Kinlan et al. 2020). According to the ROMS-NWA climate model, bottom salinity is projected to significantly increase along the continental shelf break by the end of the century, which may limit habitat suitability for deep-sea corals (NOAA Physical Sciences Laboratory 2020).

Habitat Summary: Depending on the study, there are any number of habitat parameters either alone or in combination that have been found to be important predictors of habitat suitability for, or may determine the distribution of, deep-sea corals. As one example, Quattrini et al. (2015) surveyed 11 Northeast submarine canyons, as well as intercanyon and slope sites, plus the Mytilus Seamount, and found that depth, habitat, salinity, and dissolved oxygen together explained 71% of the total variation in deep-sea coral assemblage structure, and the type of broad-scale habitat feature and high habitat heterogeneity in this region was an important factor that influenced the diversity of coral assemblages.

Examining some of the individual parameters across several studies, aragonite and calcite saturation rates, as discussed above (Morato et al. 2020), are an important factor in determining habitat suitability for both reef forming scleractinians (Tittensor et al. 2010 [on seamounts]; Davies & Guinotte 2011) and alcyonaceans (Yesson et al. 2012). Another key variable across a variety of studies is temperature, for both scleractinians (Mortensen et al. 2006; Davies and Guinotte 2011; Guinotte and Davies 2014; Buhl-Mortensen et al. 2015; Morato et al. 2020) and alcyonaceans (Mortensen and Buhl-Mortensen 2004;Bryan and Metaxas 2006; Mortensen et al. 2006; Bryan and Metaxas 2007; Yesson et al. 2012; Guinotte and Davies 2014; Buhl-Mortensen et al. 2015; Morato et al. 2020).

Depth is clearly a factor that significantly influences occurrences and distributions of both scleractinians and alcyonaceans (Davies and Guinotte 2011; Baker et al. 2012; Guinotte and Davies 2014; Kinlan et al.2020.) For example, in the Northeast, most alcyonacean species occur >500 m, while three major taxa (*Primnoa*, *Paramuricea*, *Paragorgia*) also occur on the shelf and upper continental slope at <500 m (Watling and Auster 2005; Packer et al. 2017a). Quattrini et al. (2015) also found that although species richness did not change significantly with depth over the range of their Northeast surveys (494-3271 m depth), species composition did change at ~1600-1700 m. Species composition in the canyons and other areas with hard substrates were significantly dissimilar across this depth boundary.

Since most scleractinians and alcyonaceans (other than alcyonacean sea pens) are restricted to hard substrates, both large and small hard-bottom geologic and topographic features have considerable influence on their distributions and abundances (Packer et al. 2007; Edinger et al. 2011; Baker et al. 2012;Packer et al. 2017a). The habitat suitability model for the Northeast region of Kinlan et al. (2020), for example, showed that percent gravel and rugosity (at the 1500 m scale) were important environmental predictors for scleractinians and alcyonaceans, respectively, while in the Northeast Channel off Georges Bank, Canada, abundances of *Primnoa* and *Paragorgia* were positively correlated with cobble substrate (Mortensen and Buhl-Mortensen 2004).

Another topographic feature that can affect the occurrence and distribution of alcyonacean and scleractinian colonies is slope (e.g., Yesson et al. 2012; Rengstorf et al. 2013; Guinotte and Davies 2014; Morato et al. 2020 [specifically for the solitary cup coral *Desmophyllum* and gorgonian alcyonacean *Acanthogorgia*]) often at broader scales (Jones et al. 2009; Kinlan et al. 2020). For example, Kinlan et al.(2020) found that in the Northeast region broad-scale slope and slope of slope (5 and 1.5 km scales, respectively) were identified as important environmental predictor variables for alcyonaceans, and large structure-forming taxa have been successfully predicted to occur mainly in canyon environments, particularly in areas of steep (>30°) slope . Furthermore, these areas almost always contain hard-bottom habitat (MAFMC and NMFS 2016). Slope is one of the predictors of the distribution of the alcyonaceans *Paragorgia* and *Primnoa* (Bryan and Metaxas 2006; Bryan and Metaxas 2007), with *Paragorgia* being found on steeper slopes than *Primnoa* on the Scotian Shelf (Bryan and Metaxas 2006). However, in general slope may be considered as a proxy for the distribution of hard substrates (Bryan and Metaxas 2007) and also for areas that accelerate and direct tidal currents which then transport food or nutrients to deep-sea corals as well affecting larval supply (e.g., *Lophelia*: Genin et al. 1986; Frederiksen et al. 1992;Mortensen et al. 2001; Thiem et al. 2006). Of course, the relationships between topography, currents, and the availability of food and larvae have been recognized as important factors in determining deep-sea coral distributions and suitable deep-sea coral habitat (Genin et al. 1986; Mortensen et al. 2001). Topography as well as location also has an effect on deep-sea coral reproductive output (Gulf of Maine: Fountain et al. 2019), hydrodynamic connectivity of deep-sea coral habitats (Corsair Canyon off Georges Bank: Metaxas et al. 2019) and genetic connectivity (Northwest Atlantic seamounts: Thoma et al.

2009).

Current strength is one factor that controls the distributions and abundances of *Paragorgia* and *Primnoa* in Atlantic Canada (Mortensen and Buhl-Mortensen 2004; Mortensen and Buhl-Mortensen 2005; Bryan and Metaxas 2006; Bryan and Metaxas 2007). In addition, food availability can be measured by either POC flux to the bottom or chlorophyll-*a* concentration (as a proxy for surface primary productivity) and several studies show its importance for both alcyonaceans and scleractinians (POC: White et al. 2005; Davies and Guinotte 2011; Yesson et al. 2012; Morato et al. 2020; chlorophyll-*a*: Huff et al. 2013; Knudby et al. 2013). In their modeling studies, Bryan and Metaxas (2006) and Bryan and Metaxas (2007) found that *Primnoa* and *Paragorgia* tend to occur in areas of low chlorophyll-*a* concentrations (<3.0 mg m^{-3}) because deep-sea coral habitats tend to be found under oligotrophic areas. However, deep-sea corals may not need to be situated under high productivity areas in order to obtain sufficient nutrition, so long as there are adequate levels of laterally advected POC into their habitat (Smith and Kaufmann 1999). Obviously, lack of adequate nutrition negatively affects coral physiology and condition (*Desmophyllum*:Naumann et al. 2011; *Lophelia*: Larsson et al. 2013; Büscher et al. 2017).

Finally, a few studies have shown dissolved oxygen to be another predictor variable for alcyonacean and scleractinian habitat suitability (scleractinians: Tittensor et al. 2010; alcyonaceans: Yesson et al. 2012; Quattrini et al. 2015). Climate change is expected to produce ocean deoxygenation which will become a major stressor for deep-sea corals (Sweetman et al. 2017). However, even though exposure to lowered concentrations of DO may prove detrimental or fatal to *Lophelia*, for example (Lunden et al. 2014), nevertheless there are some areas where *Lophelia* is currently thriving under both hypoxic conditions and warmer temperatures (e.g., Southeast Atlantic), thus denoting a tolerance for and the ability to adapt to extreme conditions, which may be facilitated by high surface ocean productivity (Hebbeln et al. 2020). These perhaps population-specific adaptations should be taken into consideration when trying to predict its future distributions (Hebbeln et al. 2020).

Literature Cited

Andrews AH, Cordes EE, Mahoney MM, Munk K, Coale KH, Caillet GM, Heifetz J. 2002. Age, growth,and radiometric age validation of a deep-sea, habitat forming gorgonian (*Primnoa resedaeformis*) from the Gulf of Alaska. In: Watling L, Risk M (eds.). Biology of cold water corals. Hydrobiologia 471:101-10.

Baker KD, Wareham VE, Snelgrove PVR, Haedrich RL, Fifield DA, Edinger EN, Gilkinson KD. 2012. Distributional patterns of deep-sea coral assemblages in three submarine canyons off Newfoundland, Canada. Marine Ecology Progress Series 445:235-49. https://doi.org/10.3354/meps09448

Bryan TL, Metaxas A. 2006. Distribution of deep-water corals along the North American continental margins: relationships with environmental factors. Deep-Sea Research: I53:1865–79.

Bryan TL, Metaxas A. 2007. Predicting suitable habitat for deep-water gorgonian corals on the Atlantic and Pacific Continental Margins of North America. Marine Ecology Progress Series 330:113–26.

Buhl-Mortensen L, Olafsdottir SH, Buhl-Mortensen P, Burgos JM, Ragnarsson SA. 2015. Distribution ofnine cold-water coral species (Scleractinia and Gorgonacea) in the cold temperate North Atlantic: Effects of bathymetry and hydrography. Hydrobiologia 759(1):39–61. https://doi.org/10.1007/s10750-014-2116-x

Büscher JV, Form AU, Riebesell U. 2017. Interactive effects of ocean acidification and warming on growth, fitness and survival of the cold-water coral *Lophelia pertusa* under different food availabilities. Frontiers in Marine Science 4:101. https://doi.org/10.3389/fmars.2017.00101

Büscher JV, Wisshak M, Form AU, Titschack J, Nachtigall K, Riebesell U. 2019. In situ growth and bioerosion rates of *Lophelia pertusa* in a Norwegian fjord and open shelf cold-water coral habitat. PeerJ 7:e7586. https://doi.org/10.7717/peerj.7586

Carreiro-Silva M, Cerqueira T, Godinho A, Caetano M, Santos RS, Bettencourt R. 2014. Molecular mechanisms underlying the physiological responses of the cold-water coral *Desmophyllum dianthus to* ocean acidification. Coral Reefs 33(2):465–76. https://doi.org/10.1007/s00338-014-1129-2

Cerrano C, Cardini U, Bianchelli S, Corinaldesi C, Pusceddu A, Danovaro R. 2013. Red coral extinction risk enhanced by ocean acidification. Scientific Reports 3:1457. https://doi.org/10.1038/srep01457

Davies A J, Guinotte JM. 2011. Global habitat suitability for framework-forming cold-water corals. PLoS ONE 6(4):e18483. https://doi.org/10.1371/journ al.pone.0018483

Edinger EN, Sherwood OA, Piper DJW, Wareham VE, Baker KD, Gilkinson KD, Scott DB. 2011. Geologic features supporting deep-sea coral habitat in Atlantic Canada. Continental Shelf Research 31:S69-S84. https://doi.org/10.1016/j.csr.2010.07.004

Form AU, Riebesell U. 2012. Acclimation to ocean acidification during long-term CO_2 exposure in the cold-water coral *Lophelia pertusa*. Global Change Biology 18(3):843–53. https://doi.org/10.1111/j.1365-2486.2011.02583.x

Fountain CT, Waller RG, Auster PJ. 2019. Individual and population level variation in the reproductive potential of deep-sea corals from different regions within the Gulf of Maine. Frontiers in Marine Science 6:172. doi:10.3389/fmars.2019.00172

Frederiksen R, Jensen A, Westerberg H. 1992.The distribution of the scleractinian coral *Lophelia pertusa* around the Faroe Islands and the relation to internal tidal mixing. Sarsia 77:157–71.

Genin A, Dayton PK, Lonsdale PF, Spiess FN. 1986. Corals on seamount peaks provide evidence of current acceleration over deep-sea topography. Nature 322(6074):59–61. https://doi.org/10.1038/322059a0

Gomez CE, Wickes L, Deegan D, Etnoyer PJ, Cordes EE. 2019. Growth and feeding of deep-sea coral *Lophelia pertusa* from the California margin under simulated ocean acidification conditions. PeerJ 6:e5671. https://doi.org/10.7717/peerj.5671

Gori A, Ferrier-Pagès C, Hennige SJ, Murray F, Rottier C, Wicks LC, Roberts JM. 2016. Physiological response of the cold-water coral *Desmophyllum dianthus* to thermal stress and ocean acidification. PeerJ 4:e1606. https://doi.org/10.7717/peerj.1606

Guinotte JM, Davies AJ. 2014. Predicted deep-sea coral habitat suitability for the US West Coast. PLoS ONE 9(4):e93918. https://doi.org/10.1371/journal.pone.0093918

Guinotte, JM, Orr J, Cairns S, Freiwald A, Morgan L, and George R. 2006. Will human-induced changes in seawater chemistry alter the distribution of deep-sea scleractinian corals? Frontiers in Ecology and the Environment 4(3):141–6. https://doi.org/10.1890/1540-9295

Hebbeln D, Wienberg C, Dullo W-C, Freiwald A, Mienis F, Orejas C, Titschack J. 2020. Cold-water coral reefs thriving under hypoxia. Coral Reefs 39:853–9. https://doi.org/10.1007/s00338-020-01934-6

Hennige SJ, Wicks LC, Kamenos NA, Bakker DC, Findlay HS, Dumousseaud C, Roberts JM. 2014. Short-term metabolic and growth responses of the cold-water coral *Lophelia pertusa* to ocean acidification. Deep Sea Research Part II: Topical Studies in Oceanography 9:27–35. https://doi.org/10.1016/j.dsr2.2013.07.005

Hennige SJ, Wicks LC, Kamenos NA, Perna G, Findlay HS, Roberts JM. 2015. Hidden impacts of ocean acidification to live and dead coral framework. Proceedings of the Royal Society B: Biological Sciences 282(1813):20150990. https://doi.org/10.1098/rspb.2015.0990

Huff DD, Yoklavich MM, Love MS, Watters DW, Chai F, Lindley ST. 2013. Environmental factors that influence the distribution, size, and biotic relationships of the Christmas tree coral *Antipathes dendrochristos* in the Southern California Bight. Marine Ecology Progress Series 494:159–77. https://doi.org/10.3354/meps10591

Jackson EL, Davies AJ, Howell KL, Kershaw PJ, Hall-Spencer JM. 2014. Future-proofing marine protected area networks for cold water coral reefs. ICES Journal of Marine Science 71(9):2621–29. https://doi.org/10.1093/icesj ms/fsu099

Jones K, Devillers R, Edinger E. 2009. Relationships between cold-water corals off Newfoundland and Labrador and their environment. In: Sieber RE (ed.). Proceedings of the 2009 Spatial Knowledge and Information Canada Conference, February 19-22. Fernie BC, Canada. Vol. 1:15-20.

Kinlan BP, Poti M, Drohan AF, Packer DB, Dorfman DS, Nizinski MS. 2020. Predictive modeling of suitable habitat for deep-sea corals offshore the Northeast United States. Deep Sea Research Part I:Oceanographic Research Papers 158:103229. https://doi.org/10.1016/j.dsr.2020.103229.

Knudby A, Lirette C, Kenchington E, Murillo FJ. 2013. Species distribution models of black corals, large gorgonian corals and sea pens in the NAFO regulatory area. NAFO Scientific Council Research Document 13/078, Serial Number N6276.

Larsson AI, Lundälv T, van Oevelen D. 2013. Skeletal growth, respiration rate and fatty acid composition in the cold-water coral *Lophelia pertusa* under varying food conditions. Marine Ecology Progress Series 483:169–84. https://doi.org/10.3354/meps1 0284

Lazier A, Smith JE, Risk MJ, Schwarcz HP. 1999. The skeletal structure of *Desmophyllum cristagalli*: the use of deep-water corals in sclerochronology. Lethaia 32:119-30.

Lunden JJ, McNicholl CG, Sears CR, Morrison CL, Cordes EE. 2014. Acute survivorship of the deep-sea coral *Lophelia pertusa* from the Gulf of Mexico under acidification, warming, and deoxygenation. Frontiers in Marine Science 1(78):1-12. https://doi.org/10.3389/fmars.2014.00078

Mid-Atlantic Fisheries Management Council and National Marine Fisheries Service (MAFMC and NMFS). 2016. Amendment 16 to the Atlantic mackerel, squid, and butterfish Fishery Management Plan. Measures to protect deep sea corals from impacts of fishing gear. Environmental assessment, regulatory impact review, and initial regulatory flexibility analysis.https://www.mafmc.org/s/DeepSea-Corals-EA_Signed-FONSI.pdf.

Maier C, Popp P, Sollfrank N, Weinbauer MG, Wild C, Gattuso JP. 2016. Effects of elevated pCO_2 and feeding on net calcification and energy budget of the Mediterranean cold-water coral *Madrepora oculata*. Journal of Experimental Biology 219:3208–17. https://doi.org/10.1242/jeb.127159

Maier C, Schubert A, Sànchez MMB, Weinbauer MG, Watremez P, Gattuso JP. 2013. End of the century pCO_2 levels do not impact calcification in Mediterranean cold-water corals. PLoS ONE 8(4):e62655. https://doi.org/10.1371/journ al.pone.0062655

Maier C, Watremez P, Taviani M, Weinbauer MG, Gattuso JP. 2012. Calcification rates and the effect of ocean acidification on Mediterranean cold-water corals. Proceedings of the Royal Society B: Biological Sciences 279(1734):1716–23. https://doi.org/10.1098/rspb.2011.1763

Metaxas A, Lacharité M, Natasha De Mendonca S. 2019. Hydrodynamic connectivity of habitats of deep-water corals in Corsair Canyon, Northwest Atlantic: a case for cross-boundary conservation. Frontiers in Marine Science 6:159. doi:10.3389/fmars.2019.00159

Morato T, González-Irusta JM, Dominguez-Carrió C, Wei C-L, Davies A, Sweetman AK, Taranto GH,

Beazley L et al. 2020. Climate-induced changes in the suitable habitat of cold-water corals and commercially important deep-sea fishes in the North Atlantic. Global Change Biology 26:2181-202. https://doi.org/10.1111/gcb.14996

Mortensen PB, Buhl-Mortensen L. 2004. Distribution of deep-water gorgonian corals in relation to benthic habitat features in the Northeast Channel (Atlantic Canada). Marine Biology 144:1223–38.

Mortensen PB, Buhl-Mortensen L. 2005. Morphology and growth of the deep-water gorgonians *Primnoa resedaeformis* and *Paragorgia arborea*. Marine Biology 147:775–88.

Mortensen PB, Buhl-Mortensen L, Gordon DC. 2006. Distribution of deep-water corals in Atlantic Canada. In: Proceedings of the 10th International Coral Reef Symposium:1832-48.

Mortensen PB, Hovland T, Fosså JH, Furevik DM. 2001. Distribution, abundance and size of *Lophelia pertusa* coral reefs in mid-Norway in relation to seabed characteristics. Journal of the Marine Biological Association UK 81:581–97.

Movilla J, Gori A, Calvo E, Orejas C, López-Sanz À, Domínguez-Carrió C, Grinyó J, Pelejero C. 2014. Resistance of two Mediterranean cold-water coral species to low-pH conditions. Water 6:59-67. https://doi.org/10.3390/w6010059

National Oceanic and Atmospheric Administration (NOAA). 2010. NOAA Strategic Plan for deep-sea coral and sponge ecosystems: research, management, and international cooperation. NOAA Coral Reef Conservation Program, NOAA Technical Memorandum CRCP 11:67 pp.

National Oceanic and Atmospheric Administration (NOAA) Physical Sciences Laboratory. 2020.[Internet]. ROMS-NWA Dynamical Downscaling. (Accessed 3 Nov. 2020). https://www.psl.noaa.gov/ipcc/roms/

Naumann MS, Orejas C, Wild C, Ferrier-Pagès C. 2011. First evidence for zooplankton feeding sustaining key physiological processes in a scleractinian cold-water coral. Journal of Experimental Biology 214(21):3570–76. https://doi.org/10.1242/jeb.061390

Orr JC, Fabry VJ, Aumont O, Bopp L, Doney SC, Feely RA, Gnanadesikan A, Gruber N, Ishida A, Joos F, Key RM, Lindsay K, Maier-Reimer E, Matear R, Monfray P, Mouchet A, Najjar RG, Plattner G-K, Rodgers KB, Sabine CL, Sarmiento JL, Schlitzer R, Slater RD, Totterdell IJ, Weirig M-F, Yamanaka Y, Yool A. 2005. Anthropogenic ocean acidification over the twenty-first century and its impact on calcifying organisms. Nature 437(7059):681-6. https://www.nature.com/articles/nature04095

Packer DB, Boelke D, Guida V, McGee L-A. 2007. State of deep coral ecosystems in the northeastern US region: Maine to Cape Hatteras. In: Lumsden SE, Hourigan TF, Bruckner AW, Dorr G (eds). The state of deep coral ecosystems of the United States. NOAA Technical Memorandum CRCP-3. 195-232.

Packer DB, Nizinski MS, Bachman MS, Drohan AF, Poti M, Kinlan BP. 2017a. State of the deep-sea coral and sponge ecosystems off the Northeast United States. In: Hourigan T, Etnoyer PJ, Cairns SD (eds). The state of deep-sea coral and sponge ecosystems of the United States. NOAA Technical Memorandum NMFS-OHC-4. Silver Spring, MD. 237-97. https://deepseacoraldata.noaa.gov/library/resolveuid/95df948cc33444b7bea34ae78db8b600

Packer D, Nizinski MS, Cairns SD, Hourigan TF. 2017b. [Internet]. Deep-sea coral taxa in the U.S. Northeast Region: depth and geographical distribution. https://deepseacoraldata.noaa.gov/library/2017-state-of-deep-sea-corals-report.

Quattrini AM, Nizinski MS, Chaytor JD, Demopoulos AWJ, Roark EB, France SC, Moore, JA, Heyl T, Auster PJ, Kinlan B, Ruppel C, Elliott KP, Kennedy BRC, Lobecker E, Skarke A, Shank TM. 2015. Exploration of the canyon-incised continental margin of the Northeastern United States reveals dynamic habitats and

diverse communities. PLoS ONE 10(10):e0139904. DOI:10.1371/journal.pone.0139904

Rengstorf AM, Yesson C, Brown C, Grehan AJ. 2013. High resolution habitat suitability modelling can improve conservation of vulnerable marine ecosystems in the deep sea. Journal of Biogeography 40(9):1702–14. https://doi.org/10.1111/jbi.12123

Risk MJ, Heikoop JM, Snow MG, Beukens. 2002. Lifespans and growth patterns of two deep-sea corals: *Primnoa resedaeformis* and *Desmophyllum cristagalli*. In: Watling L, Risk M (eds.). Biology of cold water corals. Hydrobiologia 471:125-31.

Roberts JM, Murray F, Anagnostou E, Hennige S, Gori A, Henry LA, Fox A, Kamenos N, Foster GL. 2016. Cold-water corals in an era of rapid global change: Are these the deep ocean's most vulnerable ecosystems? In: Goffredo S, Dubinsky Z (eds) The Cnidaria, past, present and future (pp. 593–606). Springer: Cham. p. 593-606. https://doi.org/10.1007/978-3-319-31305-4_36

Smith Jr. KL, Kaufmann RS. 1999. Long-term discrepancy between food supply and demand in the deep eastern North Pacific. Science 284:1174–77.

Sweetman AK, Thurber AR, Smith CR, Levin LA, Mora C, Wei C-L, Gooday AJ, Jones DOB, Rex M, Yasuhara M, Ingels J, Ruhl HA, Frieder CA, Danovaro R, Würzburg L, Baco A, Grupe BM, Pasulka A, Meyer KS, Dunlop KM, Henry L-A, Roberts JM. 2017. Major impacts of climate change on deep-sea benthic ecosystems. Elementa-Science of the Anthropocene 5:1–23. http://doi.org/10.1525/elementa.203

Thiem Ø, Ravagnan E, Fossa JH, Berntsen J. 2006. Food supply mechanisms for cold-water corals along a continental shelf edge. Journal of Marine Systems 60:207-19. https://doi.org/10.1016/j.jmarsys.2005.12.004.

Thoma JN, Pante E, Brugler MR, France SC. 2009. Deep-sea octocorals and antipatharians show no evidence of seamount-scale endemism in the NW Atlantic. Marine Ecology Progress Series 397:25-35.

Thresher RE, Tilbrook B, Fallon S, Wilson NC, Adkins J. 2011. Effects of chronic low carbonate saturation levels on the distribution, growth and skeletal chemistry of deep-sea corals and other seamount megabenthos. Marine Ecology Progress Series 442:87–99. https://doi.org/10.3354/meps09400

Tittensor DP, Baco AR, Hall-Spencer JM, Orr, JC, Rogers, AD. 2010. Seamounts as refugia from ocean acidification for cold-water stony corals. Marine Ecology 31:212–25.

Watling L, Auster P. 2005. Distribution of deep-water Alcyonacea off the northeast coast of the United States. *In*: Freiwald A, Roberts A (eds). Cold-water corals and ecosystems. Berlin, Heidelberg: Springer-Verlag. 259-64.

White M, Mohn C, de Stigter H, Mottram G. 2005. Deep-water coral development as a function of hydrodynamics and surface productivity around the submarine banks of the Rockall Trough, NE Atlantic. *In*: Freiwald A, Roberts A (eds). Cold-water corals and ecosystems. Berlin, Heidelberg:Springer-Verlag. 503–14.

Yesson C, Taylor ML, Tittensor DP, Davies AJ, Guinotte J, Baco A, Black J, Hall-Spencer JM, Rogers AD. 2012. Global habitat suitability of cold-water octocorals. Journal of Biogeography 39:1278–92. https://doi.org/10.1111/j.1365-2699.2011.02681.x.

Zheng MD, Cao L. 2014. Simulation of global ocean acidification and chemical habitats of shallow- and cold-water coral reefs. Advances in Climate Change Research 5(4):189–96. https://doi.org/10.1016/j.accre.2015.05.002.

Marine Rocky Bottom >200m

System: Marine

Subsystem: Subtidal >200m

Class: Rocky Bottom

Sub-class: Bedrock, Rubble, Cobble, Gravel

Geographic Area: Entire Area

Overall Vulnerability Rank = Moderate

Habitat Sensitivity = Moderate

Climate Exposure = Moderate

	**Marine Rocky Bottom >200m**	Attribute Mean	Data Quality	Distribution of Expert Scores
Sensitivity Attributes	Habitat condition	1.6	2	
	Habitat fragmentation	1.7	1.8	
	Distribution/Range	2.5	2	
	Mobility/Ability to spread or disperse	3.2	2	
	Resistance	2.6	2.4	
	Resilience	2.5	2.4	
	Sensitivity to changes in abiotic factors	2	1.8	
	Sensitivity and intensity of non−climate stressors	1.6	1.8	
	Dependency on critical ecological linkages	1.4	1.4	
	Sensitivity Component Score	Moderate		
Exposure Factors	Sea surface temp	n/a	n/a	
	Bottom temp	1.8	2.8	
	Air temp	n/a	n/a	
	River temp	n/a	n/a	
	Surface salinity	n/a	n/a	
	Bottom salinity	2.8	2.6	
	pH	4	2.4	
	Sea level rise	n/a	n/a	
	Precipitation	n/a	n/a	
	Floods	n/a	n/a	
	Droughts	n/a	n/a	
	Exposure Component Score	Moderate		
**Overall Vulnerability Rank**		**Moderate**		

- Low
- Moderate
- High
- Very High

Habitat Name: Marine Rocky Bottom >200 m

System**:** Marine

Subsystem: Subtidal >200 m

Class: Rocky Bottom

Sub-class: Bedrock, Rubble, Cobble, Gravel

Geographic Area: Entire Area

Habitat Description: This sub-class includes rocky marine offshore habitats at depths >200 m, including substrates composed of bedrock, boulders, cobble, and gravel (granules and pebbles). These deep, rocky habitats are found in the Gulf of Maine and on the outer continental shelf and slope and on the four seamounts within the U.S. EEZ. Rocky habitats are especially common on the walls of submarine canyons that cut into the shelf and slope all along the outer continental margin and at the bases of the walls where debris has fallen to the canyon floor (Ryan et al. 1978; Valentine et al. 1980; Obelcz et al. 2014). More isolated areas of gravel and boulders are also found between the canyons and on the continental slope and rise (Brothers et al. 2013; Quattrini et al. 2015). Semi-consolidated mudstones also occur in the canyon walls and are included in this habitat type (Cooper et al. 1987). In addition, this habitat subclass includes the epibenthic flora and fauna associated with hard bottoms, but excludes specific habitats identified elsewhere (i.e., non-calcareous algal bed, rooted vascular, coral-dominated hard bottom, mollusk reef). Calcareous algae and artificial reefs and wrecks are also included in this sub-class, although these habitat types are relatively rare in waters >200 m. Deep rocky bottom habitatsare more common in the canyons and along the shelf in New England that were exposed to glacial impacts than in the Mid-Atlantic (MAFMC 2016; NEFMC 2020).

Overall Climate Vulnerability Rank: Moderate (82% certainty from bootstrap analysis). Bootstrap analysis found a 18% probability that the overall vulnerability rank is low.

Climate Exposure: Moderate. The overall moderate climate exposure score was influenced by two relatively High climate exposure means: Bottom Salinity and pH (2.8 and 4.0, respectively). The exposure attribute mean for Bottom Temperature was Low-Moderate (1.8). pH is projected to drastically decrease from historic levels for the entire study area by the end-of-century (increasing ocean acidification). The scores for Bottom Salinity were placed in all four scoring bins, suggesting variability in the range of projections in the climate models. Note: climate change is also expected to reduce dissolved oxygen concentrations and the flux of organic matter to the bottom even in deep water (Levin and Le Bris 2020).

Habitat Sensitivity: Moderate. Four of the nine sensitivity attribute means were ≥2.5, with Mobility/Ability to Spread or Disperse being the highest (3.2). Rocky bottom habitats are unable to move or spread substantial distances, although the flora and fauna associated with them are generally capable of moving through reproductive dispersal or migration. The Distribution/Range, Resistance, and Resilience attribute means were 2.5 or 2.6, but were spread across all of the scoring bins. This likely reflects the variability of sensitivity in the abiotic (rocky bottom) and biotic (flora and fauna) associated with this habitat subclass. The other attribute means were between 1.6 and 2.0.

Data Quality & Gaps: The data quality scores for the three climate exposure factors were Moderate to High (i.e., 2.8, 2.6, and 2.4 for Bottom Temperature, Bottom Salinity, and pH, respectively). The relative Low data quality score for pH is likely reflecting some uncertainty in how the CMIP5 projected changes in surface pH will affect the pH and calcium carbonate ($CaCO_3$) saturation levels in deeper water. Possible data gaps for this sub-class may include the relatively low resolution and accuracy of mapped rocky bottoms, as well as the range of substrate types included in the sub-class.

For habitat sensitivity, data quality for five of the nine attributes were deemed moderate (≤2.0), while the other attributes were scored low (1.4 and 1.8).

Positive or Negative Climate Effect for the Northeast U.S.: The effect of climate change on deep offshore rocky bottom habitats in the Northeast U.S. is expected to be mostly negative (85% of the experts' scores were negative and 15% neutral).

Climate Effects on Habitat Condition and Distribution: The distribution of deep, offshore rocky substrates is not expected to change as a result of climate change. Smaller-grained gravel substrates are susceptible to physical disturbance caused by bottom currents, which can be strong in the canyons (Valentine et al. 1984; Obelcz et al. 2014), but rocky substrates are less likely to be mobilized than sand or mud. Infrequent, large-scale events such as landslides and slumps from the canyon walls or on the adjoining continental slope and turbidity currents, however, do re-distribute even large boulders to new locations (Knebel 1984; Brothers et al. 2013; Obelcz et al. 2014).

Increasing air temperature and CO_2 absorption affect surface waters more directly than deep water, but water column effects are also expected to occur in deep outer shelf and slope habitats that are likely to affect benthic animals inhabiting rocky substrates. Temperature increases of 3ºC and salinity increases of 0.7-0.8 ppt are projected in the upper ocean (0-300 m) in the Northwest Atlantic by the year 2100 with a doubling of CO_2 (Saba et al. 2016).

Future temperature and salinity changes at the bottom on the outer shelf and slope are dependent on the interaction between warm, saltier Gulf Stream water and fresher, colder Labrador slope water and the effects of meanders in the Gulf Stream and warm core rings that cause periodic exchanges of shelf and slope water (Gawarkiewicz et al. 2012; Wanninkhof et al. 2015; Zhang and Gawarkiewicz 2015). A similar situation exists in the Gulf of Maine where Labrador Slope Water mixes with warmer slope water entering the Gulf through the Northeast Channel; inter-annual variations in the contributions from these two sources affect bottom water temperatures in the deep basins (Townsend et al. 2015).

Some deep-water fauna that inhabit rocky substrates are susceptible to decreased pH and related effects on calcium carbonate chemistry. Sweetman et al. (2017) concluded that the most significant changes in pH are expected in the 200 to 3000 meter depth range in all oceans, with increased acidity (lower pH) of 0.29-0.37 units. Lower pH reduces the ability of animals to produce calcareous skeletal structures and could have additional ecosystem-level effects (Orr et al. 2005; Fabray et al. 2008). Reductions in pH of 0.2 units by 2100 are projected for more than 17% of the seafloor deeper than 500 m in the North Atlantic for three out of four climate scenarios (Gehlen et al. 2014). For the high emission scenario (RCP8.5) pH decreases exceeding 0.2 units are projected for 22.5% of North Atlantic canyons and 7.7% of seamounts by the end of the century. Aragonite saturation levels in deep water along the Atlantic coast are much lower than in shallower water and climate change will cause a shoaling of the saturation horizon into more intermediate depths (Wanninkhof et al. 2015; Levin and Le Bris 2020).

Two additional effects of climate change that are expected to affect deep water benthic habitats that were not considered in the exposure scoring of this assessment are reduced dissolved oxygen concentrations and less organic matter reaching the seafloor. Because oxygen is less soluble in warmer water, the warming of deep bottom waters would reduce the supply of dissolved oxygen available to benthic animals (Levin and Le Bris 2020). The combined effects of increased vertical stratification and reduced oxygen solubility in warmer water is expected to lead to widespread deoxygenation of oceanic waters, especially between 200 and 700 m (Levin and Le Bris 2020). Also, as surface waters warm, the water column becomes more stratified which reduces nutrient input from below the photic zone, primary productiondecreases and less particulate organic matter is delivered to the benthic animals that rely on it for food (Sweetman et al. 2017; Levin and Le Bris 2020).

The effects of climate change on the temperature, salinity, pH, and oxygen of deep bottom water are likely to vary by depth and location along the outer continental shelf and between the outer shelf and the Gulf of Maine. This is primarily due to latitudinal variations in the velocity and direction of the Gulf Stream along the outer shelf north of Cape Hatteras and the interaction between warm slope water and colder Labrador slope water entering the Gulf of Maine (Frantantoni and Pickart 2007; Townsend et al. 2015; Saba et al. 2016). Predicted changes in water temperature and salinity in the upper 300 m of the northwest Atlantic are heavily dependent on how much the northerly extent and velocity of the Gulf Stream will change as it passes Cape Hatteras (Saba et al. 2016). Long-term changes caused by global warming may not affect deep water habitats on the outer shelf and slope as much as the episodic, short term effects of meanders in the Gulf Stream and the frequency and magnitude of warm core rings.

Habitat Summary: Biological communities found in deep rocky habitats are mostly composed of attached, immobile species like corals and sponges that are susceptible to changes in water column properties. Bottom conditions at depths >200 m do not vary seasonally and are less directly affected by atmospheric conditions (e.g., increased air temperatures and absorption of oxygen and CO_2) than near-surface waters. However, bottom conditions in the canyons and outer shelf and slope and in deep water habitats of the Gulf of Maine are currently subjected to changes in temperature and salinity caused by periodic variations in southerly flowing cold bottom water (Labrador slope water) and northerly flowing warm, more saline Gulf Stream water, and by warm core rings that transfer warmer slope water on to the shelf and colder shelf water seaward (Gawarkiewicz et al. 2012; Wanninkhof et al. 2015; Zhang and Gawarkiewicz 2015). Organisms that attach to rocky substrates on the outer shelf and upper slope are, therefore, more adapted (less sensitive) to changes in temperature and salinity than animals in deeper water where temperature and salinity are less variable.

At present, ocean water in depths >200 m north of Cape Hatteras is under-saturated, or only slightly super-saturated, with aragonite, an essential carbonate mineral needed to produce $CaCO_3$ (Wanninkhof et al. 2015), thus making deep water benthic communities highly sensitive to decreases in pH and calcification capacity. Because rocky habitat biota are suspension feeders, they are sensitive to decreases in the amount of particulate organic matter (POC) that reaches the bottom (see deep offshore reef narrative). As surface waters warm and the water column becomes more stratified, primary production will be reduced and less organic matter will be delivered to the benthos (Levin and Le Bris 2020). Their feeding success also depends on the action of bottom currents that transport POC that reaches the bottom although it is unlikely that bottom current velocities or direction in deep water would be affected by climate change.

Mobile bottom-tending commercial fishing gear can affect gravel bottom habitats (Stevenson et al. 2004). Other impacts can include offshore marine mining and wind energy (Johnson et al. 2008), although these activities are less common in waters >200 m.

Literature Cited

Brothers DS, ten Brink US, Andrews BD, Chaytor JD. 2013. Geomorphic characterization of the U.S. Atlantic continental margin. Marine Geology 338:46-63.

Cooper RA, Valentine P, Uzmann JR, Slater RA. 1987. Submarine canyons. p. 52-65. *In:* Backus RH, Bourne DW (eds). Georges Bank. MIT Press, Cambridge, MA.

Fabry VJ, Seibel BA, Feely RA, Orr JC. 2008. Impacts of ocean acidification on marine fauna and ecosystem processes. ICES Journal of Marine Science 65(3):414-32.

Frantantoni PS, Pickart RS. 2007. The western North Atlantic shelf break current system in summer. Journal of Physical Oceanography 37:2509-33.

Gawarkiewicz G, Todd RE, Pluoddemann A.J, Andres M, Manning JP. 2012. Direct interaction between the Gulf Stream and the shelbreak south of New England. Scientific Reports 2:553. https://doi.org/10.1038/srep00553

Gehlen M, Séférian R, Jones DOB, Roy T, Roth R, Barry J, Bopp L, Doney SC, Dunne JP, Heinze C, Joos F, Orr JC, Resplandy L, Segschneider J, Tjiputra J. 2014. Projected pH reductions by 2100 might put deep North Atlantic biodiversity at risk. Biogeosciences 11:6955-67. https://doi.org/10.5194/bg-11-6955-2014

Knebel HJ 1984. Sedimentary processes on the Atlantic continental slope of the United States. Marine Geology 61(1):43-74.

Johnson MR, Boelke C, Chiarella LA, Colosi PD, Greene K, Lellis-Dibble K, Ludemann H, Ludwig M, McDermott S, Ortiz J, Rusanowsky D, Scott M, Smith J. 2008. Impacts to marine fisheries habitat from nonfishing activities in the northeastern United States. NOAA Technical Memorandum. National Oceanic and Atmospheric Administration, National Marine Fisheries Service.NMFS-NE-2O9. 1-328.

Levin LA, Le Bris N. 2020. The deep ocean under climate change. Science 350(6262):766-8.

Mid-Atlantic Fishery Management Council (MAFMC). 2016. Amendment 16 to the Atlantic Mackerel, Squid, and Butterfish Fishery Management Plan - Measures to Protect Deep Sea Corals from Impacts of Fishing Gear, Mid-Atlantic Fishery Management Council in cooperation with the National MarineFisheries Service**.** 420 pp.

New England Fishery Management Council (NEFMC) 2020. Omnibus Deep-Sea Coral Amendment, Including a Final Environmental Assessment. Amendment 9 to the Atlantic Herring FMP, Amendment 20 to the Atlantic Scallop FMP, Amendment 8 to the Monkfish FMP, Amendment 24 to the Northeast Multispecies FMP, Amendment 6 to the Red Crab FMP, and Amendment 7 to the Skate Complex FMP. New England Fisheries Management Council, Newburyport, MA. 566 pp.

Obelcz J, Brothers D, Chaytor J, ten Brink Uri, Ross S, Brooke S. 2014. Geomorphic characterization of four shelf-sourced submarine canyons along the U.S. Mid-Atlantic continental margin. Deep Sea Research Part II: Topical Studies in Oceanography. 104:106-19. 10.1016/j.dsr2.2013.09.013

Orr JC, Fabry VJ, Aumont O, Bopp L, Doney SC, Feely RA, Gnanadesikan A, Gruber N, Ishida A, Joos F, Key RM, Lindsay K, Maier-Reimer E, Matear R, Monfray P, Mouchet A, Najjar RG, Plattner G-K, Rodgers KB, Sabine CL, Sarmiento JL, Schlitzer R, Slater RD, Totterdell IJ, Weirig M-F, YamanakaY, Yool A. 2005. Anthropogenic ocean acidification over the twenty-first century and its impact on calcifying organisms. Nature 437(7059):681-6.

Quattrini AM, Nizinski MS, Chaytor JD, Demopoulos AWJ, Roark EB, France SC, Moore JA, Heyl T, Auster PJ, Kinlan B, Ruppel C, Elliott KP, Kennedy BRC, Lobecker E, Skarke A, Shank TM. 2015.Exploration of the canyon-incised continental margin of the northeastern United States reveals dynamic habitats and diverse communities. PLoS ONE 10(10):e0139904. https://doi.org/10.1371/journal.pone.0139904.

Ryan WBF, Cita MB, Miller EL, Hanselman D, Nesteroff WD, Hecker B, Nibbelink M. 1978. Bedrock geology in New England submarine canyons. Oceanologica Acta 1(2):233-54.

Saba VS, Griffies SM, Anderson WG, Winton M, Alexander MA, Delworth TL, Hare JA, Harrison MJ,

Rosati A, Vecchi GA, Zhang R. 2016. Enhanced warming of the Northwest Atlantic Ocean under climate change. Journal of Geophysical Research: Oceans 121(1):118-32.

Stevenson D, Chiarella L, Stephan D, Reid R, Wilhelm K, McCarthy J, Pentony M. 2004. Characterization of the fishing practices and marine benthic ecosystems of the Northeast U.S. Shelf, and an evaluation of the potential effects of fishing on Essential Fish Habitat. NOAA Technical Memorandum NMFS-NE-181. 179 p.

Sweetman AK, Thurber AR, Smith CR, Levin LA, Mora C, Wei C-L, Gooday AJ, Jones DOB, Rex M, Yasuhara M, Ingels J, Ruhl HA, Frieder CA, Danovaro R, Würzberg L, Baco A, Grupe BM, Pasulka A, Meyer KS, Dunlop KM, Henry L-A, Roberts JM. 2017. Major impacts of climate change on deep-sea benthic ecosystems. Elementa Science of the Anthropocene 5:4. http://doi.org/10.1525/elementa.203

Townsend DW, Pettigrew NR, Thomas MA, Neary MG, McGillicuddy DJ Jr, O'Donnell J. 2015. Watermasses and nutrient sources to the Gulf of Maine. Journal of Marine Research 73(3-4):93-122. doi:10.1357/002224015815848811

Valentine PC, Uzmann JR, Cooper RA. 1980. Geology and biology of Oceanographer submarine canyon. Marine Geology 38:283-312.

Valentine PC, Uzmann JR , Cooper RA. 1984. Submarine sand dunes and sedimentary processes in Oceanographer canyon. Journal of Sedimentary Petrology 54(3):0704-15.

Wanninkhof R, Barbero L, Byrne R, Cai W-J, Huang W-J, Zhang J-Z, Baringer M, Langdon C. 2015. Ocean acidification along the Gulf coast and the East coast of the USA. Continental Shelf Research 98:54-71.

Zhang WG, Gawarkiewicz GG. 2015. Dynamics of the direct intrusion of Gulf Stream ring water onto the Mid-Atlantic Bight shelf. Geophysical Research Letters 42:7687-95. doi:10.1002/2015GL065530.

Marine Mud Bottom >200m

System: Marine

Subsystem: Subtidal >200m

Class: Unconsolidated Bottom

Sub-class: Mud

Geographic Area: Entire Area

Overall Vulnerability Rank = Low

Habitat Sensitivity = Low

Climate Exposure = Moderate

	**Marine Mud Bottom >200m**	Attribute Mean	Data Quality	Distribution of Expert Scores
Sensitivity Attributes	Habitat condition	1.6	2	
	Habitat fragmentation	1.3	2.2	
	Distribution/Range	1.3	2.2	
	Mobility/Ability to spread or disperse	1.5	1.8	
	Resistance	2.2	2.2	
	Resilience	1.8	2.2	
	Sensitivity to changes in abiotic factors	1.8	2	
	Sensitivity and intensity of non−climate stressors	1.8	2.2	
	Dependency on critical ecological linkages	1.5	1.6	
	Sensitivity Component Score	Low		
Exposure Factors	Sea surface temp	n/a	n/a	
	Bottom temp	2.1	2.8	
	Air temp	n/a	n/a	
	River temp	n/a	n/a	
	Surface salinity	n/a	n/a	
	Bottom salinity	2.6	2.8	
	pH	4	2.4	
	Sea level rise	n/a	n/a	
	Precipitation	n/a	n/a	
	Floods	n/a	n/a	
	Droughts	n/a	n/a	
	Exposure Component Score	Moderate		
**Overall Vulnerability Rank**		**Low**		

Low
Moderate
High
Very High

Habitat Name: Marine Mud Bottom >200 m

System: Marine

Subsystem: Subtidal >200 m

Class: Unconsolidated Bottom

Sub-class: Mud

Geographic Area: Entire Area

Habitat Description: This offshore marine subtidal sub-class includes muddy (clay and silt) bottom habitats on the outer continental shelf and slope and in the Gulf of Maine, where the water is deeper than 200 meters, as well as associated infauna and epifauna such as mollusks, marine worms, small crustaceans, gastropods, and polychaetes. The sedimentary environment on the slope east and northeast of Georges Bank was heavily influenced by glaciation whereas the southern New England-Mid Atlantic slope is typical of an unglaciated, non-carbonate environment (Pratt 1968). Soft muddy sediments predominate on the continental slope and rise at depths beyond the shelf break at depths greater than 300-400 m, in submarine canyons, and in deep basins in the Gulf of Maine (Knebel 1984; Stanley and Wear 1978; Watling 1998; Valentine et al. 1980; Pierdomenico et al. 2015). Submarine canyons are distributed along the entire length of the outer shelf and slope in the Northeast region between Cape Hatteras and Georges Bank. Bottom habitat conditions (e.g., temperature and salinity) on the upper slope are not subject to seasonal variations, but are subject to variable interactions between colder Labrador slope waterand warmer, more saline slope water in the Gulf Stream (Wanninkhof et al. 2015; Saba et al. 2016). In deeper water on the lower continental slope and rise (>1200 m) conditions are less variable than in shallower water on the upper slope and outer shelf (Levin and Gooday 2003).

In a comparison of benthic macrofaunal species diversity from Boston harbor to Lydonia Canyon, Maciolek and Smith (2009) found the highest diversity at mid-slope depths (1220-1350 m). Hecker (1990) reported higher megafaunal densities on the upper (200-500 m) and lower slope (>1600 m) than at mid-slope depths. Kelly et al. (2010) reviewed existing taxonomic records for six deep-water zones in theNew England region and found that diversity decreased with increasing distance from shore and bottom topography. The continental slope (300-2000 m, primarily silt and clay sediments) was the zone with the greatest number of known species. (See Offshore Bottom Slope Water >200 m narrative for additional information on outer shelf and slope hydrography affecting deep-water benthic habitats).

Overall Vulnerability Rank: Low (100% certainty from bootstrap analysis).

Climate Exposure: Moderate. This habitat scored High (4.0) for climate exposure to pH changes, Moderate to High (2.8) for Bottom Salinity, and Moderate (2.1) for Bottom Temperature. The scores for Bottom Salinity were placed in all four scoring bins, suggesting variability in the range of projections in the climate models. pH is projected to drastically decrease from historic levels for the entire study area bythe end-of-century. Note: climate change is also expected to reduce dissolved oxygen concentrations and the flux of organic matter to the bottom even in deep water (Levin and Le Bris 2020).

Habitat Sensitivity: Low. Resistance, Resilience, Habitat Condition, and Sensitivity to Changes in Abiotic Factors, and Sensitivity and Intensity of Non-Climate Stressors were scored as Moderate (1.6-2.2). Sensitivities to all other factors were Low to Moderate (1.3-1.5). In general, environmental conditions in deep water are more stable than in shallower water on the shelf. However, because there is less variation in bottom temperatures and salinity, infaunal and epifaunal organisms that live in or on muddy sediments (clay and silt) in deep water may be generally less well adapted, and, therefore, more sensitive to environmental changes, should they occur, than benthic organisms in shallower, more dynamic, marine environments. This potential sensitivity to change was likely reflected in the Moderate scores for Resistance and Resilience, Sensitivity to Changes in Abiotic Factors, and Sensitivity and Intensity of Non-Climate Factors.

Data Quality & Gaps: For exposure to climate change, data quality was scored relatively High (2.8) for Bottom Temperature and Bottom Salinity, and somewhat lower (2.4) for pH. The relative low data quality score for pH is likely reflecting some uncertainty in how the CMIP5 projected changes in surface pH will affect the pH and calcium carbonate ($CaCO_3$) saturation levels in deeper water.

Four of the nine sensitivity attribute data quality scores were ≤2.0. Mobility/Ability to Spread or Disperse and Dependency on Critical Ecological Linkages scored 1.6 and 1.8, respectively, and Habitat Condition and Sensitivity to Changes in Abiotic Factors scored 2.0. The other five attributes were also not scored Very High (2.2), indicating that, overall, there was limited certainty in the sensitivity scoring for this habitat.

Positive or Negative Climate Effect in the Northeast U.S. Shelf: The effect of climate change on offshore mud bottom habitats in the region is expected to be neutral or negative, with experts' scores nearly equally divided between the two (55% neutral, 45% negative).

Climate Effects on Habitat Condition and Distribution: The distribution of deep-water mud habitats on the outer continental slope and in the deep basins of the Gulf of Maine is expected to be minimally affected by climate change. Much of the present sediment distribution in deep offshore water was determined thousands of years ago during periods of lower sea level when glacial outwash eroded the continental margin, and by the slow accumulation of soft sediment from surface waters that has been occurring since the glaciers retreated. As depth increases on the outer slope, bottom habitats are dominated by silt and clay sediments and their associated fauna. Shallower outer shelf substrates tend to be sandier, with rocky substrates largely confined to submarine canyons and seamounts (see narratives for offshore sand and rocky habitats).

Increasing air temperature and CO_2 absorption affect surface waters more directly than deep water, but water column effects are also expected to occur in deep continental slope habitats that may affect infauna and epifauna associated with muddy sediments. Temperature increases of 3°C, and salinity increases of 0.7-0.8 ppt, are predicted in depths of 0-300 m along the outer continental shelf in the northwest Atlantic by the year 2100 (Saba 2016). Future temperature and salinity changes at the bottom on the outer shelf and slope are dependant on the interaction between warm, saltier Gulf Stream water and fresher, colder Labrador slope water, as well as the effects of meanders from the Gulf Stream and warm core rings that cause periodic exchanges of shelf and slope water (Gawarkiewicz et al. 2012; Wanninkhof et al. 2015; Zhang and Gawarkiewicz 2015). A similar situation exists in the Gulf of Maine where Labrador Slope Water mixes with warmer slope water entering the Gulf through the Northeast Channel; inter-annual variations in the contributions from these two sources affect bottom water temperatures in the deep basins which vary between 6 and 10°C (Townsend et al. 2015). Bottom temperatures on the continental slope are variable, ranging from 4 to 17°C with the higher temperatures in shallower water (Worthington 1976).

Sweetman et al. (2017) concluded that the most significant changes in pH are expected in the 200-3000 m depth range in all oceans, with increased acidity (lower pH) of 0.29-0.37 units. Lower pH reduces the ability of infauna and epifauna associated with mud habitat (e.g., mollusks, small crustaceans, gastropods) to produce calcareous skeletal structures and could have additional ecosystem-level effects (Orr et al. 2005; Fabry et al. 2008). Reductions in pH of - 0.2 units by 2100 are projected for more than 17% of the seafloor deeper than 500 m in the North Atlantic for three out of four climate scenarios (Gehlen et al. 2014). Aragonite saturation levels in deep water along the Atlantic coast are much lower than in shallowerwater and climate change will cause a shoaling of the saturation horizon into more intermediate depths (Wanninkhof et al. 2015; Levin and Le Bris 2020). Current atmospheric CO_2 concentration is 412 ppm; stabilization at 450 ppm would produce calcite and aragonite under-saturation in most of the deep oceans (Caldeira and Wickett 2005). The concentration of atmospheric CO_2 is expected to go much higher than 450 ppm in the next few decades. As more CO_2 is absorbed into the upper water column, the shoaling of the aragonite saturation horizon is expected to reduce the ability of benthic organisms along the continental slope and rise to incorporate $CaCO_3$ into shells and other body parts (Orr et al. 2005; Fabry et al. 2008). Similar effects could be expected in

deepwater basins in the Gulf of Maine.

Two additional effects of climate change that are expected to affect deep water benthic habitats that werenot considered in the climate exposure scoring of this assessment are reduced dissolved oxygen concentrations and less organic matter reaching the seafloor. Because oxygen is less soluble in warmer water, the warming of deep bottom waters would reduce the supply of dissolved oxygen available to benthic animals (Levin and Le Bris 2020). The combined effects of increased vertical stratification and reduced oxygen solubility in warmer water is expected to lead to widespread deoxygenation of oceanic waters, especially between 200 and 700 meters (Levin and Le Bris 2020). Also, as surface waters warm, the water column becomes more stratified which reduces nutrient input from below the photic zone, primary production decreases and less particulate organic matter is delivered to the benthic animals that rely on it for food (Sweetman et al. 2017; Levin and Le Bris 2020).

The projected effects of climate change on the current temperature, salinity, pH, and oxygen regimes of deep bottom water are likely to vary by depth and location along the outer continental shelf due to latitudinal variations in the velocity and direction of the Gulf Stream along the outer shelf north of Cape Hatteras and the interaction between warm slope water and colder Labrador slope water entering the Gulf of Maine (Frantantoni and Pickart 2007; Townsend et al. 2015; Saba et al. 2016). Predicted changes in water temperature and salinity in the upper 300 m of the northwest Atlantic are heavily dependent on how much the northerly extent and velocity of the Gulf Stream will change as it passes Cape Hatteras (Saba et al. 2016). Long-term changes caused by global warming may not affect deep-water habitats on the outer shelf and slope as much as the episodic, short-term effects of meanders in the Gulf Stream and the frequency and magnitude of warm core rings.

Habitat Summary: Bottom conditions at depths >200 m do not vary seasonally and are less directly affected by atmospheric conditions (e.g., increased air temperatures and absorption of oxygen and CO_2) than near-surface waters. However, bottom conditions on the outer shelf and slope and in deep water habitats of the Gulf of Maine are currently subjected to changes in temperature and salinity caused by periodic variations in southerly flowing cold Labrador slope water, and northerly flowing warm, more saline Gulf Stream water, as well as from warm core rings that transfer warmer slope water on to the shelfand colder shelf water seaward (Gawarkiewicz et al. 2012; Wanninkhof et al. 2015; Zhang and Gawarkiewicz 2015). Because the warming effects of the Gulf Stream are restricted to depths above 200 m in most of the Mid-Atlantic Bight north of Cape Hatteras (Wanninkhof et al. 2015), it is likely that benthic communities in deeper, more stable cold water environments would be more sensitive to episodic or long term climate-induced changes in temperature and salinity (should they occur) than communities inshallower water that are exposed to more variable temperature and salinity conditions (Levin and Gooday2003). Also, because aragonite saturation levels are naturally low in deep water, small increases in acidification will have a disproportionate effect on shell-forming, deep water infaunal and epifaunal organisms that rely on aragonite for calcification (see references above). At present, ocean water in depths >200m north of Cape Hatteras is under-saturated, or only slightly super-saturated, with aragonite, anessential carbonate mineral needed to produce $CaCO_3$ (Wanninkhof et al. 2015).

Mobile bottom-tending commercial fishing gear can affect mud bottom habitats more so than sand bottom (Stevenson et al. 2004). Other impacts can include offshore marine mining and wind energy (Johnson et al. 2008), although these are not anticipated in waters >200 m.

Literature Cited

Caldeira K, Wickett ME. 2005. Ocean model predictions of chemistry changes from carbon dioxide emissions to the atmosphere and ocean. Journal of Geophysical Research 110:C09S04. doi:10.1029/2004JC002671.

Fabry VJ, Seibel BA, Feely RA, Orr JC. 2008. Impacts of ocean acidification on marine fauna and ecosystem processes. ICES Journal of Marine Science 65(3):414-32.

Frantantoni PS, Pickart RS. 2007. The western North Atlantic shelf break current system in summer. Journal of Physical Oceanography 37:2509-33.

Gawarkiewicz G, Todd RE, Pluoddemann AJ, Andres M, Manning JP. 2012. Direct interaction between the Gulf Stream and the shelbreak south of New England. Scientific Reports 2:553. https://doi.org/10.1038/srep00553.

Gehlen M, Séférian R, Jones DOB, Roy T, Roth R, Barry J, Bopp L, Doney SC, Dunne JP, Heinze C, Joos F, Orr JC, Resplandy L, Segschneider J, Tjiputra J. 2014. Projected pH reductions by 2100 might put deep North Atlantic biodiversity at risk. Biogeosciences 11:6955–67. https://doi.org/10.5194/bg-11-6955-2014.

Hecker B. 1990. Variation in megafaunal assemblages on the continental margin south of New England. Deep-Sea Research 37:37–57.

Johnson MR, Boelke C, Chiarella LA, Colosi PD, Greene K, Lellis-Dibble K, Ludemann H, Ludwig M, McDermott S, Ortiz J, Rusanowsky D, Scott M, Smith J. 2008. Impacts to marine fisheries habitat from nonfishing activities in the northeastern United States. NOAA Technical Memorandum. National Oceanic and Atmospheric Administration, National Marine Fisheries Service.NMFS-NE-2O9. 1-328.

Kelly NE, Shea EK, Metaxas A, Haedrich RL, Auster P. 2010. Biodiversity of the deep-sea continental margin bordering the Gulf of Maine (NW Atlantic): relationships among sub-regions and to shelf systems. PLoS ONE 5(11): e13832. doi:10.1371/journal.pone.0013832

Knebel HJ 1984. Sedimentary processes on the Atlantic continental slope of the United States. Marine Geology 61(1):43-74.

Levin LA, Gooday AJ. 2003. The Deep Atlantic Ocean. Chapter 5 *In*: Ecosystems of The World. 111-78.

Levin LA, Le Bris N. 2020. The deep ocean under climate change. Science 350(6262):766-8.

Maciolek NJ and Smith WK. 2009. Benthic species diversity along a depth gradient: Boston Harbor to Lydonia Canyon. Deep Sea Research II (56):1763-74.

Orr JC, Fabry VJ, Aumont O, Bopp L, Doney SC, Feely RA, Gnanadesikan A, Gruber N, Ishida A, Joos F, Key RM, Lindsay K, Maier-Reimer E, Matear R, Monfray P, Mouchet A, Najjar RG, Plattner G-K, Rodgers KB, Sabine CL, Sarmiento JL, Schlitzer R, Slater RD, Totterdell IJ, Weirig M-F, YamanakaY, Yool A. 2005. Anthropogenic ocean acidification over the twenty-first century and its impact on calcifying organisms. Nature 437(7059):681-6.

Pierdomenico M, Guida VG, Macelloni L, Chiocci FL, Rona PA, Scranton MI, Asper V, Diercks A. 2015. Sedimentary facies, geomorphic features and habitat distribution at the Hudson Canyon head from AUV multibeam data. Deep Sea Research Part II: Topical Studies in Oceanography 121:112-25.

Pratt RM. 1968. Atlantic Continental Shelf and Slope of the United States – physiography and sediments of the deep-sea basin. U.S. Department of the Interior Geological Survey Professional Paper 529-B:45.

Saba VS, Griffies SM, Anderson WG, Winton M, Alexander MA, Delworth TL, Hare JA, Harrison MJ, Rosati A, Vecchi GA, Zhang R. 2016. Enhanced warming of the Northwest Atlantic Ocean under climate change. Journal of Geophysical Research: Oceans 121(1):118-32.

Stanley DJ, Wear CM. 1978. The “mud line” – an erosion-depositional boundary on the upper continental slope. Marine Geology 28(1-2):M19-29.

Stevenson D, Chiarella L, Stephan D, Reid R, Wilhelm K, McCarthy J, Pentony M. 2004. Characterization of the fishing practices and marine benthic ecosystems of the northeast US shelf, and an evaluation of the potential effects of fishing on essential habitat. NOAA Technical Memorandum NMFS NE 181. 179 pp.

Sweetman AK, Thurber AR, Smith CR, Levin LA, Mora C, Wei C-L, Gooday AJ, Jones DOB, Rex M, Yasuhara M, Ingels J, Ruhl HA, Frieder CA, Danovaro R, Würzberg L, Baco A, Grupe BM, Pasulka A, Meyer KS, Dunlop KM, Henry L-A, Roberts JM. 2017. Major impacts of climate change on deep-sea benthic ecosystems. Elementa Science of the Anthropocene 5:4. http://doi.org/10.1525/elementa.203

Townsend DW, Pettigrew NR, Thomas MA, Neary MG, McGillicuddy DJ Jr, O'Donnell J. 2015. Water masses and nutrient sources to the Gulf of Maine. Journal of Marine Research 73(3-4):93-122. doi:10.1357/002224015815848811

Valentine PC, Uzmann JR, Cooper RA. 1980. Geology and biology of Oceanographer submarine canyon. Marine Geology 38:283-312.

Valentine PC, Uzmann JR , Cooper RA. 1984. Submarine sand dunes and sedimentary processes in Oceanographer canyon. Journal of Sedimentary Petrology 54(3):0704-15.

Wanninkhof R, Barbero L, Byrne R, Cai W-J, Huang W-J, Zhang J-Z, Baringer M, Langdon C. 2015. Ocean acidification along the Gulf coast and the East coast of the USA. Continental Shelf Research 98:54-71.

Watling L. 1998. Benthic fauna of soft substrates in the Gulf of Maine. *In*: Dorsey EM; Pederson J, eds. Effects of fishing gear on the sea floor of New England. MIT Sea Grant Publication 98-4:20-9.

Worthington LV. 1976. On the North Atlantic Circulation. Johns Hopkins Oceanographic Studies No. 6. Baltimore, MD, Johns Hopkins Univ. Press**:** 110 pp.

Zhang WG, Gawarkiewicz GG. 2015. Dynamics of the direct intrusion of Gulf Stream ring water onto the Mid-Atlantic Bight shelf. Geophysical Research Letters 42:7687–95. doi:10.1002/2015GL065530.

Marine Sand Bottom >200m

System: Marine

Subsystem: Subtidal >200m

Class: Unconsolidated Bottom

Sub-class: Sand

Geographic Area: Entire Area

Overall Vulnerability Rank = Low

Habitat Sensitivity = Low

Climate Exposure = Moderate

	**Marine Sand Bottom >200m**	Attribute Mean	Data Quality	Distribution of Expert Scores
Sensitivity Attributes	Habitat condition	1.4	2.4	
	Habitat fragmentation	1.4	2.2	
	Distribution/Range	1.7	2.2	
	Mobility/Ability to spread or disperse	1.4	2.4	
	Resistance	2.1	2.2	
	Resilience	1.9	2.4	
	Sensitivity to changes in abiotic factors	1.9	2	
	Sensitivity and intensity of non−climate stressors	1.2	2.2	
	Dependency on critical ecological linkages	1.5	1.8	
	Sensitivity Component Score	Low		
Exposure Factors	Sea surface temp	n/a	n/a	
	Bottom temp	2.1	2.8	
	Air temp	n/a	n/a	
	River temp	n/a	n/a	
	Surface salinity	n/a	n/a	
	Bottom salinity	2.7	2.8	
	pH	4	2.4	
	Sea level rise	n/a	n/a	
	Precipitation	n/a	n/a	
	Floods	n/a	n/a	
	Droughts	n/a	n/a	
	Exposure Component Score	Moderate		
**Overall Vulnerability Rank**		**Low**		

Low
Moderate
High
Very High

Habitat Name: Marine Sand Bottom >200 m

System: Marine

Subsystem: Subtidal >200 m

Class: Unconsolidated Bottom

Sub-class: Sand

Geographic Area: Entire Area

Habitat Description: This sub-class includes subtidal marine sandy habitats on the outer continental shelf and slope where the water is deeper than 200 m, as well as associated infauna and epifauna such as mollusks, marine worms, small crustaceans, gastropods, and polychaetes. This subclass excludes specific habitats identified elsewhere (i.e., non-calcareous algal bed, rooted vascular beds, and reef-forming mollusks, such as blue mussels and eastern oysters).

Sediments in the deep-water basins in the Gulf of Maine are primarily mud. However, deep areas around outcrop rock (e.g., Schoodic Ridge and Lindenkohl Knoll) have a greater percentage of sand than central parts of the basins. Sandy sediments generally are restricted to shallower depths on the outer shelf and slope that are more disturbed by bottom currents and in the heads, upper walls, and floors of submarine canyons where sand from the shelf is transported into and down the canyons, sometimes during episodic turbidity currents (Valentine et al. 1980; Knebel 1984; Levin and Gooday 2003; Obelcz et al. 2014; Pierdomenico et al. 2015). Sediments in deeper, less disturbed habitats are dominated by clay and silt (Pratt 1968; Knebel 1984). Submarine canyons are scattered along the entire length of the outer shelf and slope in the Northeast region between Cape Hatteras and Georges Bank. Habitat conditions at depths >200 m on the outer shelf are generally less variable than in shallower water (Mountain 2003), although there are periodic exchanges of deep, warmer, more saline slope water and colder bottom shelf water caused by warm core rings and meanders in the Gulf Stream (Churchill et al. 2003; Gawarkiewicz et al. 2012; Zhang and Gawarkiewicz 2015; Saba et al. 2016).

Climate Vulnerability Rank: Low (100% certainty from bootstrap analysis).

Climate Exposure: Moderate. This habitat scored High (4.0) for exposure to pH changes, Moderate to High (2.8) for Bottom Salinity, and Moderate (2.1) for Bottom Temperature. The scores for Bottom Salinity were placed in all four scoring bins, suggesting variability in the range of projections in the climate models. pH is projected to drastically decrease from historic levels for the entire study area by the end-of-century (increasing ocean acidification). Note: climate change is also expected to reduce dissolved oxygen concentrations and the flux of organic matter to the bottom even in deep water (Levin and Le Bris2020).

Habitat Sensitivity: Low. Resistance, Resilience, and Sensitivity to Changes in Abiotic Stressors were the three highest scored sensitivity attributes at 1.9-2.1. Sensitivities to other factors were lower (1.2-1.7). In general, environmental conditions in deep water are more stable than in shallower water on the shelf. However, because there is less variation in bottom temperatures and salinity, infaunal and epifaunal organisms that live in or on sandy sediments in deep water may be generally less well adapted, and, therefore, more sensitive to environmental changes, should they occur, than benthic organisms in shallower, more dynamic, marine environments. This potential sensitivity to change was reflected in the moderate scores for Resistance and Resilience, and Sensitivity to Changes in Abiotic Factors.

Data Quality & Gaps: For exposure to climate change, data quality was relatively High (2.8) for Bottom Temperature and Bottom Salinity. Data quality was scored somewhat lower (2.4) for pH, reflecting some uncertainty in how the CMIP5 projected changes in surface pH will affect the pH and calcium carbonate ($CaCO_3$) saturation levels in deeper water.

Two of the nine sensitivity attribute data quality scores (i.e., Dependency on Critical Ecological Linkages and

Sensitivity to Changes in Abiotic Factors) were less than or equal to 2.0. All other attributes scored 2.2 or higher, with Habitat Condition, Mobility/Ability to Spread or Disperse, and Resilience scoring 2.4, indicating that there was slightly more confidence in the sensitivity evaluations for this habitat than, for example, offshore mud habitats that occur in deeper water.

<u>Positive or Negative Climate Effect in the Northeast U.S. Shelf</u>: The effect of climate change on offshore sand habitats in the region is expected to be neutral to negative, with experts' scores equally divided between the two (50% neutral, 50% negative).

<u>Climate Effects on Habitat Condition and Distribution</u>: The distribution of sandy deep-water habitats on the outer continental shelf and upper slope is expected to be minimally affected by climate change. Much of the present sediment distribution in deep offshore water was determined thousands of years ago during periods of lower sea level when glacial outwash eroded the continental margin and transported large quantities of sand, gravel, and boulders to what is now the outer shelf and slope. In more recent geological times, lower sea levels have allowed additional sand to be transported from the shelf into the canyons and on to the upper slope, and in some cases by high-density turbidity currents that flow through the canyons (Knebel 1984; Obelcz et al. 2014). Sand that reaches the canyon floors is re-worked by bottom currents and, in some cases, forms dunes (Valentine et al. 1984). Shallower outer shelf substrates tend to be sandier, with rocky substrates largely confined to submarine canyons and seamounts and silt and clay in deeper, more depositional environments (Pratt 1968; Stanley and Wear 1978). (See narratives for offshore mud and rocky habitats).

Increasing air temperature and atmospheric CO_2 concentrations affect surface waters more directly than deep water, but the condition of deep-water habitats in the region could be affected by changes in bottom water properties. Benthic organisms in deep water will potentially be affected to varying degrees by climate-induced changes in bottom temperature, salinity, oxygen, and pH, and by the increased vertical stratification of warmer surface waters (Levin and Le Bris 2020). Temperature increases of 3ºC, and salinity increases of 0.7-0.8 ppt, are predicted in depths of 0-300 m along the outer continental shelf in thenorthwest Atlantic by the year 2100 (Saba et al. 2016).

Some deep-water fauna that inhabit sandy substrates are susceptible to decreased pH and related effects on calcium carbonate chemistry. Sweetman et al. (2017) concluded that the most significant changes in pH are expected in the 200 to 3000 meter depth range in all oceans, with increased acidity (lower pH) of 0.29-0.37 units. Lower pH reduces the ability of infauna and epifauna associated with sand habitat (e.g., mollusks, small crustaceans, gastropods) to produce calcareous skeletal structures and could have additional ecosystem-level effects (Orr et al. 2005; Fabry et al. 2008). Aragonite saturation levels in deepwater along the Atlantic coast are much lower than in shallower water and climate change will cause a shoaling of the saturation horizon into more intermediate depths (Wanninkhof et al. 2015; Levin and Le Bris 2020). At present, ocean water in depths >200m north of Cape Hatteras is under-saturated, or only slightly super-saturated, with aragonite, an essential carbonate mineral needed to produce $CaCO_3$ (Wanninkhof et al. 2015).

Future temperature and salinity changes at the bottom on the outer shelf and slope are dependant on the interaction between warm, saltier Gulf Stream water and fresher, colder Labrador slope water, as well as the effects of meanders from the Gulf Stream and warm core rings that cause periodic exchanges of shelf and slope water (Gawarkiewicz et al. 2012; Wanninkhof et al. 2015; Zhang and Gawarkiewicz 2015). A similar situation exists in the Gulf of Maine where Labrador Slope Water mixes with warmer slope water entering the Gulf through the Northeast Channel; inter-annual variations in the contributions from these two sources affect bottom water temperatures in the deep basins, which vary between 6 and 10°C (Townsend et al. 2015). Bottom temperatures on the continental slope are variable, ranging from 4 to 17°C with the higher temperatures in shallower water (Worthington 1976).

Two additional effects of climate change that are expected to affect deep water benthic habitats that were not considered in the climate exposure scoring of this assessment are reduced dissolved oxygen concentrations and less organic matter reaching the seafloor. Because oxygen is less soluble in warmer water, the warming of deep bottom waters would reduce the supply of dissolved oxygen available to benthic animals (Levin and Le Bris 2020). The combined effects of increased vertical stratification and reduced oxygen solubility in warmer water is expected to lead to widespread deoxygenation of oceanic waters, especially between 200 and 700 meters (Levin and Le Bris 2020). Also, as surface waters warm, the water column becomes more stratified which reduces nutrient input from below the photic zone, primary production decreases and less particulate organic matter is delivered to the benthic animals that rely on it for food (Sweetman et al. 2017; Levin and Le Bris 2020). As more CO_2 is absorbed into the upper water column, the shoaling of the aragonite saturation horizon is expected to reduce the ability of benthic organisms along the outer continental shelf and slope to incorporate aragonite and calcite into shells and other body parts (Orr et al. 2005; Fabry et al. 2008). Reductions in pH of 0.2 units by 2100 are projected for more than 17% of the seafloor deeper than 500 m in the North Atlantic for three out of fourclimate scenarios (Gehlen et al. 2014). For the high emission scenario (RCP8.5), pH decreases exceeding 0.2 units are projected for 22.5% of North Atlantic canyons and 7.7% of seamounts by the end of the century. Aragonite saturation levels in deep water along the Atlantic coast are much lower than in shallower water and climate change will cause a shoaling of the saturation horizon into more intermediate depths (Wanninkhof et al. 2015; Levin and Le Bris 2020).

The projected effects of climate change on the current temperature, salinity, pH, and oxygen regimes of deep bottom water are likely to vary by depth and location along the outer continental shelf due to latitudinal variations in the velocity and direction of the Gulf Stream along the outer shelf north of Cape Hatteras and the interaction between warm slope water in the Gulf Stream flowing north and colder Labrador slope water flowing south (Saba et al. 2016). Predicted changes in water temperature and salinity in the upper 300 m of the northwest Atlantic are heavily dependent on how much the northerly extent and velocity of the Gulf Stream will change as it passes Cape Hatteras (Saba et al. 2016). Long-term changes caused by global warming may not affect deep water habitats on the outer shelf and slope as much as the episodic, short term effects of meanders in the Gulf Stream and the frequency and magnitude of warm core rings.

Habitat Summary: Bottom conditions in deep water are less directly affected by atmospheric conditions (e.g., increased air temperatures and absorption of oxygen and CO_2) than near-surface waters. However, bottom conditions on the outer continental shelf are currently subject to changes in temperature and salinity caused by periodic variations in southerly flowing cold bottom water from the Labrador Slope Water and northerly flowing warm, more saline Gulf Stream water, as well as from warm core rings that transfer warmer slope water on to the shelf and colder shelf water seaward (Wanninkhof et al. 2015; Gawarkiewicz et al. 2012; Zhang and Gawarkiewicz 2015). For this reason, it is likely that benthic communities in sandy substrates on the outer shelf would be less sensitive to episodic or long-term changes in temperature and salinity than communities in deeper water muddy habitats on the continental slope where bottom temperatures and salinity are more constant (see deep offshore mud bottom narrative).

Benthic animals on the outer shelf are generally not exposed to regular changes in dissolved oxygen and pH which could become more problematic under climate change. Increased acidity is expected to be the most severe issue since deep water along the outer shelf already is more acidic than shallower water (Feely et al. 2008; Feely et al. 2009) and, therefore, has a lower capacity for $CaCO_3$ formation which could become worse if pH declines further (see above). Deposit and suspension-feeding organisms on the outer shelf are also sensitive to reductions in the flux of particulate organic matter to the bottom that are expected to occur as surface waters warm and vertical stratification of the water column becomes more pronounced.

Sand bottom habitats are less impacted by mobile bottom-tending commercial fishing gear than mud or gravel bottom habitats (Stevenson et al. 2004). Other impacts can include offshore marine mining and wind energy (Johnson et al. 2008), although these are not anticipated in waters >200 m.

Literature Cited

Churchill JH, Manning JP, Beardsley RC. 2003. Slope water intrusions on to Georges Bank. Journal of Geophysical Research 108 (C11):8012.

Fabry VJ, Seibel BA, Feely RA, Orr JC. 2008. Impacts of ocean acidification on marine fauna and ecosystem processes. ICES Journal of Marine Science 65(3):414-32.

Feely RA, Sabine CL, Hernandez-Ayon JM, Ianson D,Hales Burke. 2008. Evidence for upwelling of corrosive "acidified" water onto the continental shelf. Science 320(5882):1490-92.

Feely RA, Doney SC ,Cooley SC. 2009. Present conditions and future changes in a high-CO_2 world. Oceanography 22(4):36-47.

Gawarkiewicz G, Todd RE, Pluoddemann A.J, Andres M, Manning JP. 2012. Direct interaction between the Gulf Stream and the shelbreak south of New England. Scientific Reports 2:553. https://doi.org/10.1038/srep00553

Gehlen M, Séférian R, Jones DOB, Roy T, Roth R, Barry J, Bopp L, Doney SC, Dunne JP, Heinze C, Joos F, Orr JC, Resplandy L, Segschneider J, Tjiputra J. 2014. Projected pH reductions by 2100 might put deep North Atlantic biodiversity at risk. Biogeosciences 11:6955-67. https://doi.org/10.5194/bg-11-6955-2014

Johnson MR, Boelke C, Chiarella LA, Colosi PD, Greene K, Lellis-Dibble K, Ludemann H, Ludwig M, McDermott S, Ortiz J, Rusanowsky D, Scott M, Smith J. 2008. Impacts to marine fisheries habitat from nonfishing activities in the northeastern United States. NOAA Technical Memorandum.National Oceanic and Atmospheric Administration, National Marine Fisheries Service. NMFS-NE-2O9. 1-328.

Knebel HJ 1984. Sedimentary processes on the Atlantic continental slope of the United States. Marine Geology 61(1):43-74.

Levin LA, Gooday A.J. 2003. The Deep Atlantic Ocean. Chapter 5 *In*: Ecosystems of The World. 111-78.

Levin LA, Le Bris N. 2020. The deep ocean under climate change. Science 350(6262):766-8.

Mountain DW. 2003. Variability in the properties of Shelf Water in the Middle Atlantic Bight, 1977-1999. Journal of Geophysical Research 108(C1):3014.

Obelcz J, Brothers D, Chaytor J, ten Brink Uri, Ross S, Brooke S. 2014. Geomorphic characterization of four shelf-sourced submarine canyons along the U.S. Mid-Atlantic continental margin. Deep Sea Research Part II: Topical Studies in Oceanography 104:106–19. 10.1016/j.dsr2.2013.09.013

Orr JC, Fabry VJ, Aumont O, Bopp L, Doney SC, Feely RA, Gnanadesikan A, Gruber N, Ishida A, Joos F, Key RM, Lindsay K, Maier-Reimer E, Matear R, Monfray P, Mouchet A, Najjar RG, Plattner G-K, Rodgers KB, Sabine CL, Sarmiento JL, Schlitzer R, Slater RD, Totterdell IJ, Weirig M-F, Yamanaka Y, Yool A. 2005. Anthropogenic ocean acidification over the twenty-first century and its impact on calcifying organisms. Nature 437(7059):681-6.

Pierdomenico M, Guida VG, Macelloni L, Chiocci FL, Rona PA, Scranton MI, Asper V, Diercks A. 2015.

Sedimentary facies, geomorphic features and habitat distribution at the Hudson Canyon head from AUV multibeam data. Deep Sea Research Part II: Topical Studies in Oceanography 121:112-25.

Pratt, R.M. 1968. Atlantic Continental Shelf and Slope of the United States – Physiography and Sediments of the Deep-Sea Basin. U.S. Dept. Interior Geological Survey Professional Paper 529-B. 45 pp.

Saba VS, Griffies SM, Anderson WG, Winton M, Alexander MA, Delworth TL, Hare JA, Harrison MJ, Rosati A, Vecchi GA, Zhang R. 2016. Enhanced warming of the Northwest Atlantic Ocean under climate change. Journal of Geophysical Research: Oceans 121(1):118-32.

Stanley DJ, Wear CM. 1978. The “mud line” – an erosion-depositional boundary on the upper continental slope. Marine Geology 28(1-2):M19-M29.

Stevenson D, Chiarella L, Stephan D, Reid R, Wilhelm K, McCarthy J, Pentony M. 2004. Characterization of the fishing practices and marine benthic ecosystems of the Northeast U.S. Shelf, and an evaluation of the potential effects of fishing on Essential Fish Habitat. NOAA Technical Memorandum NMFS-NE-181. 179 p.

Sweetman AK, Thurber AR, Smith CR, Levin LA, Mora C, Wei C-L, Gooday AJ, Jones DOB, Rex M, Yasuhara M, Ingels J, Ruhl HA, Frieder CA, Danovaro R, Würzberg L, Baco A, Grupe BM, Pasulka A, Meyer KS, Dunlop KM, Henry L-A, Roberts JM. 2017. Major impacts of climate change on deep-sea benthic ecosystems. Elementa Science of the Anthropocene 5:4. http://doi.org/10.1525/elementa.203.

Valentine PC, Uzmann JR, Cooper RA. 1980. Geology and biology of Oceanographer submarine canyon. Marine Geology 38:283-312.

Valentine PC, Uzmann JR , Cooper RA. 1984. Submarine sand dunes and sedimentary processes in Oceanographer canyon. Journal of Sedimentary Petrology 54(3):0704-15.

Wanninkhof R, Barbero L, Byrne R, Cai W-J, Huang W-J, Zhang J-Z, Baringer M, Langdon C. 2015. Ocean acidification along the Gulf coast and the East coast of the USA. Continental Shelf Research 98:54-71.

Worthington LV. 1976. On the North Atlantic Circulation. Johns Hopkins Oceanographic Studies No. 6. Baltimore, MD, Johns Hopkins Univ. Press**:** 110 pp.

Zhang WG, Gawarkiewicz GG. 2015. Dynamics of the direct intrusion of Gulf Stream ring water onto the Mid-Atlantic Bight shelf. Geophysical Research Letters 42:7687–95. doi:10.1002/2015GL065530.

Marine Shellfish Aquaculture

System: Marine

Subsystem: Subtidal & Intertidal

Class: Reef

Sub-class: Mollusk Aquaculture

Geographic Area: Entire Area

Overall Vulnerability Rank = Moderate

Habitat Sensitivity = Moderate

Climate Exposure = High

	Marine Shellfish Aquaculture	Attribute Mean	Data Quality	Distribution of Expert Scores
Sensitivity Attributes	Habitat condition	1.7	1.3	
	Habitat fragmentation	1.4	2	
	Distribution/Range	1.5	2	
	Mobility/Ability to spread or disperse	1.2	2	
	Resistance	1.8	1.6	
	Resilience	1.8	1.4	
	Sensitivity to changes in abiotic factors	3.1	2.1	
	Sensitivity and intensity of non−climate stressors	2.8	2.1	
	Dependency on critical ecological linkages	2.5	1.4	
	Sensitivity Component Score	Moderate		
Exposure Factors	Sea surface temp	3.7	2.8	
	Bottom temp	n/a	n/a	
	Air temp	n/a	n/a	
	River temp	n/a	n/a	
	Surface salinity	1.6	2.6	
	Bottom salinity	n/a	n/a	
	pH	4	2.2	
	Sea level rise	2.9	2.2	
	Precipitation	2.4	2.1	
	Floods	n/a	n/a	
	Droughts	n/a	n/a	
	Exposure Component Score	High		
	**Overall Vulnerability Rank**	**Moderate**		

Low
Moderate
High
Very High

Habitat Name: Marine Shellfish Aquaculture

System**:** Marine

Subsystem: Subtidal and Intertidal

Class: Reef

Sub-class: Mollusk Aquaculture

Geographic Area: Entire Area

Habitat Description: This sub-class includes cultured shellfish used for aquaculture in marine waters >30 ppt. Aquaculture of molluscan shellfish is conducted within intertidal and subtidal nearshore marine environments along the U.S. eastern seaboard. Shellfish farms vary widely in spatial scale, type of aquaculture practiced and target species. Eastern oysters (*Crassostrea virginica*) may be cultivated on leased seafloor using traditional, on-bottom culture or using gear-based methods such as floating bags, or on-bottom cages. Blue mussels (*Mytilus edulis*) are reared on long-lines placed vertically between the sea surface and sediments and more recently, are grown offshore from floating reefs. Northern hard clams (*Mercenaria mercenaria*) are cultivated in seafloor sediments. Small-scale sea scallop (*Placopecten magellanicus*) culture is underway in the Gulf of Maine where scallops are raised in multi-tiered, lantern nets suspended in the water column, or by "ear-hanging" where a hole is drilled in the corner of the shell and the scallop is hung from a submerged line for grow-out. Molluscan aquaculture is practiced in the same waters where natural populations of shellfish occur.

Overall Climate Vulnerability Rank: Moderate (92% certainty from bootstrap analysis). Although the majority of the bootstrap results match the results of the categorical vulnerability rank, 6% of the bootstrap results were in the High vulnerability rank and 2% in the Low vulnerability rank.

Climate Exposure: High. Two factors contributed to the High exposure score: pH (4.0), and Sea Surface Temperature (3.7). Sea Level Rise (SLR) also scored moderately High (2.9), and Precipitation scored in the Moderate range (2.4). Surface Salinity was scored relatively low (1.6). Based on projected declines in pH, ocean acidification is expected to increase significantly, particularly in the Mid-Atlantic. Increases in temperature are projected to be very high throughout the study area, and greatest in the Gulf of Maine. SLR is expected to impact the entire study area, although the greatest relative rise is in the Mid-Atlantic region. The frequency and intensity of extreme rain events are projected in the study area. Because this sub-class includes both intertidal and shallow, subtidal zones, habitats are located at the nexus of significant atmospheric and oceanic change.

Habitat Sensitivity: Moderate. Three factors contributed to the Moderate sensitivity score: Sensitivity to Changes in Abiotic Factors (3.1), Sensitivity and Intensity of Non-Climate Stressors (2.8) and Dependency on Critical Ecological Linkages (2.5). Moderate habitat sensitivity, as compared to intertidal and subtidal shellfish mollusk habitats that were scored High, reflects the assumption that cultured shellfish are actively placed into areas of suitable habitat condition and that aquaculture gear is mobile. Careful site selection can mitigate effects of abiotic and non-climate factors that affect shellfish (e.g., dredging, harmful algal blooms, shoreline hardening, invasive species, marine construction, and pollution).

Data Quality & Gaps: For the climate exposure factors scored as High, data quality scores were in the Moderate range (2.1-2.8), which likely reflects the limited spatial data available for shellfish aquaculture habitat. The lack of robust spatial data specific to shellfish aquaculture habitats required that expert judgement also be applied for the overlap in climate exposure and shellfish aquaculture. In addition, data quality is lower for nearshore coastal habitats due to the low resolution of CMIP5 and ROMS-NWA projections for climate exposure in nearshore, shallow coastal areas.

All of the nine sensitivity attribute data quality scores were ≤2.1. Although the climate sensitivity for marine intertidal and subtidal mollusk reefs are believed to be moderate, there remains some uncertainty in species' capacity to adapt and respond to climate change (e.g., synergistic effects of climate and non-climate stressors).

Generally, the range of climate and non-climate effects, particularly synergistic effects, on shellfish

aquaculture is limited, and further research is needed to understand how cultured shellfish on farms or contained in aquaculture gear may be affected by climate factors.

Positive or Negative Climate Effect in the Northeast U.S. Shelf: The effect of climate change on marine shellfish aquaculture is expected to be neutral (65% of the experts' scores were neutral, 25% were negative, and 10% positive). Predictions of a neutral effect of climate on marine shellfish aquaculture may reflect the high tolerance of mollusks to changing environmental conditions. This score also takes into account the potential for growers to shift operations away from suboptimal environments, although economic and siting limitations (e.g., availability of bottom leases) may limit the capacity of growers to avoid areas subjected to climate effects. For this reason, cultured shellfish may experience impacts from declining pH, warming seawater temperatures, rising sea level, and increasing extreme precipitation, as reflected in a 25% negative score.

Climate Effects on Habitat Condition and Distribution: Nearshore marine shellfish aquaculture habitats are projected to experience high exposure to ocean acidification and reduced pH, increased seawater temperatures, and moderate exposure to sea level rise and increased extreme precipitation in the New England and Mid-Atlantic regions.

Many laboratory studies have examined effects of ocean acidification on bivalve mollusk species (e.g. oysters, blue mussels, hard clams) that are commercially cultivated. These studies have documented negative effects of ocean acidification on calcification, growth, and survival of shellfish (e.g., Gazeau et al. 2013; Ekstrom et al. 2015; Clements and Chopin 2016). Responses are species-specific, with larvae and juveniles more vulnerable to ocean acidification than adults (e.g., Kurihara 2008; Kroeker et al. 2013; Gledhill et al. 2015). Lower pH levels can make it difficult for calcifying mollusks to produce shells (Clements and Chopin 2016). Shellfish in marine environments are less susceptible to ocean acidification than their counterparts in estuarine environments, since marine systems have fewer sources of acid input and are more buffered than estuaries (Waldbusser et al. 2011). However, nearshore coastal waters are generally more susceptible to acidification than oceanic waters because they are subject to more acid sources and are generally less buffered than oceanic waters (i.e., differences in the amount of dissolved inorganic carbon, dissolved and particulate organic carbon, total alkalinity and nutrients from riverine and estuarine sources) (Ekstrom et al. 2015; Gledhill et al. 2015).

Increasing seawater temperatures may affect the condition and distribution of shellfish aquaculture species in nearshore oceanic waters. For example, southern populations of blue mussels appear to be shifting northward in response to warming temperatures (Jones et al. 2010). Thermal stress, in conjunction with ocean acidification conditions, was found to cause metabolic depression in blue mussels from the Gulf of Maine (Lesser 2016), suggesting that elevated temperatures may result in suboptimal growing conditions for this cold-adapted species. Warming seawater temperatures can increase susceptibility of shellfish to disease, parasites and predation by local and invasive species (Burge et al. 2014; Smolowitz 2013). High temperature and low salinity are known drivers for both MSX and Dermo in the eastern oyster (Burreson et al. 2000; Ford and Smolowitz 2007; Burge et al. 2014). Between 1990 and 1992, a dramatic range extension of Dermo disease was reported over a 500 km area in the northeastern United States from Delaware Bay, New Jersey to Cape Cod Bay, Massachusetts (Ford and Smolowitz 2007). By 1995, Dermo was reported in oysters as far north as Maine (Burge et al. 2014).

Increased sea level rise may permanently inundate existing intertidal habitat that support shellfish aquaculture, while extreme changes in salinity from coastal storms and increased precipitation could alter habitat quality for shellfish and affect the types of aquaculture conducted there.

Cultured mollusks are generally resilient and tolerate variability in environmental conditions. Growers closely manage shellfish beds and aquaculture gear to maintain condition and avoid loss or damage. For this reason, the effects of climate change on the condition and distribution of shellfish aquaculture habitat are expected to be largely neutral. Molluscan aquaculture is subject to many of the same influences as natural shellfish habitats and careful site selection can be used to mitigate effects of ocean acidification and other climate-

related variables on shellfish (Clements and Chopin 2016; Snyder et al. 2017).

Habitat Summary: Marine shellfish aquaculture occurs in intertidal and subtidal waters, often on seafloor that is leased to individual growers. Filter-feeding bivalves consume naturally occurring phytoplankton and particulates from the water column and provide an important link between benthic seafloor communities and primary productivity in the water column (Shumway et al. 2003). In addition, reef-building shellfish and associated aquaculture gear create hard structures that provide habitat for a variety of sessile colonizers and mobile fish and invertebrates with mollusks often serving as keystone species in these habitats.

Cultivated mollusks are exposed to a variety of non-climate and abiotic stressors in marine environments that may act synergistically with climate effects. Marine shellfish aquaculture sites may experience disturbance from storms, which are expected to increase in frequency and intensity in the Northeast region (USGCRP 2017). Episodic storms can increase physical or mechanical disturbance and disrupt shellfish farms, resulting in loss of animals and aquaculture gear. Inundation from storm events can lead to increased siltation in areas where muddy and silty sediments are present. Shellfish, although tolerant to salinity fluctuations, may be affected by extreme or sudden changes in salinity following storm and precipitation events, especially in combination with ocean acidification (Dickinson et al. 2012). Mollusks are sensitive to harmful algal blooms, which are associated with warming seawater temperatures (Gobler et al. 2017) and eutrophication and nutrient over-enrichment that can increase exposure to reductions in dissolved oxygen (Howarth et al. 2011). Pollution or chemical contaminants from anthropogenic activity may degrade habitat around shellfish farms. Farmed shellfish and associated gear may be relocated in response to suboptimal conditions, but availability of leased grounds and economic considerations can limit the ability of aquaculture operations to respond to changes in habitat quality (Allison et al. 2011). Site selection will play an important role in mitigating the effects of climate on marine shellfish aquaculture.

Literature Cited

Allison EH, Badjeck M-C, Meinhold K. 2011. The implications of global climate change for molluscan aquaculture. Chapter 17, pp. 461-490. *In*: Shellfish Aquaculture and the Environment (SE Shumway,ed.). John Wiley & Sons, Inc.

Burge CA, Eakin MC, Friedman CS, Froelich B, Hershberger PK, Hofmann EE, Petes LE, Prager KC, Weil E, Willis BL, Ford SE, Harvell CD. 2014. Climate change influences on marine infectious diseases: implications for management and society. Annual Review of Marine Science 6(1):249-77.

Burreson EM, Stokes NA, Friedman CS. 2000. Increased virulence in an introduced pathogen: *Haplosporidium nelsoni* (MSX) in the eastern oyster *Crassostrea virginica*. Journal of Aquatic Animal Health 12(1):1-8.

Clements JC, Chopin T. 2016. Ocean acidification and marine aquaculture in North America: potential impacts and mitigation strategies. Reviews in Aquaculture 9(4):326-41.

Dickinson GH, Ivanina AV, Matoo OB, Pörtner HO, Lannig G, Bock C, Beniash E, Sokolova IM. 2012. Interactive effects of salinity and elevated CO_2 levels on juvenile eastern oysters, *Crassostreavirginica.* Journal of Experimental Biology 215:29-43.

Ekstrom JA, Suatoni L, Cooley SR, Pendleton LH, Waldbusser GG, Cinner JE, Portela R. 2015. Vulnerability and adaptation of US shellfisheries to ocean acidification. Nature Climate Change5(3):207-14.

Ford SE, Smolowitz R. 2007. Infection dynamics of an oyster parasite in its newly expanded range. Marine Biology 151(1):119-33.

Gazeau F, Parker LM, Comeau S, Gattuso J-P, O'Connor WA, Martin S, Pörtner HO, Ross PM. 2013. Impacts of ocean acidification on marine shelled molluscs. Marine Biology 160:2207-45.

Gledhill DK, White MM, Salisbury J, Thomas H, Misna I, Liebman M, Mook B, Grear J, Candelmo AC, Chambers RC, Gobler CJ, Hunt C., King A., Price NN, Signorini SR, Stancioff E, Stymiest C, WahleRA, Waller JD, Rebuck ND, Wang ZA, Capson DL, Morrison JR, Cooley SR, Doney SC. 2015. Ocean and coastal acidification off New England and Nova Scotia. Oceanography 28(2):182-97.

Gobler CJ, Doherty OM, Hattenrath-Lehmanna TK, Griffith AW, Kanga Y, Litaker RW. 2017. Ocean warming since 1982 has expanded the niche of toxic algal blooms in the North Atlantic and North Pacific oceans. Proceedings of the National Academy of Science 114(19):4975-80. doi:10.1073/pnas.1619575114

Howarth R, Chan F, Conley DJ, Garnier J, Doney SC, Marino R, Billen G. 2011. Coupled biogeochemical cycles: eutrophication and hypoxia in temperate estuaries and coastal marine ecosystems. Frontiers in Ecology and the Environment 9(1):18-26.

Jones SJ, Lima FP, Wethey DS. 2010. Rising environmental temperatures and biogeography: poleward range contraction of the blue mussel, *Mytilus edulis* L., in the western Atlantic. Journal of Biogeography 37(12):2243-59.

Lesser MP. 2016. Climate change stressors cause metabolic depressions in the blue mussel, *Mytilus edulis*, from the Gulf of Maine. Limnology and Oceanography 61:1705-17.

Kroeker KJ, Kordas RL, Crim R, Hendriks IE, Ramajo L, Singh GS, Duarte CM, Gattuso J-P. 2013. Impacts of ocean acidification on marine organisms: quantifying sensitivities and interaction with warming. Global Change Biology 19(6):1884-96.

Kurihara, H. 2008. Effects of CO_2 driven ocean acidification on the early developmental stages of invertebrates. Marine Ecology Progress Series 373:275–84.

Shumway SE, David C, Downey R, Karney R, Kraeuter J, Parson J, Rheault R, Wikfors G. 2003. Shellfish aquaculture—In praise of sustainable economies and environments. World Aquaculture Society 34(4):15-8.

Smolowitz R. 2013. A review of current state of knowledge concerning *Perkinsus marinus* effects on *Crassostrea virginica* (Gmelin) (the Eastern oyster). Veterinary Pathology 50(3):404-11.

Snyder J, Boss E, Weatherbee R, Thomas AC, Brady D and Newell C. 2017. Oyster aquaculture site selection using Landsat 8-derived sea surface temperature, turbidity, and chlorophyll a. Frontiers in Marine Science 4:190. doi:10.3389/fmars.2017.00190

U.S. Global Change Research Program (USGCRP). 2017. Climate Science Special Report: Fourth National Climate Assessment. Volume I. [Wuebbles, D.J., D.W. Fahey, K.A. Hibbard, D.J. Dokken, B.C. Stewart, and T.K. Maycock (eds.)]. U.S. Global Change Research Program. Washington, DC.USA. 470 pp. doi: 10.7930/J0J964J6.

Waldbusser GG, Voigt EP, Bergschneider H, Green MA, Newell RIE. 2011. Biocalcification in the eastern oyster (*Crassostrea virginica*) in relation to long-term trends in Chesapeake Bay pH. Estuaries and Coasts 34:221-31.

Marine Artificial Structures

System: Marine

Subsystem: Subtidal & Intertidal

Class: Rocky Bottom

Sub-class: Artificial Structures

Geographic Area: Entire Area

Overall Vulnerability Rank = Low

Habitat Sensitivity = Low

Climate Exposure = High

	**Marine Artificial Structures**	Attribute Mean	Data Quality	Distribution of Expert Scores
Sensitivity Attributes	Habitat condition	1.6	1.8	
	Habitat fragmentation	1.8	1.8	
	Distribution/Range	1.7	2.2	
	Mobility/Ability to spread or disperse	1.5	2.4	
	Resistance	1.6	2.2	
	Resilience	1.5	1.8	
	Sensitivity to changes in abiotic factors	1.7	1.8	
	Sensitivity and intensity of non−climate stressors	1.6	2	
	Dependency on critical ecological linkages	1.4	1.2	
	Sensitivity Component Score	Low		
Exposure Factors	Sea surface temp	3.5	3	
	Bottom temp	n/a	n/a	
	Air temp	n/a	n/a	
	River temp	n/a	n/a	
	Surface salinity	1.2	2.8	
	Bottom salinity	n/a	n/a	
	pH	4	2.2	
	Sea level rise	2.2	2.2	
	Precipitation	n/a	n/a	
	Floods	n/a	n/a	
	Droughts	n/a	n/a	
	Exposure Component Score	High		
**Overall Vulnerability Rank**		**Low**		

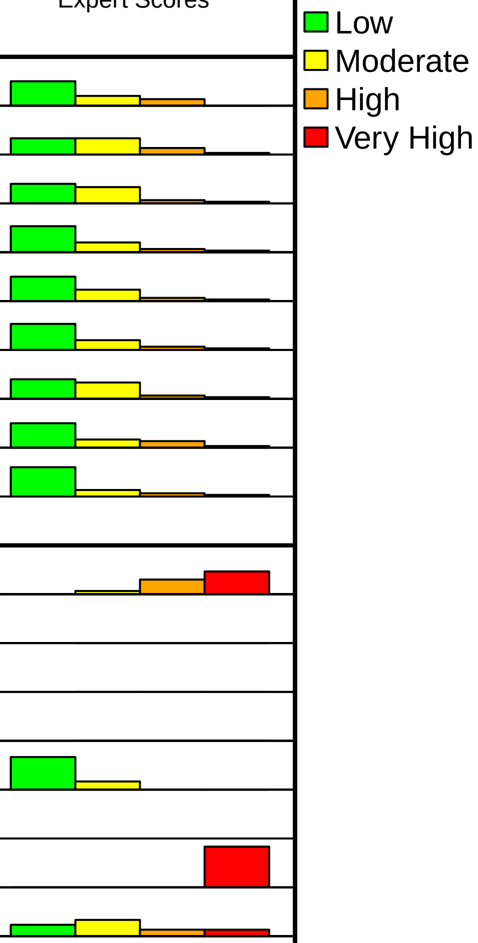

Habitat Name: Marine Artificial Structures
System: Marine
Subsystem: Subtidal and Intertidal
Class: Rocky Bottom
Sub-class: Artificial Structures
Geographic Area: Entire Area

Habitat Description: This sub-class includes artificial fishing reefs and wrecks, groins/jetties, riprap,living shorelines, and other artificial structures in the marine system. Riprap and breakwaters are common throughout the study area in populated areas. Artificial reefs are more common in the Mid-Atlantic than New England. This habitat includes the epibenthic flora and fauna associated with artificial structures, including calcareous algae (but not non-calcareous algae, which are included in marine aquatic bed habitat narrative).

Overall Climate Vulnerability Rank: **Low** (100% certainty from bootstrap analysis).

Climate Exposure: High. Two exposure factors contributed to the High exposure score: Sea Surface Temperature (3.5) and pH (4.0). The exposure attribute mean for Surface Salinity was 1.2, and Sea Level Rise (SLR) was 2.2. pH is projected to drastically decrease from historic levels for the entire study area by the end-of-century (increasing ocean acidification). Projected changes in Sea Surface Temperature are high to very high throughout the range.

Habitat Sensitivity: **Low**. All sensitivity attributes scored <2.0 (ranging from 1.4 to 1.8).

Data Quality & Gaps: Two of the exposure factors had high data quality (Sea Surface Temperature at 3.0 and Surface Salinity at 2.8), while the other two (pH and SLR) had scores of 2.2. Information about the distribution of artificial structures is limited, and the diversity of artificial structures includedin this habitat make it difficult to assess climate vulnerability in a uniform manner. Data quality is lower in general for intertidal and nearshore subtidal habitats due to the low resolution of CMIP5 and ROMS-NWA projections for climate exposure.

Five of the nine sensitivity attribute data quality scores are ≤2.0. The attributes with the highest data quality scores were Mobility/Ability to Spread or Disperse (2.4), and Distribution/Range and Resistance (both 2.2). Dependency on Critical Ecological Linkages scored 1.2, and Sensitivity to Changes in Abiotic Factors, Habitat Condition, Habitat Fragmentation, and Resilience all scored 1.8. These low data quality scores suggest moderate to high uncertainty in the response of artificial marine habitats.

Positive or Negative Climate Effect in the Northeast U.S.: The effect of climate change on marine intertidal rocky bottom in the Northeast U.S. is expected to be neutral (65% of the experts' scores were neutral, 25% were negative, and 10% were positive). The divergence in directional effects scores may be due to the diversity of artificial structures included in this single habitat type. For example, artificial reefs, riprap, and breakwaters are expected to have minimal impacts from climate change, and some flora and fauna associated with artificial structures (e.g., invasive species) may increase in abundance. Additionally, living shorelines may be more resilient to climate change (e.g., sea level rise, wave energy) than riprap.

Climate Effects on Habitat Condition and Distribution: Artificial subtidal and intertidal hard structures are not expected to be significantly impacted by climate change as compared with natural habitats. Structures like riprap and jetties are designed to deflect, withstand, or absorb wave action. However, artificial reefs located in shallow waters may be impacted by large storms, which are expected to increase in frequency and intensity in the Northeast region (USGCRP 2017). Some fauna associated with subtidal artificial structures are sensitive to climate impacts including increasing temperatures, changing salinity, and high wave energy.

Intertidal organisms are generally more adapted to extreme conditions (e.g., temperature, salinity) than those in the subtidal zone. Mollusk species associated with intertidal rocky habitats are sensitive to low pH, with larger negative effects on survival for larval stages than adults (Kroeker et al. 2013). Ocean acidification is expected to reduce the shell size, thickness, calcification rates and growth, and survival of embryo and larval bivalves and gastropods (Gazeau et al. 2013). Kroeker et al. (2013) reported echinoderms will experience negative effects on their growth and survival, with the largest effects on growth for larval stages.

Increased shoreline hardening in heavily populated and industrialized areas is expected in an attempt to reduce erosion and provide protection from storm surge as climate change effects become more severe and widespread (Balouskas and Targett 2018). Titus et al. (2009) estimated that almost 60% of land within 1 m above the high tide line on the U.S. Atlantic coast (Florida to Massachusetts) is developed or expected to be developed.

Living shorelines, or shoreline structures that incorporate natural habitat elements into their design (such as wetland vegetation, seagrasses, coarse woody debris, or shellfish reefs), may be more resilient to sea level rise and storm surge than vegetated habitats alone, depending on their setting, design, and maintenance (Mitchell and Bilkovic 2019). For example, planted marshes may be able to spread inland if migration corridors are available, and they can accrete vertically to address higher sea levels. Hybrid designs that incorporate vegetation and non-living material may be better able to withstand wave action (Browne and Chapman 2017). However, as storms and sea level rise increasingly impact shorelines, a human response may be to replace the living shoreline with harder structures.

<u>Habitat Summary</u>: The functional value of artificial hard structure as habitat is generally inherently poor, with lower species diversity, habitat complexity, and habitat function compared with natural rocky habitats (Balouskas and Targett 2018; Bulleri and Chapman 2010). Artificial shorelines can impact the growth, interactions, larval dispersal, and foraging behavior of species that live on them (e.g., limpets), and alter fish assemblages in adjacent waters (Browne and Chapman 2017). The make-up of biological communities on riprap, breakwaters, and other artificial structures differs from natural rocky habitats (Bulleri and Chapman 2010). They support fewer mobile species and more sessile animals and algae as compared with natural intertidal rocky habitats (Chapman 2003).

The material and design of engineered shorelines are important for their function as habitat, and the biodiversity of species they support. For example, sandstone has been found to support a higher diversity of species than concrete, and surveys have found more mobile species and algae on seawalls with sun exposure, and more sessile animals on shaded seawalls (Browne and Chapman 2017). Low structural complexity of engineered stone structures may limit habitat value for mobile species, which may be partly ameliorated by adding pits, crevices, and water-retaining features to seawalls (Browne and Chapman 2017). Incorporating natural habitat elements into artificial shoreline structures can improve habitat function (Bulleri and Chapman 2010). For example, studies have found that a higher density of oysters on seawalls provides better habitat and refuge for limpets and whelks (Jackson et al. 2008; Klein et al. 2011).Low species diversity may limit the resilience of the biotic community associated with artificial structures.

The presence of artificial hard structures in the Mid-Atlantic Bight has increased over the last two centuries, with shipwrecks constituting one of the most abundant types of artificial reef habitat (Steimleand Zetlin 2000; NOAA 2020). While patchy in distribution, some of these structures support biological communities including invertebrates, algae, and fish (Steimle and Zetlin 2000). As offshore wind development rapidly expands along the Northeast coast, these structures may act as artificial reefs (Glarou et al. 2020).

Flora and fauna associated with artificial rocky habitat may be sensitive to pollution and eutrophication similar to natural habitats, although invasive species may be less sensitive. Some studies suggest a higher incidence

of invasive species associated with artificial structures compared to native material(Tyrrell and Byers 2007; Pappal 2010; Geraldi et al. 2013). This may be associated with greater survival of invasive species on disturbed areas, but it could also suggest higher planktonic stage survival and greater dispersal capacity.

Artificial hard structures are highly resistant to disturbance. In fact, many shoreline structures are built to protect shorelines and withstand disturbance. Non-mobile (fouling) species that attach to riprap and rocky breakwaters re-colonize quickly following disturbance. Artificial reef habitats may be sensitive to siltation and burial, damage from fishing gear, pollution, removal, and water quality degradation (Steimle and Zetlin 2000).

Literature Cited

Balouskus RG, Targett TE. 2018. Impact of armored shorelines on shore-zone fish density in a Mid-Atlantic estuary: modulation by hypoxia and temperature. Estuaries and Coasts 41(Suppl 1):S144-S58.

Browne MA, Chapman MG. 2017. The ecological impacts of reengineering artificial shorelines: the state of the science. In Bilkovic DM, Mitchell MM, La Peyre MK, Toft JD (Eds). Living shorelines: the science and management of nature-based coastal protection (First Edition, 439-457). Boca Raton. https://doi.org/10.1201/9781315151465.

Bulleri F, Chapman MG. 2010. The introduction of coastal infrastructure as a driver of change in marine environments. Journal of Applied Ecology 47(1):26-35.

Chapman MG. 2003. Paucity of mobile species on constructed seawalls: effects of urbanization on biodiversity. Marine Ecology Progress Series 264:21–9.

Gazeau F, Parker LM, Comeau S, Gattuso J-P, O'Connor WA, Martin S, Pörtner H-O, Ross PM. 2013. Impacts of ocean acidification on marine shelled molluscs. Marine Biology 160(8):2207-45.

Geraldi NR, Smyth AR, Piehler MF, Peterson CH. 2013. Artificial substrates enhance non-native macroalga and N2 production. Biological Invasions 16(9):1819-31.

Glarou M, Zrust M, Svendsen JC. 2020. Using artificial-reef knowledge to enhance the ecological function of offshore wind turbine foundations: Implications for fish abundance and diversity. Journal of Marine Science and Engineering 8:332.

Jackson AC, Chapman MG, Underwood AJ. 2008. Ecological interactions in the provision of habitat by urban development: Whelks and engineering by oysters on artificial seawalls. Austral Ecology 33:307–16.

Klein JC, Underwood AJ, Chapman MG. 2011. Urban structures provide new insights into interactions among grazers and habitat. Ecological Applications 21:427-38.

Kroeker KJ, Kordas RL, Crim R, Hendriks IE, Ramajo L, Singh GS, Duarte CM, Gattuso J-P. 2013. Impacts of ocean acidification on marine organisms: quantifying sensitivities and interaction with warming. Global Change Biology 19(6):1884-96.

Mitchel M, Bilkovic DM. 2019. Embracing dynamic design for climate-resilient living shorelines. Journal of Applied Ecology 56(5):1099-105. https://doi.org/10.1111/1365-2664.13371.

NOAA Automated Wreck and Obstruction Information System (NOAA). 2020. [Internet]. Coasts Survey's

Wrecks and Obstructions. Automated Wreck and Obstruction Information System (AWOIS). (Accessed September 2020). https://wrecks.nauticalcharts.noaa.gov/viewer/.

Pappal A. 2010. Marine invasive species. State of the Gulf of Maine Report. [Currier P, Gould D, Hertz L, Huston J, Tremblay ML (eds)]. The Gulf of Maine Council on the Marine Environment. 1-21.

Steimle FW, Zetlin C. 2000. Reef habitats in the Middle Atlantic Bight: abundance, distribution, associated biological communities, and fishery resource use. Marine Fisheries Review 62(2):24-42.

Titus JG, Hudgens DE, Trescott DL, Craghan M, Nuckols WH, Hershner CH, Kassakian JM, Linn CJ, Merritt PG, McCue TM, O'Connell JF, Tanski J, Wang J. 2009. State and local governments plan for development of most land vulnerable to rising sea level along the US Atlantic coast. Environmental Research Letters 4(4):1-7.

Tyrrell MC, Byers JE. 2007. Do artificial substrates favor nonindigenous fouling species over native species? Journal of Experimental Marine Biology and Ecology 342(1):54-60.

U.S. Global Change Research Program (USGCRP). 2017. Climate Science Special Report: Fourth National Climate Assessment. Volume I [Wuebbles, D.J., D.W. Fahey, K.A. Hibbard, D.J. Dokken, B.C. Stewart, and T.K. Maycock (eds.)]. U.S. Global Change Research Program. Washington, DC.USA. 470 pp. doi: 10.7930/J0J964J6.

Marine Shallow Inner Shelf Water Column

System: Marine

Subsystem: Subtidal <20m

Class: Water Column

Sub-class: Shallow Inner Shelf

Geographic Area: Entire Area

Overall Vulnerability Rank = Low

Habitat Sensitivity = Low

Climate Exposure = High

	Marine Shallow Inner Shelf Water Column	Attribute Mean	Data Quality	Distribution of Expert Scores
Sensitivity Attributes	Habitat condition	1.9	2.4	
	Habitat fragmentation	1.3	2.6	
	Distribution/Range	1.2	2.6	
	Mobility/Ability to spread or disperse	1.2	2.6	
	Resistance	2	2.4	
	Resilience	1.1	2.4	
	Sensitivity to changes in abiotic factors	2.5	2.4	
	Sensitivity and intensity of non−climate stressors	2.4	2.4	
	Dependency on critical ecological linkages	1.6	2	
	Sensitivity Component Score	Low		
Exposure Factors	Sea surface temp	3.6	3	
	Bottom temp	n/a	n/a	
	Air temp	n/a	n/a	
	River temp	n/a	n/a	
	Surface salinity	1.2	2.8	
	Bottom salinity	n/a	n/a	
	pH	4	2.2	
	Sea level rise	n/a	n/a	
	Precipitation	2.2	2.1	
	Floods	n/a	n/a	
	Droughts	n/a	n/a	
	Exposure Component Score	High		
**Overall Vulnerability Rank**		**Low**		

Low
Moderate
High
Very High

Habitat Name: Marine Shallow Inner Shelf Water Column

System: Marine

Subsystem: Subtidal <20 m

Class: Water Column

Sub-class: Shallow Inner Shelf

Geographic Area: Entire Area

Habitat Description: This sub-class includes the shallow inner shelf water (<20 m water depth), which is vertically well-mixed year-round. The water column is a concept used in oceanography to describe the physical (e.g., temperature, salinity, light penetration) and chemical (e.g., pH, dissolved oxygen, nutrients, salts) characteristics of seawater at different depths. Water column habitats create the foundation for marine food webs, home to primary producers such as phytoplankton and microbes. These habitats are highly dynamic and exhibit swift responses to environmental variables.

Overall Climate Vulnerability Rank: Low (87% certainty from bootstrap analysis). Although the majority of the bootstrap results match the results of the categorical vulnerability rank, 13% of the bootstrap results were in the Moderate vulnerability rank.

Climate Exposure: High. The overall High climate exposure score was influenced by two Very High exposure means: Sea Surface Temperature and pH (3.6 and 4.0, respectively). The exposure factor scores for Surface Salinity and Precipitation were 1.2 and 2.2, respectively. Although the projected change in pH for the Gulf of Maine is less than in southern New England and the Mid-Atlantic, it is a significant decrease (>5.5 standard deviation) in pH from historic levels.

Habitat Sensitivity: Low. Only two of the nine sensitivity attribute means were ≥2.0: Sensitivity to Changes in Abiotic Factors (2.8) and Sensitivity and Intensity of Non-Climate Stressors (2.4). Habitat Condition (1.9) was the next highest scoring attribute.

Data Quality & Gaps: The data quality scores for two (Surface Salinity and Sea Surface Temperature) of the four climate exposure factors were relatively high (2.8 and 3.0, respectively). Precipitation and pH data quality scores were relatively low at 2.1 and 2.2, respectively, which may be attributed to the low resolution of CMIP5 and ROMS-NWA projections for climate exposure in nearshore, shallow coastal areas.

For habitat sensitivity, data quality for eight of the nine attributes were deemed Moderate (2.6 and 2.4). The data quality score for Dependency on Critical Ecological Linkages was 2.0. This suggests a moderate degree of confidence in the response of the water column to the physical and chemical factors that characterize the habitat.

Positive or Negative Climate Effect for the Northeast U.S.: The effect of climate change on marine inner shelf water column in the Northeast U.S. is expected to be mostly negative (80% of the experts'scores were negative and 20% neutral). However, the climate directionality is not consistent with the overall Low vulnerability rank or the exposure sensitivity component score.

Climate Effects on Habitat Condition and Distribution: Water is a universal component of marine ecosystems and is the habitat or a dominant component of the habitat for all marine organisms. Characteristics of water column habitat include temperature, salinity, dissolved oxygen, carbonate chemistry, nutrients, and primary and secondary producers. Characteristics also include currents and stratification/mixing.

Water on the inner shelf is very dynamic and impacted by air-sea exchange, inputs from terrestrial environments through freshwater runoff and estuarine flows, and mixing offshore marine waters. Currents are

largely driven by wind, tides, and buoyancy fluxes (e.g., freshwater inputs). Owing to the shallow nature of inner shelf waters, wind-driven and tidal mixing are very important and result in a generally well-mixed habitat. However, freshwater/estuarine plumes from the region's large river and estuarine systems can create stratification extending from the mouths of river and estuaries.

Owing to this dynamic nature, the characteristics of the habitat are also dynamic varying on a number of time scales. Temperature is largely influenced by heat exchange with the atmosphere and freshwater input, the temperature of which is also influenced by heat exchange with the atmosphere (Hare et al. 2010). That said, temperature is also related to exchange with waters offshore and thus the temperature of inner-shelf water is somewhat mediated compared to the temperature of the overlaying air (Shearman and Lentz 2010). As the atmosphere warms with climate change, the temperature of inner shelf waters will also warm.

Inner-shelf waters represent a boundary between shelf waters and estuarine waters and thus, the salinity is intermediate. As mentioned above, large plumes of estuarine water can occur on the inner shelf resulting in relatively low salinities (Marmorino et al. 2000). The absence of plumes results in relative higher, more marine salinities. Increases in precipitation and river flow (Demaria et al. 2016) will result in increases in estuarine water discharge onto the shelf, which would result in more episodes of decreased salinity on theinner shelf (Najjar et al. 2010).

Inner-shelf waters are relatively well-mixed but stratification can occur due to influences from estuarine plumes (Rennie et al. 1999). Increases in freshwater input related to climate change could result in increased frequency and magnitude of freshwater input into the inner shelf, with a consequent increase in stratification (Najjar et al. 2010). This could have consequences for oxygen-levels on the inner shelf.

Hypoxia does occur in estuarine systems throughout the Northeast largely as a result of summertime thermal stratification and increased primary production (Nixon et al. 2009). There is little evidence that stratification on the inner shelf can result in reductions in oxygen, but increased frequency and magnitude of freshwater plumes on the inner shelf could lead to decreases in oxygen in the future.

Carbonate chemistry on the inner-shelf is complicated owing to freshwater input, which supplies dissolved inorganic and dissolved and particulate organic carbon, primary production which uses CO_2, respiration which produces carbon dioxide, and increases in atmospheric CO_2, which dissolves into seawater causing ocean acidification. These factors and associated changes in carbonate chemistry are termed coastal acidification (Gledhill et al. 2015) and as a result of these various factors, carbonate chemistry in coastal waters exhibits a higher frequency of variability compared to shelf and oceanic waters (Waldbusser and Salisbury 2014). Carbonate chemistry in coastal waters is further complicated by eutrophication, which is an increase in nutrient supply from freshwater input that results in increased primary production. Subsequent respiration can drive up local CO_2 concentrations and drive down local O_2 concentrations. Thus, eutrophication is linked to hypoxia and anoxia in many estuarine systems in theregions (Wallace et al. 2014). In addition, nearshore coastal waters are generally more susceptible to acidification than oceanic waters because they are subject to more acid sources and are generally less buffered than oceanic waters (i.e., differences in the amount of dissolved inorganic carbon, dissolved and particulate organic carbon, total alkalinity and nutrients from riverine and estuarine sources) (Waldbusser et al. 2011; Wallace et al. 2014; Ekstrom et al. 2015; Gledhill et al. 2015). The long-term increase in atmospheric CO_2 will lead to increases in dissolved inorganic carbon and decreases in coastal pH, but the magnitude of the shorter-term variability associated with freshwater input, eutrophication, primary production, and respiration will dominate carbon carbonate chemistry.

Habitat Summary: Shallow inner shelf water column habitat has a high climate exposure owing to projected changes in sea surface temperature and pH. Sea surface temperature increases are projected throughout the

region, particularly in inner shelf regions. Ocean acidification projections also indicate large changes in pH in the future. These changes could be somewhat mediated because of the other factors that affect carbonate chemistry in coastal systems (Gledhill et al. 2015).

Despite two moderately high sensitivity attributes, shallow inner shelf water column habitat has an overall low sensitivity. This low sensitivity is attributed to good habitat condition (EPA 2012), continuous occurrence throughout the region, no barriers to movement (Beardsley and Winant 1979), and high resilience owing to advection and mixing (Lentz 2010; Gawarkiewicz et al. 2012).

One of the moderately sensitive attributes was sensitivity to abiotic stressors. Atmospheric warming leads to marine waters warming (Chen et al. 2014). Increased nutrients lead to increased productivity and decreased oxygen in bottom waters (Wallace et al. 2014). Increased freshwater inflow results in decreasesin salinity (Lentz 2010) and more acid sources and less buffered water (Wallace et al. 2014; Gledhill et al. 2015). This Sensitivity to Changes in Abiotic Factors represents the habitat's highest score in terms of sensitivity to climate change.

Shallow inner shelf water column habitat is also moderately sensitive to non-climatic stressors. For inner shelf waters thus sensitivity arises from freshwater inflow and sand mining. The Northeast region has a number of large rivers and estuaries and nutrients and contaminants from terrestrial sources enter the inner shelf water column habitat (Oczkowski et al. 2016). Sand mining also occurs in inner habitats and has a number of adverse impacts on habitat quality (Byrnes et al. 2004).

Literature Cited

Beardsley RC, Winant CD. 1979. On the mean circulation in the Mid-Atlantic Bight. Journal of Physical Oceanography 9(3):612-9.

Byrnes MR, Hammer RM, Thibaut TD, Snyder DB. 2004. Effects of sand mining on physical processes and biological communities offshore New Jersey, USA. Journal of Coastal Research 20(1):25-43.

Chen K, Gawarkiewicz GG, Lentz SJ, Bane JM. 2014. Diagnosing the warming of the Northeastern US Coastal Ocean in 2012: A linkage between the atmospheric jet stream variability and ocean response. Journal of Geophysical Research: Oceans 119(1):218-27.

Demaria EMC, Palmer RN, Round JK. 2016. Regional climate change projections of streamflow characteristics in the Northeast and Midwest U.S. Journal of Hydrology: Regional Studies 5:309-23.

Ekstrom JA, Suatoni L, Cooley SR, Pendleton LH, Waldbusser GG, Cinner JE, Ritter J, Langdon C, van Hooidonk R, Gledhill D, Wellman K, Beck MW, Brander LM, Rittschof D, Doherty C, Edwards PET, Portela R. 2015. Vulnerability and adaptation of US shellfisheries to ocean acidification. Nature Climate Change 5(3):207-14.

Gawarkiewicz G, Todd RE, Pluoddemann A.J, Andres M, Manning JP. 2012. Direct interaction between the Gulf Stream and the shelbreak south of New England. Scientific Reports 2:553. https://doi.org/10.1038/srep00553

Gledhill D, White M, Salisbury J, Thomas H, Misna I, Liebman M, Mook B, Grear J, Candelmo A, Chambers RC, Gobler C, Hunt C, King A, Price N, Signorini S, Stancioff E, Stymiest C, Wahle R, Waller J, Rebuck N, Wang Z, Capson T, Morrison JR, Cooley S, Doney S. 2015. Ocean and coastal acidification off New England and Nova Scotia. Oceanography 25(2):182-97.

Hare JA, Alexander MA, Fogarty MJ, Williams EH, Scott JD. 2010. Forecasting the dynamics of a coastal fishery species using a coupled climate–population model. Ecological Applications 20(2):452-64.

Lentz S J. 2010. The mean along-isobath heat and salt balances over the Middle Atlantic Bight continental shelf. Journal of Physical Oceanography 40(5):934-48.

Marmorino GO, Donato TF, Sletten MA, Trump C L. 2000. Observations of an inshore front associated with the Chesapeake Bay outflow plume. Continental Shelf Research 20(6):665-84.

Najjar RG, Pyke CR, Adams MB, Breitburg D, Hershner C, Kemp M, Howarth R, Mulholland MR, Paolisso M, Secor D, Sellner K, Wardrop D, Wood R. 2010. Potential climate-change impacts on the Chesapeake Bay. Estuarine, Coastal and Shelf Science 86(1):1-20.

Nixon SW, Fulweiler RW, Buckley BA, Granger SL, Nowicki BL, Henry KM. 2009. The impact of changing climate on phenology, productivity, and benthic–pelagic coupling in Narragansett Bay.Estuarine, Coastal and Shelf Science 82(1):1-18.

Oczkowski A, Kreakie B, McKinney RA, Prezioso J. 2016. Patterns in stable isotope values of nitrogen and carbon in particulate matter from the Northwest Atlantic continental shelf, from the Gulf of Maine to Cape Hatteras. Frontiers in Marine Science 3:252.

Rennie SE, Largier JL, Lentz SJ. 1999. Observations of a pulsed buoyancy current downstream of Chesapeake Bay. Journal of Geophysical Research: Oceans 104(C8):18227-40.

Shearman RK, Lentz SJ. 2010. Long-term sea surface temperature variability along the U.S. east coast. Journal of Physical Oceanography 40(5):1004-17.

U.S. Environmental Protection Agency (EPA). 2012. National Coastal Condition Report IV. EPA-R-842-10-003. US Environmental Protection Agency, Washington D.C. https://www.epa.gov/sites/production/files/2014-10/documents/0_nccr_4_report_508_bookmarks.pdf

Wallace RB, Baumann H, Grear JS, Aller RC, Gobler CJ. 2014. Coastal ocean acidification: The other eutrophication problem. Estuarine, Coastal and Shelf Science 148:1-13.

Waldbusser GG, Salisbury JE. 2014. Ocean acidification in the coastal zone from an organism's perspective: multiple system parameters, frequency domains, and habitats. Annual Review of Marine Science 6(1):221-47. https://doi.org/10.1146/annurev-marine-121211-172238

Waldbusser GG, Voigt EP, Bergschneider H, Green MA, Newell RIE. 2011. Biocalcification in the eastern oyster (*Crassostrea virginica*) in relation to long-term trends in Chesapeake Bay pH. Estuaries and Coasts 34(2):221-31.

Marine Shelf Surface Water Column

System: Marine

Subsystem: Subtidal <200m

Class: Water Column

Sub-class: Shelf Surface

Geographic Area: Entire Area

Overall Vulnerability Rank = Low

Habitat Sensitivity = Low

Climate Exposure = High

	**Marine Shelf Surface Water Column**	Attribute Mean	Data Quality	Distribution of Expert Scores
Sensitivity Attributes	Habitat condition	1.7	2.2	
	Habitat fragmentation	1.1	2.6	
	Distribution/Range	1.1	2.6	
	Mobility/Ability to spread or disperse	1.4	2.6	
	Resistance	1.8	2	
	Resilience	1.2	2	
	Sensitivity to changes in abiotic factors	2.6	2.4	
	Sensitivity and intensity of non−climate stressors	1.6	2.4	
	Dependency on critical ecological linkages	1.5	2	
	Sensitivity Component Score	Low		
Exposure Factors	Sea surface temp	3.4	3	
	Bottom temp	n/a	n/a	
	Air temp	n/a	n/a	
	River temp	n/a	n/a	
	Surface salinity	1.2	3	
	Bottom salinity	n/a	n/a	
	pH	4	2.4	
	Sea level rise	n/a	n/a	
	Precipitation	n/a	n/a	
	Floods	n/a	n/a	
	Droughts	n/a	n/a	
	Exposure Component Score	High		
**Overall Vulnerability Rank**		**Low**		

Low
Moderate
High
Very High

Habitat Name: Marine Shelf Surface Water Column

System**:** Marine

Subsystem: Subtidal <200 m

Class: Water Column

Sub-class: Shelf Surface

Geographic Area: Entire Area

Habitat Description: This sub-class includes the shelf surface water (<200 m water depth) above the seasonal thermocline with salinities >30 and <34 ppt. The water column is a concept used in oceanography to describe the physical (e.g., temperature, salinity, light penetration) and chemical (e.g., pH, dissolved oxygen, nutrients, salts) characteristics of seawater at different depths. Water column habitats create the foundation for marine food webs, home to primary producers such as phytoplankton and microbes. These habitats are highly dynamic and exhibit swift responses to environmental variables.

Overall Climate Vulnerability Rank: Low (100% certainty from bootstrap analysis).

Climate Exposure: High. Two of the climate exposure means were High: Sea Surface Temperature and pH (3.4 and 4.0, respectively). The exposure attribute score for Surface Salinity was Low (1.2). Ocean acidification (decreasing pH) is expected to drastically decrease from historic levels for the entire study area by the end of the century.

Habitat Sensitivity: Low. Only one of the nine sensitivity attribute means was relatively High: Sensitivity to Changes in Abiotic Factors (2.6). All of the other attribute means were between 1.1 and 1.8.

Data Quality & Gaps: The climate exposure data quality scores for Surface Salinity and Sea Surface Temperature were High (3.0 for both), and was Moderate for pH (2.4), suggesting relatively strong confidence in the climate projections for this habitat. It should be noted, however, that some dynamic water column features, such as the cold pool, warm-core rings, and large-scale currents, were not explicitly included in the scoring process.

For habitat sensitivity, data quality for five of the nine attributes were deemed Moderate (2.6 or 2.4), while the other attributes were scored low (2.2 or 2.0). This suggests a moderate degree of confidence in the response of the water column to the physical and chemical factors that characterize the habitat.

Positive or Negative Climate Effect for the Northeast U.S.: The effect of climate change on marine shelf surface water column habitat in the Northeast U.S. is expected to be mostly negative (75% of the experts' scores were negative and 25% neutral). However, this climate directionality is not consistent with the overall Low vulnerability rank or the sensitivity component score.

Climate Effects on Habitat Condition and Distribution: Water is a universal component of marine ecosystems and is the habitat or a dominant component of the habitat for all marine organisms. Characteristics of water column habitat include temperature, salinity, dissolved oxygen, carbonate chemistry, nutrients, and primary and secondary producers. Characteristics also include dynamic elements such as currents and stratification/mixing.

Circulation on the shelf is affected by two large-scale currents: the Labrador Coastal Current and the Gulf Stream (Loder 1998). Over the past several decades the influence of Labrador Sea water on the ecosystem has been decreasing and the influence of the Gulf Stream has been increasing. The deep flow into the ecosystem has changed from a large component of Labrador Sea influenced water (Labrador Slope Water) to predominantly Gulf Stream influenced water (Warm Slope Water); in 2017 and 2019, 99% of the water entering the Northeast Channel was Warm Slope Water (NEFSC 2020). In addition, the Gulf Stream has

shifted northward (Nye et al. 2011), the number of Gulf Stream warm-core rings has increased (Gangopadhyay et al. 2019), and interactions between warm-core rings and the shelf are being documented (Gawarkiewicz et al. 2012). These changes may in part be related to a weakening of the Atlantic Meridional Overturning Circulation (Joyce and Zhang 2010) and may be related to changes in temperature and salinity of shelf waters (see below).

In addition to large-scale advective forcing, surface shelf waters in the ecosystem are affected by air-sea exchange, wind and tidal mixing, and freshwater inflow. In the winter in the Mid-Atlantic, shelf waters are vertically well mixed through the water column (Li et al. 2015). In the winter in the Gulf of Maine, waters are partially well mixed; in the deep basins, winter mixing does not extend to the bottom (Brownand Irish 1993). In the summer, surface heating forms a strong thermocline through much of the ecosystem, separating surface waters from bottom waters (in the Mid-Atlantic) and from intermediate waters in the Gulf of Maine (Hopkins and Garfield 1979; Li et al. 2015). In some areas, however, tidal mixing is strong enough to keep the water column well mixed throughout the year (e.g., Georges Bank) (Loder et al. 1993). Winter winds are increasing in magnitude, which is increasing winter mixing (Schofield et al. 2008).

Surface temperatures across the region have been warming rapidly in recent decades (Pershing et al. 2015). A study of the 2012 marine heat wave concluded that the atmosphere was the predominant source of warming (Chen et al. 2014). Changes in the relative influence of the cold Labrador Coastal Current and warm Gulf Stream may also play a role (Shearmen and Lentz 2010). Part of the warming over the past several decades is related to the Atlantic Multidecadal Oscillation, which is a 40°–80°N pattern of warming and cooling across the North Atlantic (Kerr 2000), and part of this warming is related to long-term climate change (Chen et al. 2020). The pattern in surface warming also differs across seasons,with greater warming in summer and less warming in winter (Friedland and Hare 2007); this implies an increase in the seasonal range of temperatures in surface waters.

Salinity of shelf waters was decreasing for several decades (mid-1970s to mid-1990s), but in recent decades has been stable or increasing (mid-1990s to mid-2010s) (Wallace et al. 2018). There are several explanations for these patterns including changes in freshwater input and changes in source water from cold Labrador-origin water to warm Gulf Stream-origin water (Saba et al. 2016). Decreases in salinity from the 1970's through the 1990's was linked to increased volume transport from the Labrador Sea (Greene and Pershing 2007). Increases in salinity from 2000 have been linked to increased influence of the Gulf Stream on the ecosystem (Gangopadhyay et al 2019).

With climate change, the ocean is expected to acidify resulting from the oceanic adsorption of CO_2, a weak acid (Doney et al. 2009). In coastal and shelf ecosystems, however, other factors influence carbonate chemistry including freshwater input and primary production (Gledhill et al. 2015). In general, aragonite saturation state is somewhat lower in surface waters of the Gulf of Maine compared to the Mid-Atlantic making the region more susceptible to impacts of future decreases in pH (Wanninkhof et al.2015).

Habitat Summary: Shelf surface water column habitat has a high climate exposure component score, but a low climate vulnerability rank due to a low climate sensitivity attribute component score. Changes in pH and changes in sea surface temperature presented the highest exposure. Ocean acidification projections indicate large changes in pH in the future. These changes could be somewhat mediated because of the other factors that affect carbonate chemistry in coastal systems (Gledhill et al. 2015). However, as stated above, aragonite saturation is lower in surface waters of the Gulf of Maine indicating that these habitats are susceptible to future decreases in pH (Wanninkhof et al. 2015).

Surface temperatures are projected to increase throughout the region. However, the climate projections used in this assessment (i.e., CMIP5 and ROMS-NWA), do not explicitly include changes in circulation, which may lead to enhanced warming in the ecosystem (Saba et al. 2016).

The highest scored sensitivity attribute mean was Sensitivity to Changes in Abiotic Factors. Surface water characteristics change as the abiotic environment changes. Surface waters are subject to a number of abiotic influences including air-sea exchange and freshwater inflow. Changes in these factors will lead to changes in surface water properties. An excellent example is the 2012 heatwave, where changes in the atmosphere translated to the ocean (Chen et al. 2014).

Literature Cited

Brown WS, Irish JD. 1993. The annual variation of water mass structure in the Gulf of Maine:1986–1987. Journal of Marine Research 51(1):53-107.

Chen K, Gawarkiewicz GG, Lentz SJ, Bane JM. 2014. Diagnosing the warming of the Northeastern US Coastal Ocean in 2012: A linkage between the atmospheric jet stream variability and ocean response. Journal of Geophysical Research: Oceans 119(1):218-27.

Chen Z, Kwon YO, Chen K., Fratantoni P, Gawarkiewicz G, Joyce TM. 2020. Long-term SST variability on the Northwest Atlantic Continental Shelf and Slope. Geophysical Research Letters 47(1):e2019GL085455.

Doney SC, Fabry VJ, Feely RA, Kleypas JA. 2009. Ocean acidification: the other CO2 problem. Annual Review of Marine Science 1:169-92.

Friedland KD, Hare JA. 2007. Long-term trends and regime shifts in sea surface temperature on the continental shelf of the northeast United States. Continental Shelf Research 27(18):2313-28.

Gangopadhyay A, Gawarkiewicz G, Silva ENS, Monim M, Clark J. 2019. An observed regime shift in the formation of warm core rings from the Gulf Stream. Scientific Reports 9(1):1-9.

Gawarkiewicz G, Todd RE, Pluoddemann A.J, Andres M, Manning JP. 2012. Direct interaction between the Gulf Stream and the shelbreak south of New England. Scientific Reports 2:553. https://doi.org/10.1038/srep00553

Gledhill D, White M, Salisbury J, Thomas H, Misna I, Liebman M, Mook B, Grear J, Candelmo A, Chambers RC, Gobler C, Hunt C, King A, Price N, Signorini S, Stancioff E, Stymiest C, Wahle R, Waller J, Rebuck N, Wang Z, Capson T, Morrison JR, Cooley S, Doney S. 2015. Ocean and coastal acidification off New England and Nova Scotia. Oceanography 25(2):182-97.

Greene CH, Pershing AJ. 2007. Climate drives sea change. Science 315(5815):1084-5.

Hopkins TS, Garfield III N. 1979. Gulf of Maine intermediate water. Journal of Marine Research 37(1):103-39.

Joyce TM, Zhang R. 2010. On the path of the Gulf Stream and the Atlantic meridional overturning circulation. Journal of Climate 23(11):3146-54.

Kerr RA. 2000. A North Atlantic climate pacemaker for the centuries. Science 288(5473):1984-5.

Li Y, Fratantoni PS, Chen C, Hare JA, Sun Y, Beardsley RC, Ji R. 2015. Spatio-temporal patterns of stratification on the Northwest Atlantic shelf. Progress in Oceanography 134:123-37.

Loder JW. 1998. The coastal ocean off northeastern North America: A large-scale view. The Sea 11:105-38.

Loder JW, Drinkwater KF, Oakey NS, Horne EP. 1993. Circulation, hydrographic structure and mixing at tidal fronts: the view from Georges Bank. Philosophical Transactions of the Royal Society of London. Series A: Physical and Engineering Sciences 343(1669):447-60.

Northeast Fisheries Science Center (NEFSC). 2020. 2020 State of the Ecosystem Mid-Atlantic. https://repository.library.noaa.gov/view/noaa/23889 (last accessed 9 November 2020).

Nye JA, Joyce TM, Kwon Y-O, Link JS. 2011. Silver hake tracks changes in Northwest Atlantic circulation. Nature Communications 2:6.

Pershing AJ, Alexander MA, Hernandez CM, Kerr LA, Le Bris A, Mills KE, Nye JA, Record NR, Scannell HA, Scott JD, Sherwood GD, Thomas AC. 2015. Slow adaptation in the face of rapid warming leads to collapse of the Gulf of Maine cod fishery. Science 350(6262):1-8.

Saba VS, Griffies SM, Anderson WG, Winton M, Alexander MA, Delworth TL, Hare JA, Harrison MJ, Rosati A, Vecchi GA, Zhang R. 2016. Enhanced warming of the Northwest Atlantic Ocean under climate change. Journal of Geophysical Research: Oceans 121(1):118-32.

Schofield O, Chant R, Cahill B, Castelao R, Gong D, Kahl A, Kohut J, Montes-Hugo M, Ramadurai R, Ramey P, Yi X, Glenn S. 2008. The decadal view of the Mid-Atlantic Bight from the COOLroom: is our coastal system changing? Oceanography 21(4):108-17.

Shearman RK, Lentz SJ. 2010. Long-term sea surface temperature variability along the U.S. east coast. Journal of Physical Oceanography 40(5):1004-17.

Wallace EJ, Looney LB, Gong D. 2018. Multi-decadal trends and variability in temperature and salinity in the Mid-Atlantic Bight, Georges Bank, and Gulf of Maine. Journal of Marine Research 76(5):163-215.

Wanninkhof R, Barbero L, Byrne R, Cai W-J, Huang W-J, Zhang J-Z, Baringer M, Langdon C. 2015. Ocean acidification along the Gulf coast and the East coast of the USA. Continental Shelf Research 98:54-71.

Marine Shelf Bottom Water Column

System: Marine

Subsystem: Subtidal <200m

Class: Water Column

Sub-class: Shelf Bottom

Geographic Area: Entire Area

Overall Vulnerability Rank = Low

Habitat Sensitivity = Low

Climate Exposure = Moderate

	Marine Shelf Bottom Water Column	Attribute Mean	Data Quality	Distribution of Expert Scores
Sensitivity Attributes	Habitat condition	1.6	2.2	
	Habitat fragmentation	1.6	2.6	
	Distribution/Range	1.1	2.6	
	Mobility/Ability to spread or disperse	1.3	2.6	
	Resistance	1.8	2	
	Resilience	1.3	2	
	Sensitivity to changes in abiotic factors	2.5	2.4	
	Sensitivity and intensity of non−climate stressors	1.5	2.4	
	Dependency on critical ecological linkages	1.5	2	
	Sensitivity Component Score	Low		
Exposure Factors	Sea surface temp	n/a	n/a	
	Bottom temp	2.8	3	
	Air temp	n/a	n/a	
	River temp	n/a	n/a	
	Surface salinity	1	n/a	
	Bottom salinity	1.2	3	
	pH	4	2.4	
	Sea level rise	n/a	n/a	
	Precipitation	n/a	n/a	
	Floods	n/a	n/a	
	Droughts	n/a	n/a	
	Exposure Component Score	Moderate		
**Overall Vulnerability Rank**		**Low**		

Low
Moderate
High
Very High

Habitat Name: Marine Shelf Bottom Water Column

System**:** Marine

Subsystem: Subtidal <200 m

Class: Water Column

Sub-class: Shelf Bottom

Geographic Area: Entire Area

Habitat Description: This sub-class includes the shelf bottom water (<200 m water depth) below seasonal thermocline with salinities >30 ppt and <34 ppt. The water column is a concept used in oceanography to describe the physical (e.g., temperature, salinity, light penetration) and chemical (e.g., pH, dissolved oxygen, nutrients, salts) characteristics of seawater at different depths. Water column habitats create the foundation for marine food webs, home to primary producers such as phytoplankton and microbes. These habitats are highly dynamic and exhibit swift responses to environmental variables.

Overall Climate Vulnerability Rank: Low (100% certainty from bootstrap analysis).

Climate Exposure: Moderate. Two of the climate exposure means were relatively High: Bottom Temperature and pH (2.8 and 4.0, respectively). The exposure attribute score for Bottom Salinity was Low (1.2). Ocean acidification (decreasing pH) is expected to drastically increase from historic levels for the entire study area by the end of the century.

Habitat Sensitivity: Low. Only one of the nine sensitivity attribute means was relatively high: Sensitivity to Changes in Abiotic Factors (2.5). All of the other attribute means were between 1.1 and 1.8.

Data Quality & Gaps: The climate exposure data quality scores for Bottom Salinity and Bottom Temperature were High (3.0 for both), and was Moderate for pH (2.4), suggesting relatively strong confidence in the climate projections for this habitat. It should be noted, however, that some dynamic water column features, such as the cold pool, warm-core rings, and large-scale currents, were not explicitly included in the scoring process.

For habitat sensitivity, data quality for five of the nine attributes were deemed Moderate (2.6 and 2.4), while the other attributes were scored low (2.2 or 2.0). This suggests a moderate degree of confidence in the response of the water column to the physical and chemical factors that characterize the habitat for most of the habitat attributes.

Positive or Negative Climate Effect for the Northeast U.S.: The effect of climate change on marine shelf bottom water column habitats in the Northeast U.S. is expected to be mostly negative (75% of the experts' scores were negative and 25% were neutral). However, this climate directionality is not consistent with the overall Low vulnerability rank or the sensitivity component score.

Climate Effects on Habitat Condition and Distribution: Water is a universal component of marine ecosystems and is the habitat or a dominant component of the habitat for all marine organisms. Characteristics of water column habitat include temperature, salinity, dissolved oxygen, carbonate chemistry, nutrients, and primary and secondary producers. Characteristics also include dynamic elements such as currents and stratification / mixing.

Water currents on the shelf are affected by two large-scale currents: the Labrador Coastal Current and the Gulf Stream (Loder 1998). Over the past several decades, the influence of Labrador Sea water on the ecosystem has been decreasing and the influence of the Gulf Stream has been increasing. The deep flow into the ecosystem has changed from a large component of Labrador Sea influenced water (Labrador Slope Water) to predominantly Gulf Stream influenced water (Warm Slope Water); in 2017 and 2019, 99% of the water

entering the Northeast Channel was Warm Slope Water (NEFSC 2020). In addition, the Gulf Stream has shifted northward (Nye et al. 2011), the number of Gulf Stream warm-core rings has increased (Gangopadhyay et al. 2019), and interactions between warm-core rings and the shelf are being documented (Gawarkiewicz et al. 2012). These changes may in part be related to a weakening of the Atlantic Meridional Overturning Circulation (Joyce and Zhang 2010) and have caused changes in temperature and salinity of shelf waters (see below).

The dynamics of bottom waters in the Mid-Atlantic Bight and Gulf of Maine are different. The Mid-Atlantic Bight shelf is well-mixed in the winter but highly stratified in the summer (Li et al. 2015). Thus, bottom waters in the summer retain winter water characteristics; this feature is termed the “cold pool” and extends from south of Rhode Island to Virginia (Houghton et al. 1982). The Gulf of Maine exhibits three-layered stratification: Maine Surface Water, Maine Intermediate Water, and Maine Bottom Water (Brown and Irish 1993). Maine Surface Water is influenced by freshwater runoff, Scotian Shelf water, and is subjected to seasonal heating and cooling. The shallow bound of Maine Intermediate Water is the seasonal thermoline and deep bound is the depth of winter mixing. Thus, Maine Intermediate Wateris analogous to the “cold pool” and exhibits winter-like temperatures in the summer. Maine Bottom Water is influenced by deep inflow through the Northeast Channel and is warmer and saltier than Maine Intermediate Water (Hopkins and Garfield 1979); the characteristics of Maine Bottom Water are dependent on the source of the inflow: Labrador Slope Water or Warm Atlantic Slope Water (see above). These regional differences will have some effect on how climate change affects bottom waters: seasonal dynamics of the “cold pool” in the Mid-Atlantic and winter mixing and inflow through the Northeast Channel in the Gulf of Maine.

Wind-forcing also affects bottom waters largely through Ekman transport driving upwelling along the Mid-Atlantic shelf in the summer and wind mixing breaking down the seasonal thermocline in the fall and keeping the shelf well mixed through the winter and early spring. Winter winds are increasing in magnitude, which is increasing winter mixing (Schofield et al. 2008).

Bottom temperatures across the region have been warming. Bottom water temperatures in the Mid-Atlantic Bight are increasing and the “cold pool” is becoming weaker (Miller et al. 2016). Bottom waters in the Gulf of Maine are also warming with geographic variability in the rate (Goode et al. 2019). This warming is likely a result of increased heat content from the atmosphere and changes in source waterin the region: from cold Labrador-origin water to warm Gulf Stream-origin water (Saba et al. 2016).

Salinity of shelf waters was decreasing for several decades (mid-1970s to mid-1990s), but in recent decades has been stable or increasing (mid-1990s to mid-2010s) (Wallace et al. 2018). There are severalexplanations for these patterns including changes in freshwater input and changes in source water from cold Labrador-origin water to warm Gulf Stream-origin water (Saba et al. 2016). Decreases in salinity from the 1970’s through the 1990’s was linked to increased volume transport from the Labrador Sea (Greene and Pershing 2007). Increases in salinity from 2000 have been linked to increased influence of the Gulf Stream on the ecosystem (Gangopadhyay et al 2019).

With climate change, the ocean is expected to acidify resulting from the oceanic adsorption of CO_2, a weak acid (Doney et al. 2009). In coastal and shelf ecosystems, however, other factors influence carbonate chemistry including freshwater input and primary production (Gledhill et al. 2015). Aragonite saturation is already low in bottom waters in the Mid-Atlantic and Gulf of Maine indicating that thesehabitats are highly susceptible to decreases in pH (Wanninkhof et al. 2015).

Hypoxia of bottom waters on the shelf rarely occurs, but can have large impacts on biology. A widespread hypoxia event occurred in 1976 and widespread mortalities of benthic invertebrates were documented (Swanson and Sindermann 1979). The causes of this event are still uncertain but early stratification occurred in that year. Earlier spring onset is occurring in the ecosystem (Staudinger et al. 2019), but the timing of stratification in the Mid-Atlantic has not been quantified. Acidification and low oxygen conditions are linked

via the process of respiration and this link is relevant to shelf bottom waters.

Habitat Summary: Shelf bottom water column habitat has a low climate exposure. Changes in pH and changes in bottom water temperature presented the highest climate exposure. Ocean acidification projections indicate large changes in pH in the future. These changes could be somewhat mediated because of the other factors that affect carbonate chemistry in coastal systems (Gledhill et al. 2015). However, as stated above, aragonite saturation is already low in bottom waters in the Mid-Atlantic and Gulf of Maine indicating that these habitats are highly susceptible to future decreases in pH (Wanninkhofet al. 2015).

Bottom temperatures are projected to increase throughout the region, but less so in bottom waters compared to surface waters. These projections, however, may not include the changes in circulation,which may lead to enhanced warming of bottom waters in the ecosystem resulting from changes in circulation (Saba et al. 2016).

The highest scored sensitivity attribute was sensitivity to abiotic stressors. Bottom water characteristics change as the abiotic environment changes. However, as a result of strong seasonal stratification, bottomwater is partially isolated from many of the abiotic factors affecting water column habitats in general. However, this isolation of bottom waters can increase the persistence of abiotic changes once they occur, as evidenced by the 1976 hypoxic event in the Mid-Atlantic (Swanson and Sindermann 1979).

Literature Cited

Brown WS, Irish JD. 1993. The annual variation of water mass structure in the Gulf of Maine:1986–1987. Journal of Marine Research 51(1):53-107.

Doney SC, Fabry VJ, Feely RA, Kleypas JA. 2009. Ocean acidification: the other CO_2 problem. Annual Review of Marine Science 1:169-92.

Gangopadhyay A, Gawarkiewicz G, Silva ENS, Monim M, Clark J. 2019. An observed regime shift in the formation of warm core rings from the Gulf Stream. Scientific Reports 9(1):1-9.

Gawarkiewicz G, Todd RE, Pluoddemann A.J, Andres M, Manning JP. 2012. Direct interaction between the Gulf Stream and the shelbreak south of New England. Scientific Reports 2:553. https://doi.org/10.1038/srep00553

Gledhill D, White M, Salisbury J, Thomas H, Misna I, Liebman M, Mook B, Grear J, Candelmo A, Chambers RC, Gobler C, Hunt C, King A, Price N, Signorini S, Stancioff E, Stymiest C, Wahle R, Waller J, Rebuck N, Wang Z, Capson T, Morrison JR, Cooley S, Doney S. 2015. Ocean and coastal acidification off New England and Nova Scotia. Oceanography 25(2):182-97.

Goode AG, Brady DC, Steneck RS, Wahle RA. 2019. The brighter side of climate change: how local oceanography amplified a lobster boom in the Gulf of Maine. Global Change Biology 25(11):3906-17.

Greene CH, Pershing AJ. 2007. Climate drives sea change. Science 315(5815):1084-5.

Hopkins TS, Garfield III N. 1979. Gulf of Maine intermediate water. Journal of Marine Research 37(1):103-39.

Houghton RW, Schlitz R, Beardsley RC, Butman B, Chamberlin JL. 1982. The Middle Atlantic Bight cold pool: Evolution of the temperature structure during summer 1979. Journal of Physical Oceanography 12(10):1019-29.

Joyce TM, Zhang R. 2010. On the path of the Gulf Stream and the Atlantic meridional overturning circulation. Journal of Climate 23(11):3146-54.

Li Y, Fratantoni PS, Chen C, Hare JA, Sun Y, Beardsley RC, Ji R. 2015. Spatio-temporal patterns of stratification on the Northwest Atlantic shelf. Progress in Oceanography 134:123-37.

Loder JW. 1998. The coastal ocean off northeastern North America: a large-scale view. The Sea 11:105-38.

Miller TJ, Hare JA, Alade LA. 2016. A state-space approach to incorporating environmental effects on recruitment in an age-structured assessment model with an application to southern New England yellowtail flounder. Canadian Journal of Fisheries and Aquatic Sciences 73(8):1261-70.

Northeast Fisheries Science Center (NEFSC). 2020. [Internet]. 2020 State of the Ecosystem Mid-Atlantic. (Accessed 9 November 2020). https://repository.library.noaa.gov/view/noaa/23889.

Nye JA, Joyce TM, Kwon Y-O, Link JS. 2011. Silver hake tracks changes in Northwest Atlantic circulation. Nature Communications 2:6.

Saba VS, Griffies SM, Anderson WG, Winton M, Alexander MA, Delworth TL, Hare JA, Harrison MJ,Rosati A, Vecchi GA, Zhang R. 2016. Enhanced warming of the Northwest Atlantic Ocean under climate change. Journal of Geophysical Research: Oceans 121(1):118-32.

Schofield O, Chant R, Cahill B, Castelao R, Gong D, Kahl A, Kohut J, Montes-Hugo M, Ramadurai R, Ramey P, Yi X, Glenn S. 2008. The decadal view of the Mid-Atlantic Bight from the COOLroom: isour coastal system changing? Oceanography 21(4):108-17.

Staudinger MD, Mills KE, Stamieszkin K, Record NR, Hudak CA, Allyn A, Diamond A, Friedland KD, Golet W, Henderson ME, Hernandez CM, Huntington TG, Ji R, Johnson CL, Johnson DS, Jordaan A, Kocik J, Li Y, Liebman M, Nichols OC, Pendleton D, Richards R, Robben T, Thomas AC, Walsh H, Yakola K. 2019. It's about time: a synthesis of changing phenology in the Gulf of Maine ecosystem. Fisheries oceanography 28(5):532-66.

Swanson RL, Sindermann CJ (editors). 1979. Oxygen depletion and associated benthic mortalities in New York Bight, 1976. NOAA Professional Paper 11. National Oceanic and Atmospheric Administration. Rockville, MD. 343 pp.

Wallace EJ, Looney LB, Gong D. 2018. Multi-decadal trends and variability in temperature and salinity in the Mid-Atlantic Bight, Georges Bank, and Gulf of Maine. Journal of Marine Research 76(5):163-215.

Wanninkhof R, Barbero L, Byrne R, Cai W-J, Huang W-J, Zhang J-Z, Baringer M, Langdon C. 2015. Ocean acidification along the Gulf coast and the East coast of the USA. Continental Shelf Research 98:54-71.

Marine Slope Surface Water Column

System: Marine

Subsystem: Subtidal >200m

Class: Water Column

Sub-class: Slope Surface

Geographic Area: Entire Area

Overall Vulnerability Rank = Low

Habitat Sensitivity = Low

Climate Exposure = Low

	**Marine Slope Surface Water Column**	Attribute Mean	Data Quality	Distribution of Expert Scores
Sensitivity Attributes	Habitat condition	1.4	2	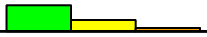
	Habitat fragmentation	1.3	2.4	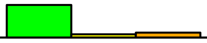
	Distribution/Range	1.1	2.6	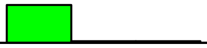
	Mobility/Ability to spread or disperse	1.2	2.6	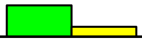
	Resistance	1.8	2	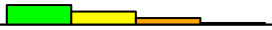
	Resilience	1.2	2	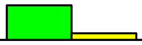
	Sensitivity to changes in abiotic factors	2.4	2.2	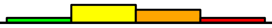
	Sensitivity and intensity of non−climate stressors	1.3	2.2	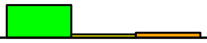
	Dependency on critical ecological linkages	1.5	1.8	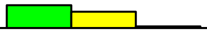
	Sensitivity Component Score	Low		
Exposure Factors	Sea surface temp	1.6	3	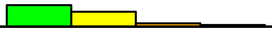
	Bottom temp	n/a	n/a	
	Air temp	n/a	n/a	
	River temp	n/a	n/a	
	Surface salinity	1.2	3	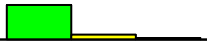
	Bottom salinity	n/a	n/a	
	pH	4	2.4	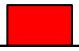
	Sea level rise	n/a	n/a	
	Precipitation	n/a	n/a	
	Floods	n/a	n/a	
	Droughts	n/a	n/a	
	Exposure Component Score	Low		
**Overall Vulnerability Rank**		**Low**		

Low
Moderate
High
Very High

Habitat Name: Marine Slope Surface Water Column

System**:** Marine

Subsystem: Subtidal >200 m

Class: Water Column

Sub-class: Slope Surface

Geographic Area: Entire Area

<u>Habitat Description</u>: This sub-class includes the slope waters on the upper 200 m of the water column onthe outer continental shelf and slope between Cape Hatteras and the Gulf of Maine with salinities >34 ppt.The water column is a concept used in oceanography to describe the physical (e.g., temperature, salinity, light penetration) and chemical (e.g., pH, dissolved oxygen, nutrients, salts) characteristics of seawater at different depths. Water column habitats create the foundation for marine food webs, home to primary producers such as phytoplankton and microbes. These habitats are highly dynamic and exhibit swift responses to environmental variables.

<u>Overall Climate Vulnerability Rank</u>: Low (100% certainty from bootstrap analysis).

<u>Climate Exposure</u>: Low. The only High climate exposure mean was pH at 4.0. The exposure means for Sea Surface Temperature and Surface Salinity were low (1.6 and 1.2, respectively). pH is projected to drastically decrease from historic levels for the entire study area by the end-of-century (increasing ocean acidification).

<u>Habitat Sensitivity</u>: Low. Only one of the nine sensitivity attribute mean scores was Moderate: Sensitivity to Changes in Abiotic Factors (2.4). All of the other attribute means were between 1.1 and 1.8.

<u>Data Quality & Gaps</u>: For climate exposure, the data quality scores for Surface Salinity and Sea Surface Temperature were High (3.0 for both), with a Moderate score for pH (2.4). This suggests somewhat strong confidence in the climate projections for this habitat. It should be noted, however, that some dynamic water column features, such as the cold pool, warm-core rings, and large-scale currents, were not explicitly included in the scoring process.

For habitat sensitivity, data quality for three of the nine attributes were deemed Moderate (2.6 or 2.4), while the other attributes were scored Low (between 1.8 and 2.2). Distribution/Range, Mobility/Ability to Spread or Disperse, and Habitat Fragmentation were the three highest scoring attributes for data quality.

<u>Positive or Negative Climate Effect in the Northeast U.S. Shelf</u>: The effect of climate change on marine slope surface water column is expected to be mostly negative (70% of the experts' scores were negative and 30% neutral). However, this climate directionality is not consistent with the overall Low vulnerability rank, or the exposure and sensitivity component scores.

<u>Climate Effects on Habitat Condition and Distribution</u>: The upper 200 m of the water column along the continental margin is directly affected by exchanges of temperature, oxygen, and carbon dioxide with the atmosphere. Climate-induced increases in air temperature and atmospheric emissions of CO_2 cause ocean water temperatures and acidity to increase. Using a high resolution model that assumes a doubling of CO_2 emissions, Saba et al. (2016) projected temperature increases of 3°C in the upper 300 m of the water column along the outer northwest Atlantic continental shelf by the year 2100, a rate of warming two to three times faster than the global average. As the upper water column warms, the thermocline strengthens and the euphotic zone becomes more isolated from nutrient-rich deeper water.

The reduction in nutrients required by phytoplankton reduces primary production which provides organic matter for the higher trophic levels of the food chain (Levin and Le Bris 2020). Gregg et al. (2003) estimated that global ocean primary production has declined by 6.3% starting in the early 1980s, corresponding to 0.2°C increase in sea surface temperature, with the largest declines in higher latitudes.

The temperature and salinity of the upper water column on the outer shelf and slope and in the Gulf of Maine are also affected by the interaction between colder, fresher Labrador Slope Water and warmer, more saline slope water that is associated with the Gulf Stream (Townsend et al. 2015; Wanninkhof et al. 2015; Saba et al. 2016), by meanders in the Gulf Stream (Gawarkiewicz et al. 2012), and by warm core rings that spin off the Gulf Stream and cause exchanges of warmer, more saline slope water with colder, fresher shelf water (Churchill et al. 2003; Zhang and Gawarkiewicz 2015).

Predicted changes in water temperature and salinity in the upper 200 m of the northwest Atlantic are partially dependent on how much the location and velocity of the Gulf Stream will change as it impinges on the shelf break and crosses the North Atlantic. Based on the CM2.6 climate model, surface salinities are predicted to increase by 1-1.5 practical salinity units (psu) over the continental slope by the end of the century and at the bottom by 0.7 to 0.8 psu in shallower water on the outer shelf and in water entering the Gulf of Maine (at 150-200 m) through the Northeast Channel (Saba et al. 2016). Future increases in salinity along the continental margin are caused by changes in water mass distribution that is related to the retreat of the Labrador Current, a northerly shift of the Gulf Stream, and an increased proportion of warmer Atlantic slope water entering the shelf (Saba et al. 2016). Long term changes caused by climate change may not affect the temperature and salinity of upper water column habitats on the outer shelf and slope as much as the episodic, short term effects of meanders in the Gulf Stream and the frequency, duration, and magnitude of warm core rings (see below).

In the winter, as shelf waters cool, a hydrographic front forms on the outer shelf and upper slope separating colder, fresher shelf water and warmer, saltier slope water, with a strong horizontal density gradient extending from the surface to the bottom in about 80 m of water (Burrage and Garvine 1988; Chapman and Gawarkiewicz 1993). In the summer, warming surface water creates strong vertical stratification with a strong pycnocline at a depth of about 20 m on the outer shelf and slope with weak horizontal gradients (no front) overlying a permanent thermocline between 200 and 600 m on the slope (Aikman 1984; Burrage and Garvine 1988). The shelf-slope front extends from Greenland to the Mid-Atlantic Bight and, although it varies in intensity and timing according to local conditions, the "foot" of the front always remains within 20 km of the shelf break (Frantantoni and Pickart 2007).

There is recent evidence that since 2000 slope water is being transported on to the shelf in the Mid-Atlantic Bight more often and with longer lasting effects (Gawarkiewicz et al. 2018). Warm core rings are occurring more frequently and lasting longer and Gulf Stream meanders are larger and extending farther on to the shelf, causing periodic onshore shifts in the front. One such event (a "marine heatwave") occurred in the winter of 2016-2017 causing water temperatures in southern New England to increase by 6°C; another one in the winter and early spring of 2017 just north of Cape Hatteras lasted for four months and increased water temperatures over a distance of 850 km in the Mid-Atlantic Bight (Gawarkiewicz et al. 2019). Inshore displacements of the front and the warming of shelf waters temporarily allows southern species to move north (Gawarkiewicz et al. 2019) and could have other biological effects such as shifting the seaward extent of migrating black sea bass and placing them in less suitable over-wintering feeding habitat (Miller et al. 2016). One of the climate models (CM2.6) predicts about a 50% reduction in the density of *Calanus finmarchicus*, an important component of the food chain and a food source for right whales, on the shelf break by 2081-2100 (Grieve et al. 2017). Species that are expected to experience the most extreme northward range extensions as ocean temperatures increase are the highly mobile species (e.g., elasmobranchs, cephalopods, and pelagic fish), some of which inhabit the upper water column on the outer shelf and slope (Welch and McHenry 2018).

The upper water column is more exposed than deeper water to atmospheric increases in CO_2, which causes acidity to increase (lower pH) and the solubility of calcium carbonate ($CaCO_3$) to decrease. Although near surface waters are more susceptible to increased acidity, they are supersaturated in aragonite, one of the mineral forms of $CaCO_3$ that many marine animals rely on to form shells and other calcified body parts (Orr et al. 2005; Fabry et al. 2008). Currently, the water column on the shelf between 50 and 200 m north of Cape

Hatterasis nearly under-saturated with respect to aragonite, reflecting the effects of colder coastal currents originating in the Labrador Sea and the Gulf of St. Lawrence (Wanninkhof et al. 2015). More saturated water associated with the Gulf Stream extends from the surface to 150 m at Cape Hatteras and is deeper water at the shelf break.

Climate change will cause a shoaling of the aragonite saturation horizon (Wanninkhof et al. 2015), and reduce the ability of planktonic organisms to form $CaCO_3$ (Riebesell et al. 2000). The impact will be less severe than in deeper benthic habitats where seawater is less saturated in aragonite and where there are more sessile animals with skeletal body parts that are unable to move into shallower water (except through larval dispersal).

Habitat Summary: Water temperatures in surface waters on the outer continental shelf and slope are highly sensitive to seasonal and climate-induced changes in air temperature. In the summer, warming surface water overlaying deeper more saline slope water creates a strong pycnocline at a depth of about 20 m. Near surface water that is already less dense than deeper water is, therefore, more sensitive to global warming than less stratified surface waters. As surface waters become even warmer, the pycnocline will intensify, further isolating the euphotic zone from inputs of vital nutrients required for photosynthesis that are produced in deeper water. Below the pycnocline, the upper water column (0-200 m) is less susceptible to increases in air temperature, but it is subjected to changes in temperature and salinity caused by periodic variations in southerly flowing cold bottom water (Labrador slope water) and northerly flowing warm, more saline Gulf Stream water, and by warm core rings that transfer warmer slope water on to the shelf and colder shelf water seaward (Gawarkiewicz et al. 2012; Townsend et al. 2015; Wanninkhof et al. 2015; Zhang and Gawarkiewicz 2015). Slope water is being transported on to the shelf in the Mid-Atlantic Bight more often and with longer lasting effects in recent years (Gawarkiewicz et al. 2018), indicating that pelagic habitat conditions in the upper water column along the continental margin are sensitive to climate-induced changes in circulation and water mass transport across the shelf break.

The upper water column has more exposure to increased ocean acidity than deeper water below 200 m because it is more directly exposed to increased atmospheric CO_2 emissions. Decreases in pH have a more pronounced effect on near-surface waters, but the upper water column is more saturated with aragonite than deeper water, facilitating the biomineralization of $CaCO_3$ (Fabry et al. 2008; Wanninkhof et al. 2015). Pelagic animals in the upper water column are, thus, less sensitive to increased acidity than they are in deeper water where aragonite saturation values are lower.

Literature Cited

Aikman III F. 1984. Pycnocline development and its consequences in the Middle Atlantic Bight. Journal of Geophysical Research 89:685-94.

Burrage DK, Garvine RW. 1988. Summertime hydrography at the shelfbreak front in the Middle Atlantic Bight. Journal of Physical Oceanography 18:1309-19.

Chapman DC, Gawarkiewicz G. 1993. On the establishment of the seasonal pycnocline in the Middle Atlantic Bight. Journal of Physical Oceanography. Note and Correspondence 23:2487-92.

Churchill JH, Manning JP , Beardsley RC. 2003. Slope water intrusions on to Georges Bank. Journal of Geophysical Research 108(8012). doi:10.1029/2002JC001400

Fabry VJ, Seibel BA, Feely RA, Orr JC. 2008. Impacts of ocean acidification on marine fauna and ecosystem processes. ICES Journal of Marine Science 65(3):414-32.

Frantantoni PS, Pickart RS. 2007. The western North Atlantic shelfbreak current system in summer. Journal of Physical Oceanography 37:2509-33.

Gawarkiewicz G, Todd RE, Pluoddemann A.J, Andres M, Manning JP. 2012. Direct interaction between the Gulf Stream and the shelfbreak south of New England. Scientific Reports 2:553. https://doi.org/10.1038/srep00553

Gawarkiewicz G, Todd RE, Zhang W, Partida J, Gangopadhyay A, Monim M-U-H, Fratantoni P, Mercer AM, Dent M. 2018. The changing nature of shelf-break exchange revealed by the OOI Pioneer Array. Oceanography 31(1):60–70. https://doi.org/10.5670/oceanog.2018.110

Gawarkiewicz G, Chen K, Forsyth J, Bahr F, Mercer AM, Ellertson A, Fratantoni P, Seim H, Haines S, Han L. 2019. Characteristics of an advective marine heatwave in the Middle Atlantic Bight in early 2017. Frontiers in Marine Science 6:14.

Gregg WW, Conkright ME, Ginoux P, O'Reilly JE, Casey NW. 2003. Ocean primary production and climate: global decadal changes. Geophysical Research Letters 30(15):1809.

Grieve BD, Hare JA, Saba VS. 2017. Projecting the effects of climate change on *Calanus finmarchicus* distribution within the U.S, Northeast Continental Shelf. Scientific Report 7:12.

Levin LA, Le Bris N. 2020. The deep ocean under climate change. Science 350(6262):766-8.

Miller AS, Shepherd GR, Frantantoni PS. 2016. Offshore habitat preference of overwintering juvenile and adult black sea bass, *Centropristis striata*, and the relationship to year-class success. PLoS One 11(1):eO147627. doi:10.1371/journal.pone.0147627.

Orr JC, Fabry VJ, Aumont O, Bopp L, Doney SC, Feely RA, Gnanadesikan A, Gruber N, Ishida A, Joos F, Key RM, Lindsay K, Maier-Reimer E, Matear R, Monfray P, Mouchet A, Najjar RG, Plattner G-K, Rodgers KB, Sabine CL, Sarmiento JL, Schlitzer R, Slater RD, Totterdell IJ, Weirig M-F, Yamanaka Y, Yool A. 2005. Anthropogenic ocean acidification over the twenty-first century and its impact on calcifying organisms. Nature 437(7059):681-6.

Riebesell U, Zondervan I, Rost B, Tortell PD, Zeebe RE, Morel FMM. 2000. Reduced calcification of marine plankton in response to increased atmospheric CO_2. Nature 407(6802):364-7.

Saba VS, Griffies SM, Anderson WG, Winton M, Alexander MA, Delworth TL, Hare JA, Harrison MJ, Rosati A, Vecchi GA, Zhang R. 2016. Enhanced warming of the Northwest Atlantic Ocean under climate change. Journal of Geophysical Research: Oceans 121(1):118-32.

Townsend DW, Pettigrew NR, Thomas MA, Neary MG, McGillicuddy DJ Jr, O'Donnell J. 2015. Water masses and nutrient sources to the Gulf of Maine. Journal of Marine Research 73(3-4):93-122. doi:10.1357/002224015815848811

Wanninkhof R, Barbero L, Byrne R, Cai W-J, Huang W-J, Zhang J-Z, Baringer M, Langdon C. 2015. Ocean acidification along the Gulf coast and the East coast of the USA. Continental Shelf Research 98:54-71.

Welch H, McHenry J. 2018. Planning for dynamic process: an assemblage-level surrogate strategy for

species seasonal movement pathways. Aquatic Conservation 28(2):337-50.

Zhang WG, Gawarkiewicz GG. 2015. Dynamics of the direct intrusion of Gulf Stream ring water onto the Mid-Atlantic Bight shelf. Geophysical Research Letters 42:7687–95. doi:10.1002/2015GL065530.

Marine Slope Bottom Water Column

System: Marine

Subsystem: Subtidal >200m

Class: Water Column

Sub-class: Slope Bottom

Geographic Area: Entire Area

Overall Vulnerability Rank = Low

Habitat Sensitivity = Low

Climate Exposure = Moderate

	**Marine Slope Bottom Water Column**	Attribute Mean	Data Quality	Distribution of Expert Scores
Sensitivity Attributes	Habitat condition	1.3	1.8	
	Habitat fragmentation	1.2	2	
	Distribution/Range	1.1	2.2	
	Mobility/Ability to spread or disperse	1.2	2.4	
	Resistance	2	2	
	Resilience	1.6	2	
	Sensitivity to changes in abiotic factors	2	2.2	
	Sensitivity and intensity of non−climate stressors	1.3	2.2	
	Dependency on critical ecological linkages	1.5	2	
	Sensitivity Component Score	Low		
Exposure Factors	Sea surface temp	n/a	n/a	
	Bottom temp	1.7	3	
	Air temp	n/a	n/a	
	River temp	n/a	n/a	
	Surface salinity	n/a	n/a	
	Bottom salinity	2.9	3	
	pH	4	2.4	
	Sea level rise	n/a	n/a	
	Precipitation	n/a	n/a	
	Floods	n/a	n/a	
	Droughts	n/a	n/a	
	Exposure Component Score	Moderate		
**Overall Vulnerability Rank**		**Low**		

Low
Moderate
High
Very High

Habitat Name: Marine Slope Bottom Water Column

System: Marine

Subsystem: Subtidal >200 m

Class: Water Column

Sub-class: Slope Bottom

Geographic Area: Entire Area

Habitat Description: This sub-class includes the slope waters between depths of 200 and 1000 m with salinities >30 ppt. The water column is a concept used in oceanography to describe the physical (e.g., temperature, salinity, light penetration), chemical (e.g., pH, dissolved oxygen, nutrients, salts), and biological characteristics of seawater at different depths. For this habitat sub-class, the focus is the deep (200-1000 m depths) water column on the outer continental shelf and slope from Cape Hatteras to the U.S.-Canada border on Georges Bank and in the deep basins of the Gulf of Maine.

Overall Climate Vulnerability Rank: Low (100% certainty from bootstrap analysis).

Climate Exposure: Moderate. The Moderate component score was driven by two of the three climate exposure means: Bottom Salinity and pH (2.9 and 4.0, respectively). The exposure attribute score for Bottom Temperature was low (1.7). pH is projected to drastically decrease from historic levels for the entire study area by the end-of-century (increasing ocean acidification). Note: climate change is also expected to reduce dissolved oxygen concentrations in deep water (Levin and Le Bris 2020).

Habitat Sensitivity: Low. All of the nine sensitivity attribute means were <2.0, and six were ≤1.5.

Data Quality & Gaps: For exposure factors, the data quality scores for Bottom Salinity and Bottom Temperature were high (3.0 for both), with a Moderate score for pH (2.4). This suggests relatively strong confidence in the climate projections for this habitat. It should be noted, however, that some dynamic water column features, such as the cold pool, warm-core rings, and large-scale currents, were not explicitly included in the scoring process.

For habitat sensitivity, all data quality scores were deemed Low to Moderate: one of the nine attributes were 1.8, while the other eight were between 2.0 to 2.4. This suggests a low to moderate degree of confidence in the response of the water column to the physical and chemical factors that characterize the habitat at these depths.

Positive or Negative Climate Effect in the Northeast U.S. Shelf: The effect of climate change on the deep marine water column is expected to be evenly split between neutral and negative (55% of the experts' scores were negative and 45% neutral).

Climate Effects on Habitat Condition and Distribution: Deep water column habitats are less dynamic than near-surface waters that are more directly affected by exchanges of temperature, oxygen, and carbon dioxide with the atmosphere. Although seasonal changes in temperature are limited to the upper 200 m onthe shelf (Mountain 2003; Richaud et al. 2016), changes in atmospheric conditions do have indirect effects on temperature, acidity (pH), and oxygen concentrations in deep water (Fabry et al. 2008; Levin and Le Bris 2020). Some of these properties of seawater are affected more than others depending on depthand latitude. The salinity, temperature, and acidity of deep water on the outer shelf and slope and in the Gulf of Maine is also affected by the interaction between colder, fresher Labrador Slope Water and warmer, more saline slope water that is associated with the Gulf Stream (Townsend et al. 2015; Wanninkhof et al. 2015; Saba et al. 2016), by meanders in the Gulf Stream (Gawarkiewicz et al. 2012), and by warm core rings that spin off the Gulf Stream and cause exchanges of warmer, more saline slope water with colder, fresher shelf water (Churchill et al. 2003; Zhang and Gawarkiewicz 2015). Long-term changes caused by global warming may

not affect the temperature and salinity of deep water column habitats on the outer shelf and slope as much as the episodic, short term effects of meanders in the Gulf Stream and the frequency, duration, and magnitude of warm core rings.

The effects of climate change on the temperature, salinity, pH, and oxygenation of the water column below 200 m at different locations along the outer continental shelf and slope and between the slope and the Gulf of Maine are likely to vary by depth and location due to latitudinal variations in the velocity and direction of the Gulf Stream north of Cape Hatteras and the interaction between warm slope water and colder Labrador slope water entering the Gulf of Maine (Frantantoni and Pickart 2007; Townsend et al. 2015; Saba et al. 2016). Future increases in salinity along the continental margin are caused by changes in water mass distribution that is related to the retreat of the Labrador Current, a northerly shift of the Gulf Stream, and an increased proportion of warmer Atlantic slope water entering the shelf (Saba et al. 2016).

Climate change will cause an increase in ocean acidification (lower pH) which, in turn, will reduce the concentrations of aragonite, one of the mineral forms of carbonate that in many marine animals is combined with calcium to form calcium carbonate ($CaCO_3$). The solubility of $CaCO_3$ increases in deep water owing to the combined effects of colder water and increased pressure (Fabry et al. 2008). Along the east coast of the U.S., ocean water in depths >200m north of Cape Hatteras is under-saturated, or only slightly super-saturated, with respect to aragonite (Wanninkhof et al. 2015). As more CO_2 is absorbed into the upper water column, the shoaling of the aragonite saturation horizon is expected to reduce the ability of benthic organisms along the outer continental shelf and slope to incorporate $CaCO_3$ into shells and other body parts (Fabry et al. 2008; Orr et al. 2005). Similar effects could be expected in deep-water basins in the Gulf of Maine. Sweetman et al. (2017) concluded that the most significant changes in pH are expected in the 200 to 3000 meter depth range in all oceans, with increased acidity of 0.29-0.37 units.

Two additional effects of climate change that are expected to affect deep water column habitats that were not considered in this assessment are reduced dissolved oxygen concentrations and a reduction in the amount of particulate organic matter (POC). The combined effects of increased vertical stratification (due to the warming of surface waters) and reduced oxygen solubility in warmer water is expected to lead to widespread deoxygenation of oceanic waters, especially between 200 and 700 meters (Levin and Le Bris 2020). This could have a negative effect on pelagic animals that inhabit intermediate waters, especially those that are not able to move into more oxygenated waters. In addition, reduced amounts of POC in deep water would cause a reduction in bacterial action that produces nutrients which are essential for photosynthesis in the photic zone and lead to a reduction in primary production (Levin and Le Bris 2020).

Habitat Summary: Water column conditions at depths >200 m do not vary seasonally and are less directly affected by atmospheric conditions (e.g., increased air temperatures and absorption of oxygen and CO_2) than near-surface waters. However, deep water column conditions on the outer shelf and upper slope and at depths between 200 and 350 meters in the Gulf of Maine are currently subjected to changes in temperature and salinity caused by periodic variations in southerly flowing cold bottom water (Labrador slope water) and northerly flowing warm, more saline Gulf Stream water, and by warm core rings that transfer warmer slope water on to the shelf and colder shelf water seaward (Gawarkiewicz et al. 2012; Townsend et al. 2015; Wanninkhof et al. 2015; Zhang and Gawarkiewicz 2015). For this reason, it is likely that pelagic animals found in deep water column habitats on the outer shelf and slope between 200 and 1000m are less sensitive to climate-induced changes in temperature and salinity than animals in deeper slope waters where conditions are more stable.

Projected changes to the physical and chemical conditions of deep water column (>200 m) from climate change, including effects to water temperature, salinity, oxygen concentration, and carbonate saturation state (Orr et al. 2005; Fabry et al. 2008; Gawarkiewicz et al. 2012; Townsend et al. 2015; Wanninkhof et al. 2015; Zhang and Gawarkiewicz 2015) is expected to have variable effects to pelagic, demersal, and benthic animals

that occur there.

Literature Cited

Churchill JH, Manning JP , Beardsley RC. 2003. Slope water intrusions on to Georges Bank. Journal of Geophysical Research 108(8012). doi:10.1029/2002JC001400

Fabry VJ, Seibel BA, Feely RA, Orr JC. 2008. Impacts of ocean acidification on marine fauna and ecosystem processes. ICES Journal of Marine Science 65(3):414-32.

Frantantoni PS, Pickart RS. 2007. The western North Atlantic shelfbreak current system in summer. Journal of Physical Oceanography 37:2509-33.

Gawarkiewicz G, Todd RE, Pluoddemann A.J, Andres M, Manning JP. 2012. Direct interaction between the Gulf Stream and the shelfbreak south of New England. Scientific Reports 2:553. https://doi.org/10.1038/srep00553

Levin LA, Le Bris N. 2020. The deep ocean under climate change. Science 350(6262):766-8.

Mountain DG. 2003. Variability in the properties of Shelf Water in the Middle Atlantic Bight, 1977–1999. Journal of Geophysical Research 108(C1).

Orr JC, Fabry VJ, Aumont O, Bopp L, Doney SC, Feely RA, Gnanadesikan A, Gruber N, Ishida A, Joos F, Key RM, Lindsay K, Maier-Reimer E, Matear R, Monfray P, Mouchet A, Najjar RG, Plattner G-K, Rodgers KB, Sabine CL, Sarmiento JL, Schlitzer R, Slater RD, Totterdell IJ, Weirig M-F, Yamanaka Y, Yool A. 2005. Anthropogenic ocean acidification over the twenty-first century and its impact on calcifying organisms. Nature 437(7059):681-6.

Richaud, B., Y-O. Kwon, T.M. Joyce, P.S. Frantantoni, and S.J. Lentz. 2016. Surface and bottom temperature and salinity climatology along the continental shelf off te Canadian and U.S. east coasts. Continental Shelf Research 124:165-81.

Saba VS, Griffies SM, Anderson WG, Winton M, Alexander MA, Delworth TL, Hare JA, Harrison MJ, Rosati A, Vecchi GA, Zhang R. 2016. Enhanced warming of the Northwest Atlantic Ocean under climate change. Journal of Geophysical Research: Oceans 121(1):118-32.

Sweetman AK, Thurber AR, Smith CR, Levin LA, Mora C, Wei C-L, Gooday AJ, Jones DOB, Rex M, Yasuhara M, Ingels J, Ruhl HA, Frieder CA, Danovaro R, Würzberg L, Baco A, Grupe BM, Pasulka A, Meyer KS, Dunlop KM, Henry L-A, Roberts JM. 2017. Major impacts of climate change on deep-sea benthic ecosystems. Elementa Science of the Anthropocene 5:4. http://doi.org/10.1525/elementa.203

Townsend DW, Pettigrew NR, Thomas MA, Neary MG, McGillicuddy DJ Jr, O'Donnell J. 2015. Water masses and nutrient sources to the Gulf of Maine. Journal of Marine Research 73(3-4):93-122. doi:10.1357/002224015815848811

Wanninkhof R, Barbero L, Byrne R, Cai W-J, Huang W-J, Zhang J-Z, Baringer M, Langdon C. 2015. Ocean acidification along the Gulf coast and the East coast of the USA. Continental Shelf Research 98:54-71.

Zhang, W.G. and G. G. Gawarkiewicz. 2015. Dynamics of the direct intrusion of Gulf Stream ring water onto the Mid-Atlantic Bight shelf. Geophysical Research Letters 42:9.

Estuarine Invasive Wetland: Mid−Atlantic

System: Estuarine

Subsystem: Intertidal

Class: Emergent Wetland

Sub-class: Invasive Wetland

Geographic Area: Mid-Atlantic

Overall Vulnerability Rank = Moderate

Habitat Sensitivity = Low

Climate Exposure = Very High

	Estuarine Invasive Wetland: Mid−Atlantic	Attribute Mean	Data Quality	Distribution of Expert Scores
Sensitivity Attributes	Habitat condition	1.7	2.2	
	Habitat fragmentation	1.6	2	
	Distribution/Range	1.7	2.3	
	Mobility/Ability to spread or disperse	1.9	2.1	
	Resistance	1.5	2.3	
	Resilience	1.6	1.8	
	Sensitivity to changes in abiotic factors	1.6	2.1	
	Sensitivity and intensity of non−climate stressors	1.7	2	
	Dependency on critical ecological linkages	1.4	1.7	
	Sensitivity Component Score	Low		
Exposure Factors	Sea surface temp	n/a	n/a	
	Bottom temp	n/a	n/a	
	Air temp	4	3	
	River temp	n/a	n/a	
	Surface salinity	2.8	2.1	
	Bottom salinity	n/a	n/a	
	pH	4	2	
	Sea level rise	3.9	2.2	
	Precipitation	2.8	2.1	
	Floods	n/a	n/a	
	Droughts	n/a	n/a	
	Exposure Component Score	Very High		
	**Overall Vulnerability Rank**	**Moderate**		

Low
Moderate
High
Very High

Habitat Name: Estuarine Invasive Wetland: Mid-Atlantic

System**:** Estuarine

Subsystem: Intertidal

Class: Emergent Wetland

Sub-class: Invasive Wetland

Geographic Area: Mid-Atlantic

Habitat Description: This sub-class includes intertidal invasive estuarine emergent wetlands or salt marshes in the Mid-Atlantic region from eastern Long Island Sound south to Cape Hatteras, North Carolina. Salt marsh communities are characterized by distinct patterns of zonation tied to frequency and depth of tidal inundation. Salinity can range from full-strength seawater to brackish. This study considers both persistent and non-persistent wetlands.

Due to expected differences between tidal wetlands in New England and the Mid-Atlantic, the two regions were considered separately in this study. New England salt marshes have higher organic content (peat) and lower sediment inputs than Mid-Atlantic marshes (Nixon 1980; Charles and Dukes 2009). A balance between primary productivity and organic matter decomposition enables New England marshes to maintain elevation (Bricker-Urso et al. 1989), whereas Mid-Atlantic marshes are coastal plain-type marshes with higher sediment input and lower organic content (Nixon 1980).

Because ecosystem functions differ between the two types of marshes, New England and Mid-Atlantic marshes are further divided into native and invasive marshes for this study. Invasive species that dominate tidal marshes in both regions include an exotic haplotype of the common reed (*Phragmites australis*), narrow-leaf cattail (*Typha angustifolia*), and purple loosestrife (*Lythrum salicaria*), which are more common in brackish water.

Overall Climate Vulnerability Rank: Moderate (100 % certainty from bootstrap analysis).

Climate Exposure: Very High. Air Temperature (4.0), pH (4.0), and Sea Level Rise (SLR) (3.9) all contributed to the Very High climate exposure score. Air Temperature is projected to increase in the study area and pHis projected to decrease (increasing ocean acidification). SLR is expected to increase in the Northeast, with the greatest increase predicted for the Mid-Atlantic. Sea surface temperatures, which impact emergent wetlands in tandem with air temperature, are increasing at a faster rate in the Mid-Atlantic compared to the global mean, and this trend is projected to continue.

Habitat Sensitivity: Low. All sensitivity scores were <2.0. Anthropogenic impacts such as coastal development and shoreline hardening have contributed to declines in habitat condition and have exacerbated naturally occurring habitat fragmentation. However, marshes dominated by invasive plant species are typically less sensitive and more resilient to such impacts than are those dominated by nativespecies.

Data Quality & Gaps: Four of the five exposure data quality scores were Low to Moderate, between 2.0 and 2.2, with the highest score for Air Temperature (3.0). Tidal marshes in the Mid-Atlantic are generally well studied but impacts of climate change to these habitats, as well as differing responses between native and invasive marshes, need further investigation. In addition, data quality for exposure is lower for estuarine and intertidal habitats due to the low resolution of CMIPS and ROMS-NWA projects for climate exposure in estuaries and nearshore, shallow coastal areas.

Two of the nine sensitivity attribute data quality scores were less than 2.0. Resilience and Dependency on Critical Ecological Linkages scored 1.8 and 1.7, respectively. Distribution/Range and Resistance scored the highest (2.3).

Positive or Negative Climate Effect in the Northeast U.S.: The effect of climate change on invasive emergent wetlands in the Mid-Atlantic is expected to be primarily positive, with 75% of the experts' scores returned as positive, with 20% neutral and 5% negative.

Climate Effects on Habitat Condition and Distribution: In general, climate change is expected to benefit

invasive emergent wetland species and exacerbate their impacts on ecosystems (Dukes and Mooney 1999). *P. australis* and other invasive wetland plants are highly adapted to disturbance, and will likely benefit from climate change by outcompeting native salt marsh species (Rejmánek and Richardson 1996;Meyerson et al. 2000; Bertness et al. 2002; Mozdzer and Megoniga 2012; Smith 2013). However, climate change is still expected to have an impact on invasive salt marshes in several ways. Most salt marsh flora are eurythermal, and an increase in temperature may lead to an increase in photosynthetic rates, plant biomass, and other plant physiological processes (Charles and Dukes 2009; Gedan and Bertness 2010; Kirwan and Mudd 2012). *P. australis* exhibited a higher germination rate than a native saltmarsh halophyte, *Spartina patens*, at temperatures projected for the end of the century (Martin 2017). Although both the native *S. alterniflora* and non-native *P. australis* responded to higher experimental temperatures with increased growth, the non-native haplotype of *P. australis* outcompeted *S. alterniflora* when additional nutrients were added (Legault et al. 2018).

Temperature can have other indirect effects on emergent wetlands, such as on production of soil organic matter, rates of evaporation and decomposition, and salt marsh community composition (Najjar et al. 2000; Charles and Dukes 2009; Gedan and Bertness 2009; Gedan and Bertness 2010; Carey et al. 2017). The habitat is also sensitive to changes in the marsh platform, as an increase in temperature can cause an increase in decay rate of the organic matter in the platform, offsetting enhanced productivity and soil carbon accumulation associated with increased temperatures (Kirwan and Blum 2011). However, *P. australis* stands accumulate detritus and have slower stem decomposition rates (Meyerson et al. 2000), so subsidence is less of a concern than it is in native marshes.

The precise responses of coastal wetlands to increased warming is difficult to predict, given the complexity of interactions among biological and environmental factors and the coarse level of resolutionof landscape-scale models (Cahoon et al. 2009), but studies have found that modest warming can increase halophyte productivity (Charles and Duke 2009; Kirwan et al. 2009). Salt marshes are sensitive to atmospheric CO_2, and could increase productivity as atmospheric CO_2 rises, although there are a number of confounding factors influencing this. Salt marshes are nitrogen-limited and are sensitive to nitrogen inputs (especially anthropogenic eutrophication, see below). Groundwater serves as a nitrogeninput to marshes and is affected by precipitation in the watershed (Valiela et al. 1978; Gardner and Reeves 2002).

Sea level rise has increased in the last several decades, which has led to marsh degradation and loss of habitat (Kearney et al. 2002; Crosby et al. 2016; Watson et al. 2017a; Watson et al. 2017b). Regional predictions of sea level rise show that levels will be highest in the Mid-Atlantic. Increased erosion of the marsh edge could result from the synergistic effects of temperature-mediated decomposition of organic matter in the marsh platform coupled with increased wave height and strength due to SLR and increased frequency of storms. Inundation and edge erosion may also be exacerbated by a decrease in accretion rate due to loss of sediment input from upstream retention of sediment by dams and decline in agriculture (Weston 2014), or a decline in soil organic matter due to higher decomposition rates fueled by higher water temperature (Carey et al. 2017). Increased edge erosion can result from a greater porosity of the marsh platform from increased decomposition, coupled with the increase in wave energy. Salt marsh erosion shows a linear response to wave intensity (Leonardi et al. 2016). However, invasive species such as *P. australis* tend to occupy higher-elevation zones in coastal marshes and lower-salinity, low-energy habitats in the upper reaches of estuaries, so would be less sensitive to impacts from marsh-edge erosion and a decrease in accretion rate.

Habitat Summary: Invasive plant species share characteristics that make it easy for them to overtake existing wetland communities (Dukes and Mooney 1999). These include a high rate of population growth, which contributes to rapid colonization; ability to move long distances, which contributes to colonizing distant habitats; tolerance of close association with humans; and tolerance of a broad range ofphysical conditions (Rejmánek and Richardson 1996). Invasive species typically tolerate disturbed areas better than natives, and will colonize and outcompete natives (Mitsch and Gosselink 2000; Dahl and Stedman 2013). Since the traits of successful invaders tend to increase their resilience to a variety of climate and non-climate disturbances, the combination of these stressors will likely further reduce natives (Rogers & McCarty 2000). Increased temperature and CO_2 concentrations projected due to climate change may boost primary productivity of invasive plants in a salt marsh. *P. australis* is able to establish in areas with significant human disturbance, including development, pollution, and

mechanical disturbance (Meyerson et al. 2000). Similarly, purple loosestrife can survive in many conditions associated with disturbed sites, and can grow in brackish and non-tidal waters, allowing it to outcompete native vegetation and to form dense stands (UMaine 2001;Middleton 2006; NH DES 2019; USGS 2019). While coastal development has led to overall fragmentation for estuarine wetlands, invasivewetlands are not functionally harmed by fragmentation. However, shoreline hardening and other anthropogenic barriers may limit their ability to spread.

Many salt marshes in the region are degraded to some extent, with pristine marshes tending to occur in non-urban areas. Degradation is most prevalent in urban settings, and marshes in these areas are proneto sediment contamination, dredging and filling, encroachment of invasive species (e.g., *P. australis*), and limits to landward retreat due to coastal development and shoreline hardening (Kennish 2001). Native marshes are more susceptible to anthropogenic impacts than marshes dominated by invasive species such as *P. australis* and *L. salicaria.* Invasive species are opportunistic and are more likely to colonize areas of disturbance and outcompete native species. Encroachment by *P. australis* may speed the loss of *S. alterniflora* marshes already under threat from a variety of anthropogenically influenced factors, including increased decomposition and coastal erosion fueled by nutrient loading, salt marsh die-off from herbivory, and sea-level rise (Legault et al. 2018).

Nutrient enrichment in coastal ecosystems can be a driver in native salt marsh loss (Bertness et al. 2002). Nutrient levels associated with coastal eutrophication can increase above-ground leaf biomass but decrease below-ground biomass of bank-stabilizing roots and increase microbial decomposition of organic matter. Further, these alterations reduce geomorphic stability, causing creek-bank collapse and conversion to unvegetated mud. Projected increases in the use of fertilizers and nitrogen fluxes to the coasts may result in larger rates of native salt marsh deterioration and loss (Deegan et al. 2012).

However, nutrient enrichment has been shown to promote non-native *P. australis* establishment, growth, and spreading (Mozdzer and Megonigal 2012). In an experimental mesocosm study both thenative *S. alterniflora* and non-native *P. australis* responded to higher nutrient levels and temperature with increased growth, but the non-native haplotype of *P. australis* outcompeted *S. alterniflora* when additional nutrients were added (Legault et al. 2018).

Literature Cited

Bertness MD, Ewanchuk PJ, Silliman BR. 2002. Anthropogenic modification of New England salt marsh landscapes. Proceedings of the National Academy of Sciences USA 99:1395±1398. https://doi.org/10.1073/pnas.022447299 PMID: 11818525

Bricker-Urso S, Nixon SW, Cochran JK, Hirschberg DJ, Hunt C. 1989. Accretion rates and sediment accumulation in Rhode Island salt marshes. Estuaries 12(4):300-17.

Cahoon DR, Reed DJ, Kolker AS, Brinson MM, Stevenson JC, Riggs S, Christian R, Reyes E,Voss C, Kunz D. 2009. Coastal wetland sustainability. Coastal Sensitivity to Sea-Level Rise: A Focus on the Mid-Atlantic Region. [Titus JG, Anderson KE, Cahoon DR, Gesch DB, Gill SK, Gutierrez BT, Thieler ER,Williams SJ (eds)]. Report by the U.S. Climate Change Science Program and the Subcommittee on Global Change Research. Synthesis and Assessment Product 4.1. 57-72.

Carey JC, Moran SB, Kelly RP, Kolker AS, Fulweiler RW. 2017. The declining role of organic matter in New England salt marshes. Estuaries and Coasts 40(3):626-39.

Charles H, Dukes JS. 2009. Effects of warming and altered precipitation on plant and nutrient dynamics of a New England salt marsh. Ecological Applications 19(7):1758-73.

Crosby SC, Sax DF, Palmer ME, Booth HS, Deegan LA, Bertness MD, Leslie HM. 2016. Salt marsh persistence is threatened by predicted sea-level rise. Estuarine, Coastal and Shelf Science 181:93-9.

Dahl TE, Stedman S-M. 2013. Status and trends of wetlands in the coastal watersheds of the conterminous United States 2004 to 2009. U.S. Department of the Interior, Fish and Wildlife Serviceand National Oceanic and Atmospheric Administration, National Marine Fisheries Service. 1-46.

Deegan LA, Johnson DS, Warren RS, Peterson BJ, Fleeger JW, Fagherazzi S, Wollheim WM. 2012. Coastal eutrophication as a driver of salt marsh loss. Nature 490(7420):388-92.

Dukes JS, and Mooney HA. 1999. Does global change increase the success of biological invaders? Trends in Ecology and Evolution 14(4):135-9.

Gardner LR, Reeves HW. 2002. Spatial patterns in soil water fluxes along a forest-marsh transect in the southeastern United States. Aquatic Sciences 64(2):141-55.

Gedan KB, Bertness MD. 2009. Experimental warming causes rapid loss of plant diversity in New England salt marshes. Ecology Letters 12(8):842-8.

Gedan KB, Bertness MD. 2010. How will warming affect the salt marsh foundation species *Spartina patens* and its ecological role? Oecologia 164(2):479-87.

Kearney MS, Rogers AS, Townshend JRG, Rizzo E, Stutzer D, Stevenson JC, Sundborg K. 2002. Landsat imagery shows decline of coastal marshes in Chesapeake and Delaware Bays. Eos, Transactions American Geophysical Union 83(16):173-8.

Kennish MJ. 2001. Coastal salt marsh systems in the US: a review of anthropogenic impacts. Journal of Coastal Research 17(3):731-48.

Kirwan ML, Blum LK. 2011. Enhanced decomposition offsets enhanced productivity and soil carbon accumulation in coastal wetlands responding to climate change. Biogeosciences 8(4):987-93.

Kirwan ML, Guntenspergen GR, Morris JT. 2009. Latitudinal trends in *Spartina alterniflora* productivityand the response of coastal marshes to global change. Global Change Biology 15(8):1982-9.

Kirwan ML, Mudd SM. 2012. Response of salt-marsh carbon accumulation to climate change. Nature 489(7417):550-3.

Legault R II, Zogg GP, Travis SE. 2018. Competitive interactions between native *Spartina alterniflora* and non-native *Phragmites australis* depend on nutrient loading and temperature. PLOS ONE 13(2):e0192234. https://doi.org/10.1371/journal.pone.0192234

Leonardi N, Ganju NK, Fagherazzi S. 2016. A linear relationship between wave power and erosion determines salt-marsh resilience to violent storms and hurricanes. Proceedings of the NationalAcademy of Sciences 113(1):64-8.

Levermann A, Griesel A, Hofmann M, Montoya M, Rahmstorf S. 2005. Dynamic sea level changes following changes in the thermohaline circulation. Climate Dynamics 24(4):347-54.

Martin RM. 2017. Effects of warming on invasive *Phragmites australis* and native *Spartina patens* seed germination rates and implications for response to climate change. Northeastern Naturalist. 24(3):235-8.

Meyerson LA, Saltonstall K, Windham L, Kiviat E, Findlay S. 2000. A comparison of *Phragmites australis* in freshwater and brackish marsh environments in North America. Wetlands Ecology and Management 8:89-103.

Middleton BA. 2006. Invasive species and climate change: U.S. Geological Survey Open-File Report 2006-1153:2. https://pubs.usgs.gov/of/2006/1153/pdf/of06-1153_508.pdf

Mitsch WJ, Gosselink JG. 2000. Wetlands, 3rd edition. John Wiley and Sons.

Mozdzer TJ, Megonigal JP. 2012. Jack-and-master trait responses to elevated CO_2 and N: a comparison of native and introduced *Phragmites australis*. PLoS ONE 7(10): e42794.doi:10.1371/journal.pone.0042794

Najjar RG, Walker HA, Anderson PJ, Barron EJ, Bord RJ, Gibson JR, Kennedy VS, Knight CG, Megonigal JP, O'Connor RE, Polsky CD, Psuty NP, Richards BA, Sorenson LG, Steele EM, Swanson RS. 2000. The potential impacts of climate change on the mid-Atlantic coastal region. Climate Research 14(3):219-33.

New Hampshire Department of Environmental Services (NHDES). 2019. Purple loosestrife. Environmental fact sheet WD-BB-45. https://www.des.nh.gov/organization/commissioner/pip/factsheets/bb/documents/bb-45.pdf

Nixon SW. 1980. The ecology of New England high salt marshes: a community profile. National Coastal Ecosystems Team, Washington, DC (USA); Rhode Island Univ., Kingston, RI (USA). Graduate School of Oceanography; 1982 Mar 1.

Rejmánek M, Richardson DM. 1996. What attributes make some plant species more invasive? Ecology 77(6):1655-61. https://esajournals.onlinelibrary.wiley.com/doi/epdf/10.2307/2265768

Rogers CE, McCarty JP. 2000. Climate change and ecosystems of the Mid Atlantic Region. Climate Research 14:235-44.

Sallenger AH, Doran KS, Howd PA. 2012. Hotspot of accelerated sea-level rise on the Atlantic coast of North America. Nature Climate Change 2(12):884-8.

Smith JAM. 2013. The role of *Phragmites australis* in mediating inland salt marsh migration in a Mid-Atlantic estuary. PLoS One 8(5):e65091.

University of Maine (UMaine). 2001. Purple loosestrife. Maine invasive plants. Bulletin #2508. https://extension.umaine.edu/publications/2508e/

United States Geological Survey (USGS). 2019. [Internet]. Non-indiginous aquatic species: purple loosestrife (website). https://nas.er.usgs.gov/queries/FactSheet.aspx?SpeciesID=239

Valiela I, Teal JM, Volkmann S, Shafer D, Carpenter EJ. 1978. Nutrient and particulate fluxes in a salt marsh ecosystem: Tidal exchanges and inputs by precipitation and groundwater 1. Limnology and Oceanography 23(4):798-812.

Watson EB, Raposa KB, Carey JC, Wigand C, Warren RS. 2017a. Anthropocene survival of southern New England's salt marshes. Estuaries and Coasts 40(3):617-25.

Watson EB, Wigand C, Davey EW, Andrews HM, Bishop J, Raposa KB. 2017b. Wetland loss patterns and inundation-productivity relationships prognosticate widespread salt marsh loss for southern New England. Estuaries and Coasts 40(3):662-81.

Weston NB. 2014. Declining sediments and rising seas: an unfortunate convergence for tidal wetlands. Estuaries and Coasts 37:1-23.

Estuarine Invasive Wetland: New England

System: Estuarine

Subsystem: Intertidal

Class: Emergent Wetland

Sub-class: Invasive Wetland

Geographic Area: New England

Overall Vulnerability Rank = Moderate

Habitat Sensitivity = Low

Climate Exposure = Very High

	**Estuarine Invasive Wetland: New England**	Attribute Mean	Data Quality	Distribution of Expert Scores
Sensitivity Attributes	Habitat condition	1.8	2.2	
	Habitat fragmentation	1.6	2	
	Distribution/Range	1.7	2.3	
	Mobility/Ability to spread or disperse	1.8	2.1	
	Resistance	1.7	2.3	
	Resilience	1.6	1.8	
	Sensitivity to changes in abiotic factors	1.7	2.1	
	Sensitivity and intensity of non−climate stressors	1.7	2	
	Dependency on critical ecological linkages	1.4	1.5	
	Sensitivity Component Score	Low		
Exposure Factors	Sea surface temp	n/a	n/a	
	Bottom temp	n/a	n/a	
	Air temp	4	3	
	River temp	n/a	n/a	
	Surface salinity	1.6	2.1	
	Bottom salinity	n/a	n/a	
	pH	4	2	
	Sea level rise	3.8	2.2	
	Precipitation	2.7	2.1	
	Floods	n/a	n/a	
	Droughts	n/a	n/a	
	Exposure Component Score	Very High		
	**Overall Vulnerability Rank**	**Moderate**		

Low
Moderate
High
Very High

Habitat Name: Estuarine Invasive Wetland: New England

System**:** Estuarine

Subsystem: Intertidal

Class: Emergent Wetland

Sub-class: Invasive Wetland

Geographic Area: New England

Habitat Description: This sub-class includes intertidal invasive estuarine emergent wetlands or salt marshes in the New England region from the Maine border of Canada south to Rhode Island. Salt marsh communities are characterized by distinct patterns of zonation tied to frequency and depth of tidal inundation, and are dominated by perennial plants (characterized by erect, rooted, herbaceous hydrophytes). Salinity can range from full-strength seawater to brackish (>0.5 ppt). This study considers both persistent and non-persistent wetlands.

Due to expected differences between tidal wetlands in New England and the Mid-Atlantic, the two regions were considered separately in this study. New England salt marshes have higher organic content (peat) and lowersediment inputs than Mid-Atlantic marshes (Nixon 1980; Charles and Dukes 2009). A balance between primary productivity and organic matter decomposition enables New England marshes to maintain elevation (Bricker-Ursoet al. 1989), whereas MA marshes are coastal plain-type marshes with higher sediment input and lowerorganic content (Nixon 1980).

Because ecosystem functions differ between the two types of marshes, New England and Mid-Atlantic marshes are further divided into native and invasive marshes for this study. Invasive species that dominate tidal marshes in both regions include an exotic haplotype of the common reed (*Phragmites australis*), narrow-leaf cattail (*Typha angustifolia*), and purple loosestrife (*Lythrum salicaria*), which are more common in brackish water.

Overall Climate Vulnerability Rank: Moderate. (100 % certainty from bootstrap analysis).

Climate Exposure: Very High. Air Temperature (4.0), pH (4.0) and Sea Level Rise (SLR) (3.8) all contributed to the Very High climate exposure score. Air temperature and SLR are projected to increase in the study area, and pH is projected to decrease (increasing ocean acidification). Sea surface temperatures, which impact emergent wetlands in tandem with air temperature, are increasing at a faster rate in the Gulf of Maine than in the rest of the region, and this trend is projected to continue.

Habitat Sensitivity: Low. All sensitivity scores were <2.0. Anthropogenic impacts such as coastal development and shoreline hardening have contributed to declines in habitat condition and have exacerbated naturally occurring habitat fragmentation. However, marshes dominated by invasive species are typically less sensitive and more resilient to such impacts than are those dominated bynative species.

Data Quality & Gaps: Four of the five exposure data quality scores were Low to Moderate, between 2.0 and 2.2, with the highest score for Air Temperature (3.0). Tidal salt marshes in New England are generally well studied but impacts of climate change to these habitats, as well as differing responses between native and invasive marshes, need further investigation. In addition, data quality for exposure is lower for estuarine and intertidal habitats due to the low resolution of CMIP5 and ROMS-NWA projections for climate exposure in estuaries and nearshore, shallow coastal areas.

Two of the nine sensitivity attribute data quality scores were less than 2.0. Resilience and Dependency on Critical Ecological Linkages scored 1.8 and 1.5, respectively. Distribution/Range and Resistance scored the highest (2.3).

Positive or Negative Climate Effect in the Northeast U.S.: The effect of climate change on invasive tidal emergent wetlands in New England is expected to be primarily positive, with 75% of the experts' scores returned as positive, with 20% neutral and 5% negative.

Climate Effects on Habitat Condition and Distribution: In general, climate change is expected to benefit invasive species and exacerbate their impacts on ecosystems (Duke and Mooney 2005). *P. australis* and other invasive wetland plants are highly adapted to disturbance, and will likely benefit from climate change by outcompeting native salt marsh species (Rejmánek and Richardson 1996; Meyerson et al. 2000; Bertness et al. 2002; Mozdzer and Megoniga 2012; Smith 2013). However, climate change is still expected to have an impact on invasive salt marsh in several ways. Most salt marsh flora are eurythermal, and an increase in temperature may lead to an increase in photosynthetic rates, plant biomass, and other plant physiological processes (Charles and Dukes 2009; Gedan and Bertness 2010; Kirwan and Mudd 2012). *P. australis* exhibited a higher germination rate than a native saltmarsh halophyte, *Spartina patens*, at temperatures projected for the end of the century (Martin 2017). Although both the native *S. alterniflora* and non-native *P. australis* responded to higher experimental temperatures with increased growth, the non-native haplotype of *P. australis* outcompeted *S. alterniflora* when additional nutrients were added (Legault et al. 2018).

Temperature can have other indirect effects on salt marsh wetlands, such as on production of soil organic matter, rates of evaporation and decomposition, and salt marsh community composition (Najjaret al. 2000; Charles and Dukes 2009; Gedan and Bertness 2009; Gedan and Bertness 2010; Carey et al. 2017). The habitat is also sensitive to changes in the marsh platform, as an increase in temperature cancause an increase in decay rate of the organic matter in the platform, offsetting enhanced productivity and soil carbon accumulation associated with increased temperatures (Kirwan and Blum 2011). However, *P. australis* stands accumulate detritus and have slower stem decomposition rates (Meyersonet al. 2000), so subsidence is less of a concern than it is in native marshes.

The precise responses of coastal wetlands to increased warming is difficult to predict, given the complexity of interactions among biological and environmental factors and the coarse level of resolution of landscape-scale models (Cahoon et al. 2009), but studies have found that modest warming can increase halophyte productivity (Charles and Duke 2009; Kirwan et al. 2009). Salt marshes are sensitive to atmospheric CO_2, and could increase productivity as atmospheric CO_2 rises, although there are a number of confounding factors influencing this. Salt marshes are nitrogen-limited and are sensitive to nitrogen inputs (especially anthropogenic eutrophication, see below). Groundwater serves as a nitrogen input to marshes and is affected by precipitation in the watershed (Valiela et al. 1978; Gardner and Reeves 2002).

Sea level rise has increased in the last several decades, which has led to marsh degradation and loss of habitat (Kearney et al. 2002; Crosby et al. 2016; Watson et al. 2017a; Watson et al. 2017b). Increased erosion of the marsh edge could result from the synergistic effects of temperature-mediated decomposition of organic matter in the marsh platform coupled with increased wave height and strength due to sea level rise and increased frequency of storms. Inundation and edge erosion may be exacerbated by a decrease in accretion rate due to loss of sediment input from upstream retention of sediment by dams and decline in agriculture (Weston 2014), or a decline in soil organic matter due to higher decomposition rates fueled by higher water temperature (Carey et al. 2017). Increased edge erosion can result from a greater porosity of the marsh platform from increased decomposition, coupledwith the increase in wave energy. Salt marsh erosion shows a linear response to wave intensity (Leonardi et al. 2016). However, invasive species such as *P. australis* tend to occupy higher-elevation zones in coastal marshes and lower-salinity, low-energy habitats in the upper reaches of estuaries, so would be less sensitive to impacts from marsh-edge erosion and a decrease in accretion rate.

Habitat Summary: Invasive plant species share characteristics that make it easy for them to overtake existing wetland communities (Dukes and Mooney 1999). These include a high rate of population growth, which contributes to rapid colonization; ability to move long distances, which contributes to colonizing distant habitats; tolerance of close association with humans; and tolerance of a broad range of physical conditions (Rejmánek and Richardson 1996). Invasive species typically tolerate disturbed areas better than natives, and will colonize and outcompete natives (Mitsch and Gosselink 2000; Dahl and Stedman 2013). Since the traits of successful invaders tend to increase their resilience to a variety ofclimate and non-climate

disturbances, the combination of these stressors will likely further reduce natives (Rogers & McCarty 2000). Increased temperature and CO_2 concentrations projected due to climate change may boost primary productivity of invasive plants in a salt marsh. *P. australis* is able to establish in areas with significant human disturbance, including development, pollution, and mechanicaldisturbance (Meyerson et al. 2000). Similarly, purple loosestrife can survive in many conditions associated with disturbed sites, and can grow in brackish and non-tidal waters, allowing it to successfully compete with native vegetation and to form dense stands (UMaine 2001; Middleton 2006; NH DES 2019;USGS 2019). While coastal development has led to overall fragmentation for estuarine wetlands, invasive wetlands are not functionally harmed by fragmentation. However, shoreline hardening and other anthropogenic barriers may limit their ability to spread.

Many salt marshes in the region are degraded to some extent, with pristine marshes tending to occur in non-urban areas. Degradation is most prevalent in urban settings, and marshes in these areas are prone to sediment contamination, dredging and filling, encroachment of invasive species (e.g., *P. australis*), and limits to landward retreat due to coastal development and shoreline hardening (Kennish 2001).

Native marshes are more susceptible to anthropogenic impacts than marshes dominated by invasive species such as *P. australis and L. salicaria*. Invasive species are opportunistic and are more likely to colonize areas of disturbance and outcompete native species. Encroachment by *P. australis* may speed the loss of *S. alterniflora* marshes already under threat from a variety of anthropogenically influenced factors, including increased decomposition and coastal erosion fueled by nutrient loading, salt marsh die-off from herbivory, and SLR (Legault et al. 2018).

Nutrient enrichment in coastal ecosystems can be a driver in native salt marsh loss (Bertness et al. 2002). Nutrient levels associated with coastal eutrophication can increase above-ground leaf biomass but decrease below-ground biomass of bank-stabilizing roots and increase microbial decomposition of organic matter. Further, these alterations reduce geomorphic stability, causing creek-bank collapse and conversion to unvegetated mud. Projected increases in the use of fertilizers and nitrogen fluxes to the coasts may result in larger rates of native salt marsh deterioration and loss (Deegan et al. 2012). However, nutrient enrichment has been shown to promote non-native *P. australis* establishment, growth, and spreading (Mozdzer and Megonigal 2012). In an experimental mesocosm study both the native *S. alterniflora* and non-native *P. australis* responded to higher nutrient levels and temperaturewith increased growth, but the non-native haplotype of *P. australis* outcompeted *S. alterniflora* when additional nutrients were added (Legault et al. 2018).

<u>Literature Cited</u>

Bertness MD, Ewanchuk PJ, Silliman BR. 2002. Anthropogenic modification of New England salt marsh landscapes. Proceedings of the National Academy of Sciences USA 99:1395±1398. https://doi.org/10.1073/pnas.022447299 PMID: 11818525

Bricker-Urso S, Nixon SW, Cochran JK, Hirschberg DJ, Hunt C. 1989. Accretion rates and sediment accumulation in Rhode Island salt marshes. Estuaries 12(4):300-17.

Cahoon DR, Reed DJ, Kolker AS, Brinson MM, Stevenson JC, Riggs S, Christian R, Reyes E, Voss C, Kunz D. 2009. Coastal wetland sustainability. Coastal Sensitivity to Sea-Level Rise: A Focus on the Mid-AtlanticRegion. [Titus JG, Anderson KE, Cahoon DR, Gesch DB, Gill SK, Gutierrez BT, Thieler ER, Williams SJ (eds)]. Report by the U.S. Climate Change Science Program and the Subcommittee on Global Change Research. Synthesis and Assessment Product 4.1. 57-72.

Carey JC, Moran SB, Kelly RP, Kolker AS, Fulweiler RW. 2017. The declining role of organic matter in New England salt marshes. Estuaries and Coasts 40(3):626-39.

Charles H, Dukes JS. 2009. Effects of warming and altered precipitation on plant and nutrient dynamics of a New England salt marsh. Ecological Applications 19(7):1758-73.

Crosby SC, Sax DF, Palmer ME, Booth HS, Deegan LA, Bertness MD, Leslie HM. 2016. Salt marsh persistence is threatened by predicted sea-level rise. Estuarine, Coastal and Shelf Science 181:93-9.

Dahl TE, Stedman S-M. 2013. Status and trends of wetlands in the coastal watersheds of the conterminous United States 2004 to 2009. U.S. Department of the Interior, Fish and Wildlife Serviceand National Oceanic and Atmospheric Administration, National Marine Fisheries Service. 1-46.

Deegan LA, Johnson DS, Warren RS, Peterson BJ, Fleeger JW, Fagherazzi S, Wollheim WM. 2012. Coastal eutrophication as a driver of salt marsh loss. Nature 490(7420):388-92.

Dukes JS, and Mooney HA. 1999. Does global change increase the success of biological invaders? Trends in Ecology and Evolution 14(4):135-9.

Gardner LR, Reeves HW. 2002. Spatial patterns in soil water fluxes along a forest-marsh transect in the southeastern United States. Aquatic Sciences 64(2):141-55.

Gedan KB, Bertness MD. 2009. Experimental warming causes rapid loss of plant diversity in New England salt marshes. Ecology Letters 12(8):842-8.

Gedan KB, Bertness MD. 2010. How will warming affect the salt marsh foundation species *Spartina patens* and its ecological role? Oecologia 164(2):479-87.

Kearney MS, Rogers AS, Townshend JRG, Rizzo E, Stutzer D, Stevenson JC, Sundborg K. 2002. Landsat imagery shows decline of coastal marshes in Chesapeake and Delaware Bays. Eos, Transactions American Geophysical Union 83(16):173-8.

Kennish MJ. 2001. Coastal salt marsh systems in the US: a review of anthropogenic impacts. Journal of Coastal Research 17(3):731-48.

Kirwan ML, Blum LK. 2011. Enhanced decomposition offsets enhanced productivity and soil carbon accumulation in coastal wetlands responding to climate change. Biogeosciences 8(4):987-93.

Kirwan ML, Guntenspergen GR, Morris JT. 2009. Latitudinal trends in *Spartina alterniflora* productivityand the response of coastal marshes to global change. Global Change Biology 15(8):1982-9.

Kirwan ML, Mudd SM. 2012. Response of salt-marsh carbon accumulation to climate change. Nature 489(7417):550-3.

Legault R II, Zogg GP, Travis SE. 2018. Competitive interactions between native *Spartina alterniflora* and non-native *Phragmites australis* depend on nutrient loading and temperature. PLOS ONE 13(2): e0192234. https://doi.org/10.1371/journal.pone.0192234

Leonardi N, Ganju NK, Fagherazzi S. 2016. A linear relationship between wave power and erosion determines salt-marsh resilience to violent storms and hurricanes. Proceedings of the National Academy of Sciences 113(1):64-68.

Martin RM. 2017. Effects of warming on invasive *Phragmites australis* and native *Spartina patens* seed germination rates and implications for response to climate change. Northeastern Naturalist 24(3):235-8.

Meyerson LA, Saltonstall K, Windham L, Kiviat E, Findlay S. 2000. A comparison of *Phragmites australis* in freshwater and brackish marsh environments in North America. Wetlands Ecology and Management 8:89-103.

Middleton BA. 2006. Invasive species and climate change: U.S. Geological Survey Open-File Report2006-1153:2. https://pubs.usgs.gov/of/2006/1153/pdf/of06-1153_508.pdf

Mitsch WJ, Gosselink JG . 2000. Wetlands, 3rd edition. John Wiley and Sons.

Mozdzer TJ, Megonigal JP. 2012. Jack-and-master trait responses to elevated CO_2 and N: a comparison of native and introduced *Phragmites australis*. PLoS ONE 7(10):e42794.doi:10.1371/journal.pone.0042794

Najjar RG, Walker HA, Anderson PJ, Barron EJ, Bord RJ, Gibson JR, Kennedy VS, Knight CG, Megonigal JP,O'Connor RE, Polsky CD, Psuty NP, Richards BA, Sorenson LG, Steele EM, Swanson RS. 2000. The potential impacts of climate change on the mid-Atlantic coastal region. Climate Research 14(3):219-33.

Nixon SW. 1980. The ecology of New England high salt marshes: a community profile. National Coastal Ecosystems Team, Washington, DC (USA); Rhode Island Univ., Kingston, RI (USA). Graduate School of Oceanography; 1982 Mar 1.

Rejmánek, M. and Richardson, D.M. 1996. What attributes make some plant species more invasive? Ecology 77(6):1655-61.

Rogers CE, McCarty JP. 2000. Climate change and ecosystems of the Mid Atlantic Region. Climate Research 14:235-44.

Smith JAM. 2013. The role of *Phragmites australis* in mediating inland salt marsh migration in a Mid-Atlantic estuary. PLoS One 8(5):e65091.

University of Maine (UMaine). 2001. Purple loosestrife. Maine invasive plants. Bulletin #2508. https://extension.umaine.edu/publications/2508e/

United States Geological Survey (USGS). 2019. [Internet]. Non-indiginous aquatic species: purple loosestrife (accessed 8 Dec. 2020). https://nas.er.usgs.gov/queries/FactSheet.aspx?SpeciesID=239

Valiela I, Teal JM, Volkmann S, Shafer D, Carpenter EJ. 1978. Nutrient and particulate fluxes in a salt marsh ecosystem: Tidal exchanges and inputs by precipitation and groundwater 1. Limnology and Oceanography 23(4):798-812.

Watson EB, Raposa KB, Carey JC, Wigand C, Warren RS. 2017a. Anthropocene survival of southern New England's salt marshes. Estuaries and Coasts 40(3):617-25.

Watson EB, Wigand C, Davey EW, Andrews HM, Bishop J, Raposa KB. 2017b. Wetland loss patterns and inundation-productivity relationships prognosticate widespread salt marsh loss for southern New England. Estuaries and Coasts 40(3):662-81.

Weston NB. 2014. Declining sediments and rising seas: an unfortunate convergence for tidal wetlands. Estuaries and Coasts 37:1–23.

Estuarine Native Wetland: Mid−Atlantic

System: Estuarine

Subsystem: Intertidal

Class: Emergent Wetland

Sub-class: Native Wetland

Geographic Area: Mid-Atlantic

Overall Vulnerability Rank = Very High

Habitat Sensitivity = High

Climate Exposure = Very High

	**Estuarine Native Wetland: Mid-Atlantic**	Attribute Mean	Data Quality	Distribution of Expert Scores
Sensitivity Attributes	Habitat condition	3.2	2.6	
	Habitat fragmentation	3	2.4	
	Distribution/Range	2	2.5	
	Mobility/Ability to spread or disperse	3	2.1	
	Resistance	3.2	2.5	
	Resilience	3.1	1.8	
	Sensitivity to changes in abiotic factors	2.9	2.3	
	Sensitivity and intensity of non−climate stressors	3.4	2.2	
	Dependency on critical ecological linkages	2.5	1.9	
	Sensitivity Component Score	High		
Exposure Factors	Sea surface temp	n/a	n/a	
	Bottom temp	n/a	n/a	
	Air temp	4	3	
	River temp	n/a	n/a	
	Surface salinity	2.8	2.1	
	Bottom salinity	n/a	n/a	
	pH	4	2	
	Sea level rise	3.9	2.2	
	Precipitation	2.8	2.1	
	Floods	n/a	n/a	
	Droughts	n/a	n/a	
	Exposure Component Score	Very High		
	**Overall Vulnerability Rank**	**Very High**		

Low
Moderate
High
Very High

Habitat Name: Estuarine Native Wetland: Mid-Atlantic

System: Estuarine

Subsystem: Intertidal

Class: Emergent Wetland

Sub-class: Native Wetland

Geographic Area: Mid-Atlantic

Habitat Description: This sub-class includes intertidal native estuarine emergent wetlands and salt marshes occurring in the Mid-Atlantic region from eastern Long Island Sound south to Cape Hatteras, North Carolina. Salt marsh communities are characterized by distinct patterns of zonation tied to frequency and depth of tidal inundation, and are dominated by perennial plants (characterized by erect, rooted, herbaceous hydrophytes). Salinity can range from full-strength seawater to brackish (>0.5ppt). This study considers both persistent and non-persistent wetlands.

Due to expected differences between tidal wetlands in New England and the Mid-Atlantic, the two regions were considered separately in this study. New England salt marshes have higher organic content (peat) and lower sediment inputs than MA marshes (Nixon 1980; Charles and Dukes 2009). A balance between primary productivity and organic matter decomposition enables New England marshes to maintain elevation (Bricker-Ursoet al. 1989), whereas Mid-Atlantic marshes are coastal plain-type marshes with higher sediment input and lower organic content (Nixon 1980).

Because ecosystem functions differ between the two types of marshes, New England and Mid-Atlantic marshes are further divided into native and invasive marshes for this study. Native halophytes that dominate tidal marshes inboth regions include temperate species such as smooth cordgrass (*Spartina alterniflora*), saltmeadow cordgrass (*Spartina patens*), and saltgrass (*Distichlis spicata*). The dominant invasive species includes thecommon reed (*Phragmites australis*), which is more common in oligohaline disturbed areas.

Overall Climate Vulnerability Rank: Very High (100% certainty from bootstrap analysis).

Climate Exposure: Air Temperature (4.0), pH (4.0), and Sea Level Rise (SLR) (3.9) all contributed to the Very High climate exposure score. Air Temperature is projected to increase in the study area, and pH is projected to decrease (increasing ocean acidification). SLR is expected to increase significantly across the Northeast, with the greatest increase predicted for the Mid-Atlantic region. Sea surface temperatures,which impact emergent wetlands in tandem with air temperature, are increasing at a faster rate in the Mid-Atlantic compared to the global mean, and this trend is projected to continue.

Habitat Sensitivity: High. The High score for habitat sensitivity was driven by Habitat Condition (3.2), Habitat Fragmentation (3.0), Resistance (3.2), Sensitivity and Intensity of Non-Climate Stressors (3.4), Mobility/Ability to Spread or Disperse (3.0), and Sensitivity to Changes in Abiotic Factors (2.9). Anthropogenic impacts such as coastal development and shoreline hardening have contributed to declines in habitat condition and have exacerbated naturally occurring habitat fragmentation, especially in the northern Mid-Atlantic.

Data Quality & Gaps: The data quality scores for four of the five exposure factors were between 2.0 and 2.2, and the highest score for Air Temperature (3.0). Tidal salt marshes in the Mid-Atlantic region are well studied but impacts associated with climate change, as well as differing responses between native and invasive marshes, need further investigation. In addition, data quality for exposure is lower for estuarine and intertidal habitats due to the low resolution of CMIP5 and ROMS-NWA projections for climate exposure in estuaries and nearshore, shallow coastal areas.

Data quality scores for two of the nine sensitivity attributes were <2.0. Resilience and Dependency on Critical Ecological Linkages scored 1.8 and 1.9, respectively.

Positive or Negative Climate Effect in the Northeast U.S.: The effect of climate change on native tidal salt

marsh in the Mid-Atlantic is expected to be primarily negative, with 85% of the experts' scores negative and 14% neutral.

Climate Effects on Habitat Condition and Distribution: Effects of anthropogenic impacts exacerbated by climate change will likely result in mostly negative impacts throughout the range, although increased temperature could boost plant primary productivity. Most salt marsh flora are eurythermal, and an increase in temperature may lead to an increase in photosynthetic rates, plant biomass, and other plant physiological processes (Charles and Dukes 2009; Gedan and Bertness 2010; Kirwan and Mudd 2012).

Temperature can also have indirect effects on salt marsh wetlands, such as on production of soil organic matter, rates of evaporation and decomposition, and salt marsh community composition (Najjar et al. 2000; Charles and Dukes 2009; Gedan and Bertness 2009; Gedan and Bertness 2010; Carey et al. 2017). The habitat is also sensitive to changes in the marsh platform, as an increase in temperature can cause an increase in decay rate of the organic matter in the platform, offsetting enhanced productivity and soil carbon accumulation associated with increased temperatures (Kirwan and Blum 2011). Increases in wave energy as sea level rise increases also leads to greater erosion of marsh edge. Increased edge erosion can result from a greater porosity of the marsh platform from increased decomposition, coupled with the increase in wave energy. Salt marsh erosion shows a linear response to wave intensity (Leonardi et al. 2016).

The precise responses of coastal wetlands to increased warming is difficult to predict, given the complexity of interactions among biological and environmental factors and the coarse level of resolution of landscape-scale models (Cahoon et al. 2009). A study in Massachusetts found salt marsh communitiesin southern New England may be resilient to modest warming (Charles and Dukes 2009). Kirwan et al. (2009) reported an increase in productivity of smooth cordgrass throughout its range in North America by about 50-100 g per m^2 per year under a projected warming of 2-4°C. For the Mid-Atlantic and New England regions, this would represent a 10-40% increase in productivity for smooth cordgrass, which approximates the projected marsh losses due to SLR. Salt marshes are sensitive to atmospheric CO_2 (but no evidence of sensitivity to carbonate chemistry), and could increase productivity as atmospheric CO_2 rises, although there are a number of confounding factors influencing this. Salt marshes are nitrogen-limited and are sensitive to nitrogen inputs (especially anthropogenic eutrophication, seebelow). Groundwater serves as a nitrogen input to marshes and is affected by precipitation in the watershed (Valiela et al. 1978; Gardner and Reeves 2002).

The marsh platform edge is susceptible to erosion from wave action. As salt marshes are accretionary habitats and depend on sediment input, the marsh is susceptible if sea level rise exceeds accretion rate. If a salt marsh builds vertically at a slower rate than the sea rises it cannot maintain its elevation relativeto sea level and will become submerged for progressively longer periods during tide cycles, and may die due to waterlogging (Nicholls et al. 1999; Donnelly and Bertness 2001; Kennedy et al. 2002; Cahoon and Guntenspergen 2010).

The rate of SLR is not uniform spatially, and in some regions the rates can be several times higher than the global mean, while in other regions sea level is falling (Bindoff et al. 2007). Yin et al. (2009) found that changes in ocean circulation in the North Atlantic – specifically, a weakening of the Gulf Stream – is playing a role in increasing sea level rise on the U.S. east coast. Sallenger et al. (2012) reported a recent acceleration in sea level rise on 1,000 km of the east coast north of Cape Hatteras, which may be attributed to high-latitude, North Atlantic warming and rising surface water buoyancy. These dynamic sea level changes associated with changing ocean currents and the Atlantic Meridional Overturning Circulation are attributed to an accelerating rate in the Mid-Atlantic region of sea level rise 3–4 times higher than the global average (Levermann et al. 2005; Yin et al. 2009; Sallenger et al. 2012). The annual mean rate of SLR for nearly all locations on the northeast coast is higher than the global average (Eggleston and Pope 2013; DeJong et al. 2015).

Habitat Summary: Approximately 7,360 acres of estuarine salt marsh (0.4 %) was lost in the coastal areaof the U.S. Atlantic coast between 2004 and 2009, predominantly along the Delaware Bay shorelines and primarily attributed to erosion and inundation related to sea level rise. There was also an increase of

approximately 330 acres (<1% increase) in the total area of intertidal wetlands on the US Atlantic Coast due to saltwater incursion in freshwater wetlands (Dahl and Stedman 2013). Salt marsh area is expected to decrease in locations with increased anthropogenic activities, e.g. human encroachment and development (Nicholls et al. 1999; Kennedy et al. 2002; Scavia et al. 2002).

Many salt marshes in the region are degraded to some extent, with pristine marshes tending to occur innon-urban areas. Degradation is most prevalent in urban settings, and marshes in these areas are proneto sediment contamination, dredging and filling, encroachment of invasive species (e.g., *P. australis*), and limits to landward retreat due to coastal development and shoreline hardening (Kennish 2001). Native marshes are more susceptible to anthropogenic impacts than marshes dominated by invasivespecies such as *P. australis*. Northern and southern edges of the range are the least degraded.

Sea level rise has become an issue in the last few decades, which has led to marsh degradation and lossof habitat (Kearney et al. 2002; Crosby et al. 2016; Watson et al. 2017a; Watson et al. 2017b). Inundation and edge erosion may be exacerbated by a decrease in accretion rate due to loss of sedimentinput from upstream retention of sediment by dams and decline in agriculture (Weston 2014), or a decline in soil organic matter due to higher decomposition rates fueled by higher water temperature (Carey et al. 2017).

Anthropogenic impacts such as shoreline hardening and coastal development will prevent landward migration of the marsh as a compensatory mechanism for sea level rise. Titus et al. (2009) estimated that almost 60% of land within 1 m above the high tide line on the U.S. Atlantic coast (Florida to Massachusetts) is developed or expected to be developed and will be unavailable for inland migration ofwetlands.

Nutrient enrichment in coastal ecosystems can be a driver in native salt marsh loss (Bertness et al. 2002). Nutrient levels associated with coastal eutrophication can increase above-ground leaf biomass but decrease below-ground biomass of bank-stabilizing roots and increase microbial decomposition of organic matter. Further, these alterations reduce geomorphic stability, causing creek-bank collapse and conversion to unvegetated mud. Projected increases in the use of fertilizers and nitrogen fluxes to the coasts may result in larger rates of salt marsh deterioration and loss (Deegan et al. 2012). Anthropogenic and climate impacts have exacerbated naturally-occuring fragmentation of Mid-Atlantic salt marshes, with impacts on the overall function of the habitat.

Literature Cited

Bertness MD, Ewanchuk PJ, Silliman BR. 2002. Anthropogenic modification of New England salt marsh landscapes. Proceedings of the National Academy of Sciences USA 99:1395±1398. https://doi.org/10.1073/pnas.022447299 PMID:11818525.

Bindoff N, Willebrand J, Artale V, Cazenave A, M. Gregory J, Gulev S, Hanawa K, Le Quéré C, Levitus S, Nojiri Y, Shum CK, Talley L, Alakkat U. 2007. Observations: oceanic climate change and sea level. Climate change 2007. The physical science basis. Contribution of Working Group I to the Fourth Assessment Report of the Intergovernmental Panel on Climate Change. [Solomon S, Qin D, ManningM, Chen Z, Marquis M, Averyt KB, Tignor M, Miller HL (eds)]. Intergovernmental Panel on Climate Change. 386-432.

Bricker-Urso S, Nixon SW, Cochran JK, Hirschberg DJ, Hunt C. 1989. Accretion rates and sediment accumulation in Rhode Island salt marshes. Estuaries 12(4):300-17.

Cahoon DR, Guntenspergen GR. 2010. Climate change, sea-level rise, and coastal wetlands. National Wetlands Newsletter 32(1):8.

Cahoon DR, Reed DJ, Kolker AS, Brinson MM, Stevenson JC, Riggs S, Christian R, Reyes E, Voss C, Kunz

D.2009. Coastal wetland sustainability. Coastal sensitivity to sea-level rise: a focus on the Mid-Atlantic Region. [Titus JG, Anderson KE, Cahoon DR, Gesch DB, Gill SK, Gutierrez BT, Thieler ER, Williams SJ (eds)]. Report by the U.S. Climate Change Science Program and the Subcommittee on Global Change Research. Synthesis and Assessment Product 4.1. 57-72.

Carey JC, Moran SB, Kelly RP, Kolker AS, Fulweiler RW. 2017. The declining role of organic matter in New England salt marshes. Estuaries and Coasts 40(3):626-39.

Charles H, Dukes JS. 2009. Effects of warming and altered precipitation on plant and nutrient dynamics of a New England salt marsh. Ecological Applications 19(7):1758-73.

Crosby SC, Sax DF, Palmer ME, Booth HS, Deegan LA, Bertness MD, Leslie HM. 2016. Salt marsh persistence is threatened by predicted sea-level rise. Estuarine, Coastal and Shelf Science 181:93-9.

Dahl TE, Stedman S-M. 2013. Status and trends of wetlands in the coastal watersheds of the conterminous United States 2004 to 2009. U.S. Department of the Interior, Fish and Wildlife Serviceand National Oceanic and Atmospheric Administration, National Marine Fisheries Service. 1-46.

Deegan LA, Johnson DS, Warren RS, Peterson BJ, Fleeger JW, Fagherazzi S, Wollheim WM. 2012. Coastal eutrophication as a driver of salt marsh loss. Nature 490(7420):388-92.

DeJong BD, Bierman PR, Newell WL, Rittenour TM, Mahan SA, Balco G, Rood DH. 2015. Pleistocene relative sea levels in the Chesapeake Bay region and their implications for the next century. GSA Today:4-10.

Donnelly JP, Bertness MD. 2001. Rapid shoreward encroachment of salt marsh cordgrass in response to accelerated sea-level rise. Proceedings of the National Academy of Sciences 98(25):14218.

Eggleston J, Pope J. 2013. Land subsidence and relative sea-level rise in the southern Chesapeake Bay region. Reston, Virginia. U.S. Department of the Interior, U.S. Geological Survey. 1-30.

Gardner LR, Reeves HW. 2002. Spatial patterns in soil water fluxes along a forest-marsh transect in the southeastern United States. Aquatic Sciences 64(2):141-55.

Gedan KB, Bertness MD. 2009. Experimental warming causes rapid loss of plant diversity in New England salt marshes. Ecology Letters 12(8):842-8.

Gedan KB, Bertness MD. 2010. How will warming affect the salt marsh foundation species *Spartina patens* and its ecological role? Oecologia 164(2):479-87.

Kearney MS, Rogers AS, Townshend JRG, Rizzo E, Stutzer D, Stevenson JC, Sundborg K. 2002. Landsat imagery shows decline of coastal marshes in Chesapeake and Delaware Bays. Eos, Transactions American Geophysical Union 83(16):173-8.

Kennedy VS, Twilley RR, Kleypas JA, Cowan JHJ, Hare SR. 2002. Coastal and marine ecosystems and global climate change: potential effects on U.S. resources. Pew Center on Global Climate Change.1-51.

Kennish MJ. 2001. Coastal salt marsh systems in the US: a review of anthropogenic impacts. Journal of Coastal Research 17(3):731-48.

Kirwan ML, Blum LK. 2011. Enhanced decomposition offsets enhanced productivity and soil carbon accumulation in coastal wetlands responding to climate change. Biogeosciences 8(4):987-93.

Kirwan ML, Guntenspergen GR, Morris JT. 2009. Latitudinal trends in *Spartina alterniflora* productivity and the response of coastal marshes to global change. Global Change Biology 15(8):1982-9.

Kirwan ML, Mudd SM. 2012. Response of salt-marsh carbon accumulation to climate change. Nature 489(7417):550-3.

Leonardi N, Ganju NK, Fagherazzi S. 2016. A linear relationship between wave power and erosion determines salt-marsh resilience to violent storms and hurricanes. Proceedings of the National Academy of Sciences 113(1):64-8.

Levermann A, Griesel A, Hofmann M, Montoya M, Rahmstorf S. 2005. Dynamic sea level changes following changes in the thermohaline circulation. Climate Dynamics 24(4):347-54.

Najjar RG, Walker HA, Anderson PJ, Barron EJ, Bord RJ, Gibson JR, Kennedy VS, Knight CG, Megonigal JP, O'Connor RE, Polsky CD, Psuty NP, Richards BA, Sorenson LG, Steele EM, Swanson RS. 2000. The potential impacts of climate change on the mid-Atlantic coastal region. Climate Research 14(3):219-33.

Nicholls RJ, Hoozemans FMJ, Marchand M. 1999. Increasing flood risk and wetland losses due to global sea-level rise: regional and global analyses. Global Environmental Change 9:S69-S87.

Nixon SW. 1980. The ecology of New England high salt marshes: a community profile. National Coastal Ecosystems Team, Washington, DC (USA); Rhode Island Univ., Kingston, RI (USA). Graduate School of Oceanography; 1982 Mar 1.

Sallenger AH, Doran KS, Howd PA. 2012. Hotspot of accelerated sea-level rise on the Atlantic coast ofNorth America. Nature Climate Change 2(12):884-8.

Scavia D, Field JC, Boesch DF, Buddemeier RW, Burkett V, Cayan DR, Fogarty M, Harwell MA, Howarth RW, Mason C, Reed DJ, Royer TC, Sallenger AH, Titus JG. 2002. Climate change impacts on US coastal and marine ecosystems. Estuaries 25(2):149-64.

Titus JG, Hudgens DE, Trescott DL, Craghan M, Nuckols WH, Hershner CH, Kassakian JM, Linn CJ, Merritt PG, McCue TM, O'Connell JF, Tanski J, Wang J. 2009. State and local governments plan for development of most land vulnerable to rising sea level along the US Atlantic coast. Environmental Research Letters 4(4):1-7.

Valiela I, Teal JM, Volkmann S, Shafer D, Carpenter EJ. 1978. Nutrient and particulate fluxes in a salt marsh ecosystem: Tidal exchanges and inputs by precipitation and groundwater 1. Limnology and Oceanography 23(4):798-812.

Watson EB, Raposa KB, Carey JC, Wigand C, Warren RS. 2017a. Anthropocene survival of southern New England's salt marshes. Estuaries and Coasts 40(3):617-25.

Watson EB, Wigand C, Davey EW, Andrews HM, Bishop J, Raposa KB. 2017b. Wetland loss patterns and inundation-productivity relationships prognosticate widespread salt marsh loss for southern New England. Estuaries and Coasts 40(3):662-81.

Weston NB. 2014. Declining sediments and rising seas: an unfortunate convergence for tidal wetlands. Estuaries and Coasts 37:1–23.

Yin J, Schlesinger ME, Stouffer RJ. 2009. Model projections of rapid sea-level rise on the northeast coast of the United States. Nature Geoscience 2(4):262-6.

Estuarine Native Wetland: New England

System: Estuarine

Subsystem: Intertidal

Class: Emergent Wetland

Sub-class: Native Wetland

Geographic Area: New England

Overall Vulnerability Rank = Very High

Habitat Sensitivity = High

Climate Exposure = Very High

	**Estuarine Native Wetland: New England**	Attribute Mean	Data Quality	Distribution of Expert Scores
Sensitivity Attributes	Habitat condition	3.2	2.6	
	Habitat fragmentation	3.1	2.4	
	Distribution/Range	2.2	2.5	
	Mobility/Ability to spread or disperse	2.8	2.1	
	Resistance	3.1	2.5	
	Resilience	2.9	2	
	Sensitivity to changes in abiotic factors	3	2.3	
	Sensitivity and intensity of non−climate stressors	3.5	2.2	
	Dependency on critical ecological linkages	2.5	1.7	
	Sensitivity Component Score	High		
Exposure Factors	Sea surface temp	n/a	n/a	
	Bottom temp	n/a	n/a	
	Air temp	4	3	
	River temp	n/a	n/a	
	Surface salinity	1.6	2.1	
	Bottom salinity	n/a	n/a	
	pH	4	2	
	Sea level rise	3.9	2.2	
	Precipitation	2.7	2.1	
	Floods	n/a	n/a	
	Droughts	n/a	n/a	
	Exposure Component Score	Very High		
**Overall Vulnerability Rank**		**Very High**		

Low
Moderate
High
Very High

Habitat Name: Estuarine Native Wetland: New England

System**:** Estuarine

Subsystem: Intertidal

Class: Emergent Wetland

Sub-class: Native Wetland

Geographic Area: New England

Habitat Description: This sub-class includes intertidal native estuarine emergent wetlands and salt marshes occurring in the New England region from the Maine border of Canada south to Rhode Island. Salt marsh communities are characterized by distinct patterns of zonation tied to frequency anddepth of tidal inundation, and are dominated by perennial plants (characterized by erect, rooted, herbaceous hydrophytes). Salinity can range from full-strength seawater to brackish (>0.5 ppt). This study considers both persistent and non-persistent wetlands.

Due to expected differences between tidal wetlands in New England and the Mid-Atlantic, the two regions were considered separately in this study. New England salt marshes have higher organic content (peat) and lower sediment inputs than MA marshes (Nixon 1980; Charles and Dukes 2009). A balance between primary productivity and organic matter decomposition enables New England marshes to maintain elevation (Bricker-Ursoet al. 1989), whereas Mid-Atlantic marshes are coastal plain-type marshes with higher sediment input and lower organic content (Nixon 1980).

Because ecosystem functions differ between the two types of marshes, New England and Mid-Atlantic marshes are further divided into native and invasive marsh plants for this study. Native halophytes that dominate tidal marshes in both regions include temperate species such as smooth cordgrass (*Spartina alterniflora*), saltmeadow cordgrass (*Spartina patens*), and saltgrass (*Distichlis spicata*). The dominant invasive species includes the invasive common reed (*Phragmites australis*), which is more common in oligohaline disturbed areas.

Overall Climate Vulnerability Rank: Very High. (100 % certainty from bootstrap analysis).

Climate Exposure: Very High. Air Temperature (4.0), pH (4.0), and Sea Level Rise (SLR) (3.9) all contributed to the Very High climate exposure score. Air temperature and SLR are projected to increase in the study area, and pH is projected to decrease (increasing ocean acidification). Sea surface temperatures, which impact emergent wetlands in tandem with air temperature, are increasing at a faster rate in the Gulf of Maine than in the rest of the region, and this trend is projected to continue.

Habitat Sensitivity: High. The High score for habitat sensitivity was driven by Habitat Condition (3.2), Habitat Fragmentation (3.1), Resistance (3.1), and Sensitivity and Intensity of Non-Climate Stressors (3.5). Anthropogenic impacts such as coastal development and shoreline hardening have contributed to declines in habitat condition and have exacerbated naturally occurring habitat fragmentation, especially in southern New England. Marsh platforms degraded by increased decomposition due to warming temperatures are at greater risk of inundation and edge erosion from sea level rise.

Data Quality & Gaps: Four of the five exposure factors scored Low to Moderate, between 2.0 and 2.2, with the highest score for Air Temperature (3.0). Tidal salt marshes in New England are well studied but impacts associated with climate change to these habitats, as well as differing responses between native and invasive marshes, need further investigation. In addition, data quality for exposure is lower for estuarine and intertidal habitats due to the low resolution of CMIP5 and ROMS-NWA projections for climate exposure in estuaries and nearshore, shallow coastal areas.

Eight of the nine sensitivity attribute data quality scores were greater than 2.0, with Dependency on Critical Ecological Linkages scoring 1.7. Habitat Condition, Distribution/Range, and Resistance were Moderate with scores of 2.5 and 2.6.

Positive or Negative Climate Effect in the Northeast U.S.: The effect of climate change on native tidal salt marsh in New England is expected to be primarily negative, with 75% of the experts' scores returned as negative (20% were neutral and 5% positive).

Climate Effects on Habitat Condition and Distribution: Effects of anthropogenic impacts exacerbated by climate change will likely result in mostly negative impacts throughout the range, although increased temperature could boost primary productivity. Most salt marsh flora are eurythermal, and an increase in temperature can lead to an increase in photosynthetic rates, plant biomass, and other plant physiological processes (Charles and Dukes 2009; Gedan and Bertness 2010; Kirwan and Mudd 2012).

Temperature can also have indirect effects on salt marsh wetlands, such as on production of soil organic matter, rates of evaporation and decomposition, and salt marsh community composition (Najjar et al. 2000; Charles and Dukes 2009; Gedan and Bertness 2009; Gedan and Bertness 2010; Carey et al. 2017). The habitat is also sensitive to changes in the marsh platform, as an increase in temperature can cause an increase in decay rate of the organic matter in the platform, offsetting enhanced productivity and soil carbon accumulation associated with increased temperatures (Kirwan and Blum 2011). Increases in wave energy as sea level rise increases also leads to greater erosion of marsh edge. Increased edge erosion can result from a greater porosity of the marsh platform from increased decomposition, coupled with the increase in wave energy. Salt marsh erosion shows a linear response to wave intensity (Leonardi et al. 2016).

Lower accretion rates for salt marsh wetlands in Narragansett Bay, RI, over the past 30 years are correlated with a decline in soil organic matter, which is attributed to higher decomposition rates fueled by higher water temperature (Carey et al. 2017). The precise responses of coastal wetlands to increased warming is difficult to predict, given the complexity of interactions among biological and environmental factors and the coarse level of resolution of landscape-scale models (Cahoon et al. 2009). In a temperature manipulation experiment on tidal salt marsh communities in Massachusetts, Charles and Dukes (2009) found that modest warming increased the above-ground biomass of smooth cordgrass (*Spartina alterniflora*) dominant community, but not the saltmeadow cordgrass-saltgrass (*Spartina patens-Distichlis spicata*) community. Warming also increased the maximum stem heights of the three species. The results of this study suggest salt marsh communities in southern New England may be resilient to modest warming. Kirwan et al. (2009) reported an increase in productivity of smooth cordgrass throughout its range in North America by about 50-100 g per m^2 per year under a projected warming of 2-4°C. For the Mid-Atlantic and New England regions, this would represent a 10-40% increase in productivity for smooth cordgrass, which approximates the projected marsh losses due to sea level rise. Salt marshes are sensitive to atmospheric CO_2 (but no evidence of sensitivity to carbonate chemistry), and could increaseproductivity as atmospheric CO_2 rises, although there are a number of confounding factors influencing this. Salt marshes are nitrogen-limited and are sensitive to nitrogen inputs (especially anthropogenic eutrophication, see below). Groundwater serves as a nitrogen input to marshes and is affected by precipitation in the watershed (Valiela et al. 1978; Gardner and Reeves 2002).

The marsh platform edge is susceptible to erosion from wave action. As salt marshes are accretionary habitats and depend on sediment input, the marsh is susceptible if sea level rise exceeds accretion rate. If a salt marsh builds vertically at a slower rate than the sea rises it cannot maintain its elevation relativeto sea level and will become submerged for progressively longer periods during tide cycles, and may die due to waterlogging (Nicholls et al. 1999; Donnelly and Bertness 2001; Kennedy et al. 2002; Cahoon and Guntenspergen 2010).

The rate of SLR is not uniform spatially, and in some regions the rates can be several times higher than the global mean (Bindoff et al. 2007). Yin et al. (2009) found that changes in ocean circulation in the North Atlantic – specifically, a weakening of the Gulf Stream – is playing a role in increasing SLR on the U.S. east coast. Sallenger et al. (2012) reported a recent acceleration in SLR on 1,000 km of the east coast north of Cape Hatteras, which may be attributed to high-latitude, North Atlantic warming and rising surface water buoyancy. The annual mean rate of SLR for nearly all locations on the northeast coast is higher than the global average (Eggleston and Pope 2013;DeJong et al. 2015).

<u>Habitat Summary</u>: New England has seen a loss of 37% of its salt marshes since the 1800s (Bromberg and Bertness 2005), primarily due to coastal development. Approximately 7,360 acres of estuarine salt marsh (-0.4 %) was lost in the coastal area of the entire U.S. Atlantic coast between 2004 and 2009, although predominantly along the Delaware Bay shorelines and primarily attributed to erosion and inundation related to sea level rise. There was also an increase of approximately 330 acres (<1% increase) in the total area of intertidal wetlands on the US Atlantic Coast due to saltwater incursion in freshwater wetlands (Dahl and Stedman 2013). Salt marsh area is expected to decrease in locations with increased anthropogenic activities, e.g. human encroachment and development (Nicholls et al. 1999; Kennedy et al. 2002; Scavia et al. 2002).

Many salt marshes in New England are degraded to some extent, with pristine marshes tending to occur in non-urban areas. Degradation is most prevalent in urban settings, and marshes in these areas are prone to sediment contamination, dredging and filling, encroachment of invasive species (e.g. *P. australis*), and limits to landward retreat due to coastal development and shoreline hardening (Kennish 2001). Native marshes are more susceptible to anthropogenic impacts than marshes dominated by invasive species such as *P. australis*. Native marshes in Cape Cod are degraded due to a native crab (*Sesarma reticulatum*) released from predation pressure by anthropogenic impacts (Holdredge et al. 2009; Coverdale et al. 2012), although the invasive green crab (*Carcinus meanas*) may be ameliorating that impact (Coverdale et al. 2013). Northern edges of the range are the least degraded from coastal development, although some evidence suggests that green crabs in Maine salt marshes are destabilizingthe marsh platform, increasing erosion risk (Aman and Grimes 2016).

Sea level rise has become an issue in the last few decades, which has led to marsh degradation and lossof habitat (Kearney et al. 2002; Crosby et al. 2016; Watson et al. 2017a; Watson et al. 2017b). Inundation and edge erosion may be exacerbated by a decrease in accretion rate due to loss of sediment input from upstream retention of sediment by dams and decline in agriculture (Weston 2014), or a decline in soil organic matter due to higher decomposition rates fueled by higher water temperature (Carey et al. 2017).

Anthropogenic impacts such as shoreline hardening and coastal development will prevent landward migration of the marsh as a compensatory mechanism for sea level rise. Titus et al. (2009) estimated that almost 60% of land within 1 m above the high tide line on the U.S. Atlantic coast (Florida to Massachusetts) is developed or expected to be developed and will be unavailable for inland migration of wetlands.

Nutrient enrichment in coastal ecosystems can be a driver in native salt marsh loss (Bertness et al. 2002). Nutrient levels associated with coastal eutrophication can increase above-ground leaf biomass but decrease below-ground biomass of bank-stabilizing roots and increase microbial decomposition of organic matter. Further, these alterations reduce geomorphic stability, causing creek-bank collapse and conversion to unvegetated mud. Projected increases in the use of fertilizers and nitrogen fluxes to the coasts may result in larger rates of salt marsh deterioration and loss (Deegan et al. 2012). Anthropogenic and climate impacts have exacerbated naturally-occuring fragmentation of New England salt marshes, with impacts on the overall function of the habitat.

<u>Literature Cited</u>

Aman J, Grimes KW. 2016. Measuring impacts of invasive European green crabs on Maine salt marshes: a novel approach. Report to the Maine Outdoor Heritage Fund; Wells National Estuarine Research Reserve: Wells, ME, USA. 2016 Feb 29:19.

Bertness MD, Ewanchuk PJ, Silliman BR. 2002. Anthropogenic modification of New England salt marsh landscapes. Proceedings of the National Academy of Sciences USA 99:1395-8. https://doi.org/10.1073/pnas.022447299 PMID:11818525

Bindoff N, Willebrand J, Artale V, Cazenave A, M. Gregory J, Gulev S, Hanawa K, Le Quéré C, Levitus S, Nojiri Y, Shum CK, Talley L, Alakkat U. 2007. Observations: oceanic climate change and sea level.

Climate change 2007. The physical science basis. Contribution of Working Group I to the Fourth Assessment Report of the Intergovernmental Panel on Climate Change. [Solomon S, Qin D, Manning M, Chen Z, Marquis M, Averyt KB, Tignor M, Miller HL (eds)]. Intergovernmental Panel on Climate Change. 386-432.

Bromberg KD, Bertness MD. 2005. Reconstructing New England salt marsh losses using historical maps. Estuaries 28(6):823-32.

Bricker-Urso S, Nixon SW, Cochran JK, Hirschberg DJ, Hunt C. 1989. Accretion rates and sediment accumulation in Rhode Island salt marshes. Estuaries 12(4):300-17.

Cahoon DR, Guntenspergen GR. 2010. Climate change, sea-level rise, and coastal wetlands. National Wetlands Newsletter 32(1):8.

Cahoon DR, Reed DJ, Kolker AS, Brinson MM, Stevenson JC, Riggs S, Christian R, Reyes E, Voss C, Kunz D.2009. Coastal wetland sustainability. Coastal sensitivity to sea-level rise: a focus on the Mid-Atlantic Region. [Titus JG, Anderson KE, Cahoon DR, Gesch DB, Gill SK, Gutierrez BT, Thieler ER, Williams SJ (eds)]. Report by the U.S. Climate Change Science Program and the Subcommittee on Global Change Research. Synthesis and Assessment Product 4.1. 57-72.

Carey JC, Moran SB, Kelly RP, Kolker AS, Fulweiler RW. 2017. The declining role of organic matter in New England salt marshes. Estuaries and Coasts 40(3):626-39.

Charles H, Dukes JS. 2009. Effects of warming and altered precipitation on plant and nutrient dynamics of a New England salt marsh. Ecological Applications 19(7):1758-73.

Coverdale TC, Altieri AH, Bertness MD. 2012 Belowground herbivory increases vulnerability of New England salt marshes to die-off. Ecology 93(9):2085-94.

Coverdale TC, Axelman EE, Brisson CP, Young EW, Altieri AH, Bertness MD. 2013. New England salt marsh recovery: opportunistic colonization of an invasive species and its non-consumptive effects. PLoS One8(8):e73823.

Crosby SC, Sax DF, Palmer ME, Booth HS, Deegan LA, Bertness MD, Leslie HM. 2016. Salt marsh persistence is threatened by predicted sea-level rise. Estuarine, Coastal and Shelf Science 181:93-9.

Dahl TE, Stedman S-M. 2013. Status and trends of wetlands in the coastal watersheds of the conterminous United States 2004 to 2009. U.S. Department of the Interior, Fish and Wildlife Service and National Oceanic and Atmospheric Administration, National Marine Fisheries Service. 1-46.

Deegan LA, Johnson DS, Warren RS, Peterson BJ, Fleeger JW, Fagherazzi S, Wollheim WM. 2012. Coastal eutrophication as a driver of salt marsh loss. Nature 490(7420):388-92.

DeJong BD, Bierman PR, Newell WL, Rittenour TM, Mahan SA, Balco G, Rood DH. 2015. Pleistocene relative sea levels in the Chesapeake Bay region and their implications for the next century. GSA Today:4-10.

Donnelly JP, Bertness MD. 2001. Rapid shoreward encroachment of salt marsh cordgrass in response to accelerated sea-level rise. Proceedings of the National Academy of Sciences 98(25):14218.

Eggleston J, Pope J. 2013. Land subsidence and relative sea-level rise in the southern Chesapeake Bay region. Reston, Virginia:: U.S. Department of the Interior, U.S. Geological Survey. 1-30.

Gardner LR, Reeves HW. 2002. Spatial patterns in soil water fluxes along a forest-marsh transect in the southeastern United States. Aquatic Sciences 64(2):141-55.

Gedan KB, Bertness MD. 2009. Experimental warming causes rapid loss of plant diversity in New England salt marshes. Ecology Letters 12(8):842-8.

Gedan KB, Bertness MD. 2010. How will warming affect the salt marsh foundation species *Spartina patens* and its ecological role? Oecologia 164(2):479-87.

Holdredge C, Bertness MD, Altieri AH. 2009 Role of crab herbivory in die-off of New England salt marshes. Conservation Biology 23(3):672-9.

Kearney MS, Rogers AS, Townshend JRG, Rizzo E, Stutzer D, Stevenson JC, Sundborg K. 2002. Landsat imagery shows decline of coastal marshes in Chesapeake and Delaware Bays. Eos, Transactions American Geophysical Union 83(16):173-8.

Kennedy VS, Twilley RR, Kleypas JA, Cowan JHJ, Hare SR. 2002. Coastal and marine ecosystems and global climate change: potential effects on U.S. resources. Pew Center on Global Climate Change.1-51.

Kennish MJ. 2001. Coastal salt marsh systems in the US: a review of anthropogenic impacts. Journal of Coastal Research 17(3):731-48.

Kirwan ML, Blum LK. 2011. Enhanced decomposition offsets enhanced productivity and soil carbon accumulation in coastal wetlands responding to climate change. Biogeosciences 8(4):987-93.

Kirwan ML, Guntenspergen GR, Morris JT. 2009. Latitudinal trends in *Spartina alterniflora* productivity and the response of coastal marshes to global change. Global Change Biology 15(8):1982-9.

Kirwan ML, Mudd SM. 2012. Response of salt-marsh carbon accumulation to climate change. Nature 89(7417):550-3.

Leonardi, N, Ganju, NK and Fagherazzi, S. 2016. A linear relationship between wave power and erosion determines salt-marsh resilience to violent storms and hurricanes. Proceedings of the National Academy of Sciences 113(1):64-8.

Najjar RG, Walker HA, Anderson PJ, Barron EJ, Bord RJ, Gibson JR, Kennedy VS, Knight CG, Megonigal JP,O'Connor RE, Polsky CD, Psuty NP, Richards BA, Sorenson LG, Steele EM, Swanson RS. 2000. The potential impacts of climate change on the mid-Atlantic coastal region. Climate Research 14(3):219-33.

Nicholls RJ, Hoozemans FMJ, Marchand M. 1999. Increasing flood risk and wetland losses due to global sea-level rise: regional and global analyses. Global Environmental Change 9:S69-S87.

Nixon SW. 1980. The ecology of New England high salt marshes: a community profile. National Coastal Ecosystems Team, Washington, DC (USA); Rhode Island Univ., Kingston, RI (USA). Graduate School of Oceanography; 1982 Mar 1.

Sallenger AH, Doran KS, Howd PA. 2012. Hotspot of accelerated sea-level rise on the Atlantic coast ofNorth America. Nature Climate Change 2(12):884-8.

Scavia D, Field JC, Boesch DF, Buddemeier RW, Burkett V, Cayan DR, Fogarty M, Harwell MA, Howarth RW, Mason C, Reed DJ, Royer TC, Sallenger AH, Titus JG. 2002. Climate change impacts on US coastal and marine ecosystems. Estuaries 25(2):149-64.

Titus JG, Hudgens DE, Trescott DL, Craghan M, Nuckols WH, Hershner CH, Kassakian JM, Linn CJ, Merritt PG, McCue TM, O'Connell JF, Tanski J, Wang J. 2009. State and local governments plan for development of most land vulnerable to rising sea level along the US Atlantic coast. Environmental Research Letters 4(4):1-7.

Valiela, I, Teal, JM, Volkmann, S, Shafer, D and Carpenter, EJ. 1978. Nutrient and particulate fluxes in a salt marsh ecosystem: Tidal exchanges and inputs by precipitation and groundwater 1. Limnology and Oceanography 23(4):798-812.

Watson EB, Raposa KB, Carey JC, Wigand C, Warren RS. 2017a. Anthropocene survival of southern New England's salt marshes. Estuaries and Coasts 40(3):617-25.

Watson EB, Wigand C, Davey EW, Andrews HM, Bishop J, Raposa KB. 2017b. Wetland loss patterns and inundation-productivity relationships prognosticate widespread salt marsh loss for southern New England. Estuaries and Coasts 40(3):662-81.

Weston NB. 2014. Declining sediments and rising seas: an unfortunate convergence for tidal wetlands. Estuaries and Coasts 37:1-23.

Yin J, Schlesinger ME, Stouffer RJ. 2009. Model projections of rapid sea-level rise on the northeast coastof the United States. Nature Geoscience 2(4):262-6.

Estuarine Intertidal Shellfish Reef

System: Estuarine

Subsystem: Intertidal

Class: Reef

Sub-class: Mollusk

Geographic Area: Entire Area

Overall Vulnerability Rank = Very High

Habitat Sensitivity = High

Climate Exposure = Very High

	Estuarine Intertidal Shellfish Reef	Attribute Mean	Data Quality	Distribution of Expert Scores
Sensitivity Attributes	Habitat condition	3.1	2.2	
	Habitat fragmentation	3	1.4	
	Distribution/Range	2.7	1.8	
	Mobility/Ability to spread or disperse	2.6	1.3	
	Resistance	2.6	1.8	
	Resilience	2.9	2	
	Sensitivity to changes in abiotic factors	3.1	2.3	
	Sensitivity and intensity of non−climate stressors	3.5	2	
	Dependency on critical ecological linkages	2.8	1.4	
	Sensitivity Component Score	High		
Exposure Factors	Sea surface temp	n/a	n/a	
	Bottom temp	n/a	n/a	
	Air temp	4	3	
	River temp	n/a	n/a	
	Surface salinity	2.7	2.1	
	Bottom salinity	n/a	n/a	
	pH	4	2	
	Sea level rise	3.9	2.2	
	Precipitation	2.8	2.1	
	Floods	n/a	n/a	
	Droughts	n/a	n/a	
	Exposure Component Score	Very High		
**Overall Vulnerability Rank**		**Very High**		

Low
Moderate
High
Very High

Habitat Name: Estuarine Intertidal Shellfish Reef

System: Estuarine

Subsystem: Intertidal

Class: Reef

Sub-class: Mollusk

Geographic Area: Entire Area

Habitat Description: This sub-class includes intertidal, reef-building shellfish that create a biotic hard substrate on benthic substrates in estuarine waters between 0.5 and 30 ppt. The most common species in the study area are blue mussels (*Mytilus edulis*) and eastern oyster (*Crassostrea virginica*). Oysters and mussels grow in dense aggregations forming reefs that extend off the seafloor. Oysters range from the Gulf of St. Lawrence, Canada to the Yucatan, West Indies and Brazil (Gunter 1951). Suitable substrates consist of sand, firm mud, or clay, whereas shifting sand and extremely soft mud are thought to be the only unsuitable substrates for oyster reef habitats (Galstoff 1964; Bahr and Lanier 1981). The blue mussel is a cosmopolitan species common to temperate and polar waters in the northern Atlantic Ocean from the southern Canadian Maritime provinces to North Carolina.

Shellfish reefs serve as habitat for diverse assemblages of polychaetes, crustaceans, and other resident invertebrate and fish species (Wells 1961; Bahr and Lanier 1981; Rothschild et al 1994; Coen et al. 1999; Peterson et al. 2003; zu Ermgassen et al. 2016). They are filter feeders, thereby promoting greater water clarity and benthic productivity (Dame et a. 1984; Newell 1988; Paerl et al. 1998; Ulanowicz and Tuttle 1992). Bivalve reefs also remove excess nitrogen from coastal estuaries by promoting bacterially mediated denitrification as a consequence of concentrating bottom deposits of feces and pseudofeces (Newell et al. 2002; Piehler and Smyth 2011).

Overall Climate Vulnerability Rank: Very High (99% certainty from bootstrap analysis).

Climate Exposure: Very High. The overall Very High exposure score was influenced by three Very High attribute means: Air Temperature (4.0), pH (4.0), and Sea Level Rise (SLR) (3.9). The exposure attribute score for Precipitation (2.8) and Surface Salinity (2.7) were also High. SLR is expected to impact the entire study area but the greatest relative rise is projected in the Mid-Atlantic. The intertidal nature of this habitat places it at the nexus of significant atmospheric and oceanic change.

Habitat Sensitivity: High. Six of the nine sensitivity attribute means were ≥2.8: Habitat Condition (3.1), Habitat Fragmentation (3), Resilience (2.9), Sensitivity to Changes in Abiotic Factors (3.1), Sensitivity and Intensity of Non-Climate Stressors (3.5), and Dependency on Critical Ecological Linkages (2.8). Meanwhile, the attribute means for Mobility/Ability to Spread or Disperse (2.6), Resistance (2.6) and Distribution/Range (2.7) were moderate.

Data Quality & Gaps: Data quality for the four climate exposure factors were mostly ranked as Low to Moderate: Surface Salinity (2.1), SLR (2.2), pH (2) and Precipitation (2.1), while the highest (Air Temperature) was 3.0. The relatively Low score for Precipitation may be due to uncertainty in the specific spatial nature of projected changes in extreme events. In addition, data quality is lower for estuarine habitats due to the low resolution of CMIP5 and ROMS-NWA projections for climate exposure in estuaries and nearshore, shallow coastal areas. Another uncertainty factor is the relative disparate spatial data for mollusk reefs in some portions of the study area.

For habitat sensitivity attributes, data quality for all of the nine attributes were scored between 1.3 and 2.3, with Habitat Fragmentation (1.4), Distribution/Range (1.8), Mobility/Ability to Spread or Disperse (1.3), Resistance (1.8) and Dependency on Critical Ecological Linkages (1.4) all <2.0. Although the climate sensitivity for estuarine subtidal mollusk reefs are believed to be high, there remains some uncertainty in species' capacity to adapt and respond to climate change (e.g., synergistic effects of climate and non-climate stressors).

Relatively moderate to low data quality scores could reflect the fact that few comprehensive assessments for shellfish habitat exist in the study area as much of it was lost centuries ago (Kirby 2004; Rothschild et al. 1994; zu Ermgassen et al. 2012).

Positive or Negative Climate Effect for the Northeast U.S.: The effect of climate change on estuarine intertidal shellfish habitats in the Northeast U.S. is expected to be mostly negative (75% of the experts' scores were negative, 20% neutral, and only 5% positive).

Climate Effects on Habitat Condition and Distribution: Several studies have examined the effects of climate and non-climate impacts on shellfish reefs. While overharvesting has historically been attributed to oyster reef habitat loss in the northeast and elsewhere, hypoxia, disease, predators, competition, and sedimentation have impeded recent restoration efforts (Rothschild et al. 1994; Kirby 2004; Beck et al. 2011; zu Ermgassen et al. 2012). In addition, there is growing evidence that suggests potential climate effects on shellfish reefs may also be a factor.

Increasing seawater temperatures may affect the condition and distribution of intertidal shellfish in estuarine waters. For example, southern populations of blue mussels appear to be shifting northward in response to warming temperatures (Jones et al. 2010). Thermal stress, in conjunction with ocean acidification conditions, was found to cause metabolic depression in blue mussels from the Gulf of Maine (Lesser 2016), suggesting that elevated temperatures may result in suboptimal conditions for this cold-adapted species. Oyster growth and reproductive rates peak in waters ranging in temperature from 20-30°C and can live in water temperatures of 0-36°C (Shumway 1996; Lenihan 1999). In comparison, mussels are common in colder, more temperate to polar waters, thriving in 5-20°C, with an upper thermal tolerance limit of 29°C (Animal Diversity Web 2020).

Although the ROMS-NWA projections limit the ability to make precise climate predictions on impacts to estuarine intertidal shellfish reefs, water and air temperatures are projected to increase compared to historic means. The ROMS-NWA projections for RCP8.5 indicate the standardized anomaly for sea surface temperature will increase by at least 4 standard deviations from the historic means, and higher in the Gulf of Maine. In addition, CMIP5 projections indicate standardized anomaly for air temperature will increase by 6 standard deviations from the historic means. Therefore, there is a possibility the maximum temperature thresholds for one or more life stages for shellfish may be exceeded by the end of the century, especially during episodic heat waves (Jones et al. 2009; Jones et al. 2010; Zippay and Helmuth 2012; Speights et al. 2017). Warming air and seawater can also increase the susceptibility of shellfish to disease, parasites and predation by local and invasive species (Smolowitz 2013; Burge et al. 2014).

Oyster performance peaks in salinities from 15 to 30 practical salinity units (psu), and they can withstand salinities of 0-40 psu. However, oysters tend to grow faster and be in better condition with less variationin salinity (Galtsoff 1964; Shumway 1996). Blue mussel is a euryhaline species, and can survive periodic fluctuations below 15 psu, although they do not thrive in low salinity conditions (Animal Diversity Web 2020). Although the ROMS-NWA climate projections for RCP8.5 indicate a decline in surface salinity for the U.S. continental shelf and the Gulf of Maine, the salinities for estuarine waters in Long Island Sound, Delaware Bay, and Chesapeake Bay are projected to increase by at least 2 standard deviations from the historic means.

Estuarine waters are generally more susceptible to acidification than oceanic waters because they are subject to more acid sources and are generally less buffered than oceanic waters (i.e., differences in the amount of dissolved inorganic carbon, dissolved and particulate organic carbon, total alkalinity and nutrients from riverine and estuarine sources) (Waldbusser et al. 2011; Ekstrom et al. 2015; Gledhill et al. 2015). A long-term monitoring program in the Chesapeake Bay found statistically significant declines in daytime average pH from 1985 to 2008 within polyhaline waters, but not in mesohaline waters of the Bay (Waldbusser et al. 2011). Rivers in New England that have a combination of cool temperatures, low alkalinity, and runoff typically consisting of soils containing carbonic acid, a by-product of organic decomposition, have

particularly low aragonite saturation state values (Salisbury et al. 2008). For example, in the Casco Bay during times of high discharges from the Kennebec River and down-welling (northerly) winds, acidic river waters with very low aragonite saturation state values have been recorded (Salisbury et al. 2008). Overall acidification is expected to increase, although estuaries also naturally experience daily fluxes in pH and some species acclimated to variability may be resilient to ocean acidification.

Ocean acidification will likely most directly negatively impact shellfish reefs, with larger negative effects on survival for larvae than adults (Kroeker et al. 2013). Ocean acidification is expected to reduce the shell size, thickness, calcification rates and growth, and survival of embryo and larval bivalves (Gazeau et al. 2013). Ries et al. (2009) exposed both mussels and oysters to low pH levels, and found that acidification negatively impacts oyster calcification rates whereas there was no relationship between pH and the calcification rate of blue mussels. Yet, increased water and air temperature in the northeastern U.S. is likely resulting in reduced physiological performance of mussels (Zippay and Helmuth 2012), potentially explaining why mussel beds are becoming less common in southern New England the western portionsof the Gulf of Maine when temperatures have exceeded the thermal maxima for mussels. Dodd et al. (2015) exposed oysters and their predators to increased acidification and found that acidification negatively affected oyster growth, but also reduced crab consumption of oysters. The synergistic effects of temperature, salinity, and pH on metabolism in marine mollusks may be greater than reduced pH alone. Some studies have shown additive and synergistic negative effects on bivalves and gastropods from ocean acidification and low dissolved oxygen (Kroeker et al. 2013; Gobler et al. 2014; Clark and Gobler 2016; Gobler and Bauman 2016; Griffith and Gobler 2017).

Estuarine intertidal shellfish habitats may experience disturbance from storms, which are expected to increase in frequency and intensity in the Northeast region (USGCRP 2017). Coastal storms may increase physical/mechanical disturbance and stress to shellfish reef structure and associated organisms, and repeated disturbance may prohibit formation of robust three-dimensional complex structures.

Sea-level rise poses both direct and indirect threats to intertidal habitats. Oyster reefs generally have the capacity to grow vertically and landward to keep up with sea level rise. However, because they exist across a wide geographical and environmental range and diverse landscape settings, they are influenced by different types and magnitudes of stress, which influence growth rates. Baillie and Grabowski (2018) reported lower recruitment and higher mortality of oysters at higher elevations in the intertidal zone, likely the result of desiccation and food limitation. Existing intertidal oyster reefs that have reached their growth ceiling could respond to sea level rise and the associated reduction in aerial exposure time by demonstrating enhanced vertical accretion. Enhanced accretion rates of intertidal oyster reefs have the potential to surpass all other coastal ecosystem engineers, including saltmarsh and seagrass (Rodriguez et al. 2014). Therefore, intertidal high-salinity areas may be hotspots for oyster-reef productivity (Rodriguez et al. 2014). However, many developed intertidal areas may not provide the physical space foroyster reefs to expand landward if these shorelines have been hardened (Rodriguez et al. 2014; Ridge etal. 2015).

<u>Habitat Summary</u>: Oysters were once abundant in nearly all the estuaries on the Atlantic coast (MacKenzie et al. 1997). Most oyster fisheries in Chesapeake Bay, Delaware Bay, and Pamlico Sound have been decimated (Coen and Grizzle 2007). Blue mussels, once a foundational species known to influence diversity and productivity in the Gulf of Maine, have declined since the 1970s and are now a minor contributor to the compositional patterns of intertidal communities (Sorte et al. 2017).

Shellfish reefs have been highly altered, with estimates in the U.S. suggesting that 68% of historic oysterreef extent and over 80% of the productivity of these habitats have been lost primarily due to overharvesting and destructive harvesting practices, but also as a consequence of dredge and fill activities disease, sedimentation, predators, and competition (zu Ermgassen et al. 2012). Historically, overharvesting has been the largest threat to shellfish habitats, with 85% of oyster reefs lost worldwide (Beck et al. 2011), and similar impacts having occurred in the U.S. (zu Ermgassen et al. 2012). Kirby (2004) suggested that harvesting of oysters peaked and then the fisheries collapsed in the northeastern U.S. in coastal Massachusetts and Southern New England in the early 1800's. Efforts to conserve and rebuild shellfish reefs have been challenged by anthropogenic

disturbances such as bottom water hypoxia, dredge and fill activity, shoreline hardening, diseases such as Dermo and MSX, sedimentation, predation, and competition (Rothschild et al. 1994; Kirby 2004; Beck et al. 2011; zu Ermgassen et al. 2012). High temperature and low salinity are known drivers for both MSX and Dermo (Burge et al. 2014;Burreson et al. 2000; Ford and Smolowitz 2007). Between 1990 and 1992, a dramatic range extension of Dermo disease was reported over a 500 km area in the northeastern United States from Delaware Bay, New Jersey to Cape Cod Bay, Massachusetts (Ford and Smolowitz 2007). By 1995, Dermo was reported as far north as Maine (Burge et al. 2014).

In coastal New England, many shellfish reefs suffer from poor recruitment, motivating restoration practitioners to seed reefs with juvenile oysters set on dead oyster shells at oyster hatcheries and then transplanted on shallow reefs (personal observation). Unfortunately, many of the restored oyster reefs in coastal Rhode Island's salt ponds have failed to increase natural recruitment (Grabowski and Hughes, unpublished data). Anecdotally, many of the intertidal mussel beds that were common in northeastern Massachusetts are no longer present (Grabowski, personal observation), possibly a consequence of local warming of sea and air temperatures (Zippay and Helmuth 2012).

Climate change will exacerbate other anthropogenic effects that have negatively impacted shellfish reefs. Oysters are thought to be among the most vulnerable of species to ocean acidification given its impacts on shell calcification rates and the protection that the shell provides from predators (Ries et al 2009). Although oyster reefs can grow relatively quickly and keep pace with sea-level rise, shoreline development in many regions may have removed available space for reefs to migrate landward. While mussels may be less vulnerable to acidification compared to oysters (Ries et al. 2009), they are being crowded out of coastal waters in New England likely as a consequence of air and sea water warming (Zippay and Helmuth 2012). Warming coupled with eutrophication common in many coastal estuaries will likely amplify the conditions that result in bottom water hypoxia, further contributing to subtidal shellfish reef habitat loss. Further investigation of how warming and acidification are impacting the early life history of oysters could help elucidate why recruitment failure is common in some areas ofthe northeastern U.S.

Literature Cited

Animal Diversity Web. 2020. [Internet]. *Mytilus edulis*. (accessed 31 August 2020). https://animaldiversity.org/accounts/Mytilus_edulis/

Bahr L M, Lanier WP. 1981. The ecology of intertidal oyster reefs of the South Atlantic Coast: acommunity profile. U.S. Fish and Wildlife Service. FWS/OBS-81/15.

Baillie CB, Grabowski JH. 2018. Factors affecting recruitment, growth and survival of the eastern oyster *Crassostrea virginica* across an intertidal elevation gradient in southern New England. Marine Ecology Progress Series 609:119–32. https://doi.org/10.3354/meps12830

Beck MW, Brumbaugh RD, Airoldi L, Carranza A, Coen LD, Crawford C, Defeo O, Edgar G, Hancock B, Kay M, Lenihan H, Luckenbach M, Toropova C, Zhang G. 2011. Oyster reefs at risk and recommendations for conservation, restoration and management. Bioscience 61:107-16.

Burge CA, Eakin CM, Friedman CS, Froelich B, Hershberger PK, Hofmann EE, Petes LE, Prager KC, Weil E, Willis BL, Ford SE, Harvell CD. 2014. Climate change influences on marine infectious diseases: implications for management and society. Annual Review of Marine Science 6:249-77.

Burreson EM, Stokes NA, Friedman CS. 2000. Increased virulence in an introduced pathogen: *Haplosporidium nelsoni* (MSX) in the eastern oyster *Crassostrea virginica*. Journal of Aquatic Animal Health 12(1):1-8.

Clark H, Gobler CJ. 2016. Diurnal fluctuations in CO_2 and dissolved oxygen concentrations do not provide a

refuge from hypoxia and acidification for early-life-stage bivalves. Marine Ecology Progress Series, 558:43114. https://doi.org/10.3354/meps11852

Coen LD, Grizzle RE. 2007. The importance of habitat created by molluscan shellfish to managed species along the Atlantic Coast of the United States. Atlantic States Marine Fishery Management Commission. Habitat Management Series #8.

Coen LD, Luckenbach MW, Breitburg DL. 1999. The role of oyster reefs as essential fish habitat: a reviewof current knowledge and some new perspectives. American Fisheries Society Symposium 22:438-54.

Dame RF, Zingmark RG, Haskins E. 1984. Oyster reefs as processors of estuarine materials. Journal of Experimental Marine Biology and Ecology 83:239-47.

Dodd LF, Grabowski JH, Piehler MF, Westfield I, Ries JB. 2015. Ocean acidification impairs crab foraging behaviour. Proceedings of the Royal Society B: Biological Sciences 282.

Ekstrom JA, Suatoni L, Cooley SR, Pendleton LH, Waldbusser GG, Cinner JE, Ritter J, Langdon C, van Hooidonk R, Gledhill D, Wellman K, Beck MW, Brander LM, Rittschof D, Doherty C, Edwards PET, Portela R. 2015. Vulnerability and adaptation of US shellfisheries to ocean acidification. NatureClimate Change 5(3):207-14.

Ford SE, Smolowitz R. 2007. Infection dynamics of an oyster parasite in its newly expanded range. Marine Biology 151(1):119-33.

Galtsoff PS. 1964. The American oyster *Crassostrea virginica* (Gmelin). U.S. Fish Wildlife Service Fisheries Bulletin 64:1-480.

Gazeau F, Parker LM, Comeau S, Gattuso J-P, O'Connor WA, Martin S, Pörtner H-O, Ross PM. 2013. Impacts of ocean acidification on marine shelled molluscs. Marine Biology 160(8):2207-45.

Gledhill D, White M, Salisbury J, Thomas H, Misna I, Liebman M, Mook B, Grear J, Candelmo A, Chambers RC, Gobler C, Hunt C, King A, Price N, Signorini S, Stancioff E, Stymiest C, Wahle R, Waller J, Rebuck N,Wang Z, Capson T, Morrison JR, Cooley S, Doney S. 2015. Ocean and coastal acidification off New England and Nova Scotia. Oceanography 25(2):182-97.

Gobler CJ, Baumann H. 2016. Hypoxia and acidification in ocean ecosystems: coupled dynamics and effects on marine life. Biology Letters 12(5):20150976.

Gobler CJ, DePasquale EL, Griffith AW, Baumann H. 2014. Hypoxia and acidification have additive and synergistic negative effects on the growth, survival, and metamorphosis of early life stage bivalves. PLoS One 9(1):e83648.

Griffith AW, Gobler CJ. 2017. Transgenerational exposure of North Atlantic bivalves to ocean acidification renders offspring more vulnerable to low pH and additional stressors. Scientific Reports 7:11394.

Gunter G. 1951. The West Indian tree on the Louisiana coast, and notes on growth of the Gulf Coast oysters. Science 113:16-517.

Jones SJ, Lima FP, Wethey DS. 2010. Rising environmental temperatures and biogeography: poleward range contraction of the blue mussel, *Mytilus edulis* L., in the western Atlantic. Journal of Biogeography 37(12):2243-59.

Jones SJ, Mieszkowska N, Wethey DS. 2009. Linking thermal tolerances and biogeography: *Mytilus edulis*

(L.) at its southern limit on the east coast of the United States. Biological Bulletin 217(1):73-85.

Jordan S, Stenger C, Olson M, Batiuk R, Mountford K. 1992. Chesapeake Bay dissolved oxygen goal for restoration of living resources. United States Environmental Protection Agency, Chesapeake Bay Program, Annapolis, Maryland, USA.

Kirby MX. 2004. Fishing down the coast: Historical expansion and collapse of oyster fisheries along continental margins. Proceedings of the National Academy of Science of the United States of America 101:13096-99.

Kroeker KJ, Kordas RL, Crim R, Hendriks IE, Ramajo L, Singh GS, Duarte CM, Gattuso J-P. 2013. Impacts ofocean acidification on marine organisms: quantifying sensitivities and interaction with warming. Global Change Biology 19(6):1884-96.

Lenihan HS. 1999. Physical-biological coupling on oyster reefs: how habitat structure influences individual performance. Ecological Monographs 69:251-75.

Lesser MP. 2016. Climate change stressors cause metabolic depressions in the blue mussel, *Mytilus edulis*, from the Gulf of Maine. Limnology and Oceanography 61:1705-17.

MacKenzie Jr CL, Burrell Jr VG, Rosenfield A, Hobart WL. (eds.). 1997. The history, present condition, and future of the molluscan fisheries of North and Central America and Europe, Volume 1, Atlantic and Gulf Coasts. U.S. Department of Commerce, NOAA Technical Report 127. 234 p.

Newell RIE. 1988. Ecological changes in Chesapeake Bay: are they the result of overharvesting the American oyster, *Crassostrea virginica*? *In:* MP Lynch and EC Krome, editors. Understanding the estuary: advances in Chesapeake Bay research. Chesapeake Research Consortium, Baltimore, Maryland, USA.

Newell RIE, Cornwell JC, Owens MS. 2002. Influence of simulated bivalve biodeposition and microphyrobenthos on sediment nitrogen dynamics: a laboratory study. Limnology and Oceanography 47:1367-79.

Paerl HW, Pinckney JL, Fear JM, Peierls BL. 1998. Ecosystem responses to internal and watershed organic matter loading: consequences for hypoxia in the eutrophying Neuse river estuary, North Carolina, USA. Marine Ecology Progress Series 166:17-25.

Peterson CH, Grabowski JH, Powers SP. 2003. Estimated enhancement of fish production resulting from restoring oyster reef habitat: quantitative valuation. Marine Ecology Progress Series 264:249-64.

Piehler MF, Smyth AR. 2011. Impacts of ecosystem engineers on estuarine nitrogen cycling. Ecosphere 2:art12.

Ridge JT, Rodriguez AB, Fodrie FJ, Lindquist NL, Brodeur MC, Coleman SE, Grabowski JH, Theuerkauf EJ. 2015. Maximizing oyster-reef growth supports green infrastructure with accelerating sea-level rise. Scientific Reports 5: 14785.

Ries JB, Cohen AL, McCorkle DC. 2009. Marine calcifiers exhibit mixed responses to CO_2-induced ocean acidification. Geology 37:1131-4.

Rodriguez AB, Fodrie FJ, Ridge JT, Lindquist NL, Theuerkauf EJ, Coleman SE, Grabowski JH, Brodeur MC, Gittman RK, Keller DA, Kenworthy MD. 2014. Oyster reefs can outpace sea-level rise. Nature ClimateChange 4:493-7.

Rothschild BJ, Ault JS, Goulletquer P, Heral M. 1994. Decline of the Chesapeake Bay oyster population: a century of habitat destruction and overfishing. Marine Ecology Progress Series 111:29-39.

Salisbury J, Green M, Hunt C, Campbell J. 2008. Coastal acidification by rivers: a threat to shellfish? Eos, Transactions American Geophysical Union 89(50):513-28.

Shumway S. 1996. Natural environmental factors. In: Kennedy VS, Newell RIE, Ebele AF (eds). The eastern oyster, *Crassostrea virginica*. Maryland Sea Grant, College Park, Maryland, USA. Pp. 467–513.

Smolowitz R. 2013. A review of current state of knowledge concerning *Perkinsus marinus* effects on *Crassostrea virginica* (Gmelin) (the Eastern oyster). Veterinary Pathology 50(3):404-11.

Speights C J, Silliman BR, McCoy MW. 2017. The effects of elevated temperature and dissolved ρCO_2 on a marine foundation species. Ecology and evolution 7(11):3808–14. https://doi.org/10.1002/ece3.2969

Sorte CJB, Davidson VE, Franklin MC, Benes KM, Doellman MM, Etters RJ, Hannigan RE, Lubchenco J, Menge BA. 2017. Long-term declines in an intertidal foundation species parallel shifts in community composition. Global Change Biology 23(1):341-52.

Ulanowicz RE, Tuttle JH. 1992. The trophic consequences of oyster stock rehabilitation in Chesapeake Bay. Estuaries 15:298-306.

U.S. Global Change Research Program (USGCRP). 2017. Climate Science Special Report: Fourth National Climate Assessment. Volume I. [Wuebbles, D.J., D.W. Fahey, K.A. Hibbard, D.J. Dokken, B.C. Stewart, and T.K. Maycock (eds.)]. U.S. Global Change Research Program. Washington, DC. USA. 470 pp. doi: 10.7930/J0J964J6.

Waldbusser GG, Voigt EP, Bergschneider H, Green MA, Newell RIE. 2011. Biocalcification in the eastern oyster (*Crassostrea virginica*) in relation to long-term trends in Chesapeake Bay pH. Estuaries and Coasts 34(2):221-31.

Wells HW. 1961. The fauna of oyster beds, with special reference to the salinity factor. Ecological Monographs 31:239-66.

Zippay ML, Helmuth B. 2012. Effects of temperature change on mussel, *Mytilus*. Integrative Zoology 7:312-27.

zu Ermgassen PSE, Grabowski JH, Gair JR, Powers SP. 2016. Quantifying fish and mobile invertebrate production from a threatened nursery habitat. Journal of Applied Ecology 53:596-606.

zu Ermgassen PSE, Spalding MD, Blake B, Coen LD, Dumbauld B, Geiger S, Grabowski JH, Grizzle R, Luckenbach M, McGraw K, Rodney W, Ruesink JL, Powers SP, Brumbaugh RD. 2012. Historical ecology with real numbers: past and present extent and biomass of an imperilled estuarine habitat. Proceedings of the Royal Society B: Biological Sciences 279:3393-400.

Estuarine Intertidal Rocky Bottom

System: Estuarine

Subsystem: Intertidal

Class: Rocky Bottom

Sub-class: Bedrock, Rubble, Cobble, Gravel

Geographic Area: Entire Area

Overall Vulnerability Rank = Moderate

Habitat Sensitivity = Low

Climate Exposure = Very High

	**Estuarine Intertidal Rocky Bottom**	Attribute Mean	Data Quality	Distribution of Expert Scores
Sensitivity Attributes	Habitat condition	2.2	2.1	
	Habitat fragmentation	1.9	1.7	
	Distribution/Range	2.4	1.7	
	Mobility/Ability to spread or disperse	2.4	1.7	
	Resistance	1.8	1.5	
	Resilience	2.4	2	
	Sensitivity to changes in abiotic factors	2	2	
	Sensitivity and intensity of non−climate stressors	2.8	2.1	
	Dependency on critical ecological linkages	2	1.8	
	Sensitivity Component Score	Low		
Exposure Factors	Sea surface temp	n/a	n/a	
	Bottom temp	n/a	n/a	
	Air temp	4	3	
	River temp	n/a	n/a	
	Surface salinity	2	2.1	
	Bottom salinity	n/a	n/a	
	pH	4	2	
	Sea level rise	3.9	2.2	
	Precipitation	2.8	2.1	
	Floods	n/a	n/a	
	Droughts	n/a	n/a	
	Exposure Component Score	Very High		
**Overall Vulnerability Rank**		**Moderate**		

Legend: Low, Moderate, High, Very High

Habitat Name: Estuarine Intertidal Rocky Bottom

System**:** Estuarine

Subsystem: Intertidal

Class: Rocky Bottom

Sub-class: Bedrock, Rubble, Cobble, Gravel

Geographic Area: Entire Area

Habitat Description: This habitat sub-class includes natural bedrock, rubble, cobble/gravel in the estuarine intertidal zone. In addition, infauna and epifauna and flora associated with rocky bottom areincluded, but not specific habitats that were assessed in other habitat narratives (i.e., non-calcareous algal and rooted vascular beds, coral-dominated hard bottom, mollusk reef). Calcareous algae is included in this class.

Natural rocky estuarine habitats occur across a wide latitudinal range in New England, but are rare in theMid-Atlantic. The extent of intertidal rocky habitats in the Gulf of Maine is limited by the tidal range and by shoreline gradient. New England, particularly the Gulf of Maine, has a larger tidal range and steeper shorelines than the Mid-Atlantic. Natural rocky habitats range from granule/pebble (or gravel) to cobbles, boulders, and ledge/bedrock. Biota associated with all these habitat types also varies.

Natural rocky estuarine habitats in New England are partially continuous with multiple, moderately-sized patches that vary between estuaries. Within individual estuaries, rocky shorelines and bottom habitats are usually in close proximity to each other.

Overall Climate Vulnerability Rank: Moderate (31% certainty from bootstrap analysis). The probabilityof a High climate vulnerability in the bootstrap results is 64%, which differs from the results of the categorical vulnerability rank (High). This is due to the Low sensitivity rank, combined with a Very High exposure rank, indicating that there is a likelihood the overall climate vulnerability rank could be High.

Climate Exposure: Very High. Three exposure factors contributed to the Very High exposure score: Air Temperature (3.9), pH (4.0), and Sea Level Rise (SLR) (3.9). Precipitation also scored relatively high (2.8), largely driven by a projected increase in the frequency and intensity of extreme rain events. Projected changes in air temperature and pH (ocean acidification) were very high throughout the range. SLR is expected to impact the entire study area but the greatest relative rise is projected in the Mid-Atlantic. The intertidal nature of this habitat places it at the nexus of significant atmospheric and oceanic change.

Habitat Sensitivity: Low. Most sensitivity attributes were centered around the Moderate scoring bin, although individual scores were placed in the Low, Moderate, and High scoring bins by the scorers for most attributes. This may reflect high variability in the expected sensitivities of rocky habitat throughout the study area. No sensitivity attributes scored above 3.0, with the highest scores for Sensitivity and Intensity of Non-Climate Stressors (2.8), Resilience (2.3), Distribution/Range (2.4), and Mobility/Ability to Spread or Disperse (2.4).

Data Quality & Gaps: Four of the five climate exposure factors had relatively low data quality scores (≤2.2), while the highest (Air Temperature) was 3.0. Data quality is generally lower for estuarine habitatsdue to the low resolution of CMIP5 and ROMS-NWA projections for climate exposure in estuaries and nearshore, shallow coastal areas, which increases the uncertainty with overlap in climate exposure projections.

Seven of the nine sensitivity attribute data quality scores were less than or equal to 2.0. Resistance scored 1.5; Habitat Fragmentation, Distribution/Range, and Mobility/Ability to Spread or Disperse scored 1.7; Dependency on Critical Ecological Linkages scored 1.8; and Resilience and Sensitivity to Changes in Abiotic Factors scored 2. The low data quality scores may reflect uncertainty in the sensitivities of intertidal rocky bottom habitats throughout the study area. For example, while the abiotic component of rocky bottom habitats are both resistant and resilient to most stressors, the fauna and flora associated with the habitat may be sensitive to higher temperature and physical impacts associated with higher intensity storms. In addition,

high-resolution maps for natural rocky habitat over the study area are limited, which increases the uncertainty with the overlap with climate exposure projections.

Positive or Negative Climate Effect in the Northeast U.S.: The effect of climate change on estuarine intertidal rocky bottoms in the Northeast U.S. is expected to be evenly split between neutral or negative effects (50% of the experts' scores were neutral and 45% were negative).

Climate Effects on Habitat Condition and Distribution: Extreme precipitation events have increased in frequency in the study area (Easterling et al. 2017) and are projected to increase further by the end of the century (USGCRP 2017). In addition, mid-latitude synoptic storm systems (e.g, blizzards and nor'easters in the northeast region) have increased in intensity and their tracks shifted northward (Vose et al. 2014; Wang et al. 2012). The intensity of tropical hurricanes in the Atlantic Ocean have also increased (Emanuel 2005; Webster et al. 2005; Kossin et al. 2007; Hartmann et al. 2013). Various researchers have hypothesized that an increase in sea surface temperature will increase the maximum potential intensity of tropical cyclones and should be reflected by an increase in the frequency of the strongest hurricanes (Trenberth 2005; Kossin et al. 2007; Knutson et al. 2010).

Heavy precipitation events can cause compounding or synergistic effects, such as increased sedimentand stormwater runoff, overloading of municipal or agricultural waste systems, and large freshwater input from rivers and streams. Heavy precipitation events and coastal storms can increase physical impacts to intertidal rocky habitats, such as scouring and erosion of smaller-grain rocky bottoms (e.g.,gravel and cobble) and the flora and fauna associated with them.

Sea level rise will cause intertidal rocky bottom habitats to be inundated more frequently, and entirely inthe lower intertidal zone. This may increase the rate of coastal erosion. Gravel beaches may be threatened by rising sea levels, and the rates of erosion are determined by the rate of sea level rise but are also influenced by the intensity and frequency of storms, tectonic events, and other factors (Nicholls et al. 2007). Cliffed coasts are also vulnerable to coastal erosion and sea level rise, although hard rock cliffs are more resistant to erosion than softer lithologies (Nicholls et al. 2007; Ashton et al. 2011).

Depending upon the grain size of the substrate, intertidal rocky bottom habitats have limited mobility tomove inland as they become inundated with higher sea levels, which may be further limited by hardened shoreline structures. However, most organisms associated with intertidal rocky habitats have the capacity to spread through larval dispersal, propagules, or vegetative growth, as long as suitable hard substrate is present. Rocky substrate is relatively common in New England, but less common in theMid-Atlantic. The fauna and flora associated with hard substrates in the Mid-Atlantic region may have a lower probability of settling and establishing on natural rocky substrates as a result of less abundances of this habitat. Although artificial hard substrates (e.g., rock revetments, groins, jetties, seawalls) areavailable for settlement in the Mid-Atlantic, studies suggest the abundance and diversity of flora and fauna associated with some artificial hard substrates may be lower than natural rock substrates (Williams and Thom 2001; Chapman 2003; Gittman et al. 2016), which may reflect reduced success in settlement, growth, and survival.

In addition, some studies suggest a higher incidence of marine exotic/invasive species associated with artificial structures compared to native material (Tyrrell and Byers 2007; Pappal 2010; Geraldi et al. 2013). Increased reproduction of marine invasive species, combined with limited biological resistance in regions with cooler water temperatures, may lead to community state changes in shallow habitats in thestudy area (Dijkstra et al. 2017). Warming waters have been shown to eliminate thermal barriers that historically limit reproductive success of marine invasive species (e.g., the tunicate *Botrylloides violaceus*) (Dijkstra et al. 2017). Marine invasive species, likely introduced via maritime transport vectors, are known to compete for space and foul benthic substrates (Pappal 2010) and may replace native rocky bottom fauna and flora in portions of the study area (Scheibling and Gagnon 2006; Trott and Entreline 2019).

Flora and fauna associated with estuarine intertidal rocky habitats are generally adapted to highly variable conditions such as temperature, desiccation, salinity, and higher wave energy. However, these species may experience impacts as air and water temperatures become more extreme and exceed threshold tolerances. Calcareous algae and gastropod fauna associated with intertidal rocky habitats have shown sensitivity to low pH conditions (Gazeau et al. 2013; Kroeker et al. 2013).

<u>Habitat Summary</u>: Natural rocky habitats in the intertidal zone are under threat from growing population density and coastal development, which are expected to cause increased habitat loss in the future (Sorte et al. 2017). Although Gittman et al. (2016) reported the biodiversity and abundance of flora and fauna associated with engineered riprap and breakwaters are not vastly different from natural shorelines, the effects were found to be highly heterogeneous across habitat type and species and the results are subject to some uncertainty. Increased shoreline hardening in heavily populated and industrialized areas is expected as sea level rise and greater frequency of extreme weather events become more severe and widespread. Titus et al. (2009) estimated that almost 60% of land within 1 m above the high tide line on the U.S. Atlantic coast (Florida to Massachusetts) is developed or expected to be developed.

Intertidal flora and fauna are also sensitive to pollution (e.g., oil spills/ polyaromatic hydrocarbons, heavy metals), and exposure to some pollutants may be more common than subtidal habitats due to the proximity to impervious surfaces and development. Fauna and flora can be highly sensitive to eutrophication from increased nutrient runoff.

Bedrock and boulders are highly resistant to disturbance, to a greater degree than smaller substrates like gravel and cobble. When physically disturbed (e.g., ice scour, storms), the function of these habitats is not impaired, though they may be temporarily buried by mobile sand. Some attached species recoverquickly, while others may take years (Bertness et al. 2002). In the presence of both native (e.g., littorinid snails) and invasive (e.g., green crabs) predators, recovery of mussels and macroalgae in the intertidal zone can take years (Bertness et al. 2002), and is dependent on species-specific methods of reproduction, patch size, and season of disturbance (Kim & DeWreede 1996). Mobile species quickly re-occupy rocky habitats after disturbance.

Intertidal rocky infauna and epifauna are sensitive to invasive species, including green crab (*Carcinus maenas*) and the colonial ascidian (*Botrylloides violaceus*). The green crab has established populations inNew England and northern Mid-Atlantic in both subtidal and intertidal zones. It is a predator of many forms of shore life, including worms and mollusks (Global Invasive Species Database 2020). Although cold water temperatures were thought to limit the northward expansion of the green crab (Global Invasive Species Database 2020), recent research suggests both warm-water and cold-water geneticlineages exist, and the species has successfully invaded intertidal and shallow subtidal habitats throughout the study area (Lehnert et al. 2018).

Species that graze (e.g., sea urchins) on the epiflora and epifauna associated with rocky bottom habitatscan control their abundances. Likewise, top predators (e.g., cod) can control the populations of grazers. For example, in some areas of the Gulf of Maine, urchin populations have exploded and decimated kelp beds (i.e., "urchin barrens"), which has been attributed to overfishing of the top predators (Steneck et al. 2002). Likewise, the collapse in the populations of sea urchins can cause proliferation of macroalgae species that reduces species diversity of rocky habitats, and can interfere with settlement of benthic and demersal larval life stages.

<u>Literature Cited</u>

Ashton AD, Walkden MJA, Dickson ME. 2011. Equilibrium responses of cliffed coasts to changes in the rate of sea level rise. Marine Geology 284(1):217-29.

Bertness MD, Trussell GC, Ewanchuk PJ, Silliman BR. 2002. Do alternate stable community states exist in the Gulf of Maine rocky intertidal zone? Ecology 83(12):3434-48.

Browne MA, Chapman MG. 2017. The ecological impacts of reengineering artificial shorelines: The state of the science. *In:* Bilkovic DM, Mitchell MM, La Peyre MK, Toft JD (Eds). Living shorelines: the science and management of nature-based coastal protection (First Edition, 439-457). Boca Raton. https://doi.org/10.1201/9781315151465.

Chapman MG. 2003. Paucity of mobile species on constructed seawalls: effects of urbanization on biodiversity. Marine Ecology Progress Series 264:21-9.

Dijkstra JA, Westerman EL, Harris LG. 2017. Elevated seasonal temperatures eliminate thermal barriers of reproduction of a dominant invasive species: A community state change for northern communities? Diversity and Distributions 23:1182–92.

Easterling, D.R., K.E. Kunkel, J.R. Arnold, T. Knutson, A.N. LeGrande, L.R. Leung, R.S. Vose, D.E. Waliser, and M.F. Wehner, 2017: Precipitation change in the United States. In *Climate Science Special Report: Fourth National Climate Assessment, Volume I*. D.J. Wuebbles, D.W. Fahey, K.A. Hibbard, D.J. Dokken, B.C. Stewart, and T.K. Maycock, Eds. U.S. Global Change Research Program, pp. 207-230, doi:10.7930/J0H993CC.

Emanuel K. 2005. Increasing destructiveness of tropical cyclones over the past 30 years. Nature 436(7051):686-8.

Gazeau F, Parker LM, Comeau S, Gattuso J-P, O'Connor WA, Martin S, Pörtner H-O, Ross PM. 2013. Impacts of ocean acidification on marine shelled molluscs. Marine Biology 160(8):2207-45.

Geraldi NR, Smyth AR, Piehler MF, Peterson CH. 2013. Artificial substrates enhance non-native macroalga and N_2 production. Biological Invasions 16(9):1819-31.

Gittman, R.K., S.B. Scyphers, C.S. Smith, I.P. Neylan, and J.H. Grabowski. 2016. Ecological consequences of shoreline hardening: a meta-analysis. BioScience 66(9):763-73.

Global Invasive Species Database (GISD). 2020. [Internet]. Species profile: *Carcinus maenas*. (Accessed 8 Dec. 2020). http://www.iucngisd.org/gisd/species.php?sc=114.

Hartmann DL, Klein Tank AMG, Rusticucci M, Alexander LV, Brönnimann S, Charabi YA-R, Dentener FJ, Dlugokencky EJ, Easterling DR, Kaplan A, Soden BJ, Thorne PW, Wild M, Zhai P. 2013. Observations: atmosphere and surface. Climate change 2013. The physical science basis. Contribution of Working Group I to the Fifth Assessment Report of the Intergovernmental Panel on Climate Change. [StockerT, Qin D, Plattner G-K, Tignor M, Allen S, Boschung J, Nauels A, Xia Y, Bex V, Midgley P (eds)]. Intergovernmental Panel on Climate Change. 159-254.

Kim JH, DeWreede RE. 1996. Effects of size and season of disturbance on algal patch recovery in a rocky intertidal community. Marine Ecology Progress Series 133:217-28.

Knutson TR, McBride JL, Chan J, Emanuel K, Holland G, Landsea C, Held I, Kossin JP, Srivastava AK, Sugi M. 2010. Tropical cyclones and climate change. Nature Geoscience 3(3):157-63.

Kossin JP, Knapp KR, Vimont DJ, Murnane RJ, Harper BA. 2007. A globally consistent reanalysis of hurricane variability and trends. Geophysical Research Letters 34(4):1-6.

Kroeker KJ, Kordas RL, Crim R, Hendriks IE, Ramajo L, Singh GS, Duarte CM, Gattuso J-P. 2013. Impacts of ocean acidification on marine organisms: quantifying sensitivities and interaction with warming. Global Change Biology 19(6):1884-96.

Lehnert SJ, DiBacco C, Jeffery NW, Blakeslee A, Isaksson J, Roman J, Wringe BF, Stanley R, Matheson K, McKenzie CH, Hamilton LC, Bradbury IR. 2018. Temporal dynamics of genetic clines of invasive European green crab (*Carcinus maenas*) in eastern North America. Evolutionary Applications 11(9):1656–70. https://doi.org/10.1111/eva.12657

Nicholls RJ, Wong PP, Burkett V, Codignotto J, Hay J, McLean R, Ragoonaden S, Woodroffe CD. 2007. Coastal systems and low-lying areas. Climate change 2007: impacts, adaptation and vulnerability. Contribution of Working Group II to the Fourth Assessment Report of the Intergovernmental Panel on Climate Change. [Parry ML, Canziani OF, Palutikof JP, van der Linden PJ, Hanson CE (eds)]. Intergovernmental Panel on Climate Change. 315-56.

Pappal A. 2010. Marine invasive species. State of the Gulf of Maine Report. [Currier P, Gould D, Hertz L, Huston J, Tremblay ML (eds)]. The Gulf of Maine Council on the Marine Environment. 1-21.

Scheibling RE, Gagnon P. 2006. Competitive interactions between the invasive green alga *Codium fragile* ssp. *tomentosoides* and native canopy-forming seaweeds in Nova Scotia (Canada). Marine Ecology Progress Series 325:1–14. DOI:10.3354/meps325001.

Sorte CJB, Davidson VE, Franklin MC, Benes KM, Doellman MM, Etters RJ, Hannigan RE, Lubchenco J, Menge BA. 2017. Long-term declines in an intertidal foundation species parallel shifts in community composition. Global Change Biology 23(1):341-52.

Steneck R, Graham MH, Bourque BJ, Corbett D, Erlandson JM, Estes JA, Tegner MJ. 2002. Kelp forest ecosystems: biodiversity, stability, resilience and future. Marine Sciences Faculty Scholarship. 65. https://digitalcommons.library.umaine.edu/sms_facpub/65

Titus JG, Hudgens DE, Trescott DL, Craghan M, Nuckols WH, Hershner CH, Kassakian JM, Linn CJ, Merritt PG, McCue TM, O'Connell JF, Tanski J, Wang J. 2009. State and local governments plan for development of most land vulnerable to rising sea level along the US Atlantic coast. Environmental Research Letters 4(4):1-7.

Trenberth K. 2005. Uncertainty in hurricanes and global warming. Science 308(5729):1753-4.

Trott TJ, Enterline C. 2019. First record of the encrusting bryozoan *Cribrilina* (*Juxtacribrilina*) *mutabilis* (Ito, Onishi and Dick, 2015) in the Northwest Atlantic Ocean. BioInvasions Records 8.

Tyrrell MC, Byers JE. 2007. Do artificial substrates favor nonindigenous fouling species over native species? Journal of Experimental Marine Biology and Ecology 342(1):54-60.

U.S. Global Change Research Program (USGCRP). 2017. Climate Science Special Report: Fourth National Climate Assessment, Volume I [Wuebbles, D.J., D.W. Fahey, K.A. Hibbard, D.J. Dokken, B.C. Stewart, and T.K. Maycock (eds.)]. U.S. Global Change Research Program, Washington, DC, USA, 470 pp, doi: 10.7930/J0J964J6.

Vose RS, Applequist S, Bourassa MA, Pryor SC, Barthelmie RJ, Blanton B, Bromirski PD, Brooks HE, DeGaetano AT, Dole RM, Easterling DR, Jensen RE, Karl TR, Katz RW, Klink K, Kruk MC, Kunkel KE, MacCracken MC, Peterson TC, Shein K, Thomas BR, Walsh JE, Wang XL, Wehner MF, Wuebbles

DJ,Young RS. 2014. Monitoring and understanding changes in extremes: extratropical storms, winds,and waves. Bulletin of the American Meteorological Society 95(3):377-86.

Wang XL, Feng Y, Compo GP, Swail VR, Zwiers FW, Allan RJ, Sardeshmukh PD. 2012. Trends and low frequency variability of extra-tropical cyclone activity in the ensemble of twentieth century reanalysis. Climate Dynamics 40(11-12):2775-800.

Webster PJ, Holland GJ, Curry JA, Chang H-R. 2005. Changes in tropical cyclone number, duration, and intensity in a warming environment. Science 309(5742):1844-6.

Williams GD, Thom RM. 2001. Marine and estuarine shoreline modification issues. [Internet]. White Paper submitted to Washington Department of Fish and Wildlife, Washington Dept. of Ecology,Washington Dept. of Transportation. Sequim (WA): Battelle Memorial Institute. 99 p.

Estuarine Intertidal Artificial Structures

System: Estuarine

Subsystem: Intertidal

Class: Rocky Bottom

Sub-class: Artificial Structures

Geographic Area: Entire Area

Overall Vulnerability Rank = Moderate

Habitat Sensitivity = Low

Climate Exposure = Very High

	**Estuarine Intertidal Artificial Structures**	Attribute Mean	Data Quality	Distribution of Expert Scores
Sensitivity Attributes	Habitat condition	2	1.9	
	Habitat fragmentation	1.9	1.5	
	Distribution/Range	1.6	1.5	
	Mobility/Ability to spread or disperse	1.4	1.5	
	Resistance	1.4	1.3	
	Resilience	1.8	1.8	
	Sensitivity to changes in abiotic factors	1.4	1.8	
	Sensitivity and intensity of non−climate stressors	1.8	1.9	
	Dependency on critical ecological linkages	1.4	1.6	
	Sensitivity Component Score	Low		
Exposure Factors	Sea surface temp	1	0	
	Bottom temp	1	0	
	Air temp	3.9	3	
	River temp	1	0	
	Surface salinity	2.4	2.1	
	Bottom salinity	1	0	
	pH	4	2	
	Sea level rise	3.9	2.2	
	Precipitation	2.7	2.1	
	Floods	1	0	
	Droughts	1	0	
	Exposure Component Score	Very High		
	**Overall Vulnerability Rank**	**Moderate**		

Low
Moderate
High
Very High

Habitat Name: Estuarine Intertidal Artificial Structures
System**:** Estuarine
Subsystem: Intertidal
Class: Rocky Bottom
Sub-class: Artificial Structures
Geographic Area: Entire Area

Habitat Description: This habitat sub-class includes riprap, artificial reefs and wrecks, groins/jetties, and living shorelines in the estuarine intertidal zone. Riprap and breakwaters are common throughout the region in populated coastal areas. This sub-class also includes the epibenthic flora and fauna associated with these structures, but does not include specific habitats that were assessed separately (i.e., non-calcareous algae).

Overall Climate Vulnerability Rank: Moderate (100% certainty from bootstrap analysis).

Climate Exposure: Very High. Three exposure factors contributed to the Very High exposure score: Air Temperature (3.9), pH (4.0), and Sea Level Rise (SLR) (3.9). Precipitation also scored relatively high (2.7), largely driven by a projected increase in the frequency and intensity of rain events. Projected changes in air temperature and pH were very high throughout the range. SLR is expected to impact the entire study area but the greatest relative rise is projected in the Mid-Atlantic. The intertidal nature of this habitat places it at the nexus of significant atmospheric and oceanic change.

Habitat Sensitivity: Low. Most sensitivity attributes were centered around the Low and Moderate scoring bins. No sensitivity attributes scored above 2.0. The highest scoring attributes included Habitat Condition (2.0), Habitat Fragmentation (both 1.9), and Resilience and Sensitivity and Intensity of Non-Climate Stressors (both 1.8).

Data Quality & Gaps: Four of the five exposure factors had relatively low data quality scores (≤2.2), while the highest (Air Temperature) was 3.0. The relatively low score for Precipitation (2.1) is likely due to spatial variability in the projected changes in extreme events. In addition, data quality is lower for estuarine habitats due to the low resolution of CMIP5 and ROMS-NWA projections for climate exposure in estuaries and nearshore, shallow coastal areas, which increases the uncertainty with overlap in climate exposure projections. Information about the distribution of artificial structures is limited, not consolidated, or easily accessible. The diversity of artificial structure types included in this habitat also make it difficult to assess climate vulnerability in a uniform manner.

All nine of the sensitivity attribute data quality scores were ≤2.0 (ranging from 1.3 to 1.9). The low data quality scores may indicate uncertainty in the expected responses of fauna and flora associated with thehabitat.

Positive or Negative Climate Effect in the Northeast U.S.: The effect of climate change on estuarine artificial intertidal hard bottom habitats in the Northeast U.S. is generally expected to be neutral (65% of the experts' scores were neutral, 25% were negative, and 10% were positive). The divergence in directional effects scores may be due to the diversity of artificial structure types included in this single habitat type.

Climate Effects on Habitat Condition and Distribution: Artificial intertidal hard structures are not expected to be significantly impacted by climate change as compared with natural habitats. However, some of the flora and fauna associated with intertidal artificial structures may be sensitive to climate impacts including increasing temperatures, changing salinity, pH, and high wave energy. Generally, organisms associated with estuarine intertidal rocky habitats are adapted to highly variable conditions such as extreme temperatures (and desiccation), changing salinity, and high wave energy. However, they may experience impacts as temperatures become more extreme and reach tolerance thresholds. Intertidal biota are also sensitive physical removal, damage, and predation.

Shoreline structures are designed to deflect, withstand, or absorb wave action. Increased shoreline hardening

in heavily populated and industrialized coastal areas is expected as climate change effects become more severe and widespread, in an attempt to reduce erosion and provide protection from storm surge (Balouskas and Targett 2018). In many places, shorelines are being converted from natural to artificial structures as coastal landowners respond to the effects of climate change. Titus et al. (2009) estimated that almost 60% of land within 1 m above the high tide line on the U.S. Atlantic coast (Florida to Massachusetts) is developed or expected to be developed. This would increase the availability of artificial structures for flora and fauna associated with them, particularly for non-native and invasive species that may benefit by warming coastal waters and greater disturbances (Stachowicz et al. 2002; Valentine 2009; Sorte et al. 2010). Artificial reefs are expected to have limited effects from climate change, although they could be impacted by larger and more intense storms and sea level rise.

Living shorelines, or shoreline structures that incorporate natural habitat elements into their design (such as wetland vegetation, seagrasses, coarse woody debris, or shellfish reefs), may be more resilient to SLR and storm surge depending on their setting, design, and maintenance (Mitchell and Bilkovic 2019). For example, planted marshes may be able to spread inland if migration corridors are available. Hybrid designs that incorporate vegetation and non-living material may be better able to withstand wave action (Browne and Chapman 2017). However, as storms and sea level rise increasingly impact shorelines, a human response may be to replace the living shoreline with harder structures.

Habitat Summary: The condition of artificial hard structure habitat, in terms of habitat function, is inherently poor, with lower species diversity, habitat complexity, and habitat function compared with natural rocky habitats (Bulleri and Chapman 2010; Balouskas and Targett 2018). Artificial shorelines can impact the growth, interactions, larval dispersal, and foraging behavior of species that live on them (e.g., limpets), and alter fish assemblages in adjacent waters (Browne and Chapman 2017). The make-up of biological communities on riprap, breakwaters, and other artificial structures differs from natural rocky habitats (Bulleri and Chapman 2010). They support fewer mobile species and more sessile animals and algae as compared with natural intertidal rocky habitats (Chapman 2003).

The material and design of engineered shorelines are important for their function as habitat, and the biodiversity of species they support. For example, sandstone has been found to support a higher diversity of species than concrete, and surveys have found more mobile species and algae on seawalls with sun exposure, and more sessile animals on shaded seawalls (Browne and Chapman 2017). Low structural complexity of engineered stone structures may limit habitat value for mobile species, which may be partly ameliorated by adding pits, crevices, and water-retaining features to seawalls (Browne and Chapman 2017). Incorporating natural habitat elements into artificial shoreline structures can improve habitat function (Bulleri and Chapman 2010). For example, studies have found that a higher density of oysters on seawalls provides better habitat and refuge for limpets and whelks (Jackson et al. 2008; Klein et al. 2011). Low species diversity may limit the resilience of the biotic community associated with artificial structures.

Hard structures in the intertidal zone are exposed to greater runoff and pollution than subtidal habitats, especially in urbanized areas. Flora and fauna associated with artificial rocky habitat may be sensitive to pollution and eutrophication similar to natural habitats, although invasive species may be less sensitive. Some studies suggest a higher incidence of invasive species associated with artificial structures compared to native material (Tyrrell and Byers 2007; Pappal 2010; Geraldi et al. 2013). This may be associated with greater survival of invasive species on disturbed areas, but it could also suggest higher planktonic stage survival and greater dispersal capacity.

Artificial hard structures are highly resistant to disturbance. In fact, many shoreline structures are built to protect shorelines and withstand disturbance. Non-mobile (fouling) species that attach to riprap and rocky breakwaters re-colonize quickly following disturbance.

Literature Cited

Balouskus RG, Targett TE. 2018. Impact of armored shorelines on shore-zone fish density in a Mid-Atlantic estuary: modulation by hypoxia and temperature. Estuaries and Coasts 41(Suppl 1):S144-58.

Browne MA, Chapman MG. 2017. The ecological impacts of reengineering artificial shorelines: The state of the science. *In:* Bilkovic DM, Mitchell MM, La Peyre MK, Toft JD (Eds). Living shorelines: the science and management of nature-based coastal protection (First Edition, 439-457). Boca Raton. https://doi.org/10.1201/9781315151465.

Bulleri F, Chapman MG. 2010. The introduction of coastal infrastructure as a driver of change in marine environments. Journal of Applied Ecology 47:26-35.

Chapman MG. 2003. Paucity of mobile species on constructed seawalls: Effects of urbanization on biodiversity. Marine Ecology Progress Series 264:21–9.

Geraldi NR, Smyth AR, Piehler MF, Peterson CH. 2013. Artificial substrates enhance non-native macroalga and N_2 production. Biological Invasions 16(9):1819-31.

Jackson AC, Chapman MG, Underwood AJ. 2008. Ecological interactions in the provision of habitat by urban development: Whelks and engineering by oysters on artificial seawalls. Austral Ecology 33:307–16.

Klein JC, Underwood AJ, Chapman MG. 2011. Urban structures provide new insights into interactions among grazers and habitat. Ecological Applications 21:427–38.

Mitchel, M. and Bilkovic, D.M. 2019. Embracing dynamic design for climate-resilient living shorelines. Journal of Applied Ecology 56(5):1099-105. https://doi.org/10.1111/1365-2664.13371

Pappal A. 2010. Marine invasive species. State of the Gulf of Maine Report. [Currier P, Gould D, Hertz L, Huston J, Tremblay ML (eds)]. The Gulf of Maine Council on the Marine Environment. 1-21.

Sorte CJB, Williams SL, Zerebecki RA. 2010. Ocean warming increases threat of invasive species in a marine fouling community. Ecology 91(8):2198-204.

Stachowicz JJ, Terwin JR, Whitlatch RB, Osman RW. 2002. Linking climate change and biological invasions: ocean warming facilitates nonindigenous species invasions. Proceedings of the National Academy of Sciences 99(24):15497-500.

Titus JG, Hudgens DE, Trescott DL, Craghan M, Nuckols WH, Hershner CH, Kassakian JM, Linn CJ, Merritt PG, McCue TM, O'Connell JF, Tanski J, Wang J. 2009. State and local governments plan for development of most land vulnerable to rising sea level along the US Atlantic coast. Environmental Research Letters 4(4):1-7.

Tyrrell MC, Byers JE. 2007. Do artificial substrates favor nonindigenous fouling species over native species? Journal of Experimental Marine Biology and Ecology 342(1):54-60.

Valentine P. 2009. Larval recruitment of the invasive colonial ascidian *Didemnum vexillum*, seasonal water temperatures in New England coastal and offshore waters, and implications for spread of the species. Aquatic Invasions 4(1):153-68.

Estuarine Intertidal Mud Bottom

System: Estuarine

Subsystem: Intertidal

Class: Unconsolidated Bottom

Sub-class: Mud

Geographic Area: Entire Area

Overall Vulnerability Rank = Moderate

Habitat Sensitivity = Low

Climate Exposure = Very High

	Estuarine Intertidal Mud Bottom	Attribute Mean	Data Quality	Distribution of Expert Scores
Sensitivity Attributes	Habitat condition	2.4	2.6	
	Habitat fragmentation	1.9	2	
	Distribution/Range	1.5	2.1	
	Mobility/Ability to spread or disperse	1.8	1.7	
	Resistance	2.4	2.4	
	Resilience	2.2	2.1	
	Sensitivity to changes in abiotic factors	2.2	2.3	
	Sensitivity and intensity of non−climate stressors	2.6	2.4	
	Dependency on critical ecological linkages	2.2	1.7	
	Sensitivity Component Score	Low		
Exposure Factors	Sea surface temp	n/a	n/a	
	Bottom temp	n/a	n/a	
	Air temp	4	3	
	River temp	n/a	n/a	
	Surface salinity	2.6	2.1	
	Bottom salinity	n/a	n/a	
	pH	4	2	
	Sea level rise	3.7	2.2	
	Precipitation	2.8	2.1	
	Floods	n/a	n/a	
	Droughts	n/a	n/a	
	Exposure Component Score	Very High		
**Overall Vulnerability Rank**		**Moderate**		

Low
Moderate
High
Very High

Habitat Name: Estuarine Intertidal Mud Bottom

System: Estuarine

Subsystem: Intertidal

Class: Unconsolidated Bottom

Sub-class: Mud

Geographic Area: Entire Area

<u>Habitat Description</u>: This sub-class includes intertidal mud habitats, as well as the overtopping water column. This habitat type also includes the epifauna and infauna associated with mud bottoms, such as non-reef-forming mollusks (e.g., soft-shell clams, hard clams, sea scallops, surf clams, ocean quahogs), marine worms, small crustaceans, gastropods, and polychaetes). This sub-class excludes specific habitats identified elsewhere (i.e., non-calcareous algal bed, rooted vascular beds, and reef-forming mollusks, suchas blue mussels, eastern oysters). This habitat occurs in the estuarine system where salinity is between 0.5 and 30 ppt.

<u>Overall Climate Vulnerability Rank</u>: Moderate (62% certainty from bootstrap analysis). The majority of the bootstrap results match the results of the categorical vulnerability rank, but 31% of the bootstrap results were in the High vulnerability rank and 7% were in the Low. This indicates that this habitat is in the high range of the Moderate vulnerability rank.

<u>Climate Exposure</u>: Very High. The overall Very High exposure score was influenced by three Very High attribute means: Air Temperature (4), pH (4), and Sea Level Rise (SLR) (3.7). Estuarine intertidal mud also scoredrelatively high for Precipitation (2.8) and Surface Salinity (2.6). The intertidal nature of this habitat places it at the nexus of significant atmospheric change in the form of increases in extreme precipitation events and air temperature, and oceanic change such as decreasing pH and rising sea level.

<u>Habitat Sensitivity</u>: Low. All of the nine sensitivity attribute means were ≤3.0. The highest sensitivity attribute means were for Sensitivity and Intensity of Non-Climate Stressors (2.6), Resistance (2.4), and Habitat Condition (2.4). The scores are generally indicative of the resilient nature of mud habitats and an acknowledgement that the threat to intertidal habitats may be associated more with coastal development and proximity to human activity than climate.

<u>Data Quality & Gaps</u>: The data quality scores for four of the five climate exposure factors (Surface Salinity, pH, Precipitation and SLR) were scored relatively low (≤2.2). The relatively low score for Precipitation (2.1) may reflect uncertainty in the specific spatial nature of projected changes in extreme events. In addition, data quality is lower for estuarine habitats due to the low resolution of CMIP5 and ROMS-NWA projections for climate exposure in estuaries and nearshore, shallow coastal areas. For pH, the lowest data quality score of the climate exposure factors, this likely reflects the uncertainty in projecting changes to pH in nearshore, shallow water habitats.

For habitat sensitivity, five of the nine attributes had relatively low (<2.2) data quality scores (Habitat Fragmentation, Distribution and range, Mobility/Ability to Spread or Disperse, Resilience, and Dependency on Critical Ecological Linkages). These low data quality scores for habitat sensitivity may reflect the uncertainty for how these habitats, which are adapted to relative extremes in temperature, tidal elevation, dissolved oxygen, pH, and a range of other natural and anthropogenic disturbances.

<u>Positive or Negative Climate Effect in the Northeast U.S. Shelf</u>: The effect of climate change on estuarine intertidal mud in the Northeast U.S. Shelf is expected to be primarily neutral (70%), with 30% of the experts' scores as negative).

<u>Climate Effects on Habitat Condition and Distribution</u>: Although the climate exposure of intertidal mud

habitats is expected to be very high, the sensitivity of these habitats to this exposure is less well known. The reasons for this low sensitivity are varied and include the general resilient nature of mud habitats and less understood dynamics related to the conversion of upland marsh habitats to mud flats with rising sea level. Sea level rise is known to create a cascade of habitat change by turning salt marsh into intertidal mud and intertidal mud into subtidal mud. Predicting the ultimate result of this change on the amount, range, and distribution, and connectivity of intertidal habitats will depend on the rate and magnitude of sea level rise and these habitat conversions. Additionally, while subtidal mud habitats may expand as sea level converts intertidal mud habitats to subtidal habitats (Vos and van Kesteren 2000), the nature of where intertidal habitats expand may be dependent on coastal development that is not anticipated to decline in the future. Therefore, the expert scores tend to reflect that the loss of marsh habitats due to sea level rise may expand intertidal mud habitats, but this conversion depends on coastal typography, tidal inundation, vegetation structure, and accretion rates (Thorne et al. 2015). The conversion of three dimensional structured habitats like salt marshes to mudflats may reduce biodiversity and biomass of estuarine associated species (Hosack et al. 2006), but may improve soft shell clamming, shorebird feeding, and nursery habitat for certain species.

Mollusks associated with estuarine intertidal mud habitats (e.g., soft-shell clams, hard clams, small crustaceans, and gastropods, are believed to be sensitive to ocean acidification (Gazeau et al. 2013). In general, larger negative effects are expected on the survival of larvae than adults (Kroeker et al. 2013). Ocean acidification is expected to reduce the shell size, thickness, calcification rates and growth, and survival of embryo and larval bivalves and gastropods (Gazeau et al. 2013). Some studies have shown additive and synergistic negative effects from ocean acidification and low dissolved oxygen on the growth, survival, and metamorphosis of larval bay scallop and hard clams (Kroeker et al. 2013; Griffith and Gobler 2017). Coastal waters are generally more susceptible to acidification than oceanic waters because they are subject to more acid sources and are generally less buffered than oceanic waters (i.e., differences in the amount of dissolved inorganic carbon, dissolved and particulate organic carbon, total alkalinity and nutrients from riverine and estuarine sources) (Waldbusser et al. 2015; Ekstrom et al. 2015; Gledhill et al. 2015).

However, the impacts of coastal acidification on these habitats will require significantly more research. While it is well known that acidification can impact bivalve larval development and growth by multiple modes of action (Waldbusser et al. 2015), the impact of acidification in the benthos is much more difficult to detect *in situ*. Climate induced expansion of green crabs (*Carcinus maenas*) into more mudflats in the Northeast U.S. has significantly increased the predation pressure on settling soft shell clams (McClenachan et al. 2015), making the detection of acidification effects on soft shell clams difficult.

Generally, ecological dependencies mediated by climate (e.g., invasive species and climate-migrations) will make direct correlations between biogeochemical dynamics, such as hypoxia and acidification, and important fisheries and protected species difficult to examine. Despite the complexity of these ecological interactions and the naturally high variability in abiotic conditions in intertidal habitats, background increases in temperature may change the species composition in these areas (e.g., green crab invasions) and pH and aragonite/calcite saturation states are expected to decrease due to combination of increased extreme precipitation events (i.e., causing large reductions in total alkalinity), high temperature induced increases in respiration, and decreases in pH in the oceanic source water due to atmospheric CO_2 absorption.

Habitat Summary: Intertidal mud habitats are important feeding and nursery habitat for shorebirds and estuarine dependent fishes. The role of these habitats in supporting migrant shorebirds and important fisheries in the Northeast U.S. will depend on how these habitats respond to a combination of coastal development and climate related factors such as sea level rise, precipitation, and coastal acidification.

Estuarine intertidal mud habitats are also exposed to coastal development and other anthropogenic stressors. Coastal population density and agriculture are associated with higher eutrophication and contamination, which

can disturb benthic habitat quality. According to the 2012 EPA Coastal Condition Report (EPA 2012), sediment quality varies throughout the region with the poorest sediment quality in Great Bay, NH; Narragansett Bay, RI; Long Island Sound, CT; the NY/NJ Harbor; the Upper Delaware Estuary; and the western tributaries of the Chesapeake Bay. Low sediment quality ratings were primarily driven by sediment contamination, which are mostly due to elevated levels of metals, polychlorinated biphenyls, and pesticides. Muddy sediments tend to provide greater surface area for sorption of both organic matter and the chemical pollutants that bind to organic matter. Aquaculture operations may also cause some disturbance to benthic habitats through the accumulation of nutrients, wastes, or sediment deposition, but good waterflow and husbandry limit this accumulation (ASMFC 2020).

Dredging, shoreline stabilization (e.g., riprap revetment, bulkheads, jetties, groins), and beach nourishment can alter the depth and sediment characteristics, with subsequent changes in infauna and epifauna/epiflora. All hardened shorelines have the potential to erode shallow, intertidal and subtidal mud bottom. Hardened shorelines have been shown to have lower abundance, biomass, and diversity of benthic prey and predators (Seitz et al. 2006; Morley et al. 2012) and can have higher incidence of marine exotic/invasive species compared to native material (Tyrrell and Byers 2007).

Finally, in contrast with subtidal mud, intertidal mud habitats may be more susceptible to habitat fragmentation and changes in its range and distribution. In some locations, sea level rise may create new intidal mud habitats while losing them in other locations. In highly fragmented intertidal habitats, recruitment of infauna can be compromised and significant reproductive effort can be lost into subtidal habitats that settling larvae are not adapted to thrive in. Future research in this area will benefit from detailed topography of intertidal and marsh areas that account for source and sink habitats under a range of sea level rise scenarios.

Literature Cited

Atlantic States Marine Fisheries Commission (ASMFC). 2020. Aquaculture: effects on fish habitat along the Atlantic Coast. Atlantic States Marine Fisheries Commission Habitat Management Series #16. http://www.asmfc.org/files/Habitat/HMS16_Aquaculture_May2020.pdf

Ekstrom JA, Suatoni L, Cooley SR, Pendleton LH, Waldbusser GG, Cinner JE, Ritter J, Langdon C, van Hooidonk R, Gledhill D, Wellman K, Beck MW, Brander LM, Rittschof D, Doherty C, Edwards PET, Portela R. 2015. Vulnerability and adaptation of US shellfisheries to ocean acidification. Nature Climate Change 5(3):207-14.

Gazeau F, Parker LM, Comeau S, Gattuso J-P, O'Connor WA, Martin S, Pörtner H-O, Ross PM. 2013. Impacts of ocean acidification on marine shelled molluscs. Marine Biology 160(8):2207-45.

Gledhill D, White M, Salisbury J, Thomas H, Misna I, Liebman M, Mook B, Grear J, Candelmo A, Chambers RC, Gobler C, Hunt C, King A, Price N, Signorini S, Stancioff E, Stymiest C, Wahle R, Waller J, Rebuck N, Wang Z, Capson T, Morrison JR, Cooley S, Doney S. 2015. Ocean and coastal acidification off New England and Nova Scotia. Oceanography 25(2):182-97.

Griffith AW, Gobler CJ. 2017. Transgenerational exposure of North Atlantic bivalves to ocean acidification renders offspring more vulnerable to low pH and additional stressors. Scientific Reports 7:11394.

Hosack, G. R., B. R. Dumbauld, J. L. Ruesink and D. A. Armstrong. 2006. Habitat associations of estuarine species: Comparisons of intertidal mudflat, seagrass (*Zostera marina*), and oyster (*Crassostrea gigas*)

habitats. Estuaries and Coasts 29(6):1150-60.

Kroeker KJ, Kordas RL, Crim R, Hendriks IE, Ramajo L, Singh GS, Duarte CM, Gattuso J-P. 2013. Impacts of ocean acidification on marine organisms: quantifying sensitivities and interaction with warming. Global Change Biology 19(6):1884-96.

McClenachan L, O'Connor G, Reynolds T. 2015. Adaptive capacity of co-management systems in the face of environmental change: the soft-shell clam fishery and invasive green crabs in Maine. Marine Policy 52:26-32.

Morley SA, Toft JD, Hanson KM. 2012. Ecological effects of shoreline armoring on intertidal habitats of a Puget Sound urban estuary. Estuaries and Coasts 35(3):774-84.

Seitz RD, Lipcius RN, Olmstead NH, Seebo MS, Lambert DM. 2006. Influence of shallow-water habitats and shoreline development on abundance, biomass, and diversity of benthic prey and predators in Chesapeake Bay. Marine Ecology Progress Series 326:11-27.

Thorne KM, Dugger BD, Buffington KJ, Freeman CM, Janousek CN, Powelson KW, Gutenspergen GR, Takekawa JY. 2015. Marshes to mudflats—effects of sea-level rise on tidal marshes along a latitudinal gradient in the Pacific Northwest. Open-File Report. Reston, VA.

Tyrrell MC, Byers JE. 2007. Do artificial substrates favor nonindigenous fouling species over native species? Journal of Experimental Marine Biology and Ecology 342(1):54-60.

U.S. Environmental Protection Agency (EPA). 2012. National Coastal Condition Report IV. EPA-R-842-10-003. US Environmental Protection Agency, Washington D.C.

Vos PC, van Kesteren WP. 2000. The long-term evolution of intertidal mudflats in the northern Netherlands during the Holocene; natural and anthropogenic processes. Continental Shelf Research 20(12):1687-710.

Waldbusser GG, Hales B, Langdon CJ, Haley BA, Schrader P, Brunner EL, Gray MW, Miller CA, Gimenez I, Hutchinsonet G. 2015. Ocean acidification has multiple modes of action on bivalve larvae. PLoS ONE 10(6): e0128376. https://doi.org/10.1371/journal.pone.0128376

Estuarine Intertidal Sand Bottom

System: Estuarine

Subsystem: Intertidal

Class: Unconsolidated Bottom

Sub-class: Sand

Geographic Area: Entire Area

Overall Vulnerability Rank = Moderate

Habitat Sensitivity = Low

Climate Exposure = Very High

	Estuarine Intertidal Sand Bottom	Attribute Mean	Data Quality	Distribution of Expert Scores
Sensitivity Attributes	Habitat condition	2.1	2.6	
	Habitat fragmentation	2	2	
	Distribution/Range	1.6	2.1	
	Mobility/Ability to spread or disperse	2	1.7	
	Resistance	2	2.4	
	Resilience	2.4	2.1	
	Sensitivity to changes in abiotic factors	1.9	2.3	
	Sensitivity and intensity of non−climate stressors	2.4	2.4	
	Dependency on critical ecological linkages	1.9	1.7	
	Sensitivity Component Score	Low		
Exposure Factors	Sea surface temp	n/a	n/a	
	Bottom temp	n/a	n/a	
	Air temp	4	3	
	River temp	n/a	n/a	
	Surface salinity	2.7	2.1	
	Bottom salinity	n/a	n/a	
	pH	4	2	
	Sea level rise	3.8	2.2	
	Precipitation	2.8	2.1	
	Floods	n/a	n/a	
	Droughts	n/a	n/a	
	Exposure Component Score	Very High		
**Overall Vulnerability Rank**		**Moderate**		

Low
Moderate
High
Very High

Habitat Name: Estuarine Intertidal Sand Bottom
System: Estuarine
Sub-System: Intertidal
Class: Unconsolidated Bottom
Sub-class: Sand
Geographic Area: Entire Area

Habitat Description: This sub-class includes estuarine intertidal sand bottom from mean high to mean low water lines, including the overtopping water column. This habitat includes the epifauna and infauna associated with estuarine sand bottom, such as non-reef-forming mollusks (e.g., soft-shell clams, hard clams, sea scallops, surf clams, ocean quahogs), marine worms, small crustaceans, gastropods, and polychaetes. This subclass excludes specific habitats identified elsewhere (i.e., non-calcareous algal bed, rooted vascular beds, and reef-forming mollusks, including blue mussels, eastern oysters).

Sandy intertidal habitats occur in estuaries throughout the study area. Intertidal habitats are limited in depth and extent by the tidal range, which is larger in New England, and the gradient of the shoreline, both of which are smaller in the Mid-Atlantic.

Overall Climate Vulnerability Rank: Moderate (97% certainty from bootstrap analysis).

Climate Exposure: Very High. Three exposure factors contributed to the Very High exposure score: Air Temperature (4.0), pH (4.0), and Sea Level Rise (SLR) (3.8). Air temperature, ocean acidification (decreasing pH), and SLR are all projected to increase significantly throughout the region. Precipitation (2.8) and Surface Salinity (2.7) also had fairly high scores. Extreme precipitation events are projected to increase in frequency and intensity throughout the region. Surface salinity is projected to increase significantly in Mid-Atlantic estuaries, where sand habitats are more common. The intertidal nature of this habitat places it at the nexus of significant atmospheric and oceanic change.

Habitat Sensitivity: Low. Most sensitivity attributes were in the Low and Moderate scoring bins. No sensitivity attributes scored above 2.4, with the highest scores for Sensitivity and Intensity of Non-Climate Stressors (2.4) and Resilience (2.4) followed by Habitat Condition (2.1).

Data Quality & Gaps: The data quality scores for the five exposure factors ranged from 2.0 (pH) to 3.0(Air Temperature). The relatively low score for Precipitation (2.1) may be due to uncertainty in the specific spatial nature of projected changes in extreme events. In addition, data quality is lower for estuarine habitats due to the low resolution of CMIP5 and ROMS-NWA projections for climate exposure in estuaries and nearshore, shallow coastal areas, which increases the uncertainty with overlap in climate exposure projections.

Three of the nine sensitivity attribute data quality scores were ≤2.0: Dependency on Critical Ecological Linkages (1.7), Mobility/Ability to Spread or Disperse (1.7), and Habitat Fragmentation (2.0). The relatively low data quality scores may reflect uncertainty in the sensitivities of biota associated with sand (i.e., infauna and epifauna) to climate change.

Positive or Negative Climate Effect in the Northeast U.S.: The effect of climate change on estuarine intertidal sand in the Northeast U.S. is expected to be neutral (85% of the experts' scores were neutral, 15% were negative).

Climate Effects on Habitat Condition and Distribution: Climate change is expected to have relatively minor impacts on this habitat. However, a number of lines of evidence suggest that an increased rate of sea level rise will cause a loss of sandy beaches due to submergence of the intertidal zone and coastal erosion.

Non-vegetated shorelines, including intertidal areas such as sheltered sand flats, sand in estuaries and sheltered lagoons are susceptible to erosion (Brown and McLachlan 2002). In addition, intertidal sand may be inundated if bulkheads and shoreline stabilization structures prevent their landward migration (Defeo et al. 2008).

Although the causes of sand shoreline erosion are complex and not all are related to climate change, the acceleration in SLR is expected to exacerbate beach erosion around the globe (Brown and McLachlan 2002; Nicholls et al. 2007; Chust et al. 2010). Estuarine intertidal sand habitats may experience some disturbance from storms, which are expected to increase in frequency and intensity in the Northeast region (USGCRP 2017). The effects of sea level rise will be exacerbated by extreme events produced by local storm surges, even if storm intensities do not increase in response to the warming of the oceans, and may accelerate the recession of sandy beaches (Brown and McLachlan 2002; Kennedy et al. 2002; Scavia et al. 2002; Church et al. 2008).

As the rate of SLR increases, natural and anthropogenic processes in estuaries and lagoons interact to act as a sand sink and raise bed elevations, potentially increasing erosion along the coast and drive major coastal instability in the vicinity of tidal inlets (Nicholls et al. 2007; FitzGerald et al. 2008). Gutierrez et al. (2007) predicted an increased likelihood of erosion and shoreline retreat for all types of mid-Atlantic coastal shorelines, including an increased likelihood for overwash and inlet breaching and the possibility of segmentation or disintegration of some barrier islands as the rate of SLR increases. In a SLR scenario assessment for the Chesapeake Bay, Glick et al. (2008) projected ocean and estuarine beaches would decline by 69% and 58%, respectively, under a 0.69-meter sea level rise by 2100. For a 1.5-meter SLR scenario, the impacts are more dramatic– virtually all the Chesapeake region's ocean beaches would disappear by 2100. Some loss of sandy intertidal habitats may be offset by beach nourishment and shoreline stabilization approaches engineered to protect sand habitats and property.

The infauna and epifauna associated with estuarine intertidal sandy habitats are more adapted to variability and extremes than those in the subtidal zone. However, some mollusks, particularly surf clams and ocean quahogs, are sensitive to increasing air and water temperatures. The distribution and landings of these species are declining in Mid-Atlantic over the past decade, while the New England populations have increased (MAFMC 2019; Lewis et al. 2001). Increasing water temperatures, high nutrient levels, and algal blooms can trigger reductions in dissolved oxygen in the nearshore water column and in sediments, which can have detrimental effects to infaunal and epifaunal species associated with sand habitats (Sharp et al. 1982; Brownlee et al. 2005).

Mollusks are also sensitive to ocean acidification (Gazeau et al. 2013). In general, larger negative effects are expected on the survival of larvae than adults (Kroeker et al. 2013). Ocean acidification is expected to reduce the shell size, thickness, calcification rates and growth, and survival of embryo and larval bivalves and gastropods (Gazeau et al. 2013). Some studies have shown additive and synergistic negative effects from ocean acidification and low dissolved oxygen on the growth, survival, and metamorphosis of larval bay scallop and hard clams (Kroeker et al. 2013; Griffith and Gobler 2017). Coastal waters are generally more susceptible to acidification than oceanic waters because they are subject to more acid sources and are generally less buffered than oceanic waters (i.e., differences in the amount of dissolved inorganic carbon, dissolved and particulate organic carbon, total alkalinity and nutrients from riverine and estuarine sources) (Waldbusser et al. 2011; Ekstrom et al. 2015; Gledhill et al. 2015).

Habitat Summary: According to the 2012 EPA Coastal Condition Report, sediment quality in the Northeast Coast region (Chesapeake Bay and north) is rated fair, with 12% of the coastal area in poor condition and 11% in fair condition, largely driven by sediment contamination from heavy metals, polychlorinated biphenyls, and pesticides, with poorer quality sediments near urban areas (EPA 2012). Southern New England

and Mid-Atlantic coastal areas have high contaminant loads due to legacy pollution from industrialization, and continued urbanization (EPA 2012). Coastal population density, development, and agriculture are all higher in the Mid-Atlantic and are associated with higher eutrophication. Benthic infauna and epifauna can be sensitive to contamination. However, coarse-grained sediments such as sand generally contain less total organic carbon (TOC) levels compared to soft-grained sediments. Because contaminants such as polyaromatic hydrocarbons, pesticides, and polychlorinated biphenyl are sequestered in the TOC fraction of sediments, sand sediments tend to be less impacted by contaminants than mud substrates (ICES 1992; Pierce 1994). Overall, further degradation of water and sediment quality is expected as urbanization and development in the coastal zone continues with increasing population growth.

Estuarine intertidal sand habitats are also exposed to anthropogenic disturbance. Associated infauna and epifauna are generally easily dispersed within and between individual estuaries, and have relatively rapid recovery rates due to short life spans, though this depends on the frequency of natural and anthropogenic disturbance. Channel dredging and shoreline alteration can convert intertidal sandy habitats to sub-tidal habitats or alter sediment characteristics (Johnson et al. 2008). Shoreline hardening with riprap revetment converts sand bottom to large diameter, engineered stone in the intertidal zone, and can increase shoreline erosion. Hardened shorelines have been shown to have lower abundance, biomass, and diversity of benthic prey and predators (Seitz et al. 2006; Morley et al. 2012). Other effects of engineered-shore structures include loss of sediment and reductions in beach volume and dimension (Kraus and McDougal 1996), loss of intertidal habitat and habitat fragmentation (Bozek and Burdick 2005; NRC 2007; Bulleri and Chapman 2010), and can have higher incidence of invasive species compared to native material (Tyrrell and Byers 2007; Pappal 2010; Geraldi et al. 2013). Titus et al. (2009) estimated that almost 60% of land within 1 m above the high tide line on the U.S. Atlantic coast (Florida to Massachusetts) is developed or expected to be developed.

Aquaculture operations may also cause some disturbance to benthic habitats through the accumulation of nutrients, wastes, or sediment deposition, but good water flow and husbandry limit this accumulation (ASMFC 2020). Sand sediments and associated infauna are sensitive to dredging impacts, although most species recovery relatively rapidly (Newell et al. 1998; Wilber et al. 2005). Shellfish generally have the capacity to rebound from poor growth conditions, if sufficient seed populations are present, and conditions for growth improve to favorable conditions. However, there is evidence that some of the changes occurring in the study area, such as increasing water temperatures, may prevent some species from rebounding (Lewis et al. 2001; MAFMC 2019), compounding the impacts of anthropogenic disturbance.

Intertidal sand infauna and epifauna are also sensitive to invasive species, including green crab (*Carcinus maenas*) which is believed to have been carried by ships in ballast water and sold as fish bait in much of the world. It now has established populations in New England and northern Mid-Atlantic in both subtidaland intertidal zones. It is a predator of many forms of shore life, including worms and mollusks (GISD 2020). In some areas (particularly New England), the crab's voracious appetite has affected the commercial shellfish industry (Webber 2013; Beal 2014).

Literature Cited

Atlantic States Marine Fisheries Commission (ASMFC). 2020. Aquaculture: effects on fish habitat along the Atlantic Coast. Atlantic States Marine Fisheries Commission Habitat Management Series #16. http://www.asmfc.org/files/Habitat/HMS16_Aquaculture_May2020.pdf

Beal BF. 2014. Final report: Green crab, *Carcinus maenas*, trapping studies in the Harraseeket River and manipulative field trials to determine effects of green crabs on the fate and growth of wild and cultured

individuals of soft-shell clams, *Mya arenaria* (May to November 2013). Downeast Institute for Applied Marine Research and Education, Beals, ME. 76 pp. (Accessed 30 Oct. 2020). https://downeastinstitute.org/wp-content/uploads/2018/08/1_24-final-report-freeport-shellfish-restoration-project-b.-beal.pdf.

Bozek CM, Burdick DM. 2005. Impacts of seawalls on saltmarsh plant communities in the Great Bay Estuary, New Hampshire USA. Wetlands Ecology and Management 13(5):553-68.

Brown AC, McLachlan A. 2002. Sandy shore ecosystems and the threats facing them: some predictions for the year 2025. Environmental Conservation 29(1):62-77.

Brownlee EF, Sellner SG, Sellner K.G. 2005. *Prorocentrum minimum* blooms: potential impacts on dissolved oxygen and Chesapeake Bay oyster settlement and growth. Harmful Algae 4 (3):593-602. https://doi.org/10.1016/j.hal.2004.08.009

Bulleri F, Chapman MG. 2010. The introduction of coastal infrastructure as a driver of change in marine environments. Journal of Applied Ecology 47(1):26-35.

Church JA, White NJ, Aarup T, Wilson WS, Woodworth PL, Domingues CM, Hunter JR, Lambeck K. 2008. Understanding global sea levels: past, present and future. Sustainability Science 3(1):9-22.

Chust G, Caballero A, Marcos M, Liria P, Hernández C, Borja Á. 2010. Regional scenarios of sea level rise and impacts on Basque (Bay of Biscay) coastal habitats, throughout the 21st century. Estuarine, Coastal and Shelf Science 87(1):113-24.

Defeo O, McLachlan A, Schoeman DS, Schlacher TA, Dugan J, Jones A, Lastra M, Scapini F. 2008.Threats to sandy beach ecosystems: a review. Estuarine, Coastal and Shelf Science 81(1):1-12.

Ekstrom JA, Suatoni L, Cooley SR, Pendleton LH, Waldbusser GG, Cinner JE, Ritter J, Langdon C, van Hooidonk R, Gledhill D, Wellman K, Beck MW, Brander LM, Rittschof D, Doherty C, Edwards PET, Portela R. 2015. Vulnerability and adaptation of US shellfisheries to ocean acidification. Nature Climate Change 5(3):207-14.

FitzGerald DM, Fenster MS, Argow BA, Buynevich IV. 2008. Coastal impacts due to sea-level rise. Annual Review of Earth and Planetary Sciences 36(1):601-47.

Gazeau F, Parker LM, Comeau S, Gattuso J-P, O'Connor WA, Martin S, Pörtner H-O, Ross PM. 2013. Impacts of ocean acidification on marine shelled molluscs. Marine Biology 160(8):2207-45.

Geraldi NR, Smyth AR, Piehler MF, Peterson CH. 2013. Artificial substrates enhance non-native macroalga and N_2 production. Biological Invasions 16(9):1819-31.

Gledhill D, White M, Salisbury J, Thomas H, Misna I, Liebman M, Mook B, Grear J, Candelmo A, Chambers RC, Gobler C, Hunt C, King A, Price N, Signorini S, Stancioff E, Stymiest C, Wahle R, Waller J, Rebuck N, Wang Z, Capson T, Morrison JR, Cooley S, Doney S. 2015. Ocean and coastal acidification off New England and Nova Scotia. Oceanography 25(2):182-97.

Global Invasive Species Database (GISD). 2020. [Internet]. Species profile: *Carcinus maenas*. (Accessed 08-03-2020). http://www.iucngisd.org/gisd/species.php?sc=114.

Griffith, A.W. and Gobler, C.J. 2017. Transgenerational exposure of North Atlantic bivalves to ocean acidification renders offspring more vulnerable to low pH and additional stressors. Scientific Reports 7:11394.

Gutierrez BT, Williams SJ, Thieler ER. 2007. Potential for shoreline changes due to sea-level rise along the U.S. Mid-Atlantic region. U.S. Geological Survey. Open-File Report 2007-1278. 1-25.

International Council for the Exploration of the Sea (ICES). 1992. Report of the ICES working group on the effects of extraction of marine sediments on fisheries. Copenhagen (Denmark): ICES Cooperative Research Report # 182.

Johnson MR, Boelke C, Chiarella LA, Colosi PD, Greene K, Lellis-Dibble K, Ludemann H, Ludwig M, McDermott S, Ortiz J, Rusanowsky D, Scott M, Smith J. 2008. Impacts to marine fisheries habitat from nonfishing activities in the northeastern United States. NOAA Technical Memorandum. National Oceanic and Atmospheric Administration, National Marine Fisheries Service. NMFS-NE-2O9. 1-328.

Kennedy VS, Twilley RR, Kleypas JA, Cowan JHJ, Hare SR. 2002. Coastal and marine ecosystems and global climate change: potential effects on U.S. resources. Pew Center on Global Climate Change. 1-51.

Kraus NC, McDougal WG. 1996. The effects of seawalls on the beach: Part 1, an updated literature review. Journal of Coastal Research 12(3):691-701.

Kroeker KJ, Kordas RL, Crim R, Hendriks IE, Ramajo L, Singh GS, Duarte CM, Gattuso J-P. 2013. Impacts of ocean acidification on marine organisms: quantifying sensitivities and interaction with warming. Global Change Biology 19(6):1884-96.

Glick P, Clough J, Nunley B. 2008. Sea-level rise and coastal habitats in the Chesapeake Bay region. Technical Report. National Wildlife Federation. 1-121.

Lewis CVW, Weinberg JR, Davis CS.. 2001. Population structure and recruitment of the bivalve *Arctica islandica* (Linnaeus, 1767) on Georges Bank from 1980-1999. Journal of Shellfish Research 20: 1135-44.

Mid-Atlantic Fishery Management Council (MAFMC). 2019. [Internet]. Ocean quahog fishery information document. April 1, 2019. (Accessed 23 November 2020). http://www.mafmc.org/surfclams-quahogs.

Morley SA, Toft JD, Hanson KM. 2012. Ecological effects of shoreline armoring on intertidal habitats of a Puget Sound urban estuary. Estuaries and Coasts 35(3):774-84.

National Research Council (NRC). 2007. Mitigating eroding sheltered shorelines: a trade-off in ecosystem services. Mitigating shore erosion along sheltered coasts. p. 78-97. Washington, DC: National Research Council.

Newell RC, Seiderer LJ, Hitchcock DR. 1998. The impact of dredging works in coastal waters: a review of the sensitivity to disturbance and subsequent recovery of biological resources on the sea bed. *In:* Ansell AD, Gibson RN, Barnes M, editors. Oceanography and Marine Biology: An Annual Review 36. Oban, Argyll (Scotland): UCL Press. 127-78.

Nicholls RJ, Wong PP, Burkett V, Codignotto J, Hay J, McLean R, Ragoonaden S, Woodroffe CD. 2007. Coastal systems and low-lying areas. Climate change 2007: impacts, adaptation and vulnerability.

Contribution of Working Group II to the Fourth Assessment Report of the Intergovernmental Panel on Climate Change. [Parry ML, Canziani OF, Palutikof JP, van der Linden PJ, Hanson CE (eds)]. Intergovernmental Panel on Climate Change. 315-56.

Pappal A. 2010. Marine invasive species. State of the Gulf of Maine Report. [Currier P, Gould D, Hertz L, Huston J, Tremblay ML (eds)]. The Gulf of Maine Council on the Marine Environment. 1-21.

Scavia D, Field JC, Boesch DF, Buddemeier RW, Burkett V, Cayan DR, Fogarty M, Harwell MA, Howarth RW, Mason C, Reed DJ, Royer TC, Sallenger AH, Titus JG. 2002. Climate change impacts on US coastal and marine ecosystems. Estuaries 25(2):149-64.

Seitz RD, Lipcius RN, Olmstead NH, Seebo MS, Lambert DM. 2006. Influence of shallow-water habitats and shoreline development on abundance, biomass, and diversity of benthic prey and predators in Chesapeake Bay. Marine Ecology Progress Series 326:11-27.

Sharp JH, Culberson CH, Church TM. 1982. The chemistry of the Delaware estuary. General considerations. Limnology and Oceanography 27. doi:10.4319/lo.1982.27.6.1015.

Titus JG, Hudgens DE, Trescott DL, Craghan M, Nuckols WH, Hershner CH, Kassakian JM, Linn CJ, Merritt PG, McCue TM, O'Connell JF, Tanski J, Wang J. 2009. State and local governments plan for development of most land vulnerable to rising sea level along the US Atlantic coast. Environmental Research Letters 4(4):1-7.

Tyrrell MC, Byers JE. 2007. Do artificial substrates favor nonindigenous fouling species over native species? Journal of Experimental Marine Biology and Ecology 342(1):54-60.

U.S. Environmental Protection Agency (EPA). 2012. National Coastal Condition Report IV. EPA-R-842-10-003. US Environmental Protection Agency, Washington D.C.

U.S. Global Change Research Program (USGCRP). 2017. Climate Science Special Report: Fourth National Climate Assessment. Volume I [Wuebbles, D.J., D.W. Fahey, K.A. Hibbard, D.J. Dokken, B.C. Stewart, and T.K. Maycock (eds.)]. U.S. Global Change Research Program. Washington, DC.USA. 470 pp. doi: 10.7930/J0J964J6.

Waldbusser GG, Voigt EP, Bergschneider H, Green MA, Newell RIE. 2011. Biocalcification in the eastern oyster (*Crassostrea virginica*) in relation to long-term trends in Chesapeake Bay pH. Estuaries and Coasts 34:221-31.

Webber MM. 2013. [Internet]. Results of the one-day green crab trapping survey conducted along the Maine coast from August 27 to 28, 2013. Maine Department of Marine Resources, Augusta, ME. 6 pp. (Accessed 30 Oct. 2020). https://www.maine.gov/dmr/science-research/species/invasives/greencrabs/documents/trapsurveyrpt2013.pdf.

Wilber DH, Brostoff W, Clarke DG, Ray GL. 2005. Sedimentation: Potential biological effects from dredging operations in estuarine and marine environments. DOER Technical Notes Collection (ERDC TN-DOER-E20). U.S. Army Engineer Research and Development Center, Vicksburg, MS.

Estuarine Kelp

System: Estuarine

Subsystem: Subtidal

Class: Aquatic Bed

Sub-class: Kelp

Geographic Area: Entire Area

Overall Vulnerability Rank = High

Habitat Sensitivity = High

Climate Exposure = High

	Estuarine Kelp	Attribute Mean	Data Quality	Distribution of Expert Scores
Sensitivity Attributes	Habitat condition	3.2	2.2	
	Habitat fragmentation	3.2	2.2	
	Distribution/Range	3.2	2.2	
	Mobility/Ability to spread or disperse	2.8	2.2	
	Resistance	3.2	2.2	
	Resilience	3.2	2.2	
	Sensitivity to changes in abiotic factors	3.4	2.2	
	Sensitivity and intensity of non−climate stressors	3.4	2.2	
	Dependency on critical ecological linkages	3.4	2	
	Sensitivity Component Score	High		
Exposure Factors	Sea surface temp	4	2.5	
	Bottom temp	n/a	n/a	
	Air temp	n/a	n/a	
	River temp	n/a	n/a	
	Surface salinity	1.9	2.1	
	Bottom salinity	n/a	n/a	
	pH	4	2	
	Sea level rise	2.4	2.2	
	Precipitation	n/a	n/a	
	Floods	n/a	n/a	
	Droughts	n/a	n/a	
	Exposure Component Score	High		
**Overall Vulnerability Rank**		**High**		

Low
Moderate
High
Very High

Habitat Name: Estuarine Kelp

System**:** Estuarine

Subsystem: Subtidal

Class: Aquatic Bed

Sub-class: Kelp

Geographic Area: Entire Area

Habitat Description: This sub-class includes kelp, consisting of non-rooted, brown algae of the order Laminariales, and are important components of nearshore, subtidal benthic communities. Kelp can form dense beds on rocky bottom habitat in the photic zone to depths of 10–25 m (Merzouk and Johnson 2011). Lopez et al. (2014) reported few kelp occur deeper than 5 m in Long Island Sound. Suitable habitat for kelp is associated with areas of rocky bottom and well mixed waters. The two largest brown algae species in the U.S. are sugar kelp (*Saccharina latissima*) and horsetail kelp (*Laminaria digitata*), although shotgun kelp (*Agarum clathratum*) and winged kelp (*Alaria esculenta*) are also prevalent in thestudy area. The historic U.S. range is Gulf of Maine to Long Island Sound, although the densities of both species in Long Island Sound are substantially reduced (Van Patton and Yarish 2009; Merzouk and Johnson 2011; Lopez et al. 2014; Wilson et al. 2019; Auster P, pers. comm., 4 June 2020). This habitat subclass also includes aquaculture (i.e., kelp farming). Commercial aquaculture of macroalgae is predominantly for rockweed (*Ascophyllum nodosum*) (included in the non-kelp macroalgae habitat narrative), although *S. latissima* and *L. digitata* are also cultured.

Overall Climate Vulnerability Rank: High (99% certainty from bootstrap analysis).

Climate Exposure: High. The overall High exposure score was influenced by two Very High attribute means: Sea Surface Temperature and pH (4.0 for both). The exposure attribute scores for Surface Salinity and Sea Level Rise (SLR) were 1.9 and 2.4, respectively. The current geographic range of kelp in the study area include Long Island Sound and the Gulf of Maine, where sea surface temperature is projected to have the greatest change. Although the projected change in pH for the Gulf of Maine is less than southern New England and the Mid-Atlantic, it is still expected to drastically decrease from historic levels.

Habitat Sensitivity: High. Eight of the nine sensitivity attribute means were ≥3.2, while the attribute mean for Mobility/Ability to Spread or Disperse was also relatively high (2.8). The three attributes with the highest sensitivity means were Sensitivity to changes in abiotic factors, Sensitivity and Intensity of Non−Climate Stressors, and Dependency on Critical Ecological Linkages.

Data Quality & Gaps: The data quality scores for three of the four climate exposure factors were relatively Low (≤ 2.2), while the highest (Sea Surface Temperature) was 2.5. For pH and Sea Surface Temperature, this likely is attributed to the low resolution of ROMS-NWA projections for climate exposure in estuaries and nearshore, shallow coastal areas. In addition, because comprehensive mapping and baseline data for kelp in the study area is lacking, the spatial distribution of kelp used forthe climate exposure scoring was based on text descriptions only.

For habitat sensitivity, data quality for all of the nine attributes were scored relatively low (2.2 and 2.0).This may reflect the lack of comprehensive assessments for kelp in the study area.

Positive or Negative Climate Effect for the Northeast U.S.: The effect of climate change on marine kelp in the Northeast U.S. is expected to be negative (95% of the experts' scores were negative).

Climate Effects on Habitat Condition and Distribution: Higher mean temperatures and heat waves have been attributed to reductions in abundance and range of kelp in the Gulf of Maine (Krumhansl et al. 2016; Witman and Lamb 2018) and southern New England (Lopez et al. 2014; Feehan et al. 2019), and climate projections indicate these trends will continue. The projected northward shift of the trailing edge of kelp species in the Northwestern Atlantic is attributed to reductions in growth and complete mortality, leading to a

36% reduction of *L. digitata* and 21% of *S. latissima* habitats for the end-of-century time frame under the RCP8.5 scenario (Wilson et al. 2019). Feehan et al. (2019) documented the decline of *S. latissima* and its replacement by turf-forming macroalgae in Narragansett Bay, Rhode Island, between 1980 and 2018, which they attributed to increasing water temperature. Turf algae generally refers to low-lying (<10 cm tall) species with densely packed fronds, and lax and filamentous branches (e.g., *Cladophora, Ulva, and Polysiphonia*) (Connell et al. 2014).

Feehan et al. (2019) reported kelp attached to turf algae required significantly less force to detach from the substrate, and a pattern of lower survival following major storm events compared to rock-attached kelp. Witman and Lamb (2018) also reported higher kelp mortality with more storm disturbance, and suggested increasing wave disturbance from climate change, as well as warmer temperatures, may also contribute to the future loss of kelp foundation species. Storms are expected to increase in frequency and intensity in the Northeast region (USGCRP 2017), and increases in wave energy exposure could have deleterious effects on shallow-water habitats. Based on the results of CMIP5 RCP8.5, Lehmann et al. (2014) concluded that the northeast region would likely experience an increase in the frequency and intensity of winter extratropical cyclone events, but a slight decrease in summer events, by 2100. Colle et al. (2013) projected extratropical cyclones may become more intense (10-40%) along the northeast coast, especially during the mid-21st century as a result of an increase in latent heat release due to a moister atmosphere. The northeast region is also affected by tropical cyclone systems that originate in the Atlantic and Caribbean basins. Increases in sea surface temperatures may increase the maximum potential intensity of tropical cyclones and should be reflected by an increase in the frequency of the strongest hurricanes (Trenberth 2005; Kossin et al. 2007; Knutson et al. 2010). Between the 1970s and early 2000s, the number of major hurricanes (Category 4 and 5) in an average year approximately doubled (Emanuel 2005; Webster et al. 2005).

The projected change in pH for marine macroalgae species in the study area is very high. However, kelp are fleshy, non-calcifying algae, and are believed to have low sensitivities to pH and carbonate chemistry (Koch et al. 2013; Kroeker et al. 2013). Marine macroalgae species appear to have higher growth rates under experimental elevated pCO_2 conditions through higher photosynthetic and growth rates (Gledhill et al. 2015; Young and Gobler 2016), which could mitigate some climate-related impacts associated withwarming waters.

Habitat Summary: Kelp are increasingly threatened by a variety of non-climate local stressors including overfishing, pollution, disease, herbivory, storms, and warming waters, and regional variation of these drivers may affect kelp populations (Steneck et al. 2002). Sea urchins are a natural predator of large brown algal species, including kelp, and in some areas urchins have decimated kelp beds (i.e., "urchin barrens"). There is evidence that overfishing of top predators may be a contributing factor to explosions of urchin populations (Steneck et al. 2002).

Wilson et al. (2019) suggested potential climate-induced shifts in dominance from native kelp species to invasive species, such as green algae (*Codium fragile*), and a loss of kelp may facilitate the transition to aturf algae dominated ecosystem. Ecosystem changes observed throughout the study area (e.g., warmingwaters, increasing intensity, frequency, and duration of coastal storms, increasing prevalence of invasive species and herbivory, and exposure to stormwater pollution) have been attributed to patterns of long-term shifts from kelp- to turf-dominated habitats in New England and the Gulf of Maine (Steneck etal. 2002; Dijkstra et al. 2017a; Dijkstra et al. 2017b; Filbee-Dexter & Wernberg 2018; Dijkstra et al. 2019;Feehan et al. 2019).

Warming ocean waters have been shown to eliminate thermal barriers that historically limit reproductive success of marine invasive species (e.g., the tunicate *Botrylloides violaceus*), which may impact kelp abundance and distribution (Dijkstra et al. 2017a). Increased reproduction of marine invasive species, combined with limited biological resistance in regions with cooler water temperatures, may lead to a community state change. Marine invasive species, likely introduced via maritime transport vectors, are known to compete for space and foul benthic substrates (Pappal 2010) and may replace kelp species in portions of the study area (Scheibling and Gagnon 2006; Trott and Entreline 2019). *S. latissima* underwent a significant 36.2% decrease in abundance between 1987 and 2015 on Cashes Ledge, concurrent with a rapid warming of

the GOM and invasion by the kelp-encrusting bryozoan *Membranipora membranacea* (Scheibling and Gagnon 2006; Witman and Lamb 2018).

Kelp requires moderate nutrient levels and well-mixed waters for optimal growth, as well as exposed rocky bottom (Steneck et al. 2002). Kelp abundance decreases in very wave sheltered areas (Bekkby etal. 2019), and offshore densities of *S. latissima* were over 150 times greater than at coastal sites with similar but lower magnitude trends for congeneric *S. digitata* (Witman and Lamb 2018). This suggests kelp in marine waters may be more resilient to change compared to kelp in estuarine waters. In addition, kelp in estuarine waters may have greater vulnerabilities because of their higher exposure to anthropogenic impacts, such as storm water pollution, dredging and filling, higher turbidity, and higherwater temperature and eutrophication due to shallower water and partial enclosure of estuaries.

Kelp requires rock bottom habitat to attach to the substrate, so placement of revetments, jetties, groins,and other structures in estuarine waters may increase habitat availability if other habitat requirements are met. Although commercial and recreational harvest and culturing of kelp and rockweed occurs in Maine, there is little information available suggesting widespread impacts associated with controlled harvesting. However, at least one study (Wilson et al. 2019) projects the geographic range of kelp will shift northward and contract in the study area (i.e., 36% reduction of *L. digitata* and 21% of *S. latissima* by the end of the century under the RCP8.5 scenario). Future harvests of these species may be problematic as abundance and distribution declines.

Literature Cited

Bekkby T, Smit C, Gundersen H, Rinde E, Steen H, Tveiten L, Gitmark JK, Fredriksen S, Albretsen J and Christie H. 2019. The abundance of kelp Is modified by the combined impact of depth, waves and currents. Frontiers of Marine Science 6:475. doi: 10.3389/fmars.2019.00475.

Colle BA, Zhang Z, Lombardo KA, Chang E, Liu P, Zhang M. 2013. Historical evaluation and future prediction of eastern North American and western Atlantic extratropical cyclones in the CMIP5 models during the cool season. Journal of Climate 26(18):6882-903.

Connell SD, Foster MS, Airoldi L. 2014. What are algal turfs? Towards a better description of turfs. Marine Ecology Progress Series 495:299–307.

Dijkstra JA, Westerman EL, Harris LG. 2017a. Elevated seasonal temperatures eliminate thermal barriers of reproduction of a dominant invasive species: A community state change for northern communities? Diversity and Distributions 23:1182–92.

Dijkstra JA, Harris LG, Mello K, Litterer A, Wells C, Ware C. 2017b. Invasive seaweeds transform habitat structure and increase biodiversity of associated species. Journal of Ecology. 10.1111/1365-2745.12775.

Dijkstra JA, Litterer A, Mello K, O'Brien BS, Rzhanov Y. 2019. Temperature, phenology, and turf macroalgae drive seascape change: Connections to mid-trophic level species. Ecosphere 10(11):e02923. https://doi.org/10.1002/ecs2.2923.

Emanuel K. 2005. Increasing destructiveness of tropical cyclones over the past 30 years. Nature 436(7051):686-8.

Feehan CJ, Grace SP, Narvaez CA. 2019. Ecological feedbacks stabilize a turf-dominated ecosystem at the southern extent of kelp forests in the Northwest Atlantic. Scientific Reports 9:7078. https://doi.org/10.1038/s41598-019-43536-5.

Filbee-Dexter K, Wernberg T. 2018. Rise of turfs: A new battlefront for globally declining kelp forests. Bio-Science 68:64–76. https://doi.org/10.1093/biosci/bix147.

Gledhill DK, White MM, Salisbury J, Thomas H, Misna I, Liebman M, Mook B, Grear J, et al. 2015. Ocean and coastal acidification off New England and Nova Scotia. Oceanography 28(2):182–97. http://dx.doi.org/10.5670/oceanog.2015.41.

Knutson TR, McBride JL, Chan J, Emanuel K, Holland G, Landsea C, Held I, Kossin JP, Srivastava AK, Sugi M. 2010. Tropical cyclones and climate change. Nature Geoscience 3(3):157-63.

Koch M, Bowes G, Ross C, Zhang X-H. 2013. Climate change and ocean acidification effects on seagrasses and marine macroalgae. Global Change Biology 19(1):103-32.

Kossin JP, Knapp KR, Vimont DJ, Murnane RJ, Harper BA. 2007. A globally consistent reanalysis of hurricane variability and trends. Geophysical Research Letters 34(4):1-6.

Kroeker KJ, Kordas RL, Crim R, Hendriks IE, Ramajo L, Singh GS, Duarte CM, Gattuso J-P. 2013. Impacts of ocean acidification on marine organisms: quantifying sensitivities and interaction with warming. Global Change Biology 19(6):1884-96.

Krumhansl KA, Okamoto DK, Rassweiler A, Novak M, Bolton JJ, Cavanaugh KC, Connell SD, Johnson CR et al. 2016. Global patterns of kelp forest change over the past half-century. Proceedings of the National Academy of Sciences 113(48):13785-90. https://doi.org/10.1073/pnas.1606102113.

Lehmann J, Coumou D, Frieler K, Eliseev AV, Levermann A. 2014. Future changes in extratropical storm tracks and baroclinicity under climate change. Environmental Research Letters 9:8.

Lopez G, Carey D, Carlton JT, Cerrato R, Dam H, DiGiovanni R, Elphick C, Frisk M, Gobler C, Hice L, Howell P, Jordaan A, Lin S, Liu S, Lonsdale D, McEnroe M, McKown K, McManus G, Orson R, Peterson B, Pickerell C, Rozsa R, Shumway SE, Siuda A, Streich K, Talmage S, Taylor G, Thomas E, Van Patten M, Vaudrey J, Yarish C, Wikfors G, Zajac R. 2014. Biology and ecology of Long Island sound. *In:* Latimer J,Tedesco M, Swanson R, Yarish C, Stacey P, Garza C. (eds) Long Island Sound. Springer Series on Environmental Management. Springer, New York, NY. https://doi.org/10.1007/978-1-4614-6126-5_6

Merzouk A, Johnson LE. 2011. Kelp distribution in the northwest Atlantic Ocean under a changing climate. Journal of Experimental Marine Biology and Ecology 400(1-2):90-8. https://doi.org/10.1016/j.jembe.2011.02.020.

Pappal A. 2010. Marine invasive species. State of the Gulf of Maine Report. [Currier P, Gould D, Hertz L, Huston J, Tremblay ML (eds)]. The Gulf of Maine Council on the Marine Environment. 1-21.

Scheibling RE, Gagnon P. 2006. Competitive interactions between the invasive green alga *Codium fragile* ssp. *tomentosoides* and native canopy-forming seaweeds in Nova Scotia (Canada). Marine Ecology Progress Series 325:1-14. DOI: 10.3354/meps325001.

Steneck R, Graham MH, Bourque BJ, Corbett D, Erlandson JM, Estes JA, Tegner MJ. 2002. Kelp forest ecosystems: biodiversity, stability, resilience and future. Marine Sciences Faculty Scholarship. 65. https://digitalcommons.library.umaine.edu/sms_facpub/65.

Trenberth K. 2005. Uncertainty in hurricanes and global warming. Science 308(5729):1753-4.

Trott TJ, Enterline C. 2019. First record of the encrusting bryozoan *Cribrilina* (*Juxtacribrilina*) *mutabilis* (Ito, Onishi and Dick, 2015) in the Northwest Atlantic Ocean. BioInvasions Records 8.

U.S. Global Change Research Program (USGCRP). 2017. Climate Science Special Report: Fourth National Climate Assessment. Volume I. [Wuebbles, D.J., D.W. Fahey, K.A. Hibbard, D.J. Dokken, B.C. Stewart, and T.K. Maycock (eds.)]. U.S. Global Change Research Program. Washington, DC. USA. 470 pp. doi: 10.7930/J0J964J6.

Van Patten MS, Yarish C. 2009. Seaweeds of Long Island Sound. Bulletin 39. Connecticut College Arboretum.

Webster PJ, Holland GJ, Curry JA, Chang H-R. 2005. Changes in tropical cyclone number, duration, and intensity in a warming environment. Science 309(5742):1844-6.

Wilson KL, Skinner MA, Lotze HK. 2019. Projected 21st-century distribution of canopy-forming seaweeds in the Northwest Atlantic with climate change. Diversity and Distributions 25: 582-602. https://doi.org/10.1111/ddi.12897.

Witman JD, Lamb RW. 2018. Persistent differences between coastal and offshore kelp forest communities in a warming Gulf of Maine. PLoS ONE 13(1):e0189388. https://doi.org/.10.1371/journal.pone.0189388.

Young CS, Gobler CJ. 2016. Ocean acidification accelerates the growth of two bloom-forming macroalgae. PLoS ONE 11(5):e0155152. doi:10.1371/journal.pone.0155152.

Estuarine Red, Green, Small-brown Algae

System: Estuarine

Subsystem: Subtidal & Intertidal

Class: Aquatic Bed

Sub-class: Red, Green, Small-brown Algae

Geographic Area: Entire Area

Overall Vulnerability Rank = Low

Habitat Sensitivity = Low

Climate Exposure = High

	**Estuarine Red, Green, Small-brown Algae**	Attribute Mean	Data Quality	Distribution of Expert Scores
Sensitivity Attributes	Habitat condition	2	2	
	Habitat fragmentation	1.9	2	
	Distribution/Range	2	2	
	Mobility/Ability to spread or disperse	1.9	2	
	Resistance	1.8	1.8	
	Resilience	1.7	1.8	
	Sensitivity to changes in abiotic factors	2.1	1.8	
	Sensitivity and intensity of non−climate stressors	2.5	1.8	
	Dependency on critical ecological linkages	2.1	1.8	
	Sensitivity Component Score	Low		
Exposure Factors	Sea surface temp	3.7	2.5	
	Bottom temp	n/a	n/a	
	Air temp	n/a	n/a	
	River temp	n/a	n/a	
	Surface salinity	2.4	2.1	
	Bottom salinity	n/a	n/a	
	pH	4	2	
	Sea level rise	2.8	2.2	
	Precipitation	2.7	2.1	
	Floods	n/a	n/a	
	Droughts	n/a	n/a	
	Exposure Component Score	High		
	**Overall Vulnerability Rank**	**Low**		

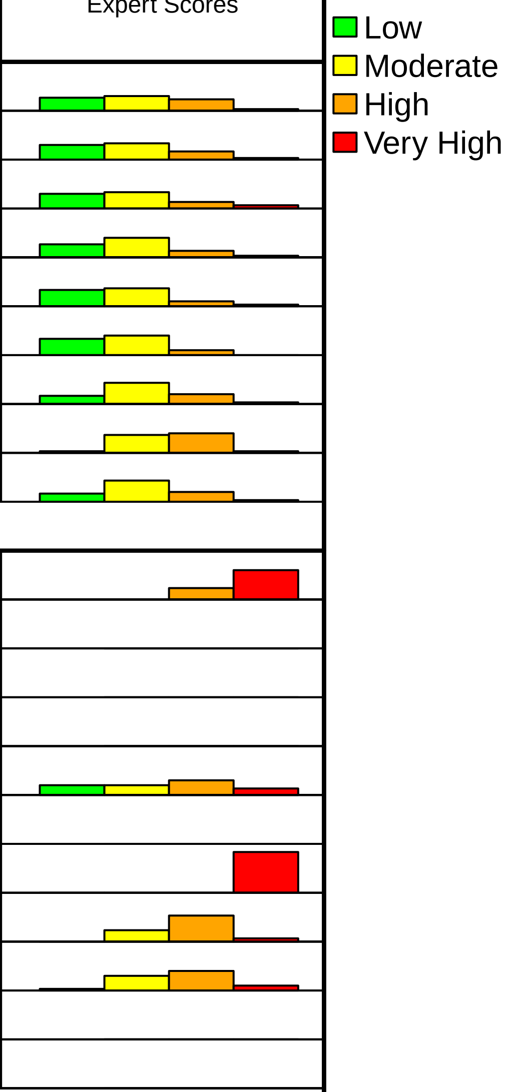

Habitat Name: Estuarine Red, Green, Small-brown Algae

System: Estuarine

Subsystem: Subtidal and Intertidal

Class: Aquatic Bed

Sub-class: Red, Green, Small-brown Algae

Geographic Area: Entire Area

<u>Habitat Description</u>: Estuarine red, green, and small brown (non-kelp) algal species are non-rooted, benthic macrophytes occurring in both subtidal and intertidal zones. Red, green, and small brown algae photosynthesize, so are limited to the photic zone. Red, green, and brown algae are distributed across the entire study area and the subclass contains a number of species, although there is considerable geographic variability among the species related to temperature tolerances and ecological requirements. A total of 316 macroalgae taxa have been identified from four Gulf of Maine embayments, including 81 green, 111 brown, and 124 red algae (Mathieson et al. 2010). Van Patton and Yarrish (2009) estimated 250 species of macroalgae in the Long Island Sound, and Orris (1980) listed 62 species from Maryland waters of the Chesapeake Bay (note the Gulf of Maine and Long Island Sound assessments include species of kelp).

Most species of red, green, and small brown algae that occur in the intertidal zone are restricted to lower and middle elevations due to their sensitivities to dessication, although green algae can occur from the uppermost part of the intertidal zone to the mid-intertidal zone (Van Patton and Yarish 2009). Red algae can absorb green and blue light, thus are capable of growing in greater depths of the subtidal zone. The red algae group contains many species and its morphologies can take the form of crusts, filamentous and branching, blades and sheets. There are also species of calcifying coralline algae, which are evaluated in the narrative for marine rocky bottom habitat. Commercial aquaculture of non-kelp macroalgae is predominantly for rockweed, *Ascophyllum nodosum*.

Examples of some of the species for this subclass include:
Small brown (non-kelp) algae: rockweeds *Ascophyllum nodosum* and *Fucus* spp., Sargasso weed *Sargassum filipendula*;
Red algae: Irish moss *Chondrus crispus, Gracilaria* spp., dulse *Palmaria palmata*, nori *Porphyra* spp., *Phyllophora pseudoceranoides,* Polly *Polysiphonia* spp.;
Green algae: sea lettuce *Ulva lactuca*, *Cladophora* spp, dead man's fingers *Codium fragile*.

Twenty taxa of non-indigenous algal species are known from the Northwest Atlantic, including two subspecies of the green alga *Codium fragile*, four brown algae, and fourteen red algae (Mathieson et al.2008). Several species of introduced macroalgae found in the study area are also considered invasive and nuisance species, such as *Dasysiphonia japonica*, *C. fragile*, *Polysiphonia harveyi, Grateloupia turuturu, Gracilaria vermiculophylla,* and several species of *Porphyra* (Mathieson et al. 2008; Witman and Lamb 2018). These species are often more tolerant of anthropogenic impacts, in particular warming waters, and in some cases compete with indigenous algal and other benthic species for space and resources (Scheibling and Gagnon 2006; Trott and Entreline 2019).

<u>Overall Climate Vulnerability Rank</u>: Low (99% certainty from bootstrap analysis).

<u>Climate Exposure</u>: High. The overall High exposure score was influenced by Sea Surface Temperature (3.7) and pH (4.0), with Sea Level Rise (SLR) (2.8), Precipitation (2.7), and Surface Salinity (2.4) scoring Moderate to High. Changes to sea surface temperature, pH, and SLR in the estuarine photic zone where algal species occur is projected to be high or very high throughout the study area.

<u>Habitat Sensitivity</u>: Low. Five of the nine sensitivity attributes mean scores were ≥2.0. Sensitivity and Intensity of Non−Climate Stressors had the highest mean (2.5), and four other attribute means were 2.0 or 2.1 (Habitat Condition, Distribution/Range, Sensitivity to Changes in Abiotic Factors, Dependency on Critical

Ecological Linkages). This suggests a moderate sensitivity to climate change for most small brown, red, and green algal populations. The high mean for Sensitivity and Intensity of Non-Climate Stressors is likely a result of moderate to high anthropogenic stressors in many estuaries in the study area.

The tolerance of the many species of non-indigenous macroalgae species to warming waters and anthropogenic effects likely contributes to the low sensitivity means for some of the sensitivity attributes in this subclass. Although the overall climate sensitivity was considered Low for non-kelp macroalgae in both estuarine and marine systems, it should be noted that the distribution of scores were distinct between the two systems. There were more scores across all of the attributes in the High bin for the estuarine system than the marine system (50 vs 16), and there were more scores in the Low bin for the marine system compared to the estuarine system (112 vs 65). Although some of these differences could be attributed to having different scorers in the two systems, the differences may reflect an understanding that macroalgae in the estuarine system are exposed to higher intensity and frequency of anthropogenic stressors than in the marine system. This could result in estuarine macroalgae being more sensitive to climate change than marine macroalgae.

Data Quality & Gaps: The data quality scores for Sea Surface Temperature was relatively High (2.5), while the others were 2.1 or 2.2. The Low data quality score for pH (2.0) was likely attributed to the low resolution of CMIP5 projections for nearshore, shallow areas. Surface Salinity data quality was scored low (2.1), which likely reflects the large range of projected change in estuarine salinities over the study area. SLR and Precipitation scored Low (2.2 and 2.1, respectively), but this may be attributed tothe uncertainty of sea level rise exposure and the influence of precipitation over the wide vertical distribution of macroalgae.

For habitat sensitivity, all of the attributes were scored relatively Low (2.0 or 1.8). Much of the low data quality scoring for sensitivity may be attributed to the abundant and diverse taxa and ecology of this subclass. Algal species are found throughout the intertidal zone to the subtidal photic zone limits across the entire study area. In addition, a number of species are non-indigenous and invasive, and tolerant of warmer water and other perturbations, and may flourish under future climate conditions.

Positive or Negative Climate Effect for the Northeast U.S.: The expected effect of climate change on marine red, green, and small brown algae in the Northeast U.S. was mostly neutral (65%). Positive and negative effect scores were split at 20% and 15%, respectively. As discussed above, this wide distribution of climate directionality is likely a reflection of the taxonomic and ecological diversity of the group as well as the prevalence of non-indigenous macroalgae species that are more resilient to perturbations than native species.

Climate Effects on Habitat Condition and Distribution: Using a species distribution model, Wilson et al. (2019) projected climate-induced shifts in dominance from native canopy-forming macroalgae to turf-algae by the end of the century. Turf algae generally refers to low-lying (<10 cm tall) species with densely packed fronds, lax and filamentous branches (e.g., *Cladophora, Ulva, and Polysiphonia*) (Connellet al. 2014).

Primarily driven by increased sea surface temperature, the model projected northern range shifts for rockweed *A. nodosum*, the fucoid algae *Fucus vesiculosus,* Irish moss *C. crispus*, and the invasive green algae *Codium fragile*. The current southern range extent for *A. nodosum* and *C. crispus* is Long Island Sound, while *F. vesiculosus* and *C. fragile* occur throughout the entire study area. Wilson et al. (2019) projected the trailing edge of *F. vesiculosus, A. nodosum,* and *C. crispus* will shift northward under RCP8.5. The model projection for *A. nodosum* effectively results in the species to be extirpated from the entire study area, and the trailing edge for *C. crispus* and *F. vesiculosus* would no longer occur in waters south of the Gulf of Maine and Long Island, respectively. However, the overall habitat of all three species would expand into waters north of the Gulf of Maine. Interestingly, the species distribution model projected no shift in the trailing edge of the invasive *C. fragile*, and minimal northward range expansion, and an overall habitat contraction of 2% (Wilson et al. 2019).

Macroalgal species that occur in the intertidal zone are also sensitive to air temperature and desiccation.

Although changes in air temperature were not evaluated in the climate exposure scoring for this sub-class, the projected change compared to historic periods under RCP8.5 was very high. Increasing air temperature may result in vertical distribution shifts in intertidal macroalgae to minimize thermal or desiccation stress during low tide (Harley et al. 2012).

Sea level rise exposure for this sub-class is Moderate to High, although the effect of rising sea levels for macroalgal species is dependent upon their vertical distribution in the water column. In this regard, species in the intertidal and the shallowest subtidal zones will be most affected by changes in sea level. Because macroalgae require some minimal threshold of light for photosynthesis, they are expected to shift distribution landward to maintain exposure to sunlight (Steneck et al. 2002). However, the availability of suitable substrate, and both natural and artificial barriers, may restrict landward migration of macroalgae.

Storms are expected to increase in frequency and intensity in the Northeast region (USGCRP 2017), and increases in wave energy exposure could have deleterious effects on intertidal and shallow-water habitats. Based on the results of CMIP5 RCP8.5, Lehmann et al. (2014) concluded that the northeast region would likely experience an increase in the frequency and intensity of winter extratropical cyclone events, but a slight decrease in summer events, by 2100. Colle et al. (2013) projected extratropical cyclones may become more intense (10-40%) along the northeast coast, especially during the mid-21st century as a result of an increase in latent heat release due to a moister atmosphere. The northeast region is also affected by tropical cyclone systems that originate in the Atlantic and Caribbean basins.

Increases in sea surface temperatures may increase the maximum potential intensity of tropical cyclones and should be reflected by an increase in the frequency of the strongest hurricanes (Trenberth 2005; Kossin et al. 2007; Knutson et al. 2010). Between the 1970s and early 2000s, the number of major hurricanes (Category 4 and 5) in an average year approximately doubled (Emanuel 2005; Webster et al. 2005).

The projected change in pH for marine macroalgae species in the study area is Very High. The majority of species in the study area are fleshy, non-calcifying algae, and are believed to have low sensitivities to pH and carbonate chemistry (Koch et al. 2013; Kroeker et al. 2013). However, coralline red algae and calcifying green algae occur in the study area, and some species have shown reduced calcification rates in high-CO_2 mesocosm experiments (Gao et al. 1993; Kroeker et al. 2013). This group of algal species are discussed under the marine and estuarine rocky bottom habitat subclasses. Some marine macroalgae have shown higher growth rates under experimental elevated pCO_2 conditions through higher photosynthetic and growth rates (Gledhill et al. 2015; Young and Gobler 2016), which could mitigate some climate-related impacts associated with warming waters.

Habitat Summary: The effects of warming ocean waters and other environmental changes will likely result in “winners and losers” for this habitat subclass. Many species of macroalgae can tolerate temperature gradients of several degrees and display ecotypes that are capable of growing and reproducing over a wide range of physical and chemical conditions. Some of these species are characterized as “turf” algae, including *Cladophora, Ulva, and Polysiphonia* (Connell et al. 2014).

Ecosystem changes observed throughout the study area (e.g., warming waters, increasing intensity, frequency, and duration of coastal storms, increasing prevalence of invasive species and herbivory, and exposure to stormwater pollution) have been attributed to patterns of long-term shifts from kelp- to turf-dominated habitats in New England and the Gulf of Maine (Steneck et al. 2002; Dijkstra et al. 2017a;Dijkstra et al. 2017b; Filbee-Dexter and Wernberg 2018; Dijkstra et al. 2019; Feehan et al. 2019).

Increased reproduction of marine invasive species, combined with limited biological resistance in regions with cooler water temperatures, may lead to community state changes in shallow habitats in the Gulf of Maine (Dijkstra et al. 2017a). Warming waters have been shown to eliminate thermal barriers that historically limit reproductive success of marine invasive species (e.g., the tunicate *Botrylloides violaceus*), which may impact

native macroalgal abundance and distribution (Dijkstra et al. 2017a). Marine invasive species, likely introduced via maritime transport vectors, are known to compete for space and foul benthic substrates (Pappal 2010) and may replace native macroalgae species in portions of the study area (Scheibling and Gagnon 2006; Trott and Entreline 2019).

Although commercial and recreational harvest and culturing of rockweed occurs in Maine, there is little information available suggesting widespread impacts associated with controlled harvesting. However, at least one study (Wilson et al. 2019) projects the rockweed *A. nodosum* will be extirpated from U.S. waters by 2100 under the RCP8.5 scenario, which suggests future harvests of this species may be problematic.

Literature Cited

Colle BA, Zhang Z, Lombardo KA, Chang E, Liu P, Zhang M. 2013. Historical evaluation and future prediction of eastern North American and western Atlantic extratropical cyclones in the CMIP5 models during the cool season. Journal of Climate 26(18):6882-903.

Connell SD, Foster MS, Airoldi L. 2014. What are algal turfs? Towards a better description of turfs. Marine Ecology Progress Series 495:299–307.

Dijkstra JA, Westerman EL, Harris LG. 2017a. Elevated seasonal temperatures eliminate thermal barriers of reproduction of a dominant invasive species: A community state change for northern communities? Diversity and Distributions 23:1182–92.

Dijkstra JA, Harris LG, Mello K, Litterer A, Wells C, Ware C. 2017b. Invasive seaweeds transform habitat structure and increase biodiversity of associated species. Journal of Ecology. 10.1111/1365-2745.12775

Dijkstra JA, Litterer A, Mello K, O'Brien BS, Rzhanov Y. 2019. Temperature, phenology, and turf macroalgae drive seascape change: Connections to mid-trophic level species. Ecosphere 10(11):e02923. https://doi.org/10.1002/ecs2.2923.

Emanuel K. 2005. Increasing destructiveness of tropical cyclones over the past 30 years. Nature 436(7051):686-8.

Feehan CJ, Grace SP, Narvaez CA. 2019. Ecological feedbacks stabilize a turf-dominated ecosystem at the southern extent of kelp forests in the Northwest Atlantic. Scientific Reports 9:7078. https://doi.org/10.1038/s41598-019-43536-5.

Filbee-Dexter K, and Wernberg T. 2018. Rise of turfs: A new battlefront for globally declining kelp forests. BioScience 68:64–76. https://doi.org/10.1093/biosci/bix147.

Gao K, Aruga Y, Asada K, Ishihara T, Akano T, Kiyohara M. 1993. Calcification in the articulated coralline alga *Corallina pilulifera*, with special reference to the effect of elevated CO_2 concentration. Marine Biology 117(1):129-32.

Gledhill DK, White MM, Salisbury J, Thomas H, Misna I, Liebman M, Mook B, Grear J et al. 2015. Ocean and coastal acidification off New England and Nova Scotia. Oceanography 28(2):182–97. http://dx.doi.org/10.5670/oceanog.2015.41.

Harley CDG, Anderson KM, Demes KW, Jorve JP, Kordas RL, Coyle TA, Graham MH. 2012. Effects of climate change on global seaweed communities. Journal of Phycology 48:1064–78. https://doi.org/10.1111/j.1529-8817.2012.01224.x.

Knutson TR, McBride JL, Chan J, Emanuel K, Holland G, Landsea C, Held I, Kossin JP, Srivastava AK, Sugi M. 2010. Tropical cyclones and climate change. Nature Geoscience 3(3):157-63.

Koch M, Bowes G, Ross C, Zhang X-H. 2013. Climate change and ocean acidification effects on seagrasses and marine macroalgae. Global Change Biology 19(1):103-32.

Kossin JP, Knapp KR, Vimont DJ, Murnane RJ, Harper BA. 2007. A globally consistent reanalysis of hurricane variability and trends. Geophysical Research Letters 34(4):1-6.

Kroeker KJ, Kordas RL, Crim R, Hendriks IE, Ramajo L, Singh GS, Duarte CM, Gattuso J-P. 2013. Impacts ofocean acidification on marine organisms: quantifying sensitivities and interaction with warming. Global Change Biology 19(6):1884-96.

Lehmann J, Coumou D, Frieler K, Eliseev AV, Levermann A. 2014. Future changes in extratropical storm tracks and baroclinicity under climate change. Environmental Research Letters 9:8.

Mathieson AC, Pederson JR, Neefus CD, Dawes CJ, Bray TL. 2008. Multiple assessments of introduced seaweeds in the Northwest Atlantic. ICES Journal of Marine Science 65:730–41.

Mathieson AC, Dawes CJ, Hehre EJ, Harris LG. 2010. Floristic studies of seaweeds from Cobscook Bay, Maine. Northeastern Naturalist 16 (Monograph 5):1–48.

Orris PK. 1980. Revised species list and commentary on the macroalgae of the Chesapeake Bay in Maryland. Estuaries 3(3):200-6.

Pappal A. 2010. Marine invasive species. State of the Gulf of Maine Report. [Currier P, Gould D, Hertz L, Huston J, Tremblay ML (eds)]. The Gulf of Maine Council on the Marine Environment. 1-21.

Scheibling RE, Gagnon P. 2006. Competitive interactions between the invasive green alga *Codium fragile* ssp. *tomentosoides* and native canopy-forming seaweeds in Nova Scotia (Canada). Marine Ecology Progress Series 325:1–14. DOI:10.3354/meps325001.

Steneck R, Graham MH, Bourque BJ, Corbett D, Erlandson JM, Estes JA, Tegner MJ. 2002. Kelp forest ecosystems: biodiversity, stability, resilience and future. Marine Sciences Faculty Scholarship. 65. https://digitalcommons.library.umaine.edu/sms_facpub/65.

Trenberth K. 2005. Uncertainty in hurricanes and global warming. Science 308(5729):1753-4.

Trott TJ, Enterline C. 2019. First record of the encrusting bryozoan *Cribrilina* (*Juxtacribrilina*) *mutabilis* (Ito, Onishi and Dick, 2015) in the Northwest Atlantic Ocean. BioInvasions Records 8.

U.S. Global Change Research Program (USGCRP). 2017. Climate Science Special Report: Fourth National Climate Assessment, Volume I [Wuebbles, D.J., D.W. Fahey, K.A. Hibbard, D.J. Dokken, B.C. Stewart, and T.K. Maycock (eds.)]. U.S. Global Change Research Program. Washington, DC, USA. 470 pp. doi: 10.7930/J0J964J6.

Van Patten MS, Yarish C. 2009. Seaweeds of Long Island Sound. Bulletin 39. Connecticut College Arboretum.

Webster PJ, Holland GJ, Curry JA, Chang H-R. 2005. Changes in tropical cyclone number, duration, and intensity in a warming environment. Science 309(5742):1844-6.

Wilson KL, Skinner MA, Lotze HK. 2019. Projected 21st-century distribution of canopy-forming seaweeds in the Northwest Atlantic with climate change. Diversity and Distributions 25:582–602. https://doi.org/10.1111/ddi.12897.

Witman JD, Lamb RW. 2018. Persistent differences between coastal and offshore kelp forest communities in a warming Gulf of Maine. PLoS ONE 13(1):e0189388. https://doi.org/.10.1371/journal.pone.0189388.

Young CS, Gobler CJ. 2016. Ocean acidification accelerates the growth of two bloom-forming macroalgae. PLoS ONE 11(5):e0155152. doi:10.1371/journal.pone.0155152.

Estuarine Submerged Aquatic Vegetation

System: Estuarine

Subsystem: Subtidal & Intertidal

Class: Aquatic Bed

Sub-class: Rooted Vascular

Geographic Area: Entire Area

Overall Vulnerability Rank = High

Habitat Sensitivity = High

Climate Exposure = High

Estuarine Submerged Aquatic Vegetation		Attribute Mean	Data Quality	Distribution of Expert Scores
Sensitivity Attributes	Habitat condition	3.2	2.6	
	Habitat fragmentation	3.3	2.3	
	Distribution/Range	2.9	2.3	
	Mobility/Ability to spread or disperse	2.9	1.6	
	Resistance	3.2	2.4	
	Resilience	3.4	2.5	
	Sensitivity to changes in abiotic factors	3.3	2.5	
	Sensitivity and intensity of non−climate stressors	3.7	2.6	
	Dependency on critical ecological linkages	2.4	2.1	
	Sensitivity Component Score	High		
Exposure Factors	Sea surface temp	3.7	2.5	
	Bottom temp	n/a	n/a	
	Air temp	n/a	n/a	
	River temp	n/a	n/a	
	Surface salinity	2.8	2.1	
	Bottom salinity	n/a	n/a	
	pH	4	2	
	Sea level rise	2.7	2.2	
	Precipitation	2.7	2.1	
	Floods	n/a	n/a	
	Droughts	n/a	n/a	
	Exposure Component Score	High		
**Overall Vulnerability Rank**		**High**		

Low

Moderate

High

Very High

Habitat Name: Estuarine Submerged Aquatic Vegetation

System: Estuarine

Sub-System: Subtidal and Intertidal

Class: Aquatic Bed

Sub-class: Rooted vascular

Geographic Area: Entire Area

Habitat Description: This sub-class includes rooted vascular beds occurring in the estuarine system of the study area from near full-salinity to brackish waters (≤30 ppt to >0.5 ppt). Eelgrass (*Zostera marina*) is the dominant rooted vascular plant found in the estuarine environment over its western Atlantic range from North Carolina to Canada (Thayer et al. 1984). Widgeon grass (*Ruppia maritima*) can be found in estuarine waters in discrete meadows or intermixed with eelgrass (Kantrud 1991). Widgeon grass has a broad geographic range spanning Florida to Canada (Kantrud 1991). Both species require sediments that allow for root penetration, thus sand and silt are the most common. However, eelgrass can colonize areas of gravel with underlying sand and in one instance was observed growing on a section of the seafloor that was Boston blue clay (Colarusso, pers obs). Due to the high light requirements for both species, they are generally restricted to shallow coastal waters (Thayer et al. 1984; Kantrud 1991).

Eelgrass has been observed rooted at 44 feet mean low water offshore of Rhode Island (Short, pers. comm.), but 25 feet mean low water is generally a maximum depth for New England (Colarusso, pers. obs.). Widgeon grass grows in shallow water with maximum depth limits of less than 10 feet mean low water (Kantrud 1991). Both species can persist in the intertidal, but only at higher latitudes (generally Maine/NH border northward) due to their sensitivity to desiccation (Thayer et al. 1984; Kantrud 1991). In addition, wild celery (*Vallisneria americana*) is a freshwater plant that can tolerate limited salinity levels to 18 ppt., although the limit of salt tolerance is in question (Doering et al. 2001). Other rooted vascular plants in estuaries include pondweed species (e.g., sago pondweed *Stuckenia pectinatus* and redhead grass *Potamogton perfoliatus*), which although have some tolerance to salinity are generally restricted to less than 10 ppt. (Moore 2009).

Overall Climate Vulnerability Rank: High (97% certainty from the bootstrap analysis)

Climate Exposure: High. The overall High exposure score was influenced by two Very High attribute means: Sea Surface Temperature (3.7) and pH (4.0). Surface Salinity and Precipitation also received Very High and high scores (2.8 and 2.7, respectively). Sea level rise (SLR) is expected to be significant throughout the entire study area, but the greatest relative rise is projected in the Mid-Atlantic. The change in sea surface temperature is projected to be greater inshore than offshore and slightly greater in New England than the Mid-Atlantic.

Habitat Sensitivity: High. All nine sensitivity attributes received some High and Very High scores. Six of the nine sensitivity attributes means were > 3.0, while the other attributes had scores between 2.4 and 2.9. The highest sensitivity attribute mean was for Sensitivity and Intensity of Non-Climate Stressors (3.7). Habitat Condition (3.2), Habitat Fragmentation (3.3), Resistance (3.2), Resilience (3.4) and Sensitivity to Changes in Abiotic Factors (3.3) all scored above 3.0.

Data Quality & Gaps: The data quality scores for four of the five climate exposure factors (pH, Precipitation, Surface Salinity and SLR) were scored relatively Low to Moderate (≤2.2). The highest score was for Sea Surface Temperature (2.5). Data quality is believed to be lower for estuarine habitats due to the low resolution of CMIP5 and ROMS-NWA projections for climate exposure in estuaries and nearshore, shallow coastal areas.

For habitat sensitivity, only two of the nine attributes had relatively low (≤2.1) data quality scores (Mobility/Ability to Spread or Disperse and Dependency on Critical Ecological Linkages). The rest were between 2.3 and 2.6, which likely reflects the relative robust understanding of responses by subtidal, rooted vascular plants to non-climate and climate stressors.

Positive or Negative Climate Effect in the Northeast U.S. Shelf: The effect of climate change on estuarine rooted vascular beds in the Northeast U.S. Shelf is expected to be negative (80% of the experts' scores were negative, 15% were neutral and 5% were positive).

Climate Effects on Habitat Condition and Distribution: Eelgrass, the dominant rooted vascular bed species in the study area, is considered a "cold" water plant because when light is not a limiting factor it does best metabolically at 5ºC (Marsh et al. 1986). In the absence of light limitation, it shows positive metabolic growth up to 25ºC, at which point carbon loss due to respiration equals carbon gain from photosynthesis (Marsh et al. 1986). At 25ºC, eelgrass growth is reduced (Kaldy 2014; Thom et al. 2014), and at temperatures above 23ºC, primary production is reduced (Moore et al. 1996). Plants can persist above 25ºC for a while by using carbon reserves, but extended time above this temperature risks depleting important reserves needed to overwinter.

Increases in water temperature may impact the normal timing of flowering and seed production in both eelgrass and widgeon grass (Short and Neckles 1999). Increases in water temperature as small as 1ºC has been shown to advance flower formation in eelgrass by 12 days and seedling maturation by 10.8 days (Blok et al. 2018). It is unclear what changes in the timing of the normal reproductive cycle may mean for the long-term survival of individual meadows.

Increased water temperatures have the potential to reduce the existing distribution and productivity of eelgrass over its existing range (Moore et al. 1996; Short and Neckles 1999). Widgeon grass is unlikely to be negatively affected by increasing water temperature along the Atlantic coast, due to its higher temperature tolerance (Kantrud 1991). As water temperatures increase, it is likely that widgeon grass distribution will actually increase in the study area, by replacing eelgrass meadows in the southern portion of eelgrass' current distribution (Moore et al. 2014). For most of its range, eelgrass actively grows from spring through fall. At the southern edge of its range, eelgrass grows from fall through spring, disappearing in the summer (Thayer et al. 1984; Short and Neckles 1999). As sea surface temperature increases, it is likely this adaptation in the growing season will move northward (Short and Neckles 1999).

Increased water temperature may also lead to greater survival and distribution of invasive species that have been shown to have negative impacts to eelgrass (Neckles 2015; Carman et al 2019; Young and Elliot 2020). Warmer winter temperatures have led to greater green crab overwinter survival (Young and Elliott 2020) and green crabs have been shown to cause the decline of hundreds of acres of eelgrass in Maine and Canada (Neckles 2015). Invasive tunicates also have the potential to lead to eelgrass shoot mortality (Wong and Vercaemer 2012). Latitudinal changes in invasive tunicates distribution on eelgrass have been documented and changes in water temperature are likely a contributing factor (Carman et al. 2016; Carman et al. 2019).

Eelgrass has relatively high light requirements for its survival and it has been shown that small changes in water clarity can result in dramatic reductions in eelgrass production and survival (Short and Wyllie-Echeverria 1996; Bertelli and Unsworth 2018). Increased frequency and volume of rainfall due to climate change will generally result in diminished water clarity in many nearshore environments. This will be particularly acute in urban areas and at the mouths of freshwater inputs. Eelgrass meadows that experience light limitation will exhibit this in two ways: the deep edge of the meadow will contract into shallower water, thus reducing the acreage of the habitat, and the main body of the meadow will also thin out, as reductions in shoot density reduces self-shading (Ralph et al. 2007). Reducing shoot density of a meadow also reduces the meadow's ecological functions. Sparser meadows are less valuable fish habitat, sequester less carbon, and are not as prolific primary producers (Novak et al. 2020). Widgeon grass tends to grow in shallower water than eelgrass, so light limitation is not as critical for this species. It is unlikely that changes in water clarity will significantly impact widgeon grass.

Increases in the frequency and magnitude of large-scale storm events can have significant impacts to both of these species. In May 2006, the "Mother's Day" storm delivered over 20 inches of rainfall to southern New

Hampshire and the Great Bay watershed. This large infusion of freshwater resulted in salinities dropping in the bay to almost 0 ppt. for several weeks. Mapping of eelgrass in 2006 showed a greater than 1,000 acre reduction compared to levels in 2005, and a decline in widgeon grass as well (Short 2008; PREP 2018). Both eelgrass and widgeon grass are nearshore shallow water plants that are susceptible to impacts from storm generated waves. Significant sections of meadows of both of these species can be uprooted particularly if coastal storm generated waves coincide with a negative low tide. Estuarine subtidal vascular bed habitats may experience greater disturbance from storms due to climate change, which are expected to increase in frequency and intensity in the Northeast region (USGCRP 2017).

None of the potential impacts of climate change occur in isolation. Reduced light penetration, due to enhanced storm activity and sea level rise, will have significant negative impacts to both eelgrass and widgeon grass distribution and productivity (Kantrud 1991; Short and Neckles 1999). For eelgrass in particular, small changes in light, co-occurring with elevated water temperatures can trigger mortality (Moore et al. 2012).

Eelgrass resilience in the face of climate change can be thought of in multiple ways. Meadows with higher genetic diversity have proven more resilient to extended heat waves (Dubois et al. 2019). Eelgrass meadows have some ability to recover from anthropogenic stress, provided that water clarity and sediment conditions remain conducive to eelgrass growth (Neckles et al. 2005).

Habitat Summary: This habitat exists in shallow water in close proximity to many anthropogenic stressors. Given the projected changes in climate and increasing anthropogenic activity in the nearshore coastal zone, the abundance and condition of this habitat is expected to decline. Areas of greatest decline are in close proximity to large population centers and waters from Cape Cod south. Some meadows periodically become carbon limited, reducing their growth and production. However, elevated CO_2 concentrations could reduce these periods of carbon limitation and enhance seagrass growth and production (Alexandre et al. 2012).

Estuarine rooted vascular plants are experiencing a global decline. It has been estimated that 110 km^2/yr is being lost and 29% of the seagrass initially recorded in the late 1800s is now gone (Waycott et al. 2009). Since 1990 the rate of global losses is accelerating and is estimated to be occurring at about 7% per year (Waycott et al. 2009).

Estuarine rooted vascular plants are at high risk for anthropogenic impacts due to their preference for shallow coastal waters. Relatively small declines in water quality, usually due to nutrient over-enrichment, can lead to relatively large reductions in plant resilience, productivity and abundance (Short and Wyllie-Echeverria 1996). Physical stressors, such as dredging, filling, scouring from moorings and cutting from propellers can impact rooted vascular plant distribution (Short and Wyllie-Echeverria 1996). Some fragmentation occurs naturally within seagrass meadows, but anthropogenic activities, especially the physical stressors can create holes and patchiness in meadows. The creation of holes and patchiness increases the amount of edge habitat, which increases the risk of erosion and reduces its value as a fish habitat.

Climate change exacerbates the levels of anthropogenic stress rooted vascular plants are already experiencing. Their preferred habitat of shallow coastal waters makes them particularly susceptible to the effects of climate change. In general, the magnitude of warming will be greater in shallow water than deeper ocean waters. Proximity to the shoreline puts these habitats at greater risk of experiencing increased levels of turbidity from larger and more frequent precipitation events. Additionally, sea level rise will contribute to reduced quantities of light reaching these meadows, forcing a landward migration into shallower water where that may be possible. Shoreline armoring can impede landward movement of seagrass beds as sea levels rise (Short and Neckles 1999; Orth et al. 2017). Shoreline structures can deflect wave energy and cause increased turbulence and scouring of sediment and vegetation along their waterward edge, and increase suspended sediments and turbidity (Williams and Thom 2001).

Literature Cited

Alexandre A, Silva J, Buapet, P, Bjork M, Santos R. 2012. Effects of CO_2 enrichment on photosynthesis, growth and nitrogen metabolism of the seagrass *Zostera noltii*. Ecology and Evolution 2(10):2625-35.

Bertelli CM, Unsworth RKF. 2018. Light stress responses by the eelgrass, *Zostera marina* (L.). Frontiers in Environmental Science 6:1-39. https://doi.org/10.3389/fenvs.2018.00039.

Blok SE, Olesen B, Krause-Jensen D. 2018. Life history events of eelgrass *Zostera marina* L. populations across a gradient of latitude and temperature. Marine Ecology Progress Series 590:70-93. https://doi.org/10.3354/meps12479.

Carman MR, Colarusso PD, Nelson EP, Grunden DW, Wong MC, McKenzie C, Matheson K, Davidson J, Fox S, Neckles HA, Bayley H, Schott S, Dijkstra JA, Stewart-Clark S. 2016. Distribution and diversity of tunicates utilizing eelgrass as substrate in the western North Atlantic between 39º and 47º north latitude (New Jersey to Newfoundland). Management of Biological Invasions 7(1):51-7.

Carman MR, Colarusso PD, Neckles HA, Bologna P, Caines S, Davidson JDP, Evans, NT, Fox SE, Grunden DW, Hoffman S, Ma KCK, Matheson K, McKenzie CH, Nelson EP, Plaisted H, Reddington E, Schott S, Wong MC. 2019. Biogeographical patterns of tunicates utilizing eelgrass as a substrate in the western North Atlantic between 39º and 47º north latitude (New Jersey to Newfoundland). Management of Biological Invasions 10(4):602-16.

Doering P, Chamberlain R, McMunigal J. 2001. Effects of simulated saltwater intrusions on the growth and survival of wild celery, *Vallisneria americana*, from the Caloosahatchee estuary (South Florida). Estuaries 24(6):894-903. http://www.jstor.org/stable/1353180

Dubois K, Abbott JM, Williams SL, Stachowicz JJ. 2019. Relative performance of eelgrass genotypes shifts during an extreme warming event: disentangling the roles of multiple traits. Marine Ecology Progress Series 615:67-77. https://doi.ord/10.3354/meps12914

Kaldy JE. 2014. Effect of temperature and nutrient manipulations on eelgrass *Zostera marina* L. from the Pacific Northwest, USA. Journal of Experimental Marine Biology and Ecology 453:108-15. https://doi.org/10.1016/j.jembe.2013.12.0202

Kantrud HA. 1991. Widgeongrass (*Ruppia maritima* L.): a literature review. U.S. Fish and Wildlife Service. Fish and Wildlife Research 10. 58 pp.

Marsh JA, Dennison WC, Alberte RS. 1986. Effects of temperature on photosynthesis and respiration in eelgrass (*Zostera marina* L.). Journal of Experimental Marine Biology and Ecology 101:257-67.

Moore KA, Neckles HA, Orth RJ. 1996. *Zostera marina* (eelgrass) growth and survival along a gradient of nutrients and turbidity in the lower Chesapeake Bay. Marine Ecology Progress Series 142(1-3):247-59.

Moore KA. 2009. Submerged aquatic vegetation of the York River. Journal of Coastal Research SI 57:50-8.

Moore KA, Shields EC, Parrish DB, Orth RJ. 2012. Eelgrass survival in two contrasting systems: role of turbidity and summer water temperatures. Marine Ecology Progress Series 448:247-58. https://doi:103354/meps09578.

Moore KA, Shields EC, Parrish DB. 2014. Impacts of varying estuarine temperature and light conditions on *Zostera marina* (eelgrass) and its interactions with *Ruppia maritima* (widgeon grass). Estuaries and Coasts 37(1):20-30.

Neckles HA. 2015. Loss of eelgrass in Casco Bay, Maine, linked to green crab disturbance. Northeastern Naturalist 22(3):478-500.

Neckles HA, Short FT, Barker S, Kopp BS. 2005. Disturbance of eelgrass *Zostera marina* by commercial mussel *Mytilus edulis* harvesting in Maine: dragging impacts and habitat recovery. Marine Ecology Progress Series 285:57-73.

Novak AB, Pelletier MC, Colarusso P, Simpson J, Gutierrez MN, Arias-Ortiz A, Charpentier M, Masque P, Vella P. 2020. Factors influencing carbon stocks and accumulation rates in eelgrass meadows across New England, USA. Estuaries and Coasts 43:2076–91. https://doi.org/10.1007/s12237-020-00754-9.

Orth RJ, Dennison WC, Lefcheck JS, Gurbisz C, Hannam M, Keisman J, Landry JB, Moore KA, Murphy RR, Patrick CJ, Testa J, Weller DE, Wilcox DJ. 2017. Submersed aquatic vegetation in Chesapeake Bay: sentinel species in a changing world. BioScience 67(8):698-712.

Piscataqua Region Estuaries Partnership (PREP). 2018. [Internet]. State of Our Estuaries Report. 52 pp. (Accessed 23 November 2020). https://prepestuaries.org/resources/2018-sooe-report/

Ralph P, Durako MJ, Enriquez S, Collier CJ, Doblin M. 2007. Impact of light limitation on seagrasses. Journal of Experimental Marine Biology and Ecology 350(1-2):176-93.

Short FT. 2008. [Internet]. Eelgrass distribution in the Great Bay estuary. PREP reports and publications. 86. (Accessed 23 November 2020). https://scholars.unh.edu/prep/86.

Short FT, Neckles HA. 1999. The effects of global climate change on seagrasses. Aquatic Botany 63:169-96.

Short FT, Wyllie-Echeverria S. 1996. Natural and human-induced disturbance of seagrasses. Environmental Conservation 23(1):17-27.

Thayer GW, Kenworthy WJ, Fonseca MS. 1984. The ecology of eelgrass meadows of the Atlantic coast: a community profile. U.S. Fish Wildlife Service. FWS/OBS-84/02. 147pp.

Thom R, Southard S, Borde A. 2014. Climate-linked mechanisms driving spatial and temporal variation in eelgrass (*Zostera marina* L.) growth and assemblage structure in Pacific Northwest estuaries, U.S.A. Journal of Coastal Research Special Issue (68):1-11.

U.S. Global Change Research Program (USGCRP). 2017. Climate Science Special Report: Fourth National Climate Assessment. Volume I. [Wuebbles, D.J., D.W. Fahey, K.A. Hibbard, D.J. Dokken, B.C. Stewart, and T.K. Maycock (eds.)]. U.S. Global Change Research Program. Washington, DC. USA. 470 pp. doi: 10.7930/J0J964J6.

Waycott M, Duarte CM, Carruthers TBJ, Orth RJ, Dennison WC, Olyarnik S, Calladine A, Fourqurean JW, Heck jr KL, Hughes AR, Kendrick GA, Kenworthy WJ, Short FT, Williams SL. 2009. Accelerating loss of seagrass across the globe threatens coastal ecosystems. Proceedings of the National Academy of Sciences United States of America 106(30):12377-81. https://doi.org/10.1073/pnas.0905620106.

Williams GD, Thom RM. 2001. Marine and estuarine shoreline modification issues. White paper submitted to Washington Department of Fish and Wildlife. Washington Department of Ecology and Washington Department of Transportation. 1-99.

Wong MC, Vercaemer, B. 2012. Effects of invasive tunicates and a native sponge on the growth, survival, and light attenuation of eelgrass (*Zostera marina*). Aquatic invasions 7(3):315-26.

Young AM, Elliott JA. 2020. Life history and population dynamics of green crabs (*Carcinus maenas*). Fishes 5(1):4. https://doi10:3390/fishes5010004.

Estuarine Subtidal Shellfish Reef

System: Estuarine

Subsystem: Subtidal

Class: Reef

Sub-class: Mollusk

Geographic Area: Entire Area

Overall Vulnerability Rank = High

Habitat Sensitivity = High

Climate Exposure = High

	**Estuarine Subtidal Shellfish Reef**	Attribute Mean	Data Quality	Distribution of Expert Scores
Sensitivity Attributes	Habitat condition	3.1	2.2	
	Habitat fragmentation	3	1.4	
	Distribution/Range	2.6	1.8	
	Mobility/Ability to spread or disperse	2.7	1.3	
	Resistance	2.4	1.8	
	Resilience	2.7	2	
	Sensitivity to changes in abiotic factors	3.3	2.3	
	Sensitivity and intensity of non−climate stressors	3.6	2	
	Dependency on critical ecological linkages	2.9	1.4	
	Sensitivity Component Score	High		
Exposure Factors	Sea surface temp	3.8	2.5	
	Bottom temp	n/a	n/a	
	Air temp	n/a	n/a	
	River temp	n/a	n/a	
	Surface salinity	2.7	2.1	
	Bottom salinity	n/a	n/a	
	pH	4	2	
	Sea level rise	2.6	2.2	
	Precipitation	2.8	2.1	
	Floods	n/a	n/a	
	Droughts	n/a	n/a	
	Exposure Component Score	High		
**Overall Vulnerability Rank**		**High**		

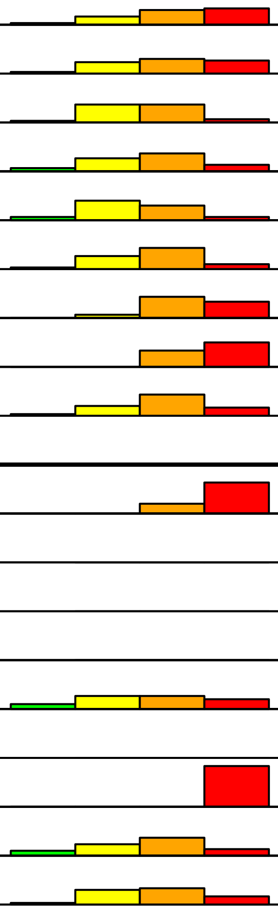

Low
Moderate
High
Very High

Habitat Name: Estuarine Subtidal Shellfish Reef

System**:** Estuarine

Subsystem: Subtidal

Class: Reef

Sub-class: Mollusk

Geographic Area: Entire Area

Habitat Description: This sub-class includes subtidal, reef-building shellfish that create a biotic hard substrate on benthic substrates in estuarine waters between 0.5 and 30 ppt. The most common species in the study area are blue mussels (*Mytilus edulis*) and eastern oyster (*Crassostrea virginica*). Oysters and mussels grow in dense aggregations forming reefs that extend off the seafloor. Oysters range from the Gulf of St. Lawrence, Canada to the Yucatan, West Indies and Brazil (Gunter 1951). Suitable substrates consist of sand, firm mud, or clay, whereas shifting sand and extremely soft mud are thought to be the only unsuitable substrates for oyster reef habitats (Galstoff 1964; Bahr and Lanier 1981). The blue mussel is a cosmopolitan species common to temperate and polar waters in the northern Atlantic Ocean from the southern Canadian Maritime provinces to North Carolina. Shellfish reefs are common in the intertidal and subtidal down to 10m.

Bivalve reefs serve as habitat for diverse assemblages of polychaetes, crustaceans, and other resident invertebrate and fish species (Wells 1961; Bahr and Lanier 1981; Rothschild et al 1994; Coen et al. 1999; Peterson et al. 2003; zu Ermgassen et al. 2016). They are filter feeders, thereby promoting greater water clarity and benthic productivity (Dame et al. 1984; Newell 1988; Ulanowicz and Tuttle 1992; Paerl et al. 1998). Bivalve reefs also remove excess nitrogen from coastal estuaries by promoting bacterially mediated denitrification as a consequence of concentrating bottom deposits of feces and pseudofeces (Newell et al. 2002; Piehler and Smyth 2011).

Overall Climate Vulnerability Rank: High (99.9% certainty from bootstrap analysis).

Climate Exposure: High. The overall High exposure score was influenced by the Very High attribute means of Sea Surface Ttemperature (3.8) and pH (4.0). The exposure attribute scores for Precipitation (2.8), Surface Salinity (2.7) and Sea Level Rise (SLR) (2.6) were also relatively high. SLR is expected to impact the entire study area but the greatest relative rise is projected in the Mid-Atlantic.

Habitat Sensitivity: High. Six of the nine sensitivity attribute means were ≥2.7: Habitat Condition (3.1), Habitat Fragmentation (3), Mobility/Ability to Spread or Disperse (2.7), Resilience (2.7), Sensitivity to Changes in Abiotic Factors (3.3), Sensitivity and Intensity of Non-Climate Stressors (3.6), and Dependency on Critical Ecological Linkages (2.9). Meanwhile, the attribute means for Resistance (2.4) and Distribution/Range (2.6) were between Moderate and High.

Data Quality & Gaps: Data quality for the climate exposure factors were scored as Moderate to High: Sea Surface Temperature (2.5), Surface Salinity (2.1), SLR (2.2), pH (2) and Precipitation (2.1). Data quality is generally lower for estuarine habitats due to the low resolution of CMIP5 and ROMS-NWA projections for climate exposure in estuaries and nearshore, shallow coastal areas. Another uncertainty factor is the relative disparate spatial data for mollusk reefs in some portions of the study area.

For habitat sensitivity attributes, data quality for all of the nine attributes were scored between 1.3 and 2.3, with Habitat Fragmentation (1.4), Distribution/Range (1.8), Mobility/Ability to Spread or Disperse (1.3), Resistance (1.8) and Dependency on Critical Ecological Linkages (1.4) under 2.0. Although the climate sensitivity for estuarine subtidal mollusk reefs are believed to be high, there remains some uncertainty in species' capacity to adapt and respond to climate change (e.g., synergistic effects of climate and non-climate stressors).

Relatively moderate to low data quality scores could reflect the fact that few comprehensive assessments for shellfish habitat exist in the study area as much of it was lost centuries ago (Rothschild et al. 1994; Kirby

2004; zu Ermgassen et al. 2012).

Positive or Negative Climate Effect for the Northeast U.S.: The effect of climate change on estuarine subtidal shellfish habitats in the Northeast U.S. is expected to be negative (60% of the experts' scores were negative, with 35% neutral and only 5% positive).

Climate Effects on Habitat Condition and Distribution: Several studies have examined the effects of climate and non-climate impacts on shellfish reefs. While overharvesting has historically been attributed primarily to oyster reef habitat loss in the northeast and elsewhere, hypoxia, disease, predators, competition, and sedimentation have impeded recent restoration efforts (Rothschild et al. 1994; Kirby 2004; Beck et al. 2011; zu Ermgassen et al. 2012). In addition, there is growing evidence that suggests potential climate effects on shellfish reefs may also be a factor.

Increasing seawater temperatures may affect the condition and distribution of subtidal shellfish in estuarine waters. For example, southern populations of blue mussels appear to be shifting northward in response to warming temperatures (Jones et al. 2010). Thermal stress, in conjunction with ocean acidification conditions, was found to cause metabolic depression in blue mussels from the Gulf of Maine (Lesser 2016), suggesting that elevated temperatures may result in suboptimal conditions for this cold-adapted species. Oyster growth and reproductive rates peak in waters ranging in temperature from 20-30°C and can live in water temperatures of 0-36°C (Shumway 1996; Lenihan 1999). In comparison, mussels are common in colder, more temperate to polar waters, thriving in 5-20°C, with an upper thermal tolerance limit of 29°C (Animal Diversity Web 2020).

Although the ROMS-NWA projections limit the ability to make precise climate predictions on impacts to estuarine subtidal shellfish reefs, water temperatures are projected to increase compared to historic means. The ROMS-NWA projections for RCP8.5 indicate the standardized anomaly for sea surface temperature will increase by at least 4 standard deviations from the historic means, and higher in the Gulf of Maine. Therefore, there is a possibility the maximum temperature thresholds for one or more life stages for shellfish may be exceeded by the end of the century, especially during episodic heat waves (Jones et al. 2009; Jones et al. 2010; Speights et al. 2017; Zippay and Helmuth 2012). Warming seawater can also increase the susceptibility of shellfish to disease, parasites and predation by local and invasive species (Burge et al. 2014; Smolowitz 2013).

Oyster performance peaks in salinities from 15 to 30 practical salinity units (psu), and they can withstand salinities of 0-40 psu. However, oysters tend to grow faster and be in better condition with less variationin salinity (Galtsoff 1964; Shumway 1996). Blue mussel is a euryhaline species, and can survive periodic fluctuations below 15 psu, although they do not thrive in low salinity conditions (Animal Diversity Web 2020). Although the ROMS-NWA climate projections for RCP8.5 indicate a decline in surface salinity for the U.S. continental shelf and the Gulf of Maine, the salinities for estuarine waters in Long Island Sound, Delaware Bay, and Chesapeake Bay are projected to increase by at least 2 standard deviations from the historic means.

Estuarine waters are generally more susceptible to acidification than oceanic waters because they are subject to more acid sources and are generally less buffered than oceanic waters (i.e., differences in the amount of dissolved inorganic carbon, dissolved and particulate organic carbon, total alkalinity and nutrients from riverine and estuarine sources) (Waldbusser et al. 2011; Ekstrom et al. 2015; Gledhill et al. 2015). A long-term monitoring program in the Chesapeake Bay found statistically significant declines in daytime average pH from 1985 to 2008 within polyhaline waters, but not in mesohaline waters of the Bay (Waldbusser et al. 2011). Rivers in New England that have a combination of cool temperatures, low alkalinity, and runoff typically consisting of soils containing carbonic acid, a by-product of organic decomposition, have particularly low aragonite saturation state values (Salisbury et al. 2008). For example, in the Casco Bay during times of high discharges from the Kennebec River and down-welling (northerly) winds, acidic river waters with very low aragonite saturation state values have been recorded (Salisbury et al. 2008). Overall acidification is expected to increase, although estuaries also naturally experience daily fluxes in pH and some

species acclimated to variability may be resilient to ocean acidification.

Ocean acidification will likely most directly negatively impact shellfish reefs, with larger negative effects on survival for larvae than adults (Kroeker et al. 2013). Ocean acidification is expected to reduce the shell size, thickness, calcification rates and growth, and survival of embryo and larval bivalves (Gazeau et al. 2013). Ries et al. (2009) exposed both mussels and oysters to low pH levels, and found that acidification negatively impacts oyster calcification rates whereas there was no relationship between pH and the calcification rate of blue mussels. Yet, increased water temperature in the northeastern U.S. is likely resulting in reduced physiological performance of mussels (Zippay and Helmuth 2012), potentially explaining why mussel beds are becoming less common in southern New England the western portions of the Gulf of Maine when temperatures have exceeded the thermal maxima for mussels. Dodd et al. (2015) exposed oysters and their predators to increased acidification and found that acidification negatively affected oyster growth, but also reduced crab consumption of oysters. The synergistic effects of temperature, salinity, and pH on metabolism in marine mollusks may be greater than reduced pH alone. Some studies have shown additive and synergistic negative effects on bivalves and gastropods from ocean acidification and low dissolved oxygen (Kroeker et al. 2013; Gobler et al. 2014; Clark and Gobler 2016; Gobler and Bauman 2016; Griffith and Gobler 2017).

Estuarine intertidal shellfish habitats may experience disturbance from storms, which are expected to increase in frequency and intensity in the Northeast region (USGCRP 2017). Coastal storms may increase physical/mechanical disturbance and stress to shellfish reef structure and associated organisms, and repeated disturbance may prohibit formation of robust three-dimensional complex structures.

Sea-level rise poses both direct and indirect threats to estuarine subtidal habitats. Shellfish reefs generally have the capacity to grow vertically and landward to keep up with sea level rise (Rodriguez et al. 2014). However, because they exist across a wide geographical and environmental range and diverse landscape settings, they are influenced by different types and magnitudes of stress, which influence growth rates. Oysters and blue mussels are restricted to shallow waters, in part as a function of water flow that influences food availability and by the movement of drifting larval and juvenile stages (Lenihan 1999), so increased rates of sea level rise could exceed the ability of subtidal shellfish reefs to maintain optimal water depths.

Habitat Summary: Oysters were once abundant in nearly all the estuaries on the Atlantic coast (MacKenzie and Burrell 1997). Most oyster fisheries in Chesapeake Bay, Delaware Bay, and Pamlico Sound have been decimated (Coen and Grizzle 2007). Blue mussels, once a foundational species known to influence diversity and productivity in the Gulf of Maine, have declined since the 1970s and are now a minor contributor to the compositional patterns of intertidal communities (Sorte et al. 2017).

Bivalve habitats have been highly altered, with estimates in the U.S. suggesting that 68% of historic oyster reef extent and over 80% of the productivity of these habitats have been lost primarily due to overharvesting and destructive harvesting practices, but also as a consequence of dredge and fill activities, disease, sedimentation, predators, and competition (zu Ermgassen et al. 2012).

Shellfish reefs have been highly altered, with estimates in the U.S. suggesting that 68% of historic oyster reef extent and over 80% of the productivity of these habitats have been lost primarily due to overharvesting and destructive harvesting practices, but also as a consequence of dredge and fill activities disease, sedimentation, predators, and competition (zu Ermgassen et al. 2012). Historically, overharvesting has been the largest threat to shellfish habitats, with 85% of oyster reefs lost worldwide (Beck et al. 2011), and similar impacts having occurred in the U.S. (zu Ermgassen et al. 2012). Kirby (2004) suggested that harvesting of oysters peaked and then the fisheries collapsed in the northeastern

U.S. in coastal Massachusetts and Southern New England in the early 1800’s. Efforts to conserve and rebuild shellfish reefs have been challenged by anthropogenic disturbances such as bottom water hypoxia, dredge and fill activity, shoreline hardening, diseases such as Dermo and MSX, sedimentation, predation, and

competition (Rothschild et al. 1994; Kirby 2004; Beck et al. 2011; zu Ermgassen et al. 2012). High temperature and low salinity are known drivers for both MSX and Dermo (Burreson et al. 2000; Ford and Smolowitz 2007; Burge et al. 2014). Between 1990 and 1992, a dramatic range extension of Dermo disease was reported over a 500 km area in the northeastern United States from Delaware Bay, New Jersey to Cape Cod Bay, Massachusetts (Ford and Smolowitz 2007). By 1995, Dermo was reported as far north as Maine (Burge et al. 2014).

In coastal New England, many shellfish reefs suffer from poor recruitment, motivating restoration practitioners to seed reefs with juvenile oysters set on dead oyster shells at oyster hatcheries and then transplanted to shallow reefs (personal observation). Unfortunately, many of the restored oyster reefs in coastal Rhode Island's salt ponds have failed to increase natural recruitment (Grabowski and Hughes, unpublished data). Anecdotally, many of the mussel beds that were common in northeastern Massachusetts are no longer present (Grabowski, personal observation), possibly a consequence of local warming of sea and air temperatures (Zippay and Helmuth 2012).

Climate change will exacerbate other anthropogenic effects that have negatively impacted shellfish reefs. Oysters are thought to be among the most vulnerable of species to ocean acidification given its impacts on shell calcification rates and the protection that the shell provides from predators (Ries et al 2009). Although oyster reefs can grow relatively quickly and keep pace with sea-level rise, shoreline development in many regions may have removed available space for reefs to migrate landward. While mussels may be less vulnerable to acidification compared to oysters (Ries et al. 2009), they are being crowded out of coastal waters in New England likely as a consequence of air and sea water warming (Zippay and Helmuth 2012). Warming coupled with eutrophication common in many coastal estuaries will likely amplify the conditions that result in bottom water hypoxia, further contributing to subtidal shellfish reef habitat loss. Further investigation of how warming and acidification are impacting the early life history of oysters could help elucidate why recruitment failure is common in some areas of the northeastern U.S.

Literature Cited

Animal Diversity Web. 2020. [Internet]. *Mytilus edulis*. (Accessed August 2020). https://animaldiversity.org/accounts/Mytilus_edulis/.

Bahr LM, Lanier WP. 1981. The ecology of intertidal oyster reefs of the South Atlantic Coast: a community profile. U.S. Fish and Wildlife Service. FWS/OBS-81/15.

Baillie CB, Grabowski JH. 2018. Competitive and agonistic interactions between the invasive Asian shore crab and juvenile American lobster. Ecology 99:2067–79.

Beck MW, Brumbaugh RD, Airoldi L, Carranza A, Coen LD, Crawford C, Defeo O, Edgar G, Hancock B, Kay M, Lenihan H, Luckenbach M, Toropova C, Zhang G. 2011. Oyster reefs at risk and recommendations for conservation, restoration and management. Bioscience 61:107-16.

Burge CA, Eakin CM, Friedman CS, Froelich B, Hershberger PK, Hofmann EE, Petes LE, Prager KC, Weil E, Willis BL, Ford SE, Harvell CD. 2014. Climate change influences on marine infectious diseases: implications for management and society. Annual Review of Marine Science 6:249-77.

Burreson EM, Stokes NA, Friedman CS. 2000. Increased virulence in an introduced pathogen: *Haplosporidium nelsoni* (MSX) in the eastern oyster *Crassostrea virginica*. Journal of Aquatic Animal Health 12(1):1-8.

Clark H, Gobler CJ. 2016. Diurnal fluctuations in CO_2 and dissolved oxygen concentrations do not provide a refuge from hypoxia and acidification for early-life-stage bivalves. Marine Ecology Progress Series

558:43114. https://doi.org/10.3354/meps11852

Coen LD, Grizzle RE. 2007. The importance of habitat created by molluscan shellfish to managed species along the Atlantic Coast of the United States. Atlantic States Marine Fishery Management Commission. Habitat Management Series #8.

Coen LD, Luckenbach MW, Breitburg DL. 1999. The role of oyster reefs as essential fish habitat: a review of current knowledge and some new perspectives. American Fisheries Society Symposium 22:438-54.

Dame RF, Zingmark RG, Haskins E. 1984. Oyster reefs as processors of estuarine materials. Journal of Experimental Marine Biology and Ecology 83:239-47.

Dodd LF, Grabowski JH, Piehler MF, Westfield I, Ries JB. 2015. Ocean acidification impairs crab foraging behaviour. Proceedings of the Royal Society B: Biological Sciences 282.

Ekstrom JA, Suatoni L, Cooley SR, Pendleton LH, Waldbusser GG, Cinner JE, Ritter J, Langdon C, van Hooidonk R, Gledhill D, Wellman K, Beck MW, Brander LM, Rittschof D, Doherty C, Edwards PET, Portela R. 2015. Vulnerability and adaptation of US shellfisheries to ocean acidification. Nature Climate Change 5(3):207-14.

Ford SE, Smolowitz R. 2007. Infection dynamics of an oyster parasite in its newly expanded range. Marine Biology 151(1):119-33.

Galtsoff PS. 1964. The American oyster *Crassostrea virginica* (Gmelin). U.S. Fish Wildlife Service Fisheries Bulletin 64:1-480.

Gazeau F, Parker LM, Comeau S, Gattuso J-P, O'Connor WA, Martin S, Pörtner H-O, Ross PM. 2013. Impacts of ocean acidification on marine shelled molluscs. Marine Biology 160(8):2207-45.

Gledhill D, White M, Salisbury J, Thomas H, Misna I, Liebman M, Mook B, Grear J, Candelmo A, Chambers RC, Gobler C, Hunt C, King A, Price N, Signorini S, Stancioff E, Stymiest C, Wahle R, Waller J, Rebuck N, Wang Z, Capson T, Morrison JR, Cooley S, Doney S. 2015. Ocean and coastal acidification off New England and Nova Scotia. Oceanography 25(2):182-97.

Gobler CJ, Baumann H. 2016. Hypoxia and acidification in ocean ecosystems: coupled dynamics and effects on marine life. Biology Letters 12(5):20150976.

Gobler CJ, DePasquale EL, Griffith AW, Baumann H. 2014. Hypoxia and acidification have additive and synergistic negative effects on the growth, survival, and metamorphosis of early life stage bivalves. PLoS One 9(1):e83648.

Griffith AW, Gobler CJ. 2017. Transgenerational exposure of North Atlantic bivalves to ocean acidification renders offspring more vulnerable to low pH and additional stressors. Scientific Reports 7:11394.

Gunter G. 1951. The West Indian tree on the Louisiana coast, and notes on growth of the Gulf Coast oysters. Science 113:16-517.

Jones SJ, Lima FP, Wethey DS. 2010. Rising environmental temperatures and biogeography: poleward range contraction of the blue mussel, *Mytilus edulis* L., in the western Atlantic. Journal of Biogeography 37(12):2243-59.

Jones SJ, Mieszkowska N, Wethey DS. 2009. Linking thermal tolerances and biogeography: *Mytilus edulis* (L.)

at its southern limit on the east coast of the United States. Biological Bulletin 217(1):73-85.

Kirby MX. 2004. Fishing down the coast: historical expansion and collapse of oyster fisheries along continental margins. Proceedings of the National Academy of Science of the United States of America 101:13096-99.

Kroeker KJ, Kordas RL, Crim R, Hendriks IE, Ramajo L, Singh GS, Duarte CM, Gattuso J-P. 2013. Impacts of ocean acidification on marine organisms: quantifying sensitivities and interaction with warming. Global Change Biology 19(6):1884-96.

Lenihan HS. 1999. Physical-biological coupling on oyster reefs: how habitat structure influences individual performance. Ecological Monographs 69:251-75.

Lesser MP. 2016. Climate change stressors cause metabolic depressions in the blue mussel, *Mytilus edulis*, from the Gulf of Maine. Limnology and Oceanography 61:1705-17.

MacKenzie Jr CL, Burrell Jr VG, Rosenfield A, Hobart WL. (eds.). 1997. The history, present condition, and future of the molluscan fisheries of North and Central America and Europe, Volume 1, Atlantic and Gulf Coasts. U.S. Department of Commerce, NOAA Technical Report 127, 234 p.

Newell RIE. 1988. Ecological changes in Chesapeake Bay: are they the result of overharvesting the American oyster, *Crassostrea virginica*? *In:* Lynch MP, Krome EC (editors). Understanding the estuary: advances in Chesapeake Bay research. Chesapeake Research Consortium, Baltimore, Maryland, USA.

Newell RIE, Cornwell JC, Owens MS. 2002. Influence of simulated bivalve biodeposition and microphyrobenthos on sediment nitrogen dynamics: a laboratory study. Limnology and Oceanography 47:1367-79.

Paerl HW, Pinckney JL, Fear JM, Peierls BL. 1998. Ecosystem responses to internal and watershed organic matter loading: consequences for hypoxia in the eutrophying Neuse river estuary, North Carolina, USA. Marine Ecology Progress Series 166:17-25.

Peterson CH, Grabowski JH, Powers SP. 2003. Estimated enhancement of fish production resulting from restoring oyster reef habitat: quantitative valuation. Marine Ecology Progress Series 264:249-64.

Piehler MF, Smyth AR. 2011. Impacts of ecosystem engineers on estuarine nitrogen cycling. Ecosphere 2:art12.

Ridge JT, Rodriguez AB, Fodrie FJ, Lindquist NL, Brodeur MC, Coleman SE, Grabowski JH, Theuerkauf EJ. 2015. Maximizing oyster-reef growth supports green infrastructure with accelerating sea-level rise. Scientific Reports 5: 14785.

Ries JB, Cohen AL, McCorkle DC. 2009. Marine calcifiers exhibit mixed responses to CO_2-induced ocean acidification. Geology 37:1131-4.

Rodriguez AB, Fodrie FJ, Ridge JT, Lindquist NL, Theuerkauf EJ, Coleman SE, Grabowski JH, Brodeur MC, Gittman RK, Keller DA, Kenworthy MD. 2014. Oyster reefs can outpace sea-level rise. Nature Climate Change 4:493-7.

Rothschild BJ, Ault JS, Goulletquer P, Heral M. 1994. Decline of the Chesapeake Bay oyster population: a century of habitat destruction and overfishing. Marine Ecology Progress Series 111:29-39.

Salisbury J, Green M, Hunt C, Campbell J. 2008. Coastal acidification by rivers: a threat to shellfish? Eos,

Transactions American Geophysical Union 89(50):513-28.

Shumway CA. 1999. A neglected science: applying behavior to aquatic conservation. Environmental Biology of Fishes 55:183-201.

Smolowitz R. 2013. A review of current state of knowledge concerning *Perkinsus marinus* effects on *Crassostrea virginica* (Gmelin) (the Eastern oyster). Veterinary Pathology 50(3):404-11.

Sorte CJB, Davidson VE, Franklin MC, Benes KM, Doellman MM, Etter RJ, Hannigan RE, Lubchenco J, Menge BA. 2017. Long-term declines in an intertidal foundation species parallel shifts in community composition. Global Change Biology 23:341-52.

Speights C J, Silliman BR, McCoy MW. 2017. The effects of elevated temperature and dissolved ρCO_2 on a marine foundation species. Ecology and evolution 7(11):3808–14. https://doi.org/10.1002/ece3.2969

Ulanowicz RE, Tuttle JH. 1992. The trophic consequences of oyster stock rehabilitation in Chesapeake Bay. Estuaries 15:298-306.

U.S. Global Change Research Program (USGCRP). 2017. Climate Science Special Report: Fourth National Climate Assessment. Volume I. [Wuebbles, D.J., D.W. Fahey, K.A. Hibbard, D.J. Dokken, B.C. Stewart, and T.K. Maycock (eds.)]. U.S. Global Change Research Program. Washington, DC. USA. 470 pp. doi: 10.7930/J0J964J6.

Waldbusser GG, Voigt EP, Bergschneider H, Green MA, Newell RIE. 2011. Biocalcification in the eastern oyster (*Crassostrea virginica*) in relation to long-term trends in Chesapeake Bay pH. Estuaries and Coasts 34(2):221-31.

Wells HW. 1961. The fauna of oyster beds, with special reference to the salinity factor. Ecological Monographs 31:239-66.

Zippay ML, Helmuth B. 2012. Effects of temperature change on mussel, *Mytilus*. Integrative Zoology 7: 312-27.

zu Ermgassen PSE, Grabowski JH, Gair JR, Powers SP. 2016. Quantifying fish and mobile invertebrate production from a threatened nursery habitat. Journal of Applied Ecology 53:596-606.

zu Ermgassen PSE, Spalding MD, Blake B, Coen LD, Dumbauld B, Geiger S, Grabowski JH, Grizzle R, Luckenbach M, McGraw K, Rodney W, Ruesink JL, Powers SP, Brumbaugh RD. 2012. Historical ecology with real numbers: past and present extent and biomass of an imperilled estuarine habitat. Proceedings of the Royal Society B: Biological Sciences 279:3393-400.

Estuarine Subtidal Rocky Bottom

System: Estuarine

Subsystem: Subtidal

Class: Rocky Bottom

Sub-class: Bedrock, Rubble, Cobble Gravel

Geographic Area: Entire Area

Overall Vulnerability Rank = Low

Habitat Sensitivity = Low

Climate Exposure = High

	Estuarine Subtidal Rocky Bottom	Attribute Mean	Data Quality	Distribution of Expert Scores
Sensitivity Attributes	Habitat condition	2	2.1	
	Habitat fragmentation	1.7	1.7	
	Distribution/Range	2.6	1.7	
	Mobility/Ability to spread or disperse	2.4	1.7	
	Resistance	1.7	1.5	
	Resilience	2.3	2	
	Sensitivity to changes in abiotic factors	2.1	2	
	Sensitivity and intensity of non−climate stressors	2.4	2.1	
	Dependency on critical ecological linkages	2.4	2	
	Sensitivity Component Score	Low		
Exposure Factors	Sea surface temp	3.8	2.5	
	Bottom temp	n/a	n/a	
	Air temp	n/a	n/a	
	River temp	n/a	n/a	
	Surface salinity	2.1	2.3	
	Bottom salinity	n/a	n/a	
	pH	4	2	
	Sea level rise	2.2	2.2	
	Precipitation	n/a	n/a	
	Floods	n/a	n/a	
	Droughts	n/a	n/a	
	Exposure Component Score	High		
**Overall Vulnerability Rank**		**Low**		

Low
Moderate
High
Very High

Habitat Name: Estuarine Subtidal Rocky Bottom
System: Estuarine
Sub-System: Subtidal
Class: Rocky Bottom
Sub-class: Bedrock, Rubble, Cobble, Gravel
Geographic Area: Entire Area

Habitat Description: This habitat sub-class includes bedrock, rubble, and cobble/gravel in the estuarine subtidal zone. The sub-class also includes the epibenthic flora and fauna associated with these hard bottoms, but does not include specific habitats that were assessed separately (i.e., non-calcareous algal and rooted vascular beds, coral-dominated hard bottom, mollusk reef). Calcareous algae is included in this sub-class.

As a result of bedrock geology and glacial history, natural rocky estuarine habitats occur across a wide latitudinal range in New England (primarily in the Gulf of Maine), but are rare in the Mid-Atlantic. Natural rocky habitats range from granule/pebble (or gravel) to cobbles, boulders, and ledge/bedrock. Biota associated with all these habitat types also varies. Rocky estuarine habitats in New England are partially continuous with multiple, moderately-sized patches that vary between estuaries. Within individual estuaries, rocky bottom habitats are usually in close proximity to each other.

Overall Climate Vulnerability Rank: Low (65% certainty from bootstrap analysis). The majority of the bootstrap results match the results of the categorical vulnerability rank, but 25% of the bootstrap results were in the Moderate rank. This indicates that this habitat is in the high range of the Low vulnerability rank.

Climate Exposure: High. Two exposure factors contributed to the High exposure score: Sea Surface Temperature (3.8) and pH (4.0), both of which are projected to change significantly throughout the region, with warming temperatures and increasing ocean acidification (decreasing pH). The exposure scores for Surface Salinity and Sea Level Rise (SLR) were 2.3 and 2.2, respectively.

Habitat Sensitivity: Low. Most sensitivity attributes were centered around the Low and Moderate scoring bins. No sensitivity attributes scored above 3.0, with the highest scores falling in the Moderate range for Distribution/Range (2.6), Mobility/Ability to Spread or Disperse (2.4), Sensitivity and Intensity of Non-Climate Stressors (2.4), Dependency on Critical Ecological Linkages (2.4), and Resilience (2.3). This may reflect high variability in the expected sensitivities and types of rocky habitat found throughout the study area. Furthermore, while the abiotic component of rocky bottom habitats are both resistant and resilient to most stressors, the fauna and flora associated with the habitat may be sensitive to higher temperature and other projected changes.

Data Quality & Gaps: For climate exposure factors, the data quality score for SLR was the lowest (2.2), followed by Surface Salinity (2.3) and Sea Surface Temperature (2.5). Data quality is generally lower for estuarine habitats due to the low resolution of CMIP5 and ROMS-NWA projections for climate exposure in estuaries and nearshore, shallow coastal areas, which increases the uncertainty with overlap in climate exposure projections.

Seven of the nine sensitivity attribute data quality scores are less than or equal to 2.0. Resistance scored 1.5; Habitat Fragmentation, Distribution/Range, and Mobility/Ability to Spread or Disperse scored 1.7; and Resilience, Sensitivity to Changes in Abiotic Factors, and Dependency on Critical Ecological Linkages scored 2.0. For most of the sensitivity attributes, scores were placed in all of the scoring bins, which may reflect uncertainty and the variability of expected sensitivities of a large range of substrate types in the habitat sub-class. In addition, the low data quality scores may indicate uncertainty in responses of the fauna and flora associated with the habitat.

Positive or Negative Climate Effect in the Northeast U.S.: The effect of climate change on estuarine subtidal rocky bottom in the Northeast U.S. is expected to be neutral (70% of the experts' scores were neutral,

30% were negative). Climate change is expected to have relatively minor impacts on this habitat, with greater impacts expected from anthropogenic stressors.

<u>Climate Effects on Habitat Condition and Distribution</u>: In general, natural rocky substrates are not themselves sensitive to climate change, but associated fauna and flora are, and responses vary between species. It is assumed that subtidal flora and fauna are less adapted to extreme conditions (e.g., temperature, salinity) than those in the intertidal zone because the physical and chemical conditions those species have evolved under have generally been more stable. Conversely, because water temperatures for subtidal rocky habitats are generally less variable than intertidal rocky habitats, which are also exposed to air temperatures, the flora and fauna may be less vulnerable temperature extremes. Nonetheless, the projected changes to sea surface temperatures are high to very high, especially in the Gulf of Maine, such that any flora and fauna sensitive to warmer temperatures will be vulnerable to climate change.

As ocean acidification increases, echinoderms associated with rocky bottom habitats will experience negative effects on their growth and survival. Kroeker et al. (2013) reported the largest effects on growth for larval life stages in echinoderms. In addition, this study found calcareous algae is expected to be negatively affected by ocean acidification by lower abundance, and rates of photosynthesis and calcification.

As sea level rise increases the depth of shallow estuarine subtidal habitats, rocky substrates themselves have limited mobility depending on the diameter of the substrate and cannot move into shallower tidal zones, which may be further limited by hardened shoreline structures. However, organisms associated with rocky estuarine habitats have a high capability to spread through larval dispersal, propagules, or vegetative growth as long as suitable hard substrate is present. Rocky substrate is relatively common in New England, but less common in the Mid-Atlantic. The fauna and flora associated with hard substrates in the Mid-Atlantic region may have a lower probability of settling and establishing on natural rocky substrates as a result of less abundances for this habitat. Although artificial hard substrates (e.g., rock revetments, groins, jetties, seawalls) are available for settlement in the Mid-Atlantic, studies suggest the abundance and diversity of flora and fauna associated with some artificial hard substrates may be lower than natural rock substrates (Williams and Thom 2001; Chapman 2003; Gittman et al. 2016), which may reflect reduced success in settlement, growth, and survival.

In addition, some studies suggest a higher incidence of marine exotic/invasive species associated with artificial structures compared to native material (Tyrrell and Byers 2007; Pappal 2010; Geraldi et al. 2013). Increased reproduction of marine invasive species, combined with limited biological resistance in regions with cooler water temperatures, may lead to community state changes in shallow habitats in the study area (Dijkstra et al. 2019).

Warming waters have been shown to benefit non-native and invasive species (Stachowicz et al. 2002; Valentine 2009; Sorte et al. 2010), such as by eliminating thermal barriers that historically limit reproductive success of marine invasive species (e.g., the tunicate *Botrylloides violaceus*) (Dijkstra et al.2017). Marine invasive species, likely introduced via maritime transport vectors, are known to compete for space and foul benthic substrates (Pappal 2010) and may replace native rocky bottom fauna and flora in portions of the study area (Scheibling and Gagnon 2006; Trott and Entreline 2019).

While coastal storms are expected to increase in frequency and intensity in the Northeast region (USGCRP 2017), estuarine subtidal bedrock, boulders, and large cobble habitats are highly resistant to disturbance to a greater degree than smaller substrates like gravel. When physically disturbed (e.g., ice scour, storms), the function of these habitats is not impaired, though they may be temporarily buried by mobile sand. The recovery of subtidal flora and fauna from disturbance varies on the life history characteristics and the type and intensity of disturbance (Bertness et al. 2002). Recovery of longer-lived species (e.g., sponges, anemones, tunicates, molluscs) in deeper water can take years even without predators present. Mobile species quickly re-occupy rocky habitats after disturbance.

Habitat Summary: Coastal development and shoreline hardening affect benthic and fish communities through impacts on water quality, reduction in shallow water habitat, changes in hydrology, and increases in nutrient inputs (Bilkovic and Roggero 2008; Kornis et al. 2017). Increased shoreline hardening in heavily populated and industrialized areas is expected as SLR and storm impacts due to climate change become more severe and widespread. Fauna and flora can be highly sensitive toeutrophication from increased nutrient runoff. Dredging in shallow subtidal areas and bottom fishing inthe lower reaches of estuaries and coastal embayments can also damage natural rocky bottom habitats(Stevenson et al. 2004). Marine construction in subtidal areas (e.g., marine terminals, marinas, docks) are common in estuaries, and can convert rocky bottom habitat to non-native habitats, and shade epiflora growing on rocky bottoms (Johnson et al. 2008). Aquaculture operations may also cause some disturbance to benthic habitats through the accumulation of nutrients, wastes, or sediment deposition,but good water flow and husbandry limit this accumulation (ASMFC 2020).

Many introduced species have become established in rocky subtidal habitats in the Northeast, leading to changes in ecological processes and dynamics of fish and other mobile species (Dijkstra et al. 2017). In particular, warming temperatures and increasing frequency, intensity, and variability of storms favor the growth and persistence of turf macroalgae, which alter rocky bottom habitats, increase patchiness, and cause changes up the food web (Djikstra et al. 2017). Subtidal rocky bottom infauna and epifauna are also sensitive green crab (*Carcinus maenas*), an invasive species which is believed to have been carried by ships in ballast water and sold as fish bait in much of the world. It now has established populations in New England and northern Mid-Atlantic in both subtidal and intertidal zones. It is a predator of many forms of shore life, including worms and mollusks (GISD 2020). Although cold water temperatures were once thought to limit the northward expansion of the green crab (GISD 2020), recent research suggests both warm-water and cold-water genetic lineages exist, and the species has successfully invaded intertidal and shallow subtidal habitats throughout the study area (Lehnert et al. 2018).

Species that graze on epiflora and epifauna (e.g., sea urchins) control the abundances of species associated with rocky bottom habitats. Likewise, top predators (e.g., cod) can control the populations ofgrazers. For example, in some areas of the Gulf of Maine, urchin populations have exploded and decimated kelp beds (i.e., "urchin barrens"), which has been attributed to overfishing of the top predators (Steneck et al. 2002). The collapse in the populations of sea urchins can cause proliferation of macroalgae species that reduces species diversity of rocky habitats, and can interfere with settlement ofbenthic and demersal larval life stages.

Literature Cited

Atlantic States Marine Fisheries Commission (ASMFC). 2020. Aquaculture: effects on fish habitat along the Atlantic Coast. Atlantic States Marine Fisheries Commission Habitat Management Series #16. http://www.asmfc.org/files/Habitat/HMS16_Aquaculture_May2020.pdf

Bilkovic DM, Roggero MM. 2008. Effects of coastal development on nearshore estuarine nekton communities. Marine Ecology Progress Series 358:27-39.

Bertness MD, Trussell GC, Ewanchuk PJ, Silliman BR. 2002. Do alternate stable community states exist in the Gulf of Maine rocky intertidal zone? Ecology 83(12):3434-48.

Chapman MG. 2003. Paucity of mobile species on constructed seawalls: effects of urbanization on biodiversity. Marine Ecology Progress Series 264:21-9.

Dijkstra JA, Westerman EL, Harris LG. 2017. Elevated seasonal temperatures eliminate thermal barriers of reproduction of a dominant invasive species: A community state change for northern communities? Diversity and Distributions 23:1182–92.

Dijkstra JA, Litterer A, Mello K, O'Brien BS, Rzhanov Y. 2019. Temperature, phenology, and turf macroalgae drive seascape change: connections to mid-trophic level species. Ecosphere 10(11):e02923. 10.1002/ecs2.2923

Geraldi NR, Smyth AR, Piehler MF, Peterson CH. 2013. Artificial substrates enhance non-native macroalga and N_2 production. Biological Invasions 16(9):1819-31.

Gittman RK, Scyphers SB, Smith CS, Neylan IP, Grabowski JH. 2016. Ecological consequences of shoreline hardening: a meta-analysis. BioScience 66(9):763-73.

Global Invasive Species Database (GISD). 2020. [Internet]. Species profile: *Carcinus maenas*. (Accessed 3 August 2020). http://www.iucngisd.org/gisd/species.php?sc=114

Johnson MR, Boelke C, Chiarella LA, Colosi PD, Greene K, Lellis-Dibble K, Ludemann H, Ludwig M, McDermott S, Ortiz J, Rusanowsky D, Scott M, Smith J. 2008. Impacts to marine fisheries habitat from nonfishing activities in the northeastern United States. NOAA Technical Memorandum. National Oceanic and Atmospheric Administration, National Marine Fisheries Service. NMFS-NE-2O9. 1-328.

Kornis MS, Breitburg D, Balouskus R, Bilkovic DM, Davias LA, Giordano S, Heggie K, Hines AH, Jacobs JM,Jordan TE, King RS, Patrick CJ, Seitz RD, Soulen H, Targett TE, Weller DE, Whigham DF, Uphoff J. 2017. Linking the abundance of estuarine fish and crustaceans in nearshore waters to shoreline hardening and land cover. Estuaries and Coasts 40:1464–86. doi:10.1007/s12237-017-0213-6

Kroeker KJ, Kordas RL, Crim R, Hendriks IE, Ramajo L, Singh GS, Duarte CM, Gattuso J-P. 2013. Impacts ofocean acidification on marine organisms: quantifying sensitivities and interaction with warming. Global Change Biology 19(6):1884-96.

Lehnert SJ, DiBacco C, Jeffery NW, Blakeslee A, Isaksson J, Roman J, Wringe BF, Stanley R, Matheson K, McKenzie CH, Hamilton LC, Bradbury IR. 2018. Temporal dynamics of genetic clines of invasive European green crab (*Carcinus maenas*) in eastern North America. Evolutionary Applications 11(9):1656-70. https://doi.org/10.1111/eva.12657

Pappal A. 2010. Marine invasive species. State of the Gulf of Maine Report. [Currier P, Gould D, Hertz L, Huston J, Tremblay ML (eds)]. The Gulf of Maine Council on the Marine Environment. 1-21.

Scheibling RE, Gagnon P. 2006. Competitive interactions between the invasive green alga *Codium fragile* ssp. *tomentosoides* and native canopy-forming seaweeds in Nova Scotia (Canada). Marine Ecology Progress Series 325:1–14. DOI: 10.3354/meps325001.

Sorte CJB, Williams SL, Zerebecki RA. 2010. Ocean warming increases threat of invasive species in a marine fouling community. Ecology 91(8):2198-204.

Stachowicz JJ, Terwin JR, Whitlatch RB, Osman RW. 2002. Linking climate change and biological invasions: ocean warming facilitates nonindigenous species invasions. Proceedings of the National Academy of Sciences 99(24):15497-500.

Steneck R, Graham MH, Bourque BJ, Corbett D, Erlandson JM, Estes JA, Tegner MJ. 2002. Kelp forest ecosystems: biodiversity, stability, resilience and future. Marine Sciences Faculty Scholarship. 65. https://digitalcommons.library.umaine.edu/sms_facpub/65

Stevenson D, Chiarella L, Stephan D, Reid R, Wilhelm K, McCarthy J, Pentony M. 2004. Characterization of the fishing practices and marine benthic ecosystems of the Northeast U.S. Shelf, and an evaluation of the

potential effects of fishing on Essential Fish Habitat. NOAA Technical Memorandum NMFS-NE-181. 179 p.

Trott TJ, Enterline C. 2019. First record of the encrusting bryozoan *Cribrilina* (*Juxtacribrilina*) *mutabilis* (Ito, Onishi and Dick, 2015) in the Northwest Atlantic Ocean. BioInvasions Records 8.

Tyrrell MC, Byers JE. 2007. Do artificial substrates favor nonindigenous fouling species over native species? Journal of Experimental Marine Biology and Ecology 342(1):54-60.

U.S. Global Change Research Program (USGCRP). 2017. Climate Science Special Report: Fourth National Climate Assessment. Volume I. [Wuebbles, D.J., D.W. Fahey, K.A. Hibbard, D.J. Dokken, B.C. Stewart, and T.K. Maycock (eds.)]. U.S. Global Change Research Program, Washington, DC. USA. 470 pp. doi: 10.7930/J0J964J6.

Valentine P. 2009. Larval recruitment of the invasive colonial ascidian *Didemnum vexillum*, seasonal water temperatures in New England coastal and offshore waters, and implications for spread of the species. Aquatic Invasions 4(1):153-68.

Williams GD, Thom RM. 2001. Marine and estuarine shoreline modification issues. [Internet]. White Paper submitted to Washington Department of Fish and Wildlife, Washington Dept. of Ecology, Washington Dept. of Transportation. Sequim (WA): Battelle Memorial Institute. 99 p.

Estuarine Shellfish Aquaculture

System: Estuarine

Subsystem: Subtidal & Intertidal

Class: Reef

Sub-class: Mollusk Aquaculture

Geographic Area: Entire Area

Overall Vulnerability Rank = Moderate

Habitat Sensitivity = Moderate

Climate Exposure = High

	Estuarine Shellfish Aquaculture	Attribute Mean	Data Quality	Distribution of Expert Scores
Sensitivity Attributes	Habitat condition	2	2	
	Habitat fragmentation	1.8	1.6	
	Distribution/Range	2	1.6	
	Mobility/Ability to spread or disperse	1.8	1.3	
	Resistance	2.4	1.6	
	Resilience	2.1	1.8	
	Sensitivity to changes in abiotic factors	2.8	2.1	
	Sensitivity and intensity of non−climate stressors	2.8	1.6	
	Dependency on critical ecological linkages	2.6	1.6	
	Sensitivity Component Score	Moderate		
Exposure Factors	Sea surface temp	3.7	2.5	
	Bottom temp	n/a	n/a	
	Air temp	n/a	n/a	
	River temp	n/a	n/a	
	Surface salinity	2.7	2.1	
	Bottom salinity	n/a	n/a	
	pH	4	2	
	Sea level rise	2.9	2.2	
	Precipitation	2.6	2.1	
	Floods	n/a	n/a	
	Droughts	n/a	n/a	
	Exposure Component Score	High		
**Overall Vulnerability Rank**		**Moderate**		

Low

Moderate

High

Very High

Habitat Name: Estuarine Shellfish Aquaculture

System: Marine

Subsystem: Subtidal and Intertidal

Class: Reef

Sub-class: Mollusk Aquaculture

Geographic Area: Entire Area

<u>Habitat Description</u>: This habitat sub-class is focused specifically on aquaculture of molluscan shellfish conducted in intertidal and subtidal nearshore estuarine environments in the study area. Shellfish farms vary widely in spatial scale, type of aquaculture practiced, and target species. Eastern oysters (*Crassostrea virginica*) may be cultivated on leased seafloor using traditional on-bottom culture or using gear-based methods such as floating bags, or on-bottom cages. Blue mussels (*Mytilus edulis*) are reared on long-lines placed vertically between the sea surface and sediments. Northern hard clams (*Mercenaria mercenaria*) are harvested from seafloor sediments. Small-scale aquaculture of bay scallops (*Argopecten irradians*) in underway using lantern nets. Oysters and hard clams are cultivated intensively in Long Island Sound and the Chesapeake Bay while mussels are primarily cultured in the Gulf of Maine (MacKenzie et al. 1997). Molluscan aquaculture is practiced in the same waters where natural populationsof shellfish occur. This sub-class also includes artificial shellfish reefs (e.g., concrete oyster "reef balls") that are used for shellfish restoration. In addition, non-native species are sometimes cultured in the aquaculture industry (e.g., European oyster, *Ostrea edulis*, in Maine).

<u>Overall Climate Vulnerability Rank</u>: Moderate (97% certainty from bootstrap analysis).

<u>Climate Exposure</u>: High. Two attributes, pH and Sea Surface Temperature, contributed to the overall High exposure score: 4.0 and 3.7, respectively. Sea Level Rise (SLR), Surface Salinity and Precipitation scored moderately High (2.9, 2.7, and 2.6, respectively). Ocean acidification, based on projected declines in pH, is expected to increase in the region. Projected changes in sea surface temperature were high throughout the range but were greatest in the Gulf of Maine. SLR is expected to impact the entire study area but the greatest relative rise is projected for the Mid-Atlantic. Surface salinity is also anticipated to decline, most notably in the Gulf of Maine. Extreme precipitation events are projected to increase in frequency and intensity throughout the region.

<u>Habitat Sensitivity</u>: Moderate. Seven of the nine sensitivity attributes scored in the Moderate to High range at 2.0 or above, while two scored Low (<2.0). The highest sensitivity scores were Sensitivity to Changes in Abiotic Factors and Sensitivity and Intensity of Non−Climate Stressors (both 2.8), and Dependency on Critical Ecological Linkages (2.6). The Moderate habitat sensitivity mean for the aquaculture sub-class, compared to the intertidal and subtidal shellfish mollusk habitats that scored high, likely reflects the view that cultured shellfish are actively placed into areas that are generally conducive for growth and survival, and that aquaculture gear is generally mobile and can be relocated if conditions are less favorable. Careful site selection may help mitigate effects of abiotic and non-climate factors that affect aquaculture shellfish operations (e.g., dredging, harmful algal blooms, shoreline hardening, invasive species, marine construction, and pollution).

<u>Data Quality & Gaps</u>: Data quality for climate exposure factors fell in the Moderate range (2.0 to 2.5), indicating limited information. Data quality is lower for estuarine habitats due to the low resolution of CMIP5 and ROMS-NWA projections for climate exposure in estuaries and nearshore, shallow coastal areas. Another uncertainty factor is the relative disparate spatial data for aquaculture mollusk reefs in some portions of the study area. In both cases, data for shellfish species naturally occurring in estuarine habitats were referenced.

Data quality scores were low (≤ 2.1) for all nine sensitivity attributes. Lack of information specific to shellfish aquaculture habitats required that expert judgement also be applied in scoring.

Generally, shellfish aquaculture as a habitat type has not been implicitly studied and further research is needed

to understand how shellfish on farms or contained in aquaculture gear may be affected by climate and other factors. For example, although the climate sensitivity for estuarine mollusk species generally are believed to be high, there remains some uncertainty in species' capacity to adapt and respond to climate change (e.g., synergistic effects of climate and non-climate stressors).

Positive or Negative Climate Effect in the Northeast U.S. Shelf: The effect of climate change on estuarine shellfish aquaculture habitat is expected to be neutral or negative (60% of the expert's scores were neutral, 40% negative) and may reflect the high tolerance of estuarine mollusks to changing environmental conditions. This score also takes into account the potential for growers to shift operations away from suboptimal environments, although economic and siting limitations (e.g., availability of bottom leases) may require that cultured mollusks remain in areas subject to climate effects. For this reason, cultured shellfish may experience exposure to warming seawater temperatures, rising sea level,and declining pH, as reflected in a 40% negative score.

Climate Effects on Habitat Condition and Distribution: Shellfish aquaculture habitats are projected to experience high exposure and moderate sensitivity to ocean acidification, reduced pH and increased seawater temperatures in the New England and mid-Atlantic regions.

Many laboratory studies have examined effects of ocean acidification on bivalve mollusk species (oysters, blue mussels, hard clams) that are commercially cultivated in nearshore coastal estuaries. These studies have documented negative effects of ocean acidification on calcification, growth, and survival of mollusks (e.g., Gazeau et al. 2013; Ekstrom et al. 2015; Clements and Chopin 2016). Responses are species-specific, with larvae and juveniles more vulnerable to ocean acidification than adults (e.g., Kurihara 2008; Kroeker et al. 2013; Gledhill et al. 2015). Lower pH levels can make it difficult for calcifying mollusks to produce shells (Clements and Chopin 2016). Shellfish in estuaries are more susceptible to ocean acidification than their counterparts in marine environments, since estuarine systems have more sources of acid input and are less buffered than oceanic waters (Waldbusser et al. 2011). The synergistic effects of temperature, salinity, and pH on metabolism in marine mollusks may begreater than reduced pH alone.

Increasing seawater temperatures may affect the condition and distribution of shellfish aquaculture species in estuaries. For example, southern populations of blue mussels appear to be shifting northward in response to warming temperatures (Jones et al. 2010). Thermal stress, in conjunction with ocean acidification conditions, was found to cause metabolic depression in blue mussels from the Gulf of Maine (Lesser 2016), suggesting that elevated temperatures may result in suboptimal growing conditionsfor this cold-adapted species. Warming seawater temperatures can increase susceptibility of shellfish to disease, parasites and predation by local and invasive species (Smolowitz 2013; Burge et al. 2014).

Moderate exposure and sensitivity to projected sea level rise and changes in surface salinity may influence availability of intertidal habitat and could affect suitability of nearshore habitat for shellfish aquaculture in estuaries. Sustained sea level rise may permanently inundate intertidal habitat while extreme or sudden changes in salinity from storms and increased precipitation could alter habitat quality for shellfish.

Cultured estuarine mollusks are resilient and tolerate variability in environmental conditions. Growers closely manage shellfish beds and aquaculture gear to maintain condition and avoid loss or damage. For this reason, the effects of climate change on the condition and distribution of shellfish aquaculture habitat are largely expected to be neutral. Molluscan aquaculture is subject to many of the same influences as natural shellfish habitats and careful site selection may help to mitigate effects of ocean acidification and other climate-related variables on shellfish (Clements and Chopin 2016; Snyder et al. 2017).

Habitat Summary: Shellfish and associated aquaculture gear creates hard structure that provides habitat for a variety of sessile colonizers, mobile fish and invertebrates while mollusks often serve as keystone species in these habitats. Reef-building bivalves like oysters and mussels create biotic multi-dimensional structured habitat on the seafloor. Filter-feeding bivalves consume naturally occurring phytoplankton and particulates

from the water column and provide an important link between benthic seafloor communities and primary productivity in the water column (Shumway et al. 2003).

Cultivated mollusks are exposed to a variety of non-climate and abiotic stressors in estuarine environments that may act synergistically with climate effects. Episodic storms can increase physical or mechanical disturbance and disrupt shellfish farms, resulting in loss of animals and aquaculture gear. Inundation from storm events can lead to increased siltation in areas where muddy and silty sediments are present.

Shellfish, although tolerant of salinity fluctuations, may be affected by extreme or sudden changes in salinity following extreme storm and precipitation events, especially in combination with ocean acidification (Dickinson et al. 2012). Bay scallops show increased sensitivity to reduced salinity at elevated seawater temperatures (Mercaldo and Rhodes 1982). Mollusks are highly sensitive to harmful algal blooms, which have increased in frequency with warming seawater temperatures (Gobler et al. 2017). Eutrophication and over enrichment can increase exposure of shellfish to harmful algal blooms and reduced dissolved oxygen in coastal estuaries (Howarth et al. 2011). Shellfish diseases, such as Dermo and MSX, have impacted both natural and cultured shellfish reefs across the northeast coastal region. High temperature and low salinity are known drivers for both MSX and Dermo (Burreson et al. 2000; Ford and Smolowitz 2007; Burge et al. 2014). Between 1990 and 1992, a dramatic range extensionof Dermo disease was reported over a 500 km area in the northeastern United States from Delaware Bay, New Jersey to Cape Cod Bay, Massachusetts (Ford and Smolowitz 2007). By 1995, Dermo was reported in oysters as far north as Maine (Burge et al. 2014).

Pollution or chemical contaminants from anthropogenic activity may degrade habitat around shellfish farms. Farmed shellfish and associated gear may have some capacity to respond to suboptimal conditions, but availability of leased grounds and economic considerations can limit the ability of aquaculture operations to respond to changes in habitat quality (Allison et al. 2011). Site selection will play an important role in mitigating the effects of climate on estuarine shellfish aquaculture (Clements and Chopin 2016).

Literature Cited

Allison EH, Badjeck M-C, Meinhold K. 2011. The implications of global climate change for molluscan aquaculture. Chapter 17, pp. 461-490. *In*: Shellfish Aquaculture and the Environment (SE Shumway, ed.) John Wiley & Sons, Inc.

Burge CA, Eakin CM, Friedman CS, Froelich B, Hershberger PK, Hofmann EE, Petes LE, Prager KC, Weil E, Willis BL, Ford SE, Harvell CD. 2014. Climate change influences on marine infectious diseases: implications for management and society. Annual Review of Marine Science 6:249-77.

Burreson EM, Stokes NA, Friedman CS. 2000. Increased virulence in an introduced pathogen: *Haplosporidium nelsoni* (MSX) in the eastern oyster *Crassostrea virginica*. Journal of Aquatic Animal Health 12(1):1-8.

Clements JC, Chopin T. 2016. Ocean acidification and marine aquaculture in North America: potential impacts and mitigation strategies. Reviews in Aquaculture 9(4):326-41.

Dickinson GH, Ivanina AV, Matoo OB, Pörtner HO, Lannig G, Bock C, Beniash E, Sokolova IM. 2012. Interactive effects of salinity and elevated CO_2 levels on juvenile eastern oysters, *Crassostrea virginica.* Journal of Experimental Biology 215:29-43.

Ekstrom JA, Suatoni L, Cooley SR, Pendleton LH, Waldbusser GG, Cinner JE, Portela R. 2015. Vulnerability and adaptation of US shellfisheries to ocean acidification. Nature Climate Change 5(3):207-14.

Ford SE, Smolowitz R. 2007. Infection dynamics of an oyster parasite in its newly expanded range. Marine Biology 151(1):119-33.

Gazeau F, Parker LM, Comeau S, Gattuso J-P, O'Connor WA, Martin S, Pörtner HO, Ross PM. 2013. Impacts of ocean acidification on marine shelled molluscs. Marine Biology 160:2207-45.

Gledhill DK, White MM, Salisbury J, Thomas H, Misna I, Liebman M, Mook B, Grear J, Candelmo AC, Chambers RC, Gobler CJ, Hunt C., King A., Price NN, Signorini SR, Stancioff E, Stymiest C, Wahle RA, Waller JD, Rebuck ND, Wang ZA, Capson DL, Morrison JR, Cooley SR, Doney SC. 2015. Ocean and coastal acidification off New England and Nova Scotia. Oceanography 28(2):182-97.

Gobler CJ, Doherty OM, Hattenrath-Lehmanna TK, Griffith AW, Kanga Y, Litaker RW. 2017. Ocean warming since 1982 has expanded the niche of toxic algal blooms in the North Atlantic and North Pacific oceans. Proceeding National Academy of Science 114 (19):4975-80.

Howarth R, Chan F, Conley DJ, Garnier J, Doney SC, Marino R, Billen G. 2011. Coupled biogeochemical cycles: eutrophication and hypoxia in temperate estuaries and coastal marine ecosystems. Frontiers in Ecology and the Environment 9(1):18-26.

Jones SJ, Lima FP, Wethey DS. 2010. Rising environmental temperatures and biogeography: poleward range contraction of the blue mussel, *Mytilus edulis* L., in the western Atlantic. Journal of Biogeography 37(12):2243-59

Lesser MP. 2016. Climate change stressors cause metabolic depressions in the blue mussel, *Mytilus edulis*, from the Gulf of Maine. Limnology and Oceanography 61:1705-17.

Kroeker KJ, Kordas RL, Crim R, Hendriks IE, Ramajo L, Singh GS, Duarte CM, Gattuso J-P. 2013. Impacts of ocean acidification on marine organisms: quantifying sensitivities and interaction with warming. Global Change Biology 19(6):1884-96.

Kurihara, H. 2008. Effects of CO_2 driven ocean acidification on the early developmental stages of invertebrates. Marine Ecology Progress Series 373:275-84.

MacKenzie Jr CL, Burrell Jr VG, Rosenfield A, Hobart WL. (eds.). 1997. The history, present condition, and future of the molluscan fisheries of North and Central America and Europe, Volume 1, Atlantic and Gulf Coasts. U.S. Department of Commerce, NOAA Technical Report 127:1-234.

Mercaldo RS, Rhodes EW. 1982. Influence of reduced salinity on the Atlantic bay scallop *Argopecten irradians* (Lamarck) at various temperatures. Journal of Shellfish Research 2(2):177-81.

Shumway SE, David C, Downey R, Karney R, Kraeuter J, Parson J, Rheault R, Wikfors G. 2003. Shellfish aquaculture—In praise of sustainable economies and environments. World Aquaculture Society34(4):15-8.

Smolowitz R. 2013. A review of current state of knowledge concerning *Perkinsus marinus* effects on *Crassostrea virginica* (Gmelin) (the Eastern oyster). Veterinary Pathology 50(3):404-11.

Snyder J, Boss E, Weatherbee R, Thomas AC, Brady D and Newell C. 2017. Oyster aquaculture site selection using Landsat 8-derived sea surface temperature, turbidity, and chlorophyll a. Frontiers of Marine Science 4:190. doi: 10.3389/fmars.2017.00190

Waldbusser GG, Voigt EP, Bergschneider H, Green MA, Newell RIE. 2011. Biocalcification in the eastern oyster (*Crassostrea virginica*) in relation to long-term trends in Chesapeake Bay pH. Estuaries and Coasts 34:221-31.

Estuarine Subtidal Artificial Structures

System: Estuarine

Subsystem: Subtidal

Class: Rocky Bottom

Sub-class: Artificial Structures

Geographic Area: Entire Area

Overall Vulnerability Rank = Low

Habitat Sensitivity = Low

Climate Exposure = High

	Estuarine Subtidal Artificial Structures	Attribute Mean	Data Quality	Distribution of Expert Scores
Sensitivity Attributes	Habitat condition	2	1.9	
	Habitat fragmentation	1.8	1.5	
	Distribution/Range	2	1.5	
	Mobility/Ability to spread or disperse	1.4	1.5	
	Resistance	1.4	1.3	
	Resilience	1.7	1.8	
	Sensitivity to changes in abiotic factors	1.8	1.8	
	Sensitivity and intensity of non−climate stressors	1.9	1.9	
	Dependency on critical ecological linkages	1.5	1.6	
	Sensitivity Component Score	Low		
Exposure Factors	Sea surface temp	3.7	2.5	
	Bottom temp	1	0	
	Air temp	1	0	
	River temp	1	0	
	Surface salinity	2.3	2.1	
	Bottom salinity	1	0	
	pH	4	2	
	Sea level rise	2.2	2.2	
	Precipitation	1	0	
	Floods	1	0	
	Droughts	1	0	
	Exposure Component Score	High		
**Overall Vulnerability Rank**		**Low**		

Low
Moderate
High
Very High

Habitat Name: Estuarine Subtidal Artificial Structures

System: Estuarine

Subsystem: Subtidal

Class: Rocky Bottom

Sub-class: Artificial Structures

Geographic Area: Entire Area

Habitat Description: This sub-class includes artificial reefs and wrecks, riprap, groins, and breakwaters inthe estuarine subtidal zone. Riprap and breakwaters are common throughout the region in populated coastal areas. Artificial reefs are generally more common in the Mid-Atlantic than New England region, although riprap, groins, and breakwaters are generally found throughout the study area. This sub-class includes the substrate, as well as the epibenthic flora and fauna associated with these structures, but does not include specific habitats that were assessed separately (i.e., non-calcareous algae). This sub-class does include calcareous algae, however.

Overall Climate Vulnerability Rank: Low (100% certainty from bootstrap analysis).

Climate Exposure: High. Two exposure factors contributed to the High exposure score: Sea Surface Temperature (3.7) and pH (4.0). Projected changes pH are very high throughout the range (increasing ocean acidification). Projected increases in sea surface temperature are high to very high throughout therange, but slightly greater in the Gulf of Maine.

Habitat Sensitivity: Low. Most sensitivity attributes were in the Low scoring bins. No sensitivity attributes scored above 2.0. The highest scoring attributes included Habitat Condition (2.0), Sensitivity and Intensity of Non−Climate Stressors (1.9), and Sensitivity to Changes in Abiotic Factors and Habitat Fragmentation (both 1.8).

Data Quality & Gaps: Three of the four climate exposure factors had relatively Low data quality scores (≤2.2), while the highest (Sea Surface Temperature) was 2.5. In addition, data quality is generally lower for estuarine habitats due to the low resolution of CMIP5 and ROMS-NWA projections for climate exposure in estuaries and nearshore, shallow coastal areas, which increases the uncertainty with overlap in climate exposure projections.

All nine of the sensitivity attribute data quality scores were ≤2.0 (ranging from 1.3 to 1.9). The low data quality scores may indicate uncertainty in the expected responses of fauna and flora associated with thehabitat.

Information about the distribution of artificial structures is limited, not consolidated, or easily accessible. The diversity of artificial structure types included in this habitat also make it difficult toassess climate vulnerability in a uniform manner.

Positive or Negative Climate Effect in the Northeast U.S.: The effect of climate change on estuarine artificial subtidal hard bottom in the Northeast U.S. is generally expected to be neutral (75% of the experts' scores were neutral, 15% were negative, and 10% were positive). The divergence in directionaleffects scores may be due to the diversity of artificial structure types included in this single habitat type.

Climate Effects on Habitat Condition and Distribution: Artificial subtidal hard structures are not expected to be significantly impacted by climate change compared to natural habitats. Structures like riprap and jetties are designed to deflect, withstand, or absorb wave action. However, artificial reefs may be impacted by larger and more intense storms. Some of the flora and fauna associated with subtidal artificial structures may be sensitive to climate impacts including increasing temperatures, changingsalinity, pH, and high wave energy.

As ocean acidification increases, echinoderms associated with artificial rocky bottom habitats will experience

negative effects on their growth and survival. Kroeker et al. (2013) reported the largest effects on growth for larval life stages in echinoderms. In addition, this study found calcareous algae is expected to be negatively affected by ocean acidification by lower abundance, and rates of photosynthesis and calcification.

Increased shoreline hardening in heavily populated and industrialized areas is expected as climate change effects become more severe and widespread, in an attempt to reduce erosion and provide protection from storm surge (Balouskas and Targett 2018). This would increase the availability of artificial structures for flora and fauna associated with them, particularly for non-native and invasive species that may benefit by warming coastal waters and greater disturbances (Stachowicz et al. 2002; Valentine 2009; Sorte et al. 2010).

Habitat Summary: The condition of artificial hard structures, in terms of habitat function, is inherently poor, with lower species diversity, habitat complexity, and habitat function compared with natural rocky habitats (Bulleri and Chapman 2010; Balouskas and Targett 2018). The make-up of biological communities on riprap, breakwaters, and other artificial structures differs from natural rocky habitats (Bulleri and Chapman 2010). The low structural complexity of engineered stone structures may limit habitat value for mobile species. Low species diversity may limit the resilience of the biotic community associated with artificial structures.

The material and design of artificial structures are important for their function as habitat, and the biodiversity of species they support. In the intertidal zone, for example, sandstone has been found to support a higher diversity of species than concrete and low structural complexity may limit the value ofartificial structures for mobile species (Browne and Chapman 2017). Incorporating natural habitat elements into artificial shoreline structures can improve habitat function (Bulleri and Chapman 2010). The presence of artificial hard structures in the Mid-Atlantic Bight has increased over the last two centuries, with shipwrecks constituting one of the most abundant types of artificial reef habitat (Steimle and Zetlin 2000; NOAA 2020). While patchy in distribution, some of these structures support biological communities including invertebrates, algae, and fish (Steimle and Zetlin 2000).

Flora and fauna associated with artificial rocky habitat may be sensitive to pollution and eutrophication similar to natural habitats, although invasive species may be less sensitive. Some studies suggest a higher incidence of invasive species associated with artificial structures compared to native material (Tyrrell and Byers 2007; Pappal 2010; Geraldi et al. 2013). This may be associated with greater survival of invasive species on disturbed areas, but it could also suggest higher planktonic stage survival and greater dispersal capacity.

Artificial hard structures are highly resistant to disturbance. In fact, many shoreline structures are built to protect shorelines and withstand disturbance. Jetties and groins are common structures built to control erosion, but are known to interfere with natural sediment transport and can often exacerbate erosion in the coastal system. This can impact adjacent soft-bottom (i.e., sand and mud) habitats (Williams and Thom 2001).

Non-mobile (fouling) species that attach to riprap and rocky breakwaters re-colonize quickly following disturbance. Artificial reef habitats may be sensitive to siltation and burial, damage from fishing gear, pollution, removal, and water quality degradation (Steimle and Zetlin 2000).

Literature Cited

Balouskus RG, Targett TE. 2018. Impact of armored shorelines on shore-zone fish density in a Mid-Atlantic estuary: modulation by hypoxia and temperature. Estuaries and Coasts 41(Suppl1):S144-58.

Browne MA, Chapman MG. 2017. The ecological impacts of reengineering artificial shorelines: The state of the science. *In:* Bilkovic DM, Mitchell MM, La Peyre MK, Toft JD (Eds). Living shorelines: the science and management of nature-based coastal protection (First Edition, 439-57). Boca Raton. https://doi.org/10.1201/9781315151465.

Bulleri F, Chapman MG. 2010. The introduction of coastal infrastructure as a driver of change in marine environments. Journal of Applied Ecology 47:26-35.

Geraldi NR, Smyth AR, Piehler MF, Peterson CH. 2013. Artificial substrates enhance non-native macroalga and N_2 production. Biological Invasions 16(9):1819-31.

Kroeker KJ, Kordas RL, Crim R, Hendriks IE, Ramajo L, Singh GS, Duarte CM, Gattuso J-P. 2013. Impacts of ocean acidification on marine organisms: quantifying sensitivities and interaction with warming. Global Change Biology 19(6):1884-96.

National Oceanic and Atmospheric Administration (NOAA). 2020. [Internet]. Coasts Survey's Wrecks and Obstructions. Automated Wreck and Obstruction Information System (AWOIS). (Accessed September 2020). https://wrecks.nauticalcharts.noaa.gov/viewer/.

Pappal A. 2010. Marine invasive species. State of the Gulf of Maine Report. [Currier P, Gould D, Hertz L, Huston J, Tremblay ML (eds)]. The Gulf of Maine Council on the Marine Environment. 1-21.

Steimle FW, Zetlin C. 2000. Reef Habitats in the Middle Atlantic Bight: Abundance, Distribution, Associated Biological Communities, and Fishery Resource Use. Marine Fisheries Review 62(2):24-42.

Sorte CJB, Williams SL, Zerebecki RA. 2010. Ocean warming increases threat of invasive species in a marine fouling community. Ecology 91(8):2198-204.

Stachowicz JJ, Terwin JR, Whitlatch RB, Osman RW. 2002. Linking climate change and biological invasions: ocean warming facilitates nonindigenous species invasions. Proceedings of the National Academy of Sciences 99(24):15497-500.

Tyrrell MC, Byers JE. 2007. Do artificial substrates favor nonindigenous fouling species over native species? Journal of Experimental Marine Biology and Ecology 342(1):54-60.

Valentine P. 2009. Larval recruitment of the invasive colonial ascidian *Didemnum vexillum*, seasonal water temperatures in New England coastal and offshore waters, and implications for spread of the species. Aquatic Invasions 4(1):153-68.

Waldbusser GG, Voigt EP, Bergschneider H, Green MA, Newell RIE. 2011. Biocalcification in theeastern oyster (*Crassostrea virginica*) in relation to long-term trends in Chesapeake Bay pH. Estuaries and Coasts 34(2):221-31.

Williams GD, Thom RM. 2001. Marine and estuarine shoreline modification issues. [Internet]. White Paper submitted to Washington Department of Fish and Wildlife, Washington Dept. of Ecology,Washington Dept. of Transportation. Sequim (WA): Battelle Memorial Institute. 99 p.

Estuarine Subtidal Mud Bottom

System: Estuarine

Subsystem: Subtidal

Class: Unconsolidated Bottom

Sub-class: Mud

Geographic Area: Entire Area

Overall Vulnerability Rank = Moderate

Habitat Sensitivity = Moderate

Climate Exposure = High

	**Estuarine Subtidal Mud Bottom**	Attribute Mean	Data Quality	Distribution of Expert Scores
Sensitivity Attributes	Habitat condition	2.9	2.6	
	Habitat fragmentation	1.8	2	
	Distribution/Range	1.4	2.1	
	Mobility/Ability to spread or disperse	1.6	1.7	
	Resistance	2.4	2.4	
	Resilience	2.2	2.1	
	Sensitivity to changes in abiotic factors	2.6	2.3	
	Sensitivity and intensity of non−climate stressors	2.8	2.4	
	Dependency on critical ecological linkages	2.4	1.7	
	Sensitivity Component Score	Moderate		
Exposure Factors	Sea surface temp	3.7	2.5	
	Bottom temp	n/a	n/a	
	Air temp	n/a	n/a	
	River temp	n/a	n/a	
	Surface salinity	2.6	2.1	
	Bottom salinity	n/a	n/a	
	pH	4	2	
	Sea level rise	2.2	2.2	
	Precipitation	n/a	n/a	
	Floods	n/a	n/a	
	Droughts	n/a	n/a	
	Exposure Component Score	High		
**Overall Vulnerability Rank**		**Moderate**		

Low
Moderate
High
Very High

Habitat Name: Estuarine Subtidal Mud Bottom
System: Estuarine
Subsystem: Subtidal
Class: Unconsolidated Bottom
Sub-class: Mud
Geographic Area: Entire Area

Habitat Description: This sub-class includes subtidal mud substrate, as well as the epifauna and infauna associated with the bottom, such as non-reef-forming mollusks (e.g., soft-shell clams, hard clams, sea scallops, surf clams, ocean quahogs), marine worms, small crustaceans, gastropods, and polychaetes.

This subclass excludes specific habitats identified elsewhere (i.e., non-calcareous algal bed, rooted vascular beds, and reef-forming mollusks, including blue mussels, eastern oysters). Specifically, estuarine subtidal mud habitats tend to occur in less exposed areas that accumulate fine particles.

Overall Climate Vulnerability Rank: Moderate (97% certainty from bootstrap analysis).

Climate Exposure: High. The overall high exposure score was influenced by two variables: Sea Surface Temperature (3.7) and pH (4). The mean exposure scores for Surface Salinity and Sea Level Rise (SLR) were 2.6 and 2.2, respectively. In general, estuarine waters are expected to become more stratified due to high sea surface temperature and decreased surface salinity. The combination of increased susceptibility tohigh bottom water respiration, low total alkalinity freshwater from land based sources, and influx of acidified ocean water due to high atmospheric CO_2 is expected to increase the exposure of estuarine mud habitats to low oxygen and pH conditions.

Habitat Sensitivity: Moderate. None of the sensitivity attributes scored ≥3.0 with the highest scores for Habitat Condition (2.9) and Sensitivity and Intensity of Non−Climate Stressors (2.8). These relatively high scores are consistent with the projection that the habitat condition of estuarine subtidal mud is expected to be influenced by hypoxia, stormwater pollution, and coastal acidification. The other sensitivity resultsare consistent with a low to moderate concern that the Distribution/Range, Resistance, Resilience, and Habitat Fragmentation of estuarine subtidal mud habitats will be impacted from climate change.

Data Quality & Gaps: The data quality scores for the four climate exposure factors were pH (2.0), Surface Salinity (2.1), SLR (2.2), and Sea Surface Temperature (2.5). Data quality is generally lower for estuarine habitats due to the low resolution of CMIP5 and ROMS-NWA projections for climate exposure in estuaries and nearshore, shallow coastal areas. Nearshore shallow water pH dynamics are strongly controlled by production-respiration internal to the estuary, freshwater flow, oceanic exchange, and air-sea exchange.

For habitat sensitivity, Habitat Fragmentation, Distribution/Range, Mobility, Resilience, Dependency on Critical Ecological Linkages all score < 2.2. Although the overall sensitivity of subtidal mud substrate is projected to be moderate, the relatively low data quality scores may reflect uncertainty in the sensitivities of biota associated with mud habitats (i.e., infauna and epifauna) to climate change.

Positive or Negative Climate Effect in the Northeast U.S. Shelf: The effect of climate change on estuarine subtidal mud habitats in the Northeast U.S. Shelf is expected to be neutral to negative (80% of the experts' scores were neutral and 20% negative). The directionality analysis also appears consistent with the general assessment that these habitats are difficult to observe (low data quality scores) and are resilient systems subject to highly variable conditions (low to moderate scores in sensitivity attributes).

Climate Effects on Habitat Condition and Distribution: Climate change will primarily affect the biota associated with subtidal mud bottom habitats (habitat function), rather than the physical features of the habitat, whereas intertidal mud bottom is naturally more variable with respect to environmental factors and more

susceptible to physical disturbance caused by humans, storms, and SLR.

The distribution of estuarine subtidal mud habitats is unlikely to change significantly due to climate except for a relatively small expansion due to SLR. However, the condition of estuarine subtidal mud habitats may be significantly altered by a combination of physical changes in estuarine stratification and biogeochemical changes as altered by coastal hypoxia and acidification. In a review of potential climate impacts on the largest estuary in the Northeast US, the Chesapeake Bay, Najjar et al. (2010) conclude that increases in precipitation and surface temperature are likely to increase stratification, which in turn is strongly connected with the formation of hypoxia (Murphy et al. 2011). Indeed, our climate projections indicate that extreme precipitation in the Mid-Atlantic is expected to become more frequent and intense and warming in estuaries is expected to increase faster than adjacent marine habitats. The rate of respiration that causes hypoxia formation in the bottom waters of subtidal mud habitats of estuaries in the Northeast U.S. is also likely to be increased by increasing temperatures (Irby et al.2018). Using a fully coupled hydrodynamic-water quality modeling approach, Ni et al (2019) recently predicted a 10-30% increase in hypoxic volume for the Chesapeake Bay. Climate induced changes in stratification and respiration may make estuarine subtidal habitats in other areas of the Northeast, such as Long Island Sound, Narragansett Bay, and Massachusetts Bay, more susceptible to seasonal hypoxia. In eutrophied locations, excess respiration can also contribute to coastal acidification in Northeast U.S. subtidal mud habitats (Cai et al. 2011). Both hypoxia and coastal acidification are known to impact ecosystem production and biodiversity. To limit these phenomena, water quality managers will likely need to reassess nutrient load management, coastal development, and pollution control to account for changing receiving waters.

Mollusk species associated with mud infauna/epifauna (e.g., soft-shell clams, hard clams, sea scallops, surf clams, ocean quahogs, small crustaceans, gastropods) are generally sensitive to low pH, with larger negative effects on survival for larvae than adults (Kroeker et al. 2013). Ocean acidification is expected to reduce the shell size, thickness, calcification rates and growth, and survival of embryo and larval bivalves and gastropods (Gazeau et al. 2013).

Coastal waters are generally more susceptible to acidification than oceanic waters because they are subject to more acid sources and are generally less buffered than oceanic waters (i.e., differences in the amount of dissolved inorganic carbon, dissolved and particulate organic carbon, total alkalinity and nutrients from riverine and estuarine sources) (Waldbusser et al. 2011; Ekstrom et al. 2015; Gledhill et al. 2015). A long-term monitoring program in the Chesapeake Bay found statistically significant declines in daytime average pH from 1985 to 2008 within polyhaline waters, but not in mesohaline waters of the Bay (Waldbusser et al. 2011). Rivers in New England that have a combination of cool temperatures, low alkalinity, and runoff typically consisting of soils containing carbonic acid, a by-product of organic decomposition, have particularly low aragonite saturation state values (Salisbury et al. 2008). For example, in the Casco Bay during times of high discharges from the Kennebec River and down-welling (northerly) winds, acidic river waters with very low aragonite saturation state values have been recorded (Salisbury et al. 2008). Overall acidification is expected to increase, although estuaries also naturally experience daily fluxes in pH and some species acclimated to variability may be resilient to ocean acidification. Some studies have shown additive and synergistic negative effects on bivalves and gastropods from ocean acidification and low dissolved oxygen (Kroeker et al. 2013; Gobler et al. 2014; Clark and Gobler 2016; Gobler and Bauman 2016; Griffith and Gobler 2017). In addition, high nutrient levels and algal blooms can trigger reductions in dissolved oxygen in the nearshore water column and in sediments, which can have detrimental effects to infaunal and epifaunal species associated with sand habitats (Sharp et al. 1982; Brownlee et al. 2005).

Estuarine subtidal mud habitats may also experience some disturbance from storms, which are expected to increase in frequency and intensity in the Northeast region (USGCRP 2017), although generally less so than intertidal mud habitats.

Habitat Summary: Estuarine subtidal mud habitats are home to important biological communities of infaunal organisms, sponges, polychaetes, amphipods, mysids, and other organisms that can take advantage

of organic matter deposition in these relatively quiescent habitats. Because this type of habitat is so extensive in the Northeast US, there is generally a high degree of connectivity. That is, if there is a physical disturbance in any one location, there is often a strong source population in the surrounding areas that can recruit to the site of the perturbation when recovered. Rijnsdorp et al. (2018) also documented that the impact of physical disturbances (such as bottom trawling) increased in habitats with many long-lived species and that mud habitats tend to contain mostly short-lived species. For these reasons, many habitat impact models developed by expert panels tend to characterize these habitats as having relatively low susceptibility and fast recovery times (Grabowski et al. 2014; Smeltz et al. 2019).

However, the most pervasive climate related threat to these habitats are likely to be the indirect effectsof warming on stratification and respiration. By cutting off the bottom waters of estuarine subtidal areas from relatively oxygenated and low acidity surface waters, so called 'dead zones' of relatively high respiration, low dissolved oxygen, and high acidity may expand without significant offsetting of land derived nutrients that fuel eutrophication (Breitburg et al. 2018). Water quality management may become even more important for the protection of these habitats as climate change makes nutrients more impactful to ecosystems. Fortunately, with comprehensive habitat protection, these habitats have shown a tendency to recover relatively quickly even after extensive hypoxia has been ameliorated.

Estuarine subtidal mud habitats are also exposed to coastal development and other anthropogenic stressors. Coastal population density and agriculture are associated with higher eutrophication and contamination, which can disturb benthic habitat quality. According to the 2012 EPA Coastal Condition Report, sediment quality varies throughout the region with the poorest sediment quality in Great Bay, Narragansett Bay, Long Island sound, the NY/NJ Harbor, the Upper Delaware Estuary, and the western tributaries of the Chesapeake Bay (EPA 2012). Low sediment quality ratings were primarily driven by sediment contamination, which are mostly due to elevated levels of metals, polychlorinated biphenyls, and pesticides. Muddy sediments tend to provide greater surface area for sorption of both organic matter and the chemical pollutants that bind to organic matter. Aquaculture operations may also cause some disturbance to benthic habitats through the accumulation of nutrients, wastes, or sediment deposition, but good water flow and husbandry limit this accumulation (ASMFC 2020).

Dredging, shoreline stabilization (e.g., riprap revetment, bulkheads, jetties, groins), and beach nourishment can alter the depth and sediment characteristics, with subsequent changes in infauna and epifauna/epiflora. Riprap revetment can convert mud bottoms to large diameter, engineered stone in the upper subtidal zone. All hardened shorelines have the potential to erode shallow water subtidal mud bottom. Hardened shorelines have been shown to have lower abundance, biomass, and diversity of benthic prey and predators (Seitz et al. 2006; Morley et al. 2012) and can have higher incidence of marine exotic/invasive species compared to native material (Tyrrell and Byers 2007).

Literature Cited

Atlantic States Marine Fisheries Commission (ASMFC). 2020. Aquaculture: effects on fish habitat along the Atlantic Coast. Atlantic States Marine Fisheries Commission Habitat Management Series #16.

Breitburg D,Levin LA, Oschlies A, Grégoire M, Chavez FP, Conley DJ, Garçon V, Gilbert D, Gutiérrez D, Isensee K, Jacinto GS, Limburg KE, Montes I, Naqvi SWA, Pitcher GC, Rabalais NN, Roman MR, Rose KA, Seibel BA, Telszewski M, Yasuhara M, Zhang J. 2018. Declining oxygen in the global ocean and coastal waters. Science 359(6371):eaam7240.

Brownlee EF, Sellner SG, Sellner K.G. 2005. *Prorocentrum minimum* blooms: potential impacts on dissolved oxygen and Chesapeake Bay oyster settlement and growth. Harmful Algae 4 (3):593-602. ISSN 1568-9883. https://doi.org/10.1016/j.hal.2004.08.009.

Cai W-J, Hu X, Huang W-J, Murrell MC, Lehrter JC, Lohrenz SE, Chou W-C, Zhai W, Hollibaugh JT, Wang

Y, Zhao P, Guo X, Gundersen K, Dai M, Gong G-C. 2011. Acidification of subsurface coastal waters enhanced by eutrophication. Nature Geoscience 4(11):766-70.

Clark H, Gobler CJ. 2016. Diurnal fluctuations in CO2 and dissolved oxygen concentrations do not provide a refuge from hypoxia and acidification for early-life-stage bivalves. Marine Ecology Progress Series 558:43114. https://doi.org/10.3354/meps11852

Ekstrom JA, Suatoni L, Cooley SR, Pendleton LH, Waldbusser GG, Cinner JE, Ritter J, Langdon C, van Hooidonk R, Gledhill D, Wellman K, Beck MW, Brander LM, Rittschof D, Doherty C, Edwards PET, Portela R. 2015. Vulnerability and adaptation of US shellfisheries to ocean acidification. Nature Climate Change 5(3):207-14.

Gazeau F, Parker LM, Comeau S, Gattuso J-P, O'Connor WA, Martin S, Pörtner H-O, Ross PM. 2013. Impacts of ocean acidification on marine shelled molluscs. Marine Biology 160(8):2207-45.

Gledhill D, White M, Salisbury J, Thomas H, Misna I, Liebman M, Mook B, Grear J, Candelmo A, Chambers RC, Gobler C, Hunt C, King A, Price N, Signorini S, Stancioff E, Stymiest C, Wahle R, Waller J, Rebuck N, Wang Z, Capson T, Morrison JR, Cooley S, Doney S. 2015. Ocean and coastal acidification off New England and Nova Scotia. Oceanography 25(2):182-97.

Gobler CJ, Baumann H. 2016. Hypoxia and acidification in ocean ecosystems: coupled dynamics and effects on marine life. Biology Letters 12(5):20150976.

Gobler CJ, DePasquale EL, Griffith AW, Baumann H. 2014. Hypoxia and acidification have additive and synergistic negative effects on the growth, survival, and metamorphosis of early life stage bivalves. PLoS One 9(1):e83648.

Grabowski JH, Bachman M, Demarest C, Eayrs S, Harris BP, Malkoski V, Packer D, Stevenson D. 2014. Assessing the vulnerability of marine benthos to fishing gear impacts. Reviews in Fisheries Science & Aquaculture 22(2):142-55.

Griffith AW, Gobler CJ. 2017. Transgenerational exposure of North Atlantic bivalves to ocean acidification renders offspring more vulnerable to low pH and additional stressors. Scientific Reports 7:11394.

Irby ID, Friedrichs MAM, Da F, Hinson KE. 2018. The competing impacts of climate change and nutrient reductions on dissolved oxygen in Chesapeake Bay. Biogeosciences 15:2649-68. doi:10.5194/bg-15-2649-2018

Kroeker KJ, Kordas RL, Crim R, Hendriks IE, Ramajo L, Singh GS, Duarte CM, Gattuso J-P. 2013. Impacts of ocean acidification on marine organisms: quantifying sensitivities and interaction with warming. Global Change Biology 19(6):1884-96.

Morley SA, Toft JD, Hanson KM. 2012. Ecological effects of shoreline armoring on intertidal habitats of a Puget Sound urban estuary. Estuaries and Coasts 35(3):774-84.

Murphy RR, Kemp WM, Ball WP. 2011. Long-term trends in Chesapeake Bay seasonal hypoxia, stratification, and nutrient loading. Estuaries and Coasts 34(6):1293-309.

Najjar RG, Pyke CR, Adams MB, Breitburg D, Hershner C, Kemp M, Howarth R, Mulholland MR, Paolisso M, Secor D, Sellner K, Wardrop D, Wood R. 2010. Potential climate-change impacts on the Chesapeake Bay. Estuarine, Coastal and Shelf Science 86(1):1-20.

Ni W, Li M, Ross AC, Najjar RG. 2019. Large projected decline in dissolved oxygen in a eutrophic estuary due to climate change. Journal of Geophysical Research: Oceans 124(11):8271-89.

Rijnsdorp AD, Bolam S. G, Garcia C, Hiddink JG, Hintzen NT, van Denderen PD, van Kooten T. 2018. Estimating sensitivity of seabed habitats to disturbance by bottom trawling based on the longevity of benthic fauna. Ecological Applications 28(5):1302-12.

Salisbury J, Green M, Hunt C, Campbell J. 2008. Coastal acidification by rivers: a threat to shellfish? Eos, Transactions American Geophysical Union 89(50):513-28.

Seitz RD, Lipcius RN, Olmstead NH, Seebo MS, Lambert DM. 2006. Influence of shallow-water habitats and shoreline development on abundance, biomass, and diversity of benthic prey and predators in Chesapeake Bay. Marine Ecology Progress Series 326:11-27.

Sharp JH, Culberson CH, Church TM. 1982. The chemistry of the Delaware estuary. General considerations. Limnology and Oceanography 27. doi:10.4319/lo.1982.27.6.1015.

Smeltz TS, Harris BP, Olson JV, Sethi SA. 2019. A seascape-scale habitat model to support management of fishing impacts on benthic ecosystems. Canadian Journal of Fisheries and Aquatic Sciences 76(10):1836-44.

Tyrrell MC, Byers JE. 2007. Do artificial substrates favor nonindigenous fouling species over native species? Journal of Experimental Marine Biology and Ecology 342(1):54-60.

U.S. Environmental Protection Agency (EPA). 2012. National Coastal Condition Report IV. EPA-R-842-10-003. US Environmental Protection Agency, Washington D.C.

U.S. Global Change Research Program (USGCRP). 2017. Climate Science Special Report: Fourth National Climate Assessment. Volume I [Wuebbles, D.J., D.W. Fahey, K.A. Hibbard, D.J. Dokken, B.C. Stewart, and T.K. Maycock (eds.)]. U.S. Global Change Research Program. Washington, DC. USA. 470 pp. doi: 10.7930/J0J964J6.

Estuarine Subtidal Sand Bottom

System: Estuarine

Subsystem: Subtidal

Class: Unconsolidated Bottom

Sub-class: Sand

Geographic Area: Entire Area

Overall Vulnerability Rank = Low

Habitat Sensitivity = Low

Climate Exposure = High

	Estuarine Subtidal Sand Bottom	Attribute Mean	Data Quality	Distribution of Expert Scores
Sensitivity Attributes	Habitat condition	2	2.6	
	Habitat fragmentation	1.7	2	
	Distribution/Range	2	2.1	
	Mobility/Ability to spread or disperse	2	1.7	
	Resistance	1.6	2.4	
	Resilience	2	2.1	
	Sensitivity to changes in abiotic factors	2.1	2.3	
	Sensitivity and intensity of non−climate stressors	2.4	2.4	
	Dependency on critical ecological linkages	1.8	1.7	
	Sensitivity Component Score	Low		
Exposure Factors	Sea surface temp	3.6	2.5	
	Bottom temp	n/a	n/a	
	Air temp	n/a	n/a	
	River temp	n/a	n/a	
	Surface salinity	2.7	2.1	
	Bottom salinity	n/a	n/a	
	pH	4	2	
	Sea level rise	2.2	2.2	
	Precipitation	n/a	n/a	
	Floods	n/a	n/a	
	Droughts	n/a	n/a	
	Exposure Component Score	High		
**Overall Vulnerability Rank**		**Low**		

Low
Moderate
High
Very High

Habitat Name: Estuarine Subtidal Sand Bottom
System: Estuarine
Sub-System: Subtidal
Class: Unconsolidated Bottom
Sub-class: Sand
Geographic Area: Entire Area

Habitat Description: This habitat sub-class includes estuarine subtidal sand bottoms. The epifauna and infauna associated with estuarine sand bottom, such as non-reef-forming mollusks (e.g., soft-shell clams,hard clams, sea scallops, surf clams, ocean quahogs), marine worms, small crustaceans, gastropods, and polychaetes, are a component of the habitat. This sub-class excludes specific habitats identified elsewhere (i.e., non-calcareous algal bed, rooted vascular beds, and reef-forming mollusks such as blue mussels, eastern oysters). Sandy subtidal habitats occur in estuaries throughout the study area, though are more frequent in the Mid-Atlantic. Lower parts of estuaries tend to be sandier, while the upper extent of estuaries is muddier.

Overall Climate Vulnerability Rank: Low (100% certainty from bootstrap analysis).

Climate Exposure: High. Two exposure factors contributed to the High exposure score: Sea Surface Temperature (3.6) and pH (4.0). Sea surface temperature and ocean acidification (decreasing pH) are projected to increase significantly throughout the region. Surface Salinity (2.7) also had a moderately-high score, and is projected to increase significantly in Mid-Atlantic estuaries, where sand habitats are more common, although decline slightly in New England estuaries. Consequently, scores for Surface Salinity were placed in all scoring bins to reflect this regional variability.

Habitat Sensitivity: Low. Most sensitivity attributes were in the Low and Moderate scoring bins. No sensitivity attributes scored above 3.0, with the highest scores for Sensitivity and Intensity of Non-Climate Stressors (2.4) and Resilience (2.4) followed by Habitat Condition (2.1).

Data Quality & Gaps: The data quality scores for the three exposure factors ranged from 2.0 (pH) to 2.5 (Sea Surface Temperature). Data quality is lower for estuarine habitats due to the low resolution of CMIP5 and ROMS-NWA projections for climate exposure in estuaries and nearshore, shallow coastal areas, which increases the uncertainty with overlap in climate exposure projections.

Three of the nine sensitivity attribute data quality scores were ≤2.0: Dependency on Critical Ecological Linkages (1.7), Mobility/Ability to Spread or Disperse (1.7), and Habitat Fragmentation (2.0). Although the overall sensitivity of subtidal sand substrate was projected to be low, the relatively low data quality scores may reflect uncertainty in the sensitivities of biota associated with sand (i.e., infauna and epifauna) to climate change.

Positive or Negative Climate Effect in the Northeast U.S.: The effect of climate change on estuarine subtidal sand in the Northeast U.S. is expected to be neutral (85% of the experts' scores were neutral,15% were negative). Climate change is expected to have relatively minor impacts on this habitat.

Climate Effects on Habitat Condition and Distribution: Climate change will primarily affect the biota associated with subtidal sand bottom habitats (habitat function), rather than the physical features of the habitat, whereas intertidal sand bottom is naturally more variable with respect to environmental factors and more susceptible to physical disturbance caused by humans, storms, and sea level rise.

Mollusk species associated with sand infauna/epifauna (e.g., soft-shell clams, hard clams, sea scallops, surf clams, ocean quahogs, small crustaceans, gastropods) are generally sensitive to low pH, with larger negative effects on survival for larvae than adults (Kroeker et al. 2013). Ocean acidification is expected to reduce the shell size, thickness, calcification rates and growth, and survival of embryo and larval bivalves and gastropods (Gazeau et al. 2013).

Coastal waters are generally more susceptible to acidification than oceanic waters because they are subject to more acid sources and are generally less buffered than oceanic waters (i.e., differences in the amount of dissolved inorganic carbon, dissolved and particulate organic carbon, total alkalinity and nutrients from riverine and estuarine sources) (Waldbusser et al. 2011; Ekstrom et al. 2015; Gledhill et al. 2015). A long-term monitoring program in the Chesapeake Bay found statistically significant declines in daytime average pH from 1985 to 2008 within polyhaline waters, but not in mesohaline waters of the Bay (Waldbusser et al. 2011). Rivers in New England that have a combination of cool temperatures, low alkalinity, and runoff typically consisting of soils containing carbonic acid, a by-product of organic decomposition, have particularly low aragonite saturation state values (Salisbury et al. 2008). For example, in the Casco Bay during times of high discharges from the Kennebec River and down-welling (northerly) winds, acidic river waters with very low aragonite saturation state values have been recorded (Salisbury et al. 2008). Overall acidification is expected to increase, although estuaries also naturally experience daily fluxes in pH and some species acclimated to variability may be resilient to ocean acidification. Some studies have shown additive and synergistic negative effects on bivalves and gastropods from ocean acidification and low dissolved oxygen (Kroeker et al. 2013; Gobler et al. 2014; Clark and Gobler 2016; Gobler and Bauman 2016; Griffith and Gobler 2017).

Mollusks are also affected by increasing water temperatures, which are affecting their range (E. Powell and R. Mann, South Carolina Department of Natural Resources, personal communication). For the past two decades, ocean quahog recruitment and landings have shifted further north along the Atlantic coastline with substantial landings from southern New England waters (Lewis et al. 2001; MAFMC 2019). In addition, high nutrient levels and algal blooms can trigger reductions in dissolved oxygen in the nearshore water column and in sediments, which can have detrimental effects to infaunal and epifaunal species associated with sand habitats (Sharp et al. 1982; Brownlee et al. 2005).

Estuarine subtidal sand habitats may also experience some disturbance from storms, which are expected to increase in frequency and intensity in the Northeast region (USGCRP 2017), although generally less so than intertidal sand habitats.

Habitat Summary: Subtidal sand is generally the most resistant of all estuarine unconsolidated habitat types to disturbance, though the shallow water subtidal zone is more sensitive to disturbance in exposed locations than deeper, subtidal habitats. According to the 2012 EPA Coastal Condition Report, sediment quality in the Northeast Coast region (Chesapeake Bay and north) is rated fair, with 12% of the coastal area in poor condition and 11% in fair condition, largely driven by sediment contamination from heavy metals, polychlorinated biphenyls, and pesticides, with poorer quality sediments near urban areas (EPA 2012). Southern New England and Mid-Atlantic coastal areas have high contaminant loads due to legacy pollution from industrialization, and continued urbanization (EPA 2012). Coastal population density, development, and agriculture are all higher in the Mid-Atlantic and are associated with higher eutrophication. Benthic infauna and epifauna can be sensitive to contamination. However, coarse-grained sediments such as sand generally contain less total organic carbon (TOC) levels compared to soft-grained sediments. Because contaminants such as polyaromatic hydrocarbons, pesticides, and polychlorinated biphenyl are sequestered in the TOC fraction of sediments, sand sediments tend to be less impacted by contaminants than mud substrates (ICES 1992; Pierce 1994). Overall, further degradation of water and sediment quality is expected as urbanization and development in the coastal zone continues with increasing population growth.

The habitat is relatively resilient, as associated infauna and epifauna are generally easily dispersed within and between individual estuaries, and have relatively rapid recovery rates due to short life spans. However, recovery rates depend on the frequency of natural and anthropogenic disturbance. Sand bottom habitat has the ability to move under appropriate hydrodynamic conditions. However, the presence of artificial structures (jetties, groins) or natural rocky bottom can restrict its movement. Bottom-tending mobile fishing gear appear to have minimal physical or biological impacts to sand bottom habitats (Stevenson et al. 2004). Aquaculture operations may also cause some disturbance to benthic habitats through the accumulation of nutrients, wastes, or sediment deposition, but good water flow and husbandry can limit this accumulation (ASMFC 2020).

Sand sediments and associated infauna are sensitive to dredging impacts, although most species recovery relatively rapidly (Newell et al. 1998; Wilber et al. 2005). Shellfish generally have the capacity to rebound from poor growth conditions, if sufficient seed populations are present, and conditions for growth improve to favorable conditions. However, there is evidence that some of the changes occurring in the study area, such as increasing water temperatures, may prevent some species from rebounding (Lewis et al. 2001; MAFMC 2019), compounding the impacts of anthropogenic disturbance. Surf clam stocks in the Mid-Atlantic region have declined dramatically over the last 10 years, especially in New Jersey waters (MAFMC 2019). For the past two decades, ocean quahog landings have shifted further north along the Atlantic coastline with substantial landings from southern New England waters (MAFMC2019).

Shoreline hardening with riprap revetment converts sand bottom to large diameter, engineered stone in the upper subtidal zone. All hardened shorelines have the potential to erode shallow water subtidal sand bottom. Hardened shorelines have been shown to have lower abundance, biomass, and diversity of benthic prey and predators (Seitz et al. 2006; Morley et al. 2012). Other effects of engineered-shore structures include loss of sediment and reductions in beach volume and dimension (Kraus and McDougal 1996), loss of intertidal habitat and habitat fragmentation (Bozek and Burdick 2005; NRC 2007; Bulleri and Chapman 2010), and can have higher incidence of invasive species compared to native material (Tyrrell and Byers 2007; Geraldi et al. 2013; Pappal 2010).

Subtidal sand infauna and epifauna are also sensitive to invasive species, including green crab (*Carcinus maenas*) which is believed to have been carried by ships in ballast water and sold as fish bait in much of the world. It now has established populations in New England and northern Mid-Atlantic in both subtidal and intertidal zones. It is a predator of many forms of marine life, including worms and mollusks (GISD 2020). In some areas (particularly New England), the crab's voracious appetite has affected the commercial shellfish industry (Webber 2013; Beal 2014).

Literature Cited

Atlantic States Marine Fisheries Commission (ASMFC). 2020. Aquaculture: effects on fish habitat along the Atlantic Coast. Atlantic States Marine Fisheries Commission Habitat Management Series #16. http://www.asmfc.org/files/Habitat/HMS16_Aquaculture_May2020.pdf

Beal BF. 2014. Final report: Green crab, *Carcinus maenas*, trapping studies in the Harraseeket River and manipulative field trials to determine effects of green crabs on the fate and growth of wild and cultured individuals of soft-shell clams, *Mya arenaria* (May to November 2013). Downeast Institute for Applied Marine Research and Education, Beals, ME. 76 pp. (Accessed 30 Oct. 2020). https://downeastinstitute.org/wp-content/uploads/2018/08/1_24-final-report-freeport-shellfish-restoration-project-b.-beal.pdf.

Bozek CM, Burdick DM. 2005. Impacts of seawalls on saltmarsh plant communities in the Great Bay Estuary, New Hampshire USA. Wetlands Ecology and Management 13(5):553-68.

Brown AC, McLachlan A. 2002. Sandy shore ecosystems and the threats facing them: some predictions for the year 2025. Environmental Conservation 29(1):62-77.

Brownlee EF, Sellner SG, Sellner K.G. 2005. *Prorocentrum minimum* blooms: potential impacts on dissolved oxygen and Chesapeake Bay oyster settlement and growth. Harmful Algae 4(3):593-602. https://doi.org/10.1016/j.hal.2004.08.009.

Bulleri F, Chapman MG. 2010. The introduction of coastal infrastructure as a driver of change in marine environments. Journal of Applied Ecology 47(1):26-35.

Clark H, Gobler CJ. 2016. Diurnal fluctuations in CO2 and dissolved oxygen concentrations do not provide a

refuge from hypoxia and acidification for early-life-stage bivalves. Marine Ecology Progress Series 558:43114. https://doi.org/10.3354/meps11852

Ekstrom JA, Suatoni L, Cooley SR, Pendleton LH, Waldbusser GG, Cinner JE, Ritter J, Langdon C, van Hooidonk R, Gledhill D, Wellman K, Beck MW, Brander LM, Rittschof D, Doherty C, Edwards PET, Portela R. 2015. Vulnerability and adaptation of US shellfisheries to ocean acidification. Nature Climate Change 5(3):207-14.

Gazeau F, Parker LM, Comeau S, Gattuso J-P, O'Connor WA, Martin S, Pörtner H-O, Ross PM. 2013. Impacts of ocean acidification on marine shelled molluscs. Marine Biology 160(8):2207-45.

Geraldi NR, Smyth AR, Piehler MF, Peterson CH. 2013. Artificial substrates enhance non-native macroalga and N_2 production. Biological Invasions 16(9):1819-31.

Gledhill D, White M, Salisbury J, Thomas H, Misna I, Liebman M, Mook B, Grear J, Candelmo A, Chambers RC, Gobler C, Hunt C, King A, Price N, Signorini S, Stancioff E, Stymiest C, Wahle R, Waller J, Rebuck N,Wang Z, Capson T, Morrison JR, Cooley S, Doney S. 2015. Ocean and coastal acidification off New England and Nova Scotia. Oceanography 25(2):182-97.

Global Invasive Species Database (GISD). 2020. [Internet]. Species profile: *Carcinus maenas*. (Accessed 3 August 2020). http://www.iucngisd.org/gisd/species.php?sc=114.

Gobler CJ, Baumann H. 2016. Hypoxia and acidification in ocean ecosystems: coupled dynamics and effects on marine life. Biology Letters 12(5):20150976.

Gobler CJ, DePasquale EL, Griffith AW, Baumann H. 2014. Hypoxia and acidification have additive and synergistic negative effects on the growth, survival, and metamorphosis of early life stage bivalves. PLoS One 9(1):e83648.

Griffith AW, Gobler CJ. 2017. Transgenerational exposure of North Atlantic bivalves to ocean acidification renders offspring more vulnerable to low pH and additional stressors. Scientific Reports 7:11394.

Gutierrez BT, Williams SJ, Thieler ER. 2007. Potential for shoreline changes due to sea-level rise along the U.S. Mid-Atlantic region. U.S. Geological Survey. Open-File Report 2007-1278. 1-25.

International Council for the Exploration of the Sea (ICES). 1992. Report of the ICES working group on the effects of extraction of marine sediments on fisheries. Copenhagen (Denmark): ICES Cooperative Research Report #182.

Kraus NC, McDougal WG. 1996. The effects of seawalls on the beach: Part 1, an updated literature review. Journal of Coastal Research 12(3):691-701.

Kroeker KJ, Kordas RL, Crim R, Hendriks IE, Ramajo L, Singh GS, Duarte CM, Gattuso J-P. 2013. Impacts of ocean acidification on marine organisms: quantifying sensitivities and interaction with warming. Global Change Biology 19(6):1884-96.

Lewis CVW, Weinberg JR, Davis CS. 2001. Population structure and recruitment of the bivalve *Arctica islandica* (Linnaeus, 1767) on Georges Bank from 1980-1999. Journal of Shellfish Research 20:1135-44.

Mid-Atlantic Fishery Management Council (MAFMC). 2019. [Internet]. Ocean quahog and surf clam fishery information document. April 1, 2019. (Accessed 8 December 2020). http://www.mafmc.org/surfclams-quahogs.

Morley SA, Toft JD, Hanson KM. 2012. Ecological effects of shoreline armoring on intertidal habitats of a

Puget Sound urban estuary. Estuaries and Coasts 35(3):774-84.

National Research Council (NRC). 2007. Mitigating eroding sheltered shorelines: a trade-off in ecosystem services. Mitigating shore erosion along sheltered coasts. p. 78-97. Washington, DC: National Research Council.

Newell RC, Seiderer LJ, Hitchcock DR. 1998. The impact of dredging works in coastal waters: a review of the sensitivity to disturbance and subsequent recovery of biological resources on the sea bed. In: Ansell AD, Gibson RN, Barnes M, editors. Oceanography and Marine Biology: An Annual Review 36. Oban, Argyll (Scotland): UCL Press. 127-78.

Pappal A. 2010. Marine invasive species. State of the Gulf of Maine Report. [Currier P, Gould D, Hertz L, Huston J, Tremblay ML (eds)]. The Gulf of Maine Council on the Marine Environment. 1-21.

Pearce JB. 1994. Mining of seabed aggregates. *In*: Langton RW, Pearce JB, Gibson JA, editors. Selected living resources, habitat conditions, and human perturbations of the Gulf of Maine: environmental and ecological considerations for fishery management. Woods Hole, MA. NOAA Technical Memorandum. NMFS-NE-106. 48-50.

Salisbury J, Green M, Hunt C, Campbell J. 2008. Coastal acidification by rivers: a threat to shellfish? Eos, Transactions American Geophysical Union 89(50):513-28.

Seitz RD, Lipcius RN, Olmstead NH, Seebo MS, Lambert DM. 2006. Influence of shallow-water habitats and shoreline development on abundance, biomass, and diversity of benthic prey and predators in Chesapeake Bay. Marine Ecology Progress Series 326:11-27.

Sharp JH, Culberson CH, Church TM. 1982. The chemistry of the Delaware estuary. General considerations. Limnology and Oceanography 27. doi:10.4319/lo.1982.27.6.1015.

Stevenson D, Chiarella L, Stephan D, Reid R, Wilhelm K, McCarthy J, Pentony M. 2004. Characterization of the fishing practices and marine benthic ecosystems of the Northeast U.S. Shelf, and an evaluation of the potential effects of fishing on Essential Fish Habitat. NOAA Technical Memorandum NMFS-NE-181. 179 p.

Tyrrell MC, Byers JE. 2007. Do artificial substrates favor nonindigenous fouling species over native species? Journal of Experimental Marine Biology and Ecology 342(1):54-60.

U.S. Environmental Protection Agency (EPA). 2012. National Coastal Condition Report IV. EPA-R-842-10-003. US Environmental Protection Agency, Washington D.C.

U.S. Global Change Research Program (USGCRP). 2017. Climate Science Special Report: Fourth National Climate Assessment. Volume I [Wuebbles, D.J., D.W. Fahey, K.A. Hibbard, D.J. Dokken, B.C. Stewart, and T.K. Maycock (eds.)]. U.S. Global Change Research Program. Washington, DC. USA. 470 pp. doi:10.7930/J0J964J6.

Waldbusser GG, Voigt EP, Bergschneider H, Green MA, Newell RIE. 2011. Biocalcification in the eastern oyster (*Crassostrea virginica*) in relation to long-term trends in Chesapeake Bay pH. Estuaries and Coasts 34(2):221-31.

Webber MM. 2013. [Internet]. Results of the one-day green crab trapping survey conducted along the Maine coast from August 27 to 28, 2013. Maine Department of Marine Resources, Augusta, ME. 6 pp. (Accessed 30 Oct. 2020). https://www.maine.gov/dmr/science-research/species/invasives/greencrabs/documents/trapsurvey rpt2013.pdf.

Wilber DH, Brostoff W, Clarke DG, Ray GL. 2005. Sedimentation: Potential biological effects from dredging operations in estuarine and marine environments. DOER Technical Notes Collection (ERDC TN-DOER-E20). U.S. Army Engineer Research and Development Center, Vicksburg, MS.

Estuarine Water Column

System: Estuarine

Subsystem: Subtidal

Class: Water Column

Sub-class: Well-mixed

Geographic Area: Entire Area

Overall Vulnerability Rank = High

Habitat Sensitivity = High

Climate Exposure = High

	Estuarine Water Column	Attribute Mean	Data Quality	Distribution of Expert Scores
Sensitivity Attributes	Habitat condition	2.8	2.5	
	Habitat fragmentation	1.9	2	
	Distribution/Range	1.4	2	
	Mobility/Ability to spread or disperse	1.8	1.8	
	Resistance	2.2	2	
	Resilience	1.7	2.1	
	Sensitivity to changes in abiotic factors	3.4	2.2	
	Sensitivity and intensity of non−climate stressors	3.4	2.2	
	Dependency on critical ecological linkages	2.5	2	
	Sensitivity Component Score	High		
Exposure Factors	Sea surface temp	3.7	2.5	
	Bottom temp	n/a	n/a	
	Air temp	n/a	n/a	
	River temp	n/a	n/a	
	Surface salinity	2.6	2.1	
	Bottom salinity	n/a	n/a	
	pH	4	2	
	Sea level rise	2.2	2.2	
	Precipitation	2.5	2.1	
	Floods	n/a	n/a	
	Droughts	n/a	n/a	
	Exposure Component Score	High		
**Overall Vulnerability Rank**		**High**		

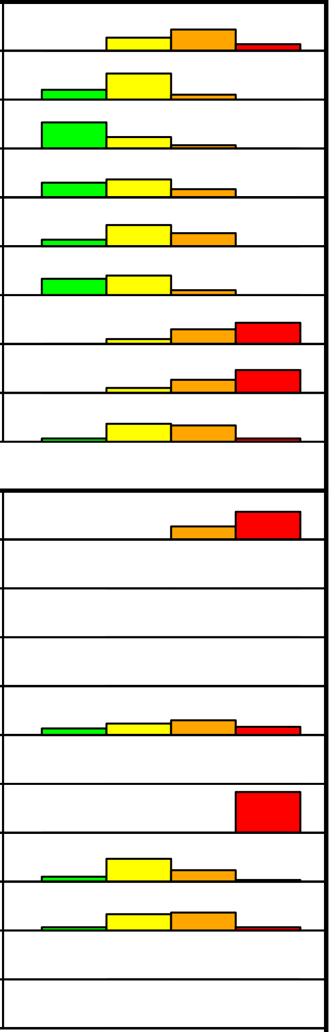

Low
Moderate
High
Very High

Habitat Name: Estuarine Water Column

System**:** Estuarine

Subsystem: Subtidal

Class: Water Column

Sub-class: Well-mixed

Geographic Area: Entire Area

Habitat Description: This sub-class includes the estuarine water column from the surface (mean low water) to the bottom of estuaries. This includes all estuaries types based on circulation (salt-wedge, well-mixed, partially-mixed, and fjord) (Dyer 1973).

The water column is a concept used in oceanography to describe the physical (e.g., temperature, salinity, light penetration) and chemical (e.g., pH, dissolved oxygen, nutrients, salts) characteristics of seawater at different depths. Water column habitats create the foundation for estuarine food webs, home to primary producers such as phytoplankton and microbes. These habitats are highly dynamic andexhibit swift responses to environmental variables.

Overall Climate Vulnerability Rank: High (100% certainty from bootstrap analysis).

Climate Exposure: High. The overall high exposure score was influenced by two very high attribute means: Sea Surface Temperature and pH (3.7 and 4.0, respectively). The exposure attribute scores for Surface Salinity, Precipitation, and Sea Level Rise (SLR) were 2.6, 2.5, and 2.2, respectively. The scores for Surface Salinity were spread over all four scoring bins, which likely reflects the large range of projected changes in estuarine salinities over the study area, as well as the variability of physical characteristics of estuaries in the study area. pH is projected to drastically decrease from historic levels for the entire study area by the end-of-century (increasing ocean acidification).

Habitat Sensitivity: High. The overall high sensitivity score was influenced by two sensitivity attributes: Sensitivity to Changes in Abiotic Factors and Sensitivity and Intensity of Non−Climate Stressors- mean of 3.4 for both. The means for the other seven sensitivity attributes ranged from 2.8 to 1.4.

Data Quality & Gaps: The data quality scores for all five climate exposure factors ranged between 2.0 and 2.5. The low data quality score for pH (2.0) was likely attributed to the low resolution of CMIP5 projections for nearshore, shallow areas. Surface Salinity data quality scored Low (2.1), which likely reflects the large range of projected change in estuarine salinities over the study area. SLR and Precipitation scored low (2.2 and 2.1, respectively), which may be attributed to the perceived uncertainty to changes in sea level and precipitation in the vertical distribution of the water column. Data quality is likely lower for estuarine habitats in general due to the low resolution of CMIP5 andROMS-NWA projections for climate exposure in estuaries and nearshore, shallow coastal areas.

For habitat sensitivity, data quality for all nine attributes ranged between 1.8 and 2.5. These moderate data quality scores may reflect the large variability of physical characteristics of estuaries over the study area. For example, some estuaries are influenced by freshwater flow from rivers, streams, and urban runoff, while others are principally affected by marine waters and this may result in differences in climate sensitivity.

Positive or Negative Climate Effect for the Northeast U.S.: The effect of climate change on estuarine water column the Northeast U.S. is expected to be neutral (60% of the experts' scores), but 40% of the experts expected negative effects. This is likely a reflection of the range of estuarine conditions throughout the study area, although may also suggest uncertainty in projected climate effects on the estuarine water column.

Climate Effects on Habitat Condition and Distribution: Water is a universal component of marine ecosystems and is the habitat or a dominant component of the habitat for all marine organisms. Characteristics of water column habitat include temperature, salinity, dissolved oxygen, carbonate chemistry,

nutrients, and primary and secondary producers. Characteristics also include currents and stratification/mixing.

The estuarine water column is very dynamic and impacted by air-sea exchange, inputs from terrestrial environments through freshwater inflows and by marine environments through mixing with salt water. Currents are largely driven by the inflow from rivers and tides and to a lesser extent by wind. Many estuaries in the northeast are "partially-mixed estuaries" (Dyer 1973), where tides partially break down the stratification between fresh, less dense water at the surface and salty, more dense water at depth. These basic physical dynamics will not be interrupted by climate changes, but changes in freshwater inflows and changes in sea level could modify both salinity gradients in estuaries and the geomorphology of estuaries.

Water temperature in estuaries is largely influenced by heat exchange with the atmosphere and freshwater input, the temperature of which is also influenced by heat exchange with the atmosphere (Hare et al. 2010). The temperature of the region's estuaries have warmed over the past several decades (Bell et al. 2014).

The salinity range in estuaries is by definition large, from fresh (0 ppt.) to almost marine (26 to 30 ppt.). Owing to increased precipitation, freshwater inflows have increased in some of the region's estuaries (Smith et al. 2010). The effects of increased inflows will likely change the spatial structure of the salinity gradient; in a modeling study, Gibson and Najjar (2000) found that increased inflow into Chesapeake affected the middle of the bay to a much greater degree than the upper reachesand the mouth of the bay. However, there is a lot of seasonal variability in streamflows and climate change effects on streamflow and river inflow have a lot of variability as well.

Stratification in estuaries is unlikely to change much because of wind and tidal mixing. However, stratification could increase as a result of increased freshwater inflows and increased air temperatures (Najjar et al. 2010). Changes in stratification could have consequences for oxygen-levels; hypoxia does occur in estuarine systems throughout the Northeast largely as a result of summertime thermal stratification and increased primary production (Nixon et al. 2009).

Carbonate chemistry in estuaries is complicated owing to freshwater input, which supplies dissolved inorganic and dissolved and particulate organic carbon; primary production, which uses carbon dioxide; respiration, which produces carbon dioxide; and increases in atmospheric carbon dioxide, which dissolves into seawater causing ocean acidification. These factors and associated changes in carbonate chemistry are termed coastal acidification (Gledhill et al. 2015) and as a result of these various factors, carbonate chemistry variability in estuarine waters exhibits a higher frequency of variability compared to shelf and oceanic waters (Waldbusser and Salisbury 2014). Carbonate chemistry in coastal waters is further complicated by eutrophication, which is an increase in nutrient supply from freshwater input that results in increased primary production. Subsequent respiration can drive up local CO_2 concentrations and drive down local O_2 concentrations. Thus, eutrophication is linked to hypoxia and anoxia in many estuarine systems in the regions (Wallace et al. 2014; Cai et al. 2017). The long-term increase in atmospheric CO_2 will lead to increases in dissolved inorganic carbon and decreases in estuarine pH, but the magnitude of the shorter term variability associated with freshwater input, eutrophication, primary production, and respiration will dominate carbon carbonate chemistry (Cai et al.2017).

Habitat Summary: Estuarine water column habitat has a high climate exposure owing to projected changes in sea surface temperature and pH. Sea surface temperature increases are projected throughout the region, particularly in estuaries (Muhling et al. 2018). Ocean acidification projections also indicate large changes in pH in the future. In estuarine systems, acidification is affected by multiple factors only one of which is increases in atmospheric CO_2. Thus, the projections used do not represent the full suite of factors determining pH (Gledhill et al. 2015).

Estuarine water column habitat also has a high sensitivity owing to high sensitivity to abiotic stressors and high sensitivity to non-climatic stressors. Temperature, salinity, dissolved oxygen, nutrients, carbonate chemistry in estuarine water columns are all highly variabile, indicating relative quick response to abiotic

stressors (Najjar et al. 2010). Estuaries in the Northeast U.S. are also highly impacted by non-climate stressors. Pollutants can remain in the water column, be flushed from the system, or be deposited in sediments (Achman et al 1996; Santschi et al. 2017). Harmful algal blooms can introduce toxins into the water column (Anderson 2002). Eutrophication remains an issue in many Northeast estuaries (Seitzinger and Sanders 1997). The Environmental Protection Agency's Coastal Condition Report rates the water quality for estuarine waters of the Northeast U.S. ecosystem as fair; the water quality index was based on measurements of five component indicators: dissolved inorganic nitrogen, dissolved inorganic phosphorus, chlorophyll a, water clarity, and dissolved oxygen (EPA 2012).

Literature Cited

Achman DR, Brownawell BJ, Zhang L. 1996. Exchange of polychlorinated biphenyls between sediment and water in the Hudson River Estuary. Estuaries 19(4):950-65.

Anderson DM, Glibert PM, Burkholder JM. 2002. Harmful algal blooms and eutrophication:nutrient sources, composition, and consequences. Estuaries 25(4):704-26.

Bell RJ, Hare JA, Manderson JP, Richardson DE. 2014. Externally driven changes in the abundance of summer and winter flounder. ICES Journal of Marine Science 71(9):2416-428.

Cai WJ, Huang WJ, Luther GW, Pierrot D, Li M, Testa J, ... Xu YY. 2017. Redox reactions and weak buffering capacity lead to acidification in the Chesapeake Bay. Nature Communications8(1):1-12.

Dyer KR. 1973. Estuaries: a physical introduction. Wiley-Interscience, New York and London. xv + I40 p. Limnology and Oceanography 18. doi:10.4319/lo.1973.18.6.1012.

Gibson JR, Najjar RG. 2000. The response of Chesapeake Bay salinity to climate-induced changes in streamflow. Limnology and Oceanography 45(8):1764-72.

Gledhill DK, White MM, Salisbury J, Thomas H, Mlsna I, Liebman M, ... Gobler CJ. 2015. Ocean and coastal acidification off New England and Nova Scotia. Oceanography 28(2):182-97.

Hare JA, Alexander MA, Fogarty MJ, Williams EH, Scott JD. 2010. Forecasting the dynamics of a coastal fishery species using a coupled climate–population model. Ecological Applications 20(2):452-64.

Muhling BA, Gaitán CF, Stock CA, Saba VS, Tommasi D, Dixon KW. 2018. Potential salinity and temperature futures for the Chesapeake Bay using a statistical downscaling spatial disaggregation framework. Estuaries and coasts 41(2):349-72.

Najjar RG, Pyke CR, Adams MB, Breitburg D, Hershner C, Kemp M, Howarth R, Mulholland MR, Paolisso M, Secor D, Sellner K, Wardrop D, Wood R. 2010. Potential climate-change impacts on the Chesapeake Bay. Estuarine, Coastal and Shelf Science 86(1):1-20.

Nixon SW, Fulweiler RW, Buckley BA, Granger SL, Nowicki BL, Henry KM. 2009. The impact of changing climate on phenology, productivity, and benthic–pelagic coupling in Narragansett Bay. Estuarine, Coastal and Shelf Science 82(1):1-18.

Santschi PH, Yeager KM, Schwehr KA, Schindler KJ. 2017. Estimates of recovery of the Penobscot River and estuarine system from mercury contamination in the 1960's. Science of theTotal Environment 596:351-9.

Seitzinger SP, Sanders R. W. 1997. Contribution of dissolved organic nitrogen from rivers to estuarine eutrophication. Marine Ecology Progress Series 159:1-12.

Smith LM, Whitehouse S, Oviatt CA. 2010. Impacts of climate change on Narragansett Bay. Northeastern Naturalist 17(1):77-90.

U.S. Environmental Protection Agency (EPA). 2012. National Coastal Condition Report IV.EPA-R-842-10-003. US Environmental Protection Agency, Washington D.C. https://www.epa.gov/sites/production/files/2014-10/documents/0_nccr_4_report_508_bookmarks.pdf

Waldbusser GG, Salisbury JE. 2014. Ocean acidification in the coastal zone from an organism's perspective: multiple system parameters, frequency domains, and habitats. Annual Review of Marine Science 6:221-47.

Wallace RB, Baumann H, Grear JS, Aller RC, Gobler CJ. 2014. Coastal ocean acidification: The other eutrophication problem. Estuarine, Coastal and Shelf Science 148:1-13.

Riverine Non−tidal Invasive Wetland

System: Riverine

Subsystem: Non-tidal

Class: Emergent Wetland

Sub-class: Invasive Wetland

Geographic Area: Entire Area

Overall Vulnerability Rank = Low

Habitat Sensitivity = Low

Climate Exposure = High

	Riverine Non−tidal Invasive Wetland	Attribute Mean	Data Quality	Distribution of Expert Scores
Sensitivity Attributes	Habitat condition	1.7	2.5	
	Habitat fragmentation	1.6	2	
	Distribution/Range	1.6	2	
	Mobility/Ability to spread or disperse	1.8	2.3	
	Resistance	1.6	1.5	
	Resilience	1.5	1.8	
	Sensitivity to changes in abiotic factors	1.8	2	
	Sensitivity and intensity of non−climate stressors	1.7	2	
	Dependency on critical ecological linkages	1.3	1.8	
	Sensitivity Component Score	Low		
Exposure Factors	Sea surface temp	n/a	n/a	
	Bottom temp	n/a	n/a	
	Air temp	4	2.8	
	River temp	n/a	n/a	
	Surface salinity	n/a	n/a	
	Bottom salinity	n/a	n/a	
	pH	n/a	n/a	
	Sea level rise	n/a	n/a	
	Precipitation	n/a	n/a	
	Floods	3	1.9	
	Droughts	3	1.9	
	Exposure Component Score	High		
**Overall Vulnerability Rank**		**Low**		

Low

Moderate

High

Very High

Habitat Name: Riverine Non-Tidal Invasive Wetland
System: Riverine
Subsystem: Non-Tidal
Class: Emergent Wetland
Sub-class: Invasive Wetland
Geographic Area: Entire Area

Habitat Description: This sub-class includes invasive riverine non-tidal freshwater wetlands dominated by perennial plants, characterized by erect, rooted, herbaceous hydrophytes, where salinity is effectively zero. The sub-class includes persistent and non-persistent wetlands above the influence of the tide.

Invasive tidal species include common reed (*Phragmites australis*) and purple loosestrife (*Lythrum salicaria*). The common reed is considered one of the most invasive plants in the marshes of northeastern North America (Tougas-Tellier et al. 2015). Purple loosestrife requires moist organic soils, but can tolerate fluctuating water levels and salinities, and can survive in many conditions associated with disturbed sites (UMaine 2001; NHDES 2019; USGS 2019). Invasive non-tidal freshwater wetlands are found throughout the study region, associated with non-tidal portions of rivers.

Overall Climate Vulnerability Rank: Low (100% certainty from bootstrap analysis).

Climate Exposure: High. The highest climate exposure score was for Air Temperature (4.0), followed by Floods (3.0), and Droughts (3.0). Air temperature is projected to increase significantly across the range. Floods and droughts are projected to increase in magnitude and frequency in the Mid-Atlantic. In New England, foods are expected to increase in frequency but decrease in magnitude, and droughts are expected to increase in magnitude but decrease in frequency.

Habitat Sensitivity: Low. All sensitivity attributes scored in the Low range, with none >2.0. Invasive wetlands are generally well adapted to anthropogenic and climate disturbances.

Data Quality & Gaps: The highest data quality score for climate exposure was Air Temperature (2.8), while Floods and Drought were both moderately low (1.9). Because of the fine-scale nature of riverine habitats and the coarse scale of this assessment, text descriptions of the spatial distribution of invasive non-tidal freshwater wetlands were used for the climate exposure scoring instead of maps of habitat distribution, which limited the ability of scorers to ascertain the overlap between the distribution of the habitat and climate exposure projections.

Seven of the nine sensitivity attribute data quality scores were ≤2.0: Resistance (1.5), Resilience (1.8), Dependency on Critical Ecological Linkages (1.8), Distribution/Range (2.0), Habitat Fragmentation (2.0), Sensitivity and Intensity of Non-Climate Stressors (2.0), and Sensitivity to Changes in Abiotic Factors (2.0).

Positive or Negative Climate Effect in the Northeast U.S.: The effect of climate change in riverine non-tidal invasive wetland in the Northeast U.S. is generally expected to be positive (85% of the experts' scores were positive, 15% were negative). Invasive non-tidal freshwater wetlands may benefit from climate change through competitive advantage over native species.

Climate Effects on Habitat Condition and Distribution: Common reed and purple loosestrife are highly adapted to disturbance, and will likely benefit from climate change by outcompeting native species. In general, climate change is expected to benefit invasive species and exacerbate their impacts on ecosystems (Dukes and Mooney 1999).

Floods and droughts are projected to change (increase) significantly in the Northeast U.S. (Demaria et al. 2015). Increased drought can increase the spread of invasive species like the common reed, and make native wetlands more vulnerable (Tougas-Tellier et al. 2015). As water levels drop, prolonged exposure of bare soil allows the seeds of common reed to germinate and spread through vegetative propagation.

Common reed also alters sediment dynamics through the build-up of vegetation, reducing water flow and changing topography, and contributing to drying (Tougas-Tellier et al. 2015). These conditions present synergistic challenges for native species, as the increases in invasives like the common reed alter sediment dynamics building up vegetation, thus reducing water flow and impacting topography, which contributes to drying. However, increased incidences of extreme precipitation and flooding may make it more difficult for common reed to colonize and restrict plants from migrating landward or upstream. Increased riverine flow may also result in scour of the wetland edge.

In general, invasive non-tidal riverine wetlands are tolerant of climate-related stressors. Sea level rise (SLR) is not expected to affect most non-tidal freshwater wetlands. However, common reed has a broad salinity tolerance (Meyerson et al. 2000), and in non-tidal areas that may experience increased salinity caused by a changing salt wedge due to SLR or changing groundwater levels, its distribution may expand in non-tidal riverine areas.

Increases in temperature can lead to an increase in photosynthetic rates and plant biomass, as can increases in CO_2 (Kirwan and Blum 2011). Higher temperatures can increase the production of soil organic matter, but also increase the rates of decomposition (Kirwan and Blum 2011). However, the common reed stands accumulate detritus and have slower stem decomposition rates (Meyerson et al. 2000), so subsidence is less of a concern than it is in native marshes.

Habitat Summary: Invasive freshwater wetlands are dominated by a few species, but those species are highly adaptable and tolerant of suboptimal conditions (Meyerson et al. 2000). Invasive species typically tolerate disturbed areas better than natives, and will colonize and outcompete natives (Mitsch and Gosselink 2000; UMaine 2001; Middleton 2006; Dahl and Stehman 2013; NHDES 2019). Common reed is able to establish in areas with significant human disturbance, including development, pollution, and mechanical disturbance (Meyerson et al. 2000). Similarly, purple loosestrife can survive in many conditions associated with disturbed sites, and can grow in brackish and non-tidal waters, allowing it to outcompete native vegetation and to form dense stands (UMaine 2001; Middleton 2006; NH DES 2019; USGS 2019). While coastal and riverine development has led to overall fragmentation for riverine wetlands, invasive freshwater wetlands are not functionally harmed by fragmentation. However, shoreline hardening and other anthropogenic barriers may limit their ability to spread.

Literature Cited

Dahl TE, Stedman S-M. 2013. Status and trends of wetlands in the coastal watersheds of the conterminous United States 2004 to 2009. U.S. Department of the Interior, Fish and Wildlife Service and National Oceanic and Atmospheric Administration, National Marine Fisheries Service. 1-46.

Demaria EMC, Palmer RN, Round JK. 2015. Regional climate change projections of streamflow characteristics in the Northeast and Midwest U.S. Journal of Hydrology: Regional Studies 5:309-23.

Dukes JS, Mooney HA. 1999. Does global change increase the success of biological invaders? Trends in Ecology and Evolution 14:135-9.

Kirwan ML, Blum LK. 2011. Enhanced decomposition offsets enhanced productivity and soil carbon accumulation in coastal wetlands responding to climate change. Biogeosciences 8(4):987-93.

Meyerson LA, Saltonstall K, Windham L, Kiviat E, Findlay S. 2000. A comparison of *Phragmites australis* in freshwater and brackish marsh environments in North America. Wetlands Ecology and Management 8:89-103.

Middleton BA. 2006. Invasive species and climate change: U.S. Geological Survey Open-File Report 2006-1153:2. https://pubs.usgs.gov/of/2006/1153/pdf/of06-1153_508.pdf

Mitsch WJ, Gosselink JG. 2000. Wetlands, 3rd edition. John Wiley and Sons.

New Hampshire Department of Environmental Services (NHDES). 2019. Purple loosestrife. Environmental fact sheet WD-BB-45.

Tougas-Tellier MA, Morin J, Hatin D, Lavoie C. 2015. Freshwater wetlands: fertile grounds for the invasive *Phragmites australis* in a climate change context. Ecology and Evolution 5(16):3421-35.

University of Maine (UMaine). 2001. Purple loosestrife. Maine invasive plants. Bulletin #2508.

United States Geological Survey (USGS). 2019. [Internet]. Non-indiginous aquatic species: purple loosestrife. (accessed 23 November 2020). https://nas.er.usgs.gov/queries/FactSheet.aspx?SpeciesID=239.

Riverine Non−tidal Native Wetland

System: Riverine

Subsystem: Non-tidal

Class: Emergent Wetland

Sub-class: Native Wetland

Geographic Area: Entire Area

Overall Vulnerability Rank = High

Habitat Sensitivity = High

Climate Exposure = High

	Riverine Non−tidal Native Wetland	Attribute Mean	Data Quality	Distribution of Expert Scores
Sensitivity Attributes	Habitat condition	3	2.5	
	Habitat fragmentation	3	2	
	Distribution/Range	1.6	2	
	Mobility/Ability to spread or disperse	3.2	2.3	
	Resistance	2.7	1.8	
	Resilience	2.3	1.8	
	Sensitivity to changes in abiotic factors	3.1	2.5	
	Sensitivity and intensity of non−climate stressors	3.7	1.8	
	Dependency on critical ecological linkages	1.5	1.8	
	Sensitivity Component Score	High		
Exposure Factors	Sea surface temp	n/a	n/a	
	Bottom temp	n/a	n/a	
	Air temp	4	2.8	
	River temp	n/a	n/a	
	Surface salinity	n/a	n/a	
	Bottom salinity	n/a	n/a	
	pH	n/a	n/a	
	Sea level rise	n/a	n/a	
	Precipitation	n/a	n/a	
	Floods	3	1.9	
	Droughts	3	1.9	
	Exposure Component Score	High		
**Overall Vulnerability Rank**		**High**		

Low
Moderate
High
Very High

Habitat Name: Riverine Non-Tidal Native Wetland

System: Riverine

Subsystem: Non-Tidal

Class: Emergent Wetland

Sub-class: Native Wetland

Geographic Area: Entire Area

Habitat Description: This sub-class includes native riverine non-tidal freshwater wetlands associated with stream and river channels, and are usually located in the floodplain of such watercourses. Riverine wetlands located in headwaters, or the upper reaches of a watershed, are very important to water quality. Native non-tidal wetlands are dominated by native hydrophytic vegetation, hydric soils, and hydrology as defined by sources and hydroperiod, and where the salinity is effectively zero. While tidal freshwater wetlands occur in brackish or alkaline waters in salinities less than or equal to 0.5 ppt, non-tidal wetlands occur in waters that are effectively freshwater. For the purposes of this study, the non-tidal riverine system terminates where the channel enters a lake.

This sub-class includes persistent and non-persistent wetlands above the influence of the tide. Native non-tidal wetland plant species include herbaceous plants, grasses, sedges and rushes which have an indicator status that reflects a likelihood that that species occurs in wetlands. Riverine non-tidal wetlands within the geographic area could include common native species such as swamp milkweed (*Asclepias incarnata*), swamp aster (*Aster puniceus*), marsh marigold (*Caltha palustris*), broad-leaved cattail (*Typha latifolia),* sedges (*Carex spps*.), rushes (*Juncus* spp.) and grasses.

Native non-tidal wetlands are found throughout the study region, limited to the freshwater portions ofrivers. Non-tidal marshes are the most prevalent and widely distributed wetlands in coastal watersheds of the U.S. (Dahl and Stedman 2013). They frequently occur in poorly drained depressions and in the shallow water along the boundaries of streams and rivers. Water levels in these wetlands generally vary from a few inches to two or three feet and some marshes may periodically dry out completely. Freshwater wetlands have characteristic patterns of zonation for flora and fauna tied to frequency and depth of inundation, and have a diverse community composition.

Overall Climate Vulnerability Rank: High (87% certainty from bootstrap analysis). Bootstrap analysis found a 13% probability that the overall vulnerability rank is Moderate.

Climate Exposure: High. The highest climate exposure score was for Air Temperature (4.0) followed by Floods (3.0) and Droughts (3.0). Air temperature is projected to increase significantly across the range. Floods and droughts are projected to increase in magnitude and frequency in the Mid-Atlantic. In New England, floods are expected to increase in frequency but decrease in magnitude, and droughts are expected to increase in magnitude but decrease in frequency.

Habitat Sensitivity: High. Five sensitivity attributes contributed to the High sensitivity score with scores ≥3.0: Sensitivity and Intensity of Non-Climate Stressors (3.7), Mobility/Ability to Spread or Disperse (3.2), Sensitivity to Changes in Abiotic Factors (3.1), Habitat Condition (3.0), and Habitat Fragmentation (3.0). Freshwater non-tidal native wetlands are sensitive to changing groundwater levels, salinity, and to changes in the marsh platform that may result from increased floods and drought, and higher temperatures. The condition of the habitat has been degraded by several current and historic anthropogenic activities including development and nonpoint source pollution.

Data Quality & Gaps: The highest data quality score for climate exposure was Air Temperature (2.8), while Floods and Drought were both moderately low (1.9). Because of the fine-scale nature of riverine habitats and the coarse scale of this assessment, text descriptions of the spatial distribution of native non-tidal freshwater wetlands were used for the climate exposure scoring instead of maps of habitat distribution, which limited the ability of scorers to ascertain the overlap between the distribution of the habitat and climate exposure projections.

Six of the nine sensitivity attribute data quality scores were ≤2.0: Resistance (1.8), Resilience (1.8), Dependency on Critical Ecological Linkages (1.8), Sensitivity and Intensity of Non-Climate Stressors (1.8), Habitat Fragmentation (2.0), and Distribution/Range (2.0).

Positive or Negative Climate Effect in the Northeast U.S.: The effect of climate change in riverine non-tidal native wetland in the Northeast U.S. is expected to be negative (75% of the experts' scores were negative, 25% were neutral).

Climate Effects on Habitat Condition and Distribution: Dominant non-tidal wetland flora are eurythermal temperate species, but are sensitive to hydrology, topography (changes in elevation), and salinity. Any disturbance that affects these characteristics can therefore impact community structure,which dictates many wetland ecosystem functions.

Floods and droughts are projected to change (increase) significantly in the Northeast U.S. (Demaria et al. 2015). If increased riverine flow is temporary (e.g. after a storm event), hydrophytes should be tolerant of the higher water levels and could act as buffers to slow down the inundating water, which may cause sediment accretion on the wetland (Najjar et al. 2000). If the increased flow or water depth is long-term, then migration of plants landward or upstream may result. However, horizontal and upstream migration will be limited by steep slopes in the upper reaches of river systems and hardened shorelines (Najjar et al. 2000; Barendregt and Swarth 2013). Anthropogenic barriers are common in urban areas, and many urban areas are located on rivers. In New England, dams and culverts limit wetland migration and can cause a loss of non-tidal wetlands (Leck and Crain 2009). Increased riverine flow may also result in scourof the wetland edge. Increasing frequency and intensity of droughts may also contribute to increased salinity. An increase in salinity may also increase microbial mineralization of organic matter, leading to a decrease in the stability of the marsh platform (Weston et al. 2006).

Sea level rise is not expected to affect most non-tidal freshwater wetlands. However, non-tidal areas that may experience increased salinity caused by a changing salt wedge due to sea level rise or changing groundwater levels could allow salt tolerant wetlands to expand in non-tidal riverine areas. In addition, common reed, which has a broad salinity tolerance (Meyerson et al. 2000), could migrate upstream and encroach on non-tidal native wetlands which may not be able to migrate upriver due to changes in geomorphology due to anthropogenic alterations to the surrounding landscape and hydrology.

Increased temperatures may result in an earlier onset of growth of those hydrophytes in which the germination of propagules and plant growth is primarily controlled by temperature. This may occur at the cost of macrophytes that have dormancy mechanisms regulated by environmental cues other than temperature (e.g., photoperiod). In addition, it seems plausible that because of milder winters, some non-native, thermophilous aquatic plants will spread to the north. Furthermore, in culturally eutrophicated waters in which the sediment compartment is heavily loaded with organic matter and/or nutrients, a rise in temperature may accelerate nutrient turnover for several years, resulting in algal blooms and shifts in quality and quantity of macrophyte vegetation (Brock and van Vierssen 1992).

Changes in temperature, precipitation and evaporation may lead to larger seasonal fluctuations in the water table and a more frequent or more prolonged period of desiccation. Some hydrophytes can cope with these circumstances, while others withstand dedication only for short periods. Macrophyte communities may also be affected in an indirect way by periodic desiccation of the habitats.

As temperatures increase, the distribution of non-tidal wetlands may shift northward (Barendregt and Swarth 2013). Increases in temperature can also lead to an increase in photosynthetic rates and plant biomass, as can increases in CO_2 (Kirwan and Blum 2011), and changes in wetland community composition. Increased temperatures increase the production of soil organic matter, but increased rates of decomposition as a result of warmer temperatures will likely outweigh the soil accumulation, causing wetland loss (Kirwan and Blum 2011).

Habitat Summary: Non-tidal freshwater wetlands condition is driven by historic practices like log drives and land conversion to agriculture, as well as current anthropogenic activities. Non-tidal freshwater wetlands are often located near urbanized areas, and most freshwater wetlands in the region experience chronic and long-term habitat degradation (Barendregt and Swarth 2013). According to the 2008-2009 National Wetland Condition Assessment report, compacted soil, ditching, and loss of vegetation are the most important anthropogenic impacts in U.S. freshwater wetlands (USEPA 2016). Other anthropogenic impacts include development, dredging, filling, sediment contamination, and invasives (e.g., purple loosestrife). Human development has led to a high degree of habitat fragmentation, especially in urbanized regions (Dahl and Stedman 2013). Invasives typically tolerate disturbed areas better than natives, and will colonize and outcompete natives (Mitsch and Gosselink 2000; Dahl and Stehman 2013).Increased eutrophication from nutrient runoff can also lead to increased decomposition rates in marsh plants, leading to an increase in porosity in the marsh platform and an elevated risk of edge erosion from floods and sea level rise.

Non-tidal freshwater wetlands can recover from natural disturbance under optimal conditions, especially with restoration efforts. However, recovery depends on level of disturbance (e.g. clear cutting but leaving roots allows faster recovery), hydrology, and other factors (Mitsch and Gosselink 2000). Competition with invasives is also an issue and can limit recovery of native species after a disturbance.

Literature Cited

Barendregt A, Swarth CW. 2013. Tidal freshwater wetlands: variation and changes. Estuaries and Coasts 36:445-56.

Brock TCM, van Vierssen W. 1992. Climate change and hydrophyte-dominated communities in inland wetland ecosystems. Wetlands Ecology and Management 2(1-2):37-49.

Dahl TE, Stedman S-M. 2013. Status and trends of wetlands in the coastal watersheds of the conterminous United States 2004 to 2009. U.S. Department of the Interior, Fish and Wildlife Service and National Oceanic and Atmospheric Administration, National Marine Fisheries Service. 1-46.

Demaria EMC, Palmer RN, Round JK. 2015. Regional climate change projections of streamflow characteristics in the Northeast and Midwest U.S. Journal of Hydrology: Regional Studies 5:309-23.

Kirwan ML, Blum LK. 2011. Enhanced decomposition offsets enhanced productivity and soil carbon accumulation in coastal wetlands responding to climate change. Biogeosciences 8(4):987-93.

Leck MA, Crain CM. 2009. Northeastern North American case studies – New Jersey and New England. *In:* Barendregt A, Whigham DF, Baldwin AH. (eds.), Tidal Freshwater Wetlands. Backhuys Publishers, Leiden, The Netherlands.

Meyerson LA, Saltonstall K, Windham L, Kiviat E, Findlay S. 2000. A comparison of *Phragmites australis* in freshwater and brackish marsh environments in North America. Wetlands Ecology and Management 8:89-103.

Mitsch WJ, Gosselink JG. 2000. Wetlands, 3rd edition. John Wiley and Sons.

Najjar RG, Walker HA, Anderson PJ, Barron EJ, Bord RJ, Gibson JR, Kennedy VS, Knight CG, Megonigal JP,O'Connor RE, Polsky CD, Psuty NP, Richards BA, Sorenson LG, Steele EM, Swanson RS. 2000. The potential impacts of climate change on the mid-Atlantic coastal region. Climate Research 14(3):219-33.

U.S. Environmental Protection Agency (EPA). 2016. National Rivers and Streams Assessment 2008-2009: A Collaborative Survey (EPA/841/R-16/007). Office of Water and Office of Research and Development.

Washington, DC. March 2016. https://www.epa.gov/sites/production/files/2016-03/documents/nrsa_0809_march_2_final.pdf.

Weston NB, Dixon RE, Joye SB. 2006. Ramifications of increased salinity in tidal freshwater sediments: geochemistry and microbial pathways of organic matter mineralization. Journal of Geophysical Research: Biogeosciences 111(G1).

Riverine Tidal Invasive Wetland

System: Riverine

Subsystem: Tidal

Class: Emergent Wetland

Sub-class: Invasive Wetland

Geographic Area: Entire Area

Overall Vulnerability Rank = Low

Habitat Sensitivity = Low

Climate Exposure = Moderate

	Riverine Tidal Invasive Wetland	Attribute Mean	Data Quality	Distribution of Expert Scores
Sensitivity Attributes	Habitat condition	1.6	1.8	
	Habitat fragmentation	1.6	2	
	Distribution/Range	1.5	1.8	
	Mobility/Ability to spread or disperse	1.8	2.3	
	Resistance	1.6	1.8	
	Resilience	1.3	1.8	
	Sensitivity to changes in abiotic factors	1.7	2.3	
	Sensitivity and intensity of non−climate stressors	1.9	2.3	
	Dependency on critical ecological linkages	1.2	2	
	Sensitivity Component Score	Low		
Exposure Factors	Sea surface temp	n/a	n/a	
	Bottom temp	n/a	n/a	
	Air temp	4	2.8	
	River temp	n/a	n/a	
	Surface salinity	n/a	n/a	
	Bottom salinity	n/a	n/a	
	pH	n/a	n/a	
	Sea level rise	2.6	2	
	Precipitation	n/a	n/a	
	Floods	2.9	1.9	
	Droughts	2.9	1.9	
	Exposure Component Score	Moderate		
**Overall Vulnerability Rank**		**Low**		

Low
Moderate
High
Very High

Habitat Name: Riverine Tidal Invasive Wetland

System**:** Riverine

Subsystem: Tidal

Class: Emergent Wetland

Sub-class: Invasive Wetland

Geographic Area: Entire Area

Habitat Description: This sub-class includes invasive riverine tidal freshwater wetlands, dominated by perennial plants (i.e., characterized by erect, rooted, herbaceous hydrophytes), where salinity is ≤0.5 ppt. This sub-class includes persistent and non-persistent wetlands within the influence of the tide.

Invasive tidal species include common reed (*Phragmites australis*) and reed canarygrass (*Phalaris arundinacea)*. Invasive tidal freshwater wetlands are found throughout the study region, associated with rivers that empty into estuaries.

Overall Climate Vulnerability Rank: Low (100% certainty from bootstrap analysis).

Climate Exposure: Moderate. The highest exposure mean was for Air Temperature (4.0), followed by Floods (2.9), Droughts (2.9), and Sea Level Rise (SLR) (2.6). Air temperature is projected to increase significantly across the range. Floods and droughts are projected to increase in magnitude and frequency in the Mid-Atlantic. In New England, floods are expected to increase in frequency but decrease in magnitude, and droughts are expected to increase in magnitude but decrease in frequency. SLR is projected to increase significantly throughout the range, although tidal freshwater wetlands are expected to be only moderately exposed to higher sea levels through saltwater intrusion and an inland moving salt wedge.

Habitat Sensitivity: Low. All sensitivity attributes were scored in the Low range, with none >2.0. Invasive wetlands are generally highly adapted to anthropogenic and climate disturbances.

Data Quality & Gaps: Three of the four climate exposure factor data quality scores were ≤2.0: SLR (2.0), and Floods and Droughts (both 1.9). Only Air Temperature (2.8) had a moderately high data quality score. Because of the fine-scale nature of riverine habitats and the coarse scale of this assessment, text descriptions of the spatial distribution of invasive tidal freshwater wetlands were used for the climate exposure scoring, which limited the ability of scorers to ascertain the overlap between the distribution of the habitat and climate exposure projections.

Six of the nine sensitivity attribute data quality scores were ≤2.0: Habitat Condition (1.8), Distribution/Range (1.8), Resilience (1.8), Resistance (1.8), Dependency on Critical Ecological Linkages (2.0), and Habitat Fragmentation (2.0).

Positive or Negative Climate Effect in the Northeast U.S.: The effect of climate change in riverine tidal invasive wetland in the Northeast U.S. is generally expected to be positive (80% of the experts' scores were positive, 15% were negative, 5% were neutral). Invasive tidal freshwater wetlands may benefit from climate change through competitive advantages over native species.

Climate Effects on Habitat Condition and Distribution: The common reed and other invasive freshwater wetland plants are generally well adapted to disturbance, and will likely benefit from climate change by outcompeting native species. In general, climate change is expected to benefit invasive species and exacerbate their impacts on ecosystems (Dukes and Mooney 1999).

Floods and droughts are projected to change significantly in the Northeast U.S. (Demaria et al. 2015), and sea level rise may also contribute to increased flooding of tidal freshwater wetlands. Invasive species respond to these changing conditions in various ways, and the response to these flow dynamics and SLR may be synergistic. For example, increased freshwater volume as a result of higher precipitation may counter SLR,

while decreased freshwater influx due to drought may amplify the effects of sea level and increase salinity levels further upstream. An increase in salinity may also increase microbialmineralization of organic matter, leading to a decrease in the stability of the marsh platform (Weston et al. 2006). In addition, drought causes water levels to drop and bare soil exposed for longer time periods, which allows seeds of invasive species to germinate and spread through vegetative propagation, resulting in expansion to freshwater marshes (Tougas-Tellier et al. 2015). These conditions present synergistic challenges for native species, as the increases in invasives like the common reed alter sediment dynamics building up vegetation, thus reducing water flow and impacting topography, which contributes to drying. Increasing frequency and intensity of droughts may also contribute to increased salinity. Increased flooding however, depending on rate and salinity may impact invasive colonization.

If increased riverine flow is temporary (e.g., after a storm event), hydrophytes should be tolerant of the higher water levels and could act as buffers to slow down the inundating water, which may cause sediment accretion on the marsh (Najjar et al. 2000). If the increased flow or water depth is long-term, then migration of plants landward or upstream may result. However, horizontal and upstream migration will be limited by steep slopes in the upper reaches of river systems and hardened shorelines (Najjar et al. 2000; Barendregt and Swarth 2013). Anthropogenic barriers are common in urban areas, and many urban areas are located on rivers. In New England, dams and culverts within the tidal zone limit upstream migration and cause loss of tidal freshwater wetlands (Leck and Crain 2009). Increased riverine flow of channelized rivers may also result in scour or erosion of riparian wetland edge.

Invasive tidal riverine wetlands are tolerant of most climate-related stressors. The common reed has a broad salinity tolerance (Meyerson et al. 2000), so will not be impacted by incursions of salt water caused by SLR and storm surge. Conversely, saltwater intrusion may facilitate the common reed expansion given its advantage over native species (Meyerson et al. 2000). Invasive reed canarygrass has shown tolerance to mildly saline but not hypersaline conditions in other parts of the United States (Waggy 2010), and dramatic increases in salinity may therefore impact its presence in the northeast.

Increases in temperature can lead to an increase in photosynthetic rates and plant biomass, as can increases in CO_2 (Kirwan and Blum 2011). Increased temperatures increase the production of soil organic matter, but also increase rates of decomposition (Kirwan and Blum 2011). However, the common reed stands accumulate detritus and have slower stem decomposition rates (Meyerson et al. 2000), so subsidence is less of a concern than it is in native marshes.

<u>Habitat Summary</u>**:** Invasive freshwater wetlands are dominated by a few species, but those species are highly adaptable and tolerant of suboptimal conditions (Meyerson et al. 2000). Invasive species share characteristics that make it easy for them to overtake pre-existing, native communities (Dukes and Mooney 1999). These include a high rate of population growth, which contributes to rapid colonization; ability to move long distances, which contributes to colonizing distant habitats; tolerance of close association with humans; and tolerance of a broad range of physical conditions (Rejmánek and Richardson 1996). Invasive species typically tolerate disturbed areas better than natives, and will colonize and outcompete natives (Mitsch and Gosselink 2000; Dahl and Stehman 2013). Since the traits of successful invaders tend to increase their resilience to a variety of climate and non-climate disturbances, the combination of these stressors will likely further reduce natives (Rogers and McCarty 2000). The common reed is able to establish in areas with significant human disturbance, including development, pollution, and mechanical disturbance (Meyerson et al. 2000). While coastal and riverine development has led to overall fragmentation for native riverine wetlands, invasive freshwater wetlands are not functionally harmed by fragmentation. However, shoreline hardening and other anthropogenic barriers may limit their ability to spread.

Literature Cited

Barendregt A, Swarth CW. 2013. Tidal freshwater wetlands: variation and changes. Estuaries and Coasts 36:445-56.

Dahl TE, Stedman S-M. 2013. Status and trends of wetlands in the coastal watersheds of the conterminous United States 2004 to 2009. U.S. Department of the Interior, Fish and Wildlife Service and National Oceanic and Atmospheric Administration, National Marine Fisheries Service. p. 1-46.

Demaria EMC, Palmer RN, Round JK. 2015. Regional climate change projections of streamflow characteristics in the Northeast and Midwest U.S. Journal of Hydrology: Regional Studies 5:309-23.

Dukes JS, Mooney HA. 1999. Does global change increase the success of biological invaders? Trends in Ecology and Evolution 14:135-9.

Kirwan ML, Blum LK. 2011. Enhanced decomposition offsets enhanced productivity and soil carbon accumulation in coastal wetlands responding to climate change. Biogeosciences 8(4):987-93.

Leck MA, Crain CM. 2009. Northeastern North American case studies – New Jersey and New England. *In:* Barendregt A, Whigham DF, Baldwin AH. (eds.), Tidal Freshwater Wetlands. Backhuys Publishers, Leiden, The Netherlands.

Meyerson LA, Saltonstall K, Windham L, Kiviat E, Findlay S. 2000. A comparison of *Phragmites australis* in freshwater and brackish marsh environments in North America. Wetlands Ecology and Management 8:89-103.

Mitsch WJ, Gosselink JG. 2000. Wetlands, 3rd edition. John Wiley and Sons.

Najjar RG, Walker HA, Anderson PJ, Barron EJ, Bord RJ, Gibson JR, Kennedy VS, Knight CG, Megonigal JP, O'Connor RE, Polsky CD, Psuty NP, Richards BA, Sorenson LG, Steele EM, Swanson RS. 2000. The potential impacts of climate change on the mid-Atlantic coastal region. Climate Research 14(3):219-33.

Rejmánek M, Richardson DM. 1996. What attributes make some plant species more invasive? Ecology 77(6):1655-61. https://esajournals.onlinelibrary.wiley.com/doi/epdf/10.2307/2265768

Rogers CE, McCarty JP. 2000. Climate change and ecosystems of the Mid Atlantic Region. Climate Research 14:235-44.

Tougas-Tellier MA, Morin J, Hatin D, Lavoie C. 2015. Freshwater wetlands: fertile grounds for the invasive *Phragmites australis* in a climate change context. Ecology and Evolution 5(16):3421-35.

Weston NB, Dixon RE, Joye SB. 2006. Ramifications of increased salinity in tidal freshwater sediments: geochemistry and microbial pathways of organic matter mineralization. Journal of Geophysical Research: Biogeosciences 111(G1).

Waggy MA. 2010. [Internet]. *Phalaris arundinacea*. *In:* Fire Effects Information System. U.S. Department of Agriculture, Forest Service, Rocky Mountain Research Station, Fire Sciences Laboratory. [accessed5 August 2020]. Available: https://www.fs.fed.us /database/feis/plants/graminoid/phaaru/all.html.

Riverine Tidal Native Wetland

System: Riverine

Subsystem: Tidal

Class: Emergent Wetland

Sub-class: Native Wetland

Geographic Area: Entire Area

Overall Vulnerability Rank = Moderate

Habitat Sensitivity = High

Climate Exposure = Moderate

	Riverine Tidal Native Wetland	Attribute Mean	Data Quality	Distribution of Expert Scores
Sensitivity Attributes	Habitat condition	3.3	2.5	
	Habitat fragmentation	2.8	2.5	
	Distribution/Range	1.8	2.3	
	Mobility/Ability to spread or disperse	2.9	2.5	
	Resistance	3.1	2	
	Resilience	2.5	1.8	
	Sensitivity to changes in abiotic factors	3.7	2.5	
	Sensitivity and intensity of non−climate stressors	3.6	2.8	
	Dependency on critical ecological linkages	1.3	2	
	Sensitivity Component Score	High		
Exposure Factors	Sea surface temp	n/a	n/a	
	Bottom temp	n/a	n/a	
	Air temp	4	2.8	
	River temp	n/a	n/a	
	Surface salinity	n/a	n/a	
	Bottom salinity	n/a	n/a	
	pH	n/a	n/a	
	Sea level rise	2.7	2	
	Precipitation	n/a	n/a	
	Floods	2.9	1.9	
	Droughts	2.9	1.9	
	Exposure Component Score	Moderate		
**Overall Vulnerability Rank**		**Moderate**		

Low
Moderate
High
Very High

Habitat Name: Riverine Tidal Native Wetland

System**:** Riverine

Subsystem: Tidal

Class: Emergent Wetland

Sub-class: Native Wetland

Geographic Area: Entire Area

Habitat Description: This sub-class includes native riverine tidal freshwater wetlands, dominated by perennial plants (i.e., erect, rooted, herbaceous hydrophytes), where salinity is ≤0.5 ppt. This sub-class includes persistent and non-persistent wetlands within the influence of the tide.

Tidal freshwater marshes tend to support a greater diversity of plants than in salt marshes, and include native species such as saltmeadow cordgrass (*Spartina patens*), marsh spikegrass (*Distichlis spicata*), saltmarsh rush (*Juncus gerardii*), arrow arum (*Peltandra virginica),* spadderdock *(Nuphar lutea),* cardinalflower (*Lobelia cardinalis),* turtlehead *(Chelone glabra),* water willow *(Justicia americana),* buttonbush (*Cephalanthus occidentalis*) pickerel weed (*Pondederia cordata),* sweetflag *(acorus calamus),* bulrushes *(Scirpus spp,)* and cattail *(Typha latifolia)*.

Tidal freshwater wetlands occur where rivers empty into the upper part of the estuaries and experience tides of up to several meters in amplitude twice a day. They occur at the interface between the brackish zone in the estuary and the river, and where brackish and freshwater mix is an area of maximum suspended matter (i.e., the maximum turbidity zone). The tidal freshwater zone plays an important role in overall patterns of nutrient cycling throughout the estuary. Although tidal freshwater wetlands do notinclude many endemic or restricted species, they are characterized by high species and habitat diversity.There is a distinct zonation in flora and fauna species, responding to the relationship between surface elevation and tidal amplitude (Barendregt et al. 2006).

Overall Climate Vulnerability Rank: Moderate (40% certainty from bootstrap analysis). The majority of the bootstrap results are in the High vulnerability rank (55%), with 5% in the Very High bin. This differs from the results of the Moderate categorical vulnerability rank. This result is due to the Floods and Droughts exposure factors being near the threshold triggering a High rank, indicating there is an increased likelihood that the vulnerability rank could be High.

Climate Exposure: Moderate. The highest exposure mean was for Air Temperature (4.0), followed by Floods (2.9), Droughts (2.9), and Sea Level Rise (SLR) (2.7). Air temperature is projected to increase significantly across the range. Floods and droughts are projected to increase in magnitude and frequency in the Mid-Atlantic. In New England, floods are expected to increase in frequency but decrease in magnitude, and droughts are expected to increase in magnitude but decrease in frequency. SLR is projected to increase significantly throughout the range, although tidal freshwater wetlands are expected to be only moderately exposed to higher sea levels through saltwater intrusion and an inlandmoving salt wedge.

Habitat Sensitivity: High. Four sensitivity attributes contributed to the High sensitivity score with scores ≥3.0: Sensitivity to Changes in Abiotic Factors (3.7), Sensitivity and Intensity of Non-Climate Stressors (3.6), Habitat Condition (3.3), and Resistance (3.1). Native freshwater tidal wetland habitat is sensitive to changing groundwater levels, saltwater intrusion, and to changes in the marsh platform that may result from higher temperatures. Its condition has been degraded by several current and historic anthropogenic activities including development and nonpoint source pollution.

Data Quality & Gaps: Three of the four climate exposure factor data quality scores were ≤2.0: SLR (2.0), and Floods and Droughts (both 1.9). Only Air Temperature (2.8) had a moderately high data quality score. Because of the fine-scale nature of riverine habitats and the coarse scale of this assessment, text descriptions of the spatial distribution of native tidal freshwater wetlands were used for the climate exposure scoring instead of maps of habitat distribution, which limited the ability of scorers to ascertain the overlap between

the distribution of the habitat and climate exposure projections.

Three of the nine sensitivity attribute data quality scores were ≤2.0: Resilience (1.8), Dependency on Critical Ecological Linkages (2.0), and Resistance (2.0).

Positive or Negative Climate Effect in the Northeast U.S.: The effect of climate change in riverine tidal native wetland in the Northeast U.S. is generally expected to be negative (65% of the experts' scores were negative, 20% were neutral, 15% were positive).

Climate Effects on Habitat Condition and Distribution: Dominant tidal freshwater marsh flora are eurythermal temperate species, but are sensitive to changes in hydrology, topography (elevation), and salinity. Tidal freshwater wetlands are also sensitive to tidal inundation, which determines marsh zonation. Any disturbance that affects these characteristics can therefore impact community structure,which dictates many marsh ecosystem functions.

Floods and droughts are projected to change (increase) significantly in the Northeast U.S. (Demaria et al. 2015). If increased riverine flow is temporary (e.g., after a storm event), hydrophytes should be tolerant of the higher water levels and could act as buffers to slow down the inundating water, which may provide beneficial sediment accretion on wetlands (Najjar et al. 2000). Sea level rise may also contribute to increased flooding of tidal freshwater wetlands. If the increased flow or water depth is long-term, then migration of plants landward or upstream may result. However, horizontal and upstream migration will be limited by steep slopes in the upper reaches of river systems and hardened shorelines (Najjar et al. 2000; Barendregt and Swarth 2013). Anthropogenic barriers are common in urban areas, and many urban areas are located on rivers. In New England, dams and culverts within the tidal zone limit upstream migration of the marsh and result in a loss of tidal freshwater wetlands (Leck and Crain 2009). Increased riverine flow may also result in scour of the marsh edge.

Sea level rise and storm surge will result in increased incursion of saltwater to tidal freshwater wetlands, leading to physiological stress and potential die-off of freshwater hydrophytes. There is evidence that tidal freshwater wetlands in Maryland and Virginia are changing in ways that suggest that one of the driving factors is an increase in inundation frequency and salinity (Perry et al. 2009). The ability of tidal freshwater wetlands to keep up with increases in the rate of SLR may be limited. Under a 0.82 m SLR scenario (this study is using a 1-m global SLR), tidal freshwater wetlands are expected to decrease by 39% due to saltwater intrusion and migration of brackish marshes inland (Craft et al 2009). While SLR can erode coastal marshes, tidal freshwater wetlands receive influxes of riverine sediments, which reduces their vulnerability to SLR as compared with salt marshes (Najjar et al. 2000).

Increasing frequency and intensity of droughts may also contribute to increased salinity. An increase in salinity may also increase microbial mineralization of organic matter, leading to a decrease in the stability of the marsh platform (Weston et al. 2006). High salinity also decreases plant productivity (Neubauer et al. 2013). In low salinity, brackish waters, increased evaporation due to higher temperatures and drought may result in abnormally higher salinity regimes, which may reduce the diversity of aquatic macrophytes (Brock and van Vierssen, 1992).

Tidal freshwater wetlands can recover from natural disturbance under optimal conditions, especially with restoration efforts. However, recovery depends on the level of disturbance (e.g., clear cutting but leaving roots allows faster recovery), hydrology, and other factors (Mitsch and Gosselink 2000). Competition with non-native and invasive species is also an issue and can limit the recovery of native species after a disturbance.

As temperatures increase, southern tidal freshwater wetland species may shift northward (Barendregt and Swarth 2013). Increases in temperature can also lead to an increase in photosynthetic rates and plant biomass, as can increases in CO_2 (Kirwan and Blum 2011), and changes in marsh community composition. Increased temperatures also increase the production of soil organic matter, but increased rates of decomposition as a result of warmer temperatures will likely outweigh the soil accumulation causing marsh loss (Kirwan and

Blum 2011). Increased edge erosion can result from a greater porosity of the marsh platform from increased decomposition.

Habitat Summary: Tidal freshwater wetland condition is driven by historic practices like log drives and land conversion to agriculture, as well as current anthropogenic activities (e.g., land development, point and non-point pollution). Tidal freshwater wetlands are often located near urbanized areas, and most freshwater wetlands in the region experience chronic and long-term habitat degradation (Barendregt and Swarth 2013). According to the 2008-2009 National Wetland Condition Assessment report, compacted soil, ditching, and loss of vegetation are the most important anthropogenic impacts in U.S. freshwater wetlands (USEPA 2016). Other anthropogenic impacts include development, dredging, filling, sediment contamination, and invasives (e.g., purple loosestrife). Coastal and riverine development has led to a high degree of habitat fragmentation, especially in urbanized regions. Invasives typically tolerate disturbed areas better than natives, and will colonize and outcompete natives (Mitsch and Gosselink 2000; Dahl and Stehman 2013). Increased eutrophication from nutrient runoff can also lead to increased decomposition rates in marsh plants, leading to increased porosity in the marsh platform and an elevated risk of edge erosion from floods and SLR.

Literature Cited

Barendregt A, Swarth CW. 2013. Tidal freshwater wetlands: variation and changes. Estuaries and Coasts 36:445-56.

Barendregt A, Whigham DF, Meire P, Baldwin AH, Van Damme S. 2006. Wetlands in the tidal freshwater zone. *In:* Bobbink R, Beltman B, Verhoeven JTA, Whigham DF (eds), Wetlands: Functioning, Biodiversity, Conservation and Restoration, Ecological Studies (Analysis and Synthesis) 191. Springer, Berlin, Heidelberg.

Brock TCM, van Vierssen W. 1992. Climate change and hydrophyte-dominated communities in inland wetland ecosystems. Wetlands Ecology and Management 2(1-2):37-49.

Craft C, Clough J, Ehman J, Joye S, Park R, Pennings S, Guo H, Machmuller M. 2009. Forecasting the effects of accelerated sea-level rise on tidal marsh ecosystem services. Frontiers in Ecology and the Environment 7(2):73-8.

Dahl TE, Stedman S-M. 2013. Status and trends of wetlands in the coastal watersheds of the conterminous United States 2004 to 2009. U.S. Department of the Interior, Fish and Wildlife Service and National Oceanic and Atmospheric Administration, National Marine Fisheries Service. 1-46.

Demaria EMC, Palmer RN, Round JK. 2015. Regional climate change projections of streamflow characteristics in the Northeast and Midwest U.S. Journal of Hydrology: Regional Studies 5:309-23.

Kirwan ML, Blum LK. 2011. Enhanced decomposition offsets enhanced productivity and soil carbon accumulation in coastal wetlands responding to climate change. Biogeosciences 8(4):987-93.

Leck MA, Crain CM. 2009. Northeastern North American case studies – New Jersey and New England. *In:* Barendregt A, Whigham DF, Baldwin AH. (eds.), Tidal Freshwater Wetlands. Backhuys Publishers, Leiden, The Netherlands.

Mitsch WJ, Gosselink JG. 2000. Wetlands, 3rd edition. John Wiley and Sons.

Najjar RG, Walker HA, Anderson PJ, Barron EJ, Bord RJ, Gibson JR, Kennedy VS, Knight CG, Megonigal JP, O'Connor RE, Polsky CD, Psuty NP, Richards BA, Sorenson LG, Steele EM, Swanson RS. 2000. The potential impacts of climate change on the mid-Atlantic coastal region. Climate Research 14(3):219-33.

Neubauer SC, Franklin RB, Berrier DJ. 2013. Saltwater intrusion into tidal freshwater marshes alters the biogeochemical processing of organic carbon. Biogeosciences 10:8171-83.

Perry JE, Bilkovic DM, Havens KJ, Hershner CH. 2009. Chapter 14. Tidal freshwater wetlands of the Mid-Atlantic and southeastern United States. *In:* Tidal Freshwater Wetlands. Barendregt A, Whigham D, Baldwin A (eds). 157-66.

U.S. Environmental Protection Agency (EPA). 2016. National Rivers and Streams Assessment 2008-2009: A Collaborative Survey (EPA/841/R-16/007). Office of Water and Office of Research and Development. Washington, DC. March 2016. https://www.epa.gov/sites/production/files/2016-03/documents/nrsa_0809_march_2_final.pdf

Weston NB, Dixon RE, Joye SB. 2006. Ramifications of increased salinity in tidal freshwater sediments: geochemistry and microbial pathways of organic matter mineralization. Journal of Geophysical Research: Biogeosciences 111(G1).

Riverine Algae

System: Riverine

Subsystem: Tidal & Non-tidal

Class: Aquatic Bed

Sub-class: Algae

Geographic Area: Entire Area

Overall Vulnerability Rank = Low

Habitat Sensitivity = Low

Climate Exposure = High

	Riverine Algae	Attribute Mean	Data Quality	Distribution of Expert Scores
Sensitivity Attributes	Habitat condition	1.5	2.3	
	Habitat fragmentation	1.4	2.3	
	Distribution/Range	1.5	2.5	
	Mobility/Ability to spread or disperse	1.4	1.8	
	Resistance	2	1.8	
	Resilience	1.3	2	
	Sensitivity to changes in abiotic factors	1.2	2	
	Sensitivity and intensity of non−climate stressors	1.6	1.8	
	Dependency on critical ecological linkages	1.7	2.3	
	Sensitivity Component Score	Low		
Exposure Factors	Sea surface temp	n/a	n/a	
	Bottom temp	n/a	n/a	
	Air temp	n/a	n/a	
	River temp	3.2	2	
	Surface salinity	n/a	n/a	
	Bottom salinity	n/a	n/a	
	pH	n/a	n/a	
	Sea level rise	1.9	2	
	Precipitation	n/a	n/a	
	Floods	3	1.9	
	Droughts	2.9	1.9	
	Exposure Component Score	High		
**Overall Vulnerability Rank**		**Low**		

Low
Moderate
High
Very High

Habitat Name: Riverine Algae

System: Riverine

Subsystem: Tidal and Non-Tidal

Class: Aquatic Bed

Sub-class: Algal Bed

Geographic Area: Entire Area

Habitat Description: This sub-class includes riverine macroalgae distributed in tidal freshwater environments (< 0.5 ppt) and non-tidal environments (0 ppt), terminating where the channel enters a lake. Macroalgae species are photosynthetic plants and generally fall into one of several groups of multicellular algae: red, green and brown. Although red and brown algae occur in brackish water habitats, green algae are more common in freshwater rivers and streams. Macroalgae provide habitat foraquatic organisms (e.g., protection, nursery areas) and can serve as indicators of local water quality conditions (Moore 2009). A number of filamentous, green algae species occur in tidal and non-tidal portions of rivers including *Spirogyra* sp. and *Cladophora* sp., and two common freshwater green algae species, muskgrass (*Chara* sp.) and brittle grass (*Nitella* sp.), occur throughout the study area (Moore 2009).

Overall Climate Vulnerability Rank: Low (100% certainty from bootstrap analysis).

Climate Exposure: High. The overall High exposure score was influenced by mean scores for River Temperature (3.2), Floods (3.0), and Droughts (2.9). The mean score for Sea Level Rise (SLR) was the lowest (1.9), and were spread over Low, Moderate, and High scoring bins. This likely reflects the differential effect that SLR may have between tidal and non-tidal sections of riverine habitats. Likewise, the scores for both Floods and Droughts were spread over the Moderate, High, and Very High scoring bins, which likely reflects the large range of projected changes in precipitation that affect floods and droughts over the study area.

Habitat Sensitivity: Low. Eight of nine of the sensitivity attributes means were ≤1.7, with Resistance being the highest at 2.0. This suggests that riverine algal beds are generally believed to be resistant to climate and non-climate perturbations.

Data Quality & Gaps: Data quality scores for exposure factors were Low, with scores ≤2.0, suggesting that data gaps may exist. Spatial data for riverine macroalgae was lacking in the assessment, which limited the ability of scorers to ascertain the overlap between the distribution of the habitat and climate exposure projections. In addition, the considerable geographic variability of floods and droughts throughout the study area likely contributed to uncertainty in the habitat's exposure and the low data quality scores.

Habitat sensitivity data quality scores ranged from Low to Moderate (1.8 to 2.5). The three lowest scores(1.8) were found within Mobility/Ability to Spread or Disperse, Resistance, and Sensitivity and Intensity of Non-Climate Stressors. The Moderate scores included Distribution/Range (2.5) and Habitat Condition, Habitat Fragmentation, and Dependency on Critical Ecological Linkages (all 2.3). These scores likely reflect the view that riverine macroalgae habitat is generally in good condition and is likely not sensitive to climate change.

Positive or Negative Climate Effect in the Northeast U.S. Shelf: The expected effect of climate change on algal beds in the study area is positive with 60% of the tallies as positive, 35% negative and 5% neutral, as determined by expert scoring.

Climate Effects on Habitat Condition and Distribution: The distribution of algae appears broad across the study area, with some species restricted to warmer Mid-Atlantic waters, while others found in cooler New England waters. In general, there is no leading or trailing edge for this group with no positive or negative trends observed (Moore 2009; Valiela et al. 1997).

Most macroalgae in riverine environments grow in highly dynamic areas (e.g., variable temperature and flow) and are relatively resistant to natural disturbance. Because climate exposure is variable throughout the study

area, it is difficult to definitively assess the vulnerability of this habitat. For example, using the CMIP5 RCP8.5 scenario, a climate assessment by Lehmann et al. (2014) projected increases in frequency and intensity of winter extratropical cyclone events in the Northeast region, but a slight decrease in summer events. Colle et al. (2013) projected extratropical cyclones may become more intense (10-40%) along the northeast coast, especially during the mid-21st century. At this time, exposure to these events is less frequent, until the periodicity increases it is unclear whether macroalgae species will experience disruption or scouring that may affect their distribution and abundance in the study area.

Additional impacts from climate change include an increase in CO_2 concentrations and ocean acidification. The response by macroalgae to changes in CO_2 concentrations and carbonate chemistry is complex and not fully understood but is at least partially dependent upon the photosynthetic pathways used by the plant species (Young and Gobler 2016). Most marine macroalgae species use the C3 photosynthetic pathway, which results in lower rates of photosynthesis under current pCO_2 levels compared to C4 plants. However, under elevated pCO_2 conditions, macroalgae species may benefit through higher photosynthetic and growth rates while C4 plants may not (Young and Gobler 2016). Gledhill et al. (2015) found 10 of the 14 marine macroalgae species examined from the Gulf of Maine region showed increased growth rates and biomass under elevated pCO_2. However, it is unclear if freshwater and tidal-fresh macroalgae species have similar responses.

It is uncertain how riverine macroalgae may respond to changing frequency and intensity of floods and droughts. Increasing droughts may have particular effects in small headwaters, especially for ephemeral or intermittent streams, which can have downstream impacts as these habitats go dry (Brooks 2009). Low flow or a complete lack of water will likely adversely affect macroalgae found in those streams.

Significant increases in stream and river temperatures have been reported in the study area, with the highest observed rates of warming in the more urbanized areas of the Mid-Atlantic (Kaushal et al. 2010). Other measures of river and stream temperature conditions in the northeast are changing, including ice thickness (Huntington et al. 2003), dates of spring ice-out and ice-affected flow (Hodgkins et al. 2002; Hodgkins et al. 2003; Hodgkins and Dudley 2006), and seasonal stream runoff volume (Hodgkins and Dudley 2005). Stream temperatures are projected to increase in the study area by the end of the century (Letcher et al. 2016), which may favor macroalgae adapted to warmer water conditions and disfavor colder water species.

As a result of these projected changes in precipitation and water temperature, fragmentation and patchiness may be more frequent and widespread. However, fragmentation is a natural characteristic of most macroalgae species along with their ability to spread and disperse.

Mobility coupled with their tolerance to higher nutrient levels and temperature have resulted in some species (e.g. *Spirogyra* sp., *Cladophora* sp., muskgrass, and brittle grass) outcompeting rooted vascular beds and reaching nuisance levels in some urbanized environments (Valiela et al. 1997; Moore 2009). Runoff from these urbanized areas may provide a nutrient benefit, but increased sediment inputs may increase turbidity and reduce light availability, and can impact other water quality parameters that macroalgae may be sensitive to (Valiela et al. 1997; Moore 2009).

Habitat Summary: Potential climate impacts seem low at this time across the study area based on tolerances observed within algal species across riverine environments. Red and green algae species are naturally widespread, which is aided by their tolerance to higher nutrient levels and temperature (Moore 2009; Valiela et al. 1997). Higher concentrations of CO_2 could potentially benefit non-calcifying, photosynthetic organisms, including riverine macroalgae (Doney et al. 2009; Koch et al. 2013; Young and Gobler 2016). Riverine algal species are restricted to the shallow, photic zone, but most species are less sensitive to low light levels compared to rooted vascular bed species within the same environments.

It is unclear if the increase in intensity and frequency of storms under climate change scenarios will havean impact on these algae species. Most macroalgae that grow in highly dynamic areas (e.g., variable temperature and flow) are relatively resistant to natural disturbances. Increases in wave energy exposure could have deleterious effects to shallow-water habitats. Based on the results of CMIP5 RCP8.5, Lehmann et al. (2014)

concluded that the northeast region would likely experience an increase in the frequency and intensity of winter storm events, but it is unclear what impacts these may have on macroalgae species within the riverine environment.

Literature Cited

Brooks RT. 2009. Potential impacts of global climate change on the hydrology and ecology of ephemeral freshwater systems of the forests of the northeastern United States. Climatic Change 95469-83. https://doi.org/10.1007/s10584-008-9531-9

Colle BA, Zhang Z, Lombardo KA, Chang E, Liu P, Zhang M. 2013. Historical evaluation and future prediction of eastern North American and western Atlantic extratropical cyclones in the CMIP5 models during the cool season. Journal of Climate 26(18):6882-903.

Doney SC, Fabry VJ, Feely RA, Kleypas JA. 2009. Ocean acidification: the other CO_2 problem. Annual Review of Marine Science 1:169-92.

Gledhill DK, White MM, Salisbury J, Thomas H, Mlsna I, Liebman M, Mook B, Grear J et al. 2015. Ocean and coastal acidification off New England and Nova Scotia. Oceanography 28(2):182-97. http://dx.doi.org/10.5670/oceanog.2015.41

Hodgkins GA, Dudley RW. 2006. Changes in the timing of winter–spring streamflows in eastern North America, 1913–2002. Geophysical Research Letters 33(6):1-5.

Hodgkins GA, James IC, Huntington TG. 2002. Historical changes in lake ice-out dates as indicators of climate change in New England, 1850-2000. International Journal of Climatology 22(15):1819-27.

Hodgkins GA, Dudley RW, Huntington TG. 2003. Changes in the timing of high river flows in New England over the 20th Century. Journal of Hydrology 278(1-4):244-52.

Hodgkins GA, Dudley RW. 2005. Changes in the magnitude of annual and monthly streamflows in New England, 1902-2002. U.S. Department of the Interior, U.S. Geological Survey. Scientific Investigations Report 2005-5135:1-37.

Huntington TG, Hodgkins GA, Dudley RW. 2003. Historical trend in river ice thickness and coherence in hydroclimatological trends in Maine. Climatic Change 61(1):217-36.

Kaushal SS, Likens GE, Jaworski NA, Pace ML, Sides AM, Seekell D, Belt KT, Secor DH, Wingate RL. 2010. Rising stream and river temperatures in the United States. Frontiers in Ecology and the Environment 8(9):461-6.

Koch M, Bowes G, Ross C, Zhang X-H. 2013. Climate change and ocean acidification effects on seagrasses and marine macroalgae. Global Change Biology 19(1):103-32.

Lehmann J, Coumou D, Frieler K, Eliseev AV, Levermann A. 2014. Future changes in extratropical storm tracks and baroclinicity under climate change. Environmental Research Letters 9:8.

Letcher BH, Hocking DJ, O'Neil K, Whiteley AR, Nislow KH, O'Donnell MJ. 2016. A hierarchical model of daily stream temperature using air-water temperature synchronization, autocorrelation, and time lags. PeerJ 4:e1727. doi:10.7717/peerj.1727.

Moore KA. 2009. Submerged aquatic vegetation of the York River. Journal of Coastal Research SI 57:50-8.

Valiela I, McClelland J, Hauxwell J, Behr PJ, Hersh D, Foreman K. 1997. Macroalgal blooms in shallow estuaries: Controls and ecophysiological and ecosystem consequences. In: Part 2. The Ecology and Oceanography of Harmful Algal Blooms. Limnology and Oceanography 42(5):1105-18.

Young CS, Gobler CJ. 2016. Ocean acidification accelerates the growth of two bloom-forming macroalgae. PLoS ONE 11(5):e0155152. doi:10.1371/journal.pone.0155152

Riverine Submerged Aquatic Vegetation

System: Riverine

Subsystem: Tidal & Non-tidal

Class: Aquatic Bed

Sub-class: Rooted Vascular

Geographic Area: Entire Area

Overall Vulnerability Rank = High

Habitat Sensitivity = High

Climate Exposure = High

	**Riverine Submerged Aquatic Vegetation**	Attribute Mean	Data Quality	Distribution of Expert Scores
Sensitivity Attributes	Habitat condition	2.6	2.3	
	Habitat fragmentation	2.7	2.3	
	Distribution/Range	2.8	2.3	
	Mobility/Ability to spread or disperse	2	1.8	
	Resistance	3.3	2.3	
	Resilience	3.2	2.5	
	Sensitivity to changes in abiotic factors	2.5	2.3	
	Sensitivity and intensity of non−climate stressors	3.4	2.8	
	Dependency on critical ecological linkages	1.7	1.8	
	Sensitivity Component Score	High		
Exposure Factors	Sea surface temp	n/a	n/a	
	Bottom temp	n/a	n/a	
	Air temp	n/a	n/a	
	River temp	3.2	2	
	Surface salinity	n/a	n/a	
	Bottom salinity	n/a	n/a	
	pH	n/a	n/a	
	Sea level rise	1.9	2	
	Precipitation	n/a	n/a	
	Floods	3	1.9	
	Droughts	3	1.9	
	Exposure Component Score	High		
**Overall Vulnerability Rank**		**High**		

Low
Moderate
High
Very High

Habitat Name: Riverine Submerged Aquatic Vegetation

System**:** Riverine

Subsystem: Tidal and Non-Tidal

Class: Aquatic Bed

Sub-class: Rooted Vascular

Geographic Area: Entire Area

Habitat Description: This sub-class includes riverine rooted vascular bed distributed in tidal freshwater environments (< 0.5 ppt) and non-tidal environments (0 ppt), terminating where the channel enters a lake. Rooted vascular plants provide habitat for aquatic organisms (e.g., protection for predators, nursery areas) and can serve as indicators of local water quality conditions (Moore 2009). Rooted vascular plant species found within the tidal freshwater portions of the riverine system includes widgeongrass (*Ruppia maritima*) – a freshwater plant that is tolerant of both fresh and saltwater and wild celery (*Vallisneria americana*). In addition, the pondweed community, including sago pondweed (*Stuckenia pectinata*) and redhead grass (*Potamogeton perfoliatus*), and invasives such as hydrilla (*Hydrilla verticillata*), are tolerant of somewhat higher salinities which can be variable based on seasonal flows. Beyond tidal influence, in freshwater, vascular plants include water stargrass (*Heteranthera dubia*), widgeon grass, wild celery, Eurasian watermilfoil (*Myriophyllum spicatum*), and hydrilla (Doering et al.2001; Moore 2009; ASMFC 2018; Flora of North America 2019).

Overall Climate Vulnerability Rank: High (100% certainty from bootstrap analysis).

Climate Exposure: High. The overall High exposure score was influenced by mean scores for River Temperature (3.2), Floods (3.0), and Droughts (3.0). The mean score for Sea Level Rise (SLR) was the lowest (1.9), and the scores were spread over Low, Moderate, and High bins. This likely reflects the differential effect that SLR may have between tidal and non-tidal sections of riverine habitats. Likewise, the scores for both Floods and Droughts were spread over the Moderate, High, and Very High bins, which likely reflects the large range of projected changes in precipitation that affect floods and droughts over the study area.

Habitat Sensitivity: High. All nine of the sensitivity attributes means were ≥1.7, and three were ≥3.2, indicating that most rooted vascular plants are Moderate to Highly sensitive to climate and non-climate perturbations. The highest sensitivity means were Sensitivity and Intensity of Non−Climate Stressors (3.4), Resistance (3.3), and Resilience (3.2), indicating this habitat is currently vulnerable to anthropogenic stressors. The Moderate to High sensitivity means for Habitat Condition, Habitat Fragmentation, and Distribution/Range suggests some of the species in this habitat sub-class may have been considered less sensitive to stressors (e.g., freshwater species with salinity tolerances, invasive species).

Data Quality & Gaps: Data quality scores for climate exposure factors were Low, with scores found ≤2.0. The low data quality scores suggest that data gaps exist. Spatial data for riverine rooted vascular plants was lacking in the assessment, which limited the ability of scorers to ascertain the overlap between the distribution of the habitat and climate exposure projections. In addition, the considerable geographic variability of floods and droughts throughout the study area likely contributed to uncertainty in the habitat's exposure and the low data quality scores.

Data quality scores for habitat sensitivity ranged from Low to Moderate (1.8 to 2.8). The two lowest scores (1.8) were in Mobility/Ability to Spread and Disperse, and Dependency on Critical Ecological Linkages. The remaining Moderate scores for data quality may reflect some uncertainty by the scorers in sensitivity due to the variable responses of species included in this sub-class, particularly the invasive species that may benefit from climate change.

Positive or Negative Climate Effect in the Northeast U.S. Shelf: The expected effect of climate change on rooted vascular bed plants in the study area is negative with 25% of the tallies as neutral and 75% as negative, as determined by expert scoring.

Climate Effects on Habitat Condition and Distribution: Vascular plants are sensitive to a number of existing and projected climate-related and non-climate stressors. Beyond physical and chemical disruption from increased urbanization and development, climate stressors for riverine rooted vascular plants include higher water temperature, low dissolved oxygen, salinization from higher sea levels, and more extreme weather events, including droughts and floods. Climate exposure for some species will be adverse, while others may be able to adapt, but in general this group is not resilient to disturbance and it may take years to decades to recover from climate and non-climate perturbations (Neckles et al. 1993;Short and Neckles 1999; Björk et al. 2008).

Sedimentation and turbidity will be exacerbated by climate-driven impacts, including increases in mean and extreme precipitation events and related impacts (e.g., erosion). Sediment transport and associated impacts from development (e.g., elevated temperature, low dissolved oxygen, turbidity, nutrients) will challenge vascular plants within riverine environments (Kantrud 1991). Unlike the native rooted vascular plants, non-native species (e.g., hydrilla, watermilfoil, muskgrass, and brittle grass) generally have higher tolerances to disturbance, poor water quality, and increased salinities, and therefore, greater opportunity for expansion (Valiela et al. 1997; Moore 2009).

Beyond impacts of poor water quality threatening these species, other considerations of climate change impacts are patterns of increased intensity and frequency of weather events (Short and Wyllie-Echeverria 1996; Duarte 2002; Fourqurean and Rutten 2004; Lehmann et al. 2014; USGCRP 2017),which may pose a threat to the current distribution of these species. Drought might have particular effect in small headwaters, especially for ephemeral or intermittent streams (Brooks 2009). Tidal and freshwater species are already moderately to highly fragmented throughout their range as a result of urbanization in some river systems. Although widgeon grass and wild celery can spread rapidly, it can also be fragmented easily by storms and degradation in water quality (Kantrud 1991; Moore 2009).

Most native riverine rooted vascular plants are not tolerant of high water temperature. For example, the upper temperature threshold of wigeon grass is about 30°C, although tolerances for flowering and germination is much lower (Kantrud 1991). Non-native species such as hydrilla have expanded its distribution in the Mid-Atlantic as a result of their tolerances to higher temperatures and eutrophic waters compared to native species (Moore 2009; Flora of North America 2019).

Riverine rooted vascular plants may benefit from increased CO_2 in riverine systems because the photosynthetic rates of these plants appear to be limited by the availability of inorganic carbon (Thom 1996; Alexandre et al. 2012). Mesocosm experiments using elevated CO_2 concentrations have shown higher photosynthetic rates and efficiencies compared to plants exposed to current levels. However, most experiments have been conducted using marine rooted vascular plants (Thom 1996; Zimmerman et al. 1997; Palacios and Zimmerman 2007; Alexandre et al. 2012), and it is unclear if freshwater specieswill respond similarly. In addition, few studies have investigated potential synergetic effects of CO_2 enhancement with other factors such as warming waters, eutrophication, or reduced light conditions. The response of seagrasses to higher-CO_2 conditions may be highly species specific, and dependent upon geochemical characteristics of the environment (Apostolaki et al. 2014).

Habitat Summary: The current range and distribution of native, riverine rooted vascular beds have declined over the past several decades in the Mid-Atlantic and New England region as a result of declining water quality and in areas of increased development (Moore 2009). Shoreline hardening and boat docks and piers are known to adversely affect rooted vascular bed species through increasing waveenergy and shading (Unsworth et al. 2015). Native rooted vascular beds in riverine systems have been displaced by macroalgae (Valiela et al. 1997) and invasive species (e.g., hydrilla, watermilfoil) in highly-urbanized coastal and riverine systems (Short and Wyllie-Echeverria 1996; Moore 2009).

Although widgeon grass can spread rapidly and has recovered and expanded its distribution in some rivers, it can be easily uprooted by storms and does not grow well in turbid water or low-oxygen substrates (Moore

2009; Kantrud 1991). Widgeon grass may be expanding in New England due to the species' tolerance to lower light and higher nitrogen levels (P. Colurusso, pers. comm). The effects of climate change and impacts will result in a variety of challenges for aquatic ecosystems which rooted vascular plants occupy. The distribution of non-native and invasive species such as hydrilla and watermilfoil in freshwater and low-salinity waters is likely to increase, and replace native rooted vascular bed species. Many species of rooted vascular plants are sensitive to increases in temperature, but are more susceptible to degradation of their habitats. The primary climate change threats to riverine, rooted vascular bed habitat include salinization through higher sea levels, high flow events, and droughts, all of which can impact water quality. The tolerance of the riverine rooted vascular plants to increased salinity are variable, some of which include widgeon grass , wild celery, some pond weeds including sago pondweed and redhead grass (Doering et al. 2001; Moore 2009; ASFMC 2018).

Although these species have the ability spread via sexual (flowering and seeds) and asexual (rhizomes) reproduction, their success is dependant on habitat conditions where they settle. Natural disturbance and anthropogenic impacts can result in fragmentation (Kantrud 1991; Moore 2009; Unsworth et al. 2015) and challenge recolonization of these native plants, which can take years to establish. These plants can be replaced by more tolerant non-native species (Short and Wyllie-Echeverria 1996; Moore 2009).

Literature Cited

Alexandre A, Silva J, Buapet P, Björk M, Santos R. 2012. Effects of CO_2 enrichment on photosynthesis, growth, and nitrogen metabolism of the seagrass *Zostera noltii*. Ecology and Evolution 2(10):2625-35.

Apostolaki ET, Vizzini S, Hendriks IE, Olsen YS. 2014. Seagrass ecosystem response to long-term high CO_2 in a Mediterranean volcanic vent. Marine Environmental Research 99:9-15.

Atlantic States Marine Fisheries Commission (ASMFC). 2018. Submerged aquatic vegetation policy: a review of past accomplishments and emerging research and management issues. Atlantic StatesMarine Fisheries Commission. Habitat Management Series #15, Spring 2018.

Björk M, Short F, Mcleod E, Beer S. 2008. Managing seagrasses for resilience to climate change. The International Union for the Conservation of Nature and Natural Resources (IUCN)/The Nature Conservancy. p. 1-56.

Brooks RT. 2009. Potential impacts of global climate change on the hydrology and ecology of ephemeral freshwater systems of the forests of the northeastern United States. Climatic Change 95:469–83. https://doi.org/10.1007/s10584-008-9531-9

Doering P, Chamberlain R, McMunigal J. 2001. Effects of simulated saltwater intrusions on the growth and survival of wild celery, *Vallisneria americana*, from the Caloosahatchee estuary (South Florida).Estuaries 24(6):894-903. http://www.jstor.org/stable/1353180

Duarte CM. 2002. The future of seagrass meadows. Environmental Conservation 29(02):192-206. Flora of North America. 2019. [Internet]. *Hydrilla verticillata*. (accessed 8 Dec. 2020). http://www.efloras.org/florataxon.aspx?flora_id=1&taxon_id=200024721

Fourqurean JW, Rutten LM. 2004. The impact of Hurricane Georges on soft-bottom, back reef communities: site- and species-specific effects in south Florida seagrass beds. Bulletin of MarineScience 75(2):239-57.

Kantrud HA. 1991. Wigeongrass (*Ruppia maritima* L.): a literature review. U.S. Fish and Wildlife Service. Fish and Wildlife Research 10. 58 pp.

Lehmann J, Coumou D, Frieler K, Eliseev AV, Levermann A. 2014. Future changes in extratropical storm tracks and baroclinicity under climate change. Environmental Research Letters 9:8.

Moore KA. 2009. Submerged aquatic vegetation of the York River. Journal of Coastal Research SI57:50-8.

Neckles HA, Wetzel RL, Orth RJ. 1993. Relative effects of nutrient enrichment and grazing on epiphyte-macrophyte (*Zostera marina* L.) dynamics. Oecologia 93(2):285-95.

Palacios SL, Zimmerman RC. 2007. Response of eelgrass *Zostera marina* to CO_2 enrichment: possible impacts of climate change and potential for remediation of coastal habitats. Marine Ecology Progress Series 344:1-13.

Short FT, Wyllie-Echeverria S. 1996. Natural and human-induced disturbance of seagrasses. Environmental Conservation 23(1):17-27.

Short FT, Neckles HA. 1999. The effects of global climate change on seagrasses. Aquatic Botany 63:169-96.

Thom RM. 1996. CO_2-Enrichment effects on eelgrass (*Zostera marina* L.) and bull kelp (*Nereocystis luetkeana* (Mert.) (P. & R.). Water, Air, and Soil Pollution 88(3):383-91.

United States Global Change Research Program (USGCRP). 2017: Climate Science Special Report: Fourth National Climate Assessment, Volume I [Wuebbles, D.J., D.W. Fahey, K.A. Hibbard, D.J. Dokken, B.C. Stewart, and T.K. Maycock (eds.)]. U.S. Global Change Research Program, Washington, DC,USA, 470 pp. doi: 10.7930/J0J964J6.

Unsworth RKF, Collier CJ, Waycott M, Mckenzie LJ, Cullen-Unsworth LC. 2015. A framework for the resilience of seagrass ecosystems. Marine Pollution Bulletin 100 (1):34-46. https://doi.org/10.1016/j.marpolbul.2015.08.016.

Valiela I, McClelland J, Hauxwell J, Behr PJ, Hersh D, Foreman K. 1997. Macroalgal blooms in shallow estuaries: Controls and ecophysiological and ecosystem consequences. In: Part 2. The Ecology and Oceanography of Harmful Algal Blooms. Limnology and Oceanography 42(5):1105-18.

Zimmerman RC, Kohrs DC, Steller DL, Alberte RS. 1997. Impacts of CO_2 enrichment on productivity and light requirements of eelgrass. Plant Physiology 115(2):599-607.

Riverine Rocky Bottom

System: Riverine

Subsystem: Tidal & Non-tidal

Class: Rocky Bottom

Sub-class: Bedrock, Rubble, Cobble, Gravel

Geographic Area: Entire Area

Overall Vulnerability Rank = Low

Habitat Sensitivity = Low

Climate Exposure = High

	**Riverine Rocky Bottom**	Attribute Mean	Data Quality	Distribution of Expert Scores
Sensitivity Attributes	Habitat condition	2.3	2.5	
	Habitat fragmentation	3.2	2.8	
	Distribution/Range	1.2	2	
	Mobility/Ability to spread or disperse	2.3	1.6	
	Resistance	1.6	2.6	
	Resilience	1.5	2	
	Sensitivity to changes in abiotic factors	1.7	2.2	
	Sensitivity and intensity of non−climate stressors	2.4	1.8	
	Dependency on critical ecological linkages	1.6	1.8	
	Sensitivity Component Score	Low		
Exposure Factors	Sea surface temp	n/a	n/a	
	Bottom temp	n/a	n/a	
	Air temp	n/a	n/a	
	River temp	3.2	2.2	
	Surface salinity	n/a	n/a	
	Bottom salinity	n/a	n/a	
	pH	n/a	n/a	
	Sea level rise	2.1	2.2	
	Precipitation	n/a	n/a	
	Floods	3	1.9	
	Droughts	2.9	1.9	
	Exposure Component Score	High		
**Overall Vulnerability Rank**		**Low**		

Low

Moderate

High

Very High

Habitat Name: Riverine Rocky Bottom
System: Riverine
Sub-system: Tidal and Non-tidal
Class: Rocky Bottom
Sub-class: Bedrock, Rubble, Cobble, Gravel
Geographic Area: Entire Area

Habitat Description: This sub-class includes riverine rocky bottom and banks, consisting of habitat in tidal and non-tidal rivers dominated by bedrock, boulders, cobbles, and gravel (i.e., median bed particle size greater than 2 mm). This habitat also includes the epibenthic flora and fauna associated with these hard bottoms, including periphytic biofilms and macroinvertebrate communities, but excludes specific habitats (i.e., macrophytic algal beds, rooted vascular, emergent wetlands) that are included in other sub-classes. Finer sediment smaller than 2 mm (i.e., sands and mud) may be present in this habitat but are typically embedded around coarser dominant rocky bed particles and/or protected by an armored coarse bed layer. This habitat also includes large woody debris, boulders, tree roots, trailing riparian vegetation, and other structural elements that may be present in otherwise rocky streambed/bank habitat. Riverine rocky shores can also support plant and animal communities, including lichens and mosses.

Riverine rocky streambed and bank habitats commonly occur throughout the Northeast U.S, with less prevalence in the Coastal Plain region (i.e., Mid-Atlantic region). Bedrock is most common, although not exclusive to, high-mountain or glaciated areas; boulders and cobbles are common in mountainous or high-gradient areas; cobbles and gravel (particle size less than 63 mm but greater than 2 mm) occurs in riffle areas or channels of many middle-gradient rivers and streams throughout the region.

Overall Climate Vulnerability Rank: Low (59% certainty from bootstrap analysis). Bootstrap analysis found that it is 41% likely that the overall climate vulnerability rank for this habitat is moderate, indicating a borderline rank.

Climate Exposure: High. Three exposure factors contributed to the High exposure score: River Temperature (4.0), Floods (3.0) and Droughts (2.9). Projected changes in river temperature are high throughout the Northeast, but highest in the very southern part of the range (Virginia and North Carolina), followed by the very northern part (northern and coastal Maine). Floods and droughts are projected to increase in magnitude and frequency in the Mid-Atlantic. In New England, floods are expected to increase in frequency but decrease in magnitude, and droughts are expected to increase inmagnitude but decrease in frequency.

Habitat Sensitivity: Low. One sensitivity attribute scored ≥3.0: Habitat Fragmentation (3.2), largely due to the prevalence of dams in rivers in the Northeast U.S. Three sensitivity attributes scored between 2.0 and 3.0: Sensitivity and Intensity of Non-Climate Stressors (2.4), Mobility/Ability to Spread or Disperse (2.3), and Habitat Condition (2.3). Scores were distributed across all scoring bins for the Mobility/Ability to Spread or Disperse attribute, suggesting differing interpretations on the ability of this habitat to spread or disperse. While the abiotic component of this habitat (e.g., boulders) generally do not move, the biotic component (e.g., infauna/epifauna and epiflora) associated with the habitat are capable of moving and dispersing. On the other hand, all rocky bottom substrates can be buried by fine sediments eroded from streambed and banks due to riparian disturbance and poor riparian cover.

Data Quality & Gaps: All four of the climate exposure mean scores were in the Low to Moderate range: River Temperature and Sea Level Rise (SLR) (both 2.2) and Floods and Droughts (both 1.9). Because of the fine-scale nature of riverine habitats and the coarse scale of this assessment, text descriptions of the spatial distribution of rocky streambed and bank were used for the climate exposure scoring rather than spatially-explicit maps of habitat distribution. This may have limited the ability of scorers to ascertain the overlap between the distribution of the habitat and climate exposure projections. Although the SLR projections are relatively consistent across the study area, this climate factor primarily only affects tidal influenced rivers and not non-tidal rivers. In addition, the considerable geographic variability of floods and droughts throughout the study area likely contributed to uncertainty in the habitat's climate exposure over the study area.

Four of the nine sensitivity attribute data quality scores were ≤2.0: Mobility/Ability to Spread or Disperse (1.6), Sensitivity and Intensity of Non-Climate Stressors (1.8), Dependency on Critical Ecological Linkages (1.8), Distribution/Range (2.0), and Resilience (2.0).

The independent scoring of the riverine streambed and bank habitat separately from the water column habitat may overlook some key interdependence between these habitats and the linked response of bed epiflora and infauna/epifauna to degraded water column conditions. While stream temperature and other abiotic components of the riverine streambed and bank habitat were considered in the sensitivity attribute scoring, scoring water column as a separate habitat may have diminished some of the sensitivity of biota in the rocky bottom sub-class. Temperature in the water column can control local temperature in gravel beds, limiting habitat suitability of these gravels (Acornley et al. 1999). In addition, bed epiflora and epifauna attached to bed substrates are known to respond to degraded water column conditions (Kenney et al. 2009).

Although rocky streambed and bank habitats are common in higher gradient headwaters, their occurrence can also span the river continuum and be present in large rivers, such as bedrock sections of the Potomac River. Grouping bedrock, cobble, rubble, and gravel together may overlook individual differences in vulnerability by size in this category.

The climate sensitivity of this habitat was scored without separating rivers in the study area into different temperature regimes, although there is noted spatial variability in cold-, cool-, and warm-water rivers across the study area, with broad patterns largely corresponding to latitude and elevation (McManamay et al. 2018). This may overlook individual differences by natural thermal regime, with cold- and cool-water rocky-bottom river systems, in contrast to rocky bottom habitats in warm waters, being particularly vulnerable to thermal changes with climate change in the study area (Eaton and Sheller 1996; DeWeber and Wagner 2018).

Positive or Negative Climate Effect in the Northeast U.S.: The effect of climate change on riverine rocky streambed and bank habitat is expected to be negative (70% of the experts' scores were negative, 30% were neutral).

Climate Effects on Habitat Condition and Distribution: Higher air temperature associated with climate change is expected to increase water temperature in streams and rivers with rocky bottom habitats, particularly in higher gradient areas where rocky bottom habitats tend to dominate.

Cold- and cool-water rocky-bottom river systems, a unique sub-class of riverine habitats determined by thermal suitability for cool- or cold-water dependent species, have been shown to be particularly vulnerable to climate-driven thermal changes in the Northeast USA (Eaton and Sheller 1996; Heino et al. 2009; DeWeber and Wagner 2018). Increased air temperatures are likely to drive water temperatures above thermally suitable ranges to be classified as coldwater habitat across broad regions in the Northeast (DeWeber and Wagner 2018). While common invertebrate taxa may persist in these conditions, a study in Europe has demonstrated that increases in headwater stream temperature could remove rare, cold-water taxa and reduce overall springtime abundance of macroinvertebrates (Durance and Ormerod 2007). Analyses in other regions suggest warming might disproportionately affect epifaunal detrital shredders and algal grazers, with implications to food web structure and energy flow to higher consumers in these habitats (Boyero et al. 2012; Pyne and Poff 2016). Local zones of groundwater intrusion may provide important thermal refugia to increasing temperatures, an importantfactor that otherwise may not be well captured by regional modeling approaches (Snyder et al. 2015).

Temperature is also a key regulator of dissolved oxygen, and climate-induced changes to temperature are expected to lower dissolved oxygen available in these habitats. Reductions in dissolved oxygen, especially during climate-driven reductions in flow, may particularly threaten current-dependent, high-oxygen-requirement epifauna taxa (e.g. rheophilic taxa) in these habitats (Poff et al. 2002). However, turbulent streamflow in higher gradient rivers, as well as relatively lower sediment organic matter in these habitats - compared to mud habitats - may help mitigate, although not eliminate, climate-driven reductions in instream

dissolved oxygen (Ficklin et al. 2013).

The natural flow regime, including floods and droughts, is a major driver of habitat condition and biodiversity, with aquatic species adapted to the natural flow regime in their natural habitat (Poff et al. 1997; Bunn and Arthington 2002). Therefore, climate-driven changes to the magnitude and frequency of droughts and floods are likely to affect habitat and biologic communities across the region. Increased frequency and/or magnitude of drought can reduce river flows, velocity, and turbulence, further magnifying reductions in dissolved oxygen and increasing water temperatures in pool habitats. Drought might have particular effect in small headwaters, especially for ephemeral or intermittent streams, which can have downstream impacts as these habitats go dry (Brooks 2009). In addition, temperature changes may likely affect seasonality in the hydrologic regime, particularly in areas with greater yearly snow accumulations, resulting in earlier snowmelt, and drive flow regime patterns to more closely resemble rain-driven watersheds.

There existed differing perspectives among scorers regarding the importance of abiotic vs. biotic components in scoring the resilience and resistance of riverine habitats due to climate change-driven changes in flooding. Abiotic rock substrate itself is relatively resistant and resilient to disturbance, as mobilized rocky-bottom substrate can continue to function as rocky-bottom habitat downriver. In contrast, while the biotic communities in rocky-bottom habitats can rapidly attain pre-disturbance diversity after intermittent floods (Lake 2000), persistent increases in flow magnitude has been shown to drive declines in macroinvertebrate diversity and abundances (Poff and Zimmerman 2010). Fewer studies have more directly studied the effect of climate-change driven increases in flood frequency in isolation, although repeated high-flow disturbance in the context of hydropower can cause behavior abandonment of the repeatedly disturbed rocky bottom habitats (Bruno et al. 2015).

Increasing precipitation intensity and overland flows can cause excess upland erosion from the upland landscape and stream banks and other in-channel sources, resulting in sedimentation over rocky bottom habitats, effectively smothering rocky-bottom habitats with sands and muds (Noe et al. 2020). A recent study in the Chesapeake Bay region of the Northeast has predicted degradation of smaller (<200 km^2 drainage) river habitat due to climate change through a regional benthic invertebrate index and predicted losses in habitat conditions across the region (Maloney et al. 2018). Model outputs suggested that increasing precipitation may mitigate the effects of increasing temperature in the future. However, Maloney et al. (2018) only examined total seasonal precipitation as a driver and did not evaluate changes associated with changes to individual storm magnitude or intensities. The study did not separate predicted impacts by habitat substrate, thermal classification, or for specific taxonomic groupings, but only a metric score. As a result, the grouped scores in this assessment of rocky bottom habitats across all thermal subclasses may underestimate the persistence and vulnerability specifically ofcold- and cool-water habitats or particularly sensitive taxa. Additional exploration of climate change impacts in the Northeast U.S. region should include responses by various riverine infauna/epifauna and epiflora communities, and associated functional traits that may indicate thermal or climate sensitivity or tolerances.

Habitat Summary: Current habitat conditions were recognized to be variable across the study region, largely due to differences in anthropogenic pressures between the Mid-Atlantic and New England, with more degraded conditions in populated regions of the Mid-Atlantic, and healthier conditions across the more expansive forested regions of New England. According to Crawford et al. (2016), most rivers in the Mid-Atlantic had low to moderate degraded habitat conditions, with some very highly degraded conditions corresponding to high population zones along the I-95 corridor. Estimated habitat conditions varied in New York and New England, with generally low or very low habitat degradation for most rivers,but a sizable number of rivers with very highly degraded conditions, particularly in Northern and Central New York, and in select regions in southern Vermont, New Hampshire, and Maine. Despite many rivers with low habitat degradation, Crawford et al. (2016) considered New York and New England to be one of the most threatened regions of the country. In New England, the most common disturbances were urban development, roads, and pastureland, although the highest risk areas are generally degradation by roads, urban and suburban sprawl, including point source effluent, agricultural land use, and mine impacts. In the Mid-Atlantic, most common disturbances were due to development and dams, while urban sprawl, agriculture, and roads were the largest

drivers in very highly degraded river habitats (Crawford et al. 2016).

A regional assessment on the EPA's National Aquatic Resource Surveys also identified similar patterns of degraded river condition, with degraded conditions in the Southern Appalachian Piedmont of the Mid-Atlantic and highly degraded conditions in the Northern Appalachian Piedmont regions of New York and New England, including the Boston Metro Area, Central Connecticut and Massachusetts, and New York's Hudson Valley (Hill et al. 2017). In the Northern Appalachians, top variables affecting river condition included several dam and reservoir metrics, population density, housing density, and temperature. In the Southern Appalachians, top drivers were temperature, agriculture on steep slopes, percent impervious, and watershed area (Hill et al. 2017). More local regional modeling in the Chesapeake Region in smaller streams (<200 km^2) highlighted degraded stream condition in the urbanized and agricultural core of Virginia, Maryland, and Pennsylvania, with most important predictors of condition being upstream impervious cover, upstream tree canopy cover, and elevation (Maloney et al. 2018).

Riverine habitats in the region are severely fragmented as a result of dam construction (Graf 1999) and installation of poorly designed culverts (Martin and Apse 2011). The Northeastern U.S.has the highest density of dams and road crossings in the country, with an average of 7 dams and 106 road-stream crossings per 100 miles of river (Anderson and Olivero Sheldon 2011). Dams can degrade rocky streambed habitat by trapping sediment, and burying the rocky streambed behind the dam. Dams can also reduce the frequency of natural hydrologic disturbances due to high flows, reducing frequency of scouring flows and reducing the diversity and condition of riverine streambed/bank habitats, as well as hydroelectric dams increasing downstream flow disturbance due to frequent, regular releases (i.e.,"hydropeaking").

Scorers differed in interpretations on the mobility/ability of this habitat to spread or disperse, represented by the wide range in individual scores; rocky bottom habitat may not be completely removed but can be buried by eroded fine sediments, effectively converting rocky-bottomed habitats in some locations to sand- or mud-bottom habitats. As noted in the 2008-2009 National Rivers and Streams Assessment, rivers in the Northern Appalachian regions are highly impacted by excess streambed fine-sediments, riparian disturbance, poor riparian vegetative cover, and poor in-stream fish habitat (EPA 2016). Excess streambed fine sediments can become embedded in the habitat spaces between cobbles and gravels (Wharton et al. 2017) or smother coarse, rocky bottom habitat, limiting habitat availability for insects and spawning areas for lithophilic fish (Kemp et al. 2011; Jones et al. 2012). Loss of riparian vegetative cover can lead to increased streambank erosion and sediment runoff. Riparian disturbance, or human activities adjacent to the river, include impervious surfaces, agriculture, dams, and logging (EPA 2016).

Literature Cited

Acornley RM. 1999. Water temperatures within spawning beds in two chalk streams and implications for salmonid egg development. Hydrological Processes 13(3):439-46.

Anderson MG, Olivero Sheldon A. 2011. Conservation status of fish, wildlife, and natural habitats in the northeast landscape: implementation of the Northeast Monitoring Framework. The Nature Conservancy, Eastern Conservation Science. 289 pp.

Boyero L, Pearson RG, Dudgeon D, Ferreira V, Graça MA, Gessner MO, Boulton AJ, Chauvet E, Yule CM, Albariño RJ, Ramírez A. 2012. Global patterns of stream detritivore distribution: implications for biodiversity loss in changing climates. Global Ecology and Biogeography 21(2):134-41.

Brooks RT. 2009. Potential impacts of global climate change on the hydrology and ecology of ephemeral freshwater systems of the forests of the northeastern United States. Climatic Change 95:469-83. https://doi.org/10.1007/s10584-008-9531-9

Bruno MC, Cashman MJ, Maiolini B, Biffi S, Zolezzi G. 2015. Responses of benthic invertebrates to repeated

hydropeaking in semi-natural flume simulations. Ecohydrology 9:68–82. 10.1002/eco.1611.

Bunn SE, Arthington AH. 2002. Basic principles and ecological consequences of altered flow regimes for aquatic biodiversity. Environmental Management 30(4):492-507.

Crawford S, Whelan G, Infante DM, Blackhart K, Daniel W, Fuller PL, Birdsong TW, Wieferich D, McClees-Funinan R, Stedman S, Herreman K. 2016. Through a fish's eye: the status of fish habitats in the United States 2015. National Fish Habitat Partnership. https://pubs.er.usgs.gov/publication/70200345

DeWeber JT, Wagner T. 2018. Probabilistic measures of climate change vulnerability, adaptation action benefits, and related uncertainty from maximum temperature metric selection. Global Change Biology 24:2735–48. https://doi.org/10.1111/gcb.14101

Durance I, Ormerod S.J. 2007. Climate change effects on upland stream macroinvertebrates over a 25-year period. Global Change Biology 13(5):942-57.

Eaton JG, Scheller RM. 1996. Effects of climate warming on fish thermal habitat in streams of the United States. Limnology and Oceanography 41(5):1109-15.

Ficklin D L, Stewart IT, Maurer EP. 2013. Effects of climate change on stream temperature, dissolved oxygen, and sediment concentration in the Sierra Nevada in California, Water Resources Research 49:2765-82. doi:10.1002/wrcr.20248.

Graf WL. 1999. Dam nation: a geographic census of American dams and their large-scale hydrologic impacts. Water Resources Research 35(4):1305-11.

Heino J, Virkkala R, Toivonen H. 2009. Climate change and freshwater biodiversity: detected patterns, future trends and adaptations in northern regions. Biological Reviews 84:39-54. doi:10.1111/j.1469-185X.2008.00060.x

Hill RA, Fox EW, Leibowitz SG, Olsen AR, Thornbrugh DJ, Weber MH. 2017. Predictive mapping of the biotic condition of conterminous US rivers and streams. Ecological Applications 27(8):2397-415.

Jones JI, Murphy JF, Collins AL, Sear DA, Naden PS, Armitage PD. 2012. The impact of fine sediment on macro-invertebrates. River Research and Applications 28(8):1055-71.

Kemp P, Sear D, Collins A, Naden P, Jones I. 2011. The impacts of fine sediment on riverine fish. Hydrological Processes 25(11):1800-21.

Kenney M, Sutton-Grier A, Smith RF, Gresens S. 2009. Benthic macroinvertebrates as indicators of water quality: the intersection of science and policy. Terrestrial Arthropod Reviews 2(2):99-128.

Lake PS. 2000. Disturbance, patchiness, and diversity in streams. Journal of the North American Benthological Society 19(4):573-92. https://doi.org/10.2307/1468118

Maloney KO, Smith ZM, Buchanan C, Nagel A, Young JA. 2018. Predicting biological conditions for small headwater streams in the Chesapeake Bay watershed. Freshwater Science 37(4):795-809.

Martin EH, Apse CD. 2011. [Internet]. Northeast aquatic connectivity: an assessment of dams on northeastern Rivers. The Nature Conservancy, Eastern Freshwater Program. https://rcngrants.org/sites/default/files/final_reports/NEAquaticConnectivity_Report.pdf. Accessed on 9/30/19.

McManamay RA, Troia MJ, DeRolph CR, Olivero Sheldon A, Barnett AR, Kao S-C, Anderson MG. 2018. A stream classification system to explore the physical habitat diversity and anthropogenic impacts in riverscapes of the eastern United States. PLOS ONE 13(6): e0198439. https://doi.org/10.1371/journal.pone.0198439

Noe GB, Cashman MJ, Skalak K, Gellis A, Hopkins KG, Moyer D, Webber J, Benthem A, Maloney K, Brakebill J, Sekellick A, Langland M, Zhang Q, Shenk G, Keisman J, Hupp C. 2020. Sediment dynamics and implications for management: state of the science from long-term research in the Chesapeake Bay watershed, USA. WIREs Water 7:e1454. https://doi.org/10.1002/wat2.1454

Poff NL, Allan JD, Bain MB, Karr JR, Prestegaard KL, Richter BD, Sparks RE, Stromberg JC. 1997. The natural flow regime. BioScience 47(11):769-84.

Poff NL, Brinson MM, Day JW. 2002. Aquatic ecosystems and global climate change: potential impacts on inland freshwater and coastal wetland ecosystems in the United States. Pew Center on Global Climate Change.

Poff NL, Zimmerman JKH. 2010. Ecological responses to altered flow regimes: a literature review to inform the science and management of environmental flows. Freshwater Biology 55:194-205.doi:10.1111/j.1365-2427.2009.02272.x

Pyne MI, Poff NL. 2017. Vulnerability of stream community composition and function to projected thermal warming and hydrologic change across ecoregions in the western United States. Global Change Biology 23(1):77-93.

Snyder CD, Hitt NP, Young JA. 2015. Accounting for groundwater in stream fish thermal habitat responses to climate change. Ecological Applications 25:1397-419. doi:10.1890/14-1354.1

U.S. Environmental Protection Agency (EPA). 2016. National Rivers and Streams Assessment 2008-2009: A Collaborative Survey (EPA/841/R-16/007). Office of Water and Office of Research and Development. Washington, DC. March 2016. https://www.epa.gov/sites/production/files/2016-03/documents/nrsa_0809_march_2_final.pdf.

Wharton G, Mohajeri SH, Righetti M. 2017. The pernicious problem of streambed colmation: a multi-disciplinary reflection on the mechanisms, causes, impacts, and management challenges.Wiley Interdisciplinary Reviews: Water 4(5):e1231.

Riverine Mud Bottom

System: Riverine

Subsystem: Tidal & Non-tidal

Class: Unconsolidated Bottom

Sub-class: Mud

Geographic Area: Entire Area

Overall Vulnerability Rank = Moderate

Habitat Sensitivity = Moderate

Climate Exposure = High

	Riverine Mud Bottom	Attribute Mean	Data Quality	Distribution of Expert Scores
Sensitivity Attributes	Habitat condition	2.4	2.1	
	Habitat fragmentation	2.8	2.6	
	Distribution/Range	1.2	1.6	
	Mobility/Ability to spread or disperse	1.8	1.6	
	Resistance	2.1	1.6	
	Resilience	1.6	1.6	
	Sensitivity to changes in abiotic factors	2.1	2	
	Sensitivity and intensity of non−climate stressors	2.9	1.8	
	Dependency on critical ecological linkages	1.3	1.8	
	Sensitivity Component Score	Moderate		
Exposure Factors	Sea surface temp	n/a	n/a	
	Bottom temp	n/a	n/a	
	Air temp	n/a	n/a	
	River temp	3.2	2.2	
	Surface salinity	n/a	n/a	
	Bottom salinity	n/a	n/a	
	pH	n/a	n/a	
	Sea level rise	2.1	2.2	
	Precipitation	n/a	n/a	
	Floods	3	1.9	
	Droughts	2.9	1.9	
	Exposure Component Score	High		
**Overall Vulnerability Rank**		**Moderate**		

Low
Moderate
High
Very High

Habitat Name: Riverine Mud Bottom

System: Riverine

Subsystem: Tidal and Non-tidal

Class: Unconsolidated Bottom

Sub-class: Mud

Geographic Area: Entire Area

Habitat Description: This sub-class includes riverine mud habitat, consisting of streambeds and banks of tidal and non-tidal rivers dominated by muds (i.e. silts and clays; median grain size less than 63 μm). This includes the epifauna/infauna (e.g., Chironomidae) and other remaining epiflora (e.g. microphytic biofilms) associated with these soft bottoms, but excludes specific habitats (i.e., macrophytic algal beds, rooted vascular, emergent wetlands) that are included in other subclasses. This sub-class also includes large woody debris, tree roots, trailing riparian vegetation, and other structural elements that occur in these habitats. Generally, this habitat is characterized by substrates lacking vegetation, except for pioneering plants during brief favorable periods.

Riverine mud streambed and bank habitats (silt/clay) remain naturally across the range of the study area, often interspersed with sand streambed, and can be found in greatest spatial extent patches in low-gradient sections of larger tidal freshwater rivers. Mud-dominated tills can also be found in NewYork and Western Massachusetts.

Overall Climate Vulnerability Rank: Moderate (96% certainty from bootstrap analysis).

Climate Exposure: High. Three exposure factors contributed to the High exposure score: River Temperature (4.0), Floods (3.0) and Droughts (2.9). Projected changes in river temperature are high throughout the Northeast U.S., but highest in the very southern part of the range (Virginia and North Carolina), followed by the very northern part (northern and coastal Maine). Floods and droughts are projected to increase in magnitude and frequency in the Mid-Atlantic. In New England, floods are expected to increase in frequency but decrease in magnitude, and droughts are expected to increase in magnitude but decrease in frequency.

Habitat Sensitivity: Moderate. No sensitivity attributes scored ≥3.0. The highest sensitivity scores were for Sensitivity and Intensity of Non-Climate Stressors (2.9), Habitat Fragmentation (2.8), and Habitat Condition (2.4).

Data Quality & Gaps: All four of the climate exposure mean scores were in the Low to Moderate range: River Temperature and Sea Level Rise (SLR) (both 2.2) and Floods and Droughts (both 1.9). Because of the fine-scale nature of riverine habitats and the coarse scale of this assessment, text descriptions of the spatial distribution of sand streambed and bank were used for the climate exposure scoring. This mayhave limited the ability of scorers to ascertain the overlap between the distribution of the habitat and climate exposure projections. Although the SLR projections are relatively consistent across thestudy area, this climate factor primarily only affects tidal influenced rivers and not non-tidal rivers. In addition, the considerable geographic variability of floods and droughts throughout the study area likely contributed to uncertainty in the habitat's climate exposure over the study area.

Seven of the nine sensitivity attribute data quality scores were ≤2.0. The data quality scores greater than 2.0 were Habitat Fragmentation (2.6) and Habitat Condition (2.1).

The independent scoring of bed habitat vulnerability in isolation from the water column may overlook some key interdependence in condition between these habitats and linked response of bed epiflora and in-fauna/epifauna to degraded water column conditions (Kenney et al. 2009). Water temperature may impact the function of mud habitats and nutrient-rich organic muds can, depending on redox conditions, sorb or desorb nutrients into the water column, which are then taken up by organisms or transported downriver (Newbold et

al. 1983; Froelich et al. 1988).

Although mud riverbed and bank habitats are common in low gradient locations, their occurrence can also span the river continuum, from marginal areas in smaller headwater habitats, accumulations behind woody debris in any river size, rivers in inland agricultural valleys, to depositional areas in lower-gradient larger rivers. Grouped scoring may overlook individual differences in vulnerability by size and type in this category.

Positive or Negative Climate Effect in the Northeast U.S.: Expert scores for the direction of the effect of climate change on riverine mud streambed and bank were split: 45% of the experts' scores were positive, 35% were neutral, and 20% were negative. This divergence may be partially because, while mud habitats will be impacted by some aspects of climate change, increased erosion from intense precipitation events may cause mud habitats to replace rocky or sandy habitats in some locations (see climate effects below for more details).

Climate Effects on Habitat Condition and Distribution: Higher air temperature associated with climate change is expected to increase water temperature in streams and rivers with mud bottom habitats, particularly in lower gradient areas with slower flows. Many of these rivers typically have warmer thermal regimes, compared to cold- or cool-water rocky headwater streams, and epifloral, epifauna, and infauna in these habitats are adapted to warmer water temperature conditions. However, increased water temperature still can increase respiration rates, and for increases above tolerable thresholds, can cause stress to these communities (Galbraith et al. 2012). Furthermore, increased water temperature decreases oxygen solubility and climate-induced changes to temperature are expected to lower dissolved oxygen available in these habitats. The high organic content, and biological oxygen demand, ofmud sediments can typically result in seasonal hypoxia, and increased temperatures may exacerbate the magnitude or frequency of typical seasonal hypoxia. Many taxa in mud-bottom habitats are adapted to lower or anoxic oxygen conditions that often occur in these habitats, including Chironomidae, Oligochaeta, and other taxa, and due to physiological adaptations have been shown the potential to maintain diverse and abundant communities during hypoxic conditions (Kornijów et al. 2010). However, the degree of taxa resilience – or compositional shifts – to climate-driven temperatures and hypoxic conditions may depend on the relative scale of change compared to historical conditions and currently unknown thresholds. More research is needed into potential thresholds and the impacts of increasing compositional shifts to tolerant taxa would affect ecosystem processes.

The natural flow regime, including floods and droughts, is a major driver of habitat condition and biodiversity, with aquatic species adapted to the natural flow regime in their natural habitat (Poff et al. 1997; Bunn and Arthington 2002). Therefore, climate-driven changes to the magnitude and frequency ofdroughts and floods are likely to affect habitat and biologic communities across the region. Increased frequency and/or magnitude of drought can reduce river flows, velocity, and turbulence, further magnifying reductions in dissolved oxygen and increasing water temperatures in and around mud-bottom habitat. Drought may also lead to mud-bottom habitats on river margins going dry with dropping water levels.

There existed differing perspectives among scorers regarding the vulnerability of abiotic versus biotic components of mud-habitats to flow disturbance. Abiotic (silt-dominant) mud substrate itself is easily moved by river flows, and thus was scored vulnerable to disturbance, although greater proportions of clay in mud substrate can result in cohesive properties which results in greater resistance to high-flow disturbance. In contrast, invertebrate communities are typically adapted to the typical disturbance rate of these bed conditions. Habitats can rapidly attain pre-disturbance diversity after intermittent floods (Lake 2000), yet generally persistent increases in flow magnitudes can consistently drive declines in macroinvertebrate diversity and abundances (Poff and Zimmerman 2010). Fewer studies have more directly studied the effect of climate-change driven increases in flood frequency in isolation, yet more persistent low flows due to river regulation have been shown to have consistent negative effects on downstream communities (Poff et al. 1997; Bunn and Arthington 2002).

Excess flows, erosion, and sedimentation from the landscape, driven by greater surface erosion under more intense precipitation events can result in increased fine sediment loads, resulting in silts and clays burying

rocky bottom or sand habitats, resulting in a possible positive impact on spatial distribution of mud habitats from climate change.

A recent study in the Chesapeake Bay region of the Northeast U.S. has examined change in stream condition due to climate change through a regional benthic invertebrate index (Maloney et al. 2020). This study predicted improvements in habitat conditions due to climate change in the small streams in the Coastal Plain, where rivers are typically soft bottom. However, model outputs suggested that increasing precipitation may mitigate the effects of increasing temperature in the future. However, Maloney et al. (2020) only examined total seasonal precipitation as a driver and did not evaluate changes associated with changes to individual storm magnitude or intensities.

Additional exploration of changes in habitat condition in the Northeast U.S. region should examine responses by various components of the epifauna, infauna, and epiflora communities, rather than just overall community metrics. Additional research into functional traits that may indicate thermal or climate sensitivity or tolerances of various taxa, and better explain expected changes with climate change in the Northeast U.S.

Habitat Summary: Current habitat conditions were recognized to be variable across the study region, largely due to differences in anthropogenic pressures between the Mid-Atlantic and New England, with more degraded conditions in populated regions of the Mid-Atlantic, and healthier conditions across the more expansive forested regions of New England. According to Crawford et al. (2016), most rivers in the Mid-Atlantic had low to moderate degraded habitat conditions, with some very highly degraded conditions corresponding to high population zones along the I-95 corridor. Estimated habitat conditions were more variable in New York and New England, with generally low or very low habitat degradation for most rivers, but also large numbers of very highly degraded rivers in Northern, Central, and Western New York, and in select regions in southern Vermont, New Hampshire, and Maine. Degraded conditions also exist for many large rivers across both regions. Despite many rivers with low habitat degradation, Crawford et al. (2016) considered New York and New England to be one of the most threatened regions of the country. In New England, most common disturbances were urban development, roads, and pastureland, although the highest risk streams are generally degradation by roads, urban and suburban sprawl, agricultural land use, and mines. In the Mid-Atlantic, most common disturbances were due to development and dams, while urban sprawl, agriculture, and roads were the largest drivers in very highly degraded river habitats (Crawford et al. 2016). Human uses of the landscape (e.g., agriculture, construction, and urbanization) cause riparian disturbance, increase the amount of fine sediments entering streams and rivers, and change the amount, timing, and intensity of water runoff into channels (EPA 2016).

A legacy of heavy metals and contaminants such as polychlorinated biphenyls (PCBs), polyaromatic hydrocarbons, and pesticides from urban and industrial activities persist in mud sediments across the region, with New England and the industrial Mid-Atlantic region having the highest levels of PCBs in streambed sediment in the U.S. (Chalmers 2002), including the Potomac River, the Delaware River and the Hudson River (Crawford et al. 2016). Heavy metals and other contaminants preferentially bind to smaller particle size mud sediments, particularly clays, resulting in high concentrations in many mud habitats. These can bioaccumulate through the food chain and present a risk to higher trophic levels. In addition, mud sediments can also bind high levels of phosphorous and other nutrient pollutants. These pollutants can also be released into the water column due to redox, pH, and microbial activity and fuel low water clarity and algal blooms (Noe et al. 2020).

Predictive modeling based on the EPA's National Aquatic Resource Surveys also identified similar patterns of degraded river condition, with degraded conditions particularly in the soft-bottom dominated Coastal Plain in the Delmarva Peninsula, New Jersey, and Long Island in New York. Predictions in the soft-bottom Coastal Plain were driven by density of stream-road crossings in sloped lands, density of ditches or canals, sand content in upland soils, agricultural land use, and fertilizer applications (Hill et al. 2017). More local regional modeling in the Chesapeake Region in smaller streams (<200 km^2) highlighted mixed stream condition in the Coastal Plain, with most important predictors of condition being upstream impervious cover, upstream tree canopy cover, and elevation (Maloney et al. 2018).

Riverine habitats in the region are severely fragmented as a result of dam construction (Graf 1999) and installation of poorly designed culverts (Martin and Apse 2011). The northeastern U.S. has the highest density of dams and road crossings in the country, with an average of 7 dams and 106 road-stream crossings per 100 miles of river (Anderson and Olivero Sheldon 2011). Dams prevent sediment from traveling downstream, starving downstream reaches of sediment supply, and leading to riverbed degradation (Brandt 2000). Where abundant sediment exists below dams, the reduction in sediment transport capacity caused by a dam can lead to excessive sediment deposition and narrowing or simplification of the river channel (Pitlick and Wilcock 2001). Dams can also reduce the frequency of hydrologic disturbance, reducing the diversity of riverine streambed/bank habitats.

Literature Cited

Anderson MG, Olivero Sheldon A. 2011. Conservation status of fish, wildlife, and natural habitats in the northeast landscape: implementation of the Northeast Monitoring Framework. The Nature Conservancy, Eastern Conservation Science. 289 pp.

Brandt SA. 2000. Classification of geomorphological effects downstream of dams. Catena 40(4):375-401.

Bunn SE, Arthington AH. 2002. Basic principles and ecological consequences of altered flow regimes for aquatic biodiversity. Environmental Management 30(4):492-507.

Chalmers A. 2002. Trace elements and organic compounds in streambed sediment and fish tissue of coastal New England streams, 1998-99. U.S. Geological Service, Water-Resources Investigations Report 02-4179. https://babel.hathitrust.org/cgi/pt?id=osu.32435085219152&view=1up&seq=3

Crawford S, Whelan G, Infante DM, Blackhart K, Daniel W, Fuller PL, Birdsong TW, Wieferich D, McClees-Funinan R, Stedman S, Herreman K. 2016. Through a fish's eye: the status of fish habitats in the United States 2015. National Fish Habitat Partnership. https://pubs.er.usgs.gov/publication/70200345

Froelich PN. 1988. Kinetic control of dissolved phosphate in natural rivers and estuaries: a primer on the phosphate buffer mechanism. Limnology and Oceanography 33. doi:10.4319/lo.1988.33.4part2.0649.

Galbraith HS, Blakeslee CJ, Lellis WA. 2012. Recent thermal history influences thermal tolerance in freshwater mussel species (Bivalvia: Unionoida). Freshwater Science 31(1):83-92.

Graf WL. 1999. Dam nation: a geographic census of American dams and their large-scale hydrologic impacts. Water Resources Research 35(4):1305-11.

Hill RA, Fox EW, Leibowitz SG, Olsen AR, Thornbrugh DJ, Weber MH. 2017. Predictive mapping of the biotic condition of conterminous US rivers and streams. Ecological Applications 27(8):2397-415.

Kenney M, Sutton-Grier A, Smith RF, Gresens S. 2009. Benthic macroinvertebrates as indicators of water quality: the intersection of science and policy. Terrestrial Arthropod Reviews 2(2):99-128.

Kornijów R, Strayer DL, Caraco NF. 2010. Macroinvertebrate communities of hypoxic habitats created by an invasive plant (*Trapa natans*) in the freshwater tidal Hudson River. Fundamental and Applied Limnology/Archiv für Hydrobiologie 176(3):199-207.

Lake PS. 2000. Disturbance, patchiness, and diversity in streams. Journal of the North American Benthological Society 19(4):573-92. https://doi.org/10.2307/1468118

Maloney KO, Smith ZM, Buchanan C, Nagel A, Young JA. 2018. Predicting biological conditions for small headwater streams in the Chesapeake Bay watershed. Freshwater Science 37(4):795-809.

Maloney KO, Krause KP, Buchanan C, Hay LE, McCabe GJ, Smith ZM, Sohl TL, Young JA. 2020. Disentangling the potential effects of land-use and climate change on stream conditions. Global Change Biology 26(4):2251-69.

Martin EH, Apse CD. 2011. [Internet]. Northeast aquatic connectivity: an assessment of dams on northeastern Rivers. The Nature Conservancy, Eastern Freshwater Program. (accessed 30 September 2019). https://rcngrants.org/sites/default/files/final_reports/NEAquaticConnectivity_Report.pdf

Newbold JD, Elwood JW, O'Neill RV, Sheldon AL. 1983. Phosphorus dynamics in a woodland stream ecosystem: a study of nutrient spiralling. Ecology 64(5):1249-65.

Noe GB, Cashman MJ, Skalak K, Gellis A, Hopkins KG, Moyer D, Webber J, Benthem A, Maloney K, Brakebill J, Sekellick A, Langland M, Zhang Q, Shenk G, Keisman J, Hupp C. 2020. Sediment dynamics and implications for management: state of the science from long-term research in the Chesapeake Bay watershed, USA. WIREs Water 7:e1454. https://doi.org/10.1002/wat2.1454

Pitlick J, Wilcock P. 2001. Relations between streamflow, sediment transport, and aquatic habitat in regulated rivers. Geomorphic Processes and Riverine Habitat, Water Science and Application 4. https://agupubs.onlinelibrary.wiley.com/doi/pdf/10.1029/WS004p0185.

Poff NL, Allan JD, Bain MB, Karr JR, Prestegaard KL, Richter BD, Sparks RE, Stromberg JC. 1997. The natural flow regime. BioScience 47(11):769-84.

Poff NL, Zimmerman JKH. 2010. Ecological responses to altered flow regimes: a literature review to inform the science and management of environmental flows. Freshwater Biology 55:194-205. doi:10.1111/j.1365-2427.2009.02272.x

U.S. Environmental Protection Agency (EPA). 2016. National Rivers and Streams Assessment 2008-2009:A Collaborative Survey (EPA/841/R-16/007). Office of Water and Office of Research and Development. Washington, DC. March 2016. https://www.epa.gov/sites/production/files/2016-03/documents/nrsa_0809_march_2_final.pdf

Riverine Sand Bottom

System: Riverine

Subsystem: Tidal & Non-tidal

Class: Unconsolidated Bottom

Sub-class: Sand

Geographic Area: Entire Area

Overall Vulnerability Rank = Moderate

Habitat Sensitivity = Moderate

Climate Exposure = High

	Riverine Sand Bottom	Attribute Mean	Data Quality	Distribution of Expert Scores
Sensitivity Attributes	Habitat condition	2.6	2.1	
	Habitat fragmentation	3.1	2.6	
	Distribution/Range	1.2	1.8	
	Mobility/Ability to spread or disperse	1.9	1.6	
	Resistance	2	1.8	
	Resilience	1.5	1.8	
	Sensitivity to changes in abiotic factors	1.6	2.2	
	Sensitivity and intensity of non−climate stressors	2.5	1.8	
	Dependency on critical ecological linkages	1.3	1.8	
	Sensitivity Component Score	Moderate		
Exposure Factors	Sea surface temp	n/a	n/a	
	Bottom temp	n/a	n/a	
	Air temp	n/a	n/a	
	River temp	3.2	2.2	
	Surface salinity	n/a	n/a	
	Bottom salinity	n/a	n/a	
	pH	n/a	n/a	
	Sea level rise	2.1	2.2	
	Precipitation	n/a	n/a	
	Floods	3	1.9	
	Droughts	2.9	1.9	
	Exposure Component Score	High		
**Overall Vulnerability Rank**		**Moderate**		

Low
Moderate
High
Very High

Habitat Name: Riverine Sand Bottom

System: Riverine

Subsystem: Tidal and Non-tidal

Class: Unconsolidated Bottom

Sub-class: Sand

Geographic Area: Entire Area

<u>Habitat Description</u>: This sub-class includes riverine sandy streambeds and banks, consisting of habitats in tidal and non-tidal rivers dominated by sand particles (i.e., median bed particle size smaller than 2 mm but greater than 63 µm). This includes the epifauna/infauna and epiflora associated with these habitats (e.g., freshwater mussels), but exclude specific habitats (i.e., algal beds, rooted vascular, emergent wetlands) that are included in other sub-classes. This habitat also includes large woody debris, tree roots, and other structural elements that occur in sand streambed/bank.

Riverine sand streambed and bank habitats are predominantly located in the Coastal Plain sections of the Mid-Atlantic (McManamay et al. 2018), as well as other rivers across the study area with low-gradients, sandy soils, and/or high sediment loads. Sand habitats can often be interspersed locally with mud streambeds, or in discrete patches in otherwise cobble-gravel streambeds.

<u>Overall Climate Vulnerability Rank</u>: Moderate (84% certainty from bootstrap analysis). Bootstrap analysis found a 15% probability that the overall vulnerability rank is Low.

<u>Climate Exposure</u>: High. Three exposure factors contributed to the High exposure score: River Temperature (4.0), Floods (3.0) and Droughts (2.9). Projected changes in River Temperature are high throughout the Northeast, but highest in the very southern part of the range (Virginia and North Carolina), followed by the very northern part (northern and coastal Maine). Floods and droughts are projected to increase in magnitude and frequency in the Mid-Atlantic. In New England, floods are expected to increase in frequency but decrease in magnitude, and droughts are expected to increase inmagnitude but decrease in frequency.

<u>Habitat Sensitivity</u>: Moderate. One sensitivity attribute scored >3.0: Habitat Fragmentation (3.1), largely due to the prevalence of dams in rivers in the Northeast U.S. Three sensitivity attributes scored between 2.0 and 3.0: Resistance (2.0), Sensitivity and Intensity of Non-Climate Stressors (2.5) and Habitat Condition (2.6). Two sensitivity attributes had scores across a wide range of bins: Mobility/Ability to Spread or Disperse and Resistance, which may reflect uncertainty in how these habitats will respond to various climate-related and non-climate perturbations and stressors.

<u>Data Quality & Gaps</u>: All four of the climate exposure mean scores were in the Low to Moderate range: River Temperature and Sea Level Rise (SLR) (both 2.2) and Floods and Droughts (both 1.9). Because of the fine-scale nature of riverine habitats and the coarse scale of this assessment, text descriptions of the spatial distribution of sand streambed and bank were used for the climate exposure scoring. This may have limited the ability of scorers to ascertain the overlap between the distribution of the habitat and climate exposure projections. Although the SLR projections are relatively consistent across the study area, this climate factor primarily only affects tidal influenced rivers and not non-tidal rivers. In addition, the considerable geographic variability of floods and droughts throughout the study area likelycontributed to uncertainty in the habitat's climate exposure over the study area.

Six of the nine sensitivity attribute data quality scores were ≤2.0. The data quality scores greater than 2.0 were Habitat Fragmentation (2.6), Sensitivity to Changes in Abiotic Factors (2.2), and Habitat Condition (2.1).

The independent scoring of sand habitat vulnerability in isolation from the water column may overlook some key interdependence in condition between these habitats and linked response of bed epiflora and epifauna to degraded water column conditions (Kenney et al. 2009). Particularly notable for sand bottom habitats, mussel taxa respond directly to water column conditions, filter feeding on drifting particles and responding to temperature and other water quality conditions (Strayer et al. 2004).

Although sandy riverbed and bank habitats are common in low gradient locations, their occurrence can also span the river continuum, from low-gradient headwaters in the Coastal Plain, medium rivers in inland agricultural valleys, to larger rivers near the coast. Scoring all of these sandy riverbed and bank habitats together overlook individual differences in vulnerability by size and gradient in this category. In addition, anthropogenic disturbance have resulted in substantial sediment deposition in river valleys and beds, and may have altered the spatial extent and degree of soft-bottomed habitat of rivers compared to pre-European colonization (Walter and Merritts 2008; Johnson et al. 2019).

Positive or Negative Climate Effect in the Northeast U.S.: Expert scores for the direction of the effect of climate change on riverine sand streambed and bank were split: 35% of the experts' scores were positive, 30% were neutral, and 35% were negative. This divergence in opinions was due to disagreement in the balance that while some sand habitats will be impacted by some aspects of climate change, increased erosion from intense precipitation events may cause sand habitats to replace rocky habitats in some locations (see climate effects below for more details).

Climate Effects on Habitat Condition and Distribution: Suitability of sand bottom habitats can be critically linked to the water column, such as for certain filter-feeding taxa such as freshwater mussels and filter-feeding macroinvertebrates, but within the sand habitat itself as surface water mixes with groundwater in a process known as hyporheic exchange (Klos et al. 2015). As a result, climate change issues associated with water column habitats would likely have compounding impacts on sand bottom habitats, even if those scores were not also incorporated in this habitat.

Higher air temperature associated with climate change is expected to increase water temperature in streams and rivers with sandy bottom habitats, particularly in lower gradient areas with slower flows. Many of these rivers typically have warmer thermal regimes, compared to cold- or cool-water rocky headwater streams, and epiflora and in-fauna/epifauna in these habitats are adapted to these naturally warmer water temperatures. However, increased water temperature can increase respiration rates, andfor increases above tolerable thresholds, can cause stress to these communities (Galbraith et al. 2012). Furthermore, increased water temperature decreases oxygen solubility and climate-induced changes to temperature are expected to lower dissolved oxygen available in these habitats. Generally, sandy sediments have greater pore space, lower sediment oxygen demand, and facilitate greater oxygen exchange than mud sediments, and are less susceptible to anoxic conditions than mud. However, while taxa in sand-bottom habitats are generally more adapted to lower oxygen requirements than some taxa in high-gradient rocky bottom habitats, such as rheophilic taxa (Poff et al. 2002), the extent of these impacts on sandy-bottom taxa will depend on the range of change compared to natural tolerances. Additional research is needed into the thermal tolerances and effects of increasing temperature and decreasing dissolved oxygen in these naturally warmer-water, soft-bottom rivers.

The natural flow regime, including floods and droughts, is a major driver of habitat condition and biodiversity, with aquatic species adapted to the natural flow regime in their natural habitat (Poff et al. 1997; Bunn and Arthington 2002). Therefore, climate-driven changes to the magnitude and frequency of droughts and floods are likely to affect habitat and biologic communities across the region. Increased frequency and/or magnitude of drought can reduce river flows, velocity, and turbulence, further magnifying reductions in dissolved oxygen and increasing water temperatures in pool or other low flow habitats. Drought will also likely lead to loss of river margin habitat as sand bottom habitats go dry with dropping water levels.

There existed some disagreement among scorers regarding the resilience and resistance of abiotic vs biotic components in riverine habitats due to climate change-driven changes in floods. Abiotic sand substrate itself is easily moved by river flows, and thus considered vulnerable to disturbance. In contrast, invertebrate communities are generally low in abundance in these habitats and prone to high turnover due to the instability of the substrate (Gibbins et al. 2010), but those communities that do persist are adapted to these bed conditions. Habitats can rapidly attain pre-disturbance diversity after intermittent floods (Lake 2000), yet generally persistent increases in flow magnitudes can consistently drive declines in macroinvertebrate

diversity and abundances (Poff and Zimmerman 2010). Fewerstudies have more directly studied the effect of climate-change driven increases in flood frequency in isolation, yet more persistent low flows due to river regulation have been shown to have consistent negative effects on downstream communities (Poff et al. 1997; Bunn and Arthington 2002).

Excess flows, erosion, and sedimentation from the landscape, driven by greater surface erosion under more intense precipitation events can cause rocky bottom habitats to turn into sand habitats, resulting in a positive impact from climate change on sand habitats.

A recent study in the Chesapeake Bay region of the Northeast has examined change in stream condition due to climate change through a regional benthic invertebrate index (Maloney et al. 2018). This study predicted improvements in habitat conditions in the small streams in the Coastal Plain, where rivers are typically soft-bottom, due to climate change. However, model outputs suggested that increasing precipitation may mitigate the effects of increasing temperature in the future. However, this study only examined total seasonal precipitation as a driver and did not evaluate changes associated with changes to individual storm magnitude or intensities.

Additional exploration of changes in habitat condition in the Northeast Region should examine responses by various components of the epifauna and epiflora communities, rather than just overall community metrics. Additional research into functional traits that may indicate thermal or climate sensitivity or tolerances of various taxa, and better explain expected changes with climate change in theNortheast.

Habitat Summary: Current habitat conditions were recognized to be variable across the study region, largely due to differences in anthropogenic pressures between the Mid-Atlantic and New England, with more degraded conditions in populated regions of the Mid-Atlantic, and healthier conditions across the more expansive forested regions of New England. According to Crawford et al. (2016), most rivers in the Mid-Atlantic had low to moderate degraded habitat conditions, with some very highly degraded conditions corresponding to high population zones along the I-95 corridor and Eerie Drift Plain in Northwest Pennsylvania. Estimated habitat conditions were more bimodal in New York and New England, with generally low or very low habitat degradation for most river areas, but the region also contained large regions of very highly degraded conditions in Northern and Central New York, and in select regions in southern Vermont, New Hampshire, and Maine. Despite many rivers with low habitat degradation, Crawford et al. (2016) considered New York and New England to be one of the most threatened regions of the country. In New England, most common disturbances were urban development, roads, and pastureland, although the highest risk streams are generally degradation by roads, urban and suburban sprawl, agricultural land use, and mines. In the Mid-Atlantic, most common disturbances were due to development and dams, while urban sprawl, agriculture, and roads were the largest drivers in very highly degraded river habitats (Crawford et al. 2016).

Predictive modeling based on the EPA's National Aquatic Resource Surveys also identified similar patterns of degraded river condition, with degraded conditions particularly in the soft-bottom dominated Coastal Plain in the Delmarva Peninsula, New Jersey, and Long Island in New York. Predictions in the soft-bottom Coastal Plain were driven by density of stream-road crossings in sloped lands, density of ditches or canals, sand content in upland soils, agricultural land use, and fertilizer applications (Hill et al. 2017). More local regional modeling in the Chesapeake Region in smaller streams (<200 km^2) highlighted mixed stream conditions in the Coastal Plain, with most important predictors of Chesapeake Bay-wide condition being upstream impervious cover, upstream tree canopy cover, and elevation (Maloney et al. 2018).

Furthermore, human uses of the landscape (e.g., agriculture, construction, and urbanization) cause riparian disturbance, increase the amount of fine sediments entering streams and rivers, and change the amount and timing of water runoff into channels (EPA 2016). This can shift streams from larger sediments to finer, more unstable particles, as sand sediments can be prone to erosion and transport.

Bottom sediments in many rivers are degraded from sediment contamination (e.g., nutrients, polychlorinated

biphenyls (PCBs), and heavy metals), with concentrations correlated with watershed population density. New England and the industrial Mid-Atlantic region have the highest levels of PCBs in streambed sediment in the U.S. (Chalmers 2002), threatening aquatic biota. Generally, however, sand habitats have reduced concentrations of contaminants in comparison to mud.

Riverine habitats in the region are severely fragmented as a result of dam construction (Graf 1999) and installation of poorly designed culverts (Martin and Apse 2011). The Northeastern U.S. has the highest density of dams and road crossings in the country, with an average of 7 dams and 106 road-stream crossings per 100 miles of river (Anderson and Olivero Sheldon 2011). Dams can lead to excessive sediment deposition and narrowing or simplification of the river channel (Pitlick and Wilcock 2001).

Dams can also reduce the frequency of flushing hydrologic disturbances, increase frequency of algal bloom occurrence, and reduce the diversity of riverine streambed/bank habitats. Although the Coastal Plain has lower dam densities than other areas in the region, rivers in the Coastal Plains ecoregion are widely channelized, confined by levees, and impacted by culverts at stream-crossings and ditching (Hill et al. 2017). Channelization can alter streambed elevation and increase erosion and sediment load (EPA 2016).

Scorers differed in interpretations on the mobility/ability of this habitat to spread or disperse, represented by the wide range in reports scores; sand habitats might expand in area, or transition into mud-bottom habitats, due to high amounts of upland erosion and local deposition. Alternatively, sand habitats might be lost in some locations with removal of dam impoundments and release of trapped impounded sandy sediments. As noted in the 2008-2009 National Rivers and Streams Assessment, rivers in the Northern Appalachian and Coastal Plains regions are highly impacted by excess streambed fine-sediments, riparian disturbance, poor riparian vegetative cover, and poor in-stream fish habitat (EPA 2016). Excess streambed fine sediments can become embedded in the habitat spaces between cobbles and gravels (Wharton et al. 2017) or completely cover rocky bottom habitat. Loss of riparian vegetative cover can lead to increased streambank erosion and sediment runoff. Riparian disturbance,or human activities adjacent to the river, include impervious surfaces, agriculture, dams, and logging (EPA 2016).

Floods and droughts are the major forms of natural disturbance in rivers. The effects of floods have been relatively well studied, often with rapid attainment of pre-disturbance levels of diversity at the local scale (Lake 2000). However, the effects of droughts are not as well understood (Lake 2000). Sand streambeds and banks and their associated invertebrates are adapted to physical disturbance (e.g., high flows, scour, etc.) but not disturbances attributed to anthropogenic effects (e.g., persistent sedimentation) or increasingly intense precipitation (Kemp 2011).

Literature Cited

Anderson MG, Olivero Sheldon A. 2011. Conservation status of fish, wildlife, and natural habitats in the northeast landscape: implementation of the Northeast Monitoring Framework. The Nature Conservancy, Eastern Conservation Science. 289 pp.

Bunn SE, Arthington AH. 2002. Basic principles and ecological consequences of altered flow regimes for aquatic biodiversity. Environmental Management 30(4):492-507.

Chalmers A. 2002. Trace elements and organic compounds in streambed sediment and fish tissue of coastal New England streams, 1998-99. U.S. Geological Service, Water-Resources Investigations Report 02-4179. https://babel.hathitrust.org/cgi/pt?id=osu.32435085219152&view=1up&seq=3

Crawford S, Whelan G, Infante DM, Blackhart K, Daniel W, Fuller PL, Birdsong TW, Wieferich D, McClees-Funinan R, Stedman S, Herreman K. 2016. Through a fish's eye: the status of fish habitats in the United States 2015. National Fish Habitat Partnership. https://pubs.er.usgs.gov/publication/70200345

Galbraith HS, Blakeslee CJ, Lellis WA. 2012. Recent thermal history influences thermal tolerance in freshwater mussel species (Bivalvia: Unionoida). Freshwater Science 31(1):83-92.

Gibbins CN, Vericat D, Batalla RJ. 2010. Relations between invertebrate drift and flow velocity in sand-bed and riffle habitats and the limits imposed by substrate stability and benthic density.Journal of the North American Benthological Society 29(3):945-58.

Graf WL. 1999. Dam nation: a geographic census of American dams and their large-scale hydrologic impacts. Water Resources Research 35(4):1305-11.

Hill RA, Fox EW, Leibowitz SG, Olsen AR, Thornbrugh DJ, Weber MH. 2017. Predictive mapping of the biotic condition of conterminous US rivers and streams. Ecological Applications 27(8):2397-415.

Johnson KM, Snyder NP, Castle S, Hopkins AJ, Waltner M, Merritts DJ, Walter RC. 2019. Legacy sediment storage in New England river valleys: anthropogenic processes in a postglacial landscape. Geomorphology 327:417-37.

Kemp P, Sear D, Collins A, Naden P, Jones I. 2011. The impacts of fine sediment on riverine fish. Hydrological Processes 25(11):1800-21.

Kenney M, Sutton-Grier A, Smith RF, Gresens S. 2009. Benthic macroinvertebrates as indicators of water quality: the intersection of science and policy. Terrestrial Arthropod Reviews 2(2):99-128.

Klos PZ, Rosenberry DO, Nelson GR. 2015. Influence of hyporheic exchange, substrate distribution, and other physically linked hydrogeomorphic characteristics on abundance of freshwater mussels. Ecohydrology 8(7):1284-91.

Lake PS. 2000. Disturbance, patchiness, and diversity in streams. Journal of the North American Benthological Society 19(4):573-92. https://doi.org/10.2307/1468118

Maloney KO, Smith ZM, Buchanan C, Nagel A, Young JA. 2018. Predicting biological conditions for small headwater streams in the Chesapeake Bay watershed. Freshwater Science 37(4):795-809.

Martin EH, Apse CD. 2011. [Internet]. Northeast aquatic connectivity: an assessment of dams on northeastern Rivers. The Nature Conservancy, Eastern Freshwater Program. (accessed 30 September 2019). https://rcngrants.org/sites/default/files/final_reports/NEAquaticConnectivity_Report.pdf

McManamay RA, Troia MJ, DeRolph CR, Olivero Sheldon A, Barnett AR, Kao S-C, Anderson MG. 2018. A stream classification system to explore the physical habitat diversity and anthropogenic impacts in riverscapes of the eastern United States. PLOS ONE 13(6):e0198439. https://doi.org/10.1371/journal.pone.0198439

Pitlick J, Wilcock P. 2001. Relations between streamflow, sediment transport, and aquatic habitat in regulated rivers. Geomorphic Processes and Riverine Habitat, Water Science and Application 4. https://agupubs.onlinelibrary.wiley.com/doi/pdf/10.1029/WS004p0185.

Poff NL, Allan JD, Bain MB, Karr JR, Prestegaard KL, Richter BD, Sparks RE, Stromberg JC. 1997. The natural flow regime. BioScience 47(11):769-84.

Poff NL, Brinson MM, Day JW. 2002. Aquatic ecosystems and global climate change: potential impactson inland freshwater and coastal wetland ecosystems in the United States. Pew Center on Global Climate Change.

Poff NL, Zimmerman JKH. 2010. Ecological responses to altered flow regimes: a literature review to inform

the science and management of environmental flows. Freshwater Biology 55:194-205. doi:10.1111/j.1365-2427.2009.02272.x

Strayer DL, Downing JA, Haag WR, King TL, Layzer JB, Newton TJ, Nichols JS. 2004. Changing perspectives on pearly mussels, North America's most imperiled animals. BioScience 54(5):429-39.

U.S. Environmental Protection Agency (EPA). 2016. National Rivers and Streams Assessment 2008-2009: A Collaborative Survey (EPA/841/R-16/007). Office of Water and Office of Research and Development. Washington, DC. March 2016. https://www.epa.gov/sites/production/files/2016-03/documents/nrsa_0809_march_2_final.pdf

Walter RC, Merritts DJ. 2008. Natural streams and the legacy of water-powered mills. Science 319(5861):299-304.

Wharton G, Mohajeri SH, Righetti M. 2017. The pernicious problem of streambed colmation: a multi-disciplinary reflection on the mechanisms, causes, impacts, and management challenges. Wiley Interdisciplinary Reviews: Water 4(5):e1231.

Riverine Water Column

System: Riverine

Subsystem: Tidal & Non-tidal

Class: Water Column

Sub-class: Well-mixed

Geographic Area: Entire Area

Overall Vulnerability Rank = High

Habitat Sensitivity = High

Climate Exposure = High

	Riverine Water Column	Attribute Mean	Data Quality	Distribution of Expert Scores
Sensitivity Attributes	Habitat condition	2.6	2.9	
	Habitat fragmentation	3	3	
	Distribution/Range	1.2	2.2	
	Mobility/Ability to spread or disperse	3	2.6	
	Resistance	1.8	2.2	
	Resilience	1.2	2.2	
	Sensitivity to changes in abiotic factors	3	2.2	
	Sensitivity and intensity of non−climate stressors	2.8	1.4	
	Dependency on critical ecological linkages	2.4	2	
	Sensitivity Component Score	High		
Exposure Factors	Sea surface temp	n/a	n/a	
	Bottom temp	n/a	n/a	
	Air temp	n/a	n/a	
	River temp	3.2	2.2	
	Surface salinity	n/a	n/a	
	Bottom salinity	n/a	n/a	
	pH	n/a	n/a	
	Sea level rise	1.8	2.2	
	Precipitation	n/a	n/a	
	Floods	3	1.9	
	Droughts	3	1.9	
	Exposure Component Score	High		
**Overall Vulnerability Rank**		**High**		

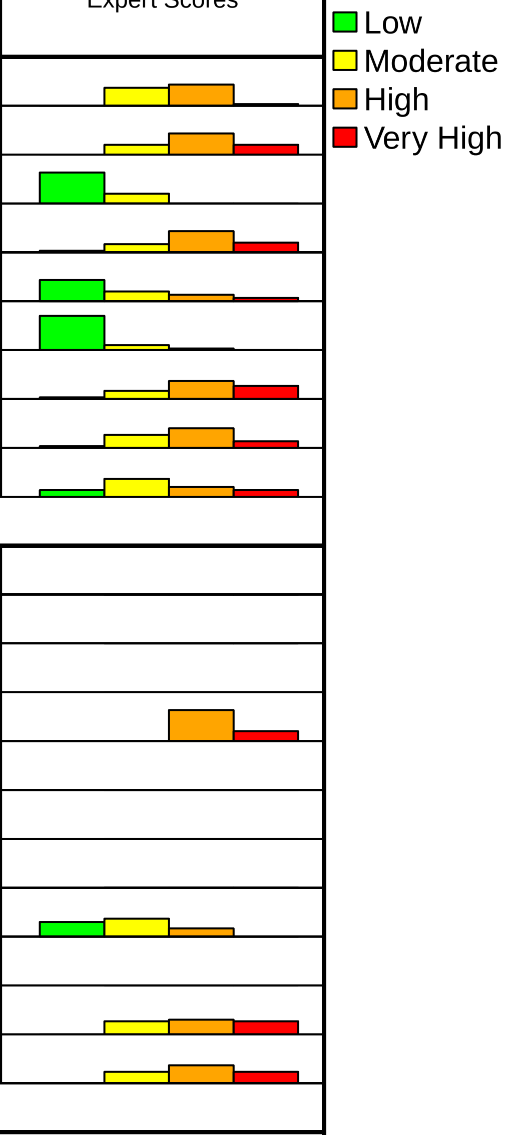

Habitat Name: Riverine Water Column

System: Riverine

Subsystem: Tidal and Non-tidal

Class: Water Column

Sub-class: Well-mixed

Geographic Area: Entire Area

Habitat Description: This sub-class includes the 3-dimensional space of water for both tidal and non-tidal zones in riverine systems. The water column includes the physical (e.g., temperature, salinity, light penetration) and chemical (e.g., pH, dissolved oxygen, nutrients, salts), and biological (e.g., zooplankton, phytoplankton) components of the water, but not the river bottom/banks, submerged vegetation, or emergent and riparian vegetation. For the purposes of this study, the riverine water column habitat terminates at the downstream end where the concentration of ocean-derived salts in the water ≥ 0.5 ppt during the period of annual average low flow, or where the channel enters a lake. The water column habitat can be highly dynamic and exhibit swift responses to environmental variables, such as air temperature and precipitation. However, some rivers and streams do not respond quickly to rain events and many rivers with a high proportion of groundwater inputs do not show rapid temperature swings (Mohseni 1998; Snyder et al. 2015).

Overall Climate Vulnerability Rank: High (49% of the bootstrap results match the results of the categorical vulnerability rank, but 51% of the bootstrap results were in the Moderate vulnerability rank indicating that the vulnerability is on the borderline between the High and Moderate vulnerability ranks.

Climate Exposure: High. The overall High exposure score was influenced by high attribute means in three of the four exposure factors: River Temperature (3.2), Floods (3.0), and Drought (3.0). The exposure attribute scores for Sea Level Rise (SLR) was 1.8, and the scores were spread over Low, Moderate, and High scoring bins. This likely reflects the differential effect that SLR may have between tidal and non-tidal sections of riverine habitats. The scores for both Floods and Droughts were spread over the Moderate, High, and Very High scoring bins, which likely reflects the large range of projected changes in precipitation that affect floods and droughts over the study area.

Habitat Sensitivity: High. The overall High sensitivity score was influenced by four of the nine sensitivity attributes scoring ≥2.8: Habitat Fragmentation, Mobility/Ability to Spread or Disperse, Sensitivity to Changes in Abiotic Factors, and Sensitivity and Intensity of Non−Climate Stressors. The means for the other sensitivity attributes ranged from 2.6 to 1.2. The higher sensitivity scores for Habitat Fragmentation and Mobility/Ability to Spread or Disperse likely reflects the effects of dams and other water diversion structures that alter the water column. The higher scores for Sensitivity to Changes in Abiotic Factors and Sensitivity and Intensity of Non−Climate Stressors likely reflect the sensitivity of the water column to changes to physical properties (e.g., water temperature) and other anthropogenic activities (e.g., urbanization, stormwater discharge).

Data Quality & Gaps: The data quality scores for the four climate exposure factors were split between 1.9 and 2.2. The relatively low data quality scores for the exposure factors was likely due to the high degree of geographic variability in the projected changes for precipitation that influences floods and droughts.

For habitat sensitivity, only two attributes had data quality scores >2.2: Habitat Condition (2.9) and Mobility/Ability to Spread or Disperse (2.6). These relatively low data quality scores likely reflect uncertainty by the scorers in understanding the sensitivity of the water column to climate and non-climate stressors. This may be viewed as a data gap, but also the complexity of separating multiple anthropogenic stressors present in the riverine water column.

Positive or Negative Climate Effect for the Northeast U.S.: The effect of climate change on the riverine water column in the study area is expected to be negative (70% of the experts' scores were negative while 30% of the scores were neutral). This may be a reflection of variability in the projected climate exposure of riverine habitats throughout the study area, as well as uncertainties in the sensitivity of riverine water column

habitat to climate and non-climate stressors.

<u>Climate Effects on Habitat Condition and Distribution</u>: The water column is an important component of riverine ecosystems for fish, invertebrates, and biotic habitats. Characteristics of water column habitat include temperature, salinity, dissolved oxygen, carbonate chemistry, nutrients, and primary and secondary producers. Characteristics also include water flow dynamics and sedimentation/turbidity.

The riverine water column is very dynamic and impacted by air-water exchange, inputs from terrestrial environments through freshwater inflows, groundwater inputs, and by estuarine and marine waters near the mouths of rivers through mixing with salt water. Riverine water sources include headwaters that can be many miles inland of the coast, groundwater, rainfall and stormwater runoff, and from snow and ice melt in the winter and spring in New England rivers.

Water temperature in rivers is influenced by heat exchange with the atmosphere, although other factors play a role including urbanization (e.g., large impervious surfaces, thermal discharges), deforestation, damming, groundwater inputs (Snyder et al. 2015), and geography, also contribute (Kaushal et al. 2010). Correlations between air temperatures and lake, river, and stream water temperatures are well established (Livingstone 1997; IPCC 2001; Huntington et al. 2003; Kaushal et al. 2010; Vaughan et al. 2013; Rice and Jastram 2015).

Significant increases in stream and river temperatures have been reported in most regions of the United States, with the highest observed rates of warming in the more urbanized areas of the Mid-Atlantic (Kaushal et al. 2010). Other measures of river and stream temperature conditions in the northeast are changing, including ice thickness (Huntington et al. 2003), dates of spring ice-out and ice-affected flow (Hodgkins et al. 2002; Hodgkins et al. 2003; Hodgkins and Dudley 2006), and seasonal stream runoff volume (Hodgkins et al. 2005). Stream temperatures are projected to increase in the study area by the end of the century (Letcher et al. 2016). However, local areas with a high proportion of groundwater input may provide important thermal refugia to increasing temperatures, an important factor that otherwise may not be well-captured by regional modeling approaches (Snyder et al. 2015).

While annual precipitation has shown a shift towards greater variability and higher totals across the entire United States since 1970, extreme precipitation events have been the highest on the east coast compared to other regions (Karl and Knight 1998; Walsh et al. 2014; Sun and Lall 2015). Changes in precipitation patterns are related to a combination of factors, including cyclic atmospheric variability and long-term trends related to climate warming. Substantial increases in streamflow volume (i.e., storm flows) can alter the hydrodynamic conditions in rivers and streams, which can have deleterious effects on water quality through erosion. However, increases in base flow volume can have beneficial effects on water quality.

<u>Habitat Summary</u>: According to the National Rivers and Streams Assessment 2008–2009 Report, river and stream water quality in the study area is generally considered fair (EPA 2016). About 30-40% of all stream lengths were rated as poor in terms of nitrogen and phosphorus pollution (EPA 2016).

Extreme precipitation events can increase streamflow volume and alter the hydrodynamic conditions in rivers and streams that negatively impacts riverine water column water quality. Heavy precipitation events can cause compounding or synergistic effects, such as increased sediment erosion and stormwater runoff, overloading of municipal or agricultural waste systems, and large increases in stream flow. These events may also cause physical impacts, such as scouring and erosion of unconsolidated sediments, which can increase suspended sediments and turbidity in the water column (McCluney 2014).

Flood trend magnitudes are highly correlated with the amount of basin urbanization (Villarini and Smith 2010; Hodgkins et al. 2019) and the regulation of flows (i.e., dams). Dams impede the natural flow of rivers and streams and have degraded water quality across the study area by increasing temperatures, reducing dissolved oxygen, and concentrating pollutants in the head ponds and reservoirs (Hall et al. 2011; Hall et al. 2012; Mattocks et al. 2017). The eastern U.S. ecoregion contains only 40–50% free-flowing rivers (Liermann et al. 2012) and there are over 200,000 river and stream barriers, including road-crossing crossings and dams, in the

13-state region from Maine and Virginia (Northeast Aquatic Connectivity Project 2019). Large dams with deep reservoirs can alter downstream and upstream temperature and other water quality factors. Although large reservoir dams are not common in the coastal region, they are present in more inland areas of the study area. The majority of dams in the coastal areas of the study are small, run-of-river dams and have minor impact on flow quantity and timing downstream, and generally only affect the water column in their impoundments. The influence of dams are reported to have greater hydrological and ecological impacts on rivers than changes in precipitation due to climate change (Magilligan et al. 2016).

By altering the natural flow of water, dams, culverts, and other water restrictions fragment riverine habitat and restrict access to the water column for organisms, particularly migrating diadromous fish (Hall et al. 2011; Hall et al. 2012; Mattocks et al. 2017). However, over the past few decades there has been a trend of dam removal and fish access projects (e.g., fish ladders) in the study area to improve fish passage (American Rivers 2019). This trend is expected to increase in the future as many dams will exceed their design life, and become vulnerable to failure.

Salinization of freshwater systems related to urbanization and increasing run-off, typically caused by introduction of road salting in winter months, can be a concern for some rivers and streams (Daley 2009). Riverine habitats near the mouths of rivers can become more saline, as the salt wedge moves further upstream due to sea level rise.

Literature Cited

American Rivers. 2019. [Internet]. Raw Dataset –ARDamRemovalList_figshare_2017. Figshare. Accessed 27 June 2021. https://doi.org/10.6084/m9.figshare.5234068.v5.

Daley ML, Potter JD, McDowell WH. 2009. Salinization of urbanizing New Hampshire streams and groundwater: effects of road salt and hydrologic variability. Journal of the North American Benthological Society 28(4):929-40.

Hall CJ, Jordaan A, Frisk MG. 2011. The historic influence of dams on diadromous fish habitat with a focus on river herring and hydrologic longitudinal connectivity. Landscape Ecology 26(1):95-107.

Hall CJ, Jordaan A, Frisk MG. 2012. Centuries of anadromous forage fish loss: consequences for ecosystem connectivity and productivity. BioScience 62(8):723-31.

Hodgkins GA, Dudley RW. 2006. Changes in the timing of winter–spring streamflows in eastern North America, 1913–2002. Geophysical Research Letters 33(6):1-5.

Hodgkins GA, James IC, Huntington TG. 2002. Historical changes in lake ice-out dates as indicators of climate change in New England, 1850-2000. International Journal of Climatology 22(15):1819-27.

Hodgkins GA, Dudley RW, Huntington TG. 2003. Changes in the timing of high river flows in New England over the 20th Century. Journal of Hydrology 278(1-4):244-52.

Hodgkins GA, Dudley RW, Huntington TG. 2005. Changes in the number and timing of days of ice-affected flow on northern New England rivers, 1930–2000. Climatic Change 71(3):319-40.

Hodgkins G, Dudley R, Archfield S, Renard B. 2019. Effects of climate, regulation, and urbanization on historical flood trends in the United States. Journal of hydrology 573:697-709. doi:10.1016/j.jhydrol.2019.03.102.

Huntington TG, Hodgkins GA, Dudley RW. 2003. Historical trend in river ice thickness and coherence in hydroclimatological trends in Maine. Climatic Change 61(1):217-36.

Intergovernmental Panel on Climate Change (IPCC). 2001. Climate change 2001. The scientific basis. Contribution of Working Group I to the Third Assessment Report of the Intergovernmental Panel on Climate Change. [Houghton J, Ding Y, Griggs D, Noguer M, van der Linden P, Dai X, Maskell K, Johnson C (eds)]. Intergovernmental Panel on Climate Change. 1-881.

Karl TR, Knight RW. 1998. Secular trends of precipitation amount, frequency, and intensity in the United States. Bulletin of the American Meteorological Society 79(2):231-42.

Kaushal SS, Likens GE, Jaworski NA, Pace ML, Sides AM, Seekell D, Belt KT, Secor DH, Wingate RL. 2010. Rising stream and river temperatures in the United States. Frontiers in Ecology and the Environment 8(9):461-6.

Letcher BH, Hocking DJ, O'Neil K, Whiteley AR, Nislow KH, O'Donnell MJ. 2016. A hierarchical model of daily stream temperature using air-water temperature synchronization, autocorrelation, and time lags. *PeerJ* 4:e1727. doi:10.7717/peerj.1727.

Liermann CR, Nilsson C, Robertson J, Ng RY. 2012. Implications of dam obstruction for global freshwater fish diversity. BioScience 62(6):539-48.

Livingstone DM. 1997. Break-up dates of alpine lakes as proxy data for local and regional mean surface air temperatures. Climatic Change 37(2):407-39.

Magilligan FJ, Graber BE, Nislow KH, Chipman JW, Sneddon CS, Fox CA. 2016. River restoration by dam removal: Enhancing connectivity at watershed scales. Elementa: Science of the Anthropocene 4:1-14.

Mattocks S, Hall CJ, Jordaan A. 2017. Damming, lost connectivity, and the historical role of anadromous fish in freshwater ecosystem dynamics. BioScience 67(8):713-28.

McCluney KE, Poff NL, Palmer MA, Thorp JH, Poole GC, Williams BS, et al. 2014. Riverine macrosystems ecology: sensitivity, resistance, and resilience of whole river basins with human alterations. Frontiers in Ecology and the Environment 12(1):48-58.

Mohseni O, Stefan HG, Erickson TR. 1998. A nonlinear regression model for weekly stream temperatures. Water Resources Research 34(10):2685-92.

Northeast Aquatic Connectivity project (2019). [Internet]. (Accessed 27 June 2021). https://www.conservationgateway.org/ConservationByGeography/NorthAmerica/UnitedStates/edc/reportsdata/freshwater/stream/Pages/default.aspx.

Rice KC, Jastram JD. 2015. Rising air and stream-water temperatures in Chesapeake Bay region, USA. Climatic Change 128(1-2):127-38.

Snyder CD, Hitt N,P Young JA. 2015. Accounting for groundwater in stream fish thermal habitat responses to climate change. Ecological Applications 25: 1397-1419. doi:10.1890/14-1354.1

Sun X, Lall U. 2015. Spatially coherent trends of annual maximum daily precipitation in the United States. Geophysical Research Letters 42(22):9781-9.

U.S. Environmental Protection Agency (EPA). 2016. National Rivers and Streams Assessment 2008-2009: A Collaborative Survey (EPA/841/R-16/007). Office of Water and Office of Research and Development. Washington, DC. March 2016. https://www.epa.gov/sites/production/files/2016-03/documents/nrsa_0809_march_2_final.pdf.

Vaughan DG, Comiso JC, Allison I, Carrasco J, Kaser G, Kwok R, Mote P, Murray T, Paul F, Ren J, Rignot E,

Solomina O, Steffen K, Zhang T. 2013. Observations: cryosphere. Climate change 2013. The physical science basis. Contribution of Working Group I to the Fifth Assessment Report of the Intergovernmental Panel on Climate Change. [Stocker TF, D. Q, Plattner G-K, Tignor M, Allen SK, Boschung J, Nauels A, Xia Y, Bex V, Midgley PM (eds)]. Intergovernmental Panel on Climate Change.p. 317-82.

Villarini G, Smith JA. 2010. Flood peak distributions for the eastern United States. Water Resources Research 46:1-17. W06504, doi:10.1029/2009WR008395.

Walsh J, Wuebbles D, Hayhoe K, Kossin J, Kunkel K, Stephens G, Thorne P, Vose R, Wehner M, Willis J. 2014. Chapter 2: our changing climate. Climate change impacts in the United States. The Third National Climate Assessment. [Melillo JM, Richmond TC, Yohe GW (eds)]. U.S. Global Change Research Program. p. 19-67.
